# Straight From the Plastome: Molecular Phylogeny and Morphological Evolution of *Fargesia* (Bambusoideae: Poaceae)

**DOI:** 10.3389/fpls.2019.00981

**Published:** 2019-08-06

**Authors:** Yun Zhou, Yu-Qu Zhang, Xiao-Cheng Xing, Jian-Qiang Zhang, Yi Ren

**Affiliations:** College of Life Sciences, Shaanxi Normal University, Xi’an, China

**Keywords:** *Fargesia*, inflorescences, phylogeny, plastome, spathe-like leaf sheath syndrome, *Yushania*

## Abstract

*Fargesia* is ecologically and economically important in mountainous forests. Many *Fargesia* species are also important sources of food for some endangered animals such as the giant panda. Recent molecular phylogenetic analyses have revealed *Fargesia* as a polyphyletic group despite some unclear lineage affinities. In the present study, we reconstructed the phylogeny of *Fargesia* and its allies, including *Thamnocalamus, Arundinaria* (incl. *Bashania*), *Yushania, Indocalamus, Ampelocalamus* and *Phyllostachys*, from a plastome sequence matrix that contained 20 *Fargesia* and five *Yushania* species as ingroups, 16 species from nine other bamboo genera plus *Oryza sativa* and *Zea mays* as outgroups. *Fargesia* and its allies were broken into eight clades. Several *Fargesia* species were clustered into the *Thamnocalamus* clade and the *Drepanostachyum* + *Himalayacalamus* clade that rendered the polyphyly of *Fargesia*. The remaining six clades, including the *Fargesia* spathe clade, the *Phyllostachys* clade, *Arundinaria fargesii*, the *Ampelocalamus* clade, the *Fargesia grossa* clade, and the *Fargesia macclureana* clade, were identified. Molecular phylogenetic analyses supported that *Yushania* should be included in *Fargesia* (s.l.) which had synapomorphy of expanded leaf sheaths in varying degree at the basis of inflorescences, and further divided into the *Fargesia* spathe clade, the *Fargesia grossa* clade, and the *Fargesia macclureana* clade. All sampled species of *Yushania* were nested within the *Fargesia grossa* clade. The probable model of the origin of the species in the *Fargesia* spathe clade with spathe-like leaf sheath syndrome was proposed. Moreover, the formation of the spathe-like leaf sheath syndrome may be correlated with cold climatic conditions in Quaternary. Our results provide new sight into the phylogenetic relationship within *Fargesia*.

## Introduction

Bamboos, especially the woody bamboos, are notoriously difficult for identification and classification due to diversified morphology of vegetative characters and flowering intervals as long as 10–120 or even up to 150 years (McClure, 1966; Janzen, 1976; Ramanayake and Weerawardene, 2003; Stapleton et al., 2009; Attigala et al., 2016; Zhang et al., 2019). Previous phylogenetic studies have shown that the temperate woody bamboos are monophyletic (Hodkinson et al., 2010; Triplett and Clark, 2010; Bamboo Phylogeny Group, 2012; Saarela et al., 2018), although the woody bamboos as a whole are paraphyletic (Triplett and Clark, 2010; Bamboo Phylogeny Group, 2012; Triplett et al., 2014). Molecular phylogenies are often incongruent with morphologically-based classification schemes, leading to difficulty in understanding the evolution of morphological traits in many bamboo clades (Triplett and Clark, 2010; Triplett et al., 2014; Attigala et al., 2016). For example, the tribe Arundinarieae have been divided into 12 or 13 major lineages based on plastid or nuclear DNA markers, respectively, for which no morphological synapomorphies have been identified (Zhang et al., 2012; Attigala et al., 2014, 2016; Ma et al., 2014). At the generic level, the problem is more severe, where morphological intermediacy, insufficient informative characters, and hybridization/introgression contribute to the lack of agreement between morphological based classification schemes and recent molecular phylogenies (Triplett and Clark, 2010; Zhang et al., 2012; Yang et al., 2013).

*Fargesia* Franch. was considered to be a distinct genus in tribe Arundinarieae (Clayton and Renvoize, 1986; Soderstrom and Ellis, 1987; Keng, 1992; Yi, 1996; Li, 1997; Ohrnberger, 1999; Yi et al., 2008; Triplett and Clark, 2010; Bamboo Phylogeny Group, 2012; Kellogg, 2015) based on vegetative morphological traits (Wang and Ye, 1981; Yi, 1982, 1988a, 1996; Keng, 1983; McNeely, 1999; Guo et al., 2001, 2002; Guo and Li, 2004; Li et al., 2006; Yi et al., 2008). *Fargesia* species play an important role in the subtropical ecosystem and also as the main food of the giant panda and some other endangered animals (Yi, 1985a, 1996; McNeely, 1999; Guo and Li, 2004; Yi et al., 2008). The species of *Fargesia* share the following diagnostic characters: pachymorph rhizomes with a short neck, 7–15 branches per node on culm, racemose or paniculate inflorescences, ebracteate, inflorescences subtended by a series of delicate or spathe-like sheaths, and pistils with two or three stigmas (Franchet, 1893; Wang and Ye, 1981; Yi, 1982, 1983b, 1988a, 1996; Keng, 1987; Li et al., 2006; Yi et al., 2008; Kellogg, 2015). However, due to considerable morphological diversity and complex evolutionary history of bamboos, *Fargesia* had faced a protracted period of taxonomic uncertainty on the generic boundary, with some species transferred into or out of other genera of Sino-Himalayan pachymorph-rhizomed bamboos based on morphological evidence, including *Thamnocalamus* (Soderstrom, 1979a,b; Soderstrom and Ellis, 1982; Clayton and Renvoize, 1986; Chao and Renvoize, 1989; Demoly, 1991; Chao and Tang, 1993; Stapleton, 1994a; Li et al., 2006), *Yushania* (Soderstrom and Ellis, 1987; Stapleton, 1994a), *Borinda* (Stapleton, 1994a; Stapleton et al., 2005), *Drepanostachyum* (Stapleton, 1994b; Li, 2003; Li et al., 2006; Stapleton et al., 2005) or *Himalayacalamus* (Stapleton, 1994b; Ohrnberger, 1996; Li et al., 2006) and the leptomorph-rhizomed genus *Arundinaria* (Soderstrom and Ellis, 1988; Stapleton, 1994a; Li et al., 2006). Previous studies suggested that the spathe-like leaf sheaths subtending at the basis of inflorescences (Yi, 1988a) and the number of stigmas may be useful for delimitation of *Fargesia* and other alpine bamboos (Guo et al., 2002; Guo and Li, 2004), but the phylogenetic relationships among them are still unclear. Subsequently, *Borinda* was treated as a synonym for *Fargesia* based on nuclear phylogenies (Guo et al., 2001, 2002; Guo and Li, 2004). *Fargesia* species were nested within the *Phyllostachys* clade of tribe Arundinarieae which included more than 15 genera based on multi-locus plastid markers or complete chloroplast genome (Triplett and Clark, 2010; Zeng et al., 2010; Attigala et al., 2014; Ma et al., 2014; Zhang et al., 2016). However, by adding the nuclear markers and multiplying the sampling size, the phylogenetic relationship between *Fargesia* and closely taxa, including *Yushania* (Hodkinson et al., 2010; Wang et al., 2017; Zhang et al., 2019), *Phyllostachys* (Ma et al., 2014; Wang et al., 2017) *Chimonocalamus* (Yang et al., 2013; Wang et al., 2017), *Bashania* (Zeng et al., 2010; Yang et al., 2013; Wang et al., 2017), *Thamnocalamus* (Guo et al., 2002; Guo and Li, 2004; Zhang et al., 2019), *Drepanostachyum* (Wang et al., 2017; Zhang et al., 2019), and *Himalayacalamus* (Wang et al., 2017; Zhang et al., 2019), could not be resolved. For instance, *Phyllostachys* species were sister to *Fargesia* based on plastomes (Ma et al., 2014; Attigala et al., 2016), but it had no affinity with *Fargesia* in the RAD-seq data (Wang et al., 2017).

The infrageneric classification of *Fargesia* was established mainly based on vegetative characteristics including the shape of buds and culm sheath blades, and the number and thickness of branches (Yi, 1988a). However, high rates of morphological divergence may result in unstable generic/infrageneric classifications due to parallel evolution or morphological homoplasy (Wei et al., 2018), which prevail in vegetative traits of bamboos (Guo and Li, 2004; Attigala et al., 2016; Zhang et al., 2019). Therefore, it needs to be tested whether the current morphologically based infrageneric classification of *Fargesia* (Yi, 1988a, 1996; Yi and Jiang, 2010; Bamboo Phylogeny Group, 2012) is credible. Recent systematic studies revealed that the *Fargesia + Yushania* clade based on RAD-data (Wang et al., 2017) or plastid regions and nrITS (Zhang et al., 2019), including most *Fargesia* species and all sampled *Yushania* species, could be further divided into the *Fargesia* spathe clade and the non-spathe clade according to their morphological traits of inflorescences and distribution rather than the key vegetative characteristics listed by Yi (1988a) for the subdivision of *Fargesia* (Zhang et al., 2019). Moreover, the species in the *Fargesia* spathe clade shared a spathe-like leaf sheath syndrome, i.e., a series of spathe-like leaf sheaths that surround compressed inflorescences with the top spathe generally longer than the inflorescences and the spikelets exserted partially and uni-laterally, which was hypothesized to be an adaptation to cold environments in the north (Zhang et al., 2019). However, the phylogenetic relationships among major subclades of the non-spathe clade including the rest of *Fargesia* species and all sampled *Yushania* species are still poorly understood, with species from both genera being intermingled with other taxa such as *Arundinaria, Indocalamus*, and *Chimonocalamus* (Ma et al., 2014; Wang et al., 2017).

Sparse taxon sampling and insufficient informative characters had been the problems for investigating the phylogeny of *Fargesia*. Thus, a high-quality phylogeny, including more representative taxa and a large number of genetic markers, is necessary to understand the evolutionary history of *Fargesia*. Full plastome sequencing has been shown to be useful in resolving complex evolutionary relationships in closely related species (Njuguna et al., 2013; Williams et al., 2016; Uribe-Convers et al., 2017; Fonseca and Lohmann, 2018). Despite the existence of previous phylogenetic analyses based on the plastomes of Arundinarieae (Ma et al., 2014; Attigala et al., 2016; Saarela et al., 2018), the phylogenetic relationships among *Fargesia* species are still poorly resolved. Our aims were to (1) test the monophyly of *Fargesia*; (2) establish a phylogenetic framework for *Fargesia* and closely related taxa; (3) evaluate the utility of whole chloroplast genome sequencing in providing a high-resolution estimation of the infrageneric phylogeny within *Fargesia*.

## Materials and Methods

### Taxon Sampling

Three to five fresh leaf samples from each individual (clumps) were collected in their native habitats and dried in silica gel and stored at room temperature for DNA extraction (Table 1). Twenty-six species, including the type species for each section and series in Yi’s (1988a) classification of *Fargesia*, were selected for Illumina sequencing. We also included the representatives of the major subclades in the *Fargesia* + *Yushania* clade based on the previous study by Zhang et al. (2019) and outgroups representing genera within the *Phyllostachys* clade of the Arundinarieae (since their phylogenetic relationship is unclear with *Fargesia*; see Ma et al., 2014) (Table 1). All vouchers were deposited in the Herbarium of Shaanxi Normal University (SANU, Xi’an, China). Seventeen previously published plastomes were downloaded from the NCBI GenBank database^1^ (Table 1).

**Table 1:** General features of plastomes used by the present study.

	Aligned
	paired-end	Mean				Genbank
Species	reads	coverage	Size (bp)	Voucher specimen	Source	accession
*Fargesia decurvata*^∗^	140,613	150.8	139,642	Zhang Yu-QuA120	Yangxian, Shaanxi	MH988719
*Fargesia denudata*^∗^	92,258	99.9	139,612	Zhang Yu-QuC205	Pingwu, Sichuan	MH988724
*Fargesia funiushanensis*^∗^	70,977	76.8	139,622	Zhang Yu-QuE612	Luanchuan, Henan	MH988730
*Fargesia nitida*	–	–	139,535	–	–	JX513416
*Fargesia qinlingensis*^∗^	164,596	176.1	139,626	Zhang Yu-QuA071	Foping, Shaanxi	MH988720
*Fargesia stenoclada*^∗^	236,512	254.5	139,622	Xing Xiao-ChengC741	Pengzhou, Sichuan	MH988723
*Fargesia spathacea*	–	–	139,767	–	–	JX513417
*Fargesia albocerea*^∗^	157,916	170.2	139,617	Zhang Yu-QuD588	Gongshan, Yunnan	MH988728
*Fargesia canaliculata*^∗^	185,351	200.2	139,606	Zhang Yu-QuC305	Mianning, Sichuan	MH988725
*Fargesia communis*^∗^	223,346	237.6	139,605	Zhang Yu-QuD540	Yuling, Yunnan	MH988721
*Fargesia edulis*^∗^	35,963	39.4	139,572	Zhang Yu-QuD418	Lushui, Yunnan	MH988724
*Fargesia fungosa*^∗^	52,788	59.3	139,582	Zhang Yu-QuD445	Huize, Yunnan	MH988726
*Fargesia hygrophila*^∗^	78,247	84.8	139,602	Zhang Yu-QuD552	Dali, Yunnan	MH988727
*Fargesia macclureana*^∗^	818,614	934.4	139,653	Zhang Yu-QuG695	Linzhi, Tibet	MH988729
*Fargesia grossa*^∗^	147,441	159	139,601	Zhang Yu-QuG687	Cuona, Tibet	MH988734
*Fargesia yunnanensis*	–	–	139,609	–	–	JX513418
*Fargesia damuniu*^∗^	296,654	278.1	139,652	Zhang Yu-QuG660	Nielamu, Tibet	MH988732
*Thamnocalamus spathiflorus*	–	–	139,778	–	–	JX513425
*Fargesia gyirongensis*^∗^	169,148	182.1	139,592	Zhang Yu-QuG673	Jilong, Tibet	MH988733
*Fargesia collaris*^∗^	104,339	112.7	139,515	Zhang Yu-QuG650	Zhangmu, Tibet	MH988731
*Fargesia* sp.^∗^	77,371	83.5	139,591	Xing Xiao-ChengC744	Tianquan, Sichuan	MH988722
*Yushania brevipaniculata*^∗^	45,980	50.2	139,568	Zhang Yu-QuC365	Wolong, Sichuan	MH988738
*Yushania confusa*^∗^	136,260	180.3	139,601	Zhang Yu-QuF642	Changyang, Hubei	MH988737
*Yushania glandulosa*^∗^	163,765	176.4	139,621	Zhang Yu-QuD453	Lushui, Yunnan	MH988740
*Yushania violascens*^∗^	105,310	113.8	139,605	Zhang Yu-QuC362	Xianggelila, Yunnan	MH988739
*Yushania levigata*	–	–	139,633	–	–	JX513426
*Ampelocalamus calcareus*^∗^	104,074	112.7	139,666	Zhang Yu-QuH712	Libo, Guizhou	MH988715
*Ampelocalamus melicoideus*^∗^	194,815	711.7	139,552	Zhang Yu-QuI702	Nanchuan, Chongqing	MH988718
*Ampelocalamus saxatilis*^∗^	314,976	338	139,618	Zhang Yu-QuC396	Xuyong, Sichuan	MH988717
*Arundinaria fargesii*^∗^	146,231	157	139,660	Zhang Yu-QuA117	Foping, Shaanxi	MH988716
*Pleioblastus amarus*^∗^	101,327	109.6	139,703	Zhang Yu-QuC373	Chengdu, Sichuan	MH988736
*Pleioblastus maculatus*	–	–	139,720	–	–	JX513424
*Arundinaria faberi*			139,629	–	–	JX513414
*Chimonocalamus longiusculus*	–	–	139,821	–	–	JX513415
*Indocalamus longiauritus*			139,668	–	–	HQ337795
*Phyllostachys edulis*	–	–	139,679	–	–	HQ337796
*Phyllostachys nigra* var. *henonis*	–	–	139,839	–	–	HQ154129
*Phyllostachys propinqua*	–		139,704	–	–	JN415113
*Phyllostachys sulphurea*	–	–	139,731	–	–	KJ722540
*Bambusa oldhamii*	–	–	139,350	–	–	FJ970915
*Dendrocalamus latiflorus*	–	–	139,371	–	–	FJ970916
*Oryza sativa*	–	–	134,502	–	–	KM103369
*Zea mays*	–	–	140,384	–	–	X86563

*^∗^Represent the newly assembled plastomes in this study.*

### Plastomes Sequencing, Assembly, and Annotation

Silica-dried leaf material was sent to the Center for Genetic and Genomic Analysis (Genesky Biotechnologies Inc., Shanghai) for library preparation and Illumina sequencing. Total genomic DNA was isolated using a DNeasy Plant Mini Kit (Qiagen, CA, United States). Short-insert (330 bp) libraries were constructed using the TruSeqTM DNA sample preparation kit (Illumina, United States) for Illumina HiSeq sequencing. We sequenced at least 2 GB of raw data for each bamboo species with an average read length of 150 bp. All of the sequencing reactions were conducted on the Illumina HiSeq 2 × 150 platform. The obtained raw reads were quality-trimmed using CLC Genomics Workbench v.8.5.1 (CLC Bio, Aarhus, Denmark)^2^ with default parameters. After trimming, high-quality paired-end reads were MIRA mapping assembly using MIRA v4.0.2 (Chevreux et al., 2004) to create initial reference sequences. These newly created reference sequences were baiting and iterative mapping with MITObim v1.8 (Hahn et al., 2013) to assemble the complete plastome sequences, and the previous published complete plastome sequences of *Fargesia spathacea* (GenBank no., JX513417), *Yushania levigata* (GenBank no., JX513426), *Pleioblastus maculatus* (GenBank no., JX513424), *Ampelocalamus calcareus* (GenBank no., KJ496369), and *Arundinaria fargesii* (GenBank no., JX513413) were used as seed reference. Raw reads were then remapped to newly assembled complete plastome sequences using Geneious v R9.0.2 (Kearse et al., 2012) under default parameters to check for misassemblies. Small gaps (46–193 bp) or ambiguous nucleotides in the remapped sequences were further confirmed by designed PCR amplifications (Supplementary Table S1) and sent to Sangon Biotech (Shanghai) Co., Ltd for Sanger sequencing. Gene annotations were assigned using DOGMA (Wyman et al., 2004). Inverted repeat (IR) boundaries were determined using the BLAST method (Altschul et al., 1990)^3^ and verified using Geneious v R9.0.2. The genome map of *Fargesia denudata* was drawn using the online program OGDRAW v 1.1 (Lohse et al., 2013).

### Data Partitioning and Alignment of Plastomes

We employed six datasets to reconstruct the phylogenetic trees: (1) the complete chloroplast genome sequences that excluded one copy of the IR region; (2) coding sequences including exons of protein-coding genes, tRNAs, and rRNAs (3) non-coding sequences including intergenic regions and introns; (4) the large single copy region (LSC), (5) the small single copy region (SSC) and (6) one inverted repeats region (IR). All 43 sequences from these six datasets were aligned using the MAFFT plugin (Katoh et al., 2002) in Geneious v.R9.0.2 with default parameters. As there is a negative effect of an increasing density of gap characters in multiple sequence alignments (Dwivedi and Gadagkar, 2009) and biases may be introduced when indels are included without precautions in Bayesian and Maximum Likelihood phylogenies (Warnow, 2012; Simmons, 2014), poorly aligned regions contained one or more gaps introduced by the alignments were excluded from the datasets (Attigala et al., 2016). Two different partitioning strategies were explored to access heterogeneity in the processes of molecular evolution for different sites in a sequence alignment. The first strategy included two partitions: coding sequences and non-coding sequences. The second strategy included three partitions: LSC, SSC, and one IR regions we then used PartitionFinder v 1.1.1 (Lanfear et al., 2012) to compare and evaluate partitioning schemes under 56 models (models = all) according to AICc values.

### Phylogenetic Analysis

We used DAMBE v6.4.29 to assess substitution saturation of the matrices before phylogenetic analyses (Xia et al., 2003; Xia and Lemey, 2009). Six datasets mentioned above with unpartitioning strategies were analyzed using Maximum likelihood (ML), Maximum parsimony (MP), and Bayesian inference (BI). ML analysis with rapid bootstrapping and 1000 replications (RAxML; Stamatakis et al., 2008) was performed using the General Time Reversible + gamma model (GTR-GAMMA model) via the CIPRES Science Gateway server (Miller et al., 2010). MP analysis was implemented in PAUP^∗^ 4.0b10 (Swofford, 2002) with 1000 random addition sequences and tree bisection-reconnection (TBR) branch swapping. A full heuristic bootstrap was conducted for MP with 1000 bootstrap replicates. The appropriate model for each dataset was selected according to AIC value (Posada and Crandall, 1998) implemented in jModelTest v2.1.4 (Darriba et al., 2012). GTR+I+G was found to be the most appropriate nucleotide substitution model for 43 complete chloroplast genome sequences, 41 complete chloroplast genome sequences, coding sequences, non-coding sequences, and LSC sequences; TVM+I G for SSC sequences; TVM+I for IR sequences; TIM1+I+G for 39 complete chloroplast genome sequences. BI analysis was performed using MrBayes v3.2.6 (Ronquist and Huelsenbeck, 2003). Two independent Markov chain Monte Carlo (MCMC) runs were executed, each with three heated and one cold chain for two million generations, sampling every 1000 generations, and a chain-heating temperature of 0.1. Twenty-five percent of trees were discarded as burn-in and the remaining samples were then summarized and a majority-rule consensus tree was constructed. Convergence of runs was also checked using Tracer v 1.6 (Rambaut et al., 2014) to assess the effective sample size (ESS) > 200 for all parameters.

### Topology Hypothesis Testing

Taxon removal experiments can aid in the investigation of whether distantly related outgroups have a biased attraction to long branches within the ingroups (Bergsten, 2005; Attigala et al., 2016). To test if outgroup selection affected the topology of the ingroups, we recovered phylogenetic trees with two datasets: (1) removing *Zea mays* and *Oryza sativa* to include only species of bamboos; (2) further removing species of Bambuseae to leave only species of *Fargesia* and its allies. Assessments of competing hypotheses (e.g., the monophyly of the *Fargesia* spathe and non-spathe clades) of the phylogeny were conducted with the approximately unbiased (AU) test (Shimodaira, 2002) as implemented in CONSEL (Shimodaira and Hasegawa, 2001). The site-wise log likelihoods for the trees were calculated using TREE-PUZZLE v5.3 (Schmidt et al., 2002). Neighbor-Net algorithm based on uncorrected *P*-distances was performed with SplitsTree v4.14.4 (Huson et al., 2008). To better visualize and evaluate the phylogenetic signal conflicts between ingroup taxa, we excluded all outgroup taxa in the SplitsTree analysis.

### Calibration and Estimation of the Divergence Time

A sequence matrix including 43 complete plastomes was created for divergence time dating. A molecular clock test was performed under the GTR model to assess the applicability of the strict molecular clock model. Our results showed that Δln*L* = 429 (*P* < 0.05), so the relaxed log-normal molecular clock was used. Divergence time dating was analyzed using BEAST v2.4.2 (Bouckaert et al., 2014) on the Cipres web server (Miller et al., 2010) with a Yule tree prior. We selected a Bambusoideae cf. *Chusquea* fossil (ca. 35 Mya) to calibrate the crown Bambusoideae (Strömberg, 2005). Runs were performed for 60 million generations, and trees were sampled every 1000 generations. Tracer v 1.6 (Rambaut et al., 2014) was used to check that all ESS values were > 200. The maximum clade credibility tree was generated by TreeAnnotator with mean node heights and 95% highest posterior density intervals. The temporal dynamics of the diversification of the major clades within *Fargesia* was visualized by constructing lineages through time (LTT) plots implemented by the APE package in R v.3.2.4 (Paradis et al., 2004).

### Morphological Character Evolution

Ancestral character state reconstruction was performed in Mesquite v 3.5 (Maddison and Maddison, 2018) using Likelihood ancestral state with Mk1 model (Lewis, 2001). The vegetative and the reproductive characters were then optimized on the BI tree inferred from the 41 plastome data. Information on morphological character states of inflorescences was generated based on published literature (Li et al., 2006; Yi et al., 2008; Zhang and Ren, 2016) and herbarium specimens in cases of the traits are missing in the published literature (Zhang Yu-QuE612, Zhang Yu-QuC305, Zhang Yu-QuA071) (Table 1 and Supplementary Table S2). These characters were (1) type of culm, (2) type of rhizomes, (3) type of leaf sheaths at the basis of inflorescences, (4) type of inflorescences, and (5) number of stigmas. Character states were scored as follows: Type of culm: (0) culm unicaepitose (clump forming due to rhizomes with short necks), (1) culm diffuse (spaced culm due to rhizomes with short necks); type of rhizomes: (0) sympodium (rhizomes pachymorph), (1) amphipodium, (2) monopodium (rhizomes leptomorph); type of leaf sheaths at the basis of inflorescences: (0) spathe-like leaf sheath syndrome, (1) open inflorescences with expanded leaf sheaths, (2) open inflorescences with non-expanded leaf sheaths, (3) absence of leaf sheaths at the basis of inflorescences; type of inflorescences: (0) semelauctant inflorescences, (1) iterauctant inflorescences; number of stigmas: (0) pistils with three stigmas, (1) two stigmas, (2) two or three stigmas.

## Results

### General Features of Plastomes

We sequenced and assembled 26 plastomes from *Fargesia* and its allies (Table 1). All sequenced genomes were similar in total size, structure, and gene content relative to previously published bamboo plastomes (Supplementary Figure S1) (Zhang et al., 2011; Burke et al., 2012; Wu and Ge, 2012; Ma et al., 2014; Attigala et al., 2016; Saarela et al., 2018). The size of the IR, large single copy (LSC) and small single copy (SSC) regions ranged from 21,793 bp (*Ampelocalamus calcareus*) to 21,813 (*F. communis*), 83,112 (*F. collaris*) to 83,418 (*Am. calcareus*), and 12,648 (*Am. calcareus*) to 12,882 (*F. damuniu*), respectively (Table 1). We identified 112 genes comprising 4 ribosomal RNAs, 31 transfer RNAs, and 77 protein-coding genes. Nineteen genes were duplicated in IR regions, and thus each chloroplast genome harbored 131 genes in total.

### Plastome Phylogenomics

The multiple sequence alignment of all 43 species (including both newly generated and previously published plastomes) was 124,853 bp after excluding one of the IR regions. After removal of alignment columns with gaps, the alignment length was reduced to 108,515 bp, including 8841 (8.15%) variable sites and 2187 (2.02%) parsimony-informative sites (Table 2 and Supplementary Table S3). The aligned sequences of six sequence datasets mentioned above were little saturated and were thus useful for phylogenetic analyses (Supplementary Table S4). The less partitioned model was found to be better than the highly partitioned model (Supplementary Table S5). The topologies estimated from the complete chloroplast sequences (Supplementary Figure S2F) were broadly similar to those estimated using the other datasets (Supplementary Figures S2A–E) but recovered relationships received much higher support values than those inferred from the five other datasets. Across these five datasets, the phylogenetic relationships inferred from the LSC sequences were largely resolved, while the SSC and IR datasets resolved only some of the lineages. Thus, we limited the discussion of phylogenetic trees estimated using partitioned analyses (Supplementary Figure S2).

**Table 2:** Data set characteristics, best substitution models and Maximum parsimony indices.

						Maximum parsimony		
		Number	Number of variable/			Tree	Consistency	Retention
Taxa	Data set	of sites	informative sites	GC (%)	Best fit model	length	index	index
43	LSC	76,310	7227/1773	37.3	GTR+I+G	8250	0.925	0.826
43	SSC	11,822	1209/334	33.7	TVM+I G	1418	0.932	0.858
43	IRs	20,610	421/83	44.4	TVM+I	655	0.9679	0.912
43	Coding	62273	3587/917	41.9	GTR+I+G	3997	0.937	0.865
43	Non-coding	48,162	5487/1334	34.7	GTR+I+G	6362	0.921	0.810
43	Data-complete	108,515	8841/2187	38.3	GTR+I+G	10092	0.927	0.831
41	Data-complete	115,500	3471/1788	38.1	GTR+I+G	3941	0.908	0.872
39	Data-complete	116,470	1978/523	38.0	TIM1+I+G	2291	0.887	0.794

**FIGURE 1:**
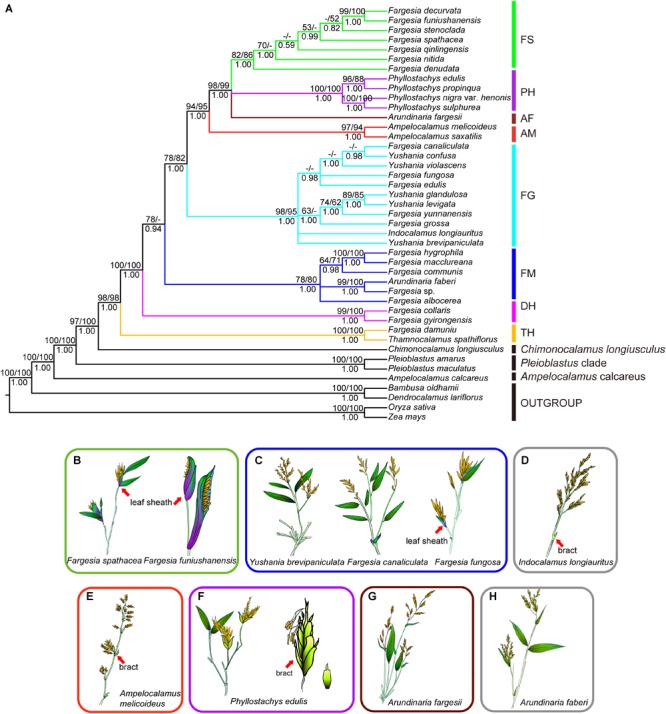
Phylogeny of *Fargesia* and its allies and floral morphology of representatives of major clades. **(A)** Phylogeny of 43 species of *Fargesia* and its allies inferred from Bayesian analysis of plastome sequences. Numbers above branches are ML bootstrap values/MP bootstrap values. Numbers below the branches are posterior probability (PP). Dashes represent nodes unresolved or without bootstrap support in the ML or MP trees or contradicted by the BI trees with PPs < 0.50. Roman numerals represent the revealed clades. FS, *Fargesia* spathe clade; PH, *Phyllostachys* clade; AF, *Arundinaria fargesii*; AM, *Ampelocalamus* clade; FG, *Fargesia grossa* clade; FM, *Fargesia macclureana* clade; DH, *Drepanostachyum* + *Himalayacalamus* clade; TH, *Thamnocalamus* clade. **(B)** *Fargesia spathacea* and *Fargesia funiushanensis*, **(C)** *Yushania brevipaniculata, Fargesia canaliculata*, and *Fargesia fungosa*, **(D)** *Indocalamus longiauritus*, **(E)** *Ampelocalamus melicoideus*, **(F)** *Phyllostachys edulis*, **(G)** *Arundinaria fargesii*, **(H)** *Arundinaria faberi*. Pictures of inflorescences are referred from Flora Republicae Popularis Sinicae (Yi, 1996), Flora of China (Li et al., 2006; Zhang and Ren, 2016).

In the phylogenetic tree based on complete plastid genome sequences (Figure 1A), *Bambusa oldhamii* and *Dendrocalamus latiflorus* was recovered as a clade (MLB/MPB/PP = 100/100/100; Figure 1A) sister to the remaining bamboo species. *Ampelocalamus calcareus* represent the earliest diverging lineage within Arundinarieae. Then *Pleioblastus* clade and *Chimonocalamus longiusculus* were recovered the distinct lineage, respectively. Eight major clades were recovered among *Fargesia* (Yi, 1988a) and its allies: the *Thamnocalamus* clade, the *Drepanostachyum* + *Himalayacalamus* clade, the *Fargesia macclureana* clade, the *Fargesia grossa* clade, the *Ampelocalamus* clade, *Arundinaria fargesii*, the *Phyllostachys* clade, and the *Fargesia* spathe clade (Figure 1A). The *Thamnocalamus* clade, which contained *F*. *damuniu* and *Thamnocalamus spathiflorus*, was supported by a high bootstrap value (MLB/MPB/PP = 100/ 100/1.00; Figure 1A). Similarly, high support (MLB/MPB/PP = 100/100/1.00; Figure 1A) was provided for *Drepanostachyum* + *Himalayacalamus* clade, which contained *F. gyirongensis* and *F. collaris*. The *Ampelocalamus* clade combined with the *Fargesia* spathe clade, the *Phyllostachys* clade, and *Arundinaria fargesii* to form a strongly supported monophyletic group (MLB/MPB/PP = 93/95/1.00; Figure 1A), but the phylogenetic relationships involving *Arundinaria fargesii*, the *Phyllostachys* clade, and the *Fargesia* spathe clade were not fully resolved. The *Fargesia* spathe clade, which including all species of *Fargesia* with the spathe-like leaf sheath syndrome (Figure 1B), is a monophyletic lineage with moderate support (MLB/MPB/PP = 82/86/1.00; Figure 1A), but the resolution among species within the clade was relatively low. The *Phyllostachys* clade, which contained *Ph. propinqua, Ph. edulis, Ph. nigra* var. *henonis*, and *Ph. sulphurea* showed strong support (MLB/MPB/PP = 100/100/1.00; Figure 1A). *Phyllostachys* species shows leptomorph rhizomes, inflorescences subtended by 2-keeled prophylls and 2–6 scale-like bracts (Figure 1F) while *Arundinaria fargesii* shows prominent sheath scars and initially compressed inflorescences with non-expanding leaf sheaths (Figure 1G). The *Ampelocalamus* clade, containing *Am. saxatilis* (= *Drepanostachyum saxatile*) and *Am. melicoideus* (= *D. melicoideum*), represented a distinct clade (MLB/MPB/PP = 99/95/1.00; Figure 1A). *Am. melicoideus* in *Ampelocalamus* clade in the present study have panicles fascicled on leafless flowering branches and 2–3 bracts on the basis of inflorescences (Figure 1E). The remaining *Fargesia* species formed two paraphyletic clades, including *F. canaliculata, F. edulis, F. fungosa, F. yunnanensis*, and *F. grossa* with *Indocalamus longiauritus* (Figure 1D) and the species from *Yushania* in a strongly supported the *Fargesia grossa* clade (MLB/MPB/PP = 98/95/1.00; Figure 1A). *F. macclureana, F. hygrophila, F. communis, F. albocerea, Fargesia* sp. and *Arundinaria faberi* formed another strongly supported the *Fargesia macclureana* clade (MLB/MPB/PP = 78/80/1.00; Figure 1A).

### Topology Testing

Since the tree topologies of the major clades based on two taxon removal experiments showed no changes when outgroups were removed (Supplementary Figures S3, S4), we concluded that the tree topology was not affected by long branch attraction.

**FIGURE 2:**
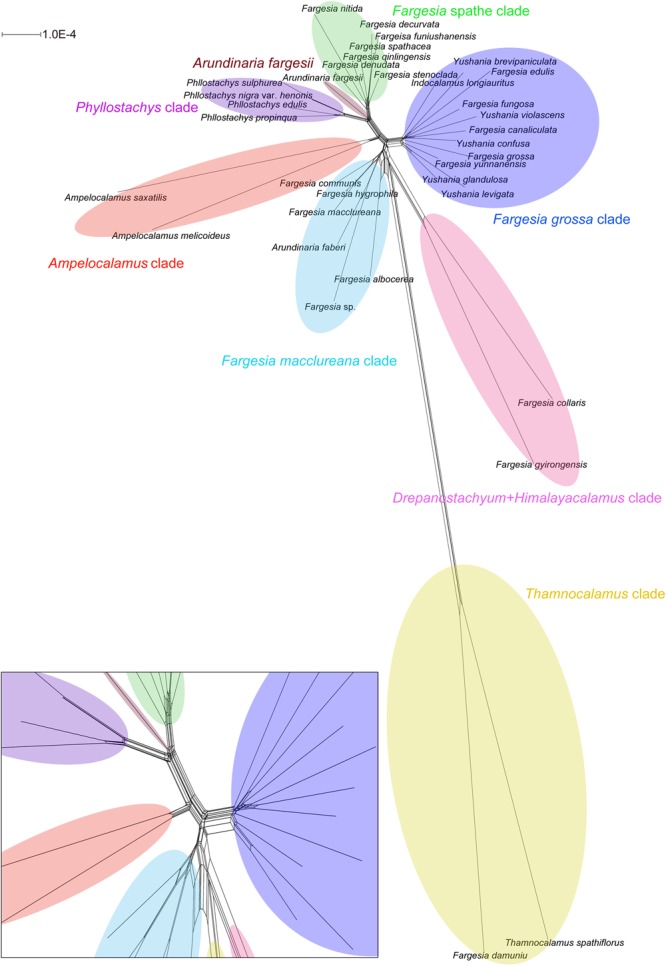
Neighbor-net analyses of *Fargesia* based on 35 plastome sequences. Clades revealed are labeled with different colors. Lower left part is a zoom-in of the network at the root.

The incompletely resolved relationships in the network suggested substantial conflicting signals (Figure 2). All species were clearly sorted into respective clades, and the clades were almost distinct. However, the relationships among the *Fargesia macclureana* clade, the *Drepanostachyum* + *Himalayacalamus* clade, and the *Thamnocalamus* clade were less clear. We used the AU test to evaluate alternative tree topologies for the relationships among the major clades of *Fargesia* and its allies (Supplementary Figure S5). The results based on the complete plastomes sequences and non-coding sequences rejected the monophyly of the *Fargesia* spathe clade (Supplementary Figure S5A), the *Fargesia grossa* clade and the *Fargesia macclureana* clade and the monophyly of the *Fargesia grossa* clade and the *Fargesia macclureana* clade (Supplementary Figures S5A,C). However, the results based on coding sequences failed to reject two topologies (Supplementary Figure S5B) involving the *Fargesia grossa* clade, the *Fargesia macclureana* clade, and the *Drepanostachyum* + *Himalayacalamus* clade.

### Divergence Time Estimates

**FIGURE 3:**
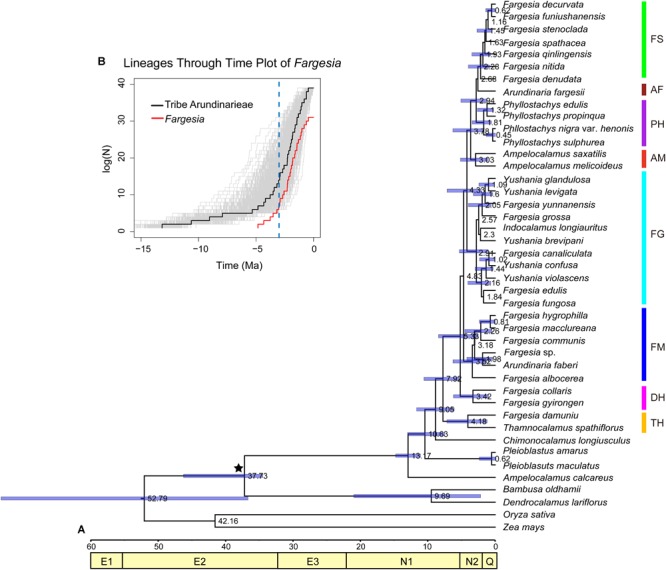
Divergence time estimates of *Fargesia* and its allies. **(A)** Chronogram of *Fargesia* and its allies based on the plastomes. E1, Paleocene; E2, Eocene; E3, Oligocene; N1, Miocene; N2, Pliocene; Q, Quaternary. Calibration points are indicated with black asterisks. **(B)** Lineage through time (LTT) plots for six major clades within *Fargesia* and tribe Arundinarieae using R-APE package. The vertical dashed lines indicate FS, *Fargesia* spathe clade; PH, *Phyllostachys* clade; AF, *Arundinaria fargesii*; AM, *Ampelocalamus* clade; FG, *Fargesia grossa* clade; FM, *Fargesia macclureana* clade; DH, *Drepanostachyum* + *Himalayacalamus* clade; TH, *Thamnocalamus* clade.

The Bayesian topology (Figure 3A) was similar to the congruent topologies from the MP, ML, and BI analyses (Figure 1A), and differed only in the placement of a few statistically unsupported nodes (e.g., *Arundinaria fargesii* and the *Phyllostachys* clade). The crown node of the Arundinarieae was dated to 13.17 Mya (95%HPD: 11.30–15.03; late Miocene); the crown node of eight clades (i.e., FS, AF, PH, AM, FG, FM, DH, TH, see Figure 1A) was dated to 7.92 Mya (95%HPD: 5.36–10.77); the crown node of six clades (i.e., FS, AF, PH, AM, FG, FM) was dated to 4.83 Mya (95%HPD: 2.19–7.30); and the *Fargesia* spathe clade crown was dated to 2.28 Mya (95%HPD: 0.93–4.13). In the diversification analyses, the slope of the six clades (i.e., FS, AF, PH, AM, FG, FM) of *Fargesia* LTT plots became steeper during ca. 3–5 Mya (Figure 3B).

### Morphological Characters Evolution

**FIGURE 4:**
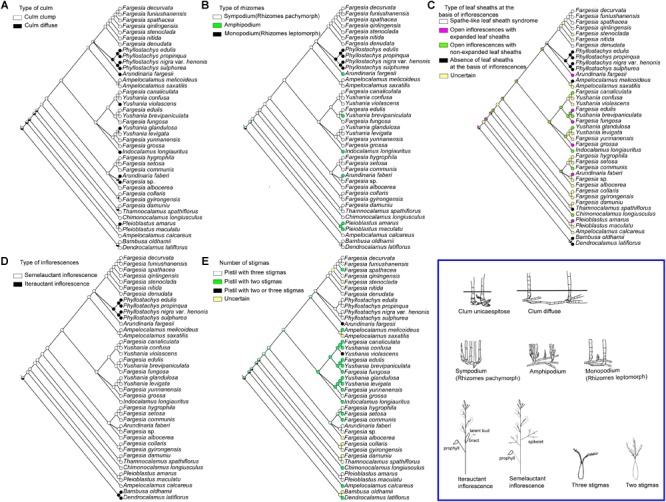
Optimization of morphological characters inferred on a 41-complete plastome sequences BI tree in Mesquite **(A)** type of culm, **(B)** type of rhizomes, **(C)** morphology of leaf sheath at the basis of inflorescences, **(D)** type of inflorescences and **(E)** number of stigmas. The illustrations of each morphological characters are shown in the blue box at the lower right.

Most extant and ancestral taxa have cespitose culms and sympodium. Diffused culms and amphipodium (e.g., *Ar. faberi* and *l. longiauritus*) or monopodium (e.g., *Phyllostachys* species) have evolved independently in several species of different lineages in parallel (Figure 4A,B). Spathe-like leaf sheath syndrome is only restricted to the *Fargesia* spathe clade (Figure 4C), whereas species in the *Fargesia grossa* clade and the *Fargesia macclureana* clade have open inflorescences with expanded leaf sheaths in varying degree (Figure 1C,D,H). The semelauctant inflorescences seem to have evolved from iterauctant inflorescences, followed by a reversal in *Phyllostachys* species (Figure 4D). The ancestral state of the number of stigmas in Arundinarieae was ambiguous due to some floral characters are unknown in outgroups (Figure 4E). The selecting of different outgroups may affect the evolutionary trend of the number of stigmas. The situation is more complex within *Fargesia* (s.l.) due to the presence of both pistils with two stigmas and three stigmas taxa in the *Fargesia* spathe clade, the *Fargesia grossa* clade and the *Fargesia macclureana* clade. Thus, the pistils with two or three stigmas might arise independently in *Fargesia* (s.l.).

## Discussion

### Polyphyly of *Fargesia*

Our phylogenetic analyses based on 43 complete plastome sequences, which represented a greater diversity known in *Fargesia* and its closely related species, confirmed that *Fargesia* (Yi, 1988a) is not monophyletic, which was consistent with previous analyses (Guo et al., 2002; Guo and Li, 2004; Triplett and Clark, 2010; Zeng et al., 2010; Wang et al., 2017; Zhang et al., 2019). In the present study, *Fargesia damuniu* and *T. spathiflorus* formed a monophyletic clade, which is in line with the previous recognition of a strongly supported the *Thamnocalamus* clade consisting of *F. damuniu* (Zhang et al., 2019) or *F. crassinoda* (Guo and Li, 2004; Zhang et al., 2019) and other *Thamnocalamus* species. The phylogenetic tree based on plastomes also supported that *F. gyirongensis* and *F. collaris* would be included in *Drepanostachyum* + *Himalayacalamus* clade. Phylogenetic relationships among the *Drepanostachyum* + *Himalayacalamus* clade, the *Fargesia grossa* clade, and the *Fargesia macclureana* clade varied depending on data partitioning. Differing rates, insufficient phylogenetic signals, and patterns of nucleotide substitution may be the cause of conflicts between partitioned data and unpartition data, as summarized by Ma et al. (2014). The isolated position of the *Drepanostachyum* + *Himalayacalamus* clade in the present study coincides well with the results from other studies that used nuclear genes (Hodkinson et al., 2010; Yang et al., 2013; Wang et al., 2017; Zhang et al., 2019) or plastid genes (Triplett and Clark, 2010; Zeng et al., 2010; Zhang et al., 2019). Furthermore, the morphology evidence (Keng, 1983; Ohrnberger, 1996; Li et al., 2006; Liu et al., 2017) supported *F. damuniu, F. gyirongensis*, and *F. collaris* should not be considered as *Fargesia* species.

Meanwhile, the phylogenetic relationships recovered in the present study corroborated *Yushania* species nested with *Fargesia* (Guo et al., 2001, 2002; Guo and Li, 2004; Yang et al., 2013; Wang et al., 2017; Zhang et al., 2019). *Yushania* was considered to be the most closely allied genus to *Fargesia* with similar vegetative characters, inflorescence types and habitats (Keng, 1957, 1984, 1987; Wang and Ye, 1980, 1981; Yi, 1982, 1985a, 1988a, 1996; Keng and Wen, 1989; Stapleton, 1994a, 1998; Wang, 1997; Guo et al., 2001, 2002; Guo and Li, 2004; Li et al., 2006). Although that opinion was supported by molecular systematic studies, the results were weakly supported and fluctuated depending on the gene and taxon sampling (Guo et al., 2001, 2002; Guo and Li, 2004; Triplett and Clark, 2010; Zhang et al., 2012; Ma et al., 2014). The recent studies based on a wider taxon sampling revealed that the sampled species of *Yushania* were nested within *Fargesia* (Wang et al., 2017; Zhang et al., 2019). Our backbone topology based on plastome data further revealed that all sampled *Yushania* species were nested within the *Fargesia grossa* clade. This result was congruent with the convergence of morphological traits. When *Yushania* was published, Keng (1957) stated that *Yushania* was different with *Fargesia* in aspects of rhizomes, simple inflorescences with only three spikelets and the acute lemma. Among those three traits, only the morphology of the rhizome was considered as the actual difference between *Fargesia* and *Yushania* (Yi, 1996; Li et al., 2006). Actually, the length and thickness of culm-necks in *Fargesia* and *Yushania* were considered as one of the highly variable traits (Wang et al., 2017) as the intermediate states were common (Yi, 1996; Guo and Li, 2004; Li et al., 2006). The pulvini at the point of the inflorescence branches were considered as an identifying characteristic of *Yushania* (Stapleton, 1994a), except for *Y. lineolate* which do not have pulvini, while *Fargesia* species have a synflorescence without pulvini except for *F. yunnanensis* with pulvini at the base of the inflorescence branches (Yi, 1996). A very small bract at the base of each of inflorescence branches in *Yushania*, which was considered as bract (Wang and Ye, 1981; Keng, 1982) or leaf sheath (Wang and Ye, 1980; Yi, 1982, 1985a,b, 1988a, 1996; Li et al., 2006; Yi et al., 2008; Yi and Jiang, 2010), is another identifying trait between these two genera (Stapleton, 1994a). However, a small bract exists at the base of each of inflorescence branches in the lower part of inflorescences in *F*. *lincangensis* and *F*. *yunnanensis* indicating the transition of this trait (Yi, 1996; Yi et al., 2008). Therefore, *Fargesia* and *Yushania* could not be distinguished by above-mentioned morphological traits.

Yi (1988a) assumed that on the premise of the similar floral morphology and the same position of a series of leaf sheaths subtending at the base of inflorescences, the small or delicate leaf sheaths in *Yushania* might evolve from the small or large leaf sheaths in *Fargesia*. *Fargesia* species have a series of leaf sheaths which expand to varying degrees, or even to spathes subtending at the basis of inflorescences (Wang and Ye, 1980, 1981; Keng, 1982; Yi, 1982, 1985a,b, 1988a, 1996; Wang, 1997; Li et al., 2006; Yi et al., 2008; Yi and Jiang, 2010) and spikelets are exserted partially and uni-laterally from leaf sheaths (Keng, 1982; Yi et al., 2008), while those of *Yushania* have small or delicate leaf sheaths which are not spathe-like (Wang and Ye, 1980, 1981; Keng, 1982; Yi, 1982, 1985a,b, 1988a, 1996; Wang, 1997; Li et al., 2006; Yi et al., 2008; Yi and Jiang, 2010) and all spikelets are exserted from leaf sheaths (Keng, 1982). However, the transitional states of this trait exist in *F. yunnanensis* and *Y. brevipaniculata*. All spikelets of *F. yunnanensis* are exserted from the delicate leaf sheaths (Yi, 1988a, 1996), while the spikelets of *Y. brevipaniculata* are exserted slightly from the non-expanded leaf sheaths (Yi, 1996). Based on basic morphological and positional similarities and the present phylogenetic analyses, we considered that a series of leaf sheaths, regardless of size, at the base of inflorescences might be a homological and synapomorphic trait in *Fargesia* and *Yushania* (Figure 1B,C, 4C). Combined with the results from our data and previous phylogenetic studies (Wang et al., 2017; Zhang et al., 2019), we suggested that *Yushania* might be included in *Fargesia* (s.l.).

### Phylogenetic Relationship Among *Fargesia* (s.l.) and Its Allies

Previous molecular systematic studies using plastid gene sequences revealed that *Fargesia* was included in the *Phyllostachys* clade of the Arundinarieae along with *Yushania, Phyllostachys, Pleioblastus, Indocalamus, Arundinaria* (incl. *Bashania*), *Drepanostachyum* and *Himalayacalamus* (Triplett and Clark, 2010; Zeng et al., 2010; Zhang et al., 2012, 2016; Ma et al., 2014; Attigala et al., 2016). Moreover, studies using nuclear markers and RAD-seq data found that *Fargesia* (incl. *Yushania*) were not monophyletic and formed a polytomy with some species from *Chimonocalamus* and *Arundinaria* subg. *Sarocalamus* (Wang et al., 2017), which had some conflicts in the plastome phylogenomics (Ma et al., 2014).

In the present study, *Arundinaria fargesii, Phyllostachys* clade and *Ampelocalamus* clade are successive sisters to the *Fargesia* spathe clade, which was incongruent with nuclear regions (Zhang et al., 2012; Wang et al., 2017). *Arundinaria fargesii* was sister to the *Fargesia* spathe clade based on our plastomes analyses, in line with previous plastid markers analyses (Zhang et al., 2012) or plastome phylogenomics (Ma et al., 2014). However, *Ar. fargesii* may be a hybrid between *F. decurvata* and *Bashania aristata* based on *GBSSI* analyses or originated from hybridization between Chinese *Pleioblastus* and *Indocalamus* based on morphological features (Zhang et al., 2012). Thus, hybridization or plastid introgression may be a reasonable explanation for different phylogenetic positions of *Ar. fargesii*.

*Phyllostachys* species were clustered within the *Fargesia* (s.l.) in our plastomes analyses, while they grouped together with other species with leptomorph rhizomes such as *Brachystachyum* and *Shibataea* based on *GBSSI* gene tree (Zhang et al., 2012) or *Pleioblastus, Acidosasa, Indosasa*, and *Shibataea* based on RAD-seq data (Wang et al., 2017), which confirmed an isolated position from species of *Fargesia* (s.l.). *Phyllostachys* species have leptomorph rhizomes and iterauctant inflorescences which have few morphological similarities compared to species of *Fargesia* (s.l.). Therefore, plastid introgression or lineage sorting may be alternative causes for the incongruence.

The previous molecular studies revealed that *Am. melicoideus* and other *Ampelocalamus* species (excluding *Am. calcareus* and *Am. actinotrichus*) grouped together with *Drepanostachyum* and *Himalayacalamus* based on nuclear gene markers (Guo et al., 2002; Guo and Li, 2004; Yang et al., 2013). The plastomes of *Ampelocalamus, Fargesia, Yushania*, and *Arundinaria* (incl. *Bashania*) might share the same origin. Plastid capture or incomplete lineage sorting could be an explanation for the incongruent phylogenetic position of *Ampelocalamus* species between plastid gene markers and nuclear gene markers (Yang et al., 2013).

The inflorescence traits also provided a clue for the differences among *Fargesia* (s.l.), *Phyllostachys* species, *Arundinaria fargesii* and *Ampelocalamus* species such as semelauctant synflorescences or iterauctant synflorescence, leafy or leafless flowering branches, and the position of inflorescence-affiliated leaf sheaths, prophylls, and bracts (Figure 1A,B,E,G, 4C). For example, the inflorescences of *Am. melicoideus* interrupted clustered on leafless flowering branches subtended by two or three bracts and one prophyll (Yi, 1996), which differ in leafy flowering branches subtended by a series of expanded leaf sheaths in varying degree of *Fargesia* (s.l.) species. However, morphological differences among these taxa might implicate intermediate forms. For example, inflorescence branches of *Ar. fargesii* subtended by very small remnants of sheaths or rings of hairs, pulvinate, which is similar to species in *Fargesia* (s.l.). Moreover, these intermediate forms may be the result of hybridization given the nested positions of complete plastomes phylogeny in the present study, which is similar to *Ar. fargesii* discussed above or what was found in the *Thamnocalamus* group (incl. *Fargesia* and *Yushania*)and *Arundinaria* groups (incl. *Bashania*) (Triplett and Clark, 2010).

Differing from the situation of *Ar. fargesii, Phyllostachys* and *Ampelocalamus, Arundinaria faberi* and *Indocalamus longiauritus* was nested within the *Fargesia grossa* clade and the *Fargesia macclureana* clade, respectively, in the present study. *Arundinaria faberi* (=*Bashania fangiana*) was considered to belong to *Arundinaria* subgenus *Sarocalamus* (Li et al., 2006) or alpine *Bashania* (Zhang et al., 2012), while *Ar. fargesii* was put into *Arundinaria* subgenus *Bashania* (Li et al., 2006). Even though they were put into *Bashania* in Floral Reipublicae Popularis Sinicae (Yi, 1996) due to similar morphology, molecular evidence suggested that no affiliation between these species was revealed (Li et al., 2006; Zeng et al., 2010; Zhang et al., 2012). *Arundinaria faberi* was clustered within the *Fargesia macclureana* clade in our plastome analyses, while *Ar. faberi* or other alpine *Bashania* species were sister to species in the *Fargesia* spathe clade rather than the species in the *Fargesia grossa* clade on the *GBSSI* gene tree (Yang et al., 2013) and RAD-seq analyses (Wang et al., 2017). In morphology, however, *Ar. faberi* have open inflorescences initially at the tips of leafy branches and later on leafless branches, and the inflorescence branches subtended by long hairs and delicate leaf sheaths (Yi, 1996; Li et al., 2006), which is similar to species in the *Fargesia macclureana* clade. *Arundinaria faberi* and species in *Fargesia* (s.l.) overlap in distribution which may provide a potential condition for hybridization. In addition, incomplete lineage sorting during speciation may be an alternative interpretation, as well as *Ampelocalamus* species.

The placement of *I. longiauritus* may stay incertae sedis due to a putative hybridization event between *Pseudosasa guanxianensis* and *Bashania qingchengshanensis* based on previous multiple plastid regions (Zeng et al., 2010) and *GBSSI* gene (Zhang et al., 2012). A lack of informative characters and taxa sampling may be responsible for the disparity between our results and previous plastid phylogenies (Zeng et al., 2010; Zhang et al., 2012; Ma et al., 2014). *Indocalamus longiauritus* has amphipodium rhizomes with spaced culms and open inflorescences with tiny bracts subtending at the basis of inflorescence branches (Figure 1D; Li et al., 2006; Yi et al., 2008), which has some morphological differences compared with *Fargesia* (s.l.). Moreover, the geographic distribution of *I. longiauritus* overlaps less with that of species of *Fargesia* (s.l.) (Yi, 1996; Li et al., 2006; Yi et al., 2008). Thus, incomplete lineage sorting or homoplasy may be another cause for the present phylogenetic disparities among *I. longiauritus* species in *Fargesia grossa* clade based on plastomes and nuclear markers.

Our results also indicated the Arundinarieae began to diversify around 13.17 Mya and *Fargesia* (s.l.) along with the *Phyllostachys* clade, *Ampelocalamus* clade, *Ar. fargesii, Ar. Faberi*, and *I. longiauritus* undergo a rapid late Pleistocene radiation at 4.83 Mya (95%HPD: 2.19–7.30). This is close to the recent recognition of a relatively recent dispersal event in the Pliocene within *Phyllostachys* clade of the Arundinarieae ranging from an estimated 2.38 to 3.10 Mya (Zhang et al., 2016). Arundinarieae may have experienced rapid radiation, especially within the *Arundinaria* clade, *Phyllostachys* clade and *Shibataea* clade of Arundinarieae, starting from ca. 7–8 Mya (Zhang et al., 2016). A bottleneck to resolving the phylogenetic uncertainty among *Fargesia* (s.l.) and its allies might be the low information and different genetic background of molecular markers (nrDNA and plastid genes), like other clades in Arundinarieae (Triplett and Clark, 2010; Zeng et al., 2010; Zhang et al., 2012; Yang et al., 2013; Ma et al., 2014; Attigala et al., 2016).

Using a combination of phylogenetic estimation and morphological evolution, we revealed that *Arundinaria fargesii, Phyllostachys* species, and *Ampelocalamus* species might not be included in *Fargesia* (s.l.) due to recent radiation, plastid capture, incomplete lineage sorting, and hybridization/introgression among different clades within *Fargesia* (s.l.). We need more species *Arundinaria* (incl. *Bashania*) and *Indocalamus* with more nuclear markers and morphological analyses to amend *Arundinaria* (incl. *Bashania*) and *Indocalamus* and further disentangle the phylogenetic relationships among *Ar. faberi, I. longiauritus* and *Fargesia* (s.l.). Thus, we temporarily excluded them from *Fargesia* (s.l.) to discuss the infrageneric phylogenetic relationships within *Fargesia* (s.l.) below.

### Infrageneric Phylogenetic Relationships Within *Fargesia* (s.l.)

*Fargesia* was first described by Franchet (1893) based on *F. spathacea*, a species with spathe-like leaf sheath syndrome. However, Yi (1983a) considered that other alpine bamboo species which have the inflorescences subtending by a series of expanded leaf sheaths in varying degree should be added in *Fargesia*. Therefore, Yi (1983a,b,c, 1985a,b,c, 1986, 1988a,b, 1989, 1990, 1991a,b, 1992a,b, 2000a,b,c) and others (Lu, 1981; Wang and Ye, 1981; Wen, 1984, 1989; Keng, 1987; Yi and Shao, 1987; Yi and Long, 1989; Yi et al., 2005, 2006, 2007a,b, 2008; Yi and Zhu, 2012; Yang and Yi, 2013) published ca. 100 new species of *Fargesia*. Subsequently, Yi (1985a,b, 1988a) amended this genus and established an infrageneric classification of *Fargesia* including two sections (Yi, 1985a,b) and six series (Yi, 1988a) based on morphology of buds, branches and culm sheaths among which only two sections were accepted (Ohrnberger, 1999; Yi et al., 2008). The morphological continuity observed in the field (Zhang et al., 2014) and molecular systematic studies (Zhang et al., 2019) have been revealed these key morphological characters are not objective.

Our analyses highlight species with spathe-like leaf sheath syndrome formed the *Fargesia* spathe clade with high supported in line with previous nuclear molecular studies (Guo et al., 2001; Guo and Li, 2004; Wang et al., 2017; Zhang et al., 2019). Here, we speculate that the spathe-like leaf sheath syndrome may be a morphological synapomorphy of *Fargesia* (s. s.) species that can be used to distinguish the species of the *Fargesia* spathe clade from those of other *Fargesia* clades. On the other hand, species assigned to the non-spathe clade by an earlier study (see Zhang et al., 2019) fell into two strongly supported paraphyletic clades, the *Fargesia grossa* clade and the *Fargesia macclureana* clade. The result of the AU test also demonstrated the non-monophyly of the *Fargesia grossa* clade and the *Fargesia macclureana* clade. The use of whole plastid genome sequences has the potential to increase the resolution of phylogenetic analyses at low taxonomic levels or among recently diverged species (Njuguna et al., 2013; Williams et al., 2016; Sancho et al., 2017; Uribe-Convers et al., 2017; Fonseca and Lohmann, 2018). Taxon sampling (including ingroups and outgroups) is important for phylogenetic inference, particularly for the complex temperate woody bamboos. The non-spathe clade may be further broken, even more clades will be recovered as sampling more *Fargesia* and *Yushania* species in the future.

The *Fargesia grossa* clade in the present study included some species of *Fargesia* (incl. *Borinda*), all species of *Yushania* and *Indocalamus longiauritus*. Those species have open inflorescences subtended by expanded leaf sheaths and pachymorph rhizomes with a long (5–20 cm) and thick (0.25–7 cm) neck (Li et al., 2006; Yi et al., 2008), except for *I. longiauritus*. The *Fargesia macclureana* clade in the present study included *F. macclureana, F. albocerea, F. hygrophila, F. communis, Fargesia* sp. and *Ar. faberi*. In this clade, all species have lax inflorescences subtended by unexpanded leaf sheaths and pachymorph rhizomes with a short (3–11 cm) and thin (0.4–2.3 cm) neck (Li et al., 2006; Yi et al., 2008), expect for *Ar. faberi*. The range of the thickness and the length of the culm neck and the degree of the openness of the leaf sheaths at the basis of the inflorescences are overlapped in different species of two clades, so that these morphological traits may not be sufficient to distinguish between these two clades. These differences may be explained, at least in part, by the absence of morphological autapomorphies, high levels of homoplasy in these two clades.

Temperate woody bamboos might flower frequently at the beginning of the rapid divergence, but climate fluctuation might influence their subsequent life cycles (Zhang et al., 2012). Thus, long generation times might mean that temperate bamboos have a relatively recent diversification compared to annual flowering plants or tropical bamboos (Stapleton et al., 2009). Basic knowledge on the genetics and inflorescence morphology of *Fargesia* is still lacking due in part to their unusual life cycle, with the vegetative phase ranging from a few to 120 or even 150 years (Janzen, 1976; Campbell, 1987). Our estimation of the divergence times for *Fargesia* (s.l.) and its allies implies that *Fargesia* (s.l.) might also undergo rapid radiation, which accounts for the difficulties of reconstructing the phylogeny of *Fargesia* to some degree. As plastomes only provide maternally inherited signals, further study with more nuclear DNA makers and an expanded sampling of taxa is necessary for a clearer elucidating involving the phylogenetic placement of major clades within *Fargesia* (s.l.) and complicated evolutionary history of *Fargesia* (s.l.).

### A Hypothesis: Putative Hybrid Origin of the *Fargesia* Spathe Clade

Putative relictual lineages, transitional series, and hybrids are common in the Arundinarieae, which experienced rapid radiation (Triplett and Clark, 2010; Zhang et al., 2012, 2016). In particular, hybridization has been a key factor in bamboo diversification (Triplett et al., 2014). Incongruence between trees based on nuclear and uniparentally inherited organellar DNA has been reported to be caused by hybridization or gene introgression (Hodkinson et al., 2010; Yang et al., 2013). *Arundinaria fargesii* and *Ar. faberi* may have different phylogenetic relationships with species in the *Fargesia* spathe clade due to hybridization or gene introgression accounting for discrepancies between the findings of our plastome-based study and those of Ma et al. (2014) and nuclear phylogenies (Zhang et al., 2012; Wang et al., 2017). Additionally, species in *Fargesia* (incl. *Yushania*) are so widely distributed that substantial geographic overlap likely exists between the hybrids and putative parental species from *Arundinaria* (Li et al., 2006), thus providing the potential for hybridization or gene introgression.

Morphological evidence along with molecular phylogenies suggested that transitional state or hybrids might be common in the *Yushania*, and *Arundinaria* sp. nested within *Fargesia* (Zhang et al., 2012; Yang et al., 2013; Ma et al., 2014; Wang et al., 2017). Wang et al. (2017) revealed that *Fargesia* and *Yushania* may have an intermediate morphology regarding the length of culm necks and the open degree of spathe subtending; the resulting taxonomic uncertainty might be due to hybridization. The derived spathe-like leaf sheath syndrome might be interpreted as a significant indicator. Evolution of the morphology of the leaf sheaths at the basis of inflorescences or the bracts at the basis of branches has remained puzzling as comparable morphologies in related taxa were unknown until the present discovery of a relationship among *Fargesia* (s.l.) and *Arundinaria*. *Arundinaria* species have initially compressed inflorescences subtended by reduced leaf sheaths (Li et al., 2006; Kellogg, 2015). Species in the *Fargesia grossa* clade and the *Fargesia macclureana* clade have open inflorescences with a series of expanded leaf sheaths in varying degree but never spathe-like at the basis of inflorescence branches. In other words, the species in the *Fargesia* spathe clade retain partial morphological similarity to *Arundinaria* and species in *Fargesia* (s.l.) without spathe-like leaf sheath syndrome.

The spathe-like leaf sheath syndrome might be an adaptive trait facing decreasing temperature, as flower buds could be protected by the expanded spathes. Our divergence time estimation showed that the *Fargesia* spathe clade originated and diversified in ca. 2.28 Mya, in the Quaternary. Climatic oscillations in the Quaternary likely had tremendous effects on current plant distribution (Hewitt, 2000, 2004). When temperature decreased in glacial times, species capable of enduring cold likely expanded northward, as in the case of the *Fargesia* spathe clade. The fact that this clade is currently distributed in the north compared to *Fargesia* species without spathe-like leaf sheath syndrome also supports the hypothesis. Our data thus present an interesting scenario concerning the evolution of the *Fargesia* spathe clade: hybridization or gene introgression between *Arundinaria* species and species that bore a preliminary spathe-like leaf sheath syndrome species in the *Fargesia grossa* clade or the *Fargesia macclureana* clade (i.e., the species have a series of leaf sheaths subtending at the basis of inflorescences or bracts at the basis of branches, which could be further expanded or elongated) produced an advanced spathe-like leaf sheath syndrome, which conferred the ability for the species in the *Fargesia* spathe clade to expand northward in the Quaternary. However, this hypothesis needs further validation from multiple nuclear marker-based phylogenies and ecological niche modeling.

We assembled and annotated 26 plastomes, which allowed us to infer a well-supported phylogenetic backbone for *Fargesia* (Yi, 1988a) and its allies. Our results revealed that all sampled species of *Yushania* were nested with most of *Fargesia* and we propose a new *Fargesia* (s.l.) and further divided into the *Fargesia* spathe clade, the *Fargesia grossa* clade and the *Fargesia macclureana* clade. *Phyllostachys* clade, *Arundinaria fargesii*, and *Ampelocalamus* clade are inserted in *Fargesia* (s.l.), sister to the *Fargesia* spathe clade. The derived spathe-like leaf sheath syndrome was believed to be unique to the *Fargesia* spathe clade, which probably originated from hybridization or gene introgression between the *Fargesia grossa* clade or the *Fargesia macclureana* clade and *Arundinaria* species. Moreover, climatic oscillations in the Quaternary may also contribute to the formation of the derived spathe-like leaf sheath syndrome. Elucidating the complex evolutionary history of *Fargesia* and its allies remains difficult as multiple biological factors may play complex roles among lineages. Future work with multiple independent molecular regions will help to test different hypotheses of biological factors generated by the current study.

## Data Availability

The datasets generated for this study can be found in GenBank (National Center for Biotechnology Information) with the accession numbers MH988715–MH988740.

## Author Contributions

Y-QZ and X-CX collected the materials and conducted the field investigations. YZ conducted the experiments and analyzed the data. YR, J-QZ, and YZ wrote the manuscript. All authors have read and approved the final manuscript.

## Conflict of Interest Statement

The authors declare that the research was conducted in the absence of any commercial or financial relationships that could be construed as a potential conflict of interest.

## Footnotes

1 https://www.ncbi.nlm.nih.gov/

2 http://www.qiagenbioinformatics.com/product-downloads/

3 https://blast.ncbi.nlm.nih.gov/Blast.cgi

## Supplementary Material

FIGURE S1 Gene map of the chloroplast genome of *Fargesia denudata*. Individual genes are labeled and those belonging to different functional groups are color coded. Dashed area in the inner circle indicates the GC content of the genome.

FIGURE S2 Phylogeny of 43 taxa dataset inferred from Bayesian analysis based on different chloroplast genome partitions. Numbers above branches are ML bootstrap values/MP bootstrap values. Numbers below the branches are posterior probabilities. Dashes represent nodes unresolved or without bootstrap support in the ML or MP trees or contradicted by the BI trees with PPs < 0.50. **(A)** coding sequences, **(B)** non-coding sequences, **(C)** large single-copy sequences, **(D)** small single-copy sequences, **(E)** inverted repeat region sequences, **(F)** complete plastome sequences.

FIGURE S3 Phylogeny of 41 taxa dataset inferred from Bayesian analysis of complete plastome sequences. Numbers above branches are ML bootstrap values/MP bootstrap values. Numbers below the branches are posterior probabilities. Dashes represent nodes unresolved or without bootstrap support in the ML or MP trees or contradicted by the BI trees with PPs < 0.50. Roman numerals represent the revealed clades. FS, *Fargesia* spathe clade; PH, *Phyllostachys* clade; AF, *Arundinaria fargesii*; AM, *Ampelocalamus* clade; FG, *Fargesia grossa* clade; FM, *Fargesia macclureana* clade; DH, *Drepanostachyum* + *Himalayacalamus* clade; TH, *Thamnocalamus* clade.

FIGURE S4 Phylogeny of 39 taxa dataset inferred from Bayesian analysis of complete plastome sequences. Numbers above branches are ML bootstrap values/MP bootstrap values. Numbers below the branches are posterior probabilities. Dashes represent nodes unresolved or without bootstrap support in the ML or MP trees or contradicted by the BI trees with PPs < 0.50. Roman numerals represent the revealed clades. FS, *Fargesia* spathe clade; PH, *Phyllostachys* clade; AF, *Arundinaria fargesii*; AM, *Ampelocalamus* clade; FG, *Fargesia grossa* clade; FM, *Fargesia macclureana* clade; DH, *Drepanostachyum* + *Himalayacalamus* clade; TH, *Thamnocalamus* clade.

FIGURE S5 The hypothetical topologies approximately unbiased (AU) test in CONSEL based on **(A)** complete plastome sequences, **(B)** coding sequences and **(C)** non-coding sequences. FS, *Fargesia* spathe clade; PH, *Phyllostachys* clade; AF, *Arundinaria fargesii*; AM, *Ampelocalamus* clade; FG, *Fargesia grossa* clade; FM, *Fargesia macclureana* clade; DH, *Drepanostachyum* + *Himalayacalamus* clade; TH, *Thamnocalamus* clade. Bold lines illustrate the lineages that were constrained as monophyletic. The optimal topologies according to the *p*-value of the approximately unbiased test are shaded.

TABLE S1 The primers newly designed in this study for gap closure and validation of the assembled chloroplast genomes.

TABLE S2 Data matrix of the five morphological characters used in the morphological evolution of *Fargesia* and the outgroup. Bold indicated morphological character states of inflorescences observed based on herbarium specimens.

TABLE S3 The full alignment of complete plastome sequences from the 43 species excluding the gaps (NEXUS format).

TABLE S4 The results of substitution saturation tests implemented in DAMBE.

TABLE S5 Comparison of partitioning strategies used for the data sets implemented in PartitionFinder v.1.1.1 (Lanfear et al., 2012).
