# Supplementary figures and images for: Straight From the Plastome: Molecular Phylogeny and Morphological Evolution of Fargesia (Bambusoideae: Poaceae)
Source: Front Plant Sci. 2019 Aug 6;10:981. doi: 10.3389/fpls.2019.00981 (PMC6691181; doi:10.3389/fpls.2019.00981)

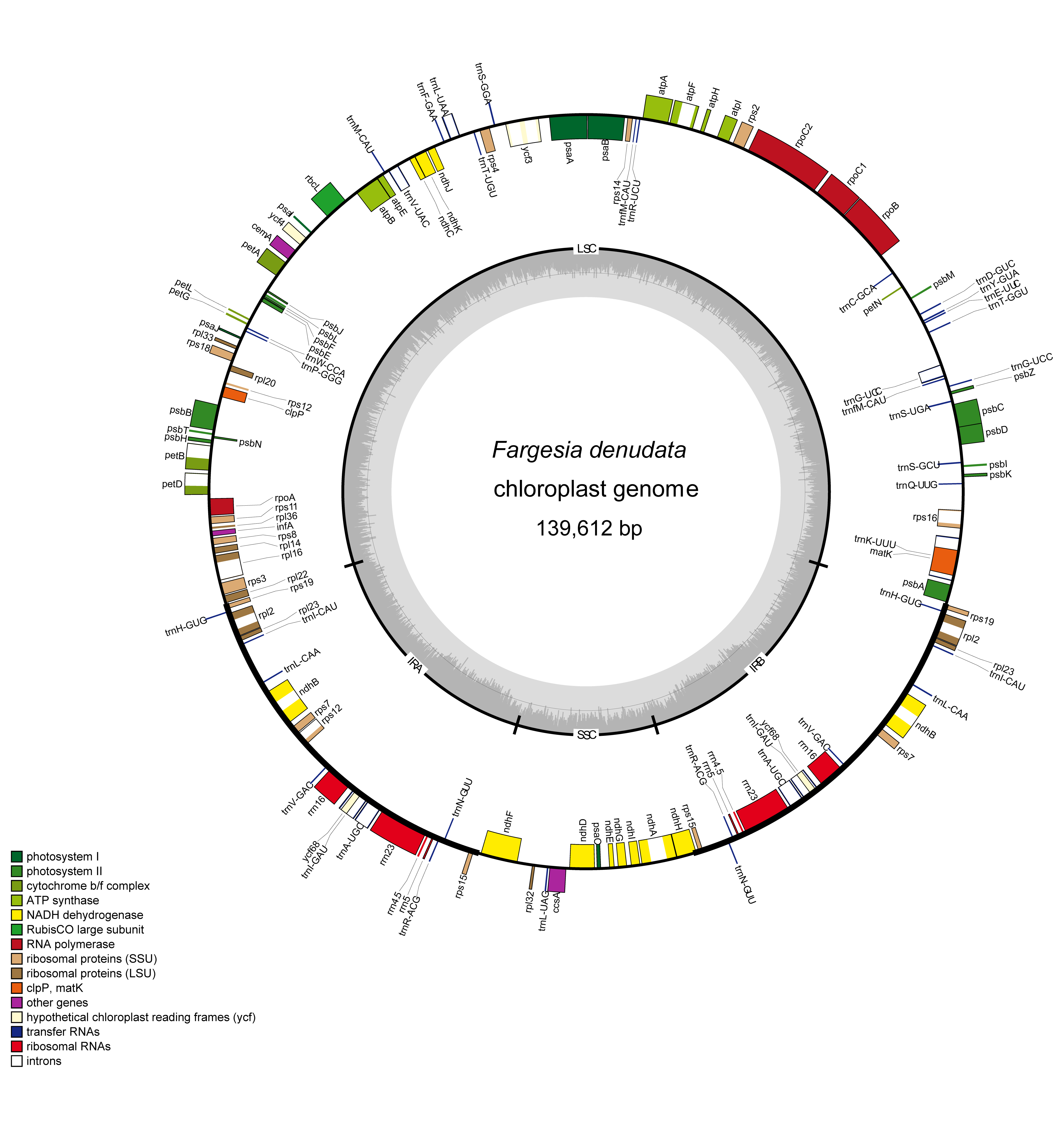

Supplement: FIGURE S1 — Gene map of the chloroplast genome of Fargesia denudata. Individual genes are labeled and those belonging to different functional groups are color coded. Dashed area in the inner circle indicates the GC content of the genome. [file Image_1.TIF]

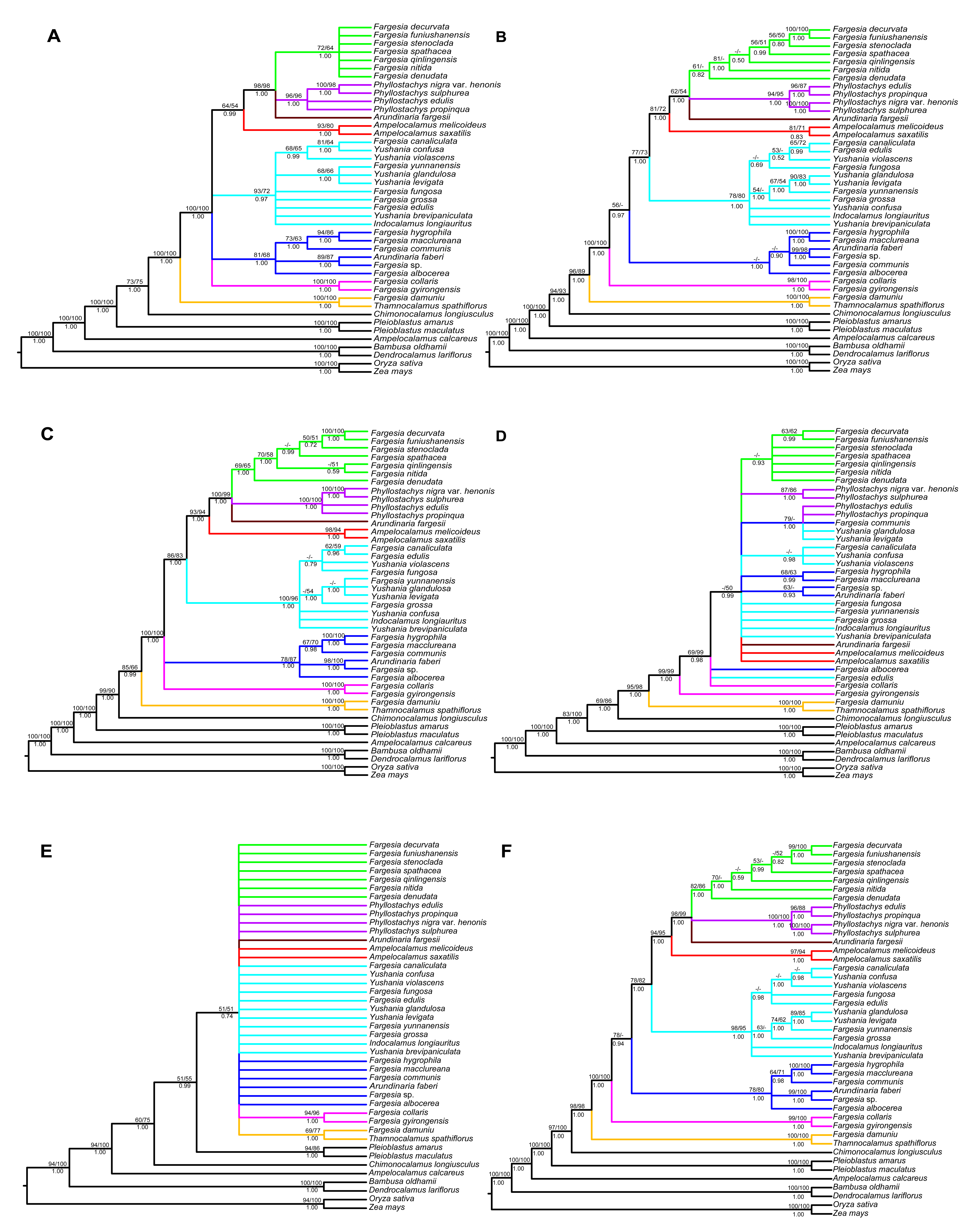

Supplement: FIGURE S2 — Phylogeny of 43 taxa dataset inferred from Bayesian analysis based on different chloroplast genome partitions. Numbers above branches are ML bootstrap values/MP bootstrap values. Numbers below the branches are posterior probabilities. Dashes represent nodes unresolved or without bootstrap support in the ML or MP trees or contradicted by the BI trees with PPs < 0.50. (A) coding sequences, (B) non-coding sequences, (C) large single-copy sequences, (D) small single-copy sequences, (E) inverted repeat region sequences, (F) complete plastome sequences. [file Image_2.TIF]

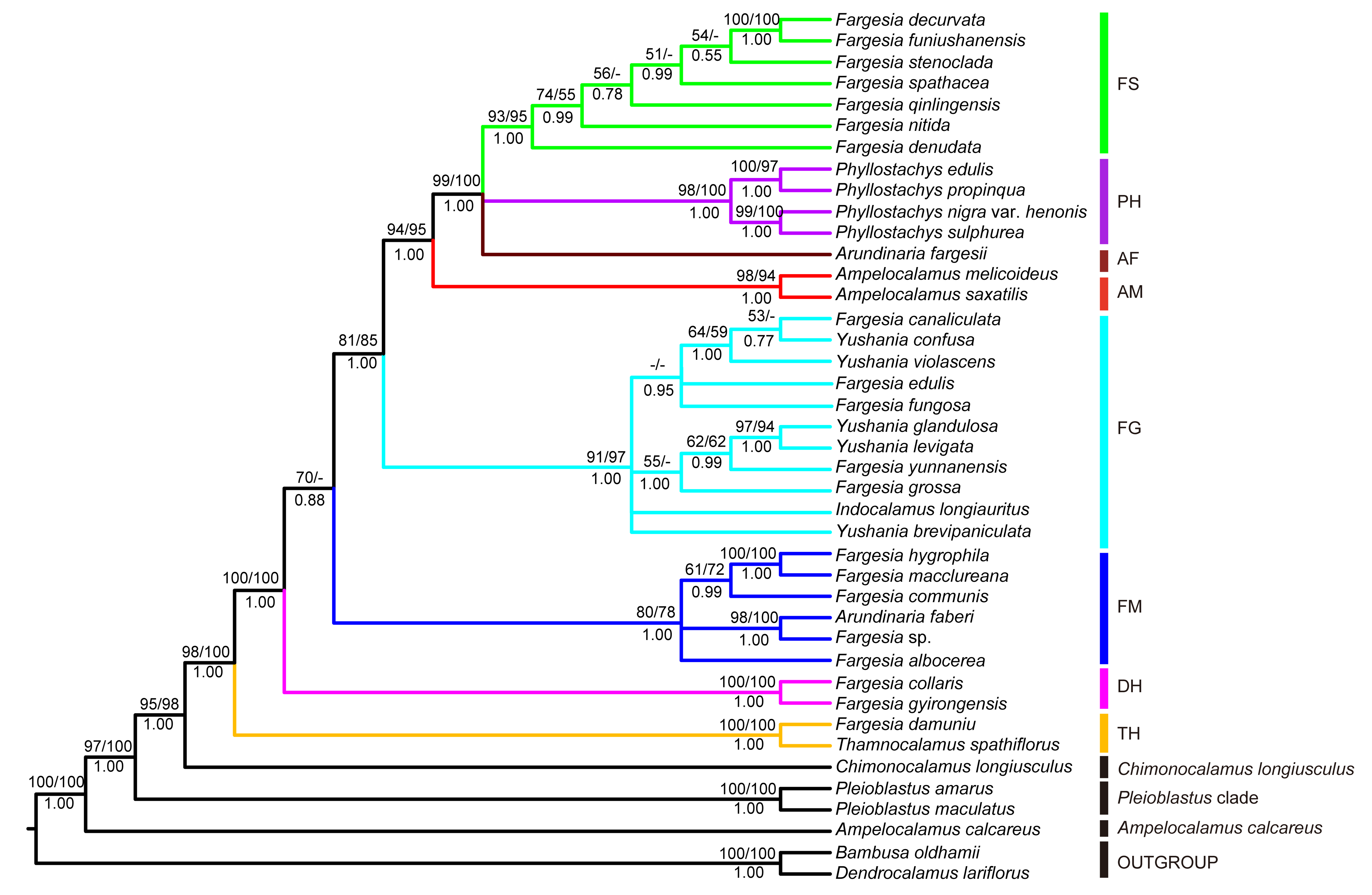

Supplement: FIGURE S3 — Phylogeny of 41 taxa dataset inferred from Bayesian analysis of complete plastome sequences. Numbers above branches are ML bootstrap values/MP bootstrap values. Numbers below the branches are posterior probabilities. Dashes represent nodes unresolved or without bootstrap support in the ML or MP trees or contradicted by the BI trees with PPs < 0.50. Roman numerals represent the revealed clades. FS, Fargesia spathe clade; PH, Phyllostachys clade; AF, Arundinaria fargesii; AM, Ampelocalamus clade; FG, Fargesia grossa clade; FM, Fargesia macclureana clade; DH, Drepanostachyum + Himalayacalamus clade; TH, Thamnocalamus clade. [file Image_3.TIF]

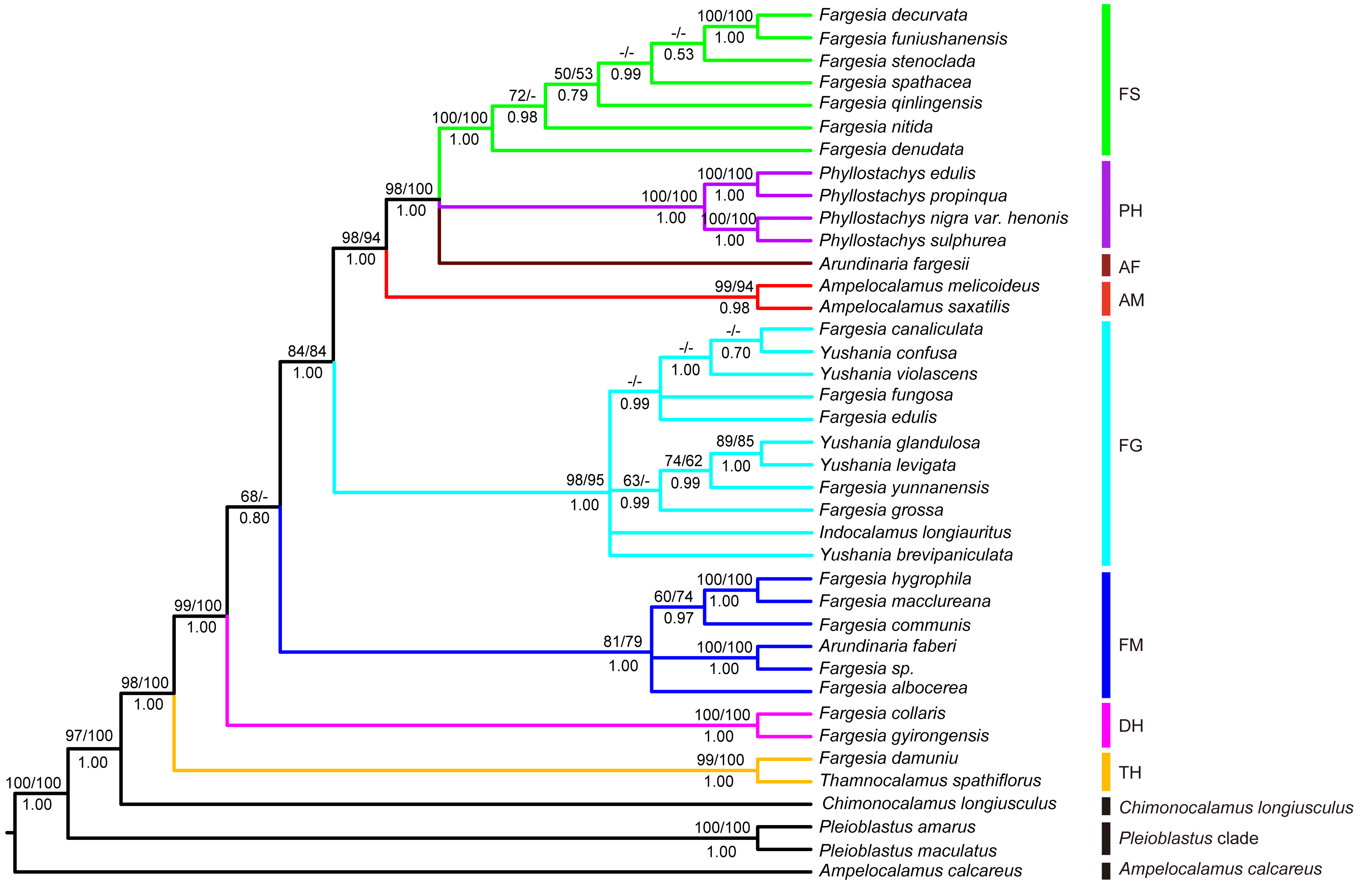

Supplement: FIGURE S4 — Phylogeny of 39 taxa dataset inferred from Bayesian analysis of complete plastome sequences. Numbers above branches are ML bootstrap values/MP bootstrap values. Numbers below the branches are posterior probabilities. Dashes represent nodes unresolved or without bootstrap support in the ML or MP trees or contradicted by the BI trees with PPs < 0.50. Roman numerals represent the revealed clades. FS, Fargesia spathe clade; PH, Phyllostachys clade; AF, Arundinaria fargesii; AM, Ampelocalamus clade; FG, Fargesia grossa clade; FM, Fargesia macclureana clade; DH, Drepanostachyum + Himalayacalamus clade; TH, Thamnocalamus clade. [file Image_4.TIF]

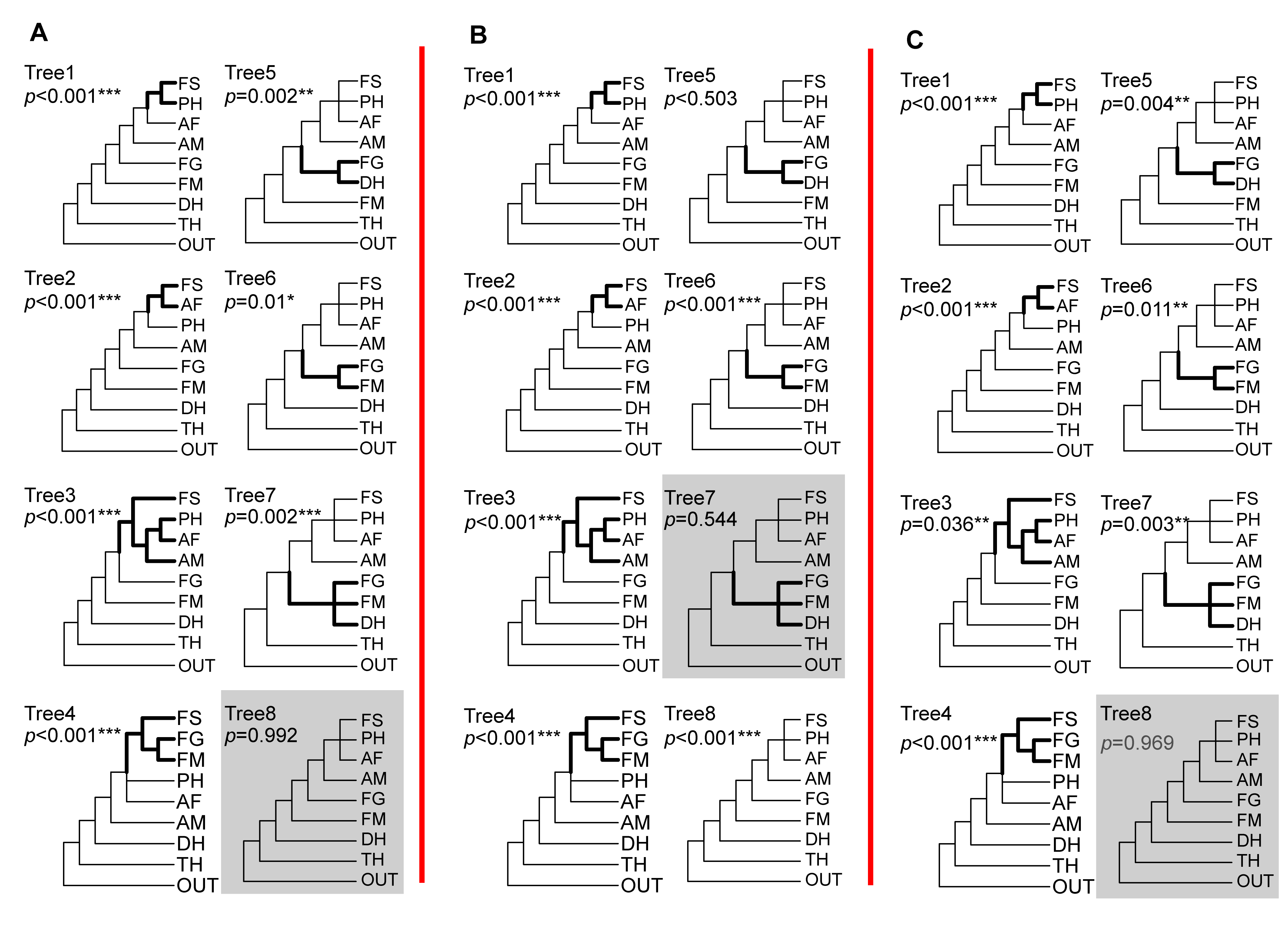

Supplement: FIGURE S5 — The hypothetical topologies approximately unbiased (AU) test in CONSEL based on (A) complete plastome sequences, (B) coding sequences and (C) non-coding sequences. FS, Fargesia spathe clade; PH, Phyllostachys clade; AF, Arundinaria fargesii; AM, Ampelocalamus clade; FG, Fargesia grossa clade; FM, Fargesia macclureana clade; DH, Drepanostachyum + Himalayacalamus clade; TH, Thamnocalamus clade. Bold lines illustrate the lineages that were constrained as monophyletic. The optimal topologies according to the p-value of the approximately unbiased test are shaded. [file Image_5.TIF]
