# Supplementary material for: Straight From the Plastome: Molecular Phylogeny and Morphological Evolution of Fargesia (Bambusoideae: Poaceae)
Source: Front Plant Sci. 2019 Aug 6;10:981. doi: 10.3389/fpls.2019.00981 (PMC6691181; doi:10.3389/fpls.2019.00981)
Supplement: TABLE S1 — The primers newly designed in this study for gap closure and validation of the assembled chloroplast genomes. [file Table_1.DOCX]

Table S1. The primers newly designed in this study for gap closure and validation of the assembled chloroplast genomes and PCR conditions.

| Primer codes | Sequence (5’-3’) | | PCR protocols |
| --- | --- | --- | --- |
| bam2 | forward | CCACAATCGATCATGTCCTTCA | 94℃,4 min; 25×(94℃, 1 min; 56℃, 1 min; 72℃, 1min); 72℃, 5min |
|  | reverse | AGCGAAAACCATCTCGATCTG |  |
| bam6 | forward | GGGTAGCCATTCGAGAAGGA | 94℃,4 min; 25×(94℃, 1 min; 57℃, 1 min; 72℃, 1min); 72℃, 5min |
|  | reverse | TCTGGCACACTTACGTCCTC |  |
| bam7 | forward | GGAGGAGTTAGGTATGTAGGGT | 94℃,4 min; 25×(94℃, 1 min; 55℃, 1 min; 72℃, 1min); 72℃, 5min |
|  | reverse | GCTCAAAACGTTCTGCAGAA |  |
| bam9 | forward | CCCCTCCATTTTGTAGTTCCA | 94℃,4 min; 25×(94℃, 1 min; 56℃, 1 min; 72℃, 1min); 72℃, 5min |
|  | reverse | TGAGATGGAGAAAAGAGGGCT |  |
| bam10 | forward | AAAACAGTCAGCCAAAATGATT | 94℃,4 min; 25×(94℃, 1 min; 52℃, 1 min; 72℃, 1min); 72℃, 5min |
|  | reverse | TGAAATTAAGTGGATGCAGTGA |  |
| bam11 | forward | AACAAATGGAAAGTGTGCGA | 94℃,4 min; 25×(94℃, 1 min; 52℃, 1 min; 72℃, 1min); 72℃, 5min |
|  | reverse | TGTTTCACTCCTGTACCTGT |  |
| bam12 | forward | CAGGGATATGTGGAGAGGTG | 94℃,4 min; 25×(94℃, 1 min; 54℃, 1 min; 72℃, 1min); 72℃, 5min |
|  | reverse | AATCAGATTGGATGGCAAAGA |  |
| bam13 | forward | TGGTTGAAAGGCCTGAAAGA | 94℃,4 min; 25×(94℃, 1 min; 55℃, 1 min; 72℃, 1min); 72℃, 5min |
|  | reverse | CCTTTACGCTTTGCCGAGAT |  |
| bam14 | forward | TTATCCAATGCCGAATCGAC | 94℃,4 min; 25×(94℃, 1 min; 55℃, 1 min; 72℃, 1min); 72℃, 5min |
|  | reverse | GAGCCTATCCTCTATCTTCTCT |  |
| bam15 | forward | GCTATTTCGCGTTTTCAAAGT | 94℃,4 min; 25×(94℃, 1 min; 53℃, 1 min; 72℃, 1min); 72℃, 5min |
|  | reverse | CGCTCCCTCGAAAAGTTTTA |  |
| bam16 | forward | ACTTGATATGCTTAACTGGAGGA | 94℃,4 min; 25×(94℃, 1 min; 54℃, 1 min; 72℃, 1min); 72℃, 5min |
|  | reverse | GAAAATGCCAAGAAATTCGCA |  |
| bam17 | forward | CATGAGGACGGGAAAAGAAA | 94℃,4 min; 25×(94℃, 1 min; 55℃, 1 min; 72℃, 1min); 72℃, 5min |
|  | reverse | CCGTCATAGAATAGGTGTCTCA |  |
| bam18 | forward | ACTTGCCTTGTTTGTTGGAT | 94℃,4 min; 25×(94℃, 1 min; 54℃, 1 min; 72℃, 1min); 72℃, 5min |
|  | reverse | TGTTGTGGAATCTGTCAGTTC |  |
| bam19 | forward | GCTTCACAACCTAGAATCCG | 94℃,4 min; 25×(94℃, 1 min; 55℃, 1 min; 72℃, 1min); 72℃, 5min |
|  | reverse | AGCCTTGTCTTCCCATTCTG |  |
| bam20-1 | forward | GTTTCCGATTCACCAAACCA | 94℃,4 min; 25×(94℃, 1 min; 55℃, 1 min; 72℃, 1min); 72℃, 5min |
|  | reverse | GCTACCGCTGGAAAATGATC |  |
| bam20-2 | forward | TCCGCATTAGAGAAAATCAAGT | 94℃,4 min; 25×(94℃, 1 min; 56℃, 1 min; 72℃, 1min); 72℃, 5min |
|  | reverse | CGTTTGGTAGTATCATTGGGG |  |
| bam22 | forward | CCGTTTGTGAGAAAGCGTGA | 94℃,4 min; 25×(94℃, 1 min; 58℃, 1 min; 72℃, 1min); 72℃, 5min |
|  | reverse | TCACAAGCCGAAACATGGTT |  |
| bam23 | forward | AACCATGTTTCGGCTTGTGA | 94℃,4 min; 25×(94℃, 1 min; 55℃, 1 min; 72℃, 1min); 72℃, 5min |
|  | reverse | ACTATCCCAGATACGTCATGGT |  |
| bam25 | forward | AATGTGAAGCAAGTCTCCGT | 94℃,4 min; 25×(94℃, 1 min; 57℃, 1 min; 72℃, 1min); 72℃, 5min |
|  | reverse | GAGCCGAAAGATGGATGCAT |  |
