# Supplementary material for: Straight From the Plastome: Molecular Phylogeny and Morphological Evolution of Fargesia (Bambusoideae: Poaceae)
Source: Front Plant Sci. 2019 Aug 6;10:981. doi: 10.3389/fpls.2019.00981 (PMC6691181; doi:10.3389/fpls.2019.00981)
Supplement: TABLE S2 — Data matrix of the five morphological characters used in the morphological evolution of Fargesia and the outgroup. Bold indicated morphological character states of inflorescences observed based on herbarium specimens. [file Table_2.DOCX]

Table S2. Data matrix of the five morphological characters used in the morphological evolution of *Fargesia* and the outgroup. Bold indicated morphological character states of inflorescences observed based on herbarium specimens.

| Species | Type of culm | Tpye of rhizomes | Types of leaf sheaths of the basis of inflorescences | Type of inflorescences | Number of stigmas |
| --- | --- | --- | --- | --- | --- |
| *Ampelocalamus calcareus* | 0 | 0 | ? | 0 | 1 |
| *Ampelocalamus melicoideus* | 0 | 0 | 3 | 0 | 1 |
| *Ampelocalamus saxatilis* | 0 | 0 | ? | 0 | ? |
| *Arundinaria faberi* | 1 | 1 | 1 | 0 | 0 |
| *Arundinaria fargesii* | 1 | 1 | 1 | 0 | 2 |
| *Bambusa oldhamii* | 0 | 0 | 3 | 1 | ? |
| *Chimonocalamus longiusculus* | 0 | 0 | 2 | 0 | 1 |
| *Dendrocalamus lariflorus* | 0 | 0 | 3 | 1 | 1 |
| *Fargesia albocerea* | 0 | 0 | ? | 0 | ? |
| *Fargesia canaliculata* | 0 | 0 | **2** | 0 | **1** |
| *Fargesia collaris* | 0 | 0 | ? | 0 | ? |
| *Fargesia communis* | 0 | 0 | 2 | 0 | 1 |
| *Fargesia damuniu* | 0 | 0 | ? | 0 | ? |
| *Fargesia decurvata* | 0 | 0 | 0 | 0 | 0 |
| *Fargesia denudata* | 0 | 0 | 0 | 0 | 0 |
| *Fargesia edulis* | 0 | 0 | 1 | 0 | 1 |
| *Fargesia fungosa* | 0 | 0 | 1 | 0 | 1 |
| *Fargesia funiushanensis* | 0 | 0 | **0** | 0 | **0** |
| *Fargesia grossa* | 0 | 0 | 1 | 0 | 0 |
| *Fargesia gyirongensis* | 0 | 0 | ? | 0 | 0 |
| *Fargesia hygrophila* | 0 | 0 | ? | 0 | 0 |
| *Fargesia macclureana* | 0 | 0 | ? | 0 | 1 |
| *Fargesia nitida* | 0 | 0 | 0 | 0 | 0 |
| *Fargesia qinlingensis* | 0 | 0 | **0** | 0 | **0** |
| *Fargesia* sp. | 1 | 0 | ? | 0 | 0 |
| *Fargesia spathacea* | 0 | 0 | 0 | 0 | 1 |
| *Fargesia stenoclada* | 0 | 0 | ? | 0 | ? |
| *Fargesia yunnanensis* | 0 | 0 | ? | 0 | 1 |
| *Indocalamus longiauritus* | 1 | 1 | 2 | 0 | 1 |
| *Phyllostachys edulis* | 1 | 2 | 3 | 1 | 0 |
| *Phyllostachys nigra* var. *henonis* | 1 | 2 | 3 | 1 | 0 |
| *Phyllostachys propinqua* | 1 | 2 | 3 | 1 | 0 |
| *Phyllostachys sulphurea* | 1 | 2 | 3 | 1 | 0 |
| *Pleioblastus amarus* | 1 | 1 | 1 | 0 | 0 |
| *Pleioblastus maculatus* | 0 | 1 | ? | 0 | 0 |
| *Thamnocalamus spathiflorus* | 0 | 0 | 3 | 0 | 0 |
| *Yushania brevipaniculata* | 0 | 1 | 2 | 0 | 1 |
| *Yushania confusa* | 0 | 0 | ? | 0 | 1 |
| *Yushania glandulosa* | 1 | 0 | 2 | 0 | 1 |
| *Yushania levigata* | 0 | 0 | ? | 0 | 1 |
| *Yushania violascens* | 1 | 0 | ? | 0 | 2 |
