# Supplementary material for: Straight From the Plastome: Molecular Phylogeny and Morphological Evolution of Fargesia (Bambusoideae: Poaceae)
Source: Front Plant Sci. 2019 Aug 6;10:981. doi: 10.3389/fpls.2019.00981 (PMC6691181; doi:10.3389/fpls.2019.00981)
Supplement: TABLE S3 — The full alignment of complete plastome sequences from the 43 species excluding the gaps (NEXUS format). [file Table_3.DOCX]

#NEXUS

[saved by seaview on Thu May 03 19:17:48 2018]

BEGIN DATA;

DIMENSIONS NTAX=43 NCHAR=108515;

FORMAT DATATYPE=DNA

GAP=-

;

MATRIX

[1] Arundinaria_fargesii

CCCAATATCTTGCTGGAACAAGATATTGGGTATTTCTGGCTTTCCTTCCTTCAAAAATTG

CTATATGTTAGCAGAAAAGCCTTATCCATTAAGAGATGGAACTTCAAGAACAGCTAGGTC

TAGAGGGAAGTTGTGAGCATTACGTTCGTGCATTACCTCCATACCAAGATTAGCACGGTT

GATGATATCAGCCCAAGTATTAATAACGCGACCTTGGCTATCAACTACGGATTGGTTGAA

ATTGAAACCATTTAGGTTGAAAGCCATAGTACTAATACCTAAAGCAGTGAACCAGATCCC

TACTACAGGCCAAGCAGCCAAGAAGAAGTGTAAAGAACGAGAGTTGTTGAAACTAGCATA

TTGGAAGATTAATCGGCCAAAATAACCATGAGCAGCCACAATATTATAAGTCTCTTCCTC

TTGACCAAATTTGTAACCCTCATTAGCAGATTCATTTTCAGTGGTTTCCCTGATCAAACT

AGAGGTTACCAAGGAACCATGCATAGCACTGAATAGGGAACCGCCGAATACACCAGCTAC

ACCTAACATGTGAAATGGATGCATAAGGATGTTGTGCTCTGCCTGGAATACAATCATAAA

GTTGAAAGTACCAGATATTCCTAAAGGCATACCATCAGAGAAACTTCCTTGACCAATAGG

GTAAATCAAGAAAACAGCAGTAGCAGCTGCAACAGGAGCTGAATATGCAACAGCAATCCA

AGGACGCATACCCAGACGGAAACTAAGTTCCCACTCACGACCCATATAACAAGCTACACC

AAGTAAGAAGTGTAGAACAATTAGCTCATAAGGACCACCGTTGTATAACCACTCATCAAC

AGATGCAGCTTCCCAAATTGGGTAAAAGTGCAATCCGATCGCCGCAGAAGTAGGAATAAT

GGCACCAGAGATAATATTGTTTCCATAAAGTAAAGAACCAGAAACAGGCTCACGAATACC

ATCAATATCTACTGGAGGAGCAGCGATGAAGGCGATAATAAATACGGAAGTTGCGGTCAA

TAAGGTAGGGATCATCAAAACACCGAACCATCCGATGTAAAGACGATTTTCGGTGCTAGT

TATCCAGTGCAGAAGCGACCCCACAGGCTTGTACTTTCGCGTCTCTCTAAAATTGCAGTC

ATGGTAAGATCTTGGTTTATTCAAATTGCAAGGACTCCCAAGCACACGTATTAACTAAAA

AGATAATAGAAGGCTTGTTATTTAACAGTATAACATAGACTGTATACCAATGTCAACCAA

GCCAGCCCCAACGATTGGATATCCATATAACTAAATTCACCAAACCAAAAATTTTGTAAA

TGAAGTGAGTGAAAATTCAAAACTCAGATTATTTCCATATGGGTTGCCCGGGACTCGAAC

CCGGAACTAGTCGGATGGAGTAGATAATTCTTCCTTGTTACAATAGAAAAAATCCCTCCC

CAAATCGTGCTTGCATTTTTCATTGCACACGACTTTCCCTATGTAGAAATAGTCAATTTC

TATTCCAAAGAGGAAGTCTACCAATTTTTTGAATAGTAAGTTGATTCACCTACTATGAAC

ATTTCAGAATGGAAAATGTGAAAGTTTTATCTTGATATCGATCTTTCTAGTGTATTAGTT

TTGTCTAATGATTAATTAAAAGGGTTCACCAGGTCATTGATACGGATAATATCCAAATAC

CAAATACGGTCACTGTGTGATCCACGGAAAGAAAAGTGGGTTGTTTTGGTGAACATCAAA

GAAAAAACTTGCTCTTCTTCCGTAAAAAATTCTTCTAAAAATACCGAACCCAACCGTTGC

ATAAAAGTTCGTACCGTGCTTTTATGTTTACGAGCTAAAGTTCTAGCGCATGAAAGTCGA

AGTATATACTTTAGTCGATACAAAGTCCGTTTTTTCGAGGATCCACTATGATAATGAAAA

AGATTTCTACATATCCGACCAAATCGATCAAGAATATCCCAATCTGATAAATCGGTCCAA

ATGGGTTTACTAATAGGATGCCCCGATCCAGTACAAAATTGAGCTTTTGATAAGGATCCA

ATGAGGGGAGTAGCAGGGACTATGGTATCGAATTTTTTCATTTGAGTCTCTATTAGAAAT

GAATTCTCTAGCATTTGATTCCTTACTAACAAAGGATTTATTGGTACACTTGAAAGGTAC

CCCAGAAAATCGAAGCAAGAGTTTACTAATTGGTTTAGATGGATCCTTCGCGGTTGAGTC

CAAAAAGAAAAAGAATATTGCCAGAAATTGACAAGGTAACATTTCCATTTCTTCTTCAAA

AGAAGAGTTCCTTTTGATGCAAGAATTGCCTTTCCTTGATATCGAACATAATGCATAAGA

GGATCCATAAAGATCCATACGGTTTTCCGAAAAAAACCTGGGTACATTACCCCAAAATGT

TCCATCTTCCTAGAAAAGTGGATTCGTTCCACAAAGGTTCCAGAAGATGTTAATGGTAAG

CAAGAAGATTGTTTACGAAGAAACAACAAGAAAAATTCATATTCTGATACATAAGAGTTA

TATAGGAATCGAAATAGTCTTTTATTTTCTTTTTTCAAAAGAAAAATCGATTTCATTGAA

GTAATAAGACTATTCCAATTCGAATAGTAGTTGAGAAAGAATCGCAATAAATGCAAAGAT

GGAACATCTTTGATCCGGTATTGAAGGAGTTGAACCAAGATTTCAAAATGGATAGGATAG

GGTATTTCTATATGTGATAGATAATGTAAATGCAAAAATTTGTCTTCTAAAAAGGGAAAT

ATTGAATGAATAGATCGTAAATTCTGAAACTTTGGTGTTTCTTTTTCTTTCGGACAAGAT

AATTCTCGTAGCGAGAATGGGATTTCTACAACGATCGCAAACCCCTCAGATAGAATCTGA

GAATAAAACTCAGAATAAAAAAAATTGTTGTAATCCAACAATCGATCTTGGTTAGGATGA

TTAACCGAGTTAATCCAAAAATTCTGCTGATACATTCGAATAATTAAACGTTTCACAAGT

AGTGAACTAAATTTCTTGTTATTACAACTAACAATTTCCACAGGTTCGGAACCTTTTAAT

CCATAATCATGGGCAAATGCATAAATATACTCCTGAAAGAGAAGTGGGTAAACGAAGTAT

TGTTGACGAGATTTCTGTTTTTCTGAATACCCTTCCAATTTTTCCATTTGTATTTCTACT

TGAATCAGAAAGAAGAAGCATTTCTCGGTTTCTCAAATGATGATACATAGTGCAATATGG

TCAAAACAGGGTGTTGCATAATACAAACCTTTCTATCCAATTAGTTTATGTTTGTTCTAA

TTACAAAAGAGAACAAATCTTTTATTTTTGCAGGCCAATCGCTCTTTTGACTTTGGAATC

CAGTCTCTTTATCAATATACTGCTTCTTTTACACATTCAATCCATAACATCCTTTTCAAT

CTATAATCAAGAATAATTAGGATTTCAAAAAAAAAAGAAAAAGGGTCCGTTCATAGGAAA

ACCAATCTTTCCCCGCATCAGGCACTAATCTATTTTTAACGTCTAATTAGATCGGGGAAT

CATTTCAATTAAGAAGTTAAGCTCGTTGCTTTTTATTTTACCAGAATTGGAGCCAGGCTC

TATCCATTTATTCACTAGACCCAGAAAATAGGAATTTTTTTATTCCAAAAAAAAAAAGAA

ATTGATTTTATTACGACATGCTATTTTTTCCATTCATTACCCTTGAGGATCAGTCGTGGT

CTTCTAGACTCTACCAAGAGTCTGGACGAATTTGTTGCTCATCCAAATGTGTAAAGATCA

TAGTCGCACTTAAAAGCCGAGTACTCTACCATTGAGTAGCACCCAGATAAATAGGATCTT

AGATACGATCGAACCCAAAATCAATGGAATTACACCACATTGAACTAGCAAAACATTAAA

AGAAAGATTTTATCGCCATTAAAAACACTCAAATGCAAAATGAACAGGTTCGGCTAAATT

TCACTAAGGTTAAAAGCGGCCCCAATCACGATAGCAAAATTGTCATTTTTTTAGCATTTA

TATATATAAATAAATCTTGTATGAGAGTACATGCAAGAGGGACAACCTTATCATTTGAGC

GAAGTGTAGACAAAAAACCTAATATGGAGTGAGGATAAAGAGACCTATCTATCTACAAAT

TCTATTTGTTCAATAGACCTTTGTCAATGGAAATACAATGAAATTAGATAGAAAAAGTAA

ATAAAATAAGGGCTTATGTTGGATTGGCACGACATAAATCCAGTCAAAAATAGGACTAAG

AGGCAAATTGTGTCTAAATAATTAAGGGATACTAGTGATCCTCTCCTACTTTTTTATTCA

TTTAGTTCTTCAATTAACTCAAAGTTCCTTCTTTTTCTTTAAAGAATTCTGCCTTCCTTA

AAATATCATAAACAGTTCTTGTAGGTTGAGCACCCTTTTCAAGGAAATAGAGAATAGCTG

GAACATTTAAACAAGTTTGATTCTTTATCGGATCATAAAAACCTACTTTTCGAAGATCTC

TTCCTTCTCTTCGAGATCGAACATCAATTGCAACGATTCGATAGACAGCTTATTGGGATA

GATGTAGCTAAACAATCCCCCCCTAGAAACGTATAGGAGGTTTTCTCCTCATACGGCTCG

AGAATATGACTTGCATTAATTTCCTTACAGAAAAACAAATTTCATTTATACTCATGACTC

AAGTTGGTTAATTTTGACTGACAGACTTAAAAGGAAAAATCCTTCCAAATTTTTTGAGTC

GTCTCTAAACTCTTTTCTTTGTCTCATCTCGAACGAATTGACTTTTATTCCTTATTCTGA

TCCAATTCTATTGTTGAGCAATTGAAAATCGCGTTTACTTGTTCCGGAATTCTTTATCTT

TGATTTGTGAAATCCTTGGGTTTAGACATTACTTCGGGAATTCCTATTCTTTTTTCTTTC

AAAAGAGTAGCAACATACCCTTTTTTCTTATTTCCTTCGATAAAGCATTTCCCTCTTCTA

TAGAAATCGAATATGGGCGATTGATTCTGATAAACTTTTAATTGAAAGAGTTTTTCCAAT

CTTCCAAAATTGGACTTTTTCTTATTTTAACCTTTCGATTTCTATATTAAGGATAGACTG

ACAAAGTTGGCCTAATTTATTAGTTTTCACTAACCCTAGATTCTTTCCCTTGATAAAAAA

TCAATTCTGTCCTCTCGAGCTCCATCGTGTACTATTTACTTACAAACAACCCAGCGCAAA

TTTGGTTCGGGACGAATAGAACAGACTATGTCGAGCCAAGAGCATTTTCATTACTATGGA

AAATGATGGATAACAAAATCCACAATCGATCATGTCCTTCAAGTCGCACGTTGCTTTCTA

CCACATCGTTTTAAACGAAGTTTTACCATAACATTCCTCTAATTTCATTGCAAAGTGGTA

TAGGGAATTGATCCAATATGGATGGGATCATGAATAGTCATTTTTTTGTATACTAATTCA

AACTTGCTATCTATGGAGAAATATGGATAAAAGAAATAAGTATTTATCGGGGAAGACTCC

GCAAAGATCCAATTTATTTAAACCCATATTCTATCATATGAAGGAAACATAGTTCGAAAA

AGACGAATAAACAAGTTTGCTTAAGACTTATTTTTTATTGAATTTCCATCCTCAACAGAG

GACTCGAGATGGTCAATCCTGAAATGAGAAGGATCGACTCTTCTCCAACAAATAAACTAT

CAACCTCAAGTTTAATTAAATTAATTAGCAATATATTTTTCCATAACAAAAACTATTAAC

TAAATAAACTATTCCAATGAAAAGAAAGTTTTTTGGTAGTTATAGAATTCTCGTACTCTT

CGACTCGAATACCAAAAGAGGACTCCAAATCAAAATTGAATCCATTCTATCCAACGAACA

GTTCTTACCTTATCCTTACCAGAATGGATCATCTGGATATTTAAAGAATCGCAGATCGAG

ATGGTTTTCGCTTAACCAAAGAGGGGCCCTTTTTACTAATAATATAATACAACAAAAATC

TATCTCTATCATAAAGGGATAGGTCTCATTTTTTATACAGTGTTTTACGTTTTTTCATGA

AAAAAAGATATTCAATTTGACTGGACTTGACACTTGATTATGTTTTCTGAGAAAGAAAAA

AATGCTTAGAAATGCATCTAATCTAAGAGTTCATAAGAGATAATTATTCTCTTTAATAAA

CTTTTGTCTCGTGTGGGGTACAATATGATTTCATCTTTCGTTTCATCAGAAAAATCTGGG

ACGGAAGGATTCGAACCTCCGAGTAACGGGACCAAAACCCGCTGCCTTACCACTTGGCCA

CGCCCCATTTCTGGTTTTATGCGACACTAATAAACACTATTATGTTTATTTGTTATTCGT

CAATCCCACTTCAATTACATAAAAATGAGGGATACTCTCTTGCTAGGATTCTAGACATGC

GGATAATATAGAATCCAAAAAATGCATTGATCATTACATGGAATTCTATTAAGATATTAT

ATGAAAGTCGAATTTCTTCCATTCTCATTTGAGAGTGCGAATACAAGGAGGTATTTTGCG

TTTGGGAAAGTCCGAAGAAAAAAGGATTTTGAACCCGCCTTTTCTTTTTTCCCTTAGAAA

AATAACTCAATCAAAATCCAATTATCTACTCTACAAGAACGAAATGCTTGTTATGCCTAA

TATACTTAGTTTAACCTGTATCTGTTTTAATTCTGTTCTTTATCCGACTAGTTTTTTCTT

CGCCAAATTGCCCGAAGCTTATGCCATTTTCAACCCAATCGTGGATTTTATGCCTGTCAT

ACCTATACTCTTTTTTCTATTAGCCTTTGTTTGGCAAGCTGCTGTAAGTTTTCGATGAAA

TCTTTACTACTCTGTCTGCCAAATTGAATGATGTATTCATTCCAAAAAAAAAAATGAATA

AAAGCCGAGAAGTCTTATATTATGAACCTTCGATTCTAAAATTCTAATTCTTCTACATTG

AATGTATAGCTGCAGCAATAAATTGGGATCCGCCTTTCTACCCCTGCACCTACGTTGAGC

AGGTACCTTTAGGTACCCACACAATACCTAACCTAATTTTTTGATAAGAGTGCTTATTAT

AAATCAATTCTTGCAATTTTTTTAAGAATTGATTTTTGCATTTTTAGGTGTAAAAATAAA

AAAACCCATCCTAGTGGATCTGTGTGGTAAGGAAAACGGGTAATCTATTCCTTAAAAAAA

ATCTTGGAGATTATGTAATGCTTACTCTCAAACTTTTTGTTTATACAGTAGTGATATTCT

TTGTTTCCCTCTTTATCTTTGGATTCTTATCTAATGACCCAGGACGTAATCCTGGGCGTG

AGGAGTAAAAATCCAAAATTTTTTCTTACAAATTGGATTTGTTTCGTACATTTATCTATG

AGAAAATCCGGGGGTCAGAATTCCTTCCAATTCGAAAGTCCCAAATGATCCGAGGGGGCG

GAAAGAGAGGGATTCGAACCCTCGGTACAAAAAAATTGTACAACGGATTAGCAATCCGCC

GCTTTAGTCCACTCAGCCATCTCTCCCCGTTCCAAATCGAAAGGTTTCCGTGATATGACA

GAGGCAAGAAATAACGATTGCAAAAAATCCTTCCTTTTTCTTTCAAAAGTCCATAAAAAT

TATATTGCCAATTCCATTTTAATTATATTCTTTTTTCTTAATAAAAAAGAAGAAAATTCT

TGTTTTTTCTTTCTAAAATTCGATATTGGCTGAGAAACAATCAGATAGATTTTCTCTTCA

GCGGGCATTTTCATATAGGACTTGTTATAATAAAACAAGCAGGTTATATAAAAAATATTT

ATAAACAAAACAAAAAGGGTTCTTATCAAACCCACCATAAAATTGGAAAGAAAGATAAAG

TAAGTAGACCTGACTCCTTGAATGATGCCTCTATCCACTATTCTGATATATAAATTCGAT

GTAGATGAAATTGTATAAGCGGATTTTTGTATTTCCTTAGACTTAGACCGCGCAAGGCAA

GAATTTTTCGCTATTTACGATTTCATATTCTTGTTACTAGATGTTCTATAGGAATAAGAA

GAAATCGCAACTCCTTTCCGCTACACATAAAAATTGATTTCGAAAGTCAATTTTTTTCAA

TATCTTTCTTTTCCTTAAAAGATAGGCTTTGAAATAGGAGTCATGGAATAATGCTGAATT

CAAATGTTTATTTCTATAGTATAAGAAAAACTAATCGAATCAAATTCATGGATTTACCAC

GACCTCGGTTGTGACCCCATAGATAAAAATAAAAAATTTCTATCTTCGAGACCTTTGAAA

AAGGGCATTGAACGAGAAAAAATCGTCCACAGATAATCTATCGTATGCCTTGGAAGTGAT

ATGAGGTGCTCGGAAATGGTTGAAGTAATTGAATAGGAGGATCACTATGACTATAGCCCT

TGGTAGAGTTACTAAAGAAGAAAATGATCTATTTGATATTATGGACGACTGGTTACGAAG

GGACCGTTTCGTTTTTGTAGGATGGTCCGGCCTATTGCTCTTTCCTTGTGCTTATTTCGC

TTTAGGGGGTTGGTTTACAGGGACAACTTTTGTAACTTCTTGGTATACCCATGGATTGGC

TAGTTCCTATTTGGAAGGTTGTAATTTCTTAACCGCGGCAGTTTCCACCCCTGCCAATAG

TTTAGCACACTCTTTGTTGCTACTATGGGGCCCGGAAGCGCAAGGGGATTTTACTCGTTG

GTGTCAATTAGGCGGTCTGTGGACTTTTGTCGCTCTCCATGGGGCTTTTGCACTAATAGG

TTTCATGTTACGTCAATTTGAACTTGCTCGGTCTGTTCAATTGCGGCCTTATAATGCAAT

TTCATTCTCTGCTCCAATCGCTGTTTTTGTTTCCGTATTCCTTATTTATCCACTGGGGCA

ATCTGGTTGGTTCTTTGCGCCGAGTTTTGGCGTAGCAGCGATATTTCGATTCATCCTCTT

TTTCCAAGGATTTCATAATTGGACATTGAACCCATTTCATATGATGGGAGTTGCCGGAGT

ATTAGGCGCGGCTCTGCTATGCGCTATTCATGGGGCGACCGTAGAAAACACTCTATTCGA

GGACGGTGATGGTGCAAATACTTTCCGCGCTTTTAACCCAACTCAAGCTGAAGAAACTTA

TTCAATGGTCACTGCTAATCGCTTTTGGTCCCAAATCTTTGGTGTTGCTTTTTCTAATAA

ACGTTGGTTACATTTCTTTATGCTATTTGTACCCGTCACCGGTTTATGGATGAGTGCTAT

TGGCGTAGTCGGCCTGGCTCTGAACCTACGTGCCTATGACTTCGTTTCCCAGGAAATCCG

TGCAGCGGAAGATCCTGAATTTGAGACTTTCTACACTAAAAATATTCTTTTAAACGAGGG

TATTCGTGCGTGGATGGCAGCTCAGGATCAGCCTCATGAAAATCTTATATTCCCTGAGGA

GGTTCTACCACGTGGAAACGCTCTTTAATGGAACTTTCGTTTTAGCTGGTCGTGACCAAG

AAACCACCGGCTTTGCTTGGTGGGCTGGGAATGCCAGACTTATCAATTTGTCCGGTAAAC

TACTTGGAGCTCACGTAGCCCATGCCGGATTAATCGTATTCTGGGCCGGAGCAATGAACC

TATTTGAGGTGGCCCATTTCGTACCAGAAAAGCCCATGTATGAACAAGGGTTGATTTTAC

TTCCGCACTTAGCTACTCTAGGTTGGGGAGTAGGGCCGGGGGGAGAAGTTCTAGATACTT

TTCCGTACTTTGTATCTGGAGTACTTCACCTAATTTCCTCCGCAGTCTTAGGCTTCGGCG

GCATTTATCACGCGCTTCTGGGACCCGAGACTCTTGAAGAATCTTTTCCATTCTTTGGTT

ATGTATGGAAAGATAGAAATAAAATGACTACAATTTTGGGTATTCACTTAATTTTGTTAG

GTATAGGTGCTTTTCTTCTAGTACTCAAGGCTCTTTATTTTGGCGGTGTATATGATACCT

GGGCCCCGGGGGGGGGAGATGTAAGAAAAATTACCAATTTGACCCTTAGCCCCAGTGTTA

TATTTGGTTATTTACTAAAATCCCCTTTTGGGGGAGAAGGGTGGATTGTTAGTGTGGATG

ATTTAGAAGATATAATTGGGGGACATGTATGGTTGGGTTTCATTTGTGTATTTGGCGGAA

TTTGGCATATCTTAACCAAACCCTTCGCATGGGCTCGCCGTGCATTTGTATGGTCTGGAG

AAGCTTACTTGTCTTATAGTTTAGGCGCTTTATCTGTCTTTGGTGTTATCGCTTGTTGTT

TTGTCTGGTTCAATAATACGGCTTATCCGAGTGAGTTTTATGGACCCACTGGGCCAGAAG

CTTCTCAAGCTCAAGCATTTACTTTTCTAGTTAGAGACCAGCGTCTTGGAGCTAATGTGG

GATCTGCCCAAGGACCCACAGGTTTAGGTAAATATCTAATGCGTTCCCCAACGGGAGAGG

TTATTTTTGGAGGGGAAACTATGCGTTTTTGGGACCTTCGTGCTCCATGGTTAGAACCTC

TAAGGGGGCCCAACGGTTTGGACTTGAGTAGGTTGAAAAAAGACATACAACCTTGGCAAG

AACGACGTTCGGCAGAATATATGACCCATGCTCCTTTAGGCTCTTTAAATTCCGTGGGTG

GCGTAGCTACCGAGATCAATGCAGTTAATTATGTCTCTCCTAGAAGTTGGTTAGCGACCT

CCCATTTTGTTCTAGGATTCTTCTTTTTTGTGGGCCATTTGTGGCATGCAGGAAGAGCCC

GGGCTGCTGCAGCAGGCTTTGAAAAGGGAATCGATCGTGATTTGGAACCTGTTCTTTACA

TGAACCCTCTTAACTAAGATTTTTTATTTATAGCTGTTCTAGTTTTTTTCTGTTCTGGCT

CGGTTATTCCATCTAGCCGAGCCATTCATTCCTTAAAAACAAATAAAGAAACAAACGTAT

TCAATAAGCAAAAGGAGAGAGAGGGATTCGAACCCTCGATAGTTCCTAAAACTATACCGG

TTTTCAAGACCGGAGCTATCAACCACTCAGCCATCTCTCCACAGCCTAATCCCTATTTTA

TTCCTACAAATAGAACATAGCCATATGAAATGATCTACTAACCCATCTCAGATGCAAGTC

CCCTTTCGATATATCTCTGTATAAGGTGGTAAGTAATAAGTTTTAAAGAGAAGAATCAAT

GGATTCATGATTAAACCCCTCCTACTTCTTGTATTTTATTACAATTTTGGTTAAGTGAGG

GATCAAATATGTAGTCAACTTTATTTGATGGTAGCTTGGAGGATTAGAAATATGACTATT

GCTTTCCAATTAGCTGTTTTTGCATTAATTGCGACTTCCTCAGTGTTAGTAATTAGTGTA

CCCCTTGTATTTGCTTCTCCTGATGGTTGGTCAAATAATAAAAACGTTGTATTTTCCGGT

ACATCATTATGGATTGGACTAGTCTTTCTGGTAGCTATTCTGAATTCTCTCATTTCTTAA

ATTTGTTTAGTATTTAGTAGCCCGATACAAAATATAAAAAGGCCATTTCTTCGAATTGTG

AGACGCATTAAAATGCAATTTGCGTTCCGAATTGATTGACAGACAATTAAAAAAAGAAAA

CTCTAATAGAAAATGAAACGGTCGACCCAGACATAGACGGTCGACCCAGGCGGATATACC

CTATAAAATATATCCCGTAGCGAGCGTAGTTCAATGGTAAAACATCTCCTTGCCAAGGAG

AAGATACGGGTTCGATTCCCGCCGCTCGCCAGCTTAATTTAGTAAGGTACTATGATAAAA

AATTTAGTCTACTTATATTAAATTAATAGGTGTTAGTCTAGTACCGTATCCCTTACTATC

TTACCCTCTTTTGCACCCCACTCAAAAAAAGGGGCTCCGGAGGCGGGAATCGAACTCGCC

AACAGGGCTCCCTAAATTGGGGATTCACCGAGACAAACAACTGGCAAACTCTTTTAAAGG

GGTAGACTGTGCCTTTCTTTCATTTCTTTTTTCTTTTCTTCTTCTTGCTAATGAATAAAA

AGGGTTGGATCCAGCCCTCTACTCTATACAAATAGAATAGTCCTTTTATACAGACTGCTA

AGTGCGGAGACGGGAATCGAACCCGTGACCTCAAGGTTATGAGCCTCGTGAGCTACCAAA

CTGCTCTACTCCGCTCTGGAGGGACGGAAACTGGTGGACGAAAAAGGTTGAATACAGGAC

TCTACCATGTCTAGACAAATAGAATAGTCCTTTTATACAGAATGGAGCGGGTAGCGGGAA

TCGAACCCGCATCGTTAGCTTGGAAGGCTAGGGGTTATAGTCGACGTTGGTTGATTAGTT

TTAACGTCTCTAATTCAAAACCGAACATGAAATTTTGATTTCATTCGGCTCCTTTATGGA

TATTCTCACCACTTAACATCTATGTCAGCTTTTCTATCTGAATGGAACCAAAGCTCTCCG

CTTTCTAGATGATCCCTATAGAGTAGGAGATAGAAATTCTACTAAATCTATCTAATCTAC

TTACTTCGTTCCCTAATTTCATTCAAGAGACCCTGAGGAAAAGAATTAGGTTTCCACCGA

GCTGAAACAATATGCTGATGGTTCTAGTAAACCAAAACTACCGTTTTTTAGCTATTTGGC

TTCCATTTCCTTTTTAACAAAAGAAGATTTAGTTACGATTGGAAATAAACTTTTTTGTAT

CTTCATCCATAGATCCTTTACTCATATTTTAAAAATTGGAATACTTAATCCAATGCAAAA

TTATGCTTCGCGACTCTGTACTCATAATCCAATTTGTATTTTGGATGCAATTTCAATTAG

TTTTTGGGTACAAATCGCGAGAATGTATATTCTTCCTCAATATGCTATTGAGAGGAAAAG

GATTAAATCCTTTATAAGAACTAAAGTTTTCATCGGAATATAAAAAACTTAAGGACGCCT

TAAGTATATCATTTCAAATTCAGTTATTAATAGAACGAATCACACTTTTACCACTAAACT

ATACCCGCTACATGTAGATTATGATACCAACGCTACCCTTTGTCAAGGGTAGCCATTCGA

GAAGGAGGCTAATTCCCCCTTATTGAATCAAAGGAGAAGGTTCATGACAGTGAGCTGTTG

GTACTTCGATCGCGGGCCTTTATTTCTTTCTTTTTTGTTCAATTCTGAACAAAGAAATTG

GGGAAGATGTTTTCTTCCCCCACTTATCATGAAGTCCGAGCCCTAGAGAAAGAGTGAGAT

GCTTTTAAAAATTCATCATAGACTTTCCCTATGGCTTGAGAGAAGCAAGAAATAACTTAA

ACGGAGAAGCGGACAGGACCCGCTGGTTAGTCGATCCTCTCCATTTACCAATTTCTTCTC

CTCTTTTCCACTCAATTCTAGTTTATTAGATTCTTGTTTAAAAGAATCAAAGAAGATGAA

TAGAACTAAGAACACATAAAAAAAGCATAGAGGACCATTACCAAATGTTCCTCCCAAGAA

TCATATTGGGTATCTGTTCCCTTCCTTTTCCCGCTAGGATCGGGAATCTAGAATCCTCCT

TTTTCCTAATCTCGGAAACAGAAAACCCATAGCCAGGAGCAGTATAAATTCGACTGCCCA

CTTTTTACAAGCAAATTGTTGCTAAAACTCCAACATAGTTTGTTCAAAATGCACCAGAAT

CCCTTGAGAATATTCAAGTACCCCCCTTCCAAGGGGTACCTGTTAAAAATAGTTTCAATT

TCTCACCAAAACAGACAAGAAGTATATCACTGAAAATTAATACCCAACCATATGGGTATA

TGAAGAGCGCGAATTCCTTTATACCCTACCCAATTAGAAGAAATAAAACATAAATGGAGA

AAGTTCTCATCATAAGATCAAAAAAAACCTCTACTTTGTGCAAGTGATAAGAGAGAATGA

AATTCTTATTTTTCTTGATTTTTTTGAACCTCGCCATGAATAAACTTCTATATCTCGATA

TATACATATATTATGTACATTATGCAGTAGACTCATAATGAGAAATCAAAGTGGCTAATT

ATTGAATATCATAAAGGGCTTTTTATTTGGTGGTAGAGTAATGCCATGGTAAGACGTAAG

TCATCGGTTCGAATCCGATAAAGTACTTTTCTACTAAATTTATTCATTTCTTTTTTGAAA

ATTTCTCTTTTTTTCTTGAATTTTATGACTTAGTGTGGGATGCATGCATTTTTGGTCTGA

ACGCTAAACGAGCACGGGGTGGAAATTACAAAAAAGAAATTGGACTCTAGATCAATCAAT

ACCTGTACTGAACTAATCTAGAATAATCTATTCTTATTCTATACCCTTTAAATGAATTTC

CCTAAAAAGTAGGGAATGATCCGTGAATTAACCTAACCATCAACTAAAAAAAATCCTATG

AAAGCATAACAGAAAAGTAGGAAAGACTCTTTGCTTGATCTAGTTCTTCGAGTATATGAC

AATTCCAAAAAACTGCTCATACTATCATTATAGTATAATGACGAGCGGTTGTATATGGCC

CTATCGTCTAGTGATGCCCCTATCGTCTAGTGGTTCAGGACATCTCTCTTTCAAGGAGGC

AGCGGGGATTCGACTTCCCCTGGGGGTAGGGAGTATTATGAAAGGAGGTTAATCATAGAT

TCTAAAAAACCCTAGAATAAATTCTTCCTGGGTCGATGCCCGAGCGGTTAATGGGGACGG

ACTGTAAATTCGTTGACGATATGTCTACGCTGGTTCAAATCCAGCTCGGCCCAAAAATCT

AGGGCTTCGTGAATATGAACTAAATCCATTTTTTTCTTCCATAAAATAAAATGTCTGATC

CATAGAAATAAAGGATAAAGCGAAAGGGGGAAATTTCTTTCTAATCCATATCTCTCTCAT

TCCTTTTTTACAAACAAAAGAGTTTTTCTTATTGAAGGTGGATTATCATCCATTTTTAGC

GATAAAAAATCGCGACATACTAGTTATGTCACTCTCACTATACCCACATATGATATGTGG

GTATGTAGTATATGATTCGTCTATTTTTAGAGTACGACAGGCGAATCGAATCTTCTATTT

AGGTATGCCATACACCCCGCGGGGATTGTAGTTCAATTGGTCAGAGCACCGCCCTGTCAA

GGCGGAAGCTGCGGGTTCGAGCCCCGTCAGTCCCGAACTAGGGTTCAATGAATGGAGAAA

TTCATCTTTCCTTTTTCCATGAAAAAGGGGGGGCAGGAGAGAAGATCAAATACCTATGGG

GCACCCTTATTTCACTTTTTTATTTCGCATTTCTCATTAAGAGGGAGGGGTATAGGATTT

TTTTTCACTACTCCCGGTTGATAAGGAAAGACATACATATCATACTTGGAGGATCTTCCT

ATGTTATACTATTCCACTCTCAACCATGAATTGATTTGATAGATCCGATATTCATAATAT

TGAATTGATTCAGTATTATCAGAATGCAAGTCCTCCCCTTGAATTTACAGGATACCCTTT

TTCCTCTCCATGGGATTACATCCCGAGTTATTGTGAAAAAAAAAGAGGTTATGGAAGTCA

ATATTCTCGCATTTATTGCTACTGCACTGTTTATTCTAGTTCCTACTGCCTTTTTACTTA

TTATTTATGTAAAAACAGTCAGCCAAAATGATTAATTGGAATTCCAATTAATCATTGAAG

AAATGAAAAAGGGATTAAATAAAATAAAAATCCAAGTCTTAAATGAAAGGATCTGGTTGG

AATCATAAAGTGTGGTAGAAAGAACTACATATAGTTTTTTCTACGACACTTTAGAGTCTT

TCTATTATATTATCTTGAATCTACATAGAATAGATTAGTAGATTGAAATAGTAGTCTAAT

TCAATTTCTTTTTTCACTGCATCCACTTAATTTCAATCAAGTCAAAATGAAAGAATCCAT

GGAGGGAGAGAAAAATAATATGAGAATAGACTATAGAAAAGAAAAAAGTAAAAGAAAAAA

CCAGCGAATCTTTCATGCTTAAACATGCGGCGAGATGCTTCAAAAGAGCATAAAAATTAT

TTAAGAATAAGAAAAGAATATAAATGGAAAGTGTGCGATATGTTGTGAATAGCTCCGTGG

AAGAAAGTCTAATTTTCTTATGTATAGAACTTTTTTAACCATTCGTCACTTCTAGTAGAA

ATTTTGAATTGCTGTAATCGCTCTTTCTATTTCTATATAGTAGAATAGAACGACTCTTTC

TTACAAGAGTTTCTTACAGGAGTGAAACAAAACTAAAGAAAGAAAGAATAAAGTTTGGCA

AAATGATTAATGCAAAACGATCAATTAAAGAAAAAAGTTGATACAACAATTCGACTACTC

AATCAATTAGTAGTATCCCTAGAGTCCACTCCTCCCCCATACTACTAGTGAAAGAGAAAA

TGTAAAGACTACCATTAAAGCAGCCCAAGCGAGACTTACTATATCCATGTAAATTATGTC

TCCTATTTCTATGAAGGAATTATTCTACTATTGATCAATAATCATAGTGGAATCAAGGGT

ACAGAGTCAAAAAGGGATTCTGCCCTAACGCTATGGATGAATCAGTTCAAGGAATTTACT

CCTAACAAATTCTTATAGGATTTCTGGTAGAATTGGAGAGCATTAAGTATAAATACGATA

CATAGCCCTTTCTATTAATAAAAGAATAAGGAAACGCAACCTCATCCTTATTGGTAGCCG

TTTGGGCCACTACCGACAAAACAAACCCTAATAGAACTATGGATTCTCAAAATCCAGTAT

CGCCAGGCCTAGTTACTCTCTTGCCCCAACTTAGCAGGGTACGAATTTGTTGAGTTCGAT

CAGTACTATAAGCCTAAGTATTTTATTGATCAGGCGGCACCCAGATTTGAACTGGGGATA

AAGGATTTGCAGTCCCCTGCCTTACCGCTTGGCCATGCCGCCAAAAAATCCGATCTAAAA

TAGAGAAAAGAGCAAGTATTCATCCAGGTTTCTTACTAAAACCTCCTTTCTTTTATCTTG

AATCTAATTCTACTTACTTTTTTCCAATCTTTTTCAAAAAAATTCCTGCTTTTTTGAATC

CAGTTTCGATTATTCTCCTCGATGGATTCTATCTTAAAACAAACATTGCTAACACTAGAA

AACTTCCCTTTTCTTTCTATTGAGATGAAAAAAGAGAAAAGTGGATTTCCAGTCACAGGC

TGCAAAATTCAGAACAAATTGGAACCATTAACTAGAATTCTATTTTTTGAATTTCGGTAT

TCTCCCCCCTTCCTTTTAATGGCATAATAAAATAGAATGGATTTATGCCTAATCCGTGTA

TAGGTAAACTACAGGTCCGAACAGCATTATTATCCATGGATCCCCCTTATGTACATATCT

CTATGGGGAATCGTGCTTTAATTTTTCATTGCATTAAATATCTTGAATAAAAAAAAGAAA

TTTGGTCTGATATGGAGAGGTGGATAACTAGATTGGCATGTACTTAAAAAAGGACTTACT

TTATTTTAGGATTCTACAATGAAATCCTATATTTTCTAGCAATTCTACTACTACGAAACA

AAAAAGAACCCTCAAATTCTTTTTAAAGGAGATAAAATGAGAAATCTTTGCCATCCAATC

TGATTATATCATTAAGTGGCAGAATTTTTTTCTAGGAATGTTTTATCAATTCATTTTCAT

TCGATTTGTACCCCTGGCAAATTCGAACTTTCGTCGAAATTGTCTCTATTCATATGTATG

AAATACATATATGAAATATGTATGTGGAGTTCCCTAGAATTTCATGTGATTCAGTAAACA

GAATATGGATTCCATAATTGCTAGATCGATCCATAGGGATTGATGAAGAGTGAGCTGATA

ATGGAATTTTTCTTCGATAAACAGGAAACTTAAGATGCTCCGGAATGGAAATGAGGGAAT

GTCCACAATACCCGGATTTAGTCAGATCCAATTCGAGGGATTTTGTAGGTTCATTAATCA

AGGCTTGGCAGAAGAACTTGAGAAGTTTCCAACAATTAAAGATCCAGATCACGAAATTGC

ATTTCAATTATTTGCGAAAGGATATCAATTGCTAGAACCCTCGATAAAAGAAAGGGATGC

TGTGTATGAATCACTCACCTATTCTTCCGAATTATATGTATCTGCGAGATTAATTTTTGG

TTTCGATGTGCAAAAGCAAACCATTTCTATTGGAAACATTCCTATAATGAATTCCTTAGG

AACCTTTATAATAAATGGAATATACCGAATTGTGATCAATCAAATATTGCTAAGTCCTGG

TATTTACTACCGCTCGGAATTAGACCATAAGGGAATTTCTATCTACACTGGGACTATAAT

ATCAGATTGGGGAGGAAGATCGGAATTAGCAATTGATAAAAAAGAAAGGATATGGGCTCG

CGTGAGTAGAAAACAAAAGATATCTATTCTAGTTCTATCATCAGCTATGGGTTCGAATCT

AAAAGAAATTCTAGATAATGTTTCCTACCCTGAAATTTTCTTATCTTTCCCGAATGCTAA

GGAGAAGAAGAGGATTGAGTCAAAAGAAAAAGCTATTTTGGAGTTTTATCAACAATTTGC

TTGTGTAGGTGGGGACCTGGTATTTTCGGAGTCCTTATGTGAGGAATTACAAAAGAAATT

TTTTCAACAAAAATGTGAATTAGGAAGGATTGGTCGACGAAATATGAATCGGAGACTGAA

TCTTGATATACCTCAGAACAATACATTCTTGTTACCACGAGATGTATTGGCCGCTACGGA

TCATTTGATTGGAATGAAATTTGGAACGGGTATACTTGACGATGACGATATGAATCACTT

GAAAAATAAACGTATTCGTTCGGTTGCGGATCTGTTACAAGATCAATTCGGACTGGCTCT

TGGTCGTTTACAACATGCGGTTCAAAAAACTATCCGTAGAGTATTCATACGTCAATCGAA

ACCGACTCCACAAACTTTGGTAACTCCAACTTCAACTTCGATTTTATTAATAACTACTTA

TGAGACCTTTTTTGGCACATACCCCTTATCTCAAGTTTTTGATCAAACCAATCCATTGAC

ACAAACTGTTCATGGGCGAAAAGTGAGTTGTTTGGGTCCTGGAGGATTGACGGGGAGAAC

TGCAAGTTTTCGGAGCCGAGATATTCATCCGAGTCACTATGGGCGTATTTGTCCAATTGA

CACGTCCGAAGGAATCAATGTTGGACTTACTGGATCCTTAGCTATTCATGCGAGAATTGA

CCACTGGTGGGGGTCCATAGAGAGTCCCTTTTATGAAATATCTGAGAAAGCAAAAGAAAA

AAAAGAGAGACAGGTGGTTTATTTATCACCAAATAGAGATGAATATTATATGATAGCAGC

AGGAAATTCTTTGTCCTTGAATCAGGGTATTCAGGAAGAACAGGTTGTTCCAGCTAGATA

CCGTCAAGAATTCCTGACTATTGCATGGGAACAGATTCATGTTAGAAGTATTTTTCCTTT

CCAATATTTTTCTATTGGAGGTTCTCTCATTCCTTTTATTGAGCATAATGATGCGAATCG

GGCTTTAATGAGTTCTAATATGCAGCGCCAAGCAGTTCCGCTTTCTCGGTCCGAGAAGTG

CATTGTTGGAACTGGATTGGAACGCCAAACAGCTCTAGATTCGAGGGTTTCCGTTATAGC

CGAACGCGAGGGAAAGATCATTTCTACTGATAGTCACAAGATCCTTTTATCAAGTAGTGG

GAAGACTATAAGTATTCCTTTAGTTACCCATCGGCGCTCTAACAAAAATACTTGTATGCA

CCAAAAACCTCGGGTTCTGCGGGGTAAATCCATTAAAAAAGGACAAATTTTAGCGGAGGG

AGCTGCTACAGTTGGTGGGGAACTTGCTTTAGGAAAAAACGTATTAGTAGCTTATATGCC

ATGGGAAGGTTACAATTTTGAAGACGCAGTACTAATTAGCGAACGTTTGGTATATGAGGA

TATTTATACTTCTTTTCACATCCGAAAATATGAAATTCAGACGGATACGACAAGCCAAGG

CTCCGCTGAAAAAATTACTAAAGAAATACCACATCTAGAAGAACATTTACTCCGCAATTT

GGACAGAAATGGAGTTGTTAGGTTGGGATCCTGGGTAGAAACTGGCGATATTTTAGTAGG

TAAATTAACGCCTCAGATAGCGAGCGAATCGTCGTATATCGCGGAAGCTGGGTTATTACG

GGCCATATTTGGCCTTGAGGTATCCACTTCAAAAGAAACTTCTCTCAAACTACCTATAGG

TGGAAGAGGGCGCGTTATCGATGTGAAATGGATCCAGAGGGACCCCCTAGACATAATGGT

TCGTGTATATATTTTACAGAAACGCGAAATCAAAGTTGGGGATAAAGTAGCCGGAAGACA

CGGGAATAAGGGGATCATTTCCAAAATTTTGCCCAGGCAAGATATGCCCTATTTGCAAGA

TGGAACACCTGTTGATATGGTCTTCAATCCCTTAGGAGTACCCTCACGAATGAATGTGGG

ACAAATATTTGAAAGCTCGCTCGGATTAGCCGGGGATCTGCTAAAGAAACATTATAGAAT

AGCACCCTTTGATGAGAGATATGAGCAAGAGGCTTCAAGAAAACTTGTGTTTTCAGAATT

ATATGAAGCCAGTAAACAAACAAAAAATCCATGGGTATTTGAACCCGAGTACCCGGGAAA

AAGCAGAATATTTGATGGAAGAACAGGAGACCCCTTCGAACAACCTGTTCTAATAGGGAA

GTCCTATATCTTAAAATTAATTCATCAAGTTGATGAGAAAATCCATGGACGTTCTACTGG

GCCCTACTCACTTGTTACACAACAACCCGTTAGAGGAAGAGCCAAGCAAGGGGGACAACG

AGTAGGAGAAATGGAAGTTTGGGCTTTAGAAGGATTTGGTGTTGCTCATATTTTACAAGA

GATACTTACTTATAAATCTGATCATCTTATAGCTCGCCAAGAAATACTTAATGCTACGAT

CTGGGGAAAAAGAGTACCTAATCACGAGTATCCTCCAGAATCTTTTCGAGTGCTCGTTCG

AGAACTACGATCTTTGGCTCTAGAACTGAATCATTTCCTTGTATCTGAGAAGAACTTCCA

GGTTAATAGGGAGGAAGTTTGATCGGAATAAATATAAATTCTTTTCTTATTTATGATTGA

CCAATATAAACATCAACAACTTCAAATTGGACTCGTTTCCCCTCAACAAATAAAGGCTTG

GGCTAACAAAAACCTACCTAATGGGGAAGTCGTTGGCGAAGTCACAAGGCCCTCTACTTT

TCATTATAAAACCGATAAACCAGAAAAAGATGGATTGTTTTGCGAAAGAATCTTTGGACC

CATAAAAAGCGGAATTTGTGCTTGTGGAAATTCTCGAGCGAGCGGAGCTGAAAACGAAGA

GGAAAGATTTTGCCAAAAATGCGGGGTAGAATTTGTTGATTCTCGGATACGAAGATATCA

AATGGGATACATCAAACTCGCATGTCCCGCGACTCATGTGTGGTATTTGAAAGGTCTTCC

TAGTTATATCGCGAACCTTTTAGATAAACCCCTTAAGAAATTGGAGGGCCTAGTATACGG

CGATTTCTCTTTTGCTAGGCCCAGCGCTAAAAAACCTACTTTCTTACGATTACGAGGTTT

ATTCGAAGATGAAATTGCATCCTGTAACCACAGCATTTCTCCCTTTTTTTCTACCCCAGG

CTTTGCAACATTTCGAAATCGGGAAATTGCGACAGGAGCAGGTGCTATTAGAGAACAATT

AGCAGATTTGGATTTGCGAATTATTATAGAGAATTCCTTGGTCGAATGGAAGGAATTAGA

AGACGAGGGGTATAGTGGAGATGAATGGGAAGATAGAAAAAGACGAATAAGAAAAGTTTT

TTTGATTAGACGCATGCAATTGGCGAAACATTTTATTCAAACAAATGTAGAACCAGAATG

GATGGTTTTGTGCTTATTACCAGTTCTTCCTCCCGAATTGAGACCCATTGTTTATAGGTC

TGGGGATAAAGTAGTGACTTCGGATATTAATGAACTTTATAAGAGAGTTATTCGTCGGAA

CAACAACCTTGCCTATCTATTAAAAAGAAGTGAATTAGCGCCAGCAGATTTAGTAATGTG

CCAGGAAAAATTGGTACAAGAAGCCGTGGATACACTTCTTGATAGTGGGTCCCGCGGGCA

ACCAACGAGGGATGGTCACAATAAAGTATACAAATCACTTTCAGATGTAATTGAAGGTAA

AGAGGGAAGGTTTCGCGAGACTCTGCTTGGAAAACGGGTCGATTACTCGGGGCGTTCTGT

CATTGTTGTGGGTCCTTCGCTTTCATTACATCAATGTGGATTACCTCTAGAGATAGCAAT

AAAGCTTTTTCAGCTATTTGTAATTCGCGATTTAATCACGAAACGCGCTACTTCTAATGT

CAGGATTGCTAAAAGGAAAATTTGGGAAAAGGAACCCATTGTATGGGAAATACTTCAAGA

AGTTATGCGGGGACATCCTGTACTGTTGAATAGAGCACCCACCCTGCATAGATTAGGCAT

ACAGGCCTTCCAACCTACTTTAGTGGAGGGGCGTACTATTTGTTTACACCCATTAGTGTG

TAAGGGTTTCAATGCGGACTTTGATGGGGATCAAATGGCTGTTCATCTACCTTTATCCTT

GGAAGCTCAGGCGGAAGCCCGTTTACTTATGTTTTCTCATATGAATCTCCTATCTCCCGC

TATTGGGGATCCTATTTGCGTACCGACCCAAGACATGCTTATCGGACTTTATGTATTAAC

GATTGGAAACCGTCGGGGTATTTGTGCAAATAGATATAATAGTTGCGCAAACTATCCAAA

TCAAAAAGTAAATTACAATAATTATAAGTATACAAAAGATAAAGAACCCCATTTTTCTAA

TTCCTATGATGCACTGGGAGCTTATAGACAGAAACGAATCAGTTTAGACAGTCCCTTGTG

GCTCCGATGGAAACTAGATCAACGCGTCATTGGGTCAAGAGAAGTTCCGATTGAAGTTCA

ATATGAATCTTTGGGGACTTATCATGAGATTTATGCCCACTATCTAATAGTGGGAAATAG

AAAAAAAGAAATCCGTTCTATATACATTCGAAGCACTCTTGGTCATATTTCTTTTTATAG

AGAAATAGAGGAAGCCATACAAGGATTTAGTCAGGCCTATTCATACACTATCTAAACAAG

GAAGTTAGATTCGGCGATGCCTTTCGGGGGGCATTCCGATTTCGCTAGTATCATCATTTT

TGCCGCGCGAATCCAGATTGAGATTAAGGAAAGGAAGTTAATTAAATTTTGAATCACTGA

CTCAGGCCCATTGTCGAATCCTACTCAGCAATTGTCGAATCCTACTCAGCCGAAAAAGGG

GGTACTTATGTATGGCGGAACGGGCCAATCTGGTCTTTCATAATAAAGAGATAGACGGAA

CTGCTATGAAACGACTTATTAGCAGATTAATAGATCATTTCGGAATGGGATATACATCCC

ATATACTGGATCAAATAAAGACTCTGGGCTTTCATCAAGCCACTACTACATCGATTTCAT

TAGGAATCGAGGATCTTTTAACAATACCATCTAAGGGATGGTTAGTGCAAGACGCGGAAC

AACAGAGTTTTCTTTTGGAGAAACACTATTATTATGGGGCTGTACACGCGGTAGAAAAAT

TACGCCAATCCGTTGAGATATGGTATGCTACAAGTGAATATTTGAAACAAGAAATGAATT

CGAATTTTCGGATAACGGATCCTTCTAATCCAGTCTATCTAATGTCTTTTTCAGGAGCCA

GAGGAAATGCATCTCAGGTACACCAATTAGTAGGTATGAGAGGATTAATGGCGGATCCTC

AAGGACAAATGATTGATTTACCTATTCAAAGCAATTTACGCGAGGGACTTTCTTTGACAG

AATATATAATTTCCTGCTACGGAGCCCGCAAAGGGGTTGTAGATACTGCTGTACGAACAG

CGGATGCTGGATATCTTACACGTAGACTTGTTGAAGTAGTTCAACATATTATTGTGCGTA

GAAGAGATTGTGGTACTATCCAAGGTATTTCTTTGAGTCCTCAAAATGGGATGACGGAAA

AACTTTTTGTCCAAACACTAATTGGTCGTGTATTAGCAGACGATATATATATTGGTTCAC

GATGCATTGCCGCTCGAAATCAAGATATTGGAATTGGATTAGTCAATCGATTCATAACTG

CCTTTCGAGCACAACCATTTCGAGCACAACCAATATATATTAGAACCCCCTTTACTTGCC

GGAGCACATCTTGGATCTGTCAATTATGTTATGGTCGGAGTCCCACTCATGGCGATCTGG

TCGAATTGGGGGAAGCCGTAGGTATTATTGCAGGTCAATCAATTGGGGAGCCAGGGACTC

AACTAACATTAAGAACTTTTCATACTGGCGGAGTATTCACAGGGGGTACTGCCGACCTTG

TACGATCCCCTTCGAATGGAAAAATCCAATTCAATGAAGATTTGGTTCACCCCACACGTA

CCCGTCATGGGCAGCCTGCTTTTCTATGTTATATAGACTTGCATGTAACTATTCAGAGTC

AGGATATTCTATATAGTGTGAATATTCCCTCAAAAAGCTTGATTCTAGTGCAAAATGATC

AGTATGTAGAATCCGAACAAGTAATTGCGGAGATTCGTGCCGGAACGTCCACTTTGCATT

TTAAAGAAAAAGTACAAAAGCATATTTATTCCGAATCAGACGGGGAAATGCACTGGAGTA

CTGATGTTTACCATGCGCCCGAATATCAATATGGTAATCTTCGTCGATTACCAAAAACAA

GCCATTTATGGATATTGTCAGTAAGTATGTGCAGATCCAGTATAGCGTCTTTTTCGCTCC

ACAAGGATCAAGATCAAATGAATACTTATTCTTTTTCTGTTGACGGAAGATATCTCTTTG

ACCTCTCAATGGCTAATGATCAAGTAAGACATAGACTGTTGGATACTTTTGGTAAAAAAG

ATAGGGAAATTCTTGATTATTCAACGCCGGATCGAATCATGTCCAATGGCCATTGGAATT

TTGTCTATCCTTCTATTCTTCAAGATAATTCGGATTTGTTGGCGAAAAAGCGAAGAAATG

GGTTCGTCATTCCATTACAATATCATCAAGAACAAGAGAAAGAACTAATATCCTGTTTGG

GGATTTCGATTGAAATACCCTTTATGGGTGTTTTACGTAGAAATACTATTTTTGCTTATT

TTGACGATCCACGATACAGAAAAGATAAAAAGGGTTCAGGAATTGTTAAATTTAGATATA

GGACCCTAGAGGACGAATATAGGACTCGAGAGGAAGACTCAGAGGACGAATATGGGACAG

AGAACGAATATAGGACCCGAGAGGAAGAGGACGAATATGAAACCCTAGAAGATGAATATG

GGATCCTAGAGGACGAATATGAAGCCCTAGAAGACGAATATAGGACTGGAGAGAAAGACT

CAGAAGACGAATATGGGAGCCCAGAGAACGAATATAGAACCCGAGAGGACGAATATGGAA

CTCTAGAGGAAGACTCAGAGGACGAATATGGGACTTTAGAGGAAGACTCAGAAGAAGACT

CAGAGGACGAATACGGGAGCCCAGAGGAAGATTCCATCTTAAAAAAAGAGGGTTTGATTG

AGCATCGAGGAACAAAAGAATTTAGTCTAAAATACCAAAAAGAACTAGATCGGTTTTTTT

TCATTCTTCAAGAACTGCATATCTTGCCGAGATCCTCATCCCTAAAGGTACTTGATAATA

GTATCATTGGAGTGGATACACAACTCACAAAAAATACAAGAAGTCGACTAGGTGGATTGG

TCCGAGTGAAGAGAAAAAAAAGCCATACGGAACTCAAAATCTTTTCCGGAGATATGCATT

TTCCTGAAGAGGCGGATAAGATATTAGGTGGTAGTTTGATACCACCAGAAAGAGAAAAGA

AAGATTCTAAGGAATCAAAAAAAAGGAAAAATTGGGTCTATGTTCAACGGAAAAAAATTC

TCAAGAGCAAGGAAAAGTATTTTGTTTCGGTTCGACCTGCAGTCGCATATGAAATGGACG

AAGGGAGAAATTTAGCAACACTTTTCCCGCAAGATCTCTTGCAAGAAGAGGATAATTTCC

AACTTCGACTTGTCAATTTTATTTCTCATGAAAATAGCAAGTTAACTCAAAGAATTTATC

ATACGAATAGTCAATTTGTTCGAACTTGCTTAGTAGTGAATTGGGAACAAGAAGAAAAAG

AGGAGGCTCGTGCTTCCCTTGTTGAGGTAAGAGCAAATGATCTGATTCGCGATTTCCTAA

GAATTGAGTTAGTCAAGTCCACTATTTCGTATACACGAAGAAGGTATGATAGGACAAGTG

CAGGACCGATTCCCAATAATAGGTTAGATCGCACCAATTCCTTTTATTCCAAGGCGAAGA

TTCAATCACTTAGCCAACATCAAGAAGCTATTGGCACCTTGTTGAATCGAAATAAAGAAT

ACCAATCTTTGATGATTTTGTCGGCATCCAACTGTTCTCGAATTGGTTTATTCAAGAATT

CGAAATATCCCAATGCGGTAAAAGAATCGAATCCTAGAATTCCTATTCGAGATATTTTTG

GGCCCTTAGCCGCTATTGTACCTAGTATATCGAATTTTTCTTCATCTTACTATTTACTAA

CGCATAATCAGATCCTGTTAAAAAAATATTTGTTCCTTGACAATTTGAAACAAACCCTCC

AAGTACTTCAAGGGCTTAAATACTCTTTAATAGATGAAAATCAAAGGATTTCGAATTTCG

ATAGTAACATCATGTTGGATCCATTCCATTTGAATTGGCACTTTCTCCATCATGATTCTT

GGGAGGAGACATCGGCAATAATTCACCTTGGACAATTTATTTGCGAAAATGTATGTCTAT

TTAAATCGCACATAAAAAAATCTGGTCAAATTTTCATTGTTAATATTGATTCCTTTGTTA

TAAGAGCAGCTAAGCCTTATTTGGCCACTACAGGAGCAACTGTTCATGGTCATTATGGAG

AAATCCTTTACAAAGGAGATAGGTTAGTTACGTTTATATATGAAAAATCGAGATCTAGTG

ACATAACGCAAGGTCTTCCAAAAGTAGAACAAATCTTTGAAGCGCGTTCAATTGATTCAC

TATCGCCGAATCTCGAAAGGAGAATTGAGGATTGGAATGAGCGTATACCAAGAATTCTTG

GGGTCCCCTGGGGATTCTTGATTGGAGCTGAGCTAACCATAGCCCAAAGTCGTATCTCTT

TGGTTAATAAGATCCAAAAGGTTTATCGATCCCAAGGGGTACAGATCCATAATAGACATA

TAGAGATTATTATACGCCAAGTAACATCAAAAGTGCGGGTTTCCGAAGATGGAATGTCTA

ATGTTTTTTCACCTGGGGAATTAATCGGACTATTACGAGCAGAACGAGCAGGACGAGCTT

TGGATGAATCGGTCTATTATCGGGCAATCTTATTGGGAATAACAAGGGCTTCCCTGAATA

CCCAAAGTTTCATATCTGAAGCAAGTTTTCAAGAAACTGCTCGAGTTTTAGCAAAAGCTG

CCCTACGAGGTCGTATTGATTGGTTGAAAGGCCTGAAAGAAAACGTAGTTCTGGGGGGGA

TTATACCTGTTGGTACCGGATTCCAAAAATTTGTGCATCATTCCCCACAAGACAAGAACC

TTTATTTCGAAATTCAAAAAAAAAATCTATTCGCGTCGGAAATGAGAGATATTTTGTTTC

TCCATACAGAATTAGTTTCTTCTGATTCTGATGTAACAAACAATTTCTATGAGACATCAG

AACCCCATTTATACGATTTAAGGATACATAAAGCAGATTTTTTTATTTAAACTAGACTTT

TGACCTTAGAACACTAACAGGTCAGATTTTGATTTTTATTAATAAGTAAAGAAGTCAGTT

AATTCATTAAGGTTACGTTTATACCATGTAGAAGGTTACATCGGAACAATTATTATTTAT

TTCAAGCTATTTCGGCTCTTTCTTAATTTTCAAAAAGAAATAAATTCCGTAATGGAAAAA

AAAGAAAAAATCAAAAGGAAGTGTGGAAAAAATGACAAGAAGATATTGGAACATCAATTT

GAAAGAGATGATAGAAGCGGGAGTTCATTTTGGTCATGGTATTAAGAAATGGAATCCTAA

AATGGCCCCTTACATCTCGGCAAAGCGTAAAGGTACTCATATTACAAATCTCGCTAGAAC

GGCTCGTTTTTTATCAGAAGCTTGTGATTTAGTTTTTGATGCAGCAAGTCAGGGAAAAAG

CTTTTTAATTGTTGGTACCAAAAAAAGAGCAGCGGATTTAGTAGCATCAGCTGCAATAAG

GGCTCGTTGTCATTATGTTAATAAAAAGTGGTTCAGTGGTATGTTAACGAATTGGTCGAT

TACGAAAACTAGACTTTCTCAATTTAGAGACTTAAGAGCAGAAGAAAAGATGGGAAAATT

CCACCATCTCCCAAAAAGAGATGTGGCAATCTTGAAGAGAAAATTATCTACCTTGCAAAG

ATATCTCGGCGGGATCAAATATATGACGAGGTTGCCGGACATTGTGATCGTCCTTGATCA

GCAAAAAGAGTATATAGCTCTTCAGGAATGTGCCATTTTGGGGATTCCTACTATTTCTTT

AGTCGATACAAATTGTGACCCAGATCTCGCAAATATATCGATTCCAGCCAACGATGACAC

TATGACTTCAATTCGATTGATTCTTAACAAATTAGTATTTGCAATTTGTGAGGGCCGTTC

TCTCTATATAAGAAATCGTTGATTAAGAAGAATAGTTCATTCTTGGGTAACTGCGTAGAT

TTATGGGATCACTTACTATTCTTTTTTGTTTTGCATAGATAAAAGAAGGGGAATATTGAT

ATATATTAGAGGGTATTGATATATATTATCATCTGATGTGATTTCTTGGTATCCTAAATA

TAAGATTAATACTTCAAGTTGCTGAGTTGAGAAAGAGATGGTTGAATCAAAAGAATTCCT

TTTTTGAAGTTCAATTTTTATCAGAGGACAATATGAATATTATACCATGTTCCGTTAAAA

CACTCAAGGGGTTTTATGATATATCGGGTGTAGAAGTAGGCCAACACTTCTATTGGCAAA

TAGGAGGTTTCCAAATTCATGCCCAAGTACTCATCACTTCTTGGGTCGTAATTACTATCT

TGCTAGGTTCAGTTATCATAGCTGTTCGGAATCCACAAACCATCCCGACCGACGGTCAGA

ATTTCTTCGAATATGTGCTTGAGTTTATTCGAGACTTGAGCAAAACTCAGATTGGAGAAG

AATATGGTCCCTGGGTTCCCTTTATTGGAACTATGTTCCTTTTTATTTTTGTTTCGAATT

GGTCGGGTGCTCTTTTACCTTGGAAAATTATACAGTTACCCCATGGGGAATTAGCAGCAC

CCACGAATGATATAAATACTACTGTTGCTTTAGCTTTACTCACATCAGCGGCATATTTTT

ATGCGGGTCTTAGCAAAAAAGGATTGAGTTATTTCGAGAAATATATTAAACCAACTCCAA

TTCTTTTACCAATTAACATCCTAGAAGATTTCACAAAACCATTATCGCTTAGTTTTCGAC

TTTTCGGGAATATATTGGCGGATGAATTAGTCGTTGTTGTTCTTGTTTCTTTAGTCCCCT

TAGTAGTCCCTATACCGGTCATGTTTCTTGGATTATTTACAAGCGGTATTCAAGCTCTTA

TTTTTGCAACGTTAGCCGCAGCCTATATAGGTGAATCCATGGAAGGTCATCATTGAATTG

ACTAGTTTTCAAAATAGTCTTTTTTTAGCTTAGCTCAATTCATGCATGGTTCCAGATAAT

CCGCTTGGTTGGAAAACTAAATAGTTAGAAATGCGTATGAATATACAACCTAGAGTTGTA

GGAGAGAGAATAGACTATATTACGTGTCAAAGTATATATGCATTAAGGGGGGCGGAGTCA

GGCTAGATCTATATCCTTAATGTCTATAAGCCAGTCATCTTTTGTGCGGGTTTTTAAGGA

ATGATTTTAGAATCCGATTCAATAGAAAATGAGAAAATACGCAAAATAGAAGAAACAAAT

GTATATGGGATATTATATATTCCTAAGTTAGATTCATTATCTAATCCGATATATGGAATT

GGATTCCATATCCAGTTCTATGCAGCATATTGTTATCAATTGTATATCTTGATTTAATTC

CTATTGGATTTGGATTAGGTCGATTTCAATAGGGGTTCTTCCTCTATTTCGTCTTTTATT

ATGTTAGATGAAGGGGAAAAAATAGGAACTCAAGGATATCGAAGAGTAAAAAGAAGAATG

GAATGAAAGAGTGGTTGGTTGGAAAGAAAGAGAAATAGAATAATGAGTACACAAACCTCT

AATGATTAGAAACTAAAAATGAGATCTCGAAGTAGTTCGGACAATTCAGATTATCATTTA

TTTGTACTTTTTAGTTACTTCTCCCCAATAGAGCTTAGAAGTAAGAATTTCTTGGTTGAT

TGTATCCTTAACCATTTCTTTTTTTTGACACGAGGAACTCACCATGAATCCACTAATTGC

TGCTGCTCCGTTATTGCTGCTGGATGCCGTAGGGCTTGCTTCTATTGGGCCTGGAGTTGG

TCAAGGTACTGCTGCAGGACAAGCTGTAGAAGGTATTGCGAGACAGCCAGAAGCAGAAGG

TAAAATACGAGGTACTTTATTGCTTAGTCTAGCTTTTATGGAAGCTTTAACAATTTATGG

ACTAGTTGTGGCACTAGCGCTTTTATTTGCGAACCCTTTTGTTTAATCCTAAAAAAGAAA

ATGAGTCCTTTAGATTAGATACTTTTTTCTTTTTTAGTAAATTGGTATTTGCTTCTGCAA

TTCCAATTATATCAATACTTTACTCCTATTTATTACTCCTGGAATTACCTATTTATCGGG

ACAGACAATACCCCACCCCAGGAAGGGCTGATTTGAGGATGATCAATTTAGAGGATATGC

TCGCCTTCTTCCTTCCCGTCCTTAGTTTAGGACAGTGGAAAGTCTTTTTCCTTTTATTTT

AGGAATTTTTGGAACATTTCAACAAAGGAGTCTTTCACAGGTCAAACGAGACCTAAGACT

TAATCTAAAAGAAATTATTAGATTGAATCTATTTGCATTAAAAAACCGATCAAAAAGGGC

GAGCGAAGTAAGTGATCAAAAACTTTGCTCTTTGTTCGTCCTATCTATAAGAGGAGAGCA

TATGAAAAATGTAACCCATTCTTTCGTTTTTTTAGCTCACTGGCCATCCGCTGGGAGTTT

CGGGCTTAATACCGATATTTTAGCAACAAATCTAATAAATCTAACTGTAGTGGTTGGTGT

ATTGATTTTTTTTGGAAAGGGAGTGTGTGCGAGTTGTCTATTTCAAGAATAGATTGATCT

ATCCGGCTGCACTTTAAAATATTTTTTAGTATTTTTTGGATAAATAAGAAAAGGTGCACG

ATCTCGACGAATTACTTCTGAATAAATTCAGAAATCATATGGAAGACCATAGCATTTCGC

GACTCATTGGTAAATCAACTTTGATTCTCTATAGACCAATAATGTGAGACCATTAACACG

GTTAAAGCTAAACTGCTTGAAGTCCAGGCAAAAAGGGGTACTCTTTCTACAACTATATTA

GTATTAGTACCAAATGCTTTAAACGGGAAATAGCTAATGTAGAATTTATCTGATATAAAA

CACTCATATCGATAAAATGGTTTGAACTATTTACTAGAAGGGCACCCTGCCCTTTTTCCA

ATGCCGAATCGACGACCTATGTATAAAAAAAGAGAAATTTTTTGGATTTGAAGAAAAAAA

AGAATTCTATCAATTTTCATTTTCCATTTATTTAGTTTCTTAATGAAATTGAAATTATTA

ACTAAAGGGCAAATACAAATAAAGAAACAACTTTGCTGACCATGATAGATTTTTATCTAG

GCGGAAGAGTCCTCTTAATATTTATCTAGTCTTATATGGGTTTCGGTATATTGAAATATA

AACAGAAAAGAGAAGATAGAGGATAGGCTCATTACATAAAAAAGATATGGAAATAGCCAT

AGCAAAAAAAAAAAAGGAGCGTGAGAGCCAAATGAATCGAAAGATTCATGTTTGGTTCGG

GAAGAGATCATAAAAATTGTAAACTTAATAGCAAGATAATCTACTTTCATTAAAAGATTT

ATTAGATAATCGAAAACAGAGAATCTTGAGTACTATTCGAAATTCGGAAGAATTGCGTAG

AGGGACCATTGAGCAGCTCGAAAAAGCTCGGGTTCGATTACAGAAAGTCGAACTAGAAGC

GGATGAGTATCGAATGAATGGATACTCTGAGATAGAACGAGAAAAAGCAAATTTGATTAA

TGCTACTTCTATTAGTTTGGAACAATTAGAAAAGTCTAAAAATGAAATCCTTTATTTTGA

AAAACAAAGGGCGATGAATCAGGTCCGACAACGGGTTTTCCAACAGGCCGTACAAGGAGC

TCTAGGAACTCTGAATAGTTGTTTGAATACCGAGTTACATTTCCGTACGATTCGTGCTAA

TATTGGCATTCTAGGGGCCATAGAATCAAAGAATTAAATTAATTAGACCTTGAACTTCTA

CTTTCGTTTAGAATTTAGGCATTATTTTTCCCTTGCTTCCGAAAAAAGAGTCAAGAAACA

CTAATGGCAACCCTTCGAGTCGACGAAATTCATAAAATTCTCCGCGAACGTATTGAACAA

TATAATAGGAAAGTAGGGATTGAGAATATCGGTCGCGTAATTCAAGTGGGGGATGGGATT

GCTCGTATTATAGGTCTTGGTGGAATAATGTCAGGTGAATTAGTCGAATTTGCAGAAGGG

ACTAGGGGTATTGCTCTGAATTTGGAATCCAAAAATGTTGGGATTGTATTAATGGGCGAT

GGGTTGATGATACAAGAGGGAAGTTTTGTAAAAGCAACAGGAAGAATTGCTCAGATACCC

GTGAGCGAGGCTTACTTGGGTCGTGTTATAAATGCTCTGGCTAAACCTATCGATGGGAGA

GGCGAAATTGTAGCTTCGGAATCTCGCTTAATTGAATCTCCTGCTCCGGGTATAATTTCC

AGGCGTTCCGTATATGAACCCCTTCAAACAGGGCTTATTGCTATCGATTCGATGATCCCC

ATAGGGCGCGGTCAGCGAGAGTTAATTATTGGGGACAGACAGACTGGCAAAACAGCAGTA

GCCACAGATACAATTCTCAATCAAAAAGGGCAAGATGTAATATGTGTTTATGTAGCTATC

GGTCAAAGAGCATCCTCCGTGGCTCAAGTAGTAACTACTTTCCATGAAGAGGGGGCCATG

GAATACACTATTGTAGTAGCTGAAATGGCGGATTCACCTGCTACATTACAATACCTCGCC

CCTTATACGGGAGCAGCCCTGGCTGAGTATTTTATGTATCGCGAACGGCATACTTTAATA

ATTTATGATGATCTCTCCAAACAAGCACAAGCTTATCGCCAAATGTCCCTTCTATTAAGA

AGACCCCCCGGCCGCGAAGCTTATCCAGGGGATGTTTTTTATTTGCATTCACGGCTTTTA

GAAAGAGCCGCTAAATTAAGTTCTCTTTTAGGCGAAGGAAGTATGACCGCTTTACCAATA

GTTGAGACTCAATCTGGAGACGTTTCCGCCTATATTCCTACTAATGTAATCTCCATTACA

GATGGACAAATATTCTTATCCGCGGATCTATTTAATGCCGGAATTCGACCTGCTATTAAT

GTGGGTATTTCTGTTTCCAGAGTAGGATCCGCGGCTCAAATTAAAGCCATGAAACAAGTA

GCTGGCAAATCAAAATTGGAACTAGCTCAATTCGCAGAATTACAAGCCTTTGCACAATTC

GCCTCTGCTCTCGATAAAACAAGTCAGAATCAATTGGCAAGGGGTCGACGATTACGGGAA

TTGCTTAAACAATCCCAATCAAACCCTCTCTCAGTGGAAGAGCAGATAGCTACTATTTAT

ACCGGAACGAGAGGATATCTTGATTCGTTAGAAATTGGACAGGTAAAGAAATTTCTGGAT

GAGTTACGTAAACACCTAAAAGATACGAAACCTCAATTCCAAGAAATTATATCTTCTAGC

AAGACATTCACCGAGCAAGCGGAAATCCTTTTGAAGGAAGCTATTCAGGAACAGCTCGAA

GGGTTTTCCCTTCAGGAACAAACATAAATTTTGCATGTCTACTCTTGTTAGTAGAAGAGG

AATCAAAGATTTTTCATTTGAATCATGCAAAAAGTTTTCTTAGTTTTTAGTATAGTTATT

TAAAGAATAGATAGAAATAAGATTGCGTCCAATAGGATTTGAACCTATACCAAAGGTTTA

GAAGACCTCTGTCCTATCCATTAGACAATGGACGCTTTTTTCATATTTTCTTCTTTCTTT

TTTTTTTTTTCTTGTGATAAAAAACTCTTAGACCGAAACTCTTTTAGGAAAGAAAAATAA

ATCCATATACAAATGGATGATGCATATATCATAAAGAAGGAATATGGAGCGGGTAGTGGG

AATCGAACCCGCAACCCCAAGGTTATGAGCCTTGTGAGCTACCAAACAGCTCTATCCTCT

TAGCGGGGAACTAGGGGGTTGGATACGCCCCTCTACCATATCTATACAAATAGAATAGTC

CATTTATACAGAATGGTAAAGAGGGCTCTTCTACGATCATCCATTCTAGAAATCCATACA

AATATGAAAGGGTATTTTATCCTTACCAACTGGATCTTGTTGCACCCGGTAACAAACATG

CATAAACCATTTCTCGAAGTATGTGTCCGGATAGTCCAAAGTCTCGATAGTTAGCTCTAG

GTCTTCCGGTCAAAAAACAACGTCGATGAAGGCGTGTAGGTGCACTATTACGTGGTAGGG

ATTGCAATTTTTCTCGCATTTTCGTTTTTTCACTCAAACTCAAGGGGGAAACTTTGCTTC

TTATCTTTTTTTTTGAAGATCGACGAATCAAATGATATTTCTGTTCTAATTTCTGCCGCT

TCTTCTCCCTCTGAATCAAACTTTTTTTTGCCATAATGTGCCGTTCCTATTATTACCAAG

TATATGGTTCTAATCCTAGATGGAAAAATAAATAGAAAAAATCTAAGAAGGCGGATCCTC

CCTCTCCATCAAGAGTAATGAACTAGGTGCTGGTACAGTACAAAACTAAATTAACCAAAC

TTGCCTGATGTTGAGGCAATCAAGAAAGCTGCATAAGTGAATATATAACCCACGGAAAAG

TGGGCTAATCCAACCAATCTTGCTTGCACAATGGAAAGAGCCACGGGCTTATCTCTCCAG

CGAATTAAATTAGCCAAAGGTGTGCGTTCATGAGCCCATGCTAAAGTCTCAATTAATTCC

TGCCAATATCCACGCCAGGAAATTAAGAACATAAATCCAGTAGCCCAAACAAGATGTCCA

AATAAGAACATCCACGCCCATACCGATAAACTATTCATCCCAAAAGGATTATATCCATTG

ATAAGTTGTGAAGAGTTTAACCATAGGTAATCTCTTAACCATCCCATCAAATAAGTGGAG

GATTCATTAAATTGTGAAACGTTGCCCTGCCATAATGTGATGTGTTTCCAATGCCAATAA

AAAGTAACCCATCCAATGGTATTTAACATCCAGAAAACTGCCAAATAAAACGCGTCCCAA

GCAGAAATATCACAAGTACCGCCGCGCCCTGGGCCGTCACAAGGAAAACTATACCCAAAA

TCCTTTTTATCCGGCATTAATTTGGAACCGCGTGCATCTAAAGCACCCTTTACTAAAATC

AATGTAGTTGTATGCAAACCTAGAGCAATAGCATGATGAACCAAGAAATCTCCAGGTCCT

ATTGTTAAGAAAAGAGAATTACTATTCTCATTAACAGCATTCAACCATCCGGGCAACCAT

AGGGTTCGACCTGCATTGAAAGCGGGGCCGCTCGTTGAAGATAAGAGTATATCGAACCCA

TATGTCGTCTTACCATGAGCAGATTGTATCCATTGGGCAAATATAGGTTCGATCAAGATT

TGCTTTTCTGGAGTACCAAAAGCAAGCATGACGTCGTTATGAACATAAAGGCCCAAGGTA

TGGAATCCTAGGAAGAGGCTAGCCCAACTTAAATGAGATATGATAGCTTCTTTATGGTCT

AACATTCTTGCCAATACATTATCCTCATTCTGTTCCGGATTGTAATCCCTAATGAAAAAA

ATAGCTCCATGAGCAAAAGCCCCTGTCATGATGAACCCTGCAATATATTGGTGATGAGTA

TATAAAGCAGCTTGAGTGGTAAAGTCTTGTGCTATGAATGCATAAGCAGGTAAAGAGTAC

ATATGTTGAGCTACTAAGGAAGTAATAACCCCTAAAGAAGCTAGAGCAAGACCTAATTGA

AAATGAATCGAATTATTGATTGTGTCGTAAAGGCCCTTATGCCCACGCCCTAATCGACCC

CCCGGAGGAGTATGTGCTTCTAAAAGATCTTTGATACTGTGCCCAATTCCGAAGTTAGTT

CGATACATATGACCGGCAATGAGAAAAATAAATGCAATAGCTAAATGATGGTGAGCAATA

TCGGTCAGCCACAAACTTTGTGTTTGTGGATGGAATCCCCCAAGAAGAGTTAGAATGGCA

GTTCCGGCTCCTTGAGCGGTACCAAATAAATGATTACTCGAATCAGGGTTTTGGGCATAA

AGATTCCACTGACCCGTCAGAAGGGGTCCCAACCCCTGGGGATAGGGTAATACATCTAAG

AAATTATTCCATCGAACGTACTCCCCCCTGGATCCGGGAATAGCGACATGAACTAAATGT

CCTGTCCAAGCCAAAGAACTTACCCCGAAAAGTCCTGACAAATGATGATTGAGACGAGAT

TCCGCGTTTTTGAACCACGAAAGGCTTGGTTTCCATTTGGGTTGTAGATGTAACCAACCC

CCTATTAAGGATAGCGTAGAAAGAAATAATAGAAACAGAGCTCCAGTATAAAGATCTCCA

TTGGTGCGTAATCCAATTGTATACCACCACTGATAAACCCCAGAATAGGCGATATTCACT

GGACCGGCAGCACCTCCTCGAGTAAAGGCTTCCACAGCGGGTTGACCAAAATGAGGATCC

CAAATCGCATGAGCAATAGGCCTTACGTGTAAAGGATCCTGTATCCATGATTCAAAATTT

CCTTGCCAAGCTACATGAAACAGATTTCCGGATGTCCATAGAAAGATTATTGCTAACTGC

CCAAAGTGAGAAGCAAAAATGTTCTGATAAAGACGTTCCTCAGTAATATCATCATGACTT

TCGAAATCATGTGCGGTAGCAATACCAAACCAAATACGACGAGTAGTGGGGTCCTGAGCT

AAGCCTTGGCTAAACCTGGGAAATCTTAATTCCATAATGCCTTTCAAATCCTCCTAGCCA

CTATCCTACTGCAATAATTCTCGCTAAGAAGAATGCCCATGTTGTGGCAATTCCACCCAG

AAGGTAATGGGTTACTCCTACAGCACGTCCTTGTATAATGCTCAAGGCTCTAGGCTGAGT

AGCAGGAGCAACTTTTAATTTGTTATGAGCCCAAACGATAGATTCAATGAGTTCTTGCCA

ATAACCACGGCCGCTGAATAAAAACATTAAACTGAAGGCCCAGACAAAATGAGCACCTAA

GAAAAAAAGACCATATGCAGATAATGAAGAACCATAAGACTGAATTACTTGGGATGCCTG

TGCCCACAAGAAATCTCGGAGCCACCCATTAATCGTAATGGAACTCTGTGCAAAGTTTCC

CCCTGTGATATGAGTTACCATCCCTTCATCGCTTATAGTACCCCAAACATCCGACTGCAT

TTTCCAACTGAAATGGAAAATGACTACCGAAATTGCATTGTACATCCAGAATAGACCTAA

GAAAACATGATCCCAGGCGGATACTTGACATGTTCCCCCTCTCCCAGGCCCATCGCAAGG

GAAGCGAAAACCAAGATTTGCTTTATCGGGTATCAAACGGGAACTGCGAGCAAATAAAAC

ACCTTTCAAAAGTATTAATACAGTCACATGGATGGTAAATGCGTGAATGTGATGGACTAA

AAAATCTGCGGTTCCTAATGGAATAGGTAACAAAGCTACTTTGCCGCCTACTGCTACTAA

CTCGCCACCTCCCCACGTTAAGCTGGTACTTGTTGTTGCACCAGGAGCTGTTACGCCAGG

CGCGTTAGCATGGATATTTTGTACCCATTGAGCAAAGATGGGTTGTAATTGTATGGCGGT

ATCCGAAAACATATCTTGGGGACGGCCTAAAGCACTCATGGTATCATTATGAATGTACAA

GCCAAAACTGTGAAAACCTAGAAATATACATACCCAGTTAAGGTGGGATATGATTGCATC

GCGGTGTCTAAGGACGCGATCTAATAGATCGTTGTATCGAGTAGTTGGATCATAGTCTCT

TACCATAAAAATGGCTGCATGTGCAGCAGCACCGACTATTAGAAATCCGCCAATCCACAT

GTGGTGTGTGAACAAGGAAAGTTGTGTACCATAGTCAGTAGCTAGGTATGGATAGGGGGG

CATAGAGTACATATGATGAGCTACAACAATGGTTGTAGAGCCTAGCATAGCTAGGTTAAG

AGATAATTGAGCGTGCCATGACGTTGTTAGGATTTCATAGAGACCCTTATGGCCTTGTCC

TGTAAATGGGCCCTTATGAGCTTCCAAAATATCTTTAAGTCCATGACCAATACCCCAGTT

GGTCCTATACATATGACCTGCGATCAGGAAAAGAATAGCAATAGCTAAATGATGGTGCGC

AATATCGCTCAACCATAGACCTCCGGTTATTGGGTCTAGTCCTCCGCGAAAACTCAGAAA

TTCTGCGTATTTGGACCAATTCAAGGTGAAAAAGGGGGTTGCTCCTTCGGCAAAACTAGG

ATAAAGTTGAGCCAAAAGGTCGCGATTCAAGATAAATTCATGAGGAAGTGGTATCTCTTT

AGGATCAACCCCAGCGTCAAGAAATTGGTTAATTGGTAAAGATACATGGATTTGGTGTCC

CGCCCAAGAAAGAGACCCAAGTCCTAATAACCCCGCTAAGTGGTGATTCAACATGGATTC

TACATCTTGGAACCAGGCCAATTTGGGAGCGGCCTTGTGATAATGGAACCAACCAGCAAA

AAGCATTAACGATGCAAAAATCAATGCACCGATTGCGGTACAATAGAGTTGTAATTCATT

AGTTATTCCAGATGCTCGCCAAAGCTGAAAAAACCCAGAGGTTATTTGGATTCCTCGGAA

ACCCCCGCCTACATCACCATTCAATATTTCTTGCCCTACTATTGGCCAAACTACCTGAGC

ACTGGGTCCAATGTGAGTAGGATCACTTAGCCATGCTTCATAATTGGAAAAACGGGCACC

ATGGAAGTACATGCCACTCAACCAAAGAAAGATAATGGAGAGTTGACCGAAATGAGCACT

AAAGACTTTTCGAGAGATCTCCTCCAAATCACCGGTATGACTATCGAAATCGTGAGCATC

AGCATGTAGGTTCCAGATCCAAGTGGTAGTATCAGGGCCCTTAGCTATTGTTCTTGAGAA

ATGGCCGGGTTTGGCCCATTCCTCAAAAGATGTTTTTACAGGATCCCTATCCACAACAAT

TTTTACTTCTGGTTCCGGCGAACGAATAATCATTAAGTCCTCCTCTTTCCGGACAAGACA

TACAAAGAGACCCGCCAACTTTTTAGTGAACCTTTGAAAGATAGATATTATGATTAGTCC

TTTTCTTTACTATCTACCGTCCTTCTATTTTTTTTAGTTATTCACTGGAGCAATTATATA

TTGAAGTCAATCCGAGGCAAGTGTTCGGATCTATTATGACATAAGGATTAGGTGCCTAAC

GGACATTGTTTATTTTGGATTTCCCGACGTACTAAAAAAACCTTTTTTAATTTACGAAGC

TAGTGTATTTTTTTAGGGTATAAGCTCCTATCTACATCTACTTTCCTTGAGTATAGATTT

TTTTATTCGATTCCAAATTCCAAGATAACTCATTAGAATTATTAATAAGACGGTCCTGAT

ATATTAGCAATATTTAGATCGCCCCCTTTATTCGCTTTATTACTTCTATTCTAGACCCTA

TCGTTTATCCTTATGAAATATAATAAAAAATAGAAGGTAGAAGAAAGGGATATAATGAAA

TTCTTGATTCGATTTACAACCAAACCCCCATTTTATGAAAAAGGAGAGTGGTCTTATTCA

AATTCAAAGCGCTTCGTAATCTTCAACCAGTTCTGTGCTTCAATATAATTTCCCGGAGTA

AGCGCTATAGCTTGTTTCCAATACTCAGCAGCTTGATCAAACCAAGCTTCCGCAATTTCC

GAATCACCCTGTAGAATGGCCTGTTCTCCTCGGTCGGAATAGGCAGTTCCTTCCCCTAGA

ACCGTACTTGAGAGTTTCCTACCTCATACGGCTCATAAATTGCTATCCTAATTTCCCCTA

TCTTAACTGAATTCGATTTCTCAAAAATCGATCCAATTTCTTCTTGGGTTAAGCAGAAGA

AGTTAATTACCTAAGTTTCAAACCCTAATTTTGATCAATAATCAGTTTGATCTTTTCTCC

CACCTTCAGAAGAATGAAGCATAGATAGACCTATAGCCTTCGTTCGAATTTTCTGAAAGG

TAACTATCTCGGTTTCTTATATGAAATTTCTATAGAATCCTTGAAAAAGACTTTTTCCAT

AAGAAAGAAAAAAGAACTTACTATCTTTGGGATCTGATACTACACCGCTGCTTATTCCTT

AGTGGATCGGCTCTATTACATAAGCAGATTCCTAAATTTTGCCCCATATCATGGGATAAG

TAAGCAGTTTTTTTTAGTTGTATCGACCCAGTCGCTCACTAATGGATCTTTACGGTGCTT

TCTCTATCAATTTGGGCTTTATCCATAGAGTAGTATAGGCCATACTTTCTTCCTATTTTG

ATTCTCGTGAAGTGTCCTTCCTTCCTACAGCTGATAGGGCAAAATCGTTGTTTTGACGAT

CCCTATGTAGAAAGCCCTTTTTCTAGTATTTACTAGAAAATTTGATCCTTTCTTTTTCTT

CTTTCTATAGTGGAGATAGTCGCACGTAATGACAGATCACGGCCATATTATTAAAAGCTT

GCGGTAAGAAGGGGTTTCGTTCTAGTGCCCGGAAATAATATTCCAAAGCCTTTGTATGCT

CTCCATTGCTTGTGTGTATAAGGCCTATATTATAGAGTATATAACTTCGATCATAGGGAT

CAATTTCTAGTCGCGTAGCTTCATAATAATTCTGCAAAGCTTCCGCATAATTTCCTTCGG

ATTGAGCCAACATCCGTTACGGTCGTTCATTCTATTCAAAAAATCTCCGTTCCAAAACCG

TACATGAGGTTTTCATCTCATACGGCTCCTCCCTTCTGTACATAGTACTAAGCGAAAAAT

CTATAGAATAAAAATAGAATTAGTCCCATCTTATTATGAACCGAAAGGGGCTGGTATTTT

TCCAAGAAATCTCTAGCCAACCTTCCCGCAAGAGGTTTTTCTTAACACCAATGAATTCTA

TTAATGCTAGAGGAAAACGATAGCTCCAAGAATTTCTTTGTTCTCAACGCCTCCTATTTA

GAGGAATTAGCCACTTCAACGATCTTTGATGGTTATAGGGGTATCCAAAGTACAAACCTG

ATGGTTGTTTGTTATCCCAACCATTCTTCCCAGCCCTGATACCGATCAGGAAAGGGCTAA

TTTCTAACAAAGTTTTTCTCTTGTTGATTCCTATTTCTAGGTGTAGTGCTTTTCTCCCCT

ATGCTGCCTATTGGTACTAGTAGAGTAGGATTGGCCTGTAATACAGAACCTATCCTGTAG

GTGTAACCTTTCGCTCAATACTCAAATCTACAATTGAAGCATCTGAGGCCGCATCAATCG

AGGATACACGACAGAAGGAATTGTTAGTTCACCTCACCTTCCCCAAGCGTGGGTTTCCTT

TACTAATTTTGTTCTCTCTATGCGAACCCCCTCTCTTTCTCGTAAGACTGAGGTGTAGGT

AGGGCTAAAAAAAAAAAGAGTCAAATCGCACCATCTCTATAATAAGTAAATGCCCTTTTT

TCCCCTGAGGTTGTCGGAATTATTCGCAATAAAATATTGGCTACAATTGAAGAGGTCTTA

TCAATGAAATTTCCATTTATACGGGATCTAGGCATAATTCCCAACCCATTCTATATAGAA

TTATTTTCATTTCTTCACAAAATAACATAAAAACAAAACATTGATTCTTATAAATCGATC

ATATGCTCTAAATGGATAAGAGAGGTATTTCCGCTCAGCTCAAATTGTCTCCTTTTCCTC

TGTTTGGACAAGAAGAGATATGAAAATTGACTAAGATTGGATTTCATTCCACTTTTCTAT

TTCTCACAATAACTTCTCTCATCAGCTATTTCGCGTTTCAAGTCATTAATTGTCTCATAC

CCTTTTTTATGGCGTAGCGTACTTATGCAAACAGAATTCTAGGGTTCCTTTTTATGGAAT

AAGAAGAATTCTTCCATCTCTTTTTCTTTGGTTTGAACCCAAACTAAAACTTTTCGAGGG

AGCGGAATTCCTAGTAAAAAAATCCTGGATCCTTCCACTTAGATGAAAAGGAATTTTTCC

AATAGAACCATGGAACCCCCGAGCCGTTGTGGTTGTACCTGTACTGCAGGAATAGGAAAA

CTCGCTATTCACTTAGTTTATTTTCCATAATAAGATTATGTAGGAGAGATGGCCGAGCGG

TTCAAGGCGTAGCATTGGAACTGCTATGTAGACTTTTGTTTACCGAGGGTTCGAATCCCT

CTCTTTCCGTTTCTCTTAATTGATCAACGTTAACGATCACAATGTATCAAATCAAATAAC

AATTTATTCCAGCAATAATACCTTTATTTAATAGAAATTTTTTATAGTAAATTACTGTGG

TATGTAAAATACACATAGAGGAAAGAACAAAGAAAAAGGATCCTAGGGTTAATCCATTTA

TGCTAGTTGAATGGAAAATACGAATTAAGGGCCTTAGGTCGATTTAGTTCGGGGGAAGGG

GAAGAAAATTCTATGAACTTTTCCTTTTTCGTTAAGTTCAAGTCTGACGAGAGTAATATT

CTACAACTAACAACTCATTTATTTTGAGACCGACCCACTTCCTATCTAGGATTTTTTTAA

CTAGTCCTTTATATTGCAATGTGTCAATCGTCAAATGCTTTGGCAATTTCCCCGGGTCGG

ATGAAGCAATAGAATTTTGAATCAGACGTTTTGATCTTTGGTTATCCTTCGTAGTAATAA

TATCTCGGGGTTTGCAACGAAAACTTGGTATATCGACTATACGACCATTAACTAAAATAT

GTCTATGGTTAACTAATTGCCGGGCCCCAGGAATGGTTGAAGCCATACCCAATCGAAAAA

GGATATTATCCAAACGCATTTCAAGTAATTGTAGTAAAACCTGACCTGTTGACCTTTTTG

CTTTTCCAGCGATATGTACATATCTAAGTAATTGTCGTTCTGTCAGACCATAATGAAAAC

GCAATTTCTGTTTTTCTTGAAGACGAATACGATATTGCTCTTTTTTCCCAGAATGGAATT

TCTTTTTCAGATTACTTCCGGATTTAGGTGTTTTTCTAGTGAGTCCTGGTAAAGCTCCCA

GACGGCGTATTTTTTTTAAACGAGGTCCTCGATAACGGGACATGAAGACTCCTTGTTTTA

TTGAAATTTCATTTTACACAATTAATTTCATTGTATTTACATTACAGAATACATCAAAAT

TAAAACTGAATTAAACTAAAGGATAAACAGAGTAAAATCTACTAAAGTACCACAAAAAAT

GGAATTTCATCAACATCTGGATTTTTGTATATATATTATTTATTTTATTGTTTTGTATCT

AGCAAAATTGTAAGGTAGAACCACATAATAGATCCTGATTCTCCATTTAATTCGGAGAAA

AAGAGAGATTCTTGTTCATGGAACATCGATATAGAAAAAAGCCGACTATCGGATTTGAAC

CGATGACCCTCGCATTACAAATGCGATGCTCTAACCTCTGAGCTAAGTGGGCTTACATAA

CAGAAATAGTGTAACAAATAGAAATATGTATATAGGAAATCCGTAAAATGTCAGATCTTA

ATTATTAATCTTAGCTATTAACTAGTTCGAAATTGGAAGTTCTACTTAGAAAAAAATACT

AGAACTTCATAAAATAAAGTTAAAGAAATTTTTGAACTTTCTTTTTTCTCTAATTTTTCT

AATAGAATCTATTCCAATTTCTATATTGAATTTGATTTCAGATATTTTCAAATGGCTCGG

ATGAGTAATCTAATACATAGAAAAGAATAATATATATGAAAGATATAATAAAGAGAAAAT

GCGAATTTCTTGCATTTTCATTCGATCATTATAGACATTTTTGAGATAATAATTTAATGA

TTAATATTTCACTAAGGAGAACATAGAATCATAGCAAATGAAATTGCTAATTCTGATTAG

AAAAAAAGAATGAATATCAAGCGTTATAGTATGATTTTGAATACTCTAAAAAAGAAAGGC

GGGGGAGAGAAAAACTTTGGGATATATTGATTCGGATTGAATTGCAAATACATCAACGAT

AGAATCAATTCAATTCTGAATTGCAATAAGCAGGGTCTGTCAAATAGAGACGAACTGCTA

GACTACGTCGAGTAATTAATTCAACGATTCAAAAAAAACTAAGAGATGGATGAAATTACA

CAAGGAATCCAGGTCTCAAGGAAAATGGGGATATGGCGAAATCGGTAGACGCTACGGACT

TGATTGTATTGAGCCTTGGTATGGAAACCTGCTAAGTGGTAACTTCCAAATTCAGAGAAA

CCCTGGAATTAAAAAAGGGCAATCTGAGCCAAATCCGTGTTTTGAAAAACAAGTGGTTCT

CGAACTAGAATCCAAAGGAAAAGGATAGGTGCAGAGACTCAATGGAAGCTGTTCTAACGA

ATCGAGTTGATAACGATTAATCACGGAACCCATATCATAATATAGGTTCTTTATTCTTTT

TTAGAATGAAATTAGGAATGATTATGAAATAGAAAATTCTGAATTTTTTTAGAATTATTG

TGAACCCATTCCAATCGAATATTGAGTAATCAAATCCTTCAATTCATAGTTTTCGAGATC

TTTTAAAAAGTGGATTAATCGGACGAGGATAAAGAGAGAGTCCCATTCTACATGTCAATA

CTGACAACAATGAAATTTCTAGTAAAAGGAAAATCCGTCGACTTTATAAGTTGTGAGGGT

TCAAGTCCCTCTATCCCCAAACCCTCTTTTATTCACTAACTATAGTATTTATCCTCTTTT

TTTTATCAATGGGTTTAAGATTCAATTGAATAGATTTCTTTTTTATTATAGTATCGGCAA

GGAATCTCGATTATTAACTCTATTTTTAAGTATTATTAAGTAAGCCATGCACAATGCATA

GGATTACCCCCCCTCATTTCCAAATTTCGAATATTGACATAGATACAAATACTCTACTAG

GATGATGCACAAGAAAAGGTCAGGATAGCTCAGTTGGTAGAGCAGAGGACTGAAAATCCT

CGTGTCACCAGTTCAAATCTGGTTCCTGGCACAGAAAAAAAGGATCTACCGAATAGGTAT

TGATACAAATACCTCGAGATGGGTTGGGATACATATTCGTTAATAATATAGATAGAGTAT

GATTTAATCTAAGTAGATAAATCTCTAAATAGAGGCACTTCTTTTTCTTCATTTTGCATT

TCTTAATTTTGTATTCCGCTATTCCGACGAATATTTATTTATTCTTGTTATCTTACTTTC

CTAGCTGTTCTAAGTAATGCGCGCGGTACAAAGTTCGTGGTAGGAACTTCTTTGAGTCAT

CATATTTTTCTGTTCATACGAAGGAAATGAATATGTGATTTTCCAACGAAATGAAGCCCT

TTTTGCTCAGTCTATCTGGACCTTTTGTATAATAGGAATTAATTAGAATATAATAGGTAT

TCCGTTTCATCTAGGAACAGAACGTAAAAATATTCCTTGACTTGAATAAAATCTGGAGTT

GTGTTGTATAAGTGAGCATGAATTTCTTATCATTCAATGAGCATCTTGTATTTCATAGAA

ATTGGGGGTTATATAGTCCTTACGTAAGGGCCAGCCTATCCAACTTTCAGGCATTAGAAT

ACGTTTAAGGCGTGGATGATTATCATAAGAGATTCCCACCATATCATAAGATTCGCGTTC

TTGAAAATCGGCACTTCTCCAAATCCAGAAGACAGACGGGATTCTAGGATTATCCTTTTG

GGCAAAGACTTTTATGCATACTTCTTCTGGGTTATCTATACCATACTGTATTCTCGTAAG

ATGATACACGCTAGCTAAAGATCCACCGGGTGCTACGTCATAAGCACATTGGGAACGTAA

ATAATTGTAACCATATACATATAAAATGACAGCAATGGAATCCCAATCCCCTGCTTTTAT

TTGTAAAGTCTCTATTCCTCGGTGATCGAAGCCCAAAGATCTATGAACCACCTCATGTTT

GACTAGCCAATTAGATAACCAACCCTGCTGCATTGTCTTGATCTCTCCCCCTTTGTATAA

ATATTTCACATTTCGAATGCAAGTTTGAAAGATTGCCCTGCTCTTTCTTTTTCTACACAA

AGAGCCCCTCCTAATTCACTAATTTTGAGGAAGATACTGGACTTTTGGATTTGAAAAAAG

TTTCAGAAGATATGTCTAAAGTAGATGGTGATTGATAGAGCAATTCTTGCTCGTAAGTTC

CAGTATAAGTACTGCGCCTAACATAAAGCTTGTGACTGGTAGTAAAACATCGATTTTTCT

TTTGAGATAGAGTTCGATCCTCAACTATTTCTCGCGATATCTTCTTACGAAGTTTTGTTA

GGGCATCTATAACTGCCTCCGGTTTAGGTGGGCAGCCCGGCAAGTAGACATCCACAGGAA

TTAACTTATCAACTCCCCGAACAGTACTATAGGAATCCGTACTGAACATTCCCCCTGTAA

TAGTACAGGCTCCCATAGCAATGACGTATTTTGGTTCAGGCATTTGCTCGTATAATCTCA

CTAAAGAGGGAGCCATTTTCATTGTTACCGTACCAGCTGTTAAAATTAGGTCTGCTTGCC

TAGGACTTGATCTTGGTACCAATCCATAACGATCAAAGTCGAATCGTGAGCCTATTAATG

AAGCAAATTCAATGAAACAACAACTGGTACCATATAGAAGGGGCCATAAACTGGAGAGTC

TTGACCAATTCGAAAGATCATTTGGTGTAGTTGAAATAACGGAATTGGAACTTGTTTGGT

CAAGTAAGGGAAACTCAATCAAACTCATAACTGTCTCAATGGAATCTTTTCCTTCTTTTT

TTTTTTTGTCTGAATATTCAGTTAAGACCATTCCAAGGCTCCTTTTCGCCATGCATAAAC

TGAACCAACAACTGGGATAAGCACGAAAATGAAAGCTTCGATAAAAACGGATACACCCAA

TACGTCGAAACTCATTGCCCAAGGGTAGAGAAAGACCGTTTCCACATCAAAAACAACAAA

AACTAGCGCAAACATGTAATAGCGTATTCGGAATTGTAACCAAGCCCCTCCCATGGGTTC

TATACCCGATTCATAACTAGAAAGCTTCTCTGGTCCTTCACTAATCGGGGCTAAAAGTCC

TGAAATCCAAAATGCAAAAATAGGAATAAGGCTTGCTATTATTAGAAATGTCCAAAAAAT

ATCATATTCGTGAAGCAGAAACATAAATGTACTCCCATTAATGTGGAATAGGCGGAACTG

AATTAGTCAATTCAAGTCAGCGTTGTCAATTTATCCAGAACTTCTCTCTTTTCCTCGGTG

AAACAAGAATCCGTTTTGCTCAAATCAAAGCACTTAGTTTAGCCTTTGTTTCCTCTGTGC

CCTGTCTTCTTTAAAGATTCATCCAATGGAATCCCGACTCCCTTTTTGATTTCCATTCTA

TTTATAATTAGAACCTAATTAAGATAGGATGACTAATGTATGCAGCCTAATGAGGAGTAA

TACAAAAATAAAGAACTCTATTTCAGAAAGTAGATCAATTTAGATAATGTATATAAAAGT

AATATACTTCAAACAAAGTAGGAATTCGCAAGATGGAGAAAATCTTGCAGTTGATTTGAT

AGAAATTCGTTTTTCTTTTCCTGTCTCTAAGATTTTCGATGAATGAGCCTGTGGTAATGC

TTTTATCTCTATTCTATGGCGCAAGCGACCGTCCAGTCTATAAACAAGTACTAATGAGGA

AATGAAAACTATACTAAAGGAAACATAGGATCTCTATCCTAAAATCTAAAAAAGGACATA

TTAGGGCTATACGGATTCGAACCGTAGACCTTCTCGGTAAAACAGATCAAACGGATATTA

TCGAAATGATTCGAACTGTTTCAAAGACCCAACATGCATTTTTTGCATTGGGCTCTTTCA

TCAACTGATGTAAAGATCAGTTAGTCCACCATAGTTTTTCTTTACGGAAAGATAATGAGA

TGGCTCCCTGTGCTCTGATTGATTATTTGTATTATGATCTATCTAGGAGCAATACCAAAG

TGTTTCAAAGGAGGATTACCTTGACTTAGGTCGCCTCCGGCCTAAATTAAATCAACCTAA

GTGAAATGGAGTCTCTATCGTTCCGCTGCAAGAGTTGACTATGAGACTTCATACACCTTA

AAGTTCATAGAACGAAAAGAAGTTTTTTGGAGGCCCTTATCCTCATTACGCCTAGCATTT

AGTGGGCTGGATATTTACCTTATCAACTAGCAAATCAATAAGGGTTCTATTTGATTAGGC

ACCTGAATTGGCACCTGAATCGGACTGAACCGACTGTTTGTCAGGCTACTGTTCTCCTAT

TCTCTCGAATCTATGAAGTAAGACATTGATTTTGCAATAAGATCAATTATGTTCATTGCA

TAATAAGCTCCCTTGAAAAGCATTGGCGCACGTGTAAGCGAGTTGCTCTACCGAACTGAG

CTATAGCCCTTGTCAGAGATATCTTAACATATAGATAATTTCTTGTCAAGATGAATATTC

TCTAATGCCAGAGGATATCCTTTGATCTGTTTACTATATAACATACCAATAACGGAGCAG

TATTGCTTATAAAAAGGATTCGATCTATAATCGATCGAAGTAATGGGTCTTCCTTTGTGG

TGATAAATTGCCTACTTAACTCAGTGGTTAGAGTATTGCTTTCATACGGCGGGAGTCATT

GGTTCAAATCCAATAGTAGGTAGGTAGGTAGAAAAATTACTAGATAGCATTGGACTTACT

TCGCTTCGCTATCTAATAACTTTTTCTACCCCTCTTCCCTTTTTCTTTGTATCAACTAAA

CCATTGGATTGTATTCAATTGGATGGGGGAATCCAATTGATAGCCTCGACTCGTATCCTA

GCTCGTCTGAGAGCTAGCTTCGCTTCAACCAACTCTTTCGTACCCTCAGCTCTACTCAAG

TTAGCTTCGGCTATTTCAAGTGCCTGTTGAGCTTCTTCCGGATCAATGTCACTACCCAGT

TCCGCATCATTTCCTAAAATGATGATCTCATTATTAACTATTCTCGCAAAACCGCTCCAC

AGAACCGCCGTTAACCATTGGTCGTTGAGGAGGCGTATTCTCAAAGGACCCATATCTACA

GCTGTGTTAATGGGGGCGTGGTTTGGTAATACGCCAATTTGGCCACTATTAGTAGATAAA

ATGATTTCTTTCACTTCACAATCCCAAATAATTCGCTTAGGAGTTAGTACATAAAGATTT

AATTTCATTTCTTCAATTTGTTCTCCTCTTCTAAGTTTATAGCTTTCGTGCTAGCTTCAT

CGATGTTACCCACCAAATAAAAAGCTTGTTCGGGTAGGCCGTCTAATTCTCCGGAAAGGA

TTAGTTGAAATCCCCTAATTGTTTCTGCAAGACCAACATACTTTCCTGCAGAACCGGTAA

AAACTTCTGCCACAAAGAACGGTTGTGATAAGAAACGTTCAATTTTTCGTGCTCTTGCTA

CAGTTAAACGATCCTCCTCTGATAATTCATCCAACCCAAGAATTGCGATAATGTCCTGAA

GTTCTTTGTAACGTTGTAAAGTTTCCTTAACTCTTTGCGCAGTTTCATAATGTTCGTTGC

CAACGATCCGAGGCTGTAACATAGTTGAGGTTGAATCTAAAGGATCTACTGCTGGATAAA

TACCCTTGGAAGCTAATCCTCTGGAAAGTACGGTAGTAGCATCCAAATGTGCAAATGTTG

TGGCAGGAGCAGGGTCGGTCAAATCGTCCGCAGGTACATAAACTGCTTGGATCGAAGTTA

TGGATCCCTTTTTTGTAGAAGCAATTCTTTCTTGCAAAGAACCCATTTCTGTACTAAGAG

TAGGTTGATAACCCACTGCGGAGGGCATTCTCCCTAATAAGGCGGATACCTCCGATCCTG

CTTGAACAAAACGAAAGATATTATCGATGAATAGAAGCACGTCTTGCTTATTAACATCTC

GGAAATATTCTGCCATAGTTAGGGCAGTTAAACCAACTCTCATACGAGCTCCCGGCGGTT

CATTCATTTGACCATAGACTAGAGCTACCTTTGATTCCTCCAAATTTTTTTCATTAATTA

CTCCGGATTCCTTCATTTCCATATAAAGATCATTTCCTTCACGAGTCCGTTCCCCTACTC

CGCCAAATACGGATACGCCTCCATGAGCTTTAGCAATGTTGTTGATTAATTCCATGATGA

GTACTGTTTTACCTACTCCAGCCCCCCCAAATAGTCCTATTTTTCCTCCACGTCGATAAG

GAGCTAAAAGATCGACCACCTTAATACCTGTTTCAAAGATGGATAATTTCGTATCTAGCT

CGATAAAGGCAGGCGCAGATCTATGAATAGGGAATGTTGCATTAATATCTACAGGACCCA

AATTGTCAATAGGTTCCCCAAGAACGTTGAAAATTCGTCCGAGAGTAGCTCCACCGACTG

GAACACTGAGAGGAGCTCCCGTGTCAATCACTTCCAATCCTCTCATCAACCCGTCTGTAG

CACTCATAGCTACAGCTCTAACTCGATTATTTCCTAATAATTGTTGTACCTCACAAGTCA

CATTAATTTGCTTACCGGCAGTGTCTCTACTCTTGACTACCAAAGCGTTATAAATATAAG

GTAACTTGCCCGGGGGAAAAGTGATATCCAGCACGGGTCCAATAATTTGATCGATACGCC

CTATGCTTTTTTCTTCAATTGTGGAAACCCCGGGACGAGAAGTAGTAGGATTGGTTCTCA

TAATTATCACATAATTTTCAAAAAAAAGGAATTTGTCGAATTTTTTCTTGTTGAATAATG

CCAAATCAAAAAAAATAGCCAAAAATCCAAAAGTCAAAAGGAAATGAATTAGTTAATTCA

ATAAGAGAGAAAGGGGGACGAGGACTTGATTTCGTTGCCCAAGCGAATCCCATTCAATCG

TTTACTCATGGAATGAGTCCGTTGGAAAGTTCAATCAATTTTTTTCATATACATTTCGCC

TTTTGTGGAGGATCTGTCCCTACTCTACTTTCCTATCTAGGACTTCGATATACAAAATAT

ATACTACTGTGAAGCATAGATTGCTGTCAACAGAGAATTTTCTTAGTATTTAGGTATTTA

CATTCAAAATAAGAAAGGGGCCTATTAAGAACTTAATAAGGATTAGGGATTGGGTTGCGC

TATATCTATCAAAGAGTATACAATAATGATGGATTTGGTGAATCAAATCATGGTTTAATA

ACGAACCATGTTAACTTACCATAACAACAACTCAATTCCTATCGAATTCCTATAGTAGAA

TTCCTATAGGATAGAACATACACAGGGTGTACGCATATATGAATGAAACATATTCATTAA

CTTAAGCATGCCCTCCATTTTCTTTAATGAGTTGATATTAATTGAATACCCTTTTTTTAG

ATTTTTGCAAAGGTTTCTTTCGCCTAATCCTATCGAGTAGACCCTGTCGTTGTGAGAATT

CTTAATTCATGAGTTGTAGGGAGGGACTTATGTCACCACAAACAGAAACTAAAGCAAGTG

TTGGATTTAAAGCTGGTGTTAAGGATTATAAATTGACTTATTACACCCCGGAGTATGAAA

CCAAGGATACTGATATCTTGGCAGCATTCCGAGTAACTCCTCAGCCCGGGGTTCCGCCCG

AAGAAGCAGGGGCTGCAGTAGCTGCCGAATCTTCTACTGGTACATGGACAACTGTTTGGA

CTGATGGACTTACCAGTCTTGATCGTTACAAAGGGCGATGCTATCACATCGAGCCCGTTG

TTGGGGAGGAAAATCAATTTATCGCTTATGTAGCTTATCCATTAGACCTATTTGAAGAGG

GTTCTGTTACTAACATGTTTACTTCCATTGTGGGTAACGTATTTGGTTTCAAAGCCCTAC

GCGCTCTACGTCTGGAGGATCTGCGAATTCCCCCTACTTATTCAAAAACTTTCCAAGGTC

CGCCTCATGGTATCCAAGTTGAAAGGGATAAGTTGAACAAGTACGGCCGTCCTTTTTTGG

GATGTACTATTAAACCAAAATTGGGATTATCCGCAAAAAATTACGGTAGAGCGTGTTATG

AGTGTCTACGCGGTGGACTTGATTTTACCAAAGATGATGAAAACGTAAACTCACAACCAT

TTATGCGCTGGAGGGACCGTTTTGTCTTTTGTGCCGAAGCAATTTATAAATCACAGGCCG

AAACCGGTGAAATCAAGGGGCATTACTTGAATGCGACTGCAGGTACATGCGAAGAAATGA

TGAAGAGAGCTATATTTGCGAGGGAATTAGGGGTTCCTATTGTAATGCATGACTACTTAA

CTGGGGGATTCACCGCAAATACTACTTTGGCTCATTATTGCCGCGACAATGGCCTACTTC

TTCACATTCACCGGGCAATGCATGCAGTTATTGATAGACAGAAAAATCATGGTATGCATT

TTCGTGTATTAGCTAAAGCATTGCGTATGTCTGGGGGAGATCATGTCCACGCCGGTACAG

TAGTAGGTAAGTTAGAAGGGGAACGCGAAATGACTTTAGGTTTTGTTGATTTATTGCGCG

ATGATTTTATTGAAAAAGATCGTGCTCGCGGTATCTTTTTCACTCAGGACTGGGTATCTA

TGCCAGGTGTTATACCGGTGGCTTCAGGGGGTATTCATGTTTGGCATATGCCAGCTCTGA

CCGAAATCTTTGGAGATGATTCCGTATTACAATTTGGTGGAGGAACTTTAGGACATCCTT

GGGGAAATGCACCTGGTGCAGTAGCTAATCGGGTGGCTTTAGAAGCTTGTGTACAAGCTC

GTAACGAAGGGCGCGATCTTGCTCGTGAAGGTAATGAAATTATCCGAGCAGCTTGCAAAT

GGAGTCCTGAACTAGCCGCAGCTTGTGAAATATGGAAGGCGATCAAATTTGAGTTCGAGC

CGGTAGATACTATAGAAAATAAAAAAGAAGCGAAATAGAAAGAGAAAAAAGCAGTTACGA

AATGCAGTAATTCTTCTTTATTCTTCTAATTGATTGCAATTAAACTCGGCTCAATCTTAA

GATTGAGCCGAATAAAAATAGATCTTGATACGATCATGAGACTTGACAAATCGAGATTCC

TCTATTCTATATATTTAGAATATATAAAGGTATAATACAATAAATAAATACAAATATAGT

ATTTATTTACTTTATTGGGAAAGAATCAATGAAAAAAGATTAGGAATCGATAATATTCTT

CTATAATCCCGGGGTCTTACTAACAGGACTTCGCTGCACGGGAAGGTAAGAGAATTTGCC

CTATTTTGGAAATTACAGCTTAATAAAAAAGTCTAAGCACTATCAATCAACTGTCGTACT

TTGAATCCAATTTCAAGTTCGATTAGAAGGATAGAAAGGCCATGAGGACGGGAAAAGAAA

AATCAAATCTTTTTAATCTCCTTTTTTGCAATTTTCTTATTATCCATTCCATTCATTTTT

TTTATAGAATACTATCTATAAAAAATCTTTTTTTGCAAACTAAAAAATACAATAGTCAAT

ATTCCTTATAATAGATATACTTAATTATATTATAAGAATCCTAAGATATTTTTCGAATAG

ATAGAAATAGTAAATTTGAATTGAGACACCTATTCTATGACGGATTTTAACTTACCTTCT

ATTTTCGTGCCTTTAGTAGGCTTAGTATTTCCGGCAATTGCAATGGCTTCTTTATTTCTT

TATGTGCAGAAAAATAAGATTGTCTAGAACCGATGGGGCCGAATTTTCTCAATGTATTTC

CAGGATCATAATACAGATATTTTTTAGTGTAAGTAATATGGTAGGGTATGTGGCTCTTTC

TACACACAAATGAAAAACGGCTATGGATGCGGATATAGGCTACGAGCATAAATGCATGCA

TATGCGGAGCCGGGTATAGCAAGTTTTTTTAAGTAGATCAACAGATATTTTTTGAATAGA

AAGTCAATGTATCTAACCAATTATTTCACAGGAGTACTAGTTACTGAAGGCGATTTCAGA

ATCAAAAAAAGTAAAGTCAAAATCATTTAGCTTATTCTCTCAATTTCAATCGACCGCTGT

TAGTATATCTAATATGAATTGGCGATCAGAACACATATGGATAGAACTTCTAAAAGGTTC

TCGAAAAAGAGGTAATTTTTTCTGGGCCTGTATTCTTTTTCTAGGTTCACTAGGATTTTT

ATCGGTTGGGGCTTCCAGTTATCTTGGTAAGAATATGATATCTGTACTTCCATCTCAACA

AATTCTTTTTTTTCCACAGGGGGTCGTGATGTCTTTCTACGGAATCGCGGGCCTATTCAT

TAGCTCCTACTTGTGGTGCACTATTTTGTGGAATGTAGGCAGTGGTTATGACCGATTCGA

TAGAAAAGAGGGAATAGTGTGCATTTTTCGTTGGGGATTCCCTGGAATAAAACGTCGCGT

CTTCCTTCGATTCCTTATGCGGGATATCCAATCAATTAGAATTCAGGTTAAAGAGGGTCT

TTATCCTCGTCGTATCCTTTATATGGAAATCCGGGGCCAGGGGGTCATTCCCTTGACTCG

TACTGATGAGAAGTTTTTTACTCCACGAGAAATTGAACAAAAAGCTGCCGAATTGGCCTA

TTTCTTGCGCGTACCAATTGAAGTATTTTGAATACCGATTTAATTTTTTGAAATTGTAAG

GAGATTTTGAGTATTTATCTAAAGAAAGGAACAAACGAGGATAAGAGAAAATTGCTTCTA

ATTTGTCCAAGTGATGGCATAATATTCTTCCATTTTCATCTGAAAGGGCTTTTTCTATTT

CTCTATTCCACTCCATCTAGATCTAAGAAAGAACCCAATGCAATGAAATTCCACTAGTAT

ACAAAAAAGAGGAATAGATACAAGGTCTCAAACCTTGTTATAGAATTTTTGCTTCAAATA

AAAGAAATATCATATATATTCATTTACAGATCAAAATGAAAAAAAAGAAAGCATTGCCTT

CTTTCCTATATCTTGTATTTATCGTACTTTTGCCCTGGGGAGTCTCTTTCTCTTTTAACA

AATGTCTGGAACTTTGGATTAAGAATTGGTGGAATACCAGGCAATCTGAAACTCTCTTAA

CTGATATTCAAGAGAAAAAGGTTCTAGAAAGATTCATAGAATTAGAAGAACTTTTTCTCT

TGGACGAAATGATAAAAGAGAAACCGAAGACACATGTACAAAAACCTCCTATAGGAATAC

ACAAGGAAATAATACAATTGGTCAAAATAGATAATGAGGATCATCTCCATATCATTTTGC

ATTTCTCGACAAATATAATCTGTTTGGCTATTCTAAGTGGTTCTTTTTTTCTGGGTAAAG

AGGAACTTGTCATTTTGAATTCTTGGGTTCAGGAATTCTTCTATAACTTAAATGACTCAA

TAAAAGCTTTTTTGATTCTTTTAGTTACTGATTTTTTTGTTGGATTTCACTCCACCCGCG

GTTGGGAACTACTAATTCGTTGGGTCTACAACGATCTTGGATGGGCTCCTAATGAGCTAA

TTTTCACTATTTTTGTTTGTAGTTTTCCAGTGATTCTAGATACATGTTTTAAATTTTGGA

TCTTTTTTTCTTTAAACCGTCTATCTCCTTCGCTTGTAGTCATTTATCATTCAATTAGTG

AAGCATAAACTCATTCGATTTCCTGATATTAATCAAATTAGCATCTTTCTTCCTTTAGAA

AGAAAGCCCTTTTCCATTTTAGCAAAATTCTTTCTATTTCTACCTGCTCAAGGTATTCAT

CATTCCAGTACAACTGTTGCAGTAGAATGACAACAGACTCGTGTATAGGGAACTAGATTA

GCTTAGCTACCTATCTAATTTATTGTAGAAATTCTGGGATCTGCGATTGGATATGGAAAA

TAGAAATACTTTTTCTTGGGTAAAGGAACAGATGATTCGATCGATTTCTGTATCGATCAT

GATATACGTAATAACTCGGACATCTATTTCAAATGCATATCCCATTTTTGCGCAGCAGGG

TTATGAAAACCCACGAGAAGCAACCGGACGAATTGTATGTGCCAATTGCCATTTAGCTAA

TAAGCCCGTGGATATTGAAGTTCCCCAAACTGTGCTTCCCGATACTGTATTTGAAGCAGT

TCTTCGAATTCCTTATGATATGCAACTGAAACAAGTTCTTGGTAATGGGAAAAAGGGAGG

GTTAAATGTGGGTGCTGTTCTTATTTTGCCCGAGGGATTCGAATTAGCGCCGCCCGACCG

TATTTCTCCTGAGTTGAAAGAAAAGATAGGAAATCTTTCTTTTCAGAGTTATCGTCCCGA

TAAAAAAAATATTCTTGTGATAGGCCCTGTTCCCGGTAAGAAATATAGTGAAATCGTCTT

TCCCATTCTTTCCCCCGATCCTGCTACGAAGAAAGACGTTCATTTCTTAAAATATCCCAT

ATATGTAGGGGGAAACCGAGGAAGGGGACAGATCTATCCTGATGGTAGCAAGAGTAACAA

TACGGTCTATAATGCTACGTCAACAGGTATAGTAAGAAAAATACTACGTAAAGAAAAGGG

GGGATATGAAATATCCATAGTCGATGCATCGGATGGACGCCAAGTGATTGATATTATACC

TTCCGGGCCAGAACTTCTTGTTTCAGAAGGGGAATCGATCAAGCTTGATCAACCATTAAC

AAGCAATCCTAATGTGGGAGGGTTTGGTCAGGGGGATGCAGAAATCGTGCTTCAGGATCC

ATTACGCGTCCAAGGCCTTTTGTTCTTCTTCGCATCTGTTATTTTGGCACAAGTTTTTTT

GGTTCTCAAAAAGAAACAGTTTGAAAAGGTTCAATTGTACGAAATGAATTTCTAGGTCCC

GGCTTACCATCAAGTTGGTAAAAAGCCGCGATGCCTGGAATTCCTTATTTCTATCTCATG

CAAAAGAAGAGAATTCAAGGCAAAGGCGAGAACAATAAAAGGATTTCCTCCTTTTAGGAG

GGATTGCTGGTCTTCTTAATCCTCTATTGGGCACAAGAAAAAGGCTTTTTTGCCTTTTTC

TTGTGTCGATTCTTCTTGTATCAAATCAGAATCATTTTTCTTCCTATTCGTCAAAGATTA

CTATTTCTTCTTTATTCGGGTCTGTCTCTTGGACCTCTTTTGCTTAGGTTCAGGCGAGGT

AGTGGACAAACGGAAAGAAAATATGGCGGGGACAAATTTCTTGTGAGCAAATAGAATTGC

TTGACTTGTTCAATTAAGTTCAACTTCGAACTTACAGAATTTTTGCAAAAAAACGTATAC

TCTTCTATTGAAGGGTGGTTTTTCTTTTTAGGCTAGTTGAGTAGTTTTGATTAAGGTTTT

ATTAGTTTATTACTCTAAACTAAATCAATGATTTGCAGGATACTTCTTCCGTGGAATAAA

ATATTGGATCCTCCCCCTTCTTTCTTGTTGCTTCATAAGAGTGAATCAATTTCATGGGCG

AAGGGATAAATCAACCGATGGATTGCTTCACTAACACCATTAACAAACAAAAGAATAAAT

AGAGGGATTCTGACCATCAGAGCAAAGGTTTCTCTTTGTTATTTTTACAAATCGAAATAG

GTAACCCATTTGTAGGTTATGGAATAAAGTTATATTATAAGAGTAAGAATTCCGCGGGTC

CTTTCCGCTCTAATCAGATAAAAGGGGGTAAGGACCCGCTAAGCTCCTACTTTTTCATGT

TTACAATCTGGTCCCTCCGATTACTATAGAGATGAACCCAATCCAGAATATGAACCGTAA

AAGAAAACACCTATTAAACCAATCACAGGAATACCAGTTACAGTACCTATCAGCCAAAGA

GGAATTCTTCCAGTAGTATCGGCCATTTCCCCTACTTTCCTCCACATTTTATCAAGTGGT

CATGCTAGAGACAAAAACAGTCATGGATAGTTATAAGGATGGTATCCTTCCAAATGGGAT

AAGAGAATTCTTACTACTCTCTTTCTTTCTCTCAATTGAAGAAGTAATTGGAAAATAAAA

CAGCAAGTACAAAAATGAGTAATAAACCCCAGTATAGACTGGTACGATTCAATTCAACAT

TTTGTTCATTCGGGTTTGATTGTGTCATAGTTCTATAGTTGGAATTTGGTTTATCGTTGG

ATGAACTGCATTGCTGATATTGATCCCAAGAAAAAAACAGTAGGTACGGCTAGTCCGTGA

ACAGCCAGCCATCGGACTGTAAAAATAGGATAGGTTCGATCTATGGTCATTGAGGGCCTC

CTAAAAGGATCTACTAAATTCATCGAGTTGTTCTAAAGAATCAAAACGGTCGGTTATTAA

GGGAATTCCTTGTCGGCTTTCCGTGAAATACTCGTTTGGCCGAGGACTTCCAAACACGTC

ATAAGCTAAACCCGTACTGACAAATAACCAACCCGCAATGAATAGGGAAGGTATAGTAAT

GCTATGAATAACCCAGTATCGAATACTGGTAATAATATCAGCAAAAGAACGTTCTCCCGT

GCTTCCAGACATGCTGAGCTCCCCAAATTTTTATTCAAAAAAGGAATTGATTCCGTAAAA

GATGGGATCAACCAGTAAATAGAAAATTACTGATATTTCATCCTTGTGAGATTGTCAATT

TTGTACCAAAGGTGTATTTTGAGTATACCGAATTAGTATAGCTATCCTTCCTATGGCACA

GCAATCTAGTTTTGCTTGGTCCCGAAACATAATTCTTTTTTTCTCTTCTTTGTTCCTTGT

CTATAGGTAAGCTATATGTTATTCAAGGCATCAATAGAAAACCCCATTTTTGGGGTCCTG

CTTATTTTCATTGGCTTCGGATTAGTAGAATAATTCGGAATAGCGGCCAAGATCTTGGGA

AAATCCAAGTTAATGATCAATAGGATAAATAATTTAGGAAAGATATTCTCATACTGACAC

AATATAAGGACAAGTATATGCGAAATCTATCCCTTAAGGAATTTAATTGGTTAGCATAAT

ATCTAATAAATAGAAAATCGAATAGTGGATAATCTGTTATGAGAGAAAGAAAACATTCTT

TGAAGAATCAAGATTCGTAATCAATACTTGCCTTGTTTACTAACTTTCTTGACTAAACTG

CAAGCGTGGAACTCAGGAAACTTCGAATCAACTTTGGTTGGGGTTCAAAAAGGAATAAAA

ATAAAGTAAATTCAAGGAAGATTTCCTTTTTTTAAGGGGCCCTCGGGGGTCGTGGAATGC

TTTTCTTCTCCTCTTATTCCATATGGAATACAATCAGTTAAAATAAGAAGGAATAGGGAA

TATTCGACTGTTTCAATTCTTTATTTATCTTATATTCCAAAATTCTCCCAAAATCCAATT

TAATTTTTCAATGGGGTTAGATGATCTAGTTCTTAATATTATTACTTTAAAGAACTGACA

GATTCCACAACAAATCTCTTGATTCGGAATTAGAGACTCATGTCCCATCTGATGAATCGA

TTTTCTTTTACACTTCTGTATCTCACTCTATCTTGTTTTTTAGTATTATCTAAAATAACC

GATGAATTATGAATTTTCCATAACTTAGGTAAGTGCTTTACCAACATATGTAGTGTAGTA

AAAAAAATGGAATTTAACCCTTTCATGCTTACTATAACTAGTTATTTCGGTTTTCTACTG

GCTGCTTTAACTATAACCCCAGCTCTATTTATTGGCTTGAACAAGATACGTCTTATTTGA

AATGAATTGAAAATCTTTCTTTTGGATTCCTGGTATTCTAGCACTAATTATCAATTCTTT

TCTTGGTCATTGAGATTCGTGGATAATTTAGACTACTATTTAGGGATAGATCGTACCTCT

TTTTTTATCCCCTCGAACAAATCGAAATGATTGAAGTTTTTCTATTTGGAATCGTCTTAG

GCCTAATTCCTATTACTTTAGCGGGATTATTCGTGACTGCGTATTTGCAATACAGACGTG

GGGATCAGTTGGATCTTTGATTGAGTAATATTTCTTTTTTGATTGACCTCCTCCAGACCA

GAGAGGAGGTCAAATTGGAGTTGCAATTTGTTTTGTTAAGTTATTTTAGCTTCGACATAA

GATAGATGGAATCACGCTCTGTAGGATTTGAACCTACGACATCGGGTTTTGGAGACCCGC

GTTCTACCGAACTGAACTAAGAGCGCTTTCAAAAAGAAAACCCTTTTCTACTCCTAACGT

GTCTCACGTACGTATAGTATCCACAAATTCAAGTTATACCCACTTTAATTGATCTCCTCG

CTACTGCCCATAAAGAAGAAAGAAGTAATAGGTAGGGATGACAGGATTTGAACCTGTGAC

ATTTTGTACCCAAAACAAACGCGCTACCAAGCTGCGCTACATCCCTTTTCCAAATTGTTG

TATAATGGCATTGTACACAATTCCTGTCTTGTTTTCCACATCGTAATTTTCTTCTCTTTC

TCTATCTATATAGAACCTTCTTGTCATTTCTTCTTTTTGGTCTCATATAATCAAGGAATG

GTATACATCTAAATCCTATCTAATTTCACCTATAAAAGAAAGATTACTATTCCTTGGTAA

TGTATAGGAAGGGGTCTTTTTAGTTAGGAATTTCGCCTAAACAAAAGAAATACAAATGAT

CTTGGGCAAGAGTATCTGATCATATATGTATTCCAATAAGGAAGGAGGATTTTCAATGCG

GGATATAAAAACATATCTCTCTGTAGCGCCCGTGCTAAGTACTCTATGGTTTGGGGCTTT

AGCAGGTTTATTGATAGAAATTAATCGTTTATTCCCAGATGCTTTGTCATTCCCTTTTTT

TTAATTCTAGTTATTGCTATGCGAGGAATTCTTCGTGACATGACGCAAATTTTCCCTTTT

TCAATCTTTTTTATAGGAAGGAAAAAGAAAGAAAAGATGGATAAGGATTGTATTCTTTAA

TTATTTCTCTATTTTTTATTACTTAATTTAAGAATTTTAAAAATTTTTTATTCGAATGGA

TTTTATTCTTCTTCTTGGGATTCAAAATAGAAGAATAACAGAATAAGTAGAAGAATTAAG

TTAAGTCAATCCAAAAAAGAAAGGAGGTTCATGGCCAAGGGGAAAGGTGTTAGAATCAGA

GTTATTTTGGAATGTATCAGTTGTGTTCGAAAAGGTGCCAATGAGGAATCGACGGGGATT

TCTAGATATAGTACTCAAAAGAATCGCCACAATACACCCGGACAATTAGAATTAAAAAAA

TTTTGTCGTTATTGTCGCAAGCATACGACTCATGACGAAATAAAAAAATAGGAGCATCGT

GTGTTCGATCTTTCCAAAGAACAGATTTAATATATAGAACATAGATAGAATACAAAATAC

AAATCAATTCATTTGATTTCAGGTAGATATTATTTCATATGTATAGAGGGTATTCATATA

CTATATGGACCAAAGAAAGACTACTTCTTCTGGATCCAAAATTAATAAAATAAAGAAATC

CATTTTTTATTTTAAAATAAAGAATAAATCATGTATACATCTAAACAACCTTTTCATAAA

TCTAAGCAAACTTTTCATAAATCCAAGCAAACTTTTCATAAATCCAAGCAAACTTTTCGT

AAATCCAAACAACCTTTTCGTAGGCGTCCTCGGATTGGCCCGGGGGATCGAATTGATTAT

AGAAACATGAGTTTAATTAATCGATTTATTAGTGAACAAGGAAAAATATTATCGAGACGA

ATAAATAGATTAACCTTGAAACAACAACGATTAATTACTCTTGCTATAAAACAGGCTCGT

ATTTTATCTTTCTTACCATTTCGTAACTATGAGAATGAGAAGCAATTTCAAGCCCAGTCA

ATTTCAATAATTACTGGTCCTAGACCCAGAAAGAATAGACATATTCCTCAATTAACGCAA

AAGTTCAATTCCAATCGAAACTTAAGAAACTCCAACCAGAATTTAAGAAACAACAATCGG

AACTTAAGTTCCGATTGTTGATGTTTTATTCGAAAGGGCCAGACCTATATAAAGAAAGTA

ATCCAGTTTTGATTCTTGTGTTTGTTATAAGAAAGAAAAATGGGGAAGAATAAATAGTTT

TTTTATTTATTGCAACATGCTCGTTGATTCCTACCACTTAATCTTAATTTATTGTATCTT

CCCGGAGTTCCCTCTCCGGGAATTCGGTTTTAATTATTCCTGTATATTACTTTTTTATCC

TTTAATTGAGGATCTTTATTTTATTGGAAATCGTGTAAAGATTATTTGGATTTGATACAG

CTACTTGTGCAAGCATTTTACGATTAAGAATCAATTCCTTCTTGTACAGATTGTGTATTA

ATTTACTATAACTATCGAATACTTTATATATCCGCGTTGCTGCGTTTATCCGAGTGATCC

ACAAACGACGAAAATCCCTCTTTTGCCTGCCTCTATCTCGATGAGAGGAAACAAAAGCTC

TTCTTACTTGTTGAGTAATCATTCGATTAAGTCTTAAATGAGCCCCTCTAAAGTTTGAGG

CAAATGAACGCATTTTTGTTCGTCGTCTCCGAGCTATATATCCTCGCGGAACTCTGGTCA

TTGAATCAAATTAACCTTAATGAATAACTAATGATTTCTCTTCTTTTAGCCATCCTTTTT

CCCATTAATAACAAAACGAATTATTCCGATATATAAAATATTAATTCCAATGGCTTTTGC

TACTGTAACCTTCCCAACCACGATTTTTTATTCTATTCAGTTATTTCGCACGATACTAAA

AAATAGTGGGTTCCATCGTTTCTATGGTTCCCTTTTAAACGGTGAGGCCCTCTCTATACA

CCGGAGCCCTTTCTTTCATCAAAAGGTATTGTGAACTTGTATAGTTCACATTCTTTGGCT

CTACCTATCCATTATAGAGTAAATAGCTCTTTTCACAATAAGAGTTATCCATACAGTGAC

GGCATTTAATTATGAAAGTTGCTAAGTAGCTGACCCTGTTAGTCCGTTCTTTTAAGATAA

AGGAGCATAAGCCTTTTTCTTTTTATTACTATTTCCTCCGCTTAATGGATACCATTTTCT

ACCAATGGGGAATTGCTTCTTCCAATTTAGATGATTGGATTTGCACCAAAGGAAACCAGA

AATTCCATATACCATAGAATAGGATAGAGAAGCTCTATCCTATTCATTGGTACCGATCAT

GGATACTTCAAAAATTGTCTTATTTGTTTGAACTCATGATCTGAACGAGTCGCACATACA

CCCTAGCACATGTTCCTCGACGCTGAGGACATCCCTTAAGCGCGGGCGATTTTCTAGCAT

TTCGTATTGGCTGTCTTGCGTTTCTAATAAGTTGTTTAACCGTTGGCATGTCGTATGTAT

ATAGAAAAAGGATTGGTTTAGATCGATCTTAACCTGATGATTGATCATCATGAAGTATTT

CTATCGCAGAAAACCTGAATTTAGGTTTGAAATAAATTTACAAGAAATCCGGCCACTACC

AATCCTTAAACATTTCTGGAAACCACACTGGATCAGTATCGCAGTGCTCGTCAATCATTT

CATCCCCTACAATATCGACAAGTCCATAAGCTTTGGCTTCGTCTGCTGACATAAAAACAT

CCCTTTCCATGTCTTCGGATACAACCCAAAAAGGCTTGCCTGTTCTTAGGGCATAAACCC

TTGTGATCATTTCGCGAACTTTGTGTAACTCTTCCACTTCTAGTAAAAATTCTGGTGTCC

TTGCCCGATAATAAGCACTAGCAGGTTGGTGAAGCATAATCCTCGCGTGAGGGAATGCTA

TACGCTTGGTGGGTTCGCCTCCAAGCAGAATGAAGGATGCCATGGACGCAGCCATTCCGA

GGCATATTGTATATATATCTGGTGTCACCGTTTGCATCGTATCAAAAATCGCCATTCCTG

AGATTAGCCACCCGCCTGGGGAGTTTATAAACAAAAAAATATCGCTAATTCCATCTTCTA

TACTGAGATATACCATGAGACCTGTAATATGATTCGTGACCTCGCAACGAATCTCTTGAC

CTAAAAAAAGTGTCCTTTCTCGATACATAACATTGTATAAGTCAACCCAAGTCGCTTCTT

CATCTCCGGGAATCCGGTAAGGTACTTTTGGAACACCAATGGGCATATTAGATTAATATT

ATTAAATTTAAGTAAGAAAACTACACTTTAATATGGAAACGTAAGAATGGAAGAGAAAGA

AAGAATCCGCAGTTTATTGTCTTTTTTTTTTCTTATTCTATATGAATACTATAGATTCTA

TTAATACGTAGATTGAAATATACTATAAGGAAGGTAAGACAGAAAGAAAAAGGAATCGGT

GATTGGAATGATAAACAAAGAGGGATAGGGATCTATTCTTCGTTTTTCCAAATAGGCCAA

GCTACCCATTGCATATTGGCACTTATCGAGTATAGAATAGATCTGCTTCTCTTTCTTCTT

ACGAACAGAATTGGCTTCTTATTTTTAATGGAATGAAATAAATATTCACGCTTTCTGACA

CAGAATCCCCTAGAGGGGTTAGGTACATAGGATATAGATAGTCTTTTCCAATGCGATAAA

ATAAAGCGACATCGTGTCTATTTTTCTTTGCTAAAGGGGTATTTCCATGGGTTTGCCTTG

GTATCGTGTTCATACTGTTGTATTGAATGATCCGGGTCGATTGCTTTCGGTGCATATAAT

GCACACAGCTTTAGTTTCTGGTTGGGCCGGCTCGATGGCTTTATATGAATTAGCGGTTTT

TGATCCCTCTGATCCTGTTCTGGATCCAATGTGGAGACAAGGTATGTTCGTAATTCCCTT

CATGACTCGTTTAGGAATAACCAATTCGTGGGGTGGTTGGAGTATTTCAGGAGGAACTGT

AACGAATCCGGGTATTTGGAGTTATGAAGGTGTGGCAGGTGCGCATATTGTGTTTTCTGG

CTTGTGTTTCTTGGCAGCTATCTGGCATTGGGTATATTGGGACCTAGAAATATTCTGTGA

TGAGCGGACAGGAAAACCCTCTTTGGATTTGCCCAAGATCTTTGGAATTCATTTATTTCT

TGCAGGGGTGGCTTGCTTTGGCTTTGGCGCATTTCATGTAACGGGTTTGTATGGTCCTGG

GATATGGGTGTCCGATCCTTATGGACTAACTGGAAAAGTACAAGCTGTAAATCCAGCGTG

GGGTGTAGAAGGTTTTGATCCTTTTGTTCCGGGGGGAATAGCTTCTCATCATATTGCTGC

GGGTACATTGGGCATATTAGCGGGCCTATTCCATCTTAGTGTCCGTCCGCCTCAACGTCT

ATACAAAGGATTACGTATGGGCAATATTGAAACTGTACTTTCCAGTAGTATCGCTGCTGT

TTTTTTTGCAGCTTTCGTAGTTGCCGGAACTATGTGGTATGGGTCAGCAACTACCCCAAT

CGAATTATTTGGGCCTACTCGTTATCAGTGGGATCAGGGATACTTTCAGCAAGAAATATA

TCGAAGAGTTAGCGATGGGTTAGCCGAAAATCTTAGTTTATCAGAAGCTTGGTCTAAAAT

TCCCGAAAAATTAGCCTTTTATGATTATATTGGTAATAATCCGGCAAAGGGGGGATTATT

CAGAGCAGGCTCAATGGACAACGGGGATGGAATAGCTGTTGGATGGTTAGGACATCCCGT

CTTTAGAGATAAAGAAGGACGCGAGCTTTTTGTACGCCGTATGCCTACTTTTTTTGAAAC

ATTTCCGGTTGTTTTGGTAGATGAAGAGGGAATTGTGAGAGCGGACGTTCCTTTTAGAAG

AGCAGAATCCAAATATAGTGTTGAACAAGTAGGTGTAACGGTGGAGTTCTATGGTGGCGA

ACTTAATGGAGTAAGTTATTCTGATCCTGCTACTGTAAAAAAATATGCGAGGCGTTCCCA

ATTAGGGGAAATTTTTGAATTAGATCGGGCTACTTTGAAATCGGATGGTGTTTTTCGCAG

CAGTCCAAGGGGTTGGTTCACTTTTGGTCATGCTACCTTTGCTTTGCTCTTCTTTTTCGG

ACACATTTGGCATGGCGCTAGAACCTTGTTCCGAGATGTTTTTGCTGGTATTGATCCAGA

CTTGGATGCTCAAGTGGAATTTGGAACATTCCAAAAAGTTGGAGATCCAACTACAAGGAG

ACAAGCAGTCTGATACCACATTGCTATGGTATCTTTCATCTCTCTTTTTTGATTTGACAT

GGGAAACATCTCCCATCCTTTCTTTGACTCTTTTTCTTTCTTTATATGGGAAAAGATCCC

AAATGACAAATGAATAGGTGTGGAAGTTATAATTGTAAATAAACCACGATCGAATCTATG

GAAGCATTGGTTTATACGTTCCTTTTAGTTTCGACTTTAGGGATAATTTTTTTCGCTATC

TTCTTCCGAGAACCACCTAAGGTTCCGACTAAAAAATGAAATAATTTCATTGAAGTAAGA

AGTCTCCCAGAGGGGAGACTTCTTACTTCAATTAGTCCCCGTGTTCTTCGAATGGATCTC

TTAATTGTTGAGAGGGTTGCCCAAACGCGGTATATAAGGCATACCCAGTAAAGCTTACAA

GTAAACCAGATATGGAGATGGCGACTAAAGTTGCTGTTTCCATTTTTATAGAATTTCAAG

ATTACAATGGATCTACGAAAAGATCTTGTATTTACAACTACAACGGAATAGTATACAAAG

TCAACACCAATGATTAAATAGAATTTATGGCTACACAAACCGTTGAAGATAGTTCTAGAC

CTGGGCCAAGACGAACTCGCGTAGGTAGTTTATTGAAACCCTTGAATTCGGAATATGGGA

AAGTAGCTCCGGGTTGGGGGACTACTCCTTTTATGGGGGTCGCAATGGCTTTATTCGCGA

TATTCCTATCTATCATTTTAGAAATTTATAATTCTTCTGTTTTACTGGACGGAATTTTAA

TGAATTAGGTTTCTACTAACTAAAACTACGAAGTCATTGTTTTTCCATCCAAAAAAGCCT

TTCTACTTTAAGCTATACATTTCTAGACATTCTGGTAGTTCGACCGTGGATTTTTTTGTT

TTGGTATCTCTGGAATATGAGTGTGTGACTTGTTAGAATGGATCCTATTGATAATACATA

GAAAGGGCCTGTTATCTCTATCAAGATGATTCTAATTCGTCGGATATTTTTTATTCTAGT

ATCTGGAACACGAAATAGATAGAGTGGATCAAAAAAAATGGAACTATGATTCATACTCAC

TATTCAGACCTCGCAACCAGACTGAAAAAAATTCAAGTAGTTTTTAATAAAAAAAGAAAT

TTTCTTCCTTCCAATTTTGTTTGCCCAAAAGACAACTTTTTTCTCTTTCAATAAATGATC

ATCAAGCGGTTCTTATTCGAAGAACCCTTGCCTTTTGGTTAGCTTGAGACTCAATCATCG

TGGCTCTAGTATGAATCTAAGGTTTTAATTGAACTGATTCATAGGATCGCAACAAGATAA

TTTCTATCAGAAAACTACTAGAATTTTTGCTTTATTTATTTACTAGTCAAAAAAAAAAGA

AATCCAAAATAGGGAAGAGAAAAGTCAAGAAGCCTCTAATGACCAACATAAGGGAAAGAA

AGACAGATGAGCCAACTTGAGATTTTTTGGCATTATCATCACAAAGAATAAGTTCTGGAT

TTTTCTTATTTCATATCTTCAAGGCAAATCGACCCAATCCAGTGGCTGATGAAGTTTTGA

ACCTTTTTTCTAATATCCGTTGAAAATTTGTGTGTTTCTGCTTGAGCCGTACGAGATGAA

ATTTTCATATACGGTTCTCGGAGGGGGACTCGGGTTAGTTACCTATCTCAATAAAGTATA

TGATTGGTTTGAGGAACGTCTTGAGATTCAGGCAATTGCGGATGATATAACTAGTAAATA

TGTTCCTCCTCATGTCAACATATTTTATTGTTTAGGGGGAATTACACTTACTTGTTTTCT

AGTACAAGTCGCTACCGGTTTTGCTATGACTTTTTACTACCGCCCAACCGTTACAGAGGC

TTTTTCCTCGGTTCAATACATAATGACCGAGGCCAACTTTGGTTGGTTAATCCGATCAGT

TCATCGATGGTCAGCAAGTATGATGGTTCTAATGATGATCCTGCACGTATTTCGTGTGTA

TCTCACAGGTGGATTTAAAAAACCCCGCGAATTAACTTGGGTGACAGGTGTGGTTTTGGG

TGTATTGACTGCATCGTTTGGTGTAACTGGTTATTCTTTGCCTTGGGATCAAATTGGTTA

TTGGGCAGTCAAAATTGTGACAGGTGTACCTGAAGCGATTCCGGTAATAGGATCGCCTTT

AGTGGAGTTATTACGTGGAAGTGCTAGTGTGGGCCAATCCACTTTGACTCGTTTTTATAG

TTTACATACCTTTGTACTGCCTCTGCTTACTGCCGTATTTATGTTAATGCACTTTCCAAT

GATACGTAAGCAAGGTATTTCGGGTCCTTTATAGGGAAGCCATATCATAGAGAATTCTAA

TTTTCATATATCATATCGGGTAGGTTGTGGTATTTCATTGCTACAAACATGGGTTATTGT

AAAATAAGACATGTCATTTGGATACTTTTCTTCAACTCCGAAGTATTTTGATACAAATAG

TTGAAGTTCATTTTATGAAAGAAAATAAGGCGGATTATGGGAGTGTGTGACTTGAATTAT

TGATTTGGCCATGCAGATAAAGAATTGGATCTGCCACATTAGAATTCACAACAAAGTGTC

TCCGCATCCAATCAACACGTAAGTCCCCTATCTAGGAAGGATAGGCTGGTTCACTTGAGG

AGAATATTTCTATGATCATACCCCAACCATGTCATCCATGAACAGGCTCCGTAAGATCCC

ATAGAGTAGAAATAGAATAAGTCATGTGACATGATCCAATTCTCTATTTATTACACTTAC

TTTTTATTATAGTATGGAAATGCATTCATTTTCTTTGCATCGATTGCGATCCGCAATACT

ATCGGAGTAAAAGAAGGTATCTAAAGAAGAACGTAGGCTAGACTTTTTGATTTTTTATTA

GTAACAAGTAAATACTTTGTTTGGACGTAAGAAACTTGCGATATTGAGGGGATAAACACC

AACTAATCAAGAGACAATCCACAAAGCAATTGATCATGATCAAATTTGCAAGCCAACTTG

GATATTGAGCATTTACCCATAAGAATAAGATTCTTTTCAATAAGTAGTTGTAGGTGCAAC

TTCGGAAAAGAGAATCTGATAAAGCTTTTCTTACCTAGAGTCATTGAGTCATTATATACC

TTATTCTATTATGGATCTTCCACGGTCTTTTTTCTTTCATTCTTGCTCGAGCCGGATGAT

GAAAAATTCTCATGTCCGGTTCCTTTGGGGGATGGATCCTAAAGAATTCACCTATCCCAA

TAACAAAGAAACCTGACTTAAATGATCCTGTATTAAGAGCAAAATTAGCTAAAGGGATGG

GACATAATTATTACGGGGAACCCGCCTGGCCCAACGATCTTTTATATATTTTTCCAGTAG

TAATTCTAGGTACTATTGCATGTAATGTAGGTTTAGCGGTTCTCGAGCCGTCAATGATTG

GTGAACCGGCGGATCCGTTTGCAACTCCTCTGGAAATATTACCCGAGTGGTACTTCTTTC

CCGTCTTTCAAATACTCCGTACAGTACCCAATAAGTTATTGGGCGTTCTCTTAATGGTTT

CTGTGCCAACGGGCTTATTGACAGTACCCTTTCTAGAGAATGTCAATAAATTCCAAAATC

CATTTCGTCGCCCAGTAGCTACGACCGTTTTTTTAATCGGTACTGCAGTAGCTCTTTGGT

TAGGTATTGGAGCAACATTACCCATTGAAAAATCCTTAACTTTAGGTCTTTTTTAGGGAT

TTTTCAGGTTGATTCATTCAACCGTGAAGTACCGTGCATAGGTATCTAGGAAATAGTTAC

TTCCAAGTGAATCTTCCCTAGATACCTAAAATCCATTTTATTATGATCCATTTCGCGAAA

ATATAGATTGTGCCAAAGATGCAAAATTGTTTTTTTTTATTCTAACTCGAAAAAGAAGAA

GAGGAAAAAATTGCAATGATTTAAAACGAGAACTTATTCTTAGGTAAATCGATTGGGAGA

TGCTTCTCTAGAGTGTCCCATATCTGTTTTCCATCTTCCATACGAAAACTGTCAATTCTC

ATAAGATCTTCTTCAGTCTTACTCAAAAGGTCCAATAGTGTATGTATATTGGCCCTTTTG

AGACAATTATACGTTCTAGAAGGCAATTCTAATTGATCAATAAAAATACAATTCAATGGA

ATTCCTTTTTTGTTTTTCTTTAGATTAGTTAATCTTTTTTGAAAGGTTAAAAGGGGTGGA

GTAAACCTGTTTTTATTTTCTTCGAAACTAGTGCCCTCTTCCTCCGCGTGTAGAAAAGGA

AGAAATAAATCAATCAAATTACGAGAAGCCTCATAAAGCGCTTCCTTAGGGGTTAAACTT

CCATTCGTCCATATTTCTAGAAAAAGTATCTCGTGTTTTTCATTTCCATTCCCACAAGAA

AAAATACTATAATTCACATTTCGAACAGGCATGGATACAGCATCTATGGGATAACTTCCA

TCTTGAGAGTTCTTTCTGAGTTCCGTGTGATATCCGCGATCTCTCTTGATCTGTAACTCA

ATACAGAAATCAATGGGCTCTGTCAAGTTAGCTATAGGTTGTGCCGTATCAACGATCTCT

ACGGAAGGTGGTAAGATGATATCTTGAGCAGTTATGTATCTAGGACCTTTGACGCAAATT

GATGCGTCTCTAACTCCATAGAGATTACTTCTCAATACAATTTCTTTCAAATTTAGTAAA

ATTTCTTGTACGGATTCTTCAATACCAGCTATTGTAGAATATTCGTGTGGCACGCTCCCA

AATTTTGCACGTGTGATACATGTTCCTTCTATTTCTCCAAGTAAAGCTCTTCGCAAGGCA

ATACCGACGGTATCCGCTTGACCTTTTCTAAGCGGGGACAAAATGAAACGACCATAATAA

AGACGCTTACTATCTACTCTTGATTCAACACACTTCCACTGTAGTGTTTGAGTGGATCCT

GCTACCTCCTCTCGAACCATATAGACTATATTATTTGATCATTGAATCGTTTATTTCTCT

TGAAAGCGTTTTAATTCTTTTACAGACGTCTTTTTTTAGGAGGTCGACATCCATTATGCG

GCATAGGTGTTACATCGCGTATACAACTTAATCGTACACCACTTTTAGCAATGGCTCGTA

ATGCGGCATCTCTTCCACTACCAGCACCCTTTACCATAACTTCTGCTCGTTGCAAACCCA

CTGTACGAATAGCATCTACTGCTGTTCTTTGACCAGCATAGGGTGATGCTTTTCTTGAGC

TTTTGAATCCACAAGTACCCGCGGAGGACCAGAAAACCACCCGACCTTGCGGGTCTGTAA

CAGTTATAATGGTATTGTTGAAACTAGCTTGAACATGAATAACTCCTTTTGTTATTCTAC

GTGCACTTTTCCGTAAACTAAAACGCGCATTCCTACGCAAACCAATACGCACTCTCCTAC

GTGAACCAATTTTTGGTATAGCTTTTGTCATATTTTATTATCTCATAAATATGAGTTAGA

AATAACAAAAAGAAAAAAGATACAAAGATATCCGTTTCAGGGTAAAATATATCCTTTACT

TTAATTATTTTATTTGGAATTTGGACGTTTCCGCGGGTACTTTTTACTTTTTAGAAAGTA

AAGTTCTTTTTCGAAAGATTACCCCTGCCTTTGTTTATGCTTCGGATTGGAACAAATGAC

TCTAATTCGTCCACGCCTACGAATCAGTCGACATTTTGTACAAATTTTACGAACGGAAGC

TCTTATTTTCATATTTCCTTATTCCTTTCTTAAACTATGAATCTAATCTTTGGAAAAAAT

AAGTCTCTTCGCTTGAATTTTGGAACTTTGAATTTATTACCCTAGAAAAACCTAATCCTT

TGAATCTTTGGTATCCTTCAAATCTTCGGTATCCTTCGAGTCTTCGGTACGCTTCGAATC

CTTATGGGGAAGTCTATAAATTATACGTCCTTTGCTTGAATCATAACGACTTACTTCAAT

TTTGACCCTATCCCCCATCAGTATTCGTATAGAACTAGACCGGATCTTTCCTGAAATATA

GCCCAGGATGATGGTGTCATTCTCTAGGCGAACGCGGAACATTCCGTTGGGTAGGGCTTC

CGTAACTAAACCTTCGAAAGTGACTTTTGCTTCTCTCGGGTTTTTTTTTTCTGTCATATT

TTTTTCTCCTATTTTTCTATTTTGATTTTCAAAATAGGAAGTTCGAGATAGAATTCGAGT

ACTATAGGAGGGGCGATTACCATATATAACATAAGACTTCTCCCCCAATTCTGTTTAGTC

GAGCTTCTCGATCTGTCATTATACCTCGAGAAGTAGAAAGAATAGCAATTCCCATTCCGC

CCAAAACTTTAGGAATTCCTTGATAGTTGGCATAAATTCGTAAGCCGGGTCGGCTGATAC

GCTTTAAAAAGGTTCTAGTTCTATATATTCCTTTTCTAGTCTTTCTCTTTTGATGTCGCA

AAGTTGAAACCAAGAAATATCTGTTACTTTCCTGATGTTTCCGAACACTTTCAATAAAAC

CCTCTCGTAGAAGTATTTTAACAATGTTTTCGGTAATATTTGTAGATACTACTCGAACTG

TTCCTTTTTTATTCATGTCCGCGTTTCTTATAGAGGTTAGTAAATCAGCAATAGTGTCCT

TGCCCATAAGACTCTAATTCTAGGTTCCTCCTAATTTTTCTATAATCAACATGTTTTCTT

TTTCTTTTACTTTTGGATTTTAAAGCATATACGTGAGACATAATCTACTAATTTTTTCTT

TGATCTATATCTCGCCTACTAGTATTTATAATACTTCAGGAGCTAATGAAACTATTTTAG

TAAAATTCAATTCTCTCAATTCCTCGGCGATCGCGCCAAAAACTCGAGTTCCTTTTGGAT

TTCCTTTTTGATCAATGATAACCGCTGCATTGTCATCATAGCGTATTATTATACCGTCTT

CGCATTTGAACTCTTTACATGTACGTACAATTACAGCTCGAATTACTTCGGATCTTTCTA

GAGGCATTTGGGGCACTGCGTCTTTGATTACAGCAACAATAACATCACCAATACGAGCAT

ATCGCTGATTACTAGCAGCTCCTATGACTCGAATACACATCAATTTTCGAGCTCCACTGT

TATCTGCTACATTTAAAAGGGTCTGAGGTTGAATCATATTATTTTGATTTCAATTTGTTA

TTTCTATGCAAAGGATGAAAGAAATATTGTCTTTCCAGAAAAAAACCTGGTTTTTTACTT

CAATACTCCTTTTTGGGATGCTATATCTCTAATCGAAGAAATTGACTTCGTATGGGCATT

TTACTGGCAGCTATGGAGATAGCTGCTCTAGCTACAGTTTCAGATACTCCGCCCATTTCA

TAAAGTATTCGACCTGGTTTAACAACGGCTACCCAATATTCGGGGGATCCCTTTCCTGAG

CCCATACGTGTTTCCGTGGGTCTTAGTGTAACCGGTTTGTCGGGAAATATACGTACCCAT

ATTTTTCCACCACGACGTGCATATCGTGTCATTGCTCTTCGTCCTGCTTCTATCTGTCTC

GACGTGATCCAAGCGGGTTCAAGTGCTTGCAGAGCATATCTACCAAAACAAATACGATTG

CCTCGGCAGGATTTTCCCTTCATTCTTCCTCTATGTTGTTTACGAAATCTGGTTCTTTTG

GGGTTATAGTCGATGGTTCTTTCTTAGTTCCATCTCTACTGCAAAACTGGACATGAGAGT

TTCTTCTCATCCAGCTCCTCGCGAATGAAATGAGAAGCGTGCAAATTTCTCTAATTCCAT

AATATTTTGGAATATTATGGATAGATGCACATTAATAGATAAAGTTAGGGTTTTTAATAT

TGAATTTGTTAAAATAGAAAAATATATAGTCAAGAAAGAAGATCTAGAATATATCTAGAT

TCTATATTTAATGTAATCTTTCTTAATCTCCTTATTTATTCAATAAAGATTTCGCGGGCG

AATATTTACTCTTTCCTGTCTTATTTGTTAATTTATAACCTTACCAAATAAGGCAATTTT

TTGGTTTGTCCGCCATCCACCAATGAAGTCTTAGGATTCTTTCAATAAATCCTATGCAGT

CATAGGTTCTGTCGTTCCCACTACTTCTCCTTTAATGGTTAGGTCTGAATCCCACAATGG

AGCTTTCAAAATTTCTTTCCGAGTCAATTTCTCAGTTTTATTAACCCGGCCGCTCTTTAT

TATTGCTTAAAATTTCTATTTTTTGTTTATTCTCTGTTATTTTTATTATATTGATGCTTT

ATCACATTGCCTTTTATGATGAACTCATAGACCATACATATTGGAATCCTATATCTTTCT

TATTCTTCTTCCTTCTTTCTATCATCCCTCCTTTTATCCACATCCCTTTAGTTTTGCTTC

ACAACCTAGAATCCTTTTCTTTTTTAGAGAAAAATTGCAGTTGCTACAACTATATGATAG

ATCTACTCATTTATGATAGATGTATCATATAGTGACTGTTTCTTAGTTAGGATCTCGACA

ATACGAAGCAATAGGTTGGTTATTAGTTAATTTTCTATAATTTTCCATTTTTGACTCTAA

AAAAAAACTAACAGTCACACACTAAGCATAGCAATTATATTAAAAGATTTATCAATTTTC

ATTAAATCTTATAGAAAGAGGTAGAATTCTTCTTTTTTTCAGGGATTTCAGGAAAAATAA

GGTCTTGTCTTTTTTATTCTATCACTGACAGAATGGGAAGACAAGGCTAGTTATTCTTCG

TCTACGAATATCCAAATTTTTACACCTAATACTCCATAGATAGTTCGAATTGGATAGCAG

CAATAATCAATTTTAGCGCGAATTGTTTGGAGGGGAAGTCTACCCTTTTTGATGCATTCG

GCACGTGCAATTTCTTTCCCTGCGAGACGACCTGCAATTTTTACTTTTACCCCCCTTATA

TCTGCTTTTTTAGTTAATTCAATGGCTTTTTTCATTGCCTTTCGGAATGAAACTCTATTT

TTTAATTGGAAAGCTATATATTCTGCAAGAATGTTAGGTTGTCTATAAGGTTCTTTAACT

TTTTCAATAGCAATATTAAGTCTCTGGTTTACAGAATTAACTTCCTTTTGTAGATCTTTC

TCTAATTCTTCGATTGCTCCTTTTTTCTTTAATAAGTTGGGGAATCCAATATGGATTATG

ACGTGGATCGTATCGATTTCTTTTTGAATTTCTATACGTGTAATTACTTCAGAACTTGAG

CCTGAGTCCATTTTTCTATTCGAGCCTTTTTTCCTATTCTTTTGTATATAGTTCTTGATA

CAATTCCGTATTTTTTTATCTTCCTGTAGACCTTCAGAATAATTTTTTGGTTGTGCGAAC

CAAAAGGAATGGTGATTTTGGGTTGTACCAAGTCTGAAACCGAGTGGATTTATTTTTTGT

CCCATATTTTTCTATTCTATTTTTTTTACTGGGAATCAAATCTAAGATGGATCTAAAGAT

TATTTAGATTTCTTTACTATATTTAGTACAATTGTTATATGACACATGGTTTTTTTTATG

GGACAACTACGTCCTCGAGCTCGAGGTCTTAATTTTTTCATGATAGTACTCCTACTGACT

TCGGCTTTAGTGATGAATAAATTCGCTTTGTCGAAATCCCTATAATGAGTAGCATTTGCT

GCTGCCGAATAAACCAACTTTAGGATGGGATAAGATGCTCGATAAGGCATGAGGTTCAGT

ATCATAACGGTTTCCTCGTAGTAACGCCAGCGAATCTCATCAAGAACTCTTTGTGCTTTG

AAAACAGACATATGGATGCGTTTTTGTGCTTTGAAAACCCGTTCGAACTTAAGTAGACGA

TCGGTTGGAACTTTCCTTGCGAGTTTCCTTAGCCCCTTCTTCTTCTTTATCCTAGGGGTA

TACTTTACCAGTTTGAAACTTGTCATAAATAAGGTTATTCCCCGCCTACCTTTGTTTTTT

TTATTTTGAATCTTTCTATTCTGAATTCAGTTAACGACGAGATTTAGTATCTTTTCTTGC

ACTTTCATAACTCGTGAAATGCCGAGTAGGCACGAATTCCCCCAATTTGCGACCTACCAT

AGGATTTGTTATGTAAATAGGTATATGTTCCTTTCCATTATGAATCGCGATTGTATGGCC

AACCATTGCGGGTAGAATGCTAGATGCCCGGGACCACGTTACTATTGTTTCTTTCTCCTC

CTTCATATTGACCTTTTCGATTTTTGCCAATAAATGATGAGCTACAAAAGGATTCGTTTT

TTTTCGTGTCACAGCTGATTACTCCTTTTTCATTTTAAAGAGTGGCATTCTATGTCCAAT

ATCTCGATCGAAGTATGGAGGTCAGAATAAATAGAATAATGATGAATGGAAAAAAGAGAA

AATCCTTTAGCTGGATAAGGGGCGGATGTAGCCAAGTGGATCAAGGCAGTGGATTGTGAA

TCCACCATGCGCGGGTTCAATTCCCGTCGTTCGCCCATCGCATTATTGCAAATTCCAAAA

ATGCAATTTTCCATATTCCTAGTTACGTATTTACTTACGGCGACGAAGAATAAAACTATC

ACTATATTTTTTCCTTTTCCTAGTTCTTCTTCCAAGCGCAGGATAACCCCAAGGGGTTGT

GGGTTTTTTTCTACCAATGGGGGCTTTCCCTTCACCGCCCCCATGGGGGTGGTCCACAGG

GTTCATAACTACCCCTCTTACTACGGGGCGTTTACCTAGCCAACACTTAGATCCGGCTCT

ACCCAAACTTTTTTGGTTCACCCCAACATTACCCACTTGTCCGACTGTTGCTAAGCAATT

TTGGGATACCAAACGGACCTCCCCAGATGGTAATCTTAAAGTGGCCGATTTACCCTCTTT

TGCAATCAGTTTCGCTACAGCACCTGCTGCTCTAGCTAATTGCCCACCCCTTCCACGTGT

GATTTCTATGTTATGTATGGCCGTGCCTAAGGGCATATCGGTTGAAGTAGATTCTTCTTT

TCTCTCAAAAACCCCTTCCCAAACTGTACAAGCTTCTTCCAAAGCATACAGCTTTCTAGA

TGTATATGACGATCTCTAGACAGATGGATCTTATATGAATCGTATGATGAAGTACCACAT

GAGTGGATATATAGGAAAGGAATCCAAATCTGCCGAATCGCTCATGTTATGATCTTCTAC

ATCCTAGGTCTCCGCGTTCCGTCATCTGGCTTATGTTCTTCATGTAGCATTCAGATCGAA

TGACTCTATGAAATTACGTCGATACTTCCACATATTATGGGTAACGTAGGAGACATCCCT

ATTTTCCCCGGGGGGTCTTAATTACCACTGCTTAGCTTTCAATTCGCCTCTGACCATCAA

ATTAAATGTGAATAACCCGTCCTCCTCTCTTTGAAACAAGGGGCGCTTCCGGTTCTGTGC

GTGCTTCAAACAATTTTGTCTTCTCCATATTACCATATCTCTAGAGTCAATAATTTTCTA

TGAGGAACTACTGAACTCAATCACTTGCTGCCGTTACTCAACAGTTTTCTGTTGAGGTCT

ATCCCGTAGAGGTAGTCAAATTGGATCAGTGATCGATTTCTAGGTTTCGTCGTAAACCTA

ATTGGTTACTTCCAATTACGTAAATCAATAGTTCAAACCGCACTCAAAGGTAGGGCATTT

CCCATTGATATAGGAACTTTTGTACCAGAAACAATAGTATCTCCAATTATAGCCCCTCTG

GGATGTAAAATATATCTCTTCTCACCATCCCCATAGTGTATGAGACAAATGTATGCATTT

CGATTAGGGTCGTATTCTATGGTTACGATTCTACCAGATATGTCTTTTTGATTCCGTCGA

AAATCGATTTTACGGTATAGGCGCTTATGACCTCCCCCTCTATGCCTTGCGGTAATGATT

CCTCTGGAATTACGACCTTTACCACAACGGTGCCGTCCATGGATCAAATTATTTCGTGGA

TTGGATTTCACTTGCCTGTCTACGGTTCCCTTGCGTGTGCTCGGGATAGGTGTTTTGTAT

AAATGTTTCGCCGTATTATTAAGTATTCTCCTTTAGTTTTTTTCTCTATCTAGAAGTGGA

ATAGAATAACCCGGTTGAAGGGTAATGATCATACGTCTGTAATGCATTGTATGTCCCAGA

ATAGGTCCCATTCTTCTACCCTTTCCGGGTAGTCGATGGCTATTCACAGCTACTACCTTA

ACACCAAAGAAGAGTTCGACCCAATGCTTTATTTCTGTCTTAGTGAATCCCGATTCGACA

TTAAAAGTATATTGATTCTTTCCCAATAAACGAAGACTTTTTTCTGTAAATACTGCGTAT

TTGATTCCATCCATAAATCGACTTTCCCTCCTATGCTCTGAGTTCCAGTATCGATAAGAA

TTCGAGTTCTTATTGTTCTTATGTTATGGTATGAATATACCATACCAATTCGTTATGTAT

GGATGATGGATGAGATTCCATGGATAGAGAGCCAGTTCCAATAGACCGTTCCCGTTCGCG

TGCATCCAGCAGGAATTGAACCCGCAAATTTACCAATTATGAGTTGGGCGCTTTAACCAT

TCAGCCATGGATGCTTAACAGGGATCATCGTACATCGTAAATAACCAATTTTCATATAGA

AAGACATATCATAGAAAAATGAAATCGAAAATATTCGGAGATGGCAAATATTCGGAGATG

ACTATGAAACACCTCTCTGGATCCTCGAATTGAAAGAGAGATTGAGAGGGATCAAGAATC

CTAATTCTCGCTATTTGGAATGGATCCAATTCTATTGAGTCTGACTCATAGTGATCATTT

CTCTTTAGCAAAGAATGACCTTGGTTATCAAAGGATTGAACAACCGGGATCCATTTACTT

ATGATACCTAGTTGACATTGATAACAAGGATCTAATGAATTATGAGTTTAATAGATCCTC

TTTAGCAGAAAGACGTATATTCCTTGCTCATCACTTATCCCAAACTCGTGTGGGCTAATC

GTTTTCATTTACCATCTCATGGAAAACCCTTTTCGTTCCGCTTAGCCCTATCGGGTATTT

TAGTGATAGGTTCTATAGGAACTGGACGATCCTATTTGGTCAAATACCTAACGAAAAATT

CCTATTTTCCTTTCATTAAGGTACGAGGGCTTCTTATTCCACAAGAACGAAAGCACCTTT

TCATTCTTTCATATACTAGGGGTTTTTACTTGGAAAAGACAATGTTCCATACTAAAGGAT

TCGGGTCCATAACCACGAGTTCCAGTGCACTAGATCTTGTAGCACTTAGCAACGAGGCCC

TATGAATAGACATATAGAATTTTTGGTCGGGAAATTCGAATGAATCATTGAGTGAAAAAG

GAGCAAAGAATGACAAAAGACGAGACTCTACTAGTCTTCACTCTTGTGGTTTCCTCGGTT

TCTGTTTTCTTATTCGGGATCTTGCTTTTCATGGTTCTCATCTCTGCAACTCGCGATTTT

CGCGAGAGAACCAAATCCAAGTTGGTGAAGATCATGATTTGGGCTGGCATAGTAGTTATT

ACCTTTGCAATTGCGGTTCGAATCTATCCGATCTTTATCTTTTTGCTCAAAGAACGAATA

AAACCCCTTGTCGAAGCCCTTTATGATAAGCTTCCCTGGATCTGGGAAGTTTCTCTTTCA

CGGTATTGGGATCGTTTGATCGATTTCCTTGATCGCTACTTATGGGCGTGCGCTCAAAGG

ATACAAACAGGAATTCGCAAACAAAAAGGGGAATTCGTAGTCACTTTTTCCTGTCGCGTA

AAAAAAAGGCTTTACGCGAGAGCAATAGAGGTTGGGATACATCTATCTCTTCTGAGCAAC

CTCTTTTGGATTCTTAAGACCACCCTTGCAGTAGGATACCGTCTGCTTTGGGTTCTTTAT

TATCTCCTTCCAGGGATTTTTAGGATCGTTCAGGCTATATTTAGTCTATTTTGGCTTTTA

CTGTCTACTTTTCTCAGGGAGATGGTTAAGGACCTCAGAAGATAGAGGAGAGCGCCAGGC

GCAGATTTCCGGAATACTTCTACGGGGAATGCTCATTCATGAGCATTCTCCATATTATGC

CTTGAAGAGGACTCGAACCTCCACGCTCTTTAGCACGAGATTTTGAGTCTCGCGTGTCTA

CCATTTCACCATCAAGGCATCTTGAAAGTGAATCGTATTCCATGAATATGATATCTATCT

AATGTGATATATTCCATATATGACAAAGGTGGAGTCTTGGAGTATTTCGATCGATCGGTC

ATATAGGCCTGAGTCAGACATCAAATAGCTTCGATTTGCATTATCCGTAGAACACCTTAT

ATGTATCAAAATCGAAAAGATGTACAATCCAATTTCTCGATTCAATAGAAGCCCAAAGAG

GTGCATATGGTACCCAAATAAGGATAGGATAGATATGTCAAAAGCAGGTCTGATTACACC

TATTCCTAATCCTAAATAGAATGTAAGGGCGTAGGGATTTCTATGTAAACAGAGTATCCT

ATTTCCATAGGCTCGAATGACCCCTTCTCATAATAAGAATGTGCACGGTCTGGTCCGGTA

TGGAATGAACTTATAATCTGATGATCGAGTCGATTCCATGATTATAAGTTCATAACCCTA

GCGCCCATTCCCATTTTGGGCGGAACAGATCTACTAATTCTTTTATTCCAGTTAGTAAGA

GGGATCTTGAACTAAGAAATAGACCTAGCAGCTAAAAGAGGGTATCCTGAGCAATTGCAA

GAATGGGGTTCATTGATATTCCTGGTATAGTAGATGCTATCACACATACAGTCATACTCA

ATTCGATGGAATTGTTTGATCTTAAAGGGGATCTTCTATAATTTCGCACATAAGGGGTTA

TTTCTTGGTTTCGTCCAGTCATTAATAACTTGATTATTTTTAGATAATAGTAGATAGAAA

GAACGCTCGTAAGGAGTCCTATTGAAACCAAGAAATATAGGCCTGCTTGCCATCCACACC

AGAATAGATAGAGTTTTCCGAAGAAACCCGCTAGTGGAGGAAGGCCTCCTAGGGATAAGA

GACATAGGGCTAAAGAGAGAGCCAAAAAAGGATCTTTCGTGTATAATCCTGCATAATCTC

GAATGTTATCAGTTCCGGTACGTAGACCAAATAATACAATGCAAGCAAAAGTTCCTAGAT

TCATGGAGATATAGAACAGCATATAAGTTATCATGCTTGCATATCCATCATTTGAGTCTC

CAACAATTATTCCAATAATTACATATCCGATTTGCCCTATGGACGAATATGCAAGCATAC

GTTTCATGCTTGTTTGAGTAATAGCAAGGAGATTCCCCAATATCATGCTAAGAATAGCTA

GGATTTCCAGAAGAAGATGCCATTCGTTTGATGAGAAATAAAAAGGAATATCGAGAATTC

GCGTGGCTGAAGCTGAAGCAGCTACTTTCGAAGTAACAGAAAGAAAAGCAACGACTGGAG

TGGGGGAGTCAGAGTCGAAAAGAGGATTCCTCGCTTCTTTCTCTCATGCAAAACCGTGCA

TGAGACTTTCATCTCGCACGGCTCCTAAGTGATAAAAGAAAGAAGAACTCGTCTTCTTTC

TTTTTTGATTACCTTCCTCGCGTATGTATAAGACCCAATCCATTCTTTTTCGAAATCGAT

TTCGAAAAAGAACTACTAATCCTTAACTTTTCGAGGAATCCTTCATCAGTGGTTGTGAAT

GACTGACTTTTTCAATCCTTTCGACTTTGGTTCCGTAGGAGCAAGTCAGAAAGGTTGAGA

AATAGAACCATCTGATTTGATTCGTTCCCAATAGCCATGAGATGATTATCTTAGGGTGAT

CCTTTTGTCAACGGATGCTCCTATTACACTCGTAGTCTCTGAAGGATGAGAACCCACTAT

GTAGCATCTACATTGATAATTCAAGCATTGTATACGTCATTAGTCCGATTCTTTGTAGGA

ACTACCCGTAATAACGAGCTTGCAAAATGGATCTGTTTATCATAAAGAGATTCGTTGTTC

CTGACCCTGCTTCACCTTAATTGTTATTTGAACAAAAAGATCACAATAAACTTTTGGTAA

AAGTTCTGTCTTGGTCGGAGTGGGGATAGCATTTCTCTTCTGCATGTCTATGGAGTTTTG

CAAAACCCAAACACCTCAGATAGAGGTAGGAATTTGTCGAACGAACCACACTCCTTCGTA

GACGTCAGGAGTCCATTGATGAAAAGGGGCTGGGGAAAGCTTGAACCCAAGTCCTACAGT

GATGGATATAAGCGCAATTGAAATTCCTGGGGAGTTATACATTTGTGTATTGATAAGACC

GTTCACAATTTCTTGAAGCTCGATCTCCCCCCCAGATGAACCATATAGCCAAGAGAAACC

ATGAACCAGAATAGAAGAGCTTGCCCCACCCATGAGTAAATATTTCATAGTAGCCTCATT

AGACCGTAGATCTCTCTTGGTATATCCAGACAATAGGTAGGAACATAAACTGAAACATTC

TGGAGCTACAAAGATAGTTATTAAATCGTTAGCACCACATAAAAACATTCCCCCTAGAGT

AGCTGTTAATACGAATAACAGAAACTCTGTTATAGCCATTTCTGTACATTCAATGTACTC

TACGGATAGAGGAATACATAAAGTTGAACATAATAAAATAAGAAATTGAAAGATTTCGTT

GAAATTGTTCGTTTGGAAATTTCCCGAAAAGCTAATTATAGGTTCTTCTCTCCATCGGAA

CAATAGGGCCGTTATGCTTATTACTAAACTTGTTGAAGAGATGAAATAGAACCAAGGTCT

ATCTTTTTGATCAGAGGTTGAATCGATCATCAGAAGAAGAATTAGGCCAAAAATTAGGAT

ACATTCTGGGAAAATGAAACTTCCATGGAAGAGAAGCAAATGAAACGCTTTCATAAAAAT

TCTCGTAGAATCGAGAATGAAGTTTTCATTCTGTACATGCCAGATCATGAATTAGTAACT

GCATCCAATCTCCGAAAAGTCCCGATTGTTTCGATTTTTGGAATGGGATATTTACGGAAT

CCCCATGAATAGGATCAAACCTTATTCCATGCTATTTCCATAAGATTCCTCTTTCTTATT

CTTAAGCAAGCCCCCGAGAGGGCTTAGTTGATCATGATTTCTGTTTTCTCTTTCTTTTCC

TTTTTGTTTGTTTCGAGAAAGATATCGTCCGATTCTCCTTCTATTGATTCTTTTCCGATC

GAGATGTATGGATCCATGTGTCTACATACCTAGATTCTGTTCATGGATTAACGAAAATGT

GCAAGAGCTCTATTTGCCTCTGCCATTCTATGAGTCGCTTCCTTTTTGCGTATGGCACCC

CCACTCCCTTTGGCAGCATCTACTAATTCGGAACTTAATTTGAAAGCCATATTTCGACCC

GGACGCTTTTGGGATGCTTCTAATAACCAACGAATGGCAAGTGCTCTTCCTTGTTTAGAT

CCTATTTCAATCGGAACTTTCCGCGTCGATCCTTTTTTATTACGTCTTGTTTTTACTCCT

ATATTGGGAGTTACTCTACGTATTGCTTGACGTAAAACCAATAGTGGATTTGTTTCTGTC

TTTTGTTGAATCTTTTTCACGGCTCGATAGAGAATTTGATAAGCCAATGATTTTTTTCCG

TCTTTCATAATACGGTTAACCACCATGTTAACTAATCGATTACGAAAAATTGGATCGGAT

TTTGCAGTTCTTTTTTCTGCAGTACCTCGACGTGACATGAGCGTGAAAGAGGTTCAAGAA

TCCGTTTTCTTTTTATAAGGGCTAAAATCACTTATTTTTTTGGCTTTTTGACCCCATATT

GTAGGGTGGATCTCGAAAGATAGGAAAGATCTCCCTCCAAGCCGTACATACGACTTTCAT

CGAATACGGCTTTCCACAGAATTCTATAGGGATCTATGAGATCGAGTATGGAATTCTGTT

TACTCACTTTAAATTGAGTATCCGTTTCCCTCCTTTCCCCCTAGGATCGGAAATCCTGTA

TTTTCCATATCCATACGATCGAGTCCTTAGGTTTCCGAAATAGTGTAATGGAAAAAGAAG

TGCTTCGAATCATTGCTATTTGACTCGGACCTGTTCTGAAAAAGTCGAGGTATTTCGAAT

TGTTTGTTGACACGGACAAAGTAAGGGAAAACCTCTGAAAGAATTTCCATATTGACCTTG

GACATATAAGAGTTCCGAATCGAATCTCTTTAGAAAGAAGATCTTTTGTCTCATGGTAGC

CTGCTCCAGTCCCCTTACGAAACTTTCGTTATTGGGTTAGCCATACACTTCACATGTTTC

TAGCGATTCACATGGCATCATCAAATGATACAAGTCTTGGATAAGAATCTACAACGCACT

AGAACGCCCTTGTTGACGATTCTTTACTGCGACAGCATCTAGGGTTCCTCGAATAATGCG

ATATCTCACACCGGGTAAATCCTTAACCCTTCCTCCTCTTACTAATACTACAGAATGTTC

TTGTAAATTATGGCCAATACCTGGTATATAAGCAGTGATTTCAAATCCAGAGGTTAATCG

TACTCTGGCAACTTTACGTAAGGCAGAGTTGGGTTTTTTGGGGTTGATAGTGGAAAAGTC

GACAGATAAGTCACCCTTACTGTCCCTCTACAGAACCGTACATGAGATTTTCACCTCATA

CGGCTCCTCGTTCAATTCTTTCGAAGGGATCCTTTTCCTCGTTCGAGAGTCTCCGCCCTT

CTTCCACTCCGTCCCGAAGACTAACTAAGACCAATGGAGTCACGTTTTCATGTTCTAATT

GAACACTTTCCATTTATGATTAAAGGAGAAGATTGTTCTTTTACCAAACATATGCGGATC

AAATCACGTCTTATAATAAGAAGAAATCTTTCTCGGTATCAATCCCCTTGCCCCTCATTC

TTTGAGAATCAGAAGGATCCTTTTCGAGTTTCCATTTCTTCATTTGGAATCTGGGCTCTT

CTATCTTCGACTTATTTTTTGGCTTTATTCTTTATTTATTTCATTTCGATTTTTCCCTCT

TCCTCTATCCCTATCCTCTAGGTACAGCGTTTGCATCAATAGAGAACCTTTTCCTCTGTA

TGAATCGATATTATTCCAATTTCTTCCCGAAACTTCCCAAGAAAAATCCCGAATTGGATC

CAAAATTGACGGGTTAATGTGAGCTTATCCATGCGGTTAGGCACTCTTCAAATAGGAATC

CATTTTCTAACTGGCTTTCGTGCTTTGGTGAGTCGTCCGAGATCCTTTCGATGACCTATG

TTGTGTTGAGGGGATATCTATATGATCCGATCGATTGCATAAGACCCGCGGTAGCATAGA

ACGGGGAAAGTATACAGAAAAGACAGTTCTTTTCAATTTCGATTATCTATATATTAGTTC

GTTTCTATTTCTAGATATCTATTTCTATATATTAGTATTAGTTAGTAGTACTATTCTATT

AGTTAGCGATCCCGGCTCTGTGAGTTCTTTCTTCCGTGATGAACTGTCGGCACCAGTCCT

ACATTTTTTCTCTGTGGACCGAGGAGAAAGGGGGTTAGCAGGATTACCATGAGAGAACAA

CCCGCTTCAAATATGGAACATGGATTCTGGCAATGCAACGGAGTTGGGTCCTCATATCGA

TCCGAATGAATCAGTCTTTCTACAGAGGTCAATCTTTCTCGATAGGACATGGATTTCTAT

TCCTATGAAATTCATAAATTAGTTAATGGAGGGCTACCATTCCTTTTTCTTTATGTGTTC

CTAAGAGAAGGAATTTGTCCATTTCATGTTTCGAGGTCTCAAAAAAGGGCGTGGAAACAG

ATAGAAACTCTTGAATGGAAATTGAAAAGAAATGTAGCCCCAGTTCCTTCGGAAATGGTA

AGATCTTTGGCGCAAGAAGAAGGGGCGACCCGTATCATCTTGACTTGGTTCTGCTTCCCC

TCTTTTTTTAAGAATACCGAGTCGGGTTCTTCTCCTACCAGTATCGAATAGAACATGCTG

AACAAGATCTTCTTCATGGAAACCTGCTCGATTTAGATCGGGAAAATCGTACAGATTTTA

TGAAACCATGTGCTATGGCTCGAATCCATAGTCAATCCTACTTTCGATAGGACCGGTTGA

CAATTGAATCCAATTTTTCCCATTATTTGACTATCCATAATAGTGCGGAAAGAAAGCCCG

GAGGAAGAGTGGCCTTGAGTTTCTCGCCCCTTTGCCTTAGGATTCGTTAATTCTCTTTCT

CGATGGGACGGGGAAGGGATATAACTCAGCGGTAGAGTGTCACCTTGACGTGGTGGAAGT

CATCAGTTCGAGCCTGATTATCCCTAAACCCAATGTGAGTTTTTTCTATTTTGACTTACT

CCCCCGCCACGATCGAACGGGAATGTATAAGAGGCTTGTGGGATTGACGTGATAGGGTAG

GGTTGGCTATACTGCTGGTGGCGAACTCCAGGCTAATAATCTGAAGCGCATGGATACAAG

TTATCCTTGGAAGGAAAGACAATTCCGAATCCGCTTTGTCTACGAATAAGGAAGCTATAA

GTAATGCAACTATGAATCTCATGGAGAGTTCGATCCTGGCTCAGGATGAACGCTGGCGGC

ATGCTTAACACATGCAAGTCGAACGGGAAGTGGTGTTTCCAGTGGCGAACGGGTGAGTAA

CGCGTAAGAACCTGCCCTTGGGAGGGGAACAACAACTGGAAACGGTTGCTAATACCCCGT

AGGCTGAGGAGCAAAAGGAGAAATCCGCCCAAGGAGGGGCTCGCGTCTGATTAGCTAGTT

GGTGAGGCAATAGCTTACCAAGGCGATGATCAGTAGCTGGTCCGAGAGGATGATCAGCCA

CACTGGGACTGAGACACGGCCCAGACTCCTACGGGAGGCAGCAGTGGGGAATTTTCCGCA

ATGGGCGAAAGCCTGACGGAGCAATGCCGCGTGGAGGTGGAAGGCCTACGGGTCGTCAAC

TTCTTTTCTCGGAGAAGAAACAATGACGGTATCTGAGGAATAAGCATCGGCTAACTCTGT

GCCAGCAGCCGCGGTAAGACAGAGGATGCAAGCGTTATCCGGAATGATTGGGCGTAAAGC

GTCTGTAGGTGGCTTTTCAAGTCCGCCGTCAAATCCCAGGGCTCAACCCTGGACAGGCGG

TGGAAACTACCAAGCTGGAGTACGGTAGGGGCAGAGGGAATTTCCGGTGGAGCGGTGAAA

TGCATTGAGATCGGAAAGAACACCAACGGCGAAAGCACTCTGCTGGGCCGACACTGACAC

TGAGAGACGAAAGCTAGGGGAGCAAATGGGATTAGAGACCCCAGTAGTCCTAGCCGTAAA

CGATGGATACTAGGTGCTGTGCGACTCGACCCGTGCAGTGCTGTAGCTAACGCGTTAAGT

ATCCCGCCTGGGGAGTACGTTCGCAAGAATGAAACTCAAAGGAATTGACGGGGGCCCGCA

CAAGCGGTGGAGCATGTGGTTTAATTCGATGCAAAGCGAAGAACCTTACCAGGGCTTGAC

ATGCCGCGAATCCTCTTGAAAGAGAGGGGTGCCCTCGGGAACGCGGACACAGGTGGTGCA

TGGCTGTCGTCAGCTCGTGCCGTAAGGTGTTGGGTTAAGTCTCGCAACGAGCGCAACCCT

CGTGTTTAGTTGCCACTATGAGTTTGGAACCCTGAACAGACCGCCGGTGTTAAGCCGGAG

GAAGGAGAGGATGAGGCCAAGTCATCATGCCCCTTATGCCCTGGGCGACACACGTGCTAC

AATGGGCGGGACAAAGGGTCGCGATCTCGCGAGGGTGAGCTAACTCCAAAAACCCGTCCT

CAGTTCGGATTGCAGGCTGCAACTCGCCTGCATGAAGCAGGAATCGCTAGTAATCGCCGG

TCAGCCATACGGCGGTGAATCCGTTCCCGGGCCTTGTACACACCGCCCGTCACACTATAG

GAGCTGGCCATGTTTGAAGTCATTACCCTTAACCGTAAGGAGGGGGATGCCTAAGGCTAG

GCTTGCGACTGGAGTGAAGTCGTAACAAGGTAGCCGTACTGGAAGGTGCGGCTGGATCAC

CTCCTTTTCAGGGAGAGCTAATGCTTATGCTTATTGGGTATTTTGGTTTGACACTGCTTC

ACGCCCAAAAAGAAGGCAGCTACGTCTGAGCTAAACTTGGATATGGAAGTCTTCTTTCGT

TTAGGGTGAAGTAAGACCAAGCTCATGAGCTTATTATCCTAGGTCGGAACAAATTAGTTG

ATAGTGATAGGATCCCCTTTTTGACGTCCCCATGTCCCCCCGTATGGCGGCATGGGGATG

TCAAAAGGAAAGGGATGGAGTTTTTCTCGCTTTTGGCGTAGCGGCCTCCCTTTGGGAGGC

CGCGCGACGGGCTATTAGCTCAGTGGTAGAGCGCGCCCCTGATAATTCGTCGTTGTGCCT

GGGCTGTGAGGGCTCTCAGCCACATGGATAGTTCAATGTGCTCATCAGCGCCTGACCCGA

AGATGTGGATCATCCAAGGCACATTAGCATGGCGTACTCCTCCTGTTTGAATCGGAGTTT

GAAACCAAACAAACTTCTCCTCAGGAGGATAGATGGGGCGATTCAGGTGAGATCCCATGT

AGATCTAACTTTCTATTCACTCGTGGGATCCGGGCGGTCCGGGGGGGGCACCACGGCTCC

TCTCTTCTCGAGAATCCATACATCCCTTATCAGTGTATGGAGAGCTATCTCTCGAGCACA

GGTTGAGGTTCGTCCTCAATGGGAAAATGGAGCACCTAACAACGCATCTTCACAGACCAA

GAACTACGAGATCACCCTTTCATTCTGGGGTGACGGAGGGATCGTACCATTCGAGCCTTT

TTTTCATGCTTTTCCCGGCGGTCTGGAGAAAGCAGCAATCAATAGGACTTCCCTAATCCT

CCCTTCCTGAAAGAAGAACGTGAAATTCTTTTTCCTTTCCGCAGGGACCAGGAGATTGGA

TCTAGCCATAAGAGGAATGCTTGGTATAAATAAGCCGCTTATTGGTCTTCGACCCCCTAT

ACGAGCGCCCCCGATCAGTGCAATGGGATGTGGCTATTTATCTATCTCTTGACTCGAAAT

GGGAGCAGAGCAGGTTTGAAAAAGGATCTTAGAGTGTCTAGGGTTGGGCCAGGAGGGTCT

CTTAACGCCTTCCTTTTTCTGCCCATCAGAGTTATTTCCCAAGGACTTGCCATGGTAAGG

GGGAGAAGGGGAAGAAGCACACTTGAAGAGCGCAGTACAACGGGGAGTTGTATGCTGCGT

TCGGGAAGGATGAATCGCTCCCGAAAAGGAGTCTATTGATTCTCTCCCAATTGGTTGGAT

CGTAGGGGCGATGATTTACTTCACGGGCGAGGTCTCTGGTTCAAGTCCAGGATGGCCCAG

CTGCGCAGGGAAAAGAATAGAAGAAGCATCTGACTCTTTCATGCATACTCCACTTGGCTC

GGGGGGATATAGCTCAGTTGGTAGAGCTCCGCTCTTGCAATTGGGTCGTTGCGATTACGG

GTTGGCTGTCTAATTGTCCAGGCGGTAATGATAGTATCTTGTACCTGAACCGGTGGCTCA

CTTTTTCTAAGTAATGGGGAAGAGGACTGAAACATGCCACTGAAAGACTCTACTGAGACA

AAAAGATGGGCTGTCAAAAAGGTAGAGGAGGTAGGATGGGCAGTTGGTCAGATCTAGTAT

GGATCGTACATGGACGATAGTTGGAGTCGGCGGCTCTCCTAGGCTTCCCTCATCTGGGAT

CCCTGGGGAAGAGGATCAAGTTGGCCCTTGCGAATAGCTTGATGCACTATCTCCCTTCAA

CCCTTTGAGCGAAATGTGGCAAAAGGAAGGAAAATCCATGGACCGACCCCATTGTCTCCA

CCCCGTAGGAACTACGAGATCACCCCAAGGACGCCTTCGGCGGGGGTCACGGACCGACCA

TAGACCCTGTTCAATAAGTGGAACACATTAGCCGTCCGCTCTCCGGTTGGGCAGTAAGGG

TCGGAGAAGGGCAATCACTCGTTCTTAAAACCAGCATTCTTAAGTTAAGATCAAAGAGTC

GGGCGGAAAAAGGGGAGAGCTCCCCGTTCCTGGTTCTCCTGTAGCTGGATTCCCCGGAAC

CACAAGAATCCTTAGAATGGGATTCCAACTCAGCACCTTTTGTTTTGAGATTTTGAGAAG

AGTTGCTCTTTGGAGAGCACAGTACGATGAAAGTTGTAAGCTGTGTTCGGGGGGGAGTTA

TTGTCTATCGTTGGCCTCTATGGTAGAACCCGTCGGGGAGGCCTGAGAGGCGGTGGTTTA

CCCTGTGGCGGATGTCAGCGGTTCGAGTCCGCTTATCTCCAGCCCGTGAACTTAGCGGAT

ACTATGATAGCACCGAATTTTGCCAATTCGGCAGTTCGATCTATGATTTCGCATTCATGG

ACGTTGATAAGATCCTTCCATTTAGTAGCACCTTAGGATGGCATAGCCTTAACGTTAATG

GCGAGGTTCAAAAGAGGAAAGGCTTGCGGTGGATACCTAGGCACCCAGAGACGAGGAAGG

GCGTAGCAAGCGACGAAATGCTTCGGGGAGTTGAAAATAAGCATAGATCCGGAGATTCCC

AAATAGGTCAACCTTTTAAACTGCCTGCTGAATCCATGAGCAGGCAAGAGACAACCTGGC

GAACTGAAACATCTTAGTAGCCAGAGGAAAAGAAAGCAAAAGCGATTCCCGTAGTAGCGG

CGAGCGAAATGGGAGCAGCCTAAACCGTGAAAACGGGGTTGTGGGAGAGCAATACAAGCG

TTGTGCTGCTAGGCGAAGCGGTTGAGTGCCGCACCCTAGATGGCTAAAGTCCAGTAGCCG

AAAGCATCACTGCTTACGCTCTGACCCGAGTAGCATGGGGCACGTGGAATCCCGTGTGAA

TCAGCAAGGACCACCTTGCAAGGCTAAATACTCCTGGGTGACCGATAGCGAAGTAGTACC

GTGAGGGAAAGGTGAAAAGAACCCCCAGTGGGTAGTGAAATAGAACGTGAAACCGTGCTG

AGCTCCCAAGCAGTGGGAGGGGAAAGTGATCTCTGACCGCGTGCCTGTTGAAGAATGAGC

CGGCGACTCATAGGCAGTGGCTTGGTTAAGGGAACGGAACCCACCGGAGCCGTAGCGAAA

GCGAGTCTTCATAGGGCGATTGTCACTGCTTATGGACCCGAACCTGGGTGATCTATCCAT

GACCAGGATGAAGCTTGGATGAAACTAAGCAGAGGTCCGAACCGACTGATGTTGAAGAAT

CAGCGGATGAGTTGTGGTTAGGGGTGAAATGCCACTCGAACCCAGAGCTAGCTGGTTCTC

CCCGAAATGCGTTGAGGCGCAGCAGTTGACTGGACATCTAGGGGTAAAGCACTGTTTCGG

TGCGGCTGCGCGAGCGGTACCAAATCGAGGCAAACTCTGAATACTAGATATGACCCAAAA

ATAACAGGGGTCAAGGTCGGCCAGTGAGACGATGGGGGATAAGCTTCATCGTCGAGAGGG

AAACAGCCCGGATCACCAGCTAAGGCCCCTAAATGACCGCTCAGTGATAAAGGAGGTGGG

GGTGCAAAGACAGCCAGGAGGTTTGCCTAGAAGCAGCCACCCTTTAAAGAGTGCGTAATA

GCTCACTGATCGGCGCCCTTGCGCTGAAGATGAACGGGGCTAAGCGATCTGCCGAAGCTG

TGGGATGTCAAAATGCATCGGTAGGGGAGCGTTCCGCCTTAGATGGAAGCAAACGCGAAA

GCGGGGGTCGACGAAGCGGAAGCGAGAATGTCGGCTTGAGTAACGAAAACATTGGTGAGA

ATCCAATGCCCCGAAAACCCAAGGTTTCCTCCGCAAGGTTCGTCCACGGAGGGTGAGTCA

GGGCCTAAGATCAGGCCGAAAGGCGTAGTCGATGGACAACAGGTCAATATTCCTGTACTA

CCCCTTGTTGGTACGGAGGGACGGAGGAGGCTAGGTTAGCCGAAAGATGGTTATAGGTTT

AAGGACACAAGGTGACCCTGCTTTTTCAGGGTAAGAAGGGGTAGAGAAAATGCCTCGAGC

CGAGGTCCGAGTACCAAGCGCTGCAGCGCTGAAGTATGAGCCCCGTGGACTAGCCATTGC

TTCTCCACGAGCCTCATACCAGGCGCTACGGCGCGAAGTATGTAACCCATGCCATACTCC

CAGGAAAAGCTCGAACGACCTTCAACAAAAGGGTACCTGTACCCGAAACCGACACAGGTG

GGTAGGTAGAGAATACCTAGGGGCGCGAGACAACTCTCTCTAAGGAACTCGGCAAAATAG

CCCCGTAACTTCGGGAGAAGGGGTGCCCCCTCGCAAAAGGGGGTCGCAGTGACCAGGCCC

GGGCGACTGTTTACCAAAAACACAGGTCTCCGCAAAGTCGTAAGACCATGTATGGGGGCT

GACGCCTGCCCAGTGCCGGAAGGTCAAGGAAGTTGGTGAACTGATGACAGGGAAGCCGGC

GACCGAAGCCCCGGTGAACGGCGGCCGTAACTATAACGGTCCTAAGGTAGCGAAATTCCT

TGTCGGGTAAGTTCCGACCCGCACGAAAGGCGTAACGATCTGGGCACTGTCTCGGAGAGA

GGCTCGGTGAAATAGACATGTCTGTGAAGATGCGGACTACCTGCACCTGGACAGAAAGAC

CCTATGAAGCTTTACTGTTCCCTGGGATTGGCTTTGGGCCTTTCCTGCGCAGCTTAGGTG

GAAGGCGAAGAAGGCCCCCTTCCGGGGGGGCCGAGCCATCAGTGAGATACCACTCTGGAA

GAGCTCGGATTCTAACCTTGTGTCAGACCCGCGGGCCAAGGGACAGTCTCAGGTAGACAG

TTTCTATGGGGCGTAGGCCTCCCAAAAGGTAACGGAGGCGTGCAAAGGTTTCCTCGGGCC

AGACGGACATTGGTCCTCGAGTGCAAAGGCAGAAGGGAGCTTGACTGCAAGACTCACCCG

TCGAGCAGAGACGAAAGTCGGCCTTAGTGATCCGACGGTGCCGAGTGGAAGGGCCGTCGC

TCAACGGATAAAAGTTACTCTAGGGATAACAGGCTGATCTTCCCCAAGAGTCCACATCGA

CGGGAAGGTTTGGCACCTCGATGTCGGCTCTTCGCCACCTGGAGCTGTAGGTGGTTCCAA

GGGTTGGGCTGTTCGCCCATTAATGCGGTACGTGAGCTGGGTTCAGAACGTCGTGAGACG

TTCGGTCCATATCCGGTGTGGGCGTTAGAGCATTGAGAGGACCTTTCCCTAGTACGAGAG

GACCGGGAAGGACGCACCTCTGGTGTACCAGTTATCGTGCCTACGGTAAACGCTGGGTAG

CCAAGTGCGGAGAGGATAACTGCTGAAAGCATATAAGTAGTAAGCCCACCCCAAGATGAG

TGCTCTCTCCTCCGACTTCCCTAGAGCCTCCGGTAGCACAGCCGAGACAGCGACGGGTTC

TCCACCCATACGGGGATGGAGCGACAGAAGTATGGAAATAGGATAAGGTAGCGGCGAGAC

GAGCCGTTTAAATAGGTGTCAAGTGGAAGTGCAGTGATGTATGCAGCTGAGGCATCCTAA

CGAACGAACGATTTGAACCTTGTTCCTACACGACCTGATCAAATTGATCAGGCACTTGCC

ATCTATCTTCATTGTTCAACTCTTTGATGAAAAGATGAAAAACCAAAAAAAGCTCTGCCC

TTCCATCTCTTGGATAGATAGAGAGGGAGGGCAGAGGCCTTTGGTGTCCCTTCCAGTCAA

GAATTGGGGCTTCACAATTACTAGCCAATATTTATCTCATGCCTTTCCTCGTTCATGGTT

CGATATTCTGGTGTCCTAGGCGTAGAGGAACCACACCAATCCATCCCGAATTTGGTGGTT

AAACTCTACTGCGGTGACGATACTGTAGGGGAGGTCCTGCGGCAAAATAGCTCGATGCCA

GAATGATAAAAAGCTTAACACCTCTTATTTGACTTTTTCACTATTTTGAAATACGAAAAA

GATCCAAATCCAAAATGCAAAGGTCGTCTTATTCAAAACCTCAATCATCCCCTCCTCCCA

CTTCACACCTCGGAACGCACTGTTCTTATAGAGAGAAAGGCGCTTTCCCATCTTCTTAAC

CCGAAATGAAATGGCTGAGGAGAGGAAGGTTCCTTTTGGGGGGTACCCCCGGGAAGAGAT

CCAGTGGAGACGGGGTGGGCCTGTAGCTCAGAGGATTAGAGCACGTGGCTACGAACCACG

GTGTCGGGGGTTCGAATCCCTCCTCGCCCACAGCCTTCCAAAGGGGAAGGGCCTTTACTT

TCCCCCTGAGGGTAGGAAAATCATGATCGGGATAGCGGACGTAAAGCTATTGAACTTGGG

TATGCTCTTTCCTTTTGTCGAAGTGGAATCGTAGAACAGAATGTGATACGATGAGATAGA

ATGCATAGAAACAAGGATAGCGAACGGGTTACCTACTCCTAAGGGTCAAAGCAAGCCCTT

TAATTCAATTCTTTATTCTTACATTAAAGAATGAATCAAATCTCCCCAAGTAGGATTCGA

ACCTACGACCAGTCAGTTAACAGCCGACCGCTCTACCACTGAGCTACTGAGGAACAAGGG

GGATTCGACCTCCTAGAGTTCAACTCCCGCTCTCAACCCATGAACAATATGAGTCCGAAG

CTTCTTTCGTAACTCCCGGAATTTCTTCGTAGTGACTCCGTTCCATGCCTCATTTCATAG

GGAAGCCCAAAGTGGCTCTATTTCATTCTATTTCACTTCCTAGCACTTCCTATCATTTAA

TATCCATCCCTTTGGTCTTATTTACATAAGAGATGTCATTTATAGTCTATCTCTTTCTAT

ATATGGAAAGTCAAGAAATTCTCATCGAAACATCGAGAAATTGTGCATATAGAAAACTCT

AAAGAAAGAAAAAAGGAGACCCATGCCATGATTTTCAAATCTTTTCTACTTAGTAGTCTA

AGTTTCTCGATGAGGATAATTAATTCGGTCGTTGTGGTCGGACTCTATTATGGATTTCTG

ACCACATTCTCCATAGGTCCCTCTTAGATCTTCTTTCTCCAATCTTGGATTAGGGAAGAA

GGAGATATTCGCGACTACTGGCGGTTTCATTATGGGGCAGCTCATGATCTTCATATCGAT

CTATTATCCACCTCTGCATCTATTCTTTCTTAGCTAAACGGGTGGAAGATCCATCCAATT

TGGTTATATCATGGACTCAAAAGCGGATCTGAATGTGACTGAAATGCACGATCTTCACAG

GTATCACTTTTCACGATACCTAAAGATGGAATAGCGATTTGAACCATTTCCTATACGAGA

ATGGTTTCCATTACTTTGAGAAATGGATTCTATATCAAACTATAGCTATTGCATTAAAGA

AGAAAAGAAACTAATAGAAGTCGAAGACGCGGAATGGTAGTGAATAGAGAGAAAGATTCT

TCTGATTTTCTTGTTCCTGAAAATATTCTATCTATCTCCTAGACGCCGTAGAGAATTGAG

AATTTTCATGTCTTTCAATTCTCGTACTCGTAATTGGAAAGTTACGGAAGGAGGTCCATC

ATTTTGCAATGAAAACAACATAAAAAACTCTGGACAATTTCGAAATCAGGCCAAGCGTCT

TAATACATATGCAAAAAAATTCATTATTGGCCCACCATTGATTAGAAGATTTAGCTTGTA

TGAATCGCTATTGGTTTGATACGAATAATGGCAGTCGTTTCAGTATGTTAAGGATACAGA

TGTATCCACAATTCATTTAGAGTTACTTAATAGCCTATTTCTTATACCATATCTCTATCC

CGTGAAATTCTCGAGCCGAAAGATGGATGCATATGCTGTGTTTCATTTTGCTAAACGATA

TCAATTAAATGGTGTATCAATTCCATAAATTGGATATAGCAATAAATAAATCAGCAAAAT

TCTTTTATTTTAGATAGAAGAAATGTTTCTTCTATCTAAAATAAAAGAATGTACCCTTCT

ATCCAAATCCAATTTGCATCGATAAAATAAATCCAAATTCCAGTAGTGGATGAATAATTG

CAAATTTTTGTGTGTACGAGATTAGAATAACTTCAAAATAACTGACATAATTTTTTATTT

TTCCTGATCAGAAAAATACATGAAAAAGAAAGGAGGTAGAAAAATTTTGGGATTTATGGT

TAAAGAAGAAAAAGAAGAAAACAGGGGTTCTGTTGAATTTCAAGTATTCAGTTTCACCAA

TAAGATACGGAGACTTGCTTCACATTTGGAATTACACAAAAAAGATTTTTCATCGGAAAG

AGGTCTACGAAGACTTTTGGGAAAACGTCAACGTTTGCTGGCTTATTTGGCAAAGAAAAA

TAGAGTACGTTATAAGAAATTAATCAGTCAGTTGGATATTCGGGAGAAGTAATTTAATCG

TTCGAATTTTTTTCTTATTTTATTAGTAGTCTTATAGTAGTCTTAGATTTTTCATTTTGA

TGAGCCTCGTTTTGAGGAATTCATGGAATAATCCATTTTCATGGAATAAAGAATAAGAAC

ACGGATACATAACATAAAAAAAAGAATAAATAAGACGATATTCGCCCTCCCCCTACATAT

TTAATTTCTTCTCCTATACAAAAACCAGCAAGACCTACTCCATTGGTAATTCCATCAATG

ACACCCTTATCGAAAAACTGCGTTAGTTCAGTTAATCCTCTTATACCCAGGGTAAAGACC

CTAGTATAGAAAATATCTATATAACCACGATTATATGACCAACTGTATATCTTTTTTTTT

ACTTGATCCGAAAAAAACTTTTTCGGACCCTCTTTTACAAGAGAATTTATTAAATCCAAA

TTCTGAAAAAAGGAATAAGCGGATCCATAGAAGATATATGCTATGGATAGACCAAACATA

GCTAGACTTACAGAAGAAATTGCATTAGTGATAAATTCATATGAATTTATGGAAGAATTA

GAACTTTCCTGGAAAAAGTTTATTGAGGGAGTTAACCACTTTGATAATATGGTTAACTCC

GCTATTTCATTATCAAAATGGATTCCTATGGATCCAATGAACAAAGTAAAAAGCAGTAAT

ATAAAAAGAGGGAATAGCATAGTATTTCCCGTTTCATGAGGATAGACAAAAGTGTTTTTA

GCCCCAAAGGAAGTACTAAAGGACCCTATCCTATTTCTTGTATTACCATGAATTTTGGAT

CTATTTTGTGAAAAAAAAGAAACTCCACTCTTCGTTGTTGATAAAATGAAATCTCTATTC

ACTCCTTTGGGTATCCTTTTTCCCCATAAGGATATTGAATACAACGAACCCTCTTTAGTG

CTACTGTAATTTTGAAAATGAACACGCAGGTACCCATCAAAAGTAAGTAAATATATCCGA

AACATATAAAACGCAGTTAATCCTGCAGTAAAAGAGGCTATTATTCCAAAAAAGGGTGAA

TACAACCAACTATTACTAAGGATTTCATCTTTGGACCAGAAGCAAGCAAGAGGTGGAATA

CCACAAAGAGAAAGCGTACCCCATAAAAAAGTAGTTCTTGTAATTGGAACGTATTTTCTT

AAACCACCCATAAGAACCATATTCTGACTTTTATCTGGTGAATATCCAACAAGAGGTTCC

ATTGAATGAATAATGGATCCGGATCCCAAGAACAATAAAGCTTTCGAATAAGCATGAGTG

ATCAAATGGAATAAAGCAGCTTGATAAGAACCTATACCTAGAGCTAACATCATATAACCC

AATTGAGACATTGTAGAATAGGCTAAGCTTCTTTTAATATCTCTCTGAGCAAGAGCTAAA

GTAGCTCCTAAGAAGAGTGTTATTGTACCTACTAAAGAAATGAAACTCATTATTAAAGGT

AGGGATATGAAAAGAGGAAGAAGTCGAGCTAGAAGAAAAATCCCCGCAGCAACCATAGTT

GCTGCGTGTATAAGAGCCGAAATGGGAGTGGGTCCTTCCATAGCATCGGGTAACCATACG

TGAAGAGGGAATTGTGCAGATTTCGCAACTGCACCAAGGAATAATAAAAAAGCACACAAA

GTAGTAAGTAAGGAATTAATCCCATTATTAGGAATCCAGTTATTAGCTATTTTGAACAAA

TCCCTAAACTCTAAACTACCTGTTATCCAAAAAAAACCTAAAATTCCTAATAACAGACCA

AAATCCCCTACACGATTAGTTACAAAAGCTTTTTGACAAGCACTCGCTGCAATTGGCCGT

GTAAACCAAAAGCCTATCAATAAATAGGAACACATTCCCACAAGTTCCCAAAAAAAATAA

ATTTGTATCAAATTGGAACTAGTAACCAATCCCAACATGGAAGTATTGAAAAAACTTATA

TAAACAAAAAATCTCAAATATCCTTCATCGTGAGACATATAATCGTCACTATAAATAAGA

ACGAGGATTCCTACAGTAGTAATTAGTATTAACATAATAGAAGTAAGCGGGTCGATCAAG

TATCCAAATTCTAAGGAAAAATCATTATTGACGGTCCAAGACCATAGATATTGATAGATA

GAACTTCCATTTATTTGTTGAATAGATAGGTGAACTGAGAATACCATAGCTATACTTAAG

AGTAAAACACAAGGAAAAGCCCATATGCGACGAAGATTTTTTGTTGCTGTCGGAATAAGA

ATAAGTCCAAACCCCATTGACATAATAACTGGAAGTGGGAGAAGAGGGATTACCCATGCA

TATTGATATGTATGTTCCATAAGAAAAGAAATTGCAATTTTTCTATAAAATAAAATTGTT

TCCGATTCACCAAACCAATTCTTATCTCTTTCTGAAGGAATTCCAAAATACTGGAATTCT

TCATTTTTCAAATTTCTCTCATTGAAATATCAAAAATGAAGAATGGGTTTACTTGGTTAA

ATTCAAAAAGTTAATTAAATAACTTTGTTACCTAGTTATTACTAAAGAAGGATATTTGTT

AAAATACAAAAAAGGATTGAATCATTTTACTTTTGTATTTCTTTCTATTAAAATGAAGCA

GCTCTCATGTTTCGTAACTGAAATTGATTGGAATTCCTTAATTAACTATTTGAATTTTCC

CTTCCTTTTATCCCCCATCTTATATGGGGGATAGGCCGTAGATCTATATATGGAGTATAC

TTAATATTAATTTAAATAGAAAACAAAAAATTCTTGTCTTATCCGCATTAGAGAAAATCA

AGTAAAAAAGAATTCAGAATTTCAGTTCAGTATCTAGTATAAATACTAAGAAAAAACAGA

AAGAAGGATTGATTTGCGGCAATAGATGTCTTTCACATACAACTAGAAAAAGTAATCTCC

TTTTTGAATGGCAGTTCCAAAAAAACGTACTTCGATGTCAAAAAAGCGTATTCGTAAAAA

TCTTTGGAAGAAAAAGACTTATTTTTCCATAGTACAATCTTATTCTTTAGCAAAATCAAG

ATCATTTTCCAGCGGTAGCGAGCATCCAAAACCAAAGGGTTTTTCTGGGCAACAAACAAA

TAATCTGGTTTTGGAATAATCTGAATTGACCTATCCCAAAGAAATTCCAATTATTTAAAA

TGAATAATTCGGATTAATTAATGAATGTACTTTTATGTGTCGAATTCCTCGGTACAATAT

TCTTAGAACTAACCCCTCTGATATATAGAACAAAAGTTTTTGGTATACTGTGTCCTAAGT

ATTCTTTTCCTATCAACGAACTTTTCATAATAGAATCCTCAAATATGAGGATTCTATTAT

GAAAAGTAGAATATTCTTGCAATAGGACTTACAACTTCTACCTATCTTATCAAAAATCCA

TTGCAACTGAAAAAAAATCCCCAATGATGAAATTCTAATGTCCTAAATTCTATGGACTCT

CCCAATCTCGACGATTTGCGAGAAAATAACTATTATTCTTTTAACTTCCCTATTATTTAA

AGTTAGCCGCCATGGTGAAATTGGTAGACACGCTGCTCTTAGGAAGCAGTGCTCAAGCAT

CTCGGTTCGAGTCCGAGTGGCGGCATTCTCGAAAAAGAATACAATAGATTAGAAATGATT

CAATTCGAAATTTCCAATTTTGTAATGGGACCTTCTCCTTATGCTATTTGCAACTTTAGA

ACATATACTAACTCATATCTCTTTCTCAACCATTTCAATTGTGATTACAATTCATTTGAT

AACCTTATTAGTTCGTGAACTTGGGGGATTACGTGATTCGTCAGAAAAAGGAATGATAGC

TACTTTTTTCTCTATAACAGGATTCTTAGTTTCTCGTTGGGCTTCTTCGGGACATTTTCC

ATTAAGTAATTTATATGAGTCATTGATCTTCCTTTCATGGGCTCTGTATATTCTTCATAC

GATTCCTAAGATACAGAACTCTAAAAATGATTTAAGCACAATAACTACGCCAAGTACTAT

TTTAACGCAAGGCTTTGCCACGTCGGGTCTTTTAACTGAAATGCATCAATCCACAATACT

AGTACCTGCTCTACAATCTCAGTGGTTAATGATGCATGTCAGTATGATGTTACTAAGCTA

TGCAACTCTTTTGTGCGGATCCTTATTATCCGCCGCTCTTCTAATCATTAAATTTCGAAA

GAATTTCAATTTCTTTTTAGAAAAGAAAAATGTTTTAAATAAAACATTTTTCTTTAGTGA

GATTGAATATTTCTATGTAAAAAGAAGTGCTTTAAAAAACACCTCTTTTCCTTCATTTCC

AAATTATTACAAATATCAATTAATTGAGCGTTTGGATTCTTGGAGTTATCGTGTCATTAG

CCTAGGGTTTACCCTTTTAACCATAGGTATTCTTTGTGGAGCAGTATGGGCTAATGAGGC

GTGGGGATCCTATTGGAATTGGGATCCTAAGGAAACTTGGGCATTTATTACTTGGACCAT

ATTCGCAATTTATTTACATAGTAGAACAAATCCAAATTGGAAGGGTACGAATTCCGCACT

TGTAGCTTCGATAGGATTTCTTATAATTTGGATCTGCTATTTTGGTATCAATCTATTAGG

AATAGGTTTACATAGTTATGGTGCATTTACATTACCATCTAAATGATTACATAACATAAA

ACCTTCGAGTTTCCATTTTTGTTTGATTTGAGAACCCTTGAACGCCTTCTCAAAGGGTTC

TCAAAAATTCGAGATAGATCTAATTAGACTTTTTTACTTTTTTCTGAATTTTTCACTATG

GAATATAGAGCGGACTAGTAAAAAAAAATTATTTAGGATAATAATTGGATAAGAGAGCCT

CTACCTTGTCAACCGATAGCGAGAGAACAAAATCTGGATAAATACCAATTCCTATTACTG

GTAAAAAGATACAGATTAAAAGAAAGAGTTCTCGTGGTCCAGAATCCACCAAATTTGCGT

TTGGAACATGAAATAGCTTGTATCCATAGAACATCTGGCGTAACATAGATAATAAAAAAA

TAGGAGTTAATATCATTCCAATTGCCATTACAAAAGTAATTAGCATTTTTGGTATTAACA

GAAATTTTGGACTAGTAATTAGTCCAAAAAATACTACTAATTCTGCAACAAAACCGCTCA

TTCCTGGTAAGGCAAGAGAAGCCATTGAAAAGCTACTAAACATGGTAAAAATTTTCGGCA

TTGGGATAGATATCCCCCCCAGTTCTTCGAGATAAACAAGACGCATTCTATCACAAGCCG

TTCCCGCCAAGAAAAAAAGTGTAGCACCAATAAATCCATGGGATAGTATTTGTAAAATAG

CTCCATTGAGTCCAATGTTGGTTATGGAACCAATTCCTATAATTATGAAACCCATGTGAG

ATACGGAGGAGTAGGCTATTCTTTTTTTGAAATTTCGTTGACCAAGAGAAGTTGAAGCTG

CATAGATTATTTGCATCGCTCCTATTATTACCAACCAGGGGGAAAATAGATAATGAGCAT

GAGGTAACAATTCCATATTGATCCGAATCAATCCGTATGCTCCCATCTTTAATAGGATTC

CCGCTAAAAGCATACATGTACTGTAATGCGCTTCCCCATGGGTATCTGGTAACCACGTAT

GTAGGGGTATAATCGGCAATTTGACAGCATAAGCAATAAGGAAGCCAAAATAAAATAGTA

TTTCCAATGTTGCAGGGTATGATCGATTAATTAATCTTTCCAAATCTAATCTTGGTTCGT

TGGAACCATATAAGCCCATACCTAGAACTCCGATTAAGAAAAAAATGGAACCGCCTGCAG

TATACAAAATAAATTTTGTAGCTGAATACAGACGCCTCTTTCCCCCCCACATGGATAAAA

GTAAGTAAACAGGAATTAATTCTAACTCCCACATGATAAAAAAAAGTAAAAGGTCTCGCG

AAGAAAATAATCCTATTTGACCACTATACATTGCTAGCATCAGGAAATAGAATAATCGCG

AATTCCGGGTAACTGGCCAAGCTGCTAAAGTAGCTAAAGTAGTGATAAATCCTGTCAATA

AAATAGATCCTAATGAAAGTCCATCGATTCCCAATCTCCAGTGGAAATCAAAGACATCTA

TCCATTTAGAATCCTCCTTTAATTGGATTAAGGGATCCTCCAATTGGAAATGATAACAGA

ATGCATAAGTCATTAGAAGGAATTCTAATAAACAAATAGATATAGTATACCACCTAACGA

TTTTGTTTCCCCTATGAGGTAAAAAGAAAATTAATGAACCTGCAAATATCGGCAAAACAA

CAAGTATTGTTAACCAAGGAAAATAACTCATGATAAAGTGATAAAGAGAAGATACGTTTT

GACCAGAAAAGCCCGTGCTCGAAATAAGCGAGCACAGGCTTCCTCGGTAAAGAGGAATCA

GACGATTCAAGTGGAGTTTTTTGTAACGTATCAATAAGATAGAGCCATGCTGCGGGTTGT

TTCAGGCCCTAAATAAACGCGGACACTTAAAAAATCTGTTGGGCAGGCAGATTCACATCT

CTTACAACCCACACAATCTTCGGTTCTCGGCGCGGAAGCAATTTGCTTGGCTTTACACCC

ATCCCAGGGTATCATTTCTAATACATCTGTTGGACAAGCTCGTACACATTGAGTGCATCC

TATACATGTATCATAAATTTTTACGGAATGTGACATTGGATCTATAAATTTTCCTTTTCA

ACATAAAAATTTTCGATCTGGTAAAAATGAAATTAGTACTATATGAGTCATATGTATTGT

AGACACCAGACGAAGCAATGGTTTATCCAAACTTCAACAAATAATGCAATATATTTCTTA

ATCCGTTTGTGAGAAAGCGTGAAAAGAGCCAAGAGACTTGAATTTTTGGCTTCAACAATC

ATAATTATACGAATTGTACATACGAATTCGAATTAGCCAATTTATTGGCTATCGTCTTTT

CAATATAAATTATTGCAATATTCAAATTGCAATATCAATGAATTGCAAAAATTCAATAAG

TAAAAAGAATACTATGTAATAACCTAATCAAAAAATAGATATTATAAAATAATAAATAGT

ATTATTAATATTTGTTATTATTATATGTGCGCCTTTGTTTAGAGGATTTTATGTCTAATT

ATTCAAAAAATTAGATTGATTGATACGAGTTGATTTCTTGTTACGATGGATGGAAGAAAG

AATGGATAGTCCAATAGCTGCTTCAGCAGCCGCAAGGGCTATAACAAAAATTGCGAAAAT

GTCTCCTTTTAATTGGCGACTATCAAATAGATCAGAAAATGTTACGAGATTTAGATTAAT

TGAATTCAGTATAAGTTCAAGACATATTAGAGCTCTAACCATGTTTCGGCTTGTGATCAA

TCCATAGATACCAATCGAAAATAAATAGACACTAAAAAAAAGTACATGCTCAAACATCAT

TAACTAACTCCTTATCAATCTCGATTCATTTCAATATGAGGACAAGAATTGAACCGATTC

CATTAATTAGAATAGAACAGTTACACAACAAAAGAGAAAAGAAGGTATTTGTTGGCAGTA

GATGGGTTTTACTAAATCAAAATTGTGATTCTTTAGTTATTTATTTTAGATTTGAAATTC

TAAGAATTTGACTAATTCTAAGTATTTCTTATTGCCGAGCCATAGTAATTGCACCTATTA

AAGAAACTAGAAGAATTATGGAAATGAGTTCAAACGGAAGATAAAAATCAGTTGCTAAAT

GAATCCCAATTTGTTGAACGTTATTTATGAGACCCTGTTCTACTATTTGGTTTGATCTTG

TAGTCCAAAGAATTCCATACCATGACGTATCTGGGATAGTAGTCATTAGTGAAAAAAGAA

TAGTTATACAAACGAGTGAAGTGAACCCATCTCCAATAGTCCAATAATTCTTATTTTTAG

ACCATTCTGAGCCATTTACGAACATTACGGCAAATATGATCAAAACATTTATAGCTCCCA

CATAAATAAGAAGTTGTGCGACAGCTACAAAGTAGGAATTCAATAAAATATAGAATAAGG

ATATACAAACAAGAACTAATCCCAGCGAAAAGGCAGAATAAATTGGGTTGGTAAGTAATA

CTACTCCTAGACCTCCTAGTAGAAGAACAAATCCCCCAAATAGCACAAGAATCTCATGTA

TTGGTCCAGGTAAATCCATTATGGATAAGAAGAAATTAATAGTATAAAATTTTTCATGAA

CTGACTAAAACTAAAAGATTCAAGGAAGGAAAAAGGGATTAGGATATTTTTTGTATATAA

GTTGTTAGTTATTCGTTTTTCTTTATAGTTAATAAAAATGGATTCTAAAAGATAAATCCT

AATAATTAGTAACCGTTCTTGAATTCCAAGATTTTTCTTCGTCTATTTTACTTTGAGTCG

AATTCCTAATTGTTTGAATTGTGTAATCTCCCATTATGGAGATTGGTAACCGACTCAAAG

CAATTTGATTGTAATTCAATTCATGACGATCATAAGTAGAAAGTTCATATTCTTCAGTCA

TTGATAAACAGCTTGTCGGACAGTACTCAACACAATTACCACAAAATATACAAACTCCGA

AATCAATACTATAATTAAGCAATTGTTTCCTTTTAATATCCTTTTCAAATCTCCAATCCA

CAAGGGGTAGATCTATCGGGCATACGCGAACACATACTTCACAAGCAATACATTTATCAA

ATTCAAAGTGGATTCGCCCCCGGAAACGCTCCGATGTAATTGATTTTTCATAAGGGTAGT

GAATCGTTATAGGTAAACGATTTGTGTGGGATAAGGTAATTATGAAACTTTGACCAATGT

ACCTTGCAGCGCGTATTGTTTGTTGACCATAACTCATGAACCCAGTTACCATAGGGAACA

TATTCTAAATATCTATGAAAAAGATATGTTTCTTTCTCTTGTTTGAGAGAACTTTTGTGT

TGAAAATATTCTTACTGTTATTGTATTCTTATTTATAGTGAAACAAGTTGGGAAGAAGTT

GTTAATAAGAGATTGCCCAGGGAAATAGGTAAAAGAAATTTCCATCCAAGATTTAATAAC

TGATCCATTCTCATCCTGGGTAAAGTCCATCTTATTGTGATAGAAATGAAGAGAAATAAA

TAAGCTTTAGTTAATGTAATAAAGATACCCATTGTCATTTCCAAAATTCCAACCATTTTA

TTCATTTGGAAAAATTCAAAAAAGGATATATAGGGAATAGAGAAATTCCACCCGCCTAAG

TAGAGAACTGTTACAAATAAAGAGGAAACTAATAAATTTAGGTAAGAAACAAGATAAAAT

AAACCATATTTGATACCGGAATATTCGGTTTGATAACCTGCTACTAATTCTTCCTCTGCT

TCTGGTAAATCAAAGGGTAATCTTTCACATTCTGCCAAAGAAGAAATTAGAAAAACCAGA

AAACCTATAGGCTGACGCCAAAGATTCCATCCAAAAAAACCATATTTTGACTGTGCTTCA

ACTATATCAACTGTACTTGAACTGTTAGATAATCATAGTCGATGATAACATCACAGTTCC

CACCGCTATTCCAAAACCGTACATGAAACCTTAGCTTCATACGGCTTCTCTATGATCAGA

AAAAGGAAAGGGTTGTTTCGTTTCGGTATTATCCCCTGGGCATAGATAGAATTAGGTAAG

ATAAAATCGATTGGAAAGTCCTAAATTAGACCAAAGGAATTCCGTCTGCTAGAATAAGAA

AAAGCGCTTCCGAATTGATCTCGTCCTTTATAATATAAAATTTTTCTTTGTTCAGTAATA

ACTTAATCTTGGAATAAAACACTCGTTATAGCAATTAATAAATGAAAAGAATTAGGCATT

AATTCATGAGGAATTCTGTATTAATATGAATAGAGGAAGGAAAAAATAAATAAATATCTT

TTTTTGTATTGCATTCCATATCTTTTGTCCTATTCTTCTTTCCCCGAAGGGTATAAAAAG

AAAAAAGGAATAAAGGATTAATTCGTTCTTGATAGCCATTTCTTTAACAAGTGAAAGGGA

ACATACTCTGGATCGGAATCCGAAGAAGTACTACTTGATCATTTCCACCAATTTCAAGTC

CTTATTATGATTCCTTTTATGAGGAAAAATCTCTAATGCCCTTTATGTACTTTAGTGTTC

CTAACCCCTCACTAATTTTTGATGGATTCCCTTATGATTATAAGTTTCCATATCGCGAAT

CCTTTATTCTTGCCCGCTTCAAGATATGATGACTAATCAAAAAATCTCAACCTTGGGGTA

AAGAGTTTACACTACTTATGTTTACTTCAATTTTTTCTTGTACGTAGGAAATGAGATTTT

TTCTTTTTACTACAAATTAATAAGTTGTTTTGTTTCACTCATATAGCTATCTAGTTTAAC

TTACCAACCCGAGAATAAGAAAAGGAAGATAAATATTCAATGGATTTTGGAGGAAAAAGA

TCCTATTTTAACGAATCACACGTAGAGATATTGCTAGCACACAAAAAGTTAATGGTATTT

CATAACTAATAGATTGAGCAGCAGCTCGTAGACCGCCTGAAAAAGAATATTTATTATTTG

AGCTATATCCTGCCATAAGAAGACCAATAGGAGCAATACTTGAAATGGCAATCCATAAAA

AAACACCAATACTAAGATCGGCTAAAACAAAACGATATCCCAAAGGGATAACTAAAAAAC

TTAATAAAATTGATATGACTGCTATAGAAGGTCCAATGCTAAATAAAGGAATATCTCCTC

GGGATGGCAGGATATCCTCCTTAAAAAGTAGCTTAGTTCCATCGGCTATAGCTTGAAGCA

GTCCCAGGGGGCCAGCATATTCAGGACCAATACGTTGTTGTATCGATGCGGATATTTCTC

TTTCTAACCACACAATTACGAGTACTTCTATTGTGATTCCCAGTAGGAGGGTCAAAATGG

GTAGAATCCATATCAGTCCATAGACTTCTTTTAATAATTCCGATTTCGAAAAAGAATTGA

TAGTTTCTATCTCTACCCTATCTATTATCATTTCAACGATCAACTTCCCCCATAATGATA

TCTATACTACCTAATATCGTCATGATATCAGCCAATTTCATTTTTTTAACTAGTTGAGGA

AGAATTTGCAAATTAATAAAACCGGGTGGACGAATTTTCCATCTCCAGGGGAAAAGACTA

TCATCTCCTACCAGATAAATTCCTAATTCACCTTTTGGAGCTTCCACTCTTACATAAAGC

TCTTGCTTTGACAATTCAAAATTGGGCGAAGGTTTTTTACCAAGAAATCGATATTCAAAA

TCATTCCATTCGGAATTCTTTGTTTTCTTAAAGCGTCGGACTTCTAAATTCTCATAAGGG

CCTCCAGGAATTTTCTCTACAGCCTGTTGAATAATTTTGATGGATTCCCTCATTTCACCC

ATTCGTACTAAATAGCGAGCTAATGAATCCCCTTCTTTTTGCCATTGGACTTTCCAATCG

AATTGGTTGTAAGACTCATAAGGATCAACTTTACGAAGATCCCATTGTATTCCAGAAGCT

CGTAACATCGGTCCCGATAAGCCCCAATTTACTGCTTCTTCTCCGCTAATAAAACCGACT

CCTTCAACTCGTTCTAAAAAAACGGGATTCCGTGTAATAAGTTGTTGATATTCAACAACT

CCTCGTAAAAAATAATCACAGAAATCTAAACATTTATCGACCCATCCATAAGGTAGATCG

GCGGCTACCCCTCCGATGCGAAAGTAATTATGCATCATTCGCATACCTGTAGCAGCTTCA

AATAGATCATATATCAATTCTCTCTCTCTAAAAATATAGAAAAAAGGGGTCTGTGCGCCT

AGATCCGCCATAAAAGGTCCAAGCCATAACAAGTGAGAAGCTATACGGCTCAACTCTAAC

ATAATTACCCTAATATAGCTGGCTCTTTGGGGTATTTGAATATTCTCCAAGAATTCTGGT

GCATTTACCGTTATTGCTTCTGTAAACATAGTAGC

[2] Fargesia_canaliculata

CCCAATATCTTGCTGGAACAAGATATTGGGTATTTCTGGCTTTCCTTCCTTCAAAAATTG

CTATATGTTAGCAGAAAAGCCTTATCCATTAAGAGATGGAACTTCAAGAACAGCTAGGTC

TAGAGGGAAGTTGTGAGCATTACGTTCGTGCATTACCTCCATACCAAGATTAGCACGGTT

GATGATATCAGCCCAAGTATTAATAACGCGACCTTGGCTATCAACTACGGATTGGTTGAA

ATTGAAACCATTTAGGTTGAAAGCCATAGTACTAATACCTAAAGCAGTGAACCAGATCCC

TACTACAGGCCAAGCAGCCAAGAAGAAGTGTAAAGAACGAGAGTTGTTGAAACTAGCATA

TTGGAAGATTAATCGGCCAAAATAACCATGAGCAGCCACAATATTATAAGTCTCTTCCTC

TTGACCAAATTTGTAACCCTCATTAGCAGATTCATTTTCAGTGGTTTCCCTGATCAAACT

AGAGGTTACCAAGGAACCATGCATAGCACTGAATAGGGAACCGCCGAATACACCAGCTAC

ACCTAACATGTGAAATGGATGCATAAGGATGTTGTGCTCTGCCTGGAATACAATCATAAA

GTTGAAAGTACCAGATATTCCTAAAGGCATACCATCAGAGAAACTTCCTTGACCAATAGG

GTAAATCAAGAAAACAGCAGTAGCAGCTGCAACAGGAGCTGAATATGCAACAGCAATCCA

AGGACGCATACCCAGACGGAAACTAAGTTCCCACTCACGACCCATATAACAAGCTACACC

AAGTAAGAAGTGTAGAACAATTAGCTCATAAGGACCGCCGTTGTATAACCACTCATCAAC

AGATGCAGCTTCCCAAATTGGGTAAAAGTGCAATCCGATCGCCGCAGAAGTAGGAATAAT

GGCACCAGAGATAATATTGTTTCCATAAAGTAAAGAACCAGAAACAGGCTCACGAATACC

ATCAATATCTACTGGAGGAGCAGCGATGAAGGCGATAATAAATACGGAAGTTGCGGTCAA

TAAGGTAGGGATCATCAAAACACCGAACCATCCGATGTAAAGACGATTTTCGGTGCTAGT

TATCCAGTGCAGAAGCGACCCCACAGGCTTGTACTTTCGCGTCTCTCTAAAATTGCAGTC

ATGGTAAGATCTTGGTTTATTCAAATTGCAAGGACTCCCAAGCACACGTATTAACTAAAA

AGATAATAGAAGGCTTGTTATTTAACAGTATAACATAGACTGTATACCAATGTCAACCAA

GCCAGCCCCAACGATTGGATATCCATATAACTAAATTCACCAAACCAAAAATTTTGTAAA

TGAAGTGAGTGAAAATTCAAAACTCAGATTATTTCCATATGGGTTGCCCGGGACTCGAAC

CCGGAACTAGTCGGATGGAGTAGATAATTCTTCCTTGTTACAATAGAAAAAATCCCTCCC

CAAATCGTGCTTGCATTTTTCATTGCACACGACTTTCCCTATGTAGAAATAGTCAATTTC

TATTCCAAAGAGGAAGTCTACCAATTTTTTGAATAGTAAGTTGATTCACCTACTATGAAC

ATTTCAGAATGGAAAATGTGAAAGTTTTATCTTGATATCGATCTTTCTAGTGTATTAGTT

TTGTCTAATGATTAATTAAAAGGGTTCACCAGGTCATTGATACGGATAATATCCAAATAC

CAAATACGGTCACTGTGTGATCCACGGAAAGAAAAGTGGGTTGTTTTGGTGAACATCAAA

GAAAAAACTTGCTCTTCTTCCGTAAAAAATTCTTCTAAAAATACCGAACCCAACCGTTGC

ATAAAAGTTCGTACCGTGCTTTTATGTTTACGAGCTAAAGTTCTAGCGCATGAAAGTCGA

AGTATATACTTTAGTCGATACAAAGTCCGTTTTTTCGAGGATCCACTATGATAATGAAAA

AGATTTCTACATATCCGACCAAATCGATCAAGAATATCCCAATCTGATAAATCGGTCCAA

ATGGGTTTACTAATAGGATGCCCCGATCCAGTACAAAATTGAGCTTTTGATAAGGATCCA

ATGAGGGGAGTAGCAGGGACTATGGTATCGAATTTTTTCATTTGAGTCTCTATTAGAAAT

GAATTCTCTAGCATTTGATTCCTTACTAACAAAGGATTTATTGGTACACTTGAAAGGTAC

CCCAGAAAATCGAAGCAAGAGTTTACTAATTGGTTTAGATGGATCCTTCGCGGTTGAGTC

CAAAAAGAAAAAGAATATTGCCAGAAATTGACAAGGTAACATTTCCATTTCTTCTTCAAA

AGAAGAGTTCCTTTTGATGCAAGAATTGCCTTTCCTTGATATCGAACATAATGCATAAGA

GGATCCATAAAGATCCATACGGTTTTCCGAAAAAAACCTGGGTACATTACCCCAAAATGT

TCCATCTTCCTAGAAAAGTGGATTCGTTCCACAAAGGTTCCAGAAGATGTTAATGGTAAG

CAAGAAGATTGTTTACGAAGAAACAACAAGAAAAATTCATATTCTGATACATAAGAGTTA

TATAGGAATCGAAATAGTCTTTTATTTTCTTTTTTCAAAAGAAAAATCGATTTCATTGAA

GTAATAAGACTATTCCAATTCGAATAGTAGTTGAGAAAGAATCGCAATAAATGCAAAGAT

GGAACATCTTTGATCCGGTATTGAAGGAGTTGAACCAAGATTTCAAAATGGATAGGATAG

GGTATTTCTATATGTGATAGATAATGTAAATGCAAAAATTTGTCTTCTAAAAAGGGAAAT

ATTGAATGAATAGATCGTAAATTCTGAAACTTTGGTGTTTCTTTTTCTTTCGGACAAGAT

AATTCTCGTAGCGAGAATGGGATTTCTACAACGATCGCAAACCCCTCAGATAGAATCTGA

GAATAAAACTCAGAATAAAAAAAATTGTTGTAATCCAACAATCGATCTTGGTTAGGATGA

TTAACCGAGTTAATCCAAAAATTCTGCTGATACATTCGAATAATTAAACGTTTCACAAGT

AGTGAACTAAATTTCTTGTTATTACAACTAACAATTTCCACAGGTTCGGAACCTTTTAAT

CCATAATCATGGGCAAATGCATAAATATACTCCTGAAAGAGAAGTGGGTAAACGAAGTAT

TGTTGACGAGATTTCTGTTTTTCTGAATACCCTTCCAATTTTTCCATTTGTATTTCTACT

TGAATCAGAAAGAAGAAGCATTTCTCGGTTTCTCAAATGATGATACATAGTGCAATATGG

TCAAAACAGGGTGTTGCATAATACAAACCTTTCTATCCAATTAGTTTATGTTTGTTCTAA

TTACAAAAGAGAACAAATCTTTTATTTTTGCAGGCCAATCGCTCTTTTGACTTTGGAATC

CAGTCTCTTTATCAATATACTGCTTCTTTTACACATTCAATCCATAACATCCTTTTCAAT

CTATAATCAAGAATAATTAGGATTTCAAAAAAAAAAGAAAAAGGGTCCGTTCATAGGAAA

ACCAACCTTTCCCCGCATCAGGCACTAATCTATTTTTAACGTCTAATTAGATCGGGGAAT

CATTTCAATTAAGAAGTTAAGCTCGTTGCTTTTTATTTTACCAGAATTGGAGCCAGGCTC

TATCCATTTATTCACTAGACCCAGAAAATAGGAATTTTTTTATTCCAAAAAAAAAAAGAA

ATTGATTTTATTACGACATGCTATTTTTTCCATTCATTACCCTTGAGGATCAGTCGTGGT

CTTCTAGACTCTACCAAGAGTCTGGACGAATTTGTTGCTCATCCAAATGTGTAAAGATCA

TAGTCGCACTTAAAAGCCGAGTACTCTACCATTGAGTAGCACCCAGATAAATAGGATCTT

AGATACGATCGAACCCAAAATCAATGGAATTACACCACATTGAACTAGCAAAACATTAAA

AGAAAGATTTTATCGCCATTAAAAACACTCAAATGCAAAATGAACAGGTCCGGCTAAATT

TCACTAAGGTTAAAAGCGGCCCCAATCACGATAGCAAAATTGTCATTTTTTTAGCATTTA

TATATATAAATAAATCTTGTATGAGAGTACATGCAAGAGGGACAACCTTATCATTTGAGC

GAAGTGTAGACAAAAAACCTAATATGGAGTGAGGATAAAGAGACCTATCTATCTACAAAT

TCTATTTGTTCAATAGACCTTTGTCAATGGAAATACAATGAAATTAGATAGAAAAAGTAA

ATAAAATAAGGGCTTATGTTGGATTGGCACGACATAAATCCAGTCAAAAATAGGACTAAG

AGGCAAATTGTGTCTAAATAATTAAGGGATACTAGTGATCCTCTCCTACTTTTTTATTCA

TTTAGTTCTTCAATTAACTCAAAGTTCCTTCTTTTTCTTTAAAGAATTCTGCCTTCCTTA

AAATATCATAAACAGTTCTTGTAGGTTGAGCACCCTTTTCAAGGAAATAGAGAATAGCTG

GAACATTTAAACAAGTTTGATTCTTTATCGGATCATAAAAACCTACTTTTCGAAGATCTC

TTCCTTCTCTTCGAGATCGAACATCAATTGCAACGATTCGATAGACAGCTTATTGGGATA

GATGTAGCTAAACAATCCCCCCCTAGAAACGTATAGGAGGTTTTCTCCTCATACGGCTCG

AGAATATGACTTGCATTAATTTCCTTACAGAAAAACAAATTTCATTTATACTCATGACTC

AAGTTGGTTAATTTTGACTGACAGACTTAAAAGGAAAAATCCTTCCAAATTTTTTGAGTC

GTCTCTAAACTCTTTTCTTTGTCTCATCTCGAACGAATTGACTTTTATTCCTTATTCTGA

TCCAATTCTATTGTTGAGCAATTGAAAATCGCGTTTACTTGTTCCGGAATTCTTTATCTT

TGATTTGTGAAATCCTTGGGTTTAGACATTACTTCGGGAATTCCTATTCTTTTTTCTTTC

AAAAGAGTAGCAACATACCCTTTTTTCTTATTTCCTTCGATAAAGCATTTCCCTCTTCTA

TAGAAATCGAATATGGGCGATTGATTCTGATAAACTTTTAATTGAAAGAGTTTTTCCAAT

CTTCCAAAATTGGACTTTTTCTTATTTTAACCTTTCGATTTCTATATTAAGGATAGACTG

ACAAAGTTGGCCTAATTTATTAGTTTTCACTAACCCTAGATTCTTTCCCTTGATAAAAAA

TCAATTCTGTCCTCTCGAGCTCCATCGTGTACTATTTACTTACAAACAACCCAGCGCAAA

TTTGGTTCGGGACGAATAGAACAGACTATGTCGAGCCAAGAGCATTTTCATTACTATGGA

AAATGATGGATAACAAAATCCACAATCGATCATGTCCTTCAAGTCGCACGTTGCTTTCTA

CCACATCGTTTTAAACGAAGTTTTACCATAACATTCCTCTAATTTCATTGCAAAGTGGTA

TAGGGAATTGATCCAATATGGATGGGATCATGAATAGTCATTTTTTTGTATACTAATTCA

AACTTGCTATCTATGGAGAAATATGGATAAAAGAAATAAGTATTTATCGGGGAAGACTCC

GCAAAGATCCAATTTATTTAAACCCATATTCTATCATATGAAGGAAACATAGTTCGAAAA

AGACGAATAAACAAGTTTGCTTAAGACTTATTTTTTATTGAATTTCCATCCTCAACAGAG

GACTCGAGATGGTCAATCCTGAAATGAGAAGGATCGACTCTTCTCCAACAAATAAACTAT

CAACCTCAAGTTTAATTAATTTAATTAGCAATATATTTTTCCATAACAAAAACTATTAAC

TAAATAAACTATTCCAATGAAAAGAAAGTTTTTTGGTAGTTATAGAATTCTCGTACTCTT

CGACTCGAATACCAAAAGAGGACTCCAAATCAAAATTGAATCCATTCTATCCAACGAACA

GTTCTTACCTTATCCTTACCAGAATGGATCATCTGGATATTTAAAGAATCGCAGATCGAG

ATGGTTTTCGCTTAACCAAAGAGGGGCCCTTTTTACTAATAATATAATACAACAAAAATC

TATCTCTATCATAAAGGGATAGGTCTCATTTTTTATACAGTGTTTTACGTTTTTTCATGA

AAAAAAGATATTCAATTTGACTGGACTTGACACTTGATTATGTTTTCTGAGAAAGAAAAA

AATGCTTAGAAATGCATCTAATCTAAGAGTTCATAAGAGATAATTATTCTCTTTAATAAA

CTTTTGTCTTGTGTGGGGTACAATATGATTTCATCTTTCGTTTCATCAGAAAAATCTGGG

ACGGAAGGATTCGAACCTCCGAGTAACGGGACCAAAACCCGCTGCCTTACCACTTGGCCA

CGCCCCATTTCTGGTTTTATGCGACACTAATAAACACTATTATGTTTATTTGTTATTCGT

CAATCCCACTTCAATTACATAAAAATGAGGGATACTCTCTTGCTAGGATTCTAGACATGC

GGATAATATAGAATCCAAAAAATGCATTGATCATTACATGGAATTCTATTAAGATATTAT

ATGAAAGTCGAATTTCTTCCATTCTCATTTGAGAGTGCGAATACAAGGAGGTATTTTGCG

TTTGGGAAAGTCCGAAGAAAAAAGGATTTTGAACCCGCCTTTTCTTTTTTCCCTTAGAAA

AATAACTCAATCAAAATCCAATTATCTACTCTACAAGAACGAAATGCTTGTTATGCCTAA

TATACTTAGTTTAACCTGTATCTGTTTTAATTCTGTTCTTTATCCGACTAGTTTTTTCTT

CGCCAAATTGCCCGAAGCTTATGCCATTTTCAACCCAATCGTGGATTTTATGCCTGTCAT

ACCTATACTCTTTTTTCTATTAGCCTTTGTTTGGCAAGCTGCTGTAAGTTTTCGATGAAA

TCTTTACTACTCTGTCTGCCAAATTGAATGATGTATTCATTCCAAAAAAAAAAATGAATA

AAAGCCGAGAAGTCTTATATTATGAACCTTCGATTCTAAAATTCTAATTCTTCTACATTG

AATGTATAGCTGCAGCAATAAATTGGGATCCGCCTTTCTACCCCTGCACCTACGTTGAGC

AGGTACCTTTAGGTACCCACACAATACCTAACCTAATTTTTTGATAAGAGTGCTTATTAT

AAATCAATTCTTGCAATTTTTTTAAGAATTGATTTTTGCATTTTTAGGTGTAAAAATAAA

AAAACCCATCCTAGTGGATCTGTGTGGTAAGGAAAACGGGTAATCTATTCCTTAAAAAAA

ATCTTGGAGATTATGTAATGCTTACTCTCAAACTTTTTGTTTATACAGTAGTGATATTCT

TTGTTTCCCTCTTTATCTTTGGATTCTTATCTAATGACCCAGGACGTAATCCTGGGCGTG

AGGAGTAAAAATCCAAAATTTTTTCTTACAAATTGGATTTGTTTCGTACATTTATCTATG

AGAAAATCCGGGGGTCAGAATTCCTTCCAATTCGAAAGTCCCAAATGATCCGAGGGGGCG

GAAAGAGAGGGATTCGAACCCTCGGTACAAAAAAATTGTACAACGGATTAGCAATCCGCC

GCTTTAGTCCACTCAGCCATCTCTCCCCGTTCCAAATCGAAAGGTTTCCGTGATATGACA

GAGGCAAGAAATAACGATTGCAAAAAATCCTTCCTTTTTCTTTCAAAAGTCCATAAAAAT

TATATTGCCAATTCCATTTTAATTATATTCTTTTTTCTTAATAAAAAAGAAGAAAATTCT

TGTTTTTTCTTTCTAAAATTCGATATTGGCTGAGAAACAATCAGATAGATTTTCTCTTCA

GCGGGCATTTTCATATAGGACTTGTTATAATAAAACAAGCAGGTTATATAAAAAATATTT

ATAAACAAAACAAAAAGGGTTCTTATCAAACCCACCATAAAATTGGAAAGAAAGATAAAG

TAAGTAGACCTGACTCCTTGAATGATGCCTCTATCCACTATTCTGATATATAAATTCGAT

GTAGATGAAATTGTATAAGCGGATTTTTGTATTTCCTTAGACTTAGACCGCGCAAGACAA

GAATTTTTCGCTATTTACGATTTCATATTCTTGTTACTAGATGTTCTATAGGAATAAGAA

GAAATCGCAACTCCTTTCCGCTACACATAAAAATTGATTTCGAAAGTCAATTTTTTTCAA

TATCTTTCTTTTCCTTAAAAGATAGGCTTTGAAATAGGAGTCATGGAATAATGCTGAATT

CAAATGTTTATTTCTATAGTATAAGAAAAACTAATCGAATCAAATTCATGGATTTACCAC

GACCTCGGTTGTGACCCCATAGATAAAAATAAAAAATTTCTATCTTCGAGACCTTTGAAA

AAGGGCATTGAACGAGAAAAAATCGTCCACAGATAATCTATCGTATGCCTTGGAAGTGAT

ATGAGGTGCTCGGAAATGGTTGAAGTAATTGAATAGGAGGATCACTATGACTATAGCCCT

TGGTAGAGTTACTAAAGAAGAAAATGATCTATTTGATATTATGGACGACTGGTTACGAAG

GGACCGTTTCGTTTTTGTAGGATGGTCCGGCCTATTGCTCTTTCCTTGTGCTTATTTCGC

TTTAGGGGGTTGGTTTACAGGGACAACTTTTGTAACTTCTTGGTATACCCATGGATTGGC

TAGTTCCTATTTGGAAGGTTGTAATTTCTTAACCGCGGCAGTTTCCACCCCTGCCAATAG

TTTAGCACACTCTTTGTTGCTACTATGGGGCCCGGAAGCGCAAGGGGATTTTACTCGTTG

GTGTCAATTAGGCGGTCTGTGGACTTTTGTCGCTCTCCATGGGGCTTTTGCACTAATAGG

TTTCATGTTACGTCAATTTGAACTTGCTCGGTCTGTTCAATTGCGGCCTTATAATGCAAT

TTCATTCTCTGCTCCAATCGCTGTTTTTGTTTCCGTATTCCTTATTTATCCACTGGGGCA

ATCTGGTTGGTTCTTTGCGCCGAGTTTTGGCGTAGCAGCGATATTTCGATTCATCCTCTT

TTTCCAAGGATTTCATAATTGGACATTGAACCCATTTCATATGATGGGAGTTGCCGGAGT

ATTAGGCGCGGCTCTGCTATGCGCTATTCATGGGGCGACCGTAGAAAACACTCTATTCGA

GGACGGTGATGGTGCAAATACTTTCCGCGCTTTTAACCCAACTCAAGCTGAAGAAACTTA

TTCAATGGTCACTGCTAATCGCTTTTGGTCCCAAATCTTTGGTGTTGCTTTTTCTAATAA

ACGTTGGTTACATTTCTTTATGCTATTTGTACCCGTCACCGGTTTATGGATGAGTGCTAT

TGGCGTAGTCGGCCTGGCTCTGAACCTACGTGCCTATGACTTCGTTTCCCAGGAAATCCG

TGCAGCGGAAGATCCTGAATTTGAGACTTTCTACACTAAAAATATTCTTTTAAACGAGGG

TATTCGTGCGTGGATGGCAGCTCAGGATCAGCCTCATGAAAATCTTATATTCCCTGAGGA

GGTTCTACCACGTGGAAACGCTCTTTAATGGAACTTTCGTTTTAGCTGGTCGTGACCAAG

AAACCACCGGCTTTGCTTGGTGGGCTGGGAATGCCAGACTTATCAATTTGTCCGGTAAAC

TACTTGGAGCTCACGTAGCCCATGCCGGATTAATCGTATTCTGGGCCGGAGCAATGAACC

TATTTGAGGTGGCCCATTTCGTACCAGAAAAGCCCATGTATGAACAAGGGTTGATTTTAC

TTCCGCACTTAGCTACTCTAGGTTGGGGAGTAGGGCCGGGGGGAGAAGTTCTAGATACTT

TTCCGTACTTTGTATCTGGAGTACTTCACCTAATTTCCTCCGCAGTCTTAGGCTTCGGCG

GCATTTATCACGCGCTTCTGGGACCCGAGACTCTTGAAGAATCTTTTCCATTCTTTGGTT

ATGTATGGAAAGATAGAAATAAAATGACTACAATTTTGGGTATTCACTTAATTTTGTTAG

GTATAGGTGCTTTTCTTCTAGTACTCAAGGCTCTTTATTTTGGCGGTGTATATGATACCT

GGGCCCCGGGGGGGGGAGATGTAAGAAAAATTACCAATTTGACCCTTAGCCCCAGTGTTA

TATTTGGTTATTTACTAAAATCCCCTTTTGGGGGAGAAGGGTGGATTGTTAGTGTGGATG

ATTTAGAAGATATAATTGGGGGACATGTATGGTTGGGTTTCATTTGTGTATTTGGCGGAA

TTTGGCATATCTTAACCAAACCCTTCGCATGGGCTCGCCGTGCATTTGTATGGTCTGGAG

AAGCTTACTTGTCTTATAGTTTAGGCGCTTTATCTGTCTTTGGTGTTATCGCTTGTTGTT

TTGTCTGGTTCAATAATACGGCTTATCCGAGTGAGTTTTATGGACCCACTGGGCCAGAAG

CTTCTCAAGCTCAAGCATTTACTTTTCTAGTTAGAGACCAGCGTCTTGGAGCTAATGTGG

GATCTGCCCAAGGACCCACAGGTTTAGGTAAATATCTAATGCGTTCCCCAACGGGAGAGG

TTATTTTTGGAGGGGAAACTATGCGTTTTTGGGACCTTCGTGCTCCATGGTTAGAACCTC

TAAGGGGGCCCAACGGTTTGGACTTGAGTAGGTTGAAAAAAGACATACAACCTTGGCAAG

AACGACGTTCGGCAGAATATATGACCCATGCTCCTTTAGGCTCTTTAAATTCCGTGGGTG

GCGTAGCTACCGAGATCAATGCAGTTAATTATGTCTCTCCTAGAAGTTGGTTAGCGACCT

CCCATTTTGTTCTAGGATTCTTCTTTTTTGTGGGCCATTTGTGGCATGCAGGAAGAGCCC

GGGCTGCTGCAGCAGGCTTTGAAAAGGGAATCGATCGTGATTTGGAACCTGTTCTTTACA

TGAACCCTCTTAACTAAGATTTTTTATTTATAGCTGTTCTAGTTTTTTTCTGTTCTGGCT

CGGTTATTCCATCTAGCCGAGCCATTCATTCCTTAAAAACAAATAAAGAAACAAACGTAT

TCAATAAGCAAAAGGAGAGAGAGGGATTCGAACCCTCGATAGTTCCTAAAACTATACCGG

TTTTCAAGACCGGAGCTATCAACCACTCAGCCATCTCTCCACAGCCTAATCCCTATTTTA

TTCCTACAAATAGAACATAGCCATATGAAATGATCTACTAACCCATCTCAGATGCAAGTC

CCCTTTCGATATATCTCTGTATAAGGTGGTAAGTAATAAGTTTTAAAGAGAAGAATCAAT

GGATTCATGATTAAACCCCTCCTACTTCTTGTATTTTATTACAATTTTGGTTAAGTGAGG

GATCAAATATGTAGTCAACTTTATTTGATGGTAGCTTGGAGGATTAGAAATATGACTATT

GCTTTCCAATTAGCTGTTTTTGCATTAATTGCGACTTCCTCAGTGTTAGTAATTAGTGTA

CCCCTTGTATTTGCTTCTCCTGATGGTTGGTCAAATAATAAAAACGTTGTATTTTCCGGT

ACATCATTATGGATTGGACTAGTCTTTCTGGTAGCTATTCTGAATTCTCTCATTTCTTAA

ATTTGTTTAGTATTTAGTAGCCCGATACAAAATATAAAAAGGCCATTTCTTCGAATTGTG

AGACGCATTAAAATGCAATTTGCGTTCCGAATTGATTGACAGACAATTAAAAAAAGAAAA

CTCTAATAGAAAATGAAACGGTCGACCCAGACATAGACGGTCGACCCAGGCGGATATACC

CTATAAAATATATCCCGTAGCGAGCGTAGTTCAATGGTAAAACATCTCCTTGCCAAGGAG

AAGATACGGGTTCGATTCCCGCCGCTCGCCAGCTTAATTTAGTAAGGTACTATGATAAAA

AATTTAGTCTACTTATATTAAATTAATAGGTGTTAGTCTAGTACCGTATCCCTTACTATC

TTACCCTCTTTTGCACCCCACTCAAAAAAAGGGGCTCCGGAGGCGGGAATCGAACTCGCC

AACAGGGCTCCCTAAATTGGGGATTCACCGAGACAAACAACTGGCAAACTCTTTTAAAGG

GGTAGACTGTGCCTTTCTTTCATTTCTTTTTTCTTTTCTTCTTCTTGCTAATGAATAAAA

AGGGTTGGATCCAGCCCTCTACTCTATACAAATAGAATAGTCCTTTTATACAGACTGCTA

AGTGCGGAGACGGGAATCGAACCCGTGACCTCAAGGTTATGAGCCTCGTGAGCTACCAAA

CTGCTCTACTCCGCTCTGGAGGGACGGAAACTGGTGGACGAAAAAGGTTGAATACAGGAC

TCTACCATGTCTAGACAAATAGAATAGTCCTTTTATACAGAATGGAGCGGGTAGCGGGAA

TCGAACCCGCATCGTTAGCTTGGAAGGCTAGGGGTTATAGTCGACGTTGGTTGATTAGTT

TTAACGTCTCTAATTCAAAACCGAACATGAAATTTTGATTTCATTCGGCTCCTTTATGGA

TATTCTCACCACTTAACATCTATGTCAGCTTTTCTATCTGAATGGAACCAAAGCTCTCCG

CTTTCTAGATGATCCCTATAGAGTAGGAGATAGAAATTCTACTAAATCTATCTAATCTAC

TTACTTCGTTCCCTAATTTCATTCAAGAGATCCTGAGGAAAAGAATTAGGTTTCCACCGA

GCTGAAACAATATGCTGATGGTTCTAGTAAACCAAAACTACCGTTTTTTAGCTATTTGGC

TTCCATTTCCTTTTTAACAAAAGAAGATTTAGTTACGATTGGAAATAAACTTTTTTGTAT

CTTCATCCATAGATCCTTTACTCATATTTTAAAAATTGGAATACTTAATCCAATGCAAAA

TTATGCTTCGCGACTCTGTACTCATAATCCAATTTGTATTTTGGATGCAATTTCAATTAG

TTTTTGGGTACAAATCGCGAGAATGTATATTCTTCCTCAATATGCTATTGAGAGGAAAAG

GATTAAATCCTTTATAAGAACTAAAGTTTTCATCGGAATATAAAAAACTTAAGGACGCCT

TAAGTATATCATTTCAAATTCAGTTATTAATAGAACGAATCACACTTTTACCACTAAACT

ATACCCGCTACATGTAGATTATGATACCAACGCTACCCTTTGTCAAGGGTAGCCATTCGA

GAAGGAGGCTAATTCCCCCTTATTGAATCAAAGGAGAAGGTTCATGACAGTGAGCTGTTG

GTACTTCGATCGCGGGCCTTTATTTCTTTCTTTTTTGTTCAATTCTGAACAAAGAAATTG

GGGAAGATGTTTTCTTCCCCCACTTATCATGAAGTCCGAGCCCTAGAGAAAGAGTGAGAT

GCTTTTAAAAATTCATCATAGACTTTCCCTATGGCTTGAGAGAAGCAAGAAATAACTTAA

ACGGAGAAGCGGACAGGACCCGCTGGTTAGTCGATCCTCTCCATTTACCAATTTCTTCTC

CTCTTTTCCACTCAATTCTAGTTTATTAGATTCTTGTTTAAAAGAATCAAAGAAGATGAA

TAGAACTAAGAACACATAAAAAAAGCATAGAGGACCATTACCAAATGTTCCTCCCAAGAA

TCATATTGGGTATCTGTTCCCTTCCTTTTCCCGCTAGGATCGGGAATCTAGAATCCTCCT

TTTTCCTAATCTCGGAAACAGAAAACCCATAGCCAGGAGCAGTATAAATTCGACTGCCCA

CTTTTTACAAGCAAATTGTTGCTAAAACTCCAACATAGTTTGTTCAAAATGCACCATAAT

CCCTTGAGAATATTAAAGTACCCCCCTTCCAAGGGGTACCTGTTAAAAATAGTTTCAATT

TCTCACCAAAACAGACAAGAAGTATATCACTGAAAATTAATACCCAACCATATGGGTATA

TGAAGAGCGCGAATTCCTTTATACCCTACCCAATTAGAAGAAATAAAACATAAATGGAGA

AAGTTCTCATCATAAGATCAAAAAAAACCTCTACTTTGTGCAAGTGATAAGAGAGAATGA

AATTCTTATTTTTCTTGATTTTTTTGAACCTCGCCATGAATAAACTTCTATATCTCGATA

TATACATATATTATGTACATTATGCAGTAGACTCATAATGAGAAATCAAAGTGGCTAATT

ATTGAATATCATAAAGGGCTTTTTATTTGGTGGTAGAGTAATGCCATGGTAAGACGTAAG

TCATCGGTTCGAATCCGATAAAGTACTTTTCTACTAAATTTATTCATTTCTTTTTTGAAA

ATTTCTCTTTTTTTCTTGAATTTTATGACTTAGTGTGGGATGCATGCATTTTTGGTCTGA

ACGCTAAACGAGCACGGGGTGGAAATTACAAAAAAGAAATTGGACTCTAGATCAATCAAT

ACCTGTACTGAACTAATCTAGAATAATCTATTCTTATTCTATACCCTTTAAATGAATTTC

CCTAAAAAGTAGGGAATGATCCGTGAATTAACCTAACCATCAACTAAAAAAAATCCTATG

AAAGCATAACAGAAAAGTAGGAAAGACTCTTTGCTTGATCTAGTTCTTCGAGTATATGAC

AATTCCAAAAAACTGCTCATACTATCATTATAGTATAATGACGAGCGGTTGTATATGGCC

CTATCGTCTAGTGATGCCCCTATCGTCTAGTGGTTCAGGACATCTCTCTTTCAAGGAGGC

AGCGGGGATTCGACTTCCCCTGGGGGTAGGGAGTATTATGAAAGGAGGTTAATCATAGAT

TCTAAAAAACCCTAGAATAAATTCTTCCTGGGTCGATGCCCGAGCGGTTAATGGGGACGG

ACTGTAAATTCGTTGACGATATGTCTACGCTGGTTCAAATCCAGCTCGGCCCAAAAATCT

AGGGCTTCGTGAATATGAACTAAATCCATTTTTTTCTTCCATAAAATAAAATGTCTGATC

CATAGAAATAAAGGATAAAGCGAAAGGGGGAAATTTCTTTCTAATCCATATCTCTCTCAT

TCCTTTTTTACAAACAAAAGAGTTTTTCTTATTGAAGGTGGATTATCATCCATTTTTAGC

GATAAAAAATCGCGACATACTAGTTATGTCACTCTCACTATACCCACATATGATATGTGG

GTATGTAGTATATGATTCGTCTATTTTTAGAGTACGACAGGCGAATCGAATCTTCTATTT

AGGTATGCCATACACCCCGCGGGGATTGTAGTTCAATTGGTCAGAGCACCGCCCTGTCAA

GGCGGAAGCTGCGGGTTCGAGCCCCGTCAGTCCCGAACTAGGGTTCAATGAATGGAGAAA

TTCATCTTTCCTTTTTCCATGAAAAAGGGGGGGCAGGAGAGAAGATCAAATACCTATGGG

GCACCCTTATTTCACTTTTTTATTTCGCATTTCTCATTAAGAGGGAGGGGTATAGGATTT

TTTTTCACTACTCCCGGTTGATAAGGAAAGACATACATATCATACTTGGAGGATCTTCCT

ATGTTATACTATTCCACTCTCAACCATGAATTGATTTGATAGATCCGATATTCATAATAT

TGAATTGATTCAGTATTATCAGAATGCAAGTCCTCCCCTTGAATTTACAGGATACCCTTT

TTCCTCTCCATGGGATTACATCCCGAGTTATTGTGAAAAAAAAAGAGGTTATGGAAGTCA

ATATTCTCGCATTTATTGCTACTGCACTGTTTATTCTAGTTCCTACTGCCTTTTTACTTA

TTATTTATGTAAAAACAGTCAGCCAAAATGATTAATTGGAATTCCAATTAATCATTGAAG

AAATGAAAAAGGGATTAAATAAAATAAAAATCCAAGTCTTAAATGAAAGGATCTGGTTGG

AATCATAAAGTGTGGTAGAAAGAACTACATATAGTTTTTTCTACGACACTTTAGAGTCTT

TCTATTATATTATCTTGAATCTACATAGAATAGATTAGTAGATTGAAATAGTAGTCTAAT

TCAATTTCTTTTTTCACTGCATCCACTTAATTTCAATCAAGTCAAAATGAAAGAATCCAT

GGAGGGAGAGAAAAATAATATGAGAATAGACTATAGAAAAGAAAAAAGTAAAAGAAAAAA

CCAGCGAATCTTTCATGCTTAAACATGCGGCGAGATGCTTCAAAAGAGCATAAAAATTAT

TTAAGAATAAGAAAAGAATATAAATGGAAAGTGTGCGATATGTTGTGAATAGCTCCGTGG

AAGAAAGTCTAATTTTCTTATGTATAGAACTTTTTTAACCATTCGTCACTTCTAGTAGAA

ATTTTGAATTGCTGTAATCGCTCTTTCTATTTCTATATAGTAGAATAGAACGACTCTTTC

TTACAAGAGTTTCTTACAGGAGTGAAACAAAACTAAAGAAAGAAAGAATAAAGTTTGGCA

AAATGATTAATGCAAAACGATCAATTAAAGAAAAAAGTTGATACAACAATTCGACTACTC

AATCAATTAGTAGTATCCCTAGAGTCCACTCCTCCCCCATACTACTAGTGAAAGAGAAAA

TGTAAAGACTACCATTAAAGCAGCCCAAGCGAGACTTACTATATCCATGTAAATTATGTC

TCCTATTTCTATGAAGGAATTATTCTACTATTGATCAATAATCATAGTGGAATCAAGGGT

ACAGAGTCAAAAAGGGATTCTGCCCTAACGCTATGGATGAATCAGTTCAAGGAATTTACT

CCTAACAAATTCTTATAGGATTTCTGGTAGAATTGGAGAGCATTAAGTATAAATACGATA

CATAGCCCTTTCTATTAATAAAAGAATAAGGAAACGCAACCTCATCCTTATTGGTAGCCG

TTTGGGCCACTACCGACAAAACAAACCCTAATAGAACTATGGATTCTCAAAATCCAGTAT

CGCCAGGCCTAGTTACTCTCTTGCCCCAACTTAGCAGGGTACGAATTTGTTGAGTTCGAT

CAGTACTATAAGCCTAAGTATTTTATTGATCAGGCGGCACCCAGATTTGAACTGGGGATA

AAGGATTTGCAGTCCCCTGCCTTACCGCTTGGCCATGCCGCCAAAAAATCCGATCTAAAA

TAGAGAAAAGAGCAAGTATTCATCCAGGTTTCTTACTAAAACCTCCTTTCTTTTATCTTG

AATCTAATTCTACTTACTTTTTTCCAATCTTTTTCAAAAAAATTCCTGCTTTTTTGAATC

CAGTTTCGATTATTCTCCTCGATGGATTCTATCTTAAAACAAACATTGCTAACACTAGAA

AACTTCCCTTTTCTTTCTATTGAGATGAAAAAAGAGAAAAGTGGATTTCCAGTCACAGGC

TGCAAAATTCAGAACAAATTGGAACCATTAACTAGAATTCTATTTTTTGAATTTCGGTAT

TCTCCCCCCTTCCTTTTAATGGCATAATAAAATAGAATGGATTTATGCCTAATCCGTGTA

TAGGTAAACTACAGGTCCGAACAGCATTATTATCCATGGATCCCCCTTATGTACATATCT

CTATGGGGAATCGTGCTTTAATTTTTCATTGCATTAAATATCTTGAATAAAAAAAAGAAA

TTTGGTCTGATATGGAGAGGTGGATAACTAGATTGGCATGTACTTAAAAAAGGACTTACT

TTATTTTAGGATTCTACAATGAAATCCTATATTTTCTAGCAATTCTACTACTACGAAACA

AAAAAGAACCCTCAAATTCTTTTTAAAGGAGATAAAATGAGAAATCTTTGCCATCCAATC

TGATTATATCATTAAGTGGCAGAATTTTTTTCTAGGAATGTTTTATCAATTCATTTTCAT

TCGATTTGTACCCCTGGCAAATTCGAACTTTCGTCGAAATTGTCTCTATTCATATGTATG

AAATACATATATGAAATATGTATGTGGAGTTCCCTAGAATTTCATGTGATTCAGTAAACA

GAATATGGATTCCATAATTGCTAGATCGATCCATAGGGATTGATGAAGAGTGAGCTGATA

ATGGAATTTTTCTTCGATAAACAGGAAACTTAAGATGCTCCGGAATGGAAATGAGGGAAT

GTCCACAATACCCGGATTTAGTCAGATCCAATTCGAGGGATTTTGTAGGTTCATTAATCA

AGGCTTGGCAGAAGAACTTGAGAAGTTTCCAACAATTAAAGATCCAGATCACGAAATTGC

ATTTCAATTATTTGCGAAAGGATATCAATTGCTAGAACCCTCGATAAAAGAAAGGGATGC

TGTGTATGAATCACTCACCTATTCTTCCGAATTATATGTATCTGCGAGATTAATTTTTGG

TTTCGATGTGCAAAAGCAAACCATTTCTATTGGAAACATTCCTATAATGAATTCCTTAGG

AACCTTTATAATAAATGGAATATACCGAATTGTGATCAATCAAATATTGCTAAGTCCTGG

TATTTACTACCGCTCGGAATTAGACCATAAGGGAATTTCTATCTACACTGGGACTATAAT

ATCAGATTGGGGAGGAAGATCGGAATTAGCAATTGATAAAAAAGAAAGGATATGGGCTCG

CGTGAGTAGAAAACAAAAGATATCTATTCTAGTTCTATCATCAGCTATGGGTTCGAATCT

AAAAGAAATTCTAGATAATGTTTCCTACCCTGAAATTTTCTTATCTTTCCCGAATGCTAA

GGAGAAGAAGAGGATTGAGTCAAAAGAAAAAGCTATTTTGGAGTTTTATCAACAATTTGC

TTGTGTAGGTGGGGACCTGGTATTTTCGGAGTCCTTATGTGAGGAATTACAAAAGAAATT

TTTTCAACAAAAATGTGAATTAGGAAGGATTGGTCGACGAAATATGAATCGGAGACTGAA

TCTTGATATACCTCAGAACAATACATTCTTGTTACCACGAGATGTATTGGCCGCTACGGA

TCATTTGATTGGAATGAAATTTGGAACGGGTATACTTGACGATGACGATATGAATCACTT

GAAAAATAAACGTATTCGTTCGGTTGCGGATCTGTTACAAGATCAATTCGGACTGGCTCT

TGGTCGTTTACAACATGCGGTTCAAAAAACTATCCGTAGAGTATTCATACGTCAATCGAA

ACCGACTCCACAAACTTTGGTAACTCCAACTTCAACTTCGATTTTATTAATAACTACTTA

TGAGACCTTTTTTGGCACATACCCCTTATCTCAAGTTTTTGATCAAACCAATCCATTGAC

ACAAACTGTTCATGGGCGAAAAGTGAGTTGTTTGGGTCCTGGAGGATTGACGGGGAGAAC

TGCAAGTTTTCGGAGCCGAGATATTCATCCGAGTCACTATGGGCGTATTTGTCCAATTGA

CACGTCCGAAGGAATCAATGTTGGACTTACTGGATCCTTAGCTATTCATGCGAGAATTGA

CCACTGGTGGGGGTCCATAGAGAGTCCCTTTTATGAAATATCTGAGAAAGCAAAAGAAAA

AAAAGAGAGACAGGTGGTTTATTTATCACCAAATAGAGATGAATATTATATGATAGCAGC

AGGAAATTCTTTGTCCTTGAATCAGGGTATTCAGGAAGAACAGGTTGTTCCAGCTAGATA

CCGTCAAGAATTCCTGACTATTGCATGGGAACAGATTCATGTTAGAAGTATTTTTCCTTT

CCAATATTTTTCTATTGGAGGTTCTCTCATTCCTTTTATTGAGCATAATGATGCGAATCG

GGCTTTAATGAGTTCTAATATGCAGCGCCAAGCAGTTCCGCTTTCTCGGTCCGAGAAGTG

CATTGTTGGAACTGGATTGGAACGCCAAACAGCTCTAGATTCGAGGGTTTCCGTTATAGC

CGAACGCGAGGGAAAGATCATTTCTACTGATAGTCACAAGATCCTTTTATCAAGTAGTGG

GAAGACTATAAGTATTCCTTTAGTTACCCATCGGCGCTCTAACAAAAATACTTGTATGCA

CCAAAAACCTCGGGTTCTGCGGGGTAAATCCATTAAAAAAGGACAAATTTTAGCGGAGGG

AGCTGCTACAGTTGGTGGGGAACTTGCTTTAGGAAAAAACGTATTAGTAGCTTATATGCC

ATGGGAAGGTTACAATTTTGAAGACGCAGTACTAATTAGCGAACGTTTGGTATATGAGGA

TATTTATACTTCTTTTCACATCCGAAAATATGAAATTCAGACGGATACGACAAGCCAAGG

CTCCGCTGAAAAAATTACTAAAGAAATACCACATCTAGAAGAACATTTACTCCGCAATTT

GGACAGAAATGGAGTTGTTAGGTTGGGATCCTGGGTAGAAACTGGCGATATTTTAGTAGG

TAAATTAACGCCTCAGATAGCGAGCGAATCGTCGTATATCGCGGAAGCTGGGTTATTACG

GGCCATATTTGGCCTTGAGGTATCCACTTCAAAAGAAACTTCTCTCAAACTACCTATAGG

TGGAAGAGGGCGCGTTATCGATGTGAAATGGATCCAGAGGGACCCCCTAGACATAATGGT

TCGTGTATATATTTTACAGAAACGCGAAATCAAAGTTGGGGATAAAGTAGCCGGAAGACA

CGGGAATAAGGGGATCATTTCCAAAATTTTGCCCAGGCAAGATATGCCCTATTTGCAAGA

TGGAACACCTGTTGATATGGTCTTCAATCCCTTAGGAGTACCCTCACGAATGAATGTGGG

ACAAATATTTGAAAGCTCGCTCGGATTAGCCGGGGATCTGCTAAAGAAACATTATAGAAT

AGCACCCTTTGATGAGAGATATGAGCAAGAGGCTTCAAGAAAACTTGTGTTTTCAGAATT

ATATGAAGCCAGTAAACAAACAAAAAATCCATGGGTATTTGAACCCGAGTACCCGGGAAA

AAGCAGAATATTTGATGGAAGAACAGGAGACCCCTTCGAACAACCTGTTCTAATAGGGAA

GTCCTATATCTTAAAATTAATTCATCAAGTTGATGAGAAAATCCATGGACGTTCTACTGG

GCCCTACTCACTTGTTACACAACAACCCGTTAGAGGAAGAGCCAAGCAAGGGGGACAACG

AGTAGGAGAAATGGAAGTTTGGGCTTTAGAAGGATTTGGTGTTGCTCATATTTTACAAGA

GATACTTACTTATAAATCTGATCATCTTATAGCTCGCCAAGAAATACTTAATGCTACGAT

CTGGGGAAAAAGAGTACCTAATCACGAGTATCCTCCAGAATCTTTTCGAGTGCTCGTTCG

AGAACTACGATCTTTGGCTCTAGAACTGAATCATTTCCTTGTATCTGAGAAGAACTTCCA

GGTTAATAGGGAGGAAGTTTGATCGGAATAAATATAAATTCTTTTCTTATTTATGATTGA

CCAATATAAACATCAACAACTTCAAATTGGACTCGTTTCCCCTCAACAAATAAAGGCTTG

GGCTAACAAAAACCTACCTAATGGGGAAGTCGTTGGCGAAGTCACAAGGCCCTCTACTTT

TCATTATAAAACCGATAAACCAGAAAAAGATGGATTGTTTTGCGAAAGAATCTTTGGACC

CATAAAAAGCGGAATTTGTGCTTGTGGAAATTCTCGAGCGAGCGGAGCTGAAAACGAAGA

GGAAAGATTTTGCCAAAAATGCGGGGTAGAATTTGTTGATTCTCGGATACGAAGATATCA

AATGGGATACATCAAACTCGCATGTCCCGCGACTCATGTGTGGTATTTGAAAGGTCTTCC

TAGTTATATCGCGAACCTTTTAGATAAACCCCTTAAGAAATTGGAGGGCCTAGTATACGG

CGATTTCTCTTTTGCTAGGCCCAGCGCTAAAAAACCTACTTTCTTACGATTACGAGGTTT

ATTCGAAGATGAAATTGCATCCTGTAACCACAGCATTTCTCCCTTTTTTTCTACCCCAGG

CTTTGCAACATTTCGAAATCGGGAAATTGCGACAGGAGCAGGTGCTATTAGAGAACAATT

AGCAGATTTGGATTTGCGAATTATTATAGAGAATTCCTTGGTCGAATGGAAGGAATTAGA

AGACGAGGGGTATAGTGGAGATGAATGGGAAGATAGAAAAAGACGAATAAGAAAAGTTTT

TTTGATTAGACGCATGCAATTGGCGAAACATTTTATTCAAACAAATGTAGAACCAGAATG

GATGGTTTTGTGCTTATTACCAGTTCTTCCTCCCGAATTGAGACCCATTGTTTATAGGTC

TGGGGATAAAGTAGTGACTTCGGATATTAATGAACTTTATAAGAGAGTTATTCGTCGGAA

CAACAACCTTGCCTATCTATTAAAAAGAAGTGAATTAGCGCCAGCAGATTTAGTAATGTG

CCAGGAAAAATTGGTACAAGAAGCCGTGGATACACTTCTTGATAGTGGGTCCCGCGGGCA

ACCAACGAGGGATGGTCACAATAAAGTATACAAATCACTTTCAGATGTAATTGAAGGTAA

AGAGGGAAGGTTTCGCGAGACTCTGCTTGGAAAACGGGTCGATTACTCGGGGCGTTCTGT

CATTGTTGTGGGTCCTTCGCTTTCATTACATCAATGTGGATTACCTCTAGAGATAGCAAT

AAAGCTTTTTCAGCTATTTGTAATTCGCGATTTAATCACGAAACGCGCTACTTCTAATGT

CAGGATTGCTAAAAGGAAAATTTGGGAAAAGGAACCCATTGTATGGGAAATACTTCAAGA

AGTTATGCGGGGACATCCTGTACTGTTGAATAGAGCACCTACCCTGCATAGATTAGGCAT

ACAGGCCTTCCAACCTACTTTAGTGGAGGGGCGTACTATTTGTTTACACCCATTAGTGTG

TAAGGGTTTCAATGCGGACTTTGATGGGGATCAAATGGCTGTTCATCTACCTTTATCCTT

GGAAGCTCAGGCGGAAGCCCGTTTACTTATGTTTTCTCATATGAATCTCCTATCTCCCGC

TATTGGGGATCCTATTTGCGTACCGACCCAAGACATGCTTATCGGACTTTATGTATTAAC

GATTGGAAACCGTCGGGGTATTTGTGCAAATAGATATAATAGTTGCGCAAACTATCCAAA

TCAAAAAGTAAACTACAATAATTATAAGTATACAAAAGATAAAGAACCCCATTTTTCTAA

TTCCTATGATGCACTGGGAGCTTATAGACAGAAACGAATCAGTTTAGACAGTCCCTTGTG

GCTCCGATGGAAACTAGATCAACGCGTCATTGGGTCAAGAGAAGTTCCGATTGAAGTTCA

ATATGAATCTTTGGGGACTTATCATGAGATTTATGCCCACTATCTAATAGTGGGAAATAG

AAAAAAAGAAATCCGTTCTATATACATTCGAAGCACTCTTGGTCATATTTCTTTTTATAG

AGAAATAGAGGAAGCCATACAAGGATTTAGTCAGGCCTATTCATACACTATCTAAACAAG

GAAGTTAGATTCGGCGATGCCTTTCGGGGGGCATTCCGATTTCGCTAGTATCATCATTTT

TGCCGCGCGAATCCAGATTGAGATTAAGGAAAGGAAGTTAATTAAATTTTGAATCACTGA

CTCAGGCCCATTGTCGAATCCTACTCAGCAATTGTCGAATCCTACTCAGCCGAAAAAGGG

GGTACTTATGTATGGCGGAACGGGCCAATCTGGTCTTTCATAATAAAGAGATAGACGGAA

CTGCTATGAAACGACTTATTAGCAGATTAATAGATCATTTCGGAATGGGATATACATCCC

ATATACTGGATCAAATAAAGACTCTGGGCTTTCATCAAGCCACTACTACATCGATTTCAT

TAGGAATCGAGGATCTTTTAACAATACCATCTAAGGGATGGTTAGTGCAAGACGCGGAAC

AACAGAGTTTTCTTTTGGAGAAACACTATTATTATGGGGCTGTACACGCGGTAGAAAAAT

TACGCCAATCCGTTGAGATATGGTATGCTACAAGTGAATATTTGAAACAAGAAATGAATT

CGAATTTTCGGATAACGGATCCTTCTAATCCAGTCTATCTAATGTCTTTTTCAGGAGCCA

GAGGAAATGCATCTCAGGTACACCAATTAGTAGGTATGAGAGGATTAATGGCGGATCCTC

AAGGACAAATGATTGATTTACCTATTCAAAGCAATTTACGCGAGGGACTTTCTTTGACAG

AATATATAATTTCCTGCTACGGAGCCCGCAAAGGGGTTGTAGATACTGCTGTACGAACAG

CGGATGCTGGATATCTTACACGTAGACTTGTTGAAGTAGTTCAACATATTATTGTGCGTA

GAAGAGATTGTGGTACTATCCAAGGTATTTCTTTGAGTCCTCAAAATGGGATGACGGAAA

AACTTTTTGTCCAAACACTAATTGGTCGTGTATTAGCAGACGATATATATATTGGTTCAC

GATGCATTGCCGCTCGAAATCAAGATATTGGAATTGGATTAGTCAATCGATTCATAACTG

CCTTTCGAGCACAACCATTTCGAGCACAACCAATATATATTAGAACCCCCTTTACTTGCC

GGAGCACATCTTGGATCTGTCAATTATGTTATGGTCGGAGTCCCACTCATGGCGATCTGG

TCGAATTGGGGGAAGCCGTAGGTATTATTGCAGGTCAATCAATTGGGGAGCCAGGGACTC

AACTAACATTAAGAACTTTTCATACTGGCGGAGTATTCACAGGGGGTACTGCCGACCTTG

TACGATCCCCTTCGAATGGAAAAATCCAATTCAATGAAGATTTGGTTCACCCCACACGTA

CCCGTCATGGGCAGCCTGCTTTTCTATGTTATATAGACTTGCATGTAACTATTCAGAGTC

AGGATATTCTATATAGTGTGAATATTCCCTCAAAAAGCTTGATTCTAGTGCAAAATGATC

AGTATGTAGAATCCGAACAAGTAATTGCGGAGATTCGTGCCGGAACGTCCACTTTGCATT

TTAAAGAAAAAGTACAAAAGCATATTTATTCCGAATCAGACGGGGAAATGCACTGGAGTA

CTGATGTTTACCATGCGCCCGAATATCAATATGGTAATCTTCGTCGATTACCAAAAACAA

GCCATTTATGGATATTGTCAGTAAGTATGTGCAGATCCAGTATAGCGTCTTTTTCGCTCC

ACAAGGATCAAGATCAAATGAATACTTATTCTTTTTCTGTTGACGGAAGATATCTCTTTG

ACCTCTCAATGGCTAATGATCAAGTAAGACATAGACTGTTGGATACTTTTGGTAAAAAAG

ATAGGGAAATTCTTGATTATTCAACGCCGGATCGAATCATGTCCAATGGCCATTGGAATT

TTGTCTATCCTTCTATTCTTCAAGATAATTCGGATTTGTTGGCGAAAAAGCGAAGAAATG

GGTTCGTCATTCCATTACAATATCATCAAGAACAAGAGAAAGAACTAATATCCTGTTTGG

GGATTTCGATTGAAATACCCTTTATGGGTGTTTTACGTAGAAATACTATTTTTGCTTATT

TTGACGATCCACGATACAGAAAAGATAAAAAGGGTTCAGGAATTGTTAAATTTAGATATA

GGACCCTAGAGGACGAATATAGGACTCGAGAGGAAGACTCAGAGGACGAATATGGGACAG

AGAACGAATATAGGACCCGAGAGGAAGAGGACGAATATGAAACCCTAGAAGATGAATATG

GGATCCTAGAGGACGAATATGAAGCCCTAGAAGACGAATATAGGACTGGAGAGAAAGACT

CAGAAGACGAATATGGGAGCCCAGAGAACGAATATAGAACCCGAGAGGACGAATATGGAA

CTCTAGAGGAAGACTCAGAGGACGAATATGGGACTTTAGAGGAAGACTCAGAAGAAGACT

CAGAGGACGAATACGGGAGCCCAGAGGAAGATTCCATCTTAAAAAAAGAGGGTTTGATTG

AGCATCGAGGAACAAAAGAATTTAGTCTAAAATACCAAAAAGAACTAGATCGGTTTTTTT

TCATTCTTCAAGAACTGCATATCTTGCCGAGATCCTCATCCCTAAAGGTACTTGATAATA

GTATCATTGGAGTGGATACACAACTCACAAAAAATACAAGAAGTCGACTAGGTGGATTGG

TCCGAGTGAAGAGAAAAAAAAGCCATACGGAACTCAAAATCTTTTCCGGAGATATGCATT

TTCCTGAAGAGGCGGATAAGATATTAGGTGGTAGTTTGATACCACCAGAAAGAGAAAAGA

AAGATTCTAAGGAATCAAAAAAAAGGAAAAATTGGGTCTATGTTCAACGGAAAAAAATTC

TCAAGAGCAAGGAAAAGTATTTTGTTTCGGTTCGACCTGCAGTCGCATATGAAATGGACG

AAGGGAGAAATTTAGCAACACTTTTCCCGCAAGATCTCTTGCAAGAAGAGGATAATTTCC

AACTTCGACTTGTCAATTTTATTTCTCATGAAAATAGCAAGTTAACTCAAAGAATTTATC

ATACGAATAGTCAATTTGTTCGAACTTGCTTAGTAGTGAATTGGGAACAAGAAGAAAAAG

AGGAGGCTCGTGCTTCCCTTGTTGAGGTAAGAGCAAATGATCTGATTCGCGATTTCCTAA

GAATTGAGTTAGTCAAGTCCACTATTTCGTATACACGAAGAAGGTATGATAGGACAAGTG

CAGGACCGATTCCCAATAATAGGTTAGATCGCACCAATTCCTTTTATTCCAAGGCGAAGA

TTCAATCACTTAGCCAACATCAAGAAGCTATTGGCACCTTGTTGAATCGAAATAAAGAAT

ACCAATCTTTGATGATTTTGTCGGCATCCAACTGTTCTCGAATTGGTTTATTCAAGAATT

CGAAATATCCCAATGCGGTAAAAGAATCGAATCCTAGAATTCCTATTCGAGATATTTTTG

GGCCCTTAGCCGCTATTGTACCTAGTATATCGAATTTTTCTTCATCTTACTATTTACTAA

CGCATAATCAGATCCTGTTAAAAAAATATTTGTTCCTTGACAATTTGAAACAAACCCTCC

AAGTACTTCAAGGGCTTAAATACTCTTTAATAGATGAAAATCAAAGGATTTCGAATTTCG

ATAGTAACATCATGTTGGATCCATTCCATTTGAATTGGCACTTTCTCCATCATGATTCTT

GGGAGGAGACATCGGCAATAATTCACCTTGGACAATTTATTTGCGAAAATGTATGTCTAT

TTAAATCGCACATAAAAAAATCTGGTCAAATTTTCATTGTTAATATTGATTCCTTTGTTA

TAAGAGCAGCTAAGCCTTATTTGGCCACTACAGGAGCAACTGTTCATGGTCATTATGGAG

AAATCCTTTACAAAGGAGATAGGTTAGTTACGTTTATATATGAAAAATCGAGATCTAGTG

ACATAACGCAAGGTCTTCCAAAAGTAGAACAAATCTTTGAAGCGCGTTCAATTGATTCAC

TATCGCCGAATCTCGAAAGGAGAATTGAGGATTGGAATGAGCGTATACCAAGAATTCTTG

GGGTCCCCTGGGGATTCTTGATTGGAGCTGAGCTAACCATAGCCCAAAGTCGTATCTCTT

TGGTTAATAAGATCCAAAAGGTTTATCGATCCCAAGGGGTACAGATCCATAATAGACATA

TAGAGATTATTATACGCCAAGTAACATCAAAAGTGCGGGTTTCCGAAGATGGAATGTCTA

ATGTTTTTTCACCTGGGGAATTAATCGGACTATTACGAGCAGAACGAGCAGGACGAGCTT

TGGATGAATCGGTCTATTATCGGGCAATCTTATTGGGAATAACAAGGGCTTCCCTGAATA

CCCAAAGTTTCATATCTGAAGCAAGTTTTCAAGAAACTGCTCGAGTTTTAGCAAAAGCTG

CCCTACGAGGTCGTATTGATTGGTTGAAAGGCCTGAAAGAAAACGTAGTTCTGGGGGGGA

TTATACCTGTTGGTACCGGATTCCAAAAATTTGTGCATCATTCCCCACAAGACAAGAACC

TTTATTTCGAAATTCAAAAAAAAAATCTATTCGCGTCGGAAATGAGAGATATTTTGTTTC

TCCATACAGAATTAGTTTCTTCTGATTCTGATGTAACAAACAATTTCTATGAGACATCAG

AACCCCATTTATACGATTTAAGGATACATAAAGCAGATTTTTTTATTTAAACTAGACTTT

TGACCTTAGAACACTAACAGGTCAGATTTTGATTTTTATTAATAAGTAAAGAAGTCAGTT

AATTCATTAAGGTTACGTTTATACCATGTAGAAGGTTACATCGGAACAATTATTATTTAT

TTCAAGCTATTTCGGCTCTTTCTTAATTTTCAAAAAGAAATAAATTCCGTAATGGAAAAA

AAAGAAAAAATCAAAAGGAAGTGTGGAAAAAATGACAAGAAGATATTGGAACATCAATTT

GAAAGAGATGATAGAAGCGGGAGTTCATTTTGGTCATGGTATTAAGAAATGGAATCCTAA

AATGGCCCCTTACATCTCGGCAAAGCGTAAAGGTACTCATATTACAAATCTCGCTAGAAC

GGCTCGTTTTTTATCAGAAGCTTGTGATTTAGTTTTTGATGCAGCAAGTCAGGGAAAAAG

CTTTTTAATTGTTGGTACCAAAAAAAGAGCAGCGGATTTAGTAGCATCAGCTGCAATAAG

GGCTCGTTGTCATTATGTTAATAAAAAGTGGTTCAGTGGTATGTTAACGAATTGGTCGAT

TACGAAAACTAGACTTTCTCAATTTAGAGACTTAAGAGCAGAAGAAAAGATGGGAAAATT

CCACCATCTCCCAAAAAGAGATGTGGCAATCTTGAAGAGAAAATTATCTACCTTGCAAAG

ATATCTCGGCGGGATCAAATATATGACGAGGTTGCCGGACATTGTGATCGTCCTTGATCA

GCAAAAAGAGTATATAGCTCTTCGGGAATGTGCCATTTTGGGGATTCCTACTATTTCTTT

AGTCGATACAAATTGTGACCCAGATCTCGCAAATATATCGATTCCAGCCAACGATGACAC

TATGACTTCAATTCGATTGATTCTTAACAAATTAGTATTTGCAATTTGTGAGGGCCGTTC

TCTCTATATAAGAAATCGTTGATTAAGAAGAATAGTTCATTCTTGGGTAACTGCGTAGAT

TTATGGGATCACTTACTATTCTTTTTTGTTTTGCATAGATAAAAGAAGGGGAATATTGAT

ATATATTAGAGGGTATTGATATATATTATCATCTGATGTGATTTCTTGGTATCCTAAATA

TAAGATTAATACTTCAAGTTGCTGAGTTGAGAAAGAGATGGTTGAATCAAAAGAATTCCT

TTTTTGAAGTTCAATTTTTATCAGAGGACAATATGAATATTATACCATGTTCCGTTAAAA

CACTCAAGGGGTTATATGATATATCGGGTGTAGAAGTAGGCCAACACTTCTATTGGCAAA

TAGGAGGTTTCCAAATTCATGCCCAAGTACTCATCACTTCTTGGGTCGTAATTACTATCT

TGCTAGGTTCAGTTATCATAGCTGTTCGGAATCCACAAACCATCCCGACCGACGGTCAGA

ATTTCTTCGAATATGTGCTTGAGTTTATTCGAGACTTGAGCAAAACTCAGATTGGAGAAG

AATATGGTCCCTGGGTTCCCTTTATTGGAACTATGTTCCTTTTTATTTTTGTTTCGAATT

GGTCGGGTGCTCTTTTACCTTGGAAAATTATACAGTTACCCCATGGGGAATTAGCAGCAC

CCACGAATGATATAAATACTACTGTTGCTTTAGCTTTACTCACATCAGCGGCATATTTTT

ATGCGGGTCTTAGCAAAAAAGGATTGAGTTATTTCGAGAAATATATTAAACCAACTCCAA

TTCTTTTACCAATTAACATCCTAGAAGATTTCACAAAACCATTATCGCTTAGTTTTCGAC

TTTTCGGGAATATATTGGCGGATGAATTAGTCGTTGTTGTTCTTGTTTCTTTAGTCCCCT

TAGTAGTCCCTATACCGGTCATGTTTCTTGGATTATTTACAAGCGGTATTCAAGCTCTTA

TTTTTGCAACGTTAGCCGCAGCCTATATAGGTGAATCCATGGAAGGTCATCATTGAATTG

ACTAGTTTTCAAAATAGTCTTTTTTTAGCTTAGCTCAATTCATGCATGGTTCCAGATAAT

CCGCTTGGTTGGAAAACTAAATAGTTAGAAATGCGTATGAATATACAACCTAGAGTTGTA

GGAGAGAGAATAGACTATATTACGTGTCAAAGTATATATGCATTAAGGGGGGCGGAGTCA

GGCTAGATCTATATCCTTAATGTCTATAAGCCAGTCATCTTTTGTGCGGGTTTTTAAGGA

ATGATTTTAGAATCCGATTCAATAGAAAATGAGAAAATACGCAAAATAGAAGAAACAAAT

GTATATGGGATATTATATATTCCTAAGTTAGATTCATTATCTAATCCGATATATGGAATT

GGATTCCATATCCAGTTCTATGCAGCATATTGTTATCAATTGTATATCTTGATTTAATTC

CTATTGGATTTGGATTAGGTCGATTTCAATAGGGGTTCTTCCTCTATTTCGTCTTTTATT

ATGTTAGATGAAGGGGAAAAAATAGGAACTCAAGGATATCGAAGAGTAAAAAGAAGAATG

GAATGAAAGAGTGGTTGGTTGGAAAGAAAGAGAAATAGAATAATGAGTACACAAACCTCT

AATGATTAGAAACTAAAAATGAGATCTCGAAGTAGTTCGGACAATTCAGATTATCATTTA

TTTGTACTTTTTAGTTACTTCTCCCCAATAGAGCTTAGAAGTAAGAATTTCTTGGTTGAT

TGTATCCTTAACCATTTCTTTTTTTTGACACGAGGAACTCACCATGAATCCACTAATTGC

TGCTGCTCCGTTATTGCTGCTGGATGCCGTAGGGCTTGCTTCTATTGGGCCTGGAGTTGG

TCAAGGTACTGCTGCAGGACAAGCTGTAGAAGGTATTGCGAGACAGCCAGAAGCAGAAGG

TAAAATACGAGGTACTTTATTGCTTAGTCTAGCTTTTATGGAAGCTTTAACAATTTATGG

ACTAGTTGTGGCACTAGCGCTTTTATTTGCGAACCCTTTTGTTTAATCCTAAAAAAGAAA

ATGAGTCCTTTAGATTAGATACTTTTTTCTTTTTTAGTAAATTGGTATTTGCTTCTGCAA

TTCCAATTATATCAATACTTTACTCCTATTTATTACTCCTGGAATTACCTATTTATCGGG

ACAGACAATACCCCACCCCAGGAAGGGCTGATTTGAGGATGATCAATTTAGAGGATATGC

TCGCCTTCTTCCTTCCCGTCCTTAGTTTAGGACAGTGGAAAGTCTTTTTCCTTTTATTTT

AGGAATTTTTGGAACATTTCAACAAAGGAGTCTTTCACAGGTCAAACGAGACCTAAGACT

TAATCTAAAAGAAATTATTAGATTGAATCTATTTGCATTAAAAAACCGATCAAAAAGGGC

GAGCGAAGTAAGTGATCAAAAACTTTGCTCTTTGTTCGTCCTATCTATAAGAGGAGAGCA

TATGAAAAATGTAACCCATTCTTTCGTTTTTTTAGCTCACTGGCCATCCGCTGGGAGTTT

CGGGCTTAATACCGATATTTTAGCAACAAATCTAATAAATCTAACTGTAGTGGTTGGTGT

ATTGATTTTTTTTGGAAAGGGAGTGTGTGCGAGTTGTCTATTTCAAGAATAGATTGATCT

ATCCGGCTGCACTTTAAAATATTTTTTAGTATTTTTTGGATAAATAAGAAAAGGTGCACG

ATCTCGACGAATTACTTCTGAATAAATTCAGAAATCATATGGAAGACCATAGCATTTCGC

GACTCATTGGTAAATCAACTTTGATTCTCTATAGACCAATAATGTGAGACCATTAACACG

GTTAAAGCTAAACTGCTTGAAGTCCAGGCAAAAAGGGGTACTCTTTCTACAACTATATTA

GTATTAGTACCAAATGCTTTAAACGGGAAATAGCTAATGTAGAATTTATCTGATATAAAA

CACTCATATCGATAAAATGGTTTGAACTATTTACTAGAAGGGCACCCTGCCCTTTTTCCA

ATGCCGAATCGACGACCTATGTATAAAAAAAGAGAAATTTTTTGGATTTGAAGAAAAAAA

AGAATTCTATCAATTTTCATTTTCCATTTATTTAGTTTCTTAATGAAATTGAAATTATTA

ACTAAAGGGCAAATACAAATAAAGAAACAACTTTGCTGACCATGATAGATTTTTATCTAG

GCGGAAGAGTCCTCTTAATATTTATCTAGTCTTATATGGGTTTCGGTATATTGAAATATA

AACAGAAAAGAGAAGATAGAGGATAGGCTCATTACATAAAAAAGATATGGAAATAGCCAT

AGCAAAAAAAAAAAAGGAGCGTGAGAGCCAAATGAATCGAAAGATTCATGTTTGGTTCGG

GAAGAGATCATAAAAATTGTAAACTTAATAGCAAGATAATCTACTTTCATTAAAAGATTT

ATTAGATAATCGAAAACAGAGAATCTTGAGTACTATTCGAAATTCGGAAGAATTGCGTAG

AGGGACCATTGAGCAGCTCGAAAAAGCTCGGGTTCGATTACAGAAAGTCGAACTAGAAGC

GGATGAGTATCGAATGAATGGATACTCTGAGATAGAACGAGAAAAAGCAAATTTGATTAA

TGCTACTTCTATTAGTTTGGAACAATTAGAAAAGTCTAAAAATGAAATCCTTTATTTTGA

AAAACAAAGGGCGATGAATCAGGTCCGACAACGGGTTTTCCAACAGGCCGTACAAGGAGC

TCTAGGAACTCTGAATAGTTGTTTGAATACCGAGTTACATTTCCGTACGATTCGTGCTAA

TATTGGCATTCTAGGGGCCATAGAATCGAAGAATTAAATTAATTAGACCTTGAACTTCTA

CTTTCGTTTAGAATTTAGGCATTATTTTTCCCTTGCTTCCGAAAAAAGAGTCAAGAAACA

CTAATGGCAACCCTTCGAGTCGACGAAATTCATAAAATTCTCCGCGAACGTATTGAACAA

TATAATAGGAAAGTAGGGATTGAGAATATCGGTCGCGTAATTCAAGTGGGGGATGGGATT

GCTCGTATTATAGGTCTTGGTGGAATAATGTCAGGTGAATTAGTCGAATTTGCAGAAGGG

ACTAGGGGTATTGCTCTGAATTTGGAATCCAAAAATGTTGGGATTGTATTAATGGGCGAT

GGGTTGATGATACAAGAGGGAAGTTTTGTAAAAGCAACAGGAAGAATTGCTCAGATACCC

GTGAGCGAGGCTTACTTGGGTCGTGTTATAAATGCTCTGGCTAAACCTATTGATGGGAGA

GGCGAAATTGTAGCTTCGGAATCTCGCTTAATTGAATCTCCTGCTCCGGGTATAATTTCC

AGGCGTTCCGTATATGAACCCCTTCAAACAGGGCTTATTGCTATCGATTCGATGATCCCC

ATAGGGCGCGGTCAGCGAGAGTTAATTATTGGGGACAGACAGACTGGCAAAACAGCAGTA

GCCACAGATACAATTCTCAATCAAAAAGGGCAAGATGTAATATGTGTTTATGTAGCTATC

GGTCAAAGAGCATCCTCCGTGGCTCAAGTAGTAACTACTTTCCATGAAGAGGGGGCCATG

GAATACACTATTGTAGTAGCTGAAATGGCGGATTCACCTGCTACATTACAATACCTCGCC

CCTTATACGGGAGCAGCCCTGGCTGAGTATTTTATGTATCGCGAACGGCATACTTTAATA

ATTTATGATGATCTCTCCAAACAAGCACAAGCTTATCGCCAAATGTCCCTTCTATTAAGA

AGACCCCCCGGCCGCGAAGCTTATCCAGGGGATGTTTTTTATTTGCATTCACGGCTTTTA

GAAAGAGCCGCTAAATTAAGTTCTCTTTTAGGCGAAGGAAGTATGACCGCTTTACCAATA

GTTGAGACTCAATCTGGAGACGTTTCCGCCTATATTCCTACTAATGTAATCTCCATTACA

GATGGACAAATATTCTTATCCGCGGATCTATTTAATGCCGGAATTCGACCTGCTATTAAT

GTGGGTATTTCTGTTTCCAGAGTAGGATCCGCGGCTCAAATTAAAGCCATGAAACAAGTA

GCTGGCAAATCAAAATTGGAACTAGCTCAATTCGCAGAATTACAAGCCTTTGCACAATTC

GCCTCTGCTCTCGATAAAACAAGTCAGAATCAATTGGCAAGGGGTCGACGATTACGGGAA

TTGCTTAAACAATCCCAATCAAACCCTCTCTCAGTGGAAGAGCAGATAGCTACTATTTAT

ACCGGAACGAGAGGATATCTTGATTCGTTAGAAATTGGACAGGTAAAGAAATTTCTGGAT

GAGTTACGTAAACACCTAAAAGATACGAAACCTCAATTCCAAGAAATTATATCTTCTAGC

AAGACATTCACCGAGCAAGCGGAAATCCTTTTGAAGGAAGCTATTCAGGAACAGCTCGAA

GGGTTTTCCCTTCAGGAACAAACATAAATTTTGCATGTCTACTCTTGTTAGTAGAAGAGG

AATCAAAGATTTTTCATTTGAATCATGCAAAAAGTTTTCTTAGTTTTTAGTATAGTTATT

TAAAGAATAGATAGAAATAAGATTGCGTCCAATAGGATTTGAACCTATACCAAAGGTTTA

GAAGACCTCTGTCCTATCCATTAGACAATGGACGCTTTTTTCATATTTTCTTCTTTCTTT

TTTTTTTTTTCTTGTGATAAAAAACTCTTAGACCGAAACTCTTTTAGGAAAGAAAAATAA

ATCCATATACAAATGGATGATGCATATATCATAAAGAAGGAATATGGAGCGGGTAGTGGG

AATCGAACCCGCAACCCCAAGGTTATGAGCCTTGTGAGCTACCAAACAGCTCTATCCTCT

TAGCGGGGAACTAGGGGGTTGGATACGCCCCTCTACCATATCTATACAAATAGAATAGTC

CATTTATACAGAATGGTAAAGAGGGCTCTTCTACGATCATCCATTCTAGAAATCCATACA

AATATGAAAGGGTATTTTATCCTTACCAACTGGATCTTGTTGCACCCGGTAACAAACATG

CATAAACCATTTCTCGAAGTATGTGTCCGGATAGTCCAAAGTCTCGATAGTTAGCTCTAG

GTCTTCCGGTCAAAAAACAACGTCGATGAAGGCGTGTAGGTGCACTATTACGTGGTAGGG

ATTGCAATTTTTCTCGCATTTTCGTTTTTTCACTCAAACTCAAGGGGGAAACTTTGCTTC

TTATCTTTTTTTTTGAAGATCGACGAATCAAATGATATTTCTGTTCTAATTTCTGCCGCT

TCTTCTCCCTCTGAATCAAACTTTTTTTTGCCATAATGTGCCGTTCCTATTATTACCAAG

TATATGGTTCTAATCCTAGATGGAAAAATAAATAGAAAAAATCTAAGAAGGCGGATCCTC

CCTCTCCATCAAGAGTAATGAACTAGGTGCTGGTACAGTACAAAACTAAATTAACCAAAC

TTGCCTGATGTTGAGGCAATCAAGAAAGCTGCATAAGTGAATATATAACCCACGGAAAAG

TGGGCTAATCCAACCAATCTTGCTTGCACAATGGAAAGAGCCACGGGCTTATCTCTCCAG

CGAATTAAATTAGCCAAAGGTGTGCGTTCATGAGCCCATGCTAAAGTCTCAATTAATTCC

TGCCAATATCCACGCCAGGAAATTAAGAACATAAATCCAGTAGCCCAAACAAGATGTCCA

AATAAGAACATCCACGCCCATACCGATAAACTATTCATCCCAAAAGGATTATATCCATTG

ATAAGTTGTGAAGAGTTTAACCATAGGTAATCTCTTAACCATCCCATCAAATAAGTGGAG

GATTCATTAAATTGTGAAACGTTGCCCTGCCATAATGTGATGTGTTTCCAATGCCAATAA

AAAGTAACCCATCCAATGGTATTTAACATCCAGAAAACTGCCAAATAAAACGCGTCCCAA

GCAGAAATATCACAAGTACCGCCGCGCCCTGGGCCGTCACAAGGAAAACTATACCCAAAA

TCCTTTTTATCCGGCATTAATTTGGAACCGCGTGCATCTAAAGCACCCTTTACTAAAATC

AATGTAGTTGTATGCAAACCTAGAGCAATAGCATGATGAACCAAGAAATCTCCAGGTCCT

ATTGTTAAGAAAAGAGAATTACTATTCTCATTAACAGCATTCAACCATCCGGGCAACCAT

AGGGTTCGACCTGCATTGAAAGCGGGGCCGCTCGTTGAAGATAAGAGTATATCGAACCCA

TATGTCGTCTTACCATGAGCAGATTGTATCCATTGGGCAAATATAGGTTCGATCAAGATT

TGCTTTTCTGGAGTACCAAAAGCAAGCATGACGTCGTTATGAACATAAAGGCCCAAGGTA

TGGAATCCTAGGAAGAGGCTAGCCCAACTTAAATGAGATATGATAGCTTCTTTATGGTCT

AACATTCTTGCCAATACATTATCCTCATTCTGTTCCGGATTGTAATCCCTAATGAAAAAA

ATAGCTCCATGAGCAAAAGCCCCTGTCATGATGAACCCTGCAATATATTGGTGATGAGTA

TATAAAGCAGCTTGAGTGGTAAAGTCTTGTGCTATGAATGCATAAGCAGGTAAAGAGTAC

ATATGTTGAGCTACTAAGGAAGTAATAACCCCTAAAGAAGCTAGAGCAAGACCTAATTGA

AAATGAATCGAATTATTGATTGTGTCGTAAAGGCCCTTATGCCCACGCCCTAATCGACCC

CCCGGAGGAGTATGTGCTTCTAAAAGATCTTTGATACTGTGCCCAATTCCGAAGTTAGTT

CGATACATATGACCGGCAATGAGAAAAATAAATGCAATAGCTAAATGATGGTGAGCAATA

TCGGTCAGCCACAAACTTTGTGTTTGTGGATGGAATCCCCCAAGAAGAGTTAGAATGGCA

GTTCCGGCTCCTTGAGCGGTACCAAATAAATGATTACTCGAATCAGGGTTTTGGGCATAA

AGATTCCACTGACCCGTCAGAAGGGGTCCCAACCCCTGGGGATAGGGTAATACATCTAAG

AAATTATTCCATCGAACGTACTCCCCCCTGGATCCGGGAATAGCGACATGAACTAAATGT

CCTGTCCAAGCCAAAGAACTTACCCCGAAAAGTCCTGACAAATGATGATTGAGACGAGAT

TCCGCGTTTTTGAACCACGAAAGGCTTGGTTTCCATTTGGGTTGTAGATGTAACCAACCC

CCTATTAAGGATAGCGTAGAAAGAAATAATAGAAACAGAGCTCCAGTATAAAGATCTCCA

TTGGTGCGTAATCCAATTGTATACCACCACTGATAAACCCCAGAATAGGCGATATTCACT

GGACCGGCAGCACCTCCTCGAGTAAAGGCTTCCACAGCGGGTTGACCAAAATGAGGATCC

CAAATCGCATGAGCAATAGGCCTTACGTGTAAAGGATCCTGTATCCATGATTCAAAATTT

CCTTGCCAAGCTACATGAAACAGATTTCCGGATGTCCATAGAAAGATTATTGCTAACTGC

CCAAAGTGAGAAGCAAAAATGTTCTGATAAAGACGTTCCTCAGTAATATCATCATGACTT

TCGAAATCATGTGCGGTAGCAATACCAAACCAAATACGACGAGTAGTGGGGTCCTGAGCT

AAGCCTTGGCTAAACCTGGGAAATCTTAATTCCATAATGCCTTTCAAATCCTCCTAGCCA

CTATCCTACTGCAATAATTCTCGCTAAGAAGAATGCCCATGTTGTGGCAATTCCACCCAG

AAGGTAATGGGTTACTCCTACAGCACGTCCTTGTATAATGCTCAAGGCTCTAGGCTGAGT

AGCAGGAGCAACTTTTAATTTGTTATGAGCCCAAACGATAGATTCAATGAGTTCTTGCCA

ATAACCACGGCCGCTGAATAAAAACATTAAACTGAAGGCCCAGACAAAATGAGCACCTAA

GAAAAAAAGACCATATGCAGATAATGAAGAACCATAAGACTGAATTACTTGGGATGCCTG

TGCCCACAAGAAATCTCGGAGCCACCCATTAATCGTAATGGAACTCTGTGCAAAGTTTCC

CCCTGTGATATGAGTTACCATCCCTTCATCGCTTATAGTACCCCAAACATCCGACTGCAT

TTTCCAACTGAAATGGAAAATGACTACCGAAATTGCATTGTACATCCAGAATAGACCTAA

GAAAACATGATCCCAGGCGGATACTTGACATGTTCCCCCTCTCCCAGGCCCATCGCAAGG

GAAGCGAAAACCAAGATTTGCTTTATCGGGTATCAAACGGGAACTGCGAGCAAATAAAAC

ACCTTTCAAAAGTATTAATACAGTCACATGGATGGTAAATGCGTGAATGTGATGGACTAA

AAAATCTGCGGTTCCTAATGGAATAGGTAACAAAGCTACTTTGCCGCCTACTGCTACTAA

CTCGCCACCTCCCCACGTTAAGCTGGTACTTGTTGTTGCACCAGGAGCTGTTACGCCAGG

CGCGTTAGCATGGATATTTTGTACCCATTGAGCAAAGATGGGTTGTAATTGTATGGCGGT

ATCCGAAAACATATCTTGGGGACGGCCTAAAGCACTCATGGTATCATTATGAATGTACAA

GCCAAAACTGTGAAAACCTAGAAATATACATACCCAGTTAAGGTGGGATATGATTGCATC

GCGGTGTCTAAGGACGCGATCTAATAGATCGTTGTATCGAGTAGTTGGATCATAGTCTCT

TACCATAAAAATGGCTGCATGTGCAGCAGCACCGACTATTAGAAATCCGCCAATCCACAT

GTGGTGTGTGAACAAGGAAAGTTGTGTACCATAGTCAGTAGCTAGGTATGGATAGGGGGG

CATAGAGTACATATGATGAGCTACAACAATGGTTGTAGAGCCTAGCATAGCTAGGTTAAG

AGATAATTGAGCGTGCCATGACGTTGTTAGGATTTCATAGAGACCCTTATGGCCTTGTCC

TGTAAATGGGCCCTTATGAGCTTCCAAAATATCTTTAAGTCCATGACCAATACCCCAGTT

GGTCCTATACATATGACCTGCGATCAGGAAAAGAATAGCAATAGCTAAATGATGGTGCGC

AATATCGCTCAACCATAGACCTCCGGTTATTGGGTCTAGTCCTCCGCGAAAACTCAGAAA

TTCTGCGTATTTGGACCAATTCAAGGTGAAAAAGGGGGTTGCTCCTTCGGCAAAACTAGG

ATAAAGTTGAGCCAAAAGGTCGCGATTCAAGATAAATTCATGAGGAAGTGGTATCTCTTT

AGGATCAACCCCAGCGTCAAGAAATTGGTTAATTGGTAAAGATACATGGATTTGGTGTCC

CGCCCAAGAAAGAGACCCAAGTCCTAATAACCCCGCTAAGTGGTGATTCAACATGGATTC

TACATCTTGGAACCAGGCCAATTTGGGAGCGGCCTTGTGATAATGGAACCAACCAGCAAA

AAGCATTAACGATGCAAAAATCAATGCACCGATTGCGGTACAATAGAGTTGTAATTCATT

AGTTATTCCAGATGCTCGCCAAAGCTGAAAAAACCCAGAGGTTATTTGGATTCCTCGGAA

ACCCCCGCCTACATCACCATTCAATATTTCTTGCCCTACTATTGGCCAAACTACCTGAGC

ACTGGGTCCAATGTGAGTAGGATCACTTAGCCATGCTTCATAATTGGAAAAACGGGCACC

ATGGAAGTACATGCCACTCAACCAAAGAAAGATAATGGAGAGTTGACCGAAATGAGCACT

AAAGACTTTTCGAGAGATCTCCTCCAAATCACCGGTATGACTATCGAAATCGTGAGCATC

AGCATGTAGGTTCCAGATCCAAGTGGTAGTATCAGGGCCCTTAGCTATTGTTCTTGAGAA

ATGGCCGGGTTTGGCCCATTCCTCAAAAGATGTTTTTACAGGATCCCTATCCACAACAAT

TTTTACTTCTGGTTCCGGCGAACGAATAATCATTAAGTCCTCCTCTTTCCGGACAAGACA

TACAAAGAGACCCGCCAACTTTTTAGTGAACCTTTGAAAGATAGATATTATGATTAGTCC

TTTTCTTTACTATCTACCGTCCTTCTATTTTTTTTAGTTATTCACTGGAGCAATTATATA

TTGAAGTCAATCCGAGGCAAGTGTTCGGATCTATTATGACATAAGGATTAGGTGCCTAAC

GGACATTGTTTATTTTGGATTTCCCGACGTACTAAAAAAACCTTTTTTAATTTACGAAGC

TAGTGTATTTTTTTAGGGTATAAGCTCCTATCTACATCTACTTTCCTTGAGTATAGATTT

TTTTATTCGATTCCAAATTCCAAGATAACTCATTAGAATTATTAATAAGACGGTCCTGAT

ATATTAGCAATATTTAGATCGCCCCCTTTATTCGCTTTATTACTTCTATTCTAGACCCTA

TCGTTTATCCTTATGAAATATAATAAAAAATAGAAGGTAGAAGAAAGGGATATAATGAAA

TTCTTGATTCGATTTACAACCAAACCCCCATTTTATGAAAAAGGAGAGTGGTCTTATTCA

AATTCAAAGCGCTTCGTAATCTTCAACCAGTTCTGTGCTTCAATATAATTTCCCGGAGTA

AGCGCTATAGCTTGTTTCCAATACTCAGCAGCTTGATCAAACCAAGCTTCCGCAATTTCC

GAATCACCCTGTAGAATGGCCTGTTCTCCTCGGTCGGAATAGGCAGTTCCTTCCCCTAGA

ACCGTACTTGAGAGTTTCCTACCTCATACGGCTCAGAAATTGCTATCCTAATTTCCCCTA

TCTTAACTGAATTCGATTTCTCAAAAATCGATCCAATTTCTTCTTGGGTTAAGCAGAAGA

AGTTAATTACCTAAGTTTCAAACCCTAATTTTGATCAATAATCAGTTTGATCTTTTCTCC

CACCTTCAGAAGAATGAAGCATAGATAGACCTATAGCCTTCGTTCGAATTTTCTGAAAGG

TAACTATCTCGGTTTCTTATATGAAATTTCTATAGAATCCTTGAAAAAGACTTTTTCCAT

AAGAAAGAAAAAAGAACTTACTATCTTTGGGATCTGATACTACACCGCTGCTTATTCCTT

AGTGGATCGGCTCTATTACATAAGCAGATTCCTAAATTTTGCCCCATATCATGGGATAAG

TAAGCAGTTTTTTTTAGTTGTATCGACCCAGTCGCTCACTAATGGATCTTTACGGTGCTT

TCTCTATCAATTTGGGCTTTATCCATAGAGTAGTATAGGCCATACTTTCTTCCTATTTTG

ATTCTCGTGAAGTGTCCTTCCTTCCTACAGCTGATAGGGCAAAATCGTTGTTTTGACGAT

CCCTATGTAGAAAGCCCTTTTTCTAGTATTTACTAGAAAATTTGATCCTTTCTTTTTCTT

CTTTCTATAGTGGAGATAGTCGCACGTAATGACAGATCACGGCCATATTATTAAAAGCTT

GCGGTAAGAAGGGGTTTCGTTCTAGTGCCCGGAAATAATATTCCAAAGCCTTTGTATGCT

CTCCATTGCTTGTGTGTATAAGGCCTATATTATAGAGTATATAACTTCGATCATAGGGAT

CAATTTCTAGTCGCGTAGCTTCATAATAATTCTGCAAAGCTTCCGCATAATTTCCTTCGG

ATTGAGCCAACATCCGTTACGGTCGTTCATTCTATTCAAAAAATCTCCGTTCCAAAACCG

TACATGAGGTTTTCATCTCATACGGCTCCTCCCTTCTGTACATAGTACTAAGCGAAAAAT

CTATAGAATAAAAATAGAATTAGTCCCATCTTATTATGAACCGAAAGGGGCTGGTATTTT

TCCAAGAAATCTCTAGCCAACCTTCCCGCAAGAGGTTTTTCTTAACACCAATGAATTCTA

TTAATGCTAGAGGAAAACGATAGCTCCAAGAATTTCTTTGTTCTCAACGCCTCCTATTTA

GAGGAATTAGCCACTTCAACGATCTTTGATGGTTATAGGGGTATCCAAAGTACAAACCTG

ATGGTTGTTTGTTATCCCAACCATTCTTCCCAGCCCTGATACCGATCAGGAAAGGGCTAA

TTTCTAACAAAGTTTTTCTCTTGTTGATTCCTATTTCTAGGTGTAGTGCTTTTCTCCCCT

ATGCTGCCTATTGGTACTAGTAGAGTAGGATTGGCCTGTAATACAGAACCTATCCTGTAG

GTGTAACCTTTCGCTCAATACTCAAATCTACAATTGAAGCATCTGAGGCCGCATCAATCG

AGGATACACGACAGAAGGAATTGTTAGTTCACCTCACCTTCCCCAAGCGTGGGTTTCCTT

TACTAATTTTGTTCTCTCTATGCGAACCCCCTCTCTTTCTCGTAAGACTGAGGTGTAGGT

AGGGCTAAAAAAAAAAAGAGTCAAATCGCACCATCTCTATAATAAGTAAATGCCCTTTTT

TCCCCTGAGGTTGTCGGAATTATTCGCAATAAAATATTGGCTACAATTGAAGAGGTCTTA

TCAATGAAATTTCCATTTATACGGGATCTAGGCATAATTCCCAACCCATTCTATATAGAA

TTGTTTTCATTTCTTCACAAAATAACATAAAAACAAAACATTGATTCTTATAAATCGATC

ATATGCTCTAAATGGATAAGAGAGGTATTTCCGCTCAGCTCAAATTGTCTCCTTTTCCTC

TGTTTGGACAAGAAGAGATATGAAAATTGACTAAGATTGGATTTCATTCCACTTTTCTAT

TTCTCACAATAACTTCTCTCATCAGCTATTTCGCGTTTCAAGTCATTAATTGTCTCATAC

CCTTTTTTATGGCGTAGCGTACTTATGCAAACAGAATTCTAGGGTTCCTTTTTATGGAAT

AAGAAGAATTCTTCCATCTCTTTTTCTTTGGTTTGAACCCAAACTAAAACTTTTCGAGGG

AGCGGAATTCCTAGTAAAAAAATCCTGGATCCTTCCACTTAGATGAAAAGGAATTTTTCC

AATAGAACCATGGAACCCCCGAGCCGTTGTGGTTGTACCTGTACTGCAGGAATAGGAAAA

CTCGCTATTCACTTAGTTTATTTTCCATAATAAGATTATGTAGGAGAGATGGCCGAGCGG

TTCAAGGCGTAGCATTGGAACTGCTATGTAGACTTTTGTTTACCGAGGGTTCGAATCCCT

CTCTTTCCGTTTCTCTTAATTGATCAACGTTAACGATCACAATGTATCAAATCAAATAAC

AATTTATTCCAGCAATAATACCTTTATTTAATAGAAATTTTTTATAGTAAATTACTGTGG

TATGTAAAATACACATAGAGGAAAGAACAAAGAAAAAGGATCCTAGGGTTAATCCATTTA

TGCTAGTTGAATGGAAAATACGAATTAAGGGCCTTAGGTCGATTTAGTTCGGGGGAAGGG

GAAGAAAATTCTATGAACTTTTCCTTTTTCGTTAAGTTCAAGTCTGACGAGAGTAATATT

CTACAACTAACAACTCATTTATTTTGAGACCGACCCACTTCCTATCTAGGATTTTTTTAA

CTAGTCCTTTATATTGCAATGTGTCAATCGTCAAATGCTTTGGCAATTTCCCCGGGTCGG

ATGAAGCAATAGAATTTTGAATCAGACGTTTTGATCTTTGGTTATCCTTCGTAGTAATAA

TATCTCGGGGTTTGCAACGAAAACTTGGTATATCGACTATACGACCATTAACTAAAATAT

GTCTATGGTTAACTAATTGCCGGGCCCCAGGAATGGTTGAAGCCATACCCAATCGAAAAA

GGATATTATCCAAACGCATTTCAAGTAATTGTAGTAAAACCTGACCTGTTGACCTTTTTG

CTTTTCCAGCGATATGTACATATCTAAGTAATTGTCGTTCTGTCAGACCATAATGAAAAC

GCAATTTCTGTTTTTCTTGAAGACGAATACGATATTGCTCTTTTTTCCCAGAATGGAATT

TCTTTTTCAGATTACTTCCGGATTTAGGTGTTTTTCTAGTGAGTCCTGGTAAAGCTCCCA

GACGGCGTATTTTTTTTAAACGAGGTCCTCGATAACGGGACATGAAGACTCCTTGTTTTA

TTGAAATTTCATTTTACACAATTAATTTCATTGTATTTACATTACAGAATACATCAAAAT

TAAAACTGAATTAAACTAAAGGATAAACAGAGTAAAATCTACTAAAGTACCACAAAAAAT

GGAATTTCATCAACATCTGGATTTTTGTATATATATTATTTATTTTATTGTTTTGTATCT

AGCAAAATTGTAAGGTAGAACCACATAATAGATCCTGATTCTCCATTTAATTCGGAGAAA

AAGAGAGATTCTTGTTCATGGAACATCGATATAGAAAAAAGCCGACTATCGGATTTGAAC

CGATGACCCTCGCATTACAAATGCGATGCTCTAACCTCTGAGCTAAGTGGGCTTACATAA

CAGAAATAGTGTAACAAATAGAAATATGTATATAGGAAATCCGTAAAATGTCAGATCTTA

ATTATTAATCTTAGCTATTAACTAGTTCGAAATTGGAAGTTCTACTTAGAAAAAAATACT

AGAACTTCATAAAATAAAGTTAAAGAAATTTTTGAACTTTCTTTTTTCTCTAATTTTTCT

AATAGAATCTATTCCAATTTCTATATTGAATTTGATTTCAGATATTTTCAAATGGCTCGG

ATGAGTAATCTAATACATAGAAAAGAATAATATATATGAAAGATATAATAAAGAGAAAAT

GCGAATTTCTTGCATTTTAATTCGATCATTATAGATATTTTTTTAGATATAATTTAATGA

TTAATATTTCACTAAGGAGAACATAGAATCATAGCAAATGAAATTGCTAATTCTGATTAG

AAAAAAAGAATGAATATCAAGCGTTATAGTATGATTTTGAATACTCTAAAAAAGAAAGGC

GGGGGAGAGAAAAACTTTGGGATATATTGATTCGGATTGAATTGCAAATACATCAACGAT

AGAATCAATTCAATTCTGAATTGCAATAAGCAGGGTCTGTCAAATAGAGACGAACTGCTA

GACTACGTCGAGTAATTAATTCAACGATTCAAAAAAAACTAAGAGATGGATGAAATTACA

CAAGGAATCCAGGTCTCAAGGAAAATGGGGATATGGCGAAATCGGTAGACGCTACGGACT

TGATTGTATTGAGCCTTGGTATGGAAACCTGCTAAGTGGTAACTTCCAAATTCAGAGAAA

CCCTGGAATTAAAAAAGGGCAATCTGAGCCAAATCCGTGTTTTGAAAAACAAGTGGTTCT

CGAACTAGAATCCAAAGGAAAAGGATAGGTGCAGAGACTCAATGGAAGCTGTTCTAACGA

ATCGAGTTGATAACGATTAATCACGGAACCCATATCATAATATAGGTTCTTTATTCTTTT

TTAGAATGAAATTAGGAATGATTATGAAATAGAAAATTCTGAATTTTTTTAGAATTATTG

TGAACCCATTCCAATCAAATATTGAGTAATCAAATCCTTCAATTCATAGTTTTCGAGATC

TTTTAAAAAGTGGATTAATCGGACGAGGATAAAGAGAGAGTCCCATTCTACATGTCAATA

CTGACAACAATGAAATTTCTAGTAAAAGGAAAATCCGTCGACTTTATAAGTTGTGAGGGT

TCAAGTCCCTCTATCCCCAAACCCTCTTTTATTCACTAACTATAGTATTTATCCTCTTTT

TTTTATCAATGGGTTTAAGATTCAATTGAATAGATTTCTTTTTTATTATAGTATCGGCAA

GGAATCTCGATTATTAACTCTATTTTTAAGTATTATTAAGTAAGCCATGCACAATGCATA

GGATTACCCCCCCTCATTTCCAAATTTCGAATATTGACATAGATACAAATACTCTACTAG

GATGATGCACAAGAAAAGGTCAGGATAGCTCAGTTGGTAGAGCAGAGGACTGAAAATCCT

CGTGTCACCAGTTCAAATCTGGTTCCTGGCACAGAAAAAAAGGATCCACCGAATAGGTAT

TGATACAAATACCTCGAGATGGGTTGGGATACATATTCGTTAATAATATAGATAGAGTAT

GATTTAATCTAAGTAGATAAATCTCTAAATAGAGGCACTTCTTTTTCTTCATTTTGCATT

TCTTAATTTTGTATTCCGCTATTCCGACGAATATTTATTTATTCTTGTTATCTTACTTTC

CTAGCTGTTCTAAGTAATGCGCGCGGTACAAAGTTCGTGGTAGGAACTTCTTTGAGTCAT

CATATTTTTCTGTTCATACGAAGGAAATGAATATGTGATTTTCCAACGAAATGAAGCCCT

TTTTGCTCAGTCTATCTGGACCTTTTGTATAATAGGAATTAATTAGAATATAATAGGTAT

TCCGTTTCATCTAGGAACAGAACGTAAAAATATTCCTTGACTTGAATAAAATCTGGAGTT

GTGTTGTATAAGTGAGCATGAATTTCTTATCATTCAATGAGCATCTTGTATTTCATAGAA

ATTGGGGGTTATATAGTCCTTACGTAAGGGCCAGCCTATCCAACTTTCAGGCATTAGAAT

ACGTTTAAGGCGTGGATGATTATCATAAGAGATTCCCACCATATCATAAGATTCGCGTTC

TTGAAAATCGGCACTTCTCCAAATCCAGAAGACAGACGGGATTCTAGGATTATCCTTTTG

GGCAAAGACTTTTATGCATACTTCTTCTGGGTTATCTATACCATACTGTATTCTCGTAAG

ATGATACACGCTAGCTAAAGATCCACCGGGTGCTACGTCATAAGCACATTGGGAACGTAA

ATAATTGTAACCATATACATATAAAATGACAGCAATGGAATCCCAATCCCCTGCTTTTAT

TTGTAAAGTCTCTATTCCTCGGTGATCGAAGCCCAAAGATCTATGAACCACCTCATGTTT

GACTAGCCAATTAGATAACCAACCCTGCTGCATTGTCTTGATCTCTCCCCCTTTGTATAA

ATATTTCACATTTCGAATGCAAGTTTGAAAGATTGCCCTGCTCTTTCTTTTTCTACACAA

AGAACCCCTCCTAATTCACTAATTTGGAGGAAGATACTGGACTTTTGGATTTGAAAAAAG

TTTCAGAAGATATGTCTAAAGTAGATGGTGATTGATAGAGCAATTCTTGCTCGTAAGTTC

CAGTATAAGTACTGCGCCTAACATAAAGCTTGTGACTGGTAGTAAAACATCGATTTTTCT

TTTGAGATAGAGTTCGATCCTCAACTATTTCTCGCGATATCTTCTTACGAAGTTTTGTTA

GGGCATCTATAACTGCCTCCGGTTTAGGTGGGCAGCCCGGCAAGTAGACATCCACAGGAA

TTAACTTATCAACTCCCCGAACAGTACTATAGGAATCCGTACTGAACATTCCCCCTGTAA

TAGTACAGGCTCCCATAGCAATGACGTATTTTGGTTCAGGCATTTGCTCGTATAATCTCA

CTAAAGAGGGAGCCATTTTCATTGTTACCGTACCAGCTGTTAAAATTAGGTCTGCTTGCC

TAGGACTTGATCTTGGTACCAATCCATAACGATCAAAGTCGAATCGTGAGCCTATTAATG

AAGCAAATTCAATGAAACAACAACTGGTACCATATAGAAGGGGCCATAAACTGGAGAGTC

TTGACCAATTCGAAAGATCATTTGGTGTAGTTGAAATAACGGAATTGGAACTTGTTTGGT

CAAGTAAGGGAAACTCAATCAAACTCATAACTGTCTCAATGGAATCTTTTCCTTCTTTTT

TTTTTTTGTCTGAATATTCAGTTAAGACCATTCCAAGGCTCCTTTTCGCCATGCATAAAC

TGAACCAACAACTGGGATAAGCACGAAAATGAAAGCTTCGATAAAAACGGATACACCCAA

TACGTCGAAACTCATTGCCCAAGGGTAGAGAAAGACCGTTTCCACATCAAAAACAACAAA

AACTAGCGCAAACATGTAATAGCGTATTCGGAATTGTAACCAAGCCCCTCCCATGGGTTC

TATACCCGATTCATAACTAGAAAGCTTCTCTGGTCCTTCACTAATCGGGGCTAAAAGTCC

TGAAATCCAAAATGCAAAAATAGGAATAAGGCTTGCTATTATTAGAAATGTCCAAAAAAT

ATCATATTCGTGAAGCAGAAACATAAATGTACTCCCATTAATGTGGAATAGGCGGAACTG

AATTAGTCAATTCAAGTCAGCGTTGTCAATTTATCCAGAACTTCTCTCTTTTCCTCGGTG

AAACAAGAATCCGTTTTGCTCAAATCAAAGCACTTAGTTTAGCCTTTGTTTCCTCTGTGC

CCTGTCTTCTTTAAAGATTCATCCAATGGAATCCCGACTCCCTTTTTGATTTCCATTCTA

TTTATAATTAGAACCTAATTAAGATAGGATGACTAATGTATGCAGCCTAATGAGGAGTAA

TACAAAAATAAAGAACTCTATTTCAGAAAGTAGATCGATTTAGATAATGTATATAAAAGT

AATATACTTCAAACAAAGTAGGAATTCGCAAGATGGAGAAAATCTTGCAGTTGATTTGAT

AGAAATTCGTTTTTCTTTTCCTGTCTCTAAGATTTTCGATGAATGAGCCTGTGGTAATGC

TTTTATCTCTATTCTATGGCGCAAGCGACCGTCCAGTCTATAAACAAGTACTAATGAGGA

AATGAAAACTATACTAAAGGAAACATAGGATCTCTATCCTAAAATCTAAAAAAGGACATA

TTAGGGCTATACGGATTCGAACCGTAGACCTTCTCGGTAAAACAGATCAAACGGATATTA

TCGAAATGATTCGAACTGTTTCAAAGACCCAACATGCATTTTTTGCATTGGGCTCTTTCA

TCAACTGATGTAAAGATCAGTTAGTCCACCATAGTTTTTCTTTACGGAAAGATAATGAGA

TGGCTCCCTGTGCTCTGATTGATTATTTGTATTATGATCTATCTAGGAGCAATACCAAAG

TGTTTCAAAGGAGGATTACCTTGACTTAGGTCGCCTCCGGCCTAAATTAAATCAACCTAA

GTGAAATGGAGTCTCTATCGTTCCGCTGCAAGAGTTGACTATGAGACTTCATACACCTTA

AAGTTCATAGAACGAAAAGAAGTTTTTTGGAGGCCCTTATCCTCATTACGCCTAGCATTT

AGTGGGCTGGATATTTACCTTATCAACTAGCAAATCAATAAGGGTTCTATTTGATTAGGC

ACCTGAATTGGCACCTGAATCGGACTGAACCGACTGTTTGTCAGGCTACTGTTCTCCTAT

TCTCTCGAATCCATGAAGTAAGACATTGATTTTGCAATAAGATCAATTATGTTCATTGCA

TAATAAGCTCCCTTGAAAAGCATTGGCGCACGTGTAAGCGAGTTGCTCTACCGAACTGAG

CTATAGCCCTTGTCAGAGATATCTTAACATATAGATAATTTCTTGTCAAGATGAATATTC

TCTAATGCCAGAGGATATCCTTTGATCTGTTTACTATATAACATACCAATAACGGAGCAG

TATTGCTTATAAAAAGGATTCGATCTATAATCGATCGAAGTAATGGGTCTTCCTTTGTGG

TGATAAATTGCCTACTTAACTCAGTGGTTAGAGTATTGCTTTCATACGGCGGGAGTCATT

GGTTCAAATCCAATAGTAGGTAGGTAGGTAGAAAAATTACTAGATAGCATTGGACTTACT

TCGCTTCGCTATCTAATAACTTTTTCTACCCCTCTTCCCTTTTTCTTTGTATCAACTAAA

CCATTGGATTGTATTCAATTGGATGGGGGAATCCAATTGATAGCCTCGACTCGTATCCTA

GCTCGTCTGAGAGCTAGCTTCGCTTCAACCAACTCTTTCGTACCCTCAGCTCTACTCAAG

TTAGCTTCGGCTATTTCAAGTGCCTGTTGAGCTTCTTCCGGATCAATGTCACTACCCAGT

TCCGCATCATTTCCTAAAATGATGATCTCATTATTAACTATTCTCGCAAAACCGCTCCAC

AGAACCGCCGTTAACCATTGGTCGTTGAGGAGGCGTATTCTCAAAGGACCCATATCTACA

GCTGTGTTAATGGGGGCGTGGTTTGGTAATACGCCAATTTGGCCACTATTAGTAGATAAA

ATGATTTCTTTCACTTCACAATCCCAAATAATTCGCTTAGGAGTTAGTACATAAAGATTT

AATTTCATTTCTTCAATTTGTTCTCCTCTTCTAAGTTTATAGCTTTCGTGCTAGCTTCAT

CGATGTTACCCACCAAATAAAAAGCTTGTTCGGGTAGGCCGTCTAATTCTCCGGAAAGGA

TTAGTTGAAATCCCCTAATTGTTTCTGCAAGACCAACATACTTTCCTGCAGAACCGGTAA

AAACTTCTGCCACAAAGAACGGTTGTGATAAGAAACGTTCAATTTTTCGTGCTCTTGCTA

CAGTTAAACGATCCTCCTCTGATAATTCATCCAACCCAAGAATTGCGATAATGTCCTGAA

GTTCTTTGTAACGTTGTAAAGTTTCCTTAACTCTTTGCGCAGTTTCATAATGTTCGTTGC

CAACGATCCGAGGCTGTAACATAGTTGAGGTTGAATCTAAAGGATCTACTGCTGGATAAA

TACCCTTGGAAGCTAATCCTCTGGAAAGTACGGTAGTAGCATCCAAATGTGCAAATGTTG

TGGCAGGAGCAGGGTCGGTCAAATCGTCCGCAGGTACATAAACTGCTTGGATCGAAGTTA

TGGATCCCTTTTTTGTAGAAGCAATTCTTTCTTGCAAAGAACCCATTTCTGTACTAAGAG

TAGGTTGATAACCCACTGCGGAGGGCATTCTCCCTAATAAGGCGGATACCTCCGATCCTG

CTTGAACAAAACGAAAGATATTATCGATGAATAGAAGCACGTCTTGCTTATTAACATCTC

GGAAATATTCTGCCATAGTTAGGGCAGTTAAACCAACTCTCATACGAGCTCCCGGCGGTT

CATTCATTTGACCATAGACTAGAGCTACCTTTGATTCCTCCAAATTTTTTTCATTAATTA

CTCCGGATTCCTTCATTTCCATATAAAGATCATTTCCTTCACGAGTCCGTTCCCCTACTC

CGCCAAATACGGATACGCCTCCATGAGCTTTAGCAATGTTGTTGATTAATTCCATGATGA

GTACTGTTTTACCTACTCCAGCCCCCCCAAATAGTCCTATTTTTCCTCCACGTCGATAAG

GAGCTAAAAGATCGACCACCTTAATACCTGTTTCAAAGATGGATAATTTCGTATCTAGCT

CGATAAAGGCAGGCGCAGATCTATGAATAGGGAATGTTGCATTAATATCTACAGGACCCA

AATTGTCAATAGGTTCCCCAAGAACGTTGAAAATTCGTCCGAGAGTAGCTCCACCGACTG

GAACACTGAGAGGAGCTCCCGTGTCAATCACTTCCAATCCTCTCATCAACCCGTCTGTAG

CACTCATAGCTACAGCTCTAACTCGATTATTTCCTAATAATTGTTGTACCTCACAAGTCA

CATTAATTTGCTTACCGGCAGTGTCTCTACTCTTGACTACCAAAGCGTTATAAATATAAG

GTAACTTGCCCGGGGGAAAAGTGATATCCAGCACGGGTCCAATAATTTGATCGATACGCC

CTATGCTTTTTTCTTCAATTGTGGAAACCCCGGGACGAGAAGTAGTAGGATTGGTTCTCA

TAATTATCACATAATTTTCAAAAAAAAGGAATTTGTCGAATTTTTTCTTGTTGAATAATG

CCAAATCAAAAAAAATAGCCAAAAATCCAAAAGTCAAAAGGAAATGAATTAGTTAATTCA

ATAAGAGAGAAAGGGGGACGAGGACTTGATTTCGTTGCCCAAGCGAATCCCATTCAATCG

TTTACTCATGGAATGAGTCCGTTGGAAAGTTCAATCAATTTTTTTCATATACATTTCGCC

TTTTGTGGAGGATCTGTCCCTACTCTACTTTCCTATCTAGGACTTCGATATACAAAATAT

ATACTACTGTGAAGCATAGATTGCTGTCAACAGAGAATTTTCTTAGTATTTAGGTATTTA

CATTCAAAATAAGAAAGGGGCCTATTAAGAACTTAATAAGGATTAGGGATTGGGTTGCGC

TATATCTATCAAAGAGTATACAATAATGATGGATTTGGTGAATCAAATCATGGTTTAATA

ACGAACCATGTTAACTTACCATAACAACAACTCAATTCCTATCGAATTCCTATAGTAGAA

TTCCTATAGGATAGAACATACACAGGGTGTACGCATATATGAATGAAACATATTCATTAA

CTTAAGCATGCCCTCCATTTTCTTTAATGAGTTGATATTAATTGAATACCCTTTTTTTAG

ATTTTTGCAAAGGTTTCTTTCGCCTAATCCTATCGAGTAGACCCTGTCGTTGTGAGAATT

CTTAATTCATGAGTTGTAGGGAGGGACTTATGTCACCACAAACAGAAACTAAAGCAAGTG

TTGGATTTAAAGCTGGTGTTAAGGATTATAAATTGACTTATTACACCCCGGAGTATGAAA

CCAAGGATACTGATATCTTGGCAGCATTCCGAGTAACTCCTCAGCCCGGGGTTCCGCCCG

AAGAAGCAGGGGCTGCAGTAGCTGCCGAATCTTCTACTGGTACATGGACAACTGTTTGGA

CTGATGGACTTACCAGTCTTGATCGTTACAAAGGGCGATGCTATCACATCGAGCCCGTTG

TTGGGGAGGAAAATCAATTTATCGCTTATGTAGCTTATCCATTAGACCTATTTGAAGAGG

GTTCTGTTACTAACATGTTTACTTCCATTGTGGGTAACGTATTTGGTTTCAAAGCCCTAC

GCGCTCTACGTCTGGAGGATCTGCGAATTCCCCCTACTTATTCAAAAACTTTCCAAGGTC

CGCCTCATGGTATCCAAGTTGAAAGGGATAAGTTGAACAAGTACGGCCGTCCTTTTTTGG

GATGTACTATTAAACCAAAATTGGGATTATCCGCAAAAAATTACGGTAGAGCGTGTTATG

AGTGTCTACGCGGTGGACTTGATTTTACCAAAGATGATGAAAACGTAAACTCACAACCAT

TTATGCGCTGGAGGGACCGTTTTGTCTTTTGTGCCGAAGCAATTTATAAATCACAGGCCG

AAACCGGTGAAATCAAGGGGCATTACTTGAATGCGACTGCAGGTACAGTCGAAGAAATGA

TGAAGAGAGCTATATTTGCGAGGGAATTAGGGGTTCCTATTGTAATGCATGACTACTTAA

CTGGGGGATTCACCGCAAATACTACTTTGGCTCATTATTGCCGCGACAATGGCCTACTTC

TTCACATTCACCGGGCAATGCATGCAGTTATTGATAGACAGAAAAATCATGGTATGCATT

TTCGTGTATTAGCTAAAGCATTGCGTATGTCTGGGGGAGATCATGTCCACGCCGGTACAG

TAGTAGGTAAGTTAGAAGGGGAACGCGAAATGACTTTAGGTTTTGTTGATTTATTGCGCG

ATGATTTTATTGAAAAAGATCGTGCTCGCGGTATCTTTTTCACTCAGGACTGGGTATCTA

TGCCAGGTGTTATACCGGTGGCTTCAGGGGGTATTCATGTTTGGCATATGCCAGCTCTGA

CCGAAATCTTTGGAGATGATTCCGTATTACAATTTGGTGGAGGAACTTTAGGACATCCTT

GGGGAAATGCACCTGGTGCAGTAGCTAATCGGGTGGCTTTAGAAGCTTGTGTACAAGCTC

GTAACGAAGGGCGCGATCTTGCTCGTGAAGGTAATGAAATTATCCGAGCAGCTTGCAAAT

GGAGTCCTGAACTAGCCGCAGCTTGTGAAATATGGAAGGCGATCAAATTTGAGTTCGAGC

CGGTAGATACTATAGAAAATAAAAAAGAAGCGAAATAGAAAGAGAAAAAAGCAGTTACGA

AATGCAGTAATTCTTCTTTATTCTTCTAATTGATTGCAATTAAACTCGGCTCAATCTTAA

GATTGAGCCGAATAAAAATAGATCTTGATACGATCATGAGACTTGACAAATCGAGATTCC

TCTATTCTATATATTTAGAATATATAAAGGTATAATACAATAAATAAATACAAATATAGT

ATTTATTTACTTTATTGGGAAAGAATCAATGAAAAAAGATTAGGAATCGATAATATTCTT

CTATAATCCCGGGGTCTTACTAACAGGACTTCGCTGCACGGGAAGGTAAGAGAATTTGCC

CTATTTTGGAAATTACAGCTTAATAAAAAAGTCTAAGCACTATCAATCAACTGTCGTACT

TTGAATCCAATTTCAAGTTCGATTAGAAGGATAGAAAGGCCATGAGGACGGGAAAAGAAA

AATCAAATCTTTTTAATCTCCTTTTTTGCAATTTTCTTATTATCCATTCCATTCATTTTT

TTTATAGAATACTATCTATAAAAAATCTTTTTTTGCAAACTAAAAAATACAATAGTCAAT

ATTCCTTATAATAGATATACTTAATTATATTATAAGAATCCTAAGATATTTTTCGAATAG

ATAGAAATAGTAAATTTGAATTGAGACACCTATTCTATGACGGATTTTAACTTACCTTCT

ATTTTCGTGCCTTTAGTAGGCTTAGTATTTCCGGCAATTGCAATGGCTTCTTTATTTCTT

TATGTGCAGAAAAATAAGATTGTCTAGAACCGATGGGGCCGAATTTTCTCAATGTATTTC

CAGGATCATAATACAGATATTTTTTAGTGTAAGTAATATGGTAGGGTATGTGGCTCTTTC

TACACACAAATGAAAAACGGCTATGGATGCGGATATAGGCTACGAGCATAAATGCATGCA

TATGCGGAGCCGGGTATAGCGAGTTTTTTTAAGTAGATCAACAGATATTTTTTGAATAGA

AAGTCAATGTATCTAACCAATTATTTCACAGGAGTACTAGTTACTGAAGGCGATTTCAGA

ATCAAAAAAAGTAAAGTCAAAATCATTTAGCTTATTCTCTCAATTTCAATCGACCGCTGT

TAGTATATCTAATATGAATTGGCGATCAGAACACATATGGATAGAACTTCTAAAAGGTTC

TCGAAAAAGAGGTAATTTTTTCTGGGCCTGTATTCTTTTTCTAGGTTCACTAGGATTTTT

ATCGGTTGGGGCTTCCAGTTATCTTGGTAAGAATATGATATCTGTACTTCCATCTCAACA

AATTCTTTTTTTTCCACAGGGGGTCGTGATGTCTTTCTACGGAATCGCGGGCCTATTCAT

TAGCTCCTACTTGTGGTGCACTATTTTGTGGAATGTAGGCAGTGGTTATGACCGATTCGA

TAGAAAAGAGGGAATAGTGTGCATTTTTCGTTGGGGATTCCCTGGAATAAAACGTCGCGT

CTTCCTTCGATTCCTTATGCGGGATATCCAATCAATTAGAATTCAGGTTAAAGAGGGTCT

TTATCCTCGTCGTATCCTTTATATGGAAATCCGGGGCCAGGGGGTCATTCCCTTGACTCG

TACTGATGAGAAGTTTTTTACTCCACGAGAAATTGAACAAAAAGCTGCCGAATTGGCCTA

TTTCTTGCGCGTACCAATTGAAGTATTTTGAATACCGATTTAATTTTTTGAAATTGTAAG

GAGATTTTGAGTATTTATCTAAAGAAAGGAACAAACGAGGATAAGAGAAAATTGCTTCTA

ATTTGTCCAAGTGATGGCATAATATTCTTCCATTTTCATCTGAAAGGGCTTTTTCTATTT

CTCTATTCCACTCCATCTAGATCTAAGAAAGAACCCAATGCAATGAAATTCCACTAGTAT

ACAAAAAAGAGGAATAGATACAAGGTCTCAAACCTTGTTATAGAATTTTTGCTTCAAATA

AAAGAAATATCATATATATTCATTTACAGATCAAAATGAAAAAAAAGAAAGCATTGCCTT

CTTTCCTATATCTTGTATTTATCGTACTTTTGCCCTGGGGAGTCTCTTTCTCTTTTAACA

AATGTCTGGAACTTTGGATTAAGAATTGGTGGAATACCAGGCAATCTGAAACTCTCTTAA

CTGATATTCAAGAGAAAAAGGTTCTAGAAAGATTCATAGAATTAGAAGAACTTTTTCTCT

TGGACGAAATGATAAAAGAGAAACCGAAGACACATGTACAAAAACCTCCTATAGGAATAC

ACAAGGAAATAATACAATTGGTCAAAATAGATAATGAGGATCATCTCCATATCATTTTGC

ATTTCTCGACAAATATAATCTGTTTGGCTATTCTAAGTGGTTCTTTTTTTCTGGGTAAAG

AGGAACTTGTCATTTTGAATTCTTGGGTTCAGGAATTCTTCTATAACTTAAATGACTCAA

TAAAAGCTTTTTTGATTCTTTTAGTTACTGATTTTTTTGTTGGATTTCACTCCACCCGCG

GTTGGGAACTACTAATTCGTTGGGTCTACAACGATCTTGGATGGGCTCCTAATGAGCTAA

TTTTCACTATTTTTGTTTGTAGTTTTCCAGTGATTCTAGATACATGTTTTAAATTTTGGA

TCTTTTTTTCTTTAAACCGTCTATCTCCTTCGCTTGTAGTCATTTATCATTCAATTAGTG

AAGCATAAACTCATTCGATTTCCTGATATTAATCAAATTAGCATCTTTCTTCCTTTAGAA

AGAAAGCCCTTTTCCATTTTAGCAAAATTCTTTCTATTTCTACCTGCTCAAGGTATTCAT

CATTCCAGTACAACTGTTGCAGTAGAATGACAACAGACTCGTGTATAGGGAACTAGATTA

GCTTAGCTACCTATCTAATTTATTGTAGAAATTCTGGGATCTGCGATTGGATATGGAAAA

TAGAAATACTTTTTCTTGGGTAAAGGAACAGATGATTCGATCGATTTCTGTATCGATCAT

GATATACGTAATAACTCGGACATCTATTTCAAATGCATATCCCATTTTTGCGCAGCAGGG

TTATGAAAACCCACGAGAAGCAACCGGACGAATTGTATGTGCCAATTGCCATTTAGCTAA

TAAGCCCGTGGATATTGAAGTTCCCCAAACTGTGCTTCCCGATACTGTATTTGAAGCAGT

TCTTCGAATTCCTTATGATATGCAACTGAAACAAGTTCTTGGTAATGGGAAAAAGGGAGG

GTTAAATGTGGGTGCTGTTCTTATTTTGCCCGAGGGATTCGAATTAGCGCCGCCCGACCG

TATTTCTCCTGAGTTGAAAGAAAAGATAGGAAATCTTTCTTTTCAGAGTTATCGTCCCGA

TAAAAAAAATATTCTTGTGATAGGCCCTGTTCCCGGTAAGAAATATAGTGAAATCGTCTT

TCCCATTCTTTCCCCCGATCCTGCTACGAAGAAAGACGTTCATTTCTTAAAATATCCCAT

ATATGTAGGGGGAAACCGAGGAAGGGGACAGATCTATCCTGATGGTAGCAAGAGTAACAA

TACGGTCTATAATGCTACGTCAACAGGTATAGTAAGAAAAATACTACGTAAAGAAAAGGG

GGGATATGAAATATCCATAGTCGATGCATCGGATGGACGCCAAGTGATTGATATTATACC

TTCCGGGCCAGAACTTCTTGTTTCAGAAGGGGAATCGATCAAGCTTGATCAACCATTAAC

AAGCAATCCTAATGTGGGAGGGTTTGGTCAGGGGGATGCAGAAATCGTGCTTCAGGATCC

ATTACGCGTCCAAGGCCTTTTGTTCTTCTTCGCATCTGTTATTTTGGCACAAGTTTTTTT

GGTTCTCAAAAAGAAACAGTTTGAAAAGGTTCAATTGTACGAAATGAATTTCTAGGTCCC

GGCTTACCATCAAGTTGGTAAAAAGCCGCGATGCCTGGAATTCCTTATTTCTATCTCATG

CAAAAGAAGAGAATTCAAGGCAAAGGCGAGAACAATAAAAGGATTTCCTCCTTTTAGGAG

GGATTGCTGGTCTTCTTAATTCTCTATTGGGCACAAGAAAAAGGCTTTTTTGCCTTTTTC

TTGTGTCGATTCTTCTTGTATCAAATCAGAATCATTTTTCTTCCTATTCGTCAAAGATTA

CTATTTCTTCTTTATTCGGGTCTGTCTCTTGGACCTCTTTTGCTTAGGTTCAGGCGAGGT

AGTGGACAAACGGAAAGAAAATATGGCGGGGACAAATTTCTTGTGAGCAAATAGAATTGC

TTGACTTGTTCAATTAAGTTCAACTTCGAACTTACAGAATTTTTGCAAAAAAACGTATAC

TCTTCTATTGAAGGGTGGTTTTTCTTTTTAGGCTAGTTGAGTAGTTTTGATTAAGGTTTT

ATTAGTTTATTACTCTAAACTAAATCAATGATTTGCAGGATACTTCTTCCGTGGAATAAA

ATATTGGATCCTCCCCCTTCTTTCTTGTTGCTTCATAAGAGTGAATCAATTTCATGGGCG

AAGGGATAAATCAACCGATGGATTGCTTCACTAACACCATTAACAAACAAAAGAATAAAT

AGAGGGATTCTGACCATCAGAGCAAAGGTTTCTCTTTGTTATTTTTACAAATCGAAATAG

GTAACCCATTTGTAGGTTATGGAATAAAGTTATATTATAAGAGTAAGAATTCCGCGGGTC

CTTTCCGCTCTAATCAGATAAAAGGGGGTAAGGACCCGCTAAGCTCCTACTTTTTCATGT

TTACAATCTGGTCCCTCCGATTACTATAGAGATGAACCCAATCCAGAATATGAACCGTAA

AAGAAAACACCTATTAAACCAATCACAGGAATACCAGTTACAGTACCTATCAGCCAAAGA

GGAATTCTTCCAGTAGTATCGGCCATTTCCCCTACTTTCCTCCACATTTTATCAAGTGGT

CATGCTAGAGACAAAAACAGTCATGGATAGTTATAAGGATGGTATCCTTCCAAATGGGAT

AAGAGAATTCTTACTACTCTCTTTCTTTCTCTCAATTGAAGAAGTAATTGGAAAATAAAA

CAGCAAGTACAAAAATGAGTAATAAACCCCAGTATAGACTGGTACGATTCAATTCAACAT

TTTGTTCATTCGGGTTTGATTGTGTCATAGTTCTATAGTTGGAATTTGGTTTATCGTTGG

ATGAACTGCATTGCTGATATTGATCCCAAGAAAAAAACAGTAGGTACGGCTAGTCCGTGA

ACAGCCAGCCATCGGACTGTAAAAATAGGATAGGTTCGATCTATGGTCATTGAGGGCCTC

CTAAAAGGATCTACTAAATTCATCGAGTTGTTCTAAAGAATCAAAACGGTCGGTTATTAA

GGGAATTCCTTGTCGGCTTTCCGTGAAATACTCGTTTGGCCGAGGACTTCCAAACACGTC

ATAAGCTAAACCCGTACTGACAAATAACCAACCCGCAATGAATAGGGAAGGTATAGTAAT

GCTATGAATAACCCAGTATCGAATACTGGTAATAATATCAGCAAAAGAACGTTCTCCCGT

GCTTCCAGACATGCTGAGCTCCCCAAATTTTTATTCAAAAAAGGAATTGATTCCGTAAAA

GATGGGATCAACCAGTAAATAGAAAATTACTGATATTTCATCCTTGTGAGATTGTCAATT

TTGTACCAAAGGTGTATTTTGAGTATACCGAATTAGTATAGCTATCCTTCCTATGGCACA

GCAATCTAGTTTTGCTTGGTCCCGAAACATAATTCTTTTTTTCTCTTCTTTGTTCCTTGT

CTATAGGTAAGCTATATGTTATTCAAGGCATCAATAGAAAACCCCATTTTTGGGGTCCTG

CTTATTTTCATTGGCTTCGGATTAGTAGAATAATTCGGAATAGCGGCCAAGATCTTGGGA

AAATCCAAGTTAATGATCAATAGGATAAATAATTTAGGAAAGATATTCTCATACTGACAC

AATATAAGGACAAGTATATGCGAAATCTATCCCTTAAGGAATTTAATTGGTTAGCATAAT

ATCTAATAAATAGAAAATCGAATAGTGGATAATCTGTTATGAGAGAAAGAAAACATTCTT

TGAAGAATCAAGATTCGTAATCAATCCTTGCCTTGTTTACTAACTTTCTTGACTAAACTG

CAAGCGTGGAACTCAGGAAACTTCGAATCAACTTTGGTTGGGGTTCAAAAAGGAATAAAA

ATAAAGTAAATTCAAGGAAGATTTCCTTTTTTTAAGGGGCCCTCGGGGGTCGTGGAATGC

TTTTCTTCTCCTCTTATTCCATATGGAATACAATCAGTTAAAATAAGAAGGAATAGGGAA

TATTCGACTGTTTCAATTCTTTATTTATCTTATATTCCAAAATTCTCCCAAAATCCAATT

TAATTTTTCAATGGGGTTAGATGATCTAGTTCTTAATATTATTACTTTAAAGAACTGACA

GATTCCACAACAAATCTCTTGATTCGGAATTAGAGACTCATGTCCCATCTGATGAATCGA

TTTTCTTTTACACTTCTGTATCTCACTCTATCTTGTTTTTTAGTATTATCTAAAATAACC

GATGAATTATGAATTTTCCATAACTTAGGTAAGTGCTTTACCAACATATGTAGTGTAGTA

AAAAAAATGGAATTTAACCCTTTCATGCTTACTATAACTAGTTATTTCGGTTTTCTACTG

GCTGCTTTAACTATAACCCCAGCTCTATTTATTGGCTTGAACAAGATACGTCTTATTTGA

AATGAATTGAAAATCTTTCTTTTGGATTCCTGGTATTCTAGCACTAATTATCAATTCTTT

TCTTGGTCATTGAGATTCGTGGATAATTTAGACTACTATTTAGGGATAGATCGTACCTCT

TTTTTTATCCCCTCGAACAAATCGAAATGATTGAAGTTTTTCTATTTGGAATCGTCTTAG

GCCTAATTCCTATTACTTTAGCGGGATTATTCGTGACTGCGTATTTGCAATACAGACGTG

GGGATCAGTTGGATCTTTGATTGAGTAATATTTCTTTTTTGATTGACCTCCTCCAGACCA

GAGAGGAGGTCAAATTGGAGTTGCAATTTGTTTTGTTAAGTTATTTTAGCTTCGACATAA

GATAGATGGAATCACGCTCTGTAGGATTTGAACCTACGACATCGGGTTTTGGAGACCCGC

GTTCTACCGAACTGAACTAAGAGCGCTTTCAAAAAGAAAACCCTTTTCTACTCCTAACGT

GTCTCACGTACGTATAGTATCCACAAATTCAAGTTATACCCACTTTAATTGATCTCCTCG

CTACTGCCCATAAAGAAGAAAGAAGTAATAGGTAGGGATGACAGGATTTGAACCTGTGAC

ATTTTGTACCCAAAACAAACGCGCTACCAAGCTGCGCTACATCCCTTTTCCAAATTGTTG

TATAATGGCATTGTACACAATTCCTGTCTTGTTTTCCACATCGTAATTTTCTTCTCTTTC

TCTATCTATATAGAACCTTCTTGTCATTTCTTCTTTTTGGTCTCATATAATCAAGGAATG

GTATACATCTAAATCCTATCTAATTTCACCTATAAAAGAAAGATTACTATTCCTTGGTAA

TGTATAGGAAGGGGTCTTTTTAGTTAGGAATTTCGCCTAAACAAAAGAAATACAAATGAT

CTTGGGCAAGAGTATCTGATCATATATGTATTCCAATAAGGAAGGAGGATTTTCAATGCG

GGATATAAAAACATATCTCTCTGTAGCGCCCGTGCTAAGTACTCTATGGTTTGGGGCTTT

AGCAGGTTTATTGATAGAAATTAATCGTTTATTCCCAGATGCTTTGTCATTCCCTTTTTT

TTAATTCTAGTTATTGCTATGCGAGGAATTCTTCGTGACATGACGCAAATTTTCCCTTTT

TCAATCTTTTTTATAGGAAGGAAAAAGAAAGAAAAGATGGATAAGGATTGTATTCTTTAA

TTATTTCTCTATTTTTTATTACTTAATTTAAGAATTTTAAAAATTTTTTATTCGAATGGA

TTTTATTCTTCTTCTTGGGATTCAAAATAGAAGAATAACAGAATAAGTAGAAGAATTAAG

TTAAGTCAATCCAAAAAAGAAAGGAGGTTCATGGCCAAGGGGAAAGGTGTTAGAATCAGA

GTTATTTTGGAATGTATCAGTTGTGTTCGAAAAGGTGCCAATGAGGAATCGACGGGGATT

TCTAGATATAGTACTCAAAAGAATCGCCACAATACACCCGGACAATTAGAATTAAAAAAA

TTTTGTCGTTATTGTCGCAAGCATACGACTCATGACGAAATAAAAAAATAGGAGCATCGT

GTGTTCGATCTTTCCAAAGAACAGATTTAATATATAGAACATAGATAGAATACAAAATAC

AAATCAATTCATTTGATTTCAGGTAGATATTATTTCATATGTATAGAGGGTATTCATATA

CTATATGGACCAAAGAAAGACTACTTCTTCTGGATCCAAAATTAATAAAATAAAGAAATC

CATTTTTTATTTTAAAATAAAGAATAAATCATGTATACATCTAAACAACCTTTTCATAAA

TCTAAGCAAACTTTTCATAAATCCAAGCAAACTTTTCATAAATCCAAGCAAACTTTTCGT

AAATCCAAACAACCTTTTCGTAGGCGTCCTCGGATTGGCCCGGGGGATCGAATTGATTAT

AGAAACATGAGTTTAATTAATCGATTTATTAGTGAACAAGGAAAAATATTATCGAGACGA

ATAAATAGATTAACCTTGAAACAACAACGATTAATTACTCTTGCTATAAAACAGGCTCGT

ATTTTATCTTTCTTACCATTTCGTAACTATGAGAATGAGAAGCAATTTCAAGCCCAGTCA

ATTTCAATAATTACTGGTCCTAGACCCAGAAAGAATAGACATATTCCTCAATTAACGCAA

AAGTTCAATTCCAATCGAAACTTAAGAAACTCCAACCAGAATTTAAGAAACAACAATCGG

AACTTAAGTTCCGATTGTTGATGTTTTATTCGAAAGGGCCAGACCTATATAAAGAAAGTA

ATCCAGTTTTGATTCTTGTGTTTGTTATAAGAAAGAAAAATGGGGAAGAATAAATAGTTT

TTTTATTTATTGCAACATGCTCGTTGATTCCTACCACTTAATCTTAATTTATTGTATCTT

CCCGGAGTTCCCTCTCCGGGAATTCGGTTTTAATTATTCCTGTATATTACTTTTTTATCC

TTTAATTGAGGATCTTTATTTTATTGGAAATCGTGTAAAGATTATTTGGATTTGATACAG

CTACTTGTGCAAGCATTTTACGATTAAGAATCAATTCCTTCTTGTACAGATTGTGTATTA

ATTTACTATAACTATCGAATACTTTATATATCCGCGTTGCTGCGTTTATCCGAGTGATCC

ACAAACGACGAAAATCCCTCTTTTGCCTGCCTCTATCTCGATGAGAGGAAACAAAAGCTC

TTCTTACTTGTTGAGTAATCATTCGATTAAGTCTTAAATGAGCCCCTCTAAAGTTTGAGG

CAAATGAACGCATTTTTGTTCGTCGTCTCCGAGCTATATATCCTCGCGGAACTCTGGTCA

TTGAATCAAATTAACCTTAATGAATAACTAATGATTTCTCTTCTTTTAGCCATCCTTTTT

CCCATTAATAACAAAACGAATTATTCCGATATATAAAATATTAATTCCAATGGCTTTTGC

TACTGTAACCTTCCCAACCACGATTTTTTATTCTATTCAGTTATTTCGCACGATACTAAA

AAATAGTGGGTTCCATCGTTTCTATGGTTCCCTTTTAAACGGTGAGGCCCTCTCTATACA

CCGGAGCCCTTTCTTTCATCAAAAGGTATTGTGAACTTGTATAGTTCACATTCTTTGGCT

CTACCTATCCATTATAGAGTAAATAGCTCTTTTCACAATAAGAGTTATCCATACAGTGAC

GGCATTTAATTATGAAAGTTGCTAAGTAGCTGACCCTGTTAGTCCGTTCTTTTAAGATAA

AGGAGCATAAGCCTTTTTCTTTTTATTACTATTTCCTCCGCTTAATGGATACCATTTTCT

ACCAATGGGGAATTGCTTCTTCCAATTTAGATGATTGGATTTGCACCAAAGGAAACCAGA

AATTCCATATACCATAGAATAGGATAGAGAAGCTCTATCCTATTCATTGGTACCGATCAT

GGATACTTCAAAAATTGTCTTATTTGTTTGAACTCATGATCTGAACGAGTCGCACATACA

CCCTAGCACATGTTCCTCGACGCTGAGGACATCCCTTAAGCGCGGGCGATTTTCTAGCAT

TTCGTATTGGCTGTCTTGCGTTTCTAATAAGTTGTTTAACCGTTGGCATGTCGTATGTAT

ATAGAAAAAGGATTGGTTTAGATCGATCTTAACCTGATGATTGATCATCATGAAGTATTT

CTATCGCAGAAAACCTGAATTTAGGTTTGAAATAAATTTACAAGAAATCCGGCCACTACC

AATCCTTAAACATTTCTGGAAACCACACTGGATCAGTATCGCAGTGCTCGTCAATCATTT

CATCCCCTACAATATCGACAAGTCCATAAGCTTTGGCTTCGTCTGCTGACATAAAAACAT

CCCTTTCCATGTCTTCGGATACAACCCAAAAAGGCTTGCCTGTTCTTAGGGCATAAACCC

TTGTGATCATTTCGCGAACTTTGTGTAACTCTTCCACTTCTAGTAAAAATTCTGGTGTCC

TTGCCCGATAATAAGCACTAGCAGGTTGGTGAAGCATAATCCTCGCGTGAGGGAATGCTA

TACGCTTGGTGGGTTCGCCTCCAAGCAGAATGAAGGATGCCATGGACGCAGCCATTCCGA

GGCATATTGTATATATATCTGGTGTCACCGTTTGCATCGTATCAAAAATCGCCATTCCTG

AGATTAGCCACCCGCCTGGGGAGTTTATAAACAAAAAAATATCGCTAATTCCATCTTCTA

TACTGAGATATACCATGAGACCTGTAATATGATTCGTGACCTCGCAACGAATCTCTTGAC

CTAAAAAAAGTGTCCTTTCTCGATACATAACATTGTATAAGTCAACCCAAGTCGCTTCTT

CATCTCCGGGAATCCGGTAAGGTACTTTTGGAACACCAATGGGCATATTAGATTAATATT

ATTAAATTTAAGTAAGAAAACTACACTTTAATATGGAAACGTAAGAATGGAAGAGAAAGA

AAGAATCCGCAGTTTATTGTCTTTTTTTTTTCTTATTCTATATGAATACTATAGATTCTA

TTAATACGTAGATTGAAATATACTATAAGGAAGGTAAGACAGAAAGAAAAAGGAATCGGT

GATTGGAATGATAAACAAAGAGGGATAGGGATCTATTCTTCGTTTTTCCAAATAGGCCAA

GCTACCCATTGCATATTGGCACTTATCGAGTATAGAATAGATCTGCTTCTCTTTCTTCTT

ACGAACAGAATTGGCTTCTTATTTTTAATGGAATGAAATAAATATTCACGCTTTCTGACA

CAGAATCCCCTAGAGGGGTTAGGTACATAGGATATAGATAGTCTTTTCCAATGCGATAAA

ATAAAGCGACATCGTGTCTATTTTTCTTTGCTAAAGGGGTATTTCCATGGGTTTGCCTTG

GTATCGTGTTCATACTGTTGTATTGAATGATCCGGGTCGATTGCTTTCGGTGCATATAAT

GCACACAGCTTTAGTTTCTGGTTGGGCCGGCTCGATGGCTTTATATGAATTAGCGGTTTT

TGATCCCTCTGATCCTGTTCTGGATCCAATGTGGAGACAAGGTATGTTCGTAATTCCCTT

CATGACTCGTTTAGGAATAACCAATTCGTGGGGTGGTTGGAGTATTTCAGGAGGAACTGT

AACGAATCCGGGTATTTGGAGTTATGAAGGTGTGGCAGGTGCGCATATTGTGTTTTCTGG

CTTGTGTTTCTTGGCAGCTATCTGGCATTGGGTATATTGGGACCTAGAAATATTCTGTGA

TGAGCGGACAGGAAAACCCTCTTTGGATTTGCCCAAGATCTTTGGAATTCATTTATTTCT

TGCAGGGGTGACTTGCTTTGGCTTTGGCGCATTTCATGTAACGGGTTTGTATGGTCCTGG

GATATGGGTGTCCGATCCTTATGGACTAACTGGAAAAGTACAAGCTGTAAATCCAGCGTG

GGGTGTAGAAGGTTTTGATCCTTTTGTTCCGGGGGGAATAGCTTCTCATCATATTGCTGC

GGGTACATTGGGCATATTAGCGGGCCTATTCCATCTTAGTGTCCGTCCGCCTCAACGTCT

ATACAAAGGATTACGTATGGGCAATATTGAAACTGTACTTTCCAGTAGTATCGCTGCTGT

TTTTTTTGCAGCTTTCGTAGTTGCCGGAACTATGTGGTATGGGTCAGCAACTACCCCAAT

CGAATTATTTGGGCCTACTCGTTATCAGTGGGATCAGGGATACTTTCAGCAAGAAATATA

TCGAAGAGTTAGCGATGGGTTAGCCGAAAATCTTAGTTTATCAGAAGCTTGGTCTAAAAT

TCCCGAAAAATTAGCCTTTTATGATTATATTGGTAATAATCCGGCAAAGGGGGGATTATT

CAGAGCAGGCTCAATGGACAACGGGGATGGAATAGCTGTTGGATGGTTAGGACATCCCGT

CTTTAGAGATAAAGAAGGACGCGAGCTTTTTGTACGCCGTATGCCTACTTTTTTTGAAAC

ATTTCCGGTTGTTTTGGTAGATGAAGAGGGAATTGTGAGAGCGGACGTTCCTTTTAGAAG

AGCAGAATCCAAATATAGTGTTGAACAAGTAGGTGTAACGGTGGAGTTCTATGGTGGCGA

ACTTAATGGAGTAAGTTATTCTGATCCTGCTACTGTAAAAAAATATGCGAGGCGTTCCCA

ATTAGGGGAAATTTTTGAATTAGATCGGGCTACTTTGAAATCGGATGGTGTTTTTCGCAG

CAGTCCAAGGGGTTGGTTCACTTTTGGTCATGCTACCTTTGCTTTGCTCTTCTTTTTCGG

ACACATTTGGCATGGCGCTAGAACCTTGTTCCGAGATGTTTTTGCTGGTATTGATCCAGA

CTTGGATGCTCAAGTGGAATTTGGAACATTCCAAAAAGTTGGAGATCCAACTACAAGGAG

ACAAGCAGTCTGATACCACATTGCTATGGTATCTTTCATCTCTCTTTTTTGATTTGACAT

GGGAAACATCTCCCATCCTTTCTTTGACTCTTTTTCTTTCTTTATATGGGAAAAGATCCC

AAATGACAAATGAATAGGTGTGGAAGTTATAATTGTAAATAAACCACGATCGAATCTATG

GAAGCATTGGTTTATACGTTCCTTTTAGTTTCGACTTTAGGGATAATTTTTTTCGCTATC

TTCTTCCGAGAACCACCTAAGGTTCCGACTAAAAAATGAAATAATTTCATTGAAGTAAGA

AGTCTCCCAGAGGGGAGACTTCTTACTTCAATTAGTCCCCGTGTTCTTCGAATGGATCTC

TTAATTGTTGAGAGGGTTGCCCAAACGCGGTATATAAGGCATACCCAGTAAAGCTTACAA

GTAAACCAGATATGGAGATGGCGACTAAAGTTGCTGTTTCCATTTTTATAGAATTTCAAG

ATTACAATGGATCTACGAAAAGATCTTGTATTTACAACTACAACGGAATAGTATACAAAG

TCAACACCAATGATTAAATAGAATTTATGGCTACACAAACCGTTGAAGATAGTTCTAGAC

CTGGGCCAAGACGAACTCGCGTAGGTAGTTTATTGAAACCCTTGAATTCGGAATATGGGA

AAGTAGCTCCGGGTTGGGGGACTACTCCTTTTATGGGGGTCGCAATGGCTTTATTCGCGA

TATTCCTATCTATCATTTTAGAAATTTATAATTCTTCTGTTTTACTGGACGGAATTTTAA

TGAATTAGGTTTCTACTAACTAAAACTACGAAGTCATTGTTTTTCCATCCAAAAAAGCCT

TTCTACTTTAAGCTATACATTTCTAGACATTCTGGTAGTTCGACCGTGGATTTTTTTGTT

TTGGTATCTCTGGAATATGAGTGTGTGACTTGTTAGAATGGATCCTATTGATAATACATA

GAAAGGGCCTGTTATCTCTATCAAGATGATTCTAATTCGTCGGATATTTTTTATTCTAGT

ATCTGGAACACGAAATAGATAGAGTGGATCAAAAAAAATGGAACTATGATTCATACTCAC

TATTCAGACCTCGCAACCAGACTGAAAAAAATTCAAGTAGTTTTTAATAAAAAAAGAAAT

TTTCTTCCTTCCAATTTTGTTTGCCCAAAAGACAACTTTTTTCTCTTTCAATAAATGATC

ATCAAGCGGTTCTTATTCGAAGAACCCTTGCCTTTTGGTTAGCTTGAGACTCAATCATCG

TGGCTCTAGTATGAATCTAAGGTTTTAATTGAACTGATTCATAGGATCGCAACAAGATAA

TTTCTATCAGAAAACTACTAGAATTTTTGCTTTATTTATTTACTAGTCAAAAAAAAAAGA

AATCCAAAATAGGGAAGAGAAAAGTCAAGAAGCCTCTAATGACCAACATAAGGGAAAGAA

AGACAGATGAGCCAACTTGAGATTTTTTGGCATTATCATCACAAAGAATAAGTTCTGGAT

TTTTCTTATTTCATATCTTCAAGGCAAATCGACCCAATCCAGTGGCTGATGAAGTTTTGA

ACCTTTTTTCTAATATCCGTTGAAAATTTGTGTGTTTCTGCTTGAGCCGTACGAGATGAA

ATTTTCATATACGGTTCTCGGAGGGGGACTCGGGTTAGTTACCTATCTCAATAAAGTATA

TGATTGGTTTGAGGAACGTCTTGAGATTCAGGCAATTGCGGATGATATAACTAGTAAATA

TGTTCCTCCTCATGTCAACATATTTTATTGTTTAGGGGGAATTACACTTACTTGTTTTCT

AGTACAAGTCGCTACCGGTTTTGCTATGACTTTTTACTACCGCCCAACCGTTACAGAGGC

TTTTTCCTCGGTTCAATACATAATGACCGAGGCCAACTTTGGTTGGTTAATCCGATCAGT

TCATCGATGGTCAGCAAGTATGATGGTTCTAATGATGATCCTGCACGTATTTCGTGTGTA

TCTCACAGGTGGATTTAAAAAACCCCGCGAATTAACTTGGGTGACAGGTGTGGTTTTGGG

TGTATTGACTGCATCGTTTGGTGTAACTGGTTATTCTTTGCCTTGGGATCAAATTGGTTA

TTGGGCAGTCAAAATTGTGACAGGTGTACCTGAAGCGATTCCGGTAATAGGATCGCCTTT

AGTGGAGTTATTACGTGGAAGTGCTAGTGTGGGCCAATCCACTTTGACTCGTTTTTATAG

TTTACATACCTTTGTACTGCCTCTGCTTACTGCCGTATTTATGTTAATGCACTTTCCAAT

GATACGTAAGCAAGGTATTTCGGGTCCTTTATAGGGAAGCCATATCATAGAGAATTCTAA

TTTTCATATATCATATCGGGTAGGTTGTGGTATTTCATTGCTACAAACATGGGTTATTGT

AAAATAAGACATGTCATTTGGATACTTTTCTTCAACTCCGAAGTATTTTGATACAAATAG

TTGAAGTTCATTTTATGAAAGAAAATAAGGCGGATTATGGGAGTGTGTGACTTGAATTAT

TGATTTGGCCATGCAGATAAAGAATTGGATCTGCCACATTAGAATTCACAACAAAGTGTC

TCCGCATCCAATCAACACGTAAGTCCCCTATCTAGGAAGGATAGGCTGGTTCACTTGAGG

AGAATATTTCTATGATCATACCCCAACCATGTCATCCATGAACAGGCTCCGTAAGATCCC

ATAGAGTAGAAATAGAATAAGTCATGTGACATGATCCAATTCTCTATTTATTACACTTAC

TTTTTATTATAGTATGGAAATGCATTCATTTTCTTTGCATCGATTGCGATCCGCAATACT

ATCGGAGTAAAAGAAGGTATCTAAAGAAGAACGTAGGCTAGACTTTTTGATTTTTTATTA

GTAACAAGTAAATACTTTGTTTGGACGTAAGAAACTTGCGATATTGAGGGGATAAACACC

AACTAATCAAGAGACAATCCACAAAGCAATTGATCATGATCAAATTTGCAAGCCAACTTG

GATATTGAGCATTTACCCATAAGAATAAGATTCTTTTCAATAAGTAGTTGTAGGTGCAAC

TTCGGAAAAGAGAATCTGATAAAGCTTTTCTTACCTAGAGTCATTGAGTCATTATATACC

TTATTCTATTATGGATCTTCCACGGTCTTTTTTCTTTCATTCTTGCTCGAGCCGGATGAT

GAAAAATTCTCATGTCCGGTTCCTTTGGGGGATGGATCCTAAAGAATTCACCTATCCCAA

TAACAAAGAAACCTGACTTAAATGATCCTGTATTAAGAGCAAAATTAGCTAAAGGGATGG

GACATAATTATTACGGGGAACCCGCCTGGCCCAACGATCTTTTATATATTTTTCCAGTAG

TAATTCTAGGTACTATTGCATGTAATGTAGGTTTAGCGGTTCTCGAGCCGTCAATGATTG

GTGAACCGGCGGATCCGTTTGCAACTCCTCTGGAAATATTACCCGAGTGGTACTTCTTTC

CCGTCTTTCAAATACTCCGTACAGTACCCAATAAGTTATTGGGCGTTCTCTTAATGGTTT

CTGTGCCAACGGGCTTATTGACAGTACCCTTTCTAGAGAATGTCAATAAATTCCAAAATC

CATTTCGTCGCCCAGTAGCTACGACCGTTTTTTTAATCGGTACTGCAGTAGCTCTTTGGT

TAGGTATTGGAGCAACATTACCCATTGAAAAATCCTTAACTTTAGGTCTTTTTTAGGGAT

TTTTCAGGTTGATTCATTCAACCGTGAAGTACCGTGCATAGGTATCTAGGAAATAGTTAC

TTCCAAGTGAATCTTCCCTAGATACCTAAAATCCATTTTATTATGATCCATTTCGCGAAA

ATATAGATTGTGCCAAAGATGCAAAATTGTTTTTTTTTATTCTAACTCGAAAAAGAAGAA

GAGGAAAAAATTGCAATGATTTAAAACGAGAACTTATTCTTAGGTAAATCGATTGGGAGA

TGCTTCTCTAGAGTGTCCCATATCTGTTTTCCATCTTCCATACGAAAACTGTCAATTCTC

ATAAGATCTTCTTCAGTCTTACTCAAAAGGTCCAATAGTGTATGTATATTGGCCCTTTTG

AGACAATTATACGTTCTAGAAGGCAATTCTAATTGATCAATAAAAATACAATTCAATGGA

ATTCCTTTTTTGTTTTTCTTTAGATTAGTTAATCTTTTTTGAAAGGTTAAAAGGGGTGGA

GTAAACCTGTTTTTATTTTCTTCGAAACTAGTGCCCTCTTCCTCCGCGTGTAGAAAAGGA

AGAAATAAATCAATCAAATTACGAGAAGCCTCATAAAGCGCTTCCTTAGGGGTTAAACTT

CCATTCGTCCATATTTCTAGAAAAAGTATCTCGTGTTTTTCATTTCCATTCCCACAAGAA

AAAATACTATAATTCACATTTCGAACAGGCATGGATACAGCATCTATGGGATAACTTCCA

TCTTGAGAGTTCTTTCTGAGTTCCGTGTGATATCCGCGATCTCTCTTGATCTGTAACTCA

ATACAGAAATCAATGGGCTCTGTCAAGTTAGCTATAGGTTGTGCCGTATCAACGATCTCT

ACGGAAGGTGGTAAGATGATATCTTGAGCAGTTATGTATCTAGGACCTTTGACGCAAATT

GATGCGTCTCTAACTCCATAGAGATTACTTCTCAATACAATTTCTTTCAAATTTAGTAAA

ATTTCTTGTACGGATTCTTCAATACCAGCTATTGTAGAATATTCGTGTGGCACGCTCCCA

AATTTTGCACGTGTGATACATGTTCCTTCTATTTCTCCAAGTAAAGCTCTTCGCAAGGCA

ATACCGACGGTATCCGCTTGACCTTTTCTAAGCGGGGACAAAATGAAACGACCATAATAA

AGACGCTTACTATCTACTCTTGATTCAACACACTTCCACTGTAGTGTTTGAGTGGATCCT

GCTACCTCCTCTCGAACCATATAGACTATATTATTTGATCATTGAATCGTTTATTTCTCT

TGAAAGCGTTTTAATTCTTTTACAGACGTCTTTTTTTAGGAGGTCGACATCCATTATGCG

GCATAGGTGTTACATCGCGTATACAACTTAATCGTACACCACTTTTAGCAATGGCTCGTA

ATGCGGCATCTCTTCCACTACCAGCACCCTTTACCATAACTTCTGCTCGTTGCAAACCCA

CTGTACGAATAGCATCTACTGCTGTTCTTTGACCAGCATAGGGTGATGCTTTTCTTGAGC

TTTTGAATCCACAAGTACCCGCGGAGGACCAGAAAACCACCCGACCTTGCGGGTCTGTAA

CAGTTATAATGGTATTGTTGAAACTAGCTTGAACATGAATAACTCCTTTTGTTATTCTAC

GTGCACTTTTCCGTAAACTAAAACGCGCATTCCTACGCAAACCAATACGCACTCTCCTAC

GTGAACCAATTTTTGGTATAGCTTTTGTCATATTTTATTATCTCATAAATATGAGTTAGA

AATAACAAAAAGAAAAAAGATACAAAGATATCCGTTTCAGGGTAAAATATATCCTTTACT

TTAATTATTTTATTTGGAATTTGGACGTTTCCGCGGGTACTTTTTACTTTTTAGAAAGTA

AAGTTCTTTTTCGAAAGATTACCCCTGCCTTTGTTTATGCTTCGGATTGGAACAAATGAC

TCTAATTCGTCCACGCCTACGAATCAGTCGACATTTTGTACAAATTTTACGAACGGAAGC

TCTTATTTTCATATTTCCTTATTCCTTTCTTAAACTATGAATCTAATCTTTGGAAAAAAT

AAGTCTCTTCGCTTGAATTTTGGAACTTTGAATTTATTACCCTAGAAAAACCTAATCCTT

TGAATCTTTGGTATCCTTCAAATCTTCGGTATCCTTCGAGTCTTCGGTACGCTTCGAATC

CTTATGGGGAAGTCTATAAATTATACGTCCTTTGCTTGAATCATAACGACTTACTTCAAT

TTTGACCCTATCCCCCATCAGTATTCGTATAGAACTAGACCGGATCTTTCCTGAAATATA

GCCCAGGATGATGGTGTCATTCTCTAGGCGAACGCGGAACATTCCGTTGGGTAGGGCTTC

CGTAACTAAACCTTCGAAAGTGACTTTTGCTTCTCTCGGGTTTTTTTTTTCTGTCATATT

TTTTTCTCCTATTTTTCTATTTTGATTTTCAAAATAGGAAGTTCGAGATAGAATTCGAGT

ACTATAGGAGGGGCGATTACCATATATAACATAAGACTTCTCCCCCAATTCTGTTTAGTC

GAGCTTCTCGATCTGTCATTATACCTCGAGAAGTAGAAAGAATAGCAATTCCCATTCCGC

CCAAAACTTTAGGAATTCCTTGATAGTTGGCATAAATTCGTAAGCCGGGTCGGCTGATAC

GCTTTAAAAAGGTTCTAGTTCTATATATTCCTTTTCTAGTCTTTCTCTTTTGATGTCGCA

AAGTTGAAACCAAGAAATATCTGTTACTTTCCTGATGTTTCCGAACACTTTCAATAAAAC

CCTCTCGTAGAAGTATTTTAACAATGTTTTCGGTAATATTTGTAGATACTACTCGAACTG

TTCCTTTTTTATTCATGTCCGCGTTTCTTATAGAGGTTAGTAAATCAGCAATAGTGTCCT

TGCCCATAAGACTCTAATTCTAGGTTCCTCCTAATTTTTCTATAATCAACATGTTTTCTT

TTTCTTTTACTTTTGGATTTTAAAGCATATACGTGAGACATAATCTACTAATTTTTTCTT

TGATCTATATCTCGCCTACTAGTATTTATAATACTTCAGGAGCTAATGAAACTATTTTAG

TAAAATTCAATTCTCTCAATTCCTCGGCGATCGCGCCAAAAACTCGAGTTCCTTTTGGAT

TTCCTTTTTGATCAATGATAACCGCTGCATTGTCATCATAGCGTATTATTATACCGTCTT

CGCATTTGAACTCTTTACATGTACGTACAATTACAGCTCGAATTACTTCGGATCTTTCTA

GAGGCATTTGGGGCACTGCGTCTTTGATTACAGCAACAATAACATCACCAATACGAGCAT

ATCGCTGATTACTAGCAGCTCCTATGACTCGAATACACATCAATTTTCGAGCTCCACTGT

TATCTGCTACATTTAAAAGGGTCTGAGGTTGAATCATATTATTTTGATTTCAATTTGTTA

TTTCTATGCAAAGGATGAAAGAAATATTGTCTTTCCAGAAAAAAACCTGGTTTTTTACTT

CAATACTCCTTTTTGGGATGCTATATCTCTAATCGAAGAAATTGACTTCGTATGGGCATT

TTACTGGCAGCTATGGAGATAGCTGCTCTAGCTACAGTTTCAGATACTCCGCCCATTTCA

TAAAGTATTCGACCTGGTTTAACAACGGCTACCCAATATTCGGGGGATCCCTTTCCTGAG

CCCATACGTGTTTCCGTGGGTCTTAGTGTAACCGGTTTGTCGGGAAATATACGTACCCAT

ATTTTTCCACCACGACGTGCATATCGTGTCATTGCTCTTCGTCCTGCTTCTATCTGTCTC

GACGTGATCCAAGCGGGTTCAAGTGCTTGCAGAGCATATCTACCAAAACAAATACGATTG

CCTCGGCAGGATTTTCCCTTCATTCTTCCTCTATGTTGTTTACGAAATCTGGTTCTTTTG

GGGTTATAGTCGATGGTTCTTTCTTAGTTCCATCTCTACTGCAAAACTGGACATGAGAGT

TTCTTCTCATCCAGCTCCTCGCGAATGAAATGAGAAGCGTGCAAATTTCTCTAATTCCAT

AATATTTTGGAATATTATGGATAGATGCACATTAATAGATAAAGTTAGGGTTTTTAATAT

TGAATTTGTTAAAATAGAAAAATATATAGTCAAGAAAGAAGATCTAGAATATATCTAGAT

TCTATATTTAATGTAATCTTTCTTAATCTCCTTATTTATTCAATAAAGATTTCGCGGGCG

AATATTTACTCTTTCCTGTCTTATTTGTTAATTTATAACCTTACCAAATAAGGCAATTTT

TTGGTTTGTCCGCCATCCACCAATGAAGTCTTAGGATTCTTTCAATAAATCCTATGCAGT

CATAGGTTCTGTCGTTCCCACTACTTCTCCTTTAATGGTTAGGTCTGAATCCCACAATGG

AGCTTTCAAAATTTCTTTCCGAGTCAATTTCTCAGTTTTATTAACCCGGCCGCTCTTTAT

TATTGCTTAAAATTTCTATTTTTTGTTTATTCTCTGTTATTTTTATTATATTGATGCTTT

ATCACATTGCCTTTTATGATGAACTCATAGACCATACATATTGGAATCCTATATCTTTCT

TATTCTTCTTCCTTCTTTCTATCATCCCTCCTTTTATCCACATCCCTTTAGTTTTGCTTC

ACAACCTAGAATCCTTTTCTTTTTTAGAGAAAAATTGCAGTTGCTACAACTATATGATAG

ATCTACTCATTTATGATAGATGTATCATATAGTGACTGTTTCTTAGTTAGGATCTCGACA

ATACGAAGCAATAGGTTGGTTATTAGTTAATTTTCTATAATTTTCCATTTTTGACTCTAA

AAAAAAACTAACAGTCACACACTAAGCATAGCAATTATATTAAAAGATTTATCAATTTTC

ATTAAATCTTATAGAAAGAGGTAGAATTCTTCTTTTTTTCAGGGATTTCAGGAAAAATAA

GGTCTTGTCTTTTTTATTCTATCACTGACAGAATGGGAAGACAAGGCTAGTTATTCTTCG

TCTACGAATATCCAAATTTTTACACCTAATACTCCATAGATAGTTCGAATTGGATAGCAG

CAATAATCAATTTTAGCGCGAATTGTTTGGAGGGGAAGTCTACCCTTTTTGATGCATTCG

GCACGTGCAATTTCTTTCCCTGCGAGACGACCTGCAATTTTTACTTTTACCCCCCTTATA

TCTGCTTTTTTAGTTAATTCAATGGCTTTTTTCATTGCCTTTCGGAATGAAACTCTATTT

TTTAATTGGAAAGCTATATATTCTGCAAGAATGTTAGGTTGTCTATAAGGTTCTTTAACT

TTTTCAATAGCAATATTAAGTCTCTGGTTTACAGAATTAACTTCCTTTTGTAGATCTTTC

TCTAATTCTTCGATTGCTCCTTTTTTCTTTAATAAATTGGGGAATCCAATATGGATTATG

ACGTGGATCGTATCGATTTCTTTTTGAATTTCTATACGTGTAATTACTTCAGAACTTGAG

CCTGAGTCCATTTTTCTATTCGAGCCTTTTTTCCTATTCTTTTGTATATAGTTCTTGATA

CAATTCCGTATTTTTTTATCTTCCTGTAGACCTTCAGAATAATTTTTTGGTTGTGCGAAC

CAAAAGGAATGGTGATTTTGGGTTGTACCAAGTCTGAAACCGAGTGGATTTATTTTTTGT

CCCATATTTTTCTATTCTATTTTTTTTACTGGGAATCAAATCTAAGATGGATCTAAAGAT

TATTTAGATTTCTTTACTATATTTAGTACAATTGTTATATGACACATGGTTTTTTTTATG

GGACAACTACGTCCTCGAGCTCGAGGTCTTAATTTTTTCATGATAGTACTCCTACTGACT

TCGGCTTTAGTGATGAATAAATTTGCTTTGTCGAAATCCCTATAATGAGTAGCATTTGCT

GCTGCCGAATAAACCAACTTTAGGATGGGATAAGATGCTCGATAAGGCATGAGGTTCAGT

ATCATAACAGTTTCCTCGTAGTAACGCCAGCGAATCTCATCAAGAACTCTTTGTGCTTTG

AAAACAGACATATGGATGCGTTTTTGTGCTTTGAAAACCCGTTCGAACTTAAGTAGACGA

TCGGTTGGAACTTTCCTTGCGAGTTTCCTTAGCCCCTTCTTCTTCTTTATCCTAGGGGTA

TACTTTACCAGTTTGAAACTTGTCATAAATAAGGTTATTCCCCGCCTACCTTTGTTTTTT

TTATTTTGAATCTTTCTATTCTGAATTCAGTTAACGACGAGATTTAGTATCTTTTCTTGC

ACTTTCATAACTCGTGAAATGCCGAGTAGGCACGAATTCCCCCAATTTGCGACCTACCAT

AGGATTTGTTATGTAAATAGGTATATGTTCCTTTCCATTATGAATCGCGATTGTATGGCC

AACCATTGCGGGTAGAATGCTAGATGCCCGGGACCACGTTACTATTGTTTCTTTCTCCTC

CTTCATATTGACCTTTTCGATTTTTGCCAATAAATGATGAGCTACAAAAGGATTCGTTTT

TTTTCGTGTCACAGCTGATTACTCCTTTTTCATTTTAAAGAGTGGCATTCTATGTCCAAT

ATCTCGATCGAAGTATGGAGGTCAGAATAAATAGAATAATGATGAATGGAAAAAAGAGAA

AATCCTTTAGCTGGATAAGGGGCGGATGTAGCCAAGTGGATCAAGGCAGTGGATTGTGAA

TCCACCATGCGCGGGTTCAATTCCCGTCGTTCGCCCATCGCATTATTGCAAATTCCAAAA

ATGCAATTTTCCATATTCCTAGTTACGTATTTACTTACGGCGACGAAGAATAAAACTATC

ACTATATTTTTTCCTTTTCCTAGTTCTTCTTCCAAGCGCAGGATAACCCCAAGGGGTTGT

GGGTTTTTTTCTACCAATGGGGGCTTTCCCTTCACCGCCCCCATGGGGGTGGTCCACAGG

GTTCATAACTACCCCTCTTACTACGGGGCGTTTACCTAGCCAACACTTAGATCCGGCTCT

ACCCAAACTTTTTTGGTTCACCCCAACATTACCCACTTGTCCGACTGTTGCTAAGCAATT

TTGGGATACCAAACGGACCTCCCCAGATGGTAATCTTAAAGTGGCCGATTTACCCTCTTT

TGCAATCAGTTTCGCTACAGCACCTGCTGCTCTAGCTAATTGCCCACCCCTTCCACGTGT

GATTTCTATGTTATGTATGGCCGTGCCTAAGGGCATATCGGTTGAAGTAGATTCTTCTTT

TCTCTCAAAAACCCCTTCCCAAACTGTACAAGCTTCTTCCAAAGCATACAGCTTTCTAGA

TGTATATGACGATCTCTAGACAGATGGATCTTATATGAATCGTATGATGAAGTACCACAT

GAGTGGATATATAGGAAAGGAATCCAAATCTGCCGAATCGCTCATGTTATGATCTTCTAC

ATCCTAGGTCTCCGCGTTCCGTCATCTGGCTTATGTTCTTCATGTAGCATTCAGATCGAA

TGACTCTATGAAATTACGTCGATACTTCCACATATTATGGGTAACGTAGGAGACATCCCT

ATTTTCCCCGGGGGGTCTTAATTACCACTGCTTAGCTTTCAATTCGCCTCTGACCATCAA

ATTAAATGTGAATAACCCGTCCTCCTCTCTTTGAAACAAGGGGCGCTTCCGGTTCTGTGC

GTGCTTCAAACAATTTTGTCTTCTCCATATTACCATATCTCTAGAGTCAATAATTTTCTA

TGAGGAACTACTGAACTCAATCACTTGCTGCCGTTACTCAACAGTTTTCTGTTGAGGTCT

ATCCCGTAGAGGTAGTCAAATTGGATCAGTGATCGATTTCTAGGTTTCGTCGTAAACCTA

ATTGGTTACTTCCAATTACGTAAATCAATAGTTCAAACCGCACTCAAAGGTAGGGCATTT

CCCATTGATATAGGAACTTTTGTACCAGAAACAATAGTATCTCCAATTATAGCCCCTCTG

GGATGTAAAATATATCTCTTCTCACCATCCCCATAGTGTATGAGACAAATGTATGCATTT

CGATTAGGGTCGTATTCTATGGTTACGATTCTACCAGATATGTCTTTTTGATTCCGTCGA

AAATCGATTTTACGGTATAGGCGCTTATGACCTCCCCCTCTATGCCTTGCGGTAATGATT

CCTCTGGAATTACGACCTTTACCACAACGGTGCCGTCCATGGATCAAATTATTTCGTGGA

TTGGATTTCACTTGCCTGTCTACGGTTCCCTTGCGTGTGCTCGGGATAGGTGTTTTGTAT

AAATGTTTCGCCGTATTATTAAGTATTCTCCTTTAGTTTTTTTCTCTATCTAGAAGTGGA

ATAGAATAACCCGGTTGAAGGGTAATGATCATACGTCTGTAATGCATTGTATGTCCCAGA

ATAGGTCCCATTCTTCTACCCTTTCCGGGTAGTCGATGGCTATTCACAGCTACTACCTTA

ACACCAAAGAAGAGTTCGACCCAATGCTTTATTTCTGTCTTAGTGAATCCCGATTCGACA

TTAAAAGTATATTGATTCTTTCCCAATAAACGAAGACTTTTTTCTGTAAATACTGCGTAT

TTGATTCCATCCATAAATCGACTTTCCCTCCTATGCTCTGAGTTCCAGTATCGATAAGAA

TTCGAGTTCTTATTGTTCTTATGTTATGGTATGAATATACCATACCAATTCGTTATGTAT

GGATGATGGATGAGATTCCATGGATAGAGAGCCAGTTCCAATAGACCGTTCCCGTTCGCG

TGCATCCAGCAGGAATTGAACCCGCAAATTTACCAATTATGAGTTGGGCGCTTTAACCAT

TCAGCCATGGATGCTTAACAGGGATCATCGTACATCGTAAATAACCAATTTTCATATAGA

AAGACATATCATAGAAAAATGAAATCGAAAATATTCGGAGATGGCAAATATTCGGAGATG

ACTATGAAACACCTCTCTGGATCCTCGAATTGAAAGAGAGATTGAGAGGGATCAAGAATC

CTAATTCTCGCTATTTGGAATGGATCCAATTCTATTGAGTCTGACTCATAGTGATCATTT

CTCTTTAGCAAAGAATGACCTTGGTTATCAAAGGATTGAACAACCGGGATCCATTTACTT

ATGATACCTAGTTGACATTGATAACAAGGATCTAATGAATTATGAGTTTAATAGATCCTC

TTTAGCAGAAAGACGTATATTCCTTGCTCATCACTTATCCCAAACTCGTGTGGGCTAATC

GTTTTCATTTACCATCTCATGGAAAACCCTTTTCGTTCCGCTTAGCCCTATCGGGTATTT

TAGTGATAGGTTCTATAGGAACTGGACGATCCTATTTGGTCAAATACCTAACGAAAAATT

CCTATTTTCCTTTCATTAAGGTACGAGGGCTTCTTATTCCACAAGAACGAAAGCACCTTT

TCATTCTTTCATATACTAGGGGTTTTTACTTGGAAAAGACAATGTTCCATACTAAAGGAT

TCGGGTCCATAACCACGAGTTCCAGTGCACTAGATCTTGTAGCACTTAGCAACGAGGCCC

TATGAATAGACATATAGAATTTTTGGTCGGGAAATTCGAATGAATCATTGAGTGAAAAAG

GAGCAAAGAATGACAAAAGACGAGACTCTACTAGTCTTCACTCTTGTGGTTTCCTCGGTT

TCTGTTTTCTTATTCGGGATCTTGCTTTTCATGGTTCTCATCTCTGCAACTCGCGATTTT

CGCGAGAGAACCAAATCCAAGTTGGTGAAGATCATGATTTGGGCTGGCATAGTAGTTATT

ACCTTTGCAATTGCGGTTCGAATCTATCCGATCTTTATCTTTTTGCTCAAAGAACGAATA

AAACCCCTTGTCGAAGCCCTTTATGATAAGCTTCCCTGGATCTGGGAAGTTTCTCTTTCA

CGGTATTGGGATCGTTTGATCGATTTCCTTGATCGCTACTTATGGGCGTGCGCTCAAAGG

ATACAAACAGGAATTCGCAAACAAAAAGGGGAATTCGTAGTCACTTTTTCCTGTCGCGTA

AAAAAAAGGCTTTACGCGAGAGCAATAGAGGTTGGGATACATCTATCTCTTCTGAGCAAC

CTCTTTTGGATTCTTAAGACCACCCTTGCAGTAGGATACCGTCTGCTTTGGGTTCTTTAT

TATCTCCTTCCAGGGATTTTTAGGATCGTTCAGGCTATATTTAGTCTATTTTGGCTTTTA

CTGTCTACTTTTCTCAGGGAGATGGTTAAGGACCTCAGAAGATAGAGGAGAGCGCCAGGC

GCAGATTTCCGGAATACTTCTACGGGGAATGCTCATTCATGAGCATTCTCCATATTATGC

CTTGAAGAGGACTCGAACCTCCACGCTCTTTAGCACGAGATTTTGAGTCTCGCGTGTCTA

CCATTTCACCATCAAGGCATCTTGAAAGTGAATCGTATTCCATGAATATGATATCTATCT

AATGTGATATATTCCATATATGACAAAGGTGGAGTCTTGGAGTATTTCGATCGATCGGTC

ATATAGGCCTGAGTCAGACATCAAATAGCTTCGATTTGCATTATCCGTAGAACACCTTAT

ATGTATCAAAATCGAAAAGATGTACAATCCAATTTCTCGATTCAATAGAAGCCCAAAGAG

GTGCATATGGTACCCAAATAAGGATAGGATAGATATGTCAAAAGCAGGTCTGATTACACC

TATTCCTAATCCTAAATAGAATGTAAGGGCGTAGGGATTTCTATGTAAACAGAGTATCCT

ATTTCCATAGGCTCGAATGACCCCTTCTCATAATAAGAATGTGCACGGTCTGGTCCGGTA

TGGAATGAACTTATAATCTGATGATCGAGTCGATTCCATGATTATAAGTTCATAACCCTA

GCGCCCATTCCCATTTTGGGCGGAACAGATCTACTAATTCTTTTATTCCAGTTAGTAAGA

GGGATCTTGAACTAAGAAATAGACCTAGCAGCTAAAAGAGGGTATCCTGAGCAATTGCAA

GAATGGGGTTCATTGATATTCCTGGTATAGTAGATGCTATCACACATACAGTCATACTCA

ATTCGATGGAATTGTTTGATCTTAAAGGGGATCTTCTATAATTTCGCACATAAGGGGTTA

TTTCTTGGTTTCGTCCAGTCATTAATAACTTGATTATTTTTAGATAATAGTAGATAGAAA

GAACGCTCGTAAGGAGTCCTATTGAAACCAAGAAATATAGGCCTGCTTGCCATCCACACC

AGAATAGATAGAGTTTTCCGAAGAAACCCGCTAGTGGAGGAAGGCCTCCTAGGGATAAGA

GACATAGGGCTAAAGAGAGAGCCAAAAAAGGATCTTTCGTGTATAATCCTGCATAATCTC

GAATGTTATCAGTTCCGGTACGTAGACCAAATAATACAATGCAAGCAAAAGTTCCTAGAT

TCATGGAGATATAGAACAGCATATAAGTTATCATGCTTGCATATCCATCATTTGAGTCTC

CAACAATTATTCCAATAATTACATATCCGATTTGCCCTATGGACGAATATGCAAGCATAC

GTTTCATGCTTGTTTGAGTAATAGCAAGGAGATTCCCCAATATCATGCTAAGAATAGCTA

GGATTTCCAGAAGAAGATGCCATTCGTTTGATGAGAAATAAAAAGGAATATCGAGAATTC

GCGTGGCTGAAGCTGAAGCAGCTACTTTCGAAGTAACAGAAAGAAAAGCAACGACTGGAG

TGGGGGAGTCAGAGTCGAAAAGAGGATTCCTCGCTTCTTTCTCTCATGCAAAACCGTGCA

TGAGACTTTCATCTCGCACGGCTCCTAAGTGATAAAAGAAAGAAGAACTCGTCTTCTTTC

TTTTTTGATTACCTTCCTCGCGTATGTATAAGACCCAATCCATTCTTTTTCGAAATCGAT

TTCGAAAAAGAACTACTAATCCTTAACTTTTCGAGGAATCCTTCATCAGTGGTTGTGAAT

GACTGACTTTTTCAATCCTTTCGACTTTGGTTCCGTAGGAGCAAGTCAGAAAGGTTGAGA

AATAGAACCATCTGATTTGATTCGTTCCCAATAGCCATGAGATGATTATCTTAGGGTGAT

CCTTTTGTCAACGGATGCTCCTATTACACTCGTAGTCTCTGAAGGATGAGAACCCACTAT

GTAGCATCTACATTGATAATTCAAGCATTGTATACGTCATTAGTCCGATTCTTTGTAGGA

ACTACCCGTAATAACGAGCTTGCAAAATGGATCTGTTTATCATAAAGAGATTCGTTGTTC

CTGACCCTGCTTCACCTTAATTGTTATTTGAACAAAAAGATCACAATAAACTTTTGGTAA

AAGTTCTGTCTTGGTCGGAGTGGGGATAGCATTTCTCTTCTGCATGTCTATGGAGTTTTG

CAAAACCCAAACACCTCAGATAGAGGTAGGAATTTGTCGAACGAACCACACTCCTTCGTA

GACGTCAGGAGTCCATTGATGAAAAGGGGCTGGGGAAAGCTTGAACCCAAGTCCTACAGT

GATGGATATAAGCGCAATTGAAATTCCTGGGGAGTTATACATTTGTGTATTGATAAGACC

GTTCACAATTTCTTGAAGCTCGATCTCCCCCCCAGATGAACCATATAGCCAAGAGAAACC

ATGAACCAGAATAGAAGAGCTTGCCCCACCCATGAGTAAATATTTCATAGTAGCCTCATT

AGACCGTAGATCTCTCTTGGTATATCCAGACAATAGGTAGGAACATAAACTGAAACATTC

TGGAGCTACAAAGATAGTTATTAAATCGTTAGCACCACATAAAAACATTCCCCCTAGAGT

AGCTGTTAATACGAATAACAGAAACTCTGTTATAGCCATTTCTGTACATTCAATGTACTC

TACGGATAGAGGAATACATAAAGTTGAACATAATAAAATAAGAAATTGAAAGATTTCGTT

GAAATTGTTCGTTTGGAAATTTCCCGAAAAGCTAATTATAGGTTCTTCTCTCCATCGGAA

CAATAGGGCCGTTATGCTTATTACTAAACTTGTTGAAGAGATGAAATAGAACCAAGGTCT

ATCTTTTTGATCAGAGGTTGAATCGATCATCAGAAGAAGAATTAGGCCAAAAATTAGGAT

ACATTCTGGGAAAATGAAACTTCCATGGAAGAGAAGCAAATGAAACGCTTTCATAAAAAT

TCTCGTAGAATCGAGAATGAAGTTTTCATTCTGTACATGCCAGATCATGAATTAGTAACT

GCATCCAATCTCCGAAAAGTCCCGATTGTTTCGATTTTTGGAATGGGATATTTACGGAAT

CCCCATGAATAGGATCAAACCTTATTCCATGCTATTTCCATAAGATTCCTCTTTCTTATT

CTTAAGCAAGCCCCCGAGAGGGCTTAGTTGATCATGATTTCTGTTTTCTCTTTCTTTTCC

TTTTTGTTTGTTTCGAGAAAGATATCGTCCGATTCTCCTTCTATTGATTCTTTTCCGATC

GAGATGTATGGATCCATGTGTCTACATACCTAGATTCTGTTCATGGATTAACGAAAATGT

GCAAGAGCTCTATTTGCCTCTGCCATTCTATGAGTCGCTTCCTTTTTGCGTATGGCACCC

CCACTCCCTTTGGCAGCATCTACTAATTCGGAACTTAATTTGAAAGCCATATTTCGACCC

GGACGCTTTTGGGATGCTTCTAATAACCAACGAATGGCAAGTGCTCTTCCTTGTTTAGAT

CCTATTTCAATCGGAACTTTCCGCGTCGATCCTTTTTTATTACGTCTTGTTTTTACTCCT

ATATTGGGAGTTACTCTACGTATTGCTTGACGTAAAACCAATAGTGGATTTGTTTCTGTC

TTTTGTTGAATCTTTTTCACGGCTCGATAGAGAATTTGATAAGCCAATGATTTTTTTCCG

TCTTTCATAATACGGTTAACCACCATGTTAACTAATCGATTACGAAAAATTGGATCGGAT

TTTGCAGTTCTTTTTTCTGCAGTACCTCGACGTGACATGAGCGTGAAAGAGGTTCAAGAA

TCCGTTTTCTTTTTATAAGGGCTAAAATCACTTATTTTTTTGGCTTTTTGACCCCATATT

GTAGGGTGGATCTCGAAAGATAGGAAAGATCTCCCTCCAAGCCGTACATACGACTTTCAT

CGAATACGGCTTTCCACAGAATTCTATAGGGATCTATGAGATCGAGTATGGAATTCTGTT

TACTCACTTTAAATTGAGTATCCGTTTCCCTCCTTTCCCCCTAGGATCGGAAATCCTGTA

TTTTCCATATCCATACGATCGAGTCCTTAGGTTTCCGAAATAGTGTAATGGAAAAAGAAG

TGCTTCGAATCATTGCTATTTGACTCGGACCTGTTCTGAAAAAGTCGAGGTATTTCGAAT

TGTTTGTTGACACGGACAAAGTAAGGGAAAACCTCTGAAAGAATTTCCATATTGACCTTG

GACATATAAGAGTTCCGAATCGAATCTCTTTAGAAAGAAGATCTTTTGTCTCATGGTAGC

CTGCTCCAGTCCCCTTACGAAACTTTCGTTATTGGGTTAGCCATACACTTCACATGTTTC

TAGCGATTCACATGGCATCATCAAATGATACAAGTCTTGGATAAGAATCTACAACGCACT

AGAACGCCCTTGTTGACGATTCTTTACTGCGACAGCATCTAGGGTTCCTCGAATAATGCG

ATATCTCACACCGGGTAAATCCTTAACCCTTCCTCCTCTTACTAATACTACAGAATGTTC

TTGTAAATTATGGCCAATACCTGGTATATAAGCAGTGATTTCAAATCCAGAGGTTAATCG

TACTCTGGCAACTTTACGTAAGGCAGAGTTGGGTTTTTTGGGGTTGATAGTGGAAAAGTC

GACAGATAAGTCACCCTTACTGTCCCTCTACAGAACCGTACATGAGATTTTCACCTCATA

CGGCTCCTCGTTCAATTCTTTCGAAGGGATCCTTTTCCTCGTTCGAGAGTCTCCGCCCTT

CTTCCACTCCGTCCCGAAGACTAACTAAGACCAATGGAGTCACGTTTTCATGTTCTAATT

GAACACTTTCCATTTATGATTAAAGGAGAAGATTGTTCTTTTACCAAACATATGCGGATC

AAATCACGTCTTATAATAAGAAGAAATCTTTCTCGGTATCAATCCCCTTGCCCCTCATTC

TTTGAGAATCAGAAGGATCCTTTTCGAGTTTCCATTTCTTCATTTGGAATCTGGGCTCTT

CTATCTTCGACTTATTTTTTGGCTTTATTCTTTATTTATTTCATTTCGATTTTTCCCTCT

TCCTCTATCCCTATCCTCTAGGTACAGCGTTTGCATCAATAGAGAACCTTTTCCTCTGTA

TGAATCGATATTATTCCAATTTCTTCCCGAAACTTCCCAAGAAAAATCCCGAATTGGATC

CAAAATTGACGGGTTAATGTGAGCTTATCCATGCGGTTAGGCACTCTTCAAATAGGAATC

CATTTTCTAACTGGCTTTCGTGCTTTGGTGAGTCGTCCGAGATCCTTTCGATGACCTATG

TTGTGTTGAGGGGATATCTATATGATCCGATCGATTGCATAAGACCCGCGGTAGCATAGA

ACGGGGAAAGTATACAGAAAAGACAGTTCTTTTCAATTTCGATTATCTATATATTAGTTC

GTTTCTATTTCTAGATATCTATTTCTATATATTAGTATTAGTTAGTAGTACTATTCTATT

AGTTAGCGATCCCGGCTCTGTGAGTTCTTTCTTCCGTGATGAACTGTCGGCACCAGTCCT

ACATTTTTTCTCTGTGGACCGAGGAGAAAGGGGGTTAGCAGGATTACCATGAGAGAACAA

CCCGCTTCAAATATGGAACATGGATTCTGGCAATGCAACGGAGTTGGGTCCTCATATCGA

TCCGAATGAATCAGTCTTTCTACAGAGGTCAATCTTTCTCGATAGGACATGGATTTCTAT

TCCTATGAAATTCATAAATTAGTTAATGGAGGGCTACCATTCCTTTTTCTTTATGTGTTC

CTAAGAGAAGGAATTTGTCCATTTCATGTTTCGAGGTCTCAAAAAAGGGCGTGGAAACAG

ATAGAAACTCTTGAATGGAAATTGAAAAGAAATGTAGCCCCAGTTCCTTCGGAAATGGTA

AGATCTTTGGCGCAAGAAGAAGGGGCGACCCGTATCATCTTGACTTGGTTCTGCTTCCCC

TCTTTTTTTAAGAATACCGAGTCGGGTTCTTCTCCTACCAGTATCGAATAGAACATGCTG

AACAAGATCTTCTTCATGGAAACCTGCTCGATTTAGATCGGGAAAATCGTACAGATTTTA

TGAAACCATGTGCTATGGCTCGAATCCATAGTCAATCCTACTTTCGATAGGACCGGTTGA

CAATTGAATCCAATTTTTCCCATTATTTGACTATCCATAATAGTGCGGAAAGAAAGCCCG

GAGGAAGAGTGGCCTTGAGTTTCTCGCCCCTTTGCCTTAGGATTCGTTAATTCTCTTTCT

CGATGGGACGGGGAAGGGATATAACTCAGCGGTAGAGTGTCACCTTGACGTGGTGGAAGT

CATCAGTTCGAGCCTGATTATCCCTAAACCCAATGTGAGTTTTTTCTATTTTGACTTACT

CCCCCGCCACGATCGAACGGGAATGTATAAGAGGCTTGTGGGATTGACGTGATAGGGTAG

GGTTGGCTATACTGCTGGTGGCGAACTCCAGGCTAATAATCTGAAGCGCATGGATACAAG

TTATCCTTGGAAGGAAAGACAATTCCGAATCCGCTTTGTCTACGAATAAGGAAGCTATAA

GTAATGCAACTATGAATCTCATGGAGAGTTCGATCCTGGCTCAGGATGAACGCTGGCGGC

ATGCTTAACACATGCAAGTCGAACGGGAAGTGGTGTTTCCAGTGGCGAACGGGTGAGTAA

CGCGTAAGAACCTGCCCTTGGGAGGGGAACAACAACTGGAAACGGTTGCTAATACCCCGT

AGGCTGAGGAGCAAAAGGAGAAATCCGCCCAAGGAGGGGCTCGCGTCTGATTAGCTAGTT

GGTGAGGCAATAGCTTACCAAGGCGATGATCAGTAGCTGGTCCGAGAGGATGATCAGCCA

CACTGGGACTGAGACACGGCCCAGACTCCTACGGGAGGCAGCAGTGGGGAATTTTCCGCA

ATGGGCGAAAGCCTGACGGAGCAATGCCGCGTGGAGGTGGAAGGCCTACGGGTCGTCAAC

TTCTTTTCTCGGAGAAGAAACAATGACGGTATCTGAGGAATAAGCATCGGCTAACTCTGT

GCCAGCAGCCGCGGTAAGACAGAGGATGCAAGCGTTATCCGGAATGATTGGGCGTAAAGC

GTCTGTAGGTGGCTTTTCAAGTCCGCCGTCAAATCCCAGGGCTCAACCCTGGACAGGCGG

TGGAAACTACCAAGCTGGAGTACGGTAGGGGCAGAGGGAATTTCCGGTGGAGCGGTGAAA

TGCATTGAGATCGGAAAGAACACCAACGGCGAAAGCACTCTGCTGGGCCGACACTGACAC

TGAGAGACGAAAGCTAGGGGAGCAAATGGGATTAGAGACCCCAGTAGTCCTAGCCGTAAA

CGATGGATACTAGGTGCTGTGCGACTCGACCCGTGCAGTGCTGTAGCTAACGCGTTAAGT

ATCCCGCCTGGGGAGTACGTTCGCAAGAATGAAACTCAAAGGAATTGACGGGGGCCCGCA

CAAGCGGTGGAGCATGTGGTTTAATTCGATGCAAAGCGAAGAACCTTACCAGGGCTTGAC

ATGCCGCGAATCCTCTTGAAAGAGAGGGGTGCCCTCGGGAACGCGGACACAGGTGGTGCA

TGGCTGTCGTCAGCTCGTGCCGTAAGGTGTTGGGTTAAGTCTCGCAACGAGCGCAACCCT

CGTGTTTAGTTGCCACTATGAGTTTGGAACCCTGAACAGACCGCCGGTGTTAAGCCGGAG

GAAGGAGAGGATGAGGCCAAGTCATCATGCCCCTTATGCCCTGGGCGACACACGTGCTAC

AATGGGCGGGACAAAGGGTCGCGATCTCGCGAGGGTGAGCTAACTCCAAAAACCCGTCCT

CAGTTCGGATTGCAGGCTGCAACTCGCCTGCATGAAGCAGGAATCGCTAGTAATCGCCGG

TCAGCCATACGGCGGTGAATCCGTTCCCGGGCCTTGTACACACCGCCCGTCACACTATAG

GAGCTGGCCATGTTTGAAGTCATTACCCTTAACCGTAAGGAGGGGGATGCCTAAGGCTAG

GCTTGCGACTGGAGTGAAGTCGTAACAAGGTAGCCGTACTGGAAGGTGCGGCTGGATCAC

CTCCTTTTCAGGGAGAGCTAATGCTTATGCTTATTGGGTATTTTGGTTTGACACTGCTTC

ACGCCCAAAAAGAAGGCAGCTACGTCTGAGCTAAACTTGGATATGGAAGTCTTCTTTCGT

TTAGGGTGAAGTAAGACCAAGCTCATGAGCTTATTATCCTAGGTCGGAACAAATTAGTTG

ATAGTGATAGGATCCCCTTTTTGACGTCCCCATGTCCCCCCGTATGGCGGCATGGGGATG

TCAAAAGGAAAGGGATGGAGTTTTTCTCGCTTTTGGCGTAGCGGCCTCCCTTTGGGAGGC

CGCGCGACGGGCTATTAGCTCAGTGGTAGAGCGCGCCCCTGATAATTCGTCGTTGTGCCT

GGGCTGTGAGGGCTCTCAGCCACATGGATAGTTCAATGTGCTCATCAGCGCCTGACCCGA

AGATGTGGATCATCCAAGGCACATTAGCATGGCGTACTCCTCCTGTTTGAATCGGAGTTT

GAAACCAAACAAACTTCTCCTCAGGAGGATAGATGGGGCGATTCAGGTGAGATCCCATGT

AGATCTAACTTTCTATTCACTCGTGGGATCCGGGCGGTCCGGGGGGGGCACCACGGCTCC

TCTCTTCTCGAGAATCCATACATCCCTTATCAGTGTATGGAGAGCTATCTCTCGAGCACA

GGTTGAGGTTCGTCCTCAATGGGAAAATGGAGCACCTAACAACGCATCTTCACAGACCAA

GAACTACGAGATCACCCTTTCATTCTGGGGTGACGGAGGGATCGTACCATTCGAGCCTTT

TTTTCATGCTTTTCCCGGCGGTCTGGAGAAAGCAGCAATCAATAGGACTTCCCTAATCCT

CCCTTCCTGAAAGAAGAACGTGAAATTCTTTTTCCTTTCCGCAGGGACCAGGAGATTGGA

TCTAGCCATAAGAGGAATGCTTGGTATAAATAAGCCGCTTATTGGTCTTCGACCCCCTAT

ACGAGCGCCCCCGATCAGTGCAATGGGATGTGGCTATTTATCTATCTCTTGACTCGAAAT

GGGAGCAGAGCAGGTTTGAAAAAGGATCTTAGAGTGTCTAGGGTTGGGCCAGGAGGGTCT

CTTAACGCCTTCCTTTTTCTGCCCATCAGAGTTATTTCCCAAGGACTTGCCATGGTAAGG

GGGAGAAGGGGAAGAAGCACACTTGAAGAGCGCAGTACAACGGGGAGTTGTATGCTGCGT

TCGGGAAGGATGAATCGCTCCCGAAAAGGAGTCTATTGATTCTCTCCCAATTGGTTGGAT

CGTAGGGGCGATGATTTACTTCACGGGCGAGGTCTCTGGTTCAAGTCCAGGATGGCCCAG

CTGCGCAGGGAAAAGAATAGAAGAAGCATCTGACTCTTTCATGCATACTCCACTTGGCTC

GGGGGGATATAGCTCAGTTGGTAGAGCTCCGCTCTTGCAATTGGGTCGTTGCGATTACGG

GTTGGCTGTCTAATTGTCCAGGCGGTAATGATAGTATCTTGTACCTGAACCGGTGGCTCA

CTTTTTCTAAGTAATGGGGAAGAGGACTGAAACATGCCACTGAAAGACTCTACTGAGACA

AAAAGATGGGCTGTCAAAAAGGTAGAGGAGGTAGGATGGGCAGTTGGTCAGATCTAGTAT

GGATCGTACATGGACGATAGTTGGAGTCGGCGGCTCTCCTAGGCTTCCCTCATCTGGGAT

CCCTGGGGAAGAGGATCAAGTTGGCCCTTGCGAATAGCTTGATGCACTATCTCCCTTCAA

CCCTTTGAGCGAAATGTGGCAAAAGGAAGGAAAATCCATGGACCGACCCCATTGTCTCCA

CCCCGTAGGAACTACGAGATCACCCCAAGGACGCCTTCGGCGGGGGTCACGGACCGACCA

TAGACCCTGTTCAATAAGTGGAACACATTAGCCGTCCGCTCTCCGGTTGGGCAGTAAGGG

TCGGAGAAGGGCAATCACTCGTTCTTAAAACCAGCATTCTTAAGTTAAGATCAAAGAGTC

GGGCGGAAAAAGGGGAGAGCTCCCCGTTCCTGGTTCTCCTGTAGCTGGATTCCCCGGAAC

CACAAGAATCCTTAGAATGGGATTCCAACTCAGCACCTTTTGTTTTGAGATTTTGAGAAG

AGTTGCTCTTTGGAGAGCACAGTACGATGAAAGTTGTAAGCTGTGTTCGGGGGGGAGTTA

TTGTCTATCGTTGGCCTCTATGGTAGAACCCGTCGGGGAGGCCTGAGAGGCGGTGGTTTA

CCCTGTGGCGGATGTCAGCGGTTCGAGTCCGCTTATCTCCAGCCCGTGAACTTAGCGGAT

ACTATGATAGCACCGAATTTTGCCAATTCGGCAGTTCGATCTATGATTTCGCATTCATGG

ACGTTGATAAGATCCTTCCATTTAGTAGCACCTTAGGATGGCATAGCCTTAACGTTAATG

GCGAGGTTCAAAAGAGGAAAGGCTTGCGGTGGATACCTAGGCACCCAGAGACGAGGAAGG

GCGTAGCAAGCGACGAAATGCTTCGGGGAGTTGAAAATAAGCATAGATCCGGAGATTCCC

AAATAGGTCAACCTTTTAAACTGCCTGCTGAATCCATGAGCAGGCAAGAGACAACCTGGC

GAACTGAAACATCTTAGTAGCCAGAGGAAAAGAAAGCAAAAGCGATTCCCGTAGTAGCGG

CGAGCGAAATGGGAGCAGCCTAAACCGTGAAAACGGGGTTGTGGGAGAGCAATACAAGCG

TTGTGCTGCTAGGCGAAGCGGTTGAGTGCCGCACCCTAGATGGCTAAAGTCCAGTAGCCG

AAAGCATCACTGCTTACGCTCTGACCCGAGTAGCATGGGGCACGTGGAATCCCGTGTGAA

TCAGCAAGGACCACCTTGCAAGGCTAAATACTCCTGGGTGACCGATAGCGAAGTAGTACC

GTGAGGGAAAGGTGAAAAGAACCCCCAGTGGGTAGTGAAATAGAACGTGAAACCGTGCTG

AGCTCCCAAGCAGTGGGAGGGGAAAGTGATCTCTGACCGCGTGCCTGTTGAAGAATGAGC

CGGCGACTCATAGGCAGTGGCTTGGTTAAGGGAACGGAACCCACCGGAGCCGTAGCGAAA

GCGAGTCTTCATAGGGCGATTGTCACTGCTTATGGACCCGAACCTGGGTGATCTATCCAT

GACCAGGATGAAGCTTGGATGAAACTAAGCAGAGGTCCGAACCGACTGATGTTGAAGAAT

CAGCGGATGAGTTGTGGTTAGGGGTGAAATGCCACTCGAACCCAGAGCTAGCTGGTTCTC

CCCGAAATGCGTTGAGGCGCAGCAGTTGACTGGACATCTAGGGGTAAAGCACTGTTTCGG

TGCGGCTGCGCGAGCGGTACCAAATCGAGGCAAACTCTGAATACTAGATATGACCCAAAA

ATAACAGGGGTCAAGGTCGGCCAGTGAGACGATGGGGGATAAGCTTCATCGTCGAGAGGG

AAACAGCCCGGATCACCAGCTAAGGCCCCTAAATGACCGCTCAGTGATAAAGGAGGTGGG

GGTGCAAAGACAGCCAGGAGGTTTGCCTAGAAGCAGCCACCCTTTAAAGAGTGCGTAATA

GCTCACTGATCGGCGCCCTTGCGCTGAAGATGAACGGGGCTAAGCGATCTGCCGAAGCTG

TGGGATGTCAAAATGCATCGGTAGGGGAGCGTTCCGCCTTAGATGGAAGCAAACGCGAAA

GCGGGGGTCGACGAAGCGGAAGCGAGAATGTCGGCTTGAGTAACGAAAACATTGGTGAGA

ATCCAATGCCCCGAAAACCCAAGGTTTCCTCCGCAAGGTTCGTCCACGGAGGGTGAGTCA

GGGCCTAAGATCAGGCCGAAAGGCGTAGTCGATGGACAACAGGTCAATATTCCTGTACTA

CCCCTTGTTGGTACGGAGGGACGGAGGAGGCTAGGTTAGCCGAAAGATGGTTATAGGTTT

AAGGACACAAGGTGACCCTGCTTTTTCAGGGTAAGAAGGGGTAGAGAAAATGCCTCGAGC

CGAGGTCCGAGTACCAAGCGCTGCAGCGCTGAAGTATGAGCCCCGTGGACTAGCCATTGC

TTCTCCACGAGCCTCATACCAGGCGCTACGGCGCGAAGTATGTAACCCATGCCATACTCC

CAGGAAAAGCTCGAACGACCTTCAACAAAAGGGTACCTGTACCCGAAACCGACACAGGTG

GGTAGGTAGAGAATACCTAGGGGCGCGAGACAACTCTCTCTAAGGAACTCGGCAAAATAG

CCCCGTAACTTCGGGAGAAGGGGTGCCCCCTCGCAAAAGGGGGTCGCAGTGACCAGGCCC

GGGCGACTGTTTACCAAAAACACAGGTCTCCGCAAAGTCGTAAGACCATGTATGGGGGCT

GACGCCTGCCCAGTGCCGGAAGGTCAAGGAAGTTGGTGAACTGATGACAGGGAAGCCGGC

GACCGAAGCCCCGGTGAACGGCGGCCGTAACTATAACGGTCCTAAGGTAGCGAAATTCCT

TGTCGGGTAAGTTCCGACCCGCACGAAAGGCGTAACGATCTGGGCACTGTCTCGGAGAGA

GGCTCGGTGAAATAGACATGTCTGTGAAGATGCGGACTACCTGCACCTGGACAGAAAGAC

CCTATGAAGCTTTACTGTTCCCTGGGATTGGCTTTGGGCCTTTCCTGCGCAGCTTAGGTG

GAAGGCGAAGAAGGCCCCCTTCCGGGGGGGCCGAGCCATCAGTGAGATACCACTCTGGAA

GAGCTCGGATTCTAACCTTGTGTCAGACCCGCGGGCCAAGGGACAGTCTCAGGTAGACAG

TTTCTATGGGGCGTAGGCCTCCCAAAAGGTAACGGAGGCGTGCAAAGGTTTCCTCGGGCC

AGACGGACATTGGTCCTCGAGTGCAAAGGCAGAAGGGAGCTTGACTGCAAGACTCACCCG

TCGAGCAGAGACGAAAGTCGGCCTTAGTGATCCGACGGTGCCGAGTGGAAGGGCCGTCGC

TCAACGGATAAAAGTTACTCTAGGGATAACAGGCTGATCTTCCCCAAGAGTCCACATCGA

CGGGAAGGTTTGGCACCTCGATGTCGGCTCTTCGCCACCTGGAGCTGTAGGTGGTTCCAA

GGGTTGGGCTGTTCGCCCATTAATGCGGTACGTGAGCTGGGTTCAGAACGTCGTGAGACG

TTCGGTCCATATCCGGTGTGGGCGTTAGAGCATTGAGAGGACCTTTCCCTAGTACGAGAG

GACCGGGAAGGACGCACCTCTGGTGTACCAGTTATCGTGCCTACGGTAAACGCTGGGTAG

CCAAGTGCGGAGAGGATAACTGCTGAAAGCATATAAGTAGTAAGCCCACCCCAAGATGAG

TGCTCTCTCCTCCGACTTCCCTAGAGCCTCCGGTAGCACAGCCGAGACAGCGACGGGTTC

TCCACCCATACGGGGATGGAGCGACAGAAGTATGGAAATAGGATAAGGTAGCGGCGAGAC

GAGCCGTTTAAATAGGTGTCAAGTGGAAGTGCAGTGATGTATGCAGCTGAGGCATCCTAA

CGAACGAACGATTTGAACCTTGTTCCTACACGACCTGATCAAATTGATCAGGCACTTGCC

ATCTATCTTCATTGTTCAACTCTTTGATGAAAAGATGAAAAACCAAAAAAAGCTCTGCCC

TTCCATCTCTTGGATAGATAGAGAGGGAGGGCAGAGGCCTTTGGTGTCCCTTCCAGTCAA

GAATTGGGGCTTCACAATTACTAGCCAATATTTATCTCATGCCTTTCCTCGTTCATGGTT

CGATATTCTGGTGTCCTAGGCGTAGAGGAACCACACCAATCCATCCCGAATTTGGTGGTT

AAACTCTACTGCGGTGACGATACTGTAGGGGAGGTCCTGCGGCAAAATAGCTCGATGCCA

GAATGATAAAAAGCTTAACACCTCTTATTTGACTTTTTCACTATTTTGAAATACGAAAAA

GATCCAAATCCAAAATGCAAAGGTCGTCTTATTCAAAACCTCAATCATCCCCTCCTCCCA

CTTCACACCTCGGAACGCACTGTTCTTATAGAGAGAAAGGCGCTTTCCCATCTTCTTAAC

CCGAAATGAAATGGCTGAGGAGAGGAAGGTTCCTTTTGGGGGGTACCCCCGGGAAGAGAT

CCAGTGGAGACGGGGTGGGCCTGTAGCTCAGAGGATTAGAGCACGTGGCTACGAACCACG

GTGTCGGGGGTTCGAATCCCTCCTCGCCCACAGCCTTCCAAAGGGGAAGGGCCTTTACTT

TCCCCCTGAGGGTAGGAAAATCATGATCGGGATAGCGGACGTAAAGCTATTGAACTTGGG

TATGCTCTTTCCTTTTGTCGAAGTGGAATCGTAGAACAGAATGTGATACGATGAGATAGA

ATGCATAGAAACAAGGATAGCGAACGGGTTACCTACTCCTAAGGGTCAAAGCAAGCCCTT

TAATTCAATTCTTTATTCTTACATTAAAGAATGAATCAAATCTCCCCAAGTAGGATTCGA

ACCTACGACCAGTCAGTTAACAGCCGACCGCTCTACCACTGAGCTACTGAGGAACAAGGG

GGATTCGACCTCCTAGAGTTCAACTCCCGCTCTCAACCCATGAACAATATGAGTCCGAAG

CTTCTTTCGTAACTCCCGGAATTTCTTCGTAGTGACTCCGTTCCATGCCTCATTTCATAG

GGAAGCCCAAAGTGGCTCTATTTCATTCTATTTCACTTCCTAGCACTTCCTATCATTTAA

TATCCATCCCTTTGGTCTTATTTACATAAGAGATGTCATTTATAGTCTATCTCTTTCTAT

ATATGGAAAGTCAAGAAATTCTCATCGAAACATCGAGAAATTGTGCATATAGAAAACTCT

AAAGAAAGAAAAAAGGAGACCCATGCCATGATTTTCAAATCTTTTCTACTTAGTAGTCTA

AGTTTCTCGATGAGGATAATTAATTCGGTCGTTGTGGTCGGACTCTATTATGGATTTCTG

ACCACATTCTCCATAGGTCCCTCTTAGATCTTCTTTCTCCAATCTTGGATTAGGGAAGAA

GGAGATATTCGCGACTACTGGCGGTTTCATTATGGGGCAGCTCATGATCTTCATATCGAT

CTATTATCCACCTCTGCATCTATTCTTTCTTAGCTAAACGGGTGGAAGATCCATCCAATT

TGGTTATATCATGGACTCAAAAGCGGATCTGAATGTGACTGAAATGCACGATCTTCACAG

GTATCACTTTTCACGATACCTAAAGATGGAATAGCGATTTGAACCATTTCCTATACGAGA

ATGGTTTCCATTACTTTGAGAAATGGATTCTATATCAAACTATAGCTATTGCATTAAAGA

AGAAAAGAAACTAATAGAAGTCGAAGACGCGGAATGGTAGTGAATAGAGAGAAAGATTCT

TCTGATTTTCTTGTTCCTGAAAATATTCTATCTATCTCCTAGACGCCGTAGAGAATTGAG

AATTTTCATGTCTTTCAATTCTCGTACTCGTAATTGGAAAGTTACGGAAGGAGGTCCATC

ATTTTGCAATGAAAACAACATAAAAAACTCTGGACAATTTCGAAATCAGGCCAAGCGTCT

TAATACATATGCAAAAAAATTCATTATTGGCCCACCATTGATTAGAAGATTTAGCTTGTA

TGAATCGCTATTGGTTTGATACGAATAATGGCAGTCGTTTCAGTATGTTAAGGATACAGA

TGTATCCACAATTCATTTAGAGTTACTTAATAGCCTATTTCTTATACCATATCTCTATCC

CGTGAAATTCTCGAGCCGAAAGATGGATGCATATGCTGTGTTTCATTTTGCTAAACGATA

TCAATTAAATGGTGTATCAATTCCATAAATTGGATATAGCAATAAATAAATCAGCAAAAT

TCTTTTATTTTAGATAGAAGAAATGTTTCTTCTATCTAAAATAAAAGAATGTACCCTTCT

ATCCAAATCCAATTTGCATCGATAAAATAAATCCAAATTCCAGTAGTGGATGAATAATTG

CAAATTTTTGTGTGTACGAGATTAGAATAACTTCAAAATAACTGACATAATTTTTTATTT

TTCCTGATCAGAAAAATACATGAAAAAGAAAGGAGGTAGAAAAATTTTGGGATTTATGGT

TAAAGAAGAAAAAGAAGAAAACAGGGGTTCTGTTGAATTTCAAGTATTCAGTTTCACCAA

TAAGATACGGAGACTTGCTTCACATTTGGAATTACACAAAAAAGATTTTTCATCGGAAAG

AGGTCTACGAAGACTTTTGGGAAAACGTCAACGTTTGCTGGCTTATTTGGCAAAGAAAAA

TAGAGTACGTTATAAGAAATTAATCAGTCAGTTGGATATTCGGGAGAAGTAATTTAATCG

TTCGAATTTTTTTCTTATTTTATTAGTAGTCTTATAGTAGTCTTAGATTTTTCATTTTGA

TGAGCCTCGTTTTGAGGAATTCATGGAATAATCCATTTTCATGGAATAAAGAATAAGAAC

ACGGATACATAACATAAAAAAAAGAATAAATAAGACGATATTCGCCCTCCCCCTACATAT

TTAATTTCTTCTCCTATACAAAAACCAGCAAGACCTACTCCATTGGTAATTCCATCAATG

ACACCCTTATCGAAAAACTGCGTTAGTTCAGTTAATCCTCTTATACCCAGGGTAAAGACC

CTAGTATAGAAAATATCTATATAACCACGATTATATGACCAACTGTATATCTTTTTTTTT

ACTTGATCCGAAAAAAACTTTTTCGGACCCTCTTTTACAAGAGAATTTATTAAATCCAAA

TTCTGAAAAAAGGAATAAGCGGATCCATAGAAGATATATGCTATGGATAGACCAAACATA

GCTAGACTTACAGAAGAAATTGCATTAGTGATAAATTCATATGAATTTATGGAAGAATTA

GAACTTTCCTGGAAAAAGTTTATTGAGGGAGTTAACCACTTTGATAATATGGTTAACTCC

GCTATTTCATTATCAAAATGGATTCCTATGGATCCAATGAACAAAGTAAAAAGCAGTAAT

ATAAAAAGAGGGAATAGCATAGTATTTCCCGTTTCATGAGGATAGACAAAAGTGTTTTTA

GCCCCAAAGGAAGTACTAAAGGACCCTATCCTATTTCTTGTATTACCATGAATTTTGGAT

CTATTTTGTGAAAAAAAAGAAACTCCACTCTTCGTTGTTGATAAAATGAAATCTCTATTC

ACTCCTTTGGGTATCCTTTTTCCCCATAAGGATATTGAATACAACGAACCCTCTTTAGTG

CTACTGTAATTTTGAAAATGAACACGCAGGTACCCATCAAAAGTAAGTAAATATATCCGA

AACATATAAAACGCAGTTAATCCTGCAGTAAAAGAGGCTATTATTCCAAAAAAGGGTGAA

TACAACCAACTATTACTAAGGATTTCATCTTTGGACCAGAAGCAAGCAAGAGGTGGAATA

CCACAAAGAGAAAGCGTACCCCATAAAAAAGTAGTTCTTGTAATTGGAACGTATTTTCTT

AAACCACCCATAAGAACCATATTCTGACTTTTATCTGGTGAATATCCAACAAGAGGTTCC

ATTGAATGAATAATGGATCCGGATCCCAAGAACAATAAAGCTTTCGAATAAGCATGAGTG

ATCAAATGGAATAAAGCAGCTTGATAAGAACCTATACCTAGAGCTAACATCATATAACCC

AATTGAGACATTGTAGAATAGGCTAAGCTTCTTTTAATATCTCTCTGAGCAAGAGCTAAA

GTAGCTCCTAAGAAGAGTGTTATTGTACCTACTAAAGAAATGAAACTCATTATTAAAGGT

AGGGATATGAAAAGAGGAAGAAGTCGAGCTAGAAGAAAAATCCCCGCAGCAACCATAGTT

GCTGCGTGTATAAGAGCCGAAATGGGAGTGGGTCCTTCCATAGCATCGGGTAACCATACG

TGAAGAGGGAATTGTGCAGATTTCGCAACTGCACCAAGGAATAATAAAAAAGCACACAAA

GTAGTAAGTAAGGAATTAATCCCATTATTAGGAATCCAGTTATTAGCTATTTTGAACAAA

TCCCTAAACTCTAAACTACCTGTTATCCAAAAAAAACCTAAAATTCCTAATAACAGACCA

AAATCCCCTACACGATTAGTTACAAAAGCTTTTTGACAAGCACTCGCTGCAATTGGCCGT

GTAAACCAAAAGCCTATCAATAAATAGGAACACATTCCCACAAGTTCCCAAAAAAAATAA

ATTTGTATCAAATTGGAACTAGTAACCAATCCCAACATGGAAGTATTGAAAAAACTTATA

TAAACAAAAAATCTCAAATATCCTTCATCGTGAGACATATAATCGTCACTATAAATAAGA

ACGAGGATTCCTACAGTAGTAATTAGTATTAACATAATAGAAGTAAGCGGGTCGATCAAG

TATCCAAATTCTAAGGAAAAATCATTATTGACGGTCCAAGACCATAGATATTGATAGATA

GAACTTCCATTTATTTGTTGAATAGATAGGTGAACTGAGAATACCATAGCTATACTTAAG

AGTAAAACACAAGGAAAAGCCCATATGCGACGAAGATTTTTTGTTGCTGTCGGAATAAGA

ATAAGTCCAAACCCCATTGACATAATAACTGGAAGTGGGAGAAGAGGGATTACCCATGCA

TATTGATATGTATGTTCCATAAGAAAAGAAATTGCAATTTTTCTATAAAATAAAATTGTT

TCCGATTCACCAAACCAATTCTTATCTCTTTCTGAAGGAATTCCAAAATACTGGAATTCT

TCATTTTTCCAATTTCTCTCATTGAAATATCAAAAATGAAGAATGGGTTTACTTGGTTAA

ATTCAAAAAGTTAATTAAATAACTTTGTTACCTAGTTATTACTAAAGAAGGATATTTGTT

AAAATACAAAAAAGGATTTAATCATTTTACTTTTGTATTTCTTTCTATTAAAATGAAGCA

GCTCTCATGTTTCGTAACTGAAATTGATTGGAATTCCTTAATTAACTATTTGAATTTTCC

CTTCCTTTTATCCCCCATCTTATATGGGGGATAGGCCGTAGATCTATATATGGAGTATAC

TTAATATTAATTTAAATAGAAAACAAAAAATTCTTGTCTTATCCGCATTAGAGAAAATAA

AGTAAAAAAGAATTCAGAATTTCAGTTCAGTATCTAGTATAAATACTAAGAAAAAACAGA

AAGAAGGATTGATTTGCGGCAATAGATGTCTTTCACATACAACTAGAAAAAGTAATCTCC

TTTTTGAATGGCAGTTCCAAAAAAACGTACTTCGATGTCAAAAAAGCGTATTCGTAAAAA

TCTTTGGAAGAAAAAGACTTATTTTTCCATAGTACAATCTTATTCTTTAGCAAAATCAAG

ATCATTTTCCAGCGGTAGCGAGCATCCAAAACCAAAGGGTTTTTCTGGGCAACAAACAAA

TAATCTGGTTTTGGAATAATCTGAATTGACCTATCCCAAAGAAATTCCAATTATTTAAAA

TGAATAATTCGGATTAATTAATGAATGTACTTTTATGTGTCGAATTCCTCGGTACAATAT

TCTTAGAACTAACCCCTCTGATATATAGAACAAAAGTTTTTGGTATACTGTGTCCTAAGT

ATTCTTTTCCTATCAACGAACTTTTCATAATAGAATCCTCAAATATGAGGATTCTATTAT

GAAAAGTAGAGTATTCTTGCAATAGGACTTACAACTTCTACCTATCTTATCAAAAATCCA

TTGCAACTGAAAAAAAATCCCCAATGATGAAATTCTAATGTCCTAAATTCTATGGACTCT

CCCAATCTCGACGATTTGCGAGAAAATAACTATTATTCTTTTAACTTCCCTATTATTTAA

AGTTAGCCGCCATGGTGAAATTGGTAGACACGCTGCTCTTAGGAAGCAGTGCTCAAGCAT

CTCGGTTCGAGTCCGAGTGGCGGCATTCTCGAAAAAGAATACAATAGATTAGAAATGATT

CAATTCGAAATTTCCAATTTTGTAATGGGACCTTCTCCTTATGCTATTTGCAACTTTAGA

ACATATACTAACTCATATCTCTTTCTCAACCATTTCAATTGTGATTACAATTCATTTGAT

AACCTTATTAGTTCGTGAACTTGGGGGATTACGTGATTCGTCAGAAAAAGGAATGATAGC

TACTTTTTTCTCTATAACAGGATTCTTAGTTTCTCGTTGGGCTTCTTCGGGACATTTTCC

ATTAAGTAATTTATATGAGTCATTGATCTTCCTTTCATGGGCTCTGTATATTCTTCATAC

GATTCCTAAGATACAGAACTCTAAAAATGATTTAAGCACAATAACTACGCCAAGTACTAT

TTTAACGCAAGGCTTTGCCACGTCGGGTCTTTTAACTGAAATGCATCAATCCACAATACT

AGTACCTGCTCTACAATCTCAGTGGTTAATGATGCATGTCAGTATGATGTTACTAAGCTA

TGCAACTCTTTTGTGCGGATCCTTATTATCCGCCGCTCTTCTAATCATTAAATTTCGAAA

GAATTTCAATTTCTTTTTAGAAAAGAAAAATGTTTTAAATAAAACATTTTTCTTTAGTGA

GATTGAATATTTCTATGTAAAAAGAAGTGCTTTAAAAAACACCTCTTTTCCTTCATTTCC

AAATTATTACAAATATCAATTAATTGAGCGTTTGGATTCTTGGAGTTATCGTGTCATTAG

CCTAGGGTTTACCCTTTTAACCATAGGTATTCTTTGTGGAGCAGTATGGGCTAATGAGGC

GTGGGGATCCTATTGGAATTGGGATCCTAAGGAAACTTGGGCATTTATTACTTGGACCAT

ATTCGCAATTTATTTACATAGTAGAACAAATCCAAATTGGAAGGGTACGAATTCCGCACT

TGTAGCTTCGATAGGATTTCTTATAATTTGGATCTGCTATTTTGGTATCAATCTATTAGG

AATAGGTTTACATAGTTATGGTGCATTTACATTACCATCTAAATGATTACATAACATAAA

ACCTTCGAGTTTCCATTTTTGTTTGATTTGAGAACCCTTGAACGCCTTCTCAAAGGGTTC

TCAAAAATTCGAGATAGATCTAATTAGACTTTTTTACTTTTTTCTGAATTTTTCACTATG

GAATATAGAGCGGACTAGTAAAAAAAAATTATTTAGGATAATAATTGGATAAGAGAGCCT

CTACCTTGTCAACCGATAGCGAGAGAACAAAATCTGGATAAATACCAATTCCTATTACTG

GTAAAAAGATACAGATTAAAAGAAAGAGTTCTCGTGGTCCAGAATCCACCAAATTTGCGT

TTGGAACATGAAATAGCTTGTATCCATAGAACATCTGGCGTAACATAGATAATAAAAAAA

TAGGAGTTAATATCATTCCAATTGCCATTACAAAAGTAATTAGCATTTTTGGTATTAACA

GAAATTTTGGACTAGTAATTAGTCCAAAAAATACTACTAATTCTGCAACAAAACCGCTCA

TTCCTGGTAAGGCAAGAGAAGCCATTGAAAAGCTACTAAACATGGTAAAAATTTTCGGCA

TTGGGATAGATATCCCCCCCAGTTCTTCGAGATAAACAAGACGCATTCTATCACAAGCCG

TTCCCGCCAAGAAAAAAAGTGTAGCACCAATAAATCCATGGGATAGTATTTGTAAAATAG

CTCCATTGAGTCCAATGTTGGTTATGGAACCAATTCCTATAATTATGAAACCCATGTGAG

ATACGGAGGAGTAGGCTATTCTTTTTTTGAAATTTCGTTGACCAAGAGAAGTTGAAGCTG

CATAGATTATTTGCATCGCTCCTATTATTACCAACCAGGGGGAAAATAGATAATGAGCAT

GAGGTAACAATTCCATATTGATCCGAATCAATCCGTATGCTCCCATCTTTAATAGGATTC

CCGCTAAAAGCATACATGTACTGTAATGCGCTTCCCCATGGGTATCTGGTAACCACGTAT

GTAGGGGTATAATCGGCAATTTGACAGCATAAGCAATAAGGAAGCCAAAATAAAATAGTA

TTTCCAATGTTGCAGGGTATGATCGATTAATTAATCTTTCCAAATCTAATCTTGGTTCGT

TGGAACCATATAAGCCCATACCTAGAACTCCGATTAAGAAAAAAATGGAACCGCCTGCAG

TATACAAAATAAATTTTGTAGCTGAATACAGACGCCTCTTTCCCCCCCACATGGATAAAA

GTAAGTAAACAGGAATTAATTCTAACTCCCACATGATAAAAAAAAGTAAAAGGTCTCGCG

AAGAAAATAATCCTATTTGACCACTATACATTGCTAGCATCAGGAAATAGAATAATCGCG

AATTCCGGGTAACTGGCCAAGCTGCTAAAGTAGCTAAAGTAGTGATAAATCCTGTCAATA

AAATAGATCCTAATGAAAGTCCATCGATTCCCAATCTCCAGTGGAAATCAAAGACATCTA

TCCATTTAGAATCCTCCTTTAATTGGATTAAGGGATCCTCCAATTGGAAATGATAACAGA

ATGCATAAGTCATTAGAAGGAATTCTAATAAACAAATAGATATAGTATACCACCTAACGA

TTTTGTTTCCCCTATGAGGTAAAAAGAAAATTAATGAACCTGCAAATATCGGCAAAACAA

CAAGTATTGTTAACCAAGGAAAATAACTCATGATAAAGTGATAAAGAGAAGATACGTTTT

GACCAGAAAAGCCCGTGCTCGAAATAAGCGAGCACAGGCTTCCTCGGTAAAGAGGAATCA

GACGATTCAAGTGGAGTTTTTTGTAACGTATCAATAAGATAGAGCCATGCTGCGGGTTGT

TTCAGGCCCTAAATAAACGCGGACACTTAAAAAATCTGTTGGGCAGGCAGATTCACATCT

CTTACAACCCACACAATCTTCGGTTCTCGGCGCGGAAGCAATTTGCTTGGCTTTACACCC

ATCCCAGGGTATCATTTCTAATACATCTGTTGGACAAGCTCGTACACATTGAGTGCATCC

TATACATGTATCATAAATTTTTACGGAATGTGACATTGGATCTATAAATTTTCCTTTTCA

ACATAAAAATTTTCGATCTGGTAAAAATGAAATTAGTACTATATGAGTCATATGTATTGT

AGACACCAGACGAAGCAATGGTTTATCCAAACTTCAACAAATAATGCAATATATTTCTTA

ATCCGTTTGTGAGAAAGCGTGAAAAGAGCCAAGAGACTTGAATTTTTGGCTTCAACAATC

ATAATTATACGAATTGTACATACGAATTCGAATTAGCCAATTTATTGGCTATCGTCTTTT

CAATATAAATTATTGCAATATTCAAATTGCAATATCAATGAATTGCAAAAATTCAATAAG

TAAAAAGAATACTATGTAATAACCTAATCAAAAAATAGATATTATAAAATAATAAATAGT

ATTATTAATATTTGTTATTATTATATGTGCGCCTTTGTTTAGAGGATTTTATGTCTAATT

ATTCAAAAAATTAGATTGATTGATACGAGTTGATTTCTTGTTACGATGGATGGAAGAAAG

AATGGATAGTCCAATAGCTGCTTCAGCAGCCGCAAGGGCTATAACAAAAATTGCGAAAAT

GTCTCCTTTTAATTGGCGACTATCAAATAGATCAGAAAATGTTACGAGATTTAGATTAAT

TGAATTCAGTATAAGTTCAAGACATATTAGAGCTCTAACCATGTTTCGGCTTGTGATCAA

TCCATAGATACCAATCGAAAATAAATAGACACTAAAAAAAAGTACATGCTCAAACATCAT

TAACTAACTCCTTATCAATCTCGATTCATTTCAATATGAGGACAAGAATTGAACCGATTC

CATTAATTAGAATAGAACAGTTACACAACAAAAGAGAAAAGAAGGTATTTGTTGGCAGTA

GATGGGTTTTACTAAATCAAAATTGTGATTCTTTAGTTATTTATTTTAGATTTGAAATTC

TAAGAATTTGACTAATTCTAAGTATTTCTTATTGCCGAGCCATAGTAATTGCACCTATTA

AAGAAACTAGAAGAATTATGGAAATGAGTTCAAACGGAAGATAAAAATCAGTTGCTAAAT

GAATCCCAATTTGTTGAACGTTATTTATGAGACCCTGTTCTACTATTTGGTTTGATCTTG

TAGTCCAAAGAATTCCATACCATGACGTATCTGGGATAGTAGTCATTAGTGAAAAAAGAA

TAGTTATACAAACGAGTGAAGTGAACCCATCTCCAATAGTCCAATAATTCTTATTTTTAG

ACCATTCTGAGCCATTTACGAACATTACGGCAAATATGATCAAAACATTTATAGCTCCCA

CATAAATAAGAAGTTGTGCGACAGCTACAAAGTAGGAATTCAATAAAATATAGAATAAGG

ATATACAAACAAGAACTAATCCCAGCGAAAAGGCAGAATAAATTGGGTTGGTAAGTAATA

CTACTCCTAGACCTCCTAGTAGAAGAACAAATCCCCCAAATAGCACAAGAATCTCATGTA

TTGGTCCAGGTAAATCCATTATGGATAAGAAGAAATTAATAGTATAAAATTTTTCATGAA

CTGACTAAAACTAAAAGATTCAAGGAAGGAAAAAGGGATTAGGATATTTTTTGTATATAA

GTTGTTAGTTATTCGTTTTTCTTTATAGTTAATAAAAATGGATTCTAAAAGATAAATCCT

AATAATTAGTAACCGTTCTTGAATTCCAAGATTTTTCTTCGTCTATTTTACTTTGAGTCG

AATTCCTAATTGTTTGAATTGTGTAATCTCCCATTATGGAGATTGGTAACCGACTCAAAG

CAATTTGATTGTAATTCAATTCATGACGATCATAAGTAGAAAGTTCATATTCTTCAGTCA

TTGATAAACAGCTTGTCGGACAGTACTCAACACAATTACCACAAAATATACAAACTCCGA

AATCAATACTATAATTAAGCAATTGTTTCCTTTTAATATCCTTTTCAAATCTCCAATCCA

CAAGGGGTAGATCTATCGGGCATACGCGAACACATACTTCACAAGCAATACATTTATCAA

ATTCAAAGTGGATTCGCCCCCGGAAACGCTCCGATGTAATTGATTTTTCATAAGGGTAGT

GAATCGTTATAGGTAAACGATTTGTGTGGGATAAGGTAATTATGAAACTTTGACCAATGT

ACCTTGCAGCGCGTATTGTTTGTTGACCATAACTCATGAACCCAGTTACCATAGGGAACA

TATTCTAAATATCTATGAAAAAGATATGTTTCTTTCTCTTGTTTGAGAGAACTTTTGTGT

TGAAAATATTCTTACTGTTATTGTATTCTTATTTATAGTGAAACAAGTTGGGAAGAAGTT

GTTAATAAGAGATTGCCCAGGGAAATAGGTAAAAGAAATTTCCATCCAAGATTTAATAAC

TGATCCATTCTCATCCTGGGTAAAGTCCATCTTATTGTGATAGAAATGAAGAGAAATAAA

TAAGCTTTAGTTAATGTAATAAAGATACCCATTGTCATTTCCAAAATTCCAACCATTTTA

TTCATTTGGAAAAATTCAAAAAAGGATATATAGGGAATAGAGAAATTCCACCCGCCTAAG

TAGAGAACTGTTACAAATAAAGAGGAAACTAATAAATTTAGGTAAGAAACAAGATAAAAT

AAACCATATTTGATACCGGAATATTCGGTTTGATAACCTGCTACTAATTCTTCCTCTGCT

TCTGGTAAATCAAAGGGTAATCTTTCACATTCTGCCAAAGAAGAAATTAGAAAAACCAGA

AAACCTATAGGCTGACGCCAAAGATTCCATCCAAAAAAACCATATTTTGACTGTGCTTCA

ACTATATCAACTGTACTTGAACTGTTAGATAATCATAGTCGATGATAACATCACAGTTCC

CACCGCTATTCCAAAACCGTACATGAAACCTTAGCTTCATACGGCTTCTCTATGATCAGA

AAAAGGAAAGGGTTGTTTCGTTTCGGTATTATCCCCTGGGCATAGATAGAATTAGGTAAG

ATAAAATCGATTGGAAAGTCCTAAATTAGACCAAAGGAATTCCGTCTGCTAGAATAAGAA

AAAGCGCTTCCGAATTGATCTCGTCCTTTATAATATAAAATTTTTCTTTGTTCAGTAATA

ACTTAATCTTGGAATAAAACACTCGTTATAGCAATTAATAAATGAAAAGAATTAGGCATT

AATTCATGAGGAATTCTGTATTAATATGAATAGAGGAAGGAAAAAATAAATAAATATCTT

TTTTTGTATTGCATTCCATATCTTTTGTCCTATTCTTCTTTCCCCGAAGGGTATAAAAAG

AAAAAAGGAATAAAGGATTAATTCGTTCTTGATAGCCATTTCTTTAACAAGTGAAAGGGA

ACATACTCTGGATCGGAATCCGAAGAAGTACTACTTGATCATTTCCACCAATTTCAAGTC

CTTATTATGATTCCTTTTATGAGGAAAAATCTCTAATGCCCTTTATGTACTTTAGTGTTC

CTAACCCCTCACTAATTTTTGATGGATTCCCTTATGATTATAAGTTTCCATATCGCGAAT

CCTTTATTCTTGCCCGCTTCAAGATATGATGACTAATCAAAAAATCTCAACCTTGGGGTA

AAGAGTTTACACTACTTATGTTTACTTCAATTTTTTCTTGTACGTAGGAAATGAGATTTT

TTCTTTTTACTACAAATTAATAAGTTGTTTTGTTTCACTCATATAGCTATCTAGTTTAAC

TTACCAACCCGAGAATAAGAAAAGGAAGATAAATATTCAATGGATTTTGGAGGAAAAAGA

TCCTATTTTAACGAATCACACGTAGAGATATTGCTAGCACACAAAAAGTTAATGGTATTT

CATAACTAATAGATTGAGCAGCAGCTCGTAGACCGCCTGAAAAAGAATATTTATTATTTG

AGCTATATCCTGCCATAAGAAGACCAATAGGAGCAATACTTGAAATGGCAATCCATAAAA

AAACACCAATACTAAGATCGGCTAAAACAAAACGATATCCCAAAGGGATAACTAAAAAAC

TTAATAAAATTGATATGACTGCTATAGAAGGTCCAATGCTAAATAAAGGAATATCCCCTC

GGGATGGCAGGATATCCTCCTTAAAAAGTAGCTTAGTTCCATCGGCTATAGCTTGAAGCA

GTCCCAGGGGGCCAGCATATTCAGGACCAATACGTTGTTGTATCGATGCGGATATTTCTC

TTTCTAACCACACAATTACGAGTACTTCTATTGTGATTCCCAGTAGGAGGGTCAAAATGG

GTAGAATCCATATCAGTCCATAGACTTCTTTTAATAATTCCGATTTCGAAAAAGAATTGA

TAGTTTCTATCTCTACCCTATCTATTATCATTTCAACGATCAACTTCCCCCATAATGATA

TCTATACTACCTAATATCGTCATGATATCAGCCAATTTCATTTTTTTAACTAGTTGAGGA

AGAATTTGCAAATTAATAAAACCGGGTGGACGAATTTTCCATCTCCAGGGGAAAAGACTA

TCATCTCCTACCAGATAAATTCCTAATTCACCTTTTGGAGCTTCCACTCTTACATAAAGC

TCTTGCTTTGACAATTCAAAATTGGGCGAAGGTTTTTTACCAAGAAATCGATATTCAAAA

TCATTCCATTCGGAATTCTTTGTTTTCTTAAAGCGTCGGACTTCTAAATTCTCATAAGGG

CCTCCAGGAATTTTCTCTACAGCCTGTTGAATAATTTTGATGGATTCCCTCATTTCACCC

ATTCGTACTAAATAGCGAGCTAATGAATCCCCTTCTTTTTGCCATTGGACTTTCCAATCG

AATTGGTTGTAAGACTCATAAGGATCAACTTTACGAAGATCCCATTGTATTCCAGAAGCT

CGTAACATCGGTCCCGATAAGCCCCAATTTACTGCTTCTTCTCCGCTAATAAAACCGACT

CCTTCAACTCGTTCTAAAAAAACGGGATTCCGTGTAATAAGTTGTTGATATTCAACAACT

CCTCGTAAAAAATAATCACAGAAATCTAAACATTTATCGACCCATCCATAAGGTAGATCG

GCGGCTACCCCTCCGATGCGAAAGTAATTATGCATCATTCGCATACCTGTAGCAGCTTCA

AATAGATCATATATCAATTCTCTCTCTCTAAAAATATAGAAAAAAGGGGTCTGTGCGCCT

AGATCCGCCATAAAAGGTCCAAGCCATAACAAGTGAGAAGCTATACGGCTCAACTCTAAC

ATAATTACCCTAATATAGCTGGCTCTTTGGGGTATTTGAATATTCTCCAAGAATTCTGGT

GCATTTACCGTTATTGCTTCTGTAAACATAGTAGC

[3] Fargesia_communis

CCCAATATCTTGCTGGAACAAGATATTGGGTATTTCTGGCTTTCCTTCCTTCAAAAATTG

CTATATGTTAGCAGAAAAGCCTTATCCATTAAGAGATGGAACTTCAAGAACAGCTAGGTC

TAGAGGGAAGTTGTGAGCATTACGTTCGTGCATTACCTCCATACCAAGATTAGCACGGTT

GATGATATCAGCCCAAGTATTAATAACGCGACCTTGGCTATCAACTACGGATTGGTTGAA

ATTGAAACCATTTAGGTTGAAAGCCATAGTACTAATACCTAAAGCAGTGAACCAGATCCC

TACTACAGGCCAAGCAGCCAAGAAGAAGTGTAAAGAACGAGAGTTGTTGAAACTAGCATA

TTGGAAGATTAATCGGCCAAAATAACCATGAGCAGCCACAATATTATAAGTCTCTTCCTC

TTGACCAAATTTGTAACCCTCATTAGCAGATTCATTTTCAGTGGTTTCCCTGATCAAACT

AGAGGTTACCAAGGAACCATGCATAGCACTGAATAGGGAACCGCCGAATACACCAGCTAC

ACCTAACATGTGAAATGGATGCATAAGGATGTTGTGCTCTGCCTGGAATACAATCATAAA

GTTGAAAGTACCAGATATTCCTAAAGGCATACCATCAGAGAAACTTCCTTGACCAATAGG

GTAAATCAAGAAAACAGCAGTAGCAGCTGCAACAGGAGCTGAATATGCAACAGCAATCCA

AGGACGCATACCCAGACGGAAACTAAGTTCCCACTCACGACCCATATAACAAGCTACACC

AAGTAAGAAGTGTAGAACAATTAGCTCATAAGGACCGCCGTTGTATAACCACTCATCAAC

AGATGCAGCTTCCCAAATTGGGTAAAAGTGCAATCCGATCGCCGCAGAAGTAGGAATAAT

GGCACCAGAGATAATATTGTTTCCATAAAGTAAAGAACCAGAAACAGGCTCACGAATACC

ATCAATATCTACTGGAGGAGCAGCGATGAAGGCGATAATAAATACGGAAGTTGCGGTCAA

TAAGGTAGGGATCATCAAAACACCGAACCATCCGATGTAAAGACGATTTTCGGTGCTAGT

TATCCAGTGCAGAAGCGACCCCACAGGCTTGTACTTTCGCGTCTCTCTAAAATTGCAGTC

ATGGTAAGATCTTGGTTTATTCAAATTGCAAGGACTCCCAAGCACACGTATTAACTAAAA

AGATAATAGAAGGCTTGTTATTTAACAGTATAACATAGACTGTATACCAATGTCAACCAA

GCCAGCCCCAACGATTGGATATCCATATAACTAAATTCACCAAACCAAAAATTTTGTAAA

TGAAGTGAGTGAAAATTCAAAACTCAGATTATTTCCATATGGGTTGCCCGGGACTCGAAC

CCGGAACTAGTCGGATGGAGTAGATAATTCTTCCTTGTTACAATAGAAAAAATCCCTCCC

CAAATCGTGCTTGCATTTTTCATTGCACACGACTTTCCCTATGTAGAAATAGTCAATTTC

TATTCCAAAGAGGAAGTCTACCAATTTTTTGAATAGTAAGTTGATTCACCTACTATGAAC

ATTTCAGAATGGAAAATGTGAAAGTTTTATCTTGATATCGATCTTTCTAGTGTATTAGTT

TTGTCTAATGATTAATTAAAAGGGTTCACCAGGTCATTGATACGGATAATATCCAAATAC

CAAATACGGTCACTGTGTGATCCACGGAAAGAAAAGTGGGTTGTTTTGGTGAACATCAAA

GAAAAAACTTGCTCTTCTTCCGTAAAAAATTCTTCTAAAAATACCGAACCCAACCGTTGC

ATAAAAGTTCGTACCGTGCTTTTATGTTTACGAGCTAAAGTTCTAGCGCATGAAAGTCGA

AGTATATACTTTAGTCGATACAAAGTCCGTTTTTTCGAGGATCCACTATGATAATGAAAA

AGATTTCTACATATCCGACCAAATCGATCAAGAATATCCCAATCTGATAAATCGGTCCAA

ATGGGTTTACTAATAGGATGCCCCGATCCAGTACAAAATTGAGCTTTTGATAAGGATCCA

ATGAGGGGAGTAGCAGGGACTATGGTATCGAATTTTTTCATTTGAGTCTCTATTAGAAAT

GAATTCTCTAGCATTTGATTCCTTACTAACAAAGGATTTATTGGTACACTTGAAAGGTAC

CCCAGAAAATCGAAGCAAGAGTTTACTAATTGGTTTAGATGGATCCTTCGCGGTTGAGTC

CAAAAAGAAAAAGAATATTGCCAGAAATTGACAAGGTAACATTTCCATTTCTTCTTCAAA

AGAAGAGTTCCTTTTGATGCAAGAATTGCCTTTCCTTGATATCGAACATAATGCATAAGA

GGATCCATAAAGATCCATACGGTTTTCCGAAAAAAACCTGGGTACATTACCCCAAAATGT

TCCATCTTCCTAGAAAAGTGGATTCGTTCCACAAAGGTTCCAGAAGATGTTAATGGTAAG

CAAGAAGATTGTTTACGAAGAAACAACAAGAAAAATTCATATTCTGATACATAAGAGTTA

TATAGGAATCGAAATAGTCTTTTATTTTCTTTTTTCAAAAGAAAAATCGATTTCATTGAA

GTAATAAGACTATTCCAATTCGAATAGTAGTTGAGAAAGAATCGCAATAAATGCAAAGAT

GGAACATCTTTGATCCGGTATTGAAGGAGTTGAACCAAGATTTCAAAATGGATAGGATAG

GGTATTTCTATATGTGATAGATAATGTAAATGCAAAAATTTGTCTTCTAAAAAGGGAAAT

ATTGAATGAATAGATCGTAAATTCTGAAACTTTGGTGTTTCTTTTTCTTTCGGACAAGAT

AATTCTCGTAGCGAGAATGGGATTTCTACAACGATCGCAAACCCCTCAGATAGAATCTGA

GAATAAAACTCAGAATAAAAAAAATTGTTGTAATCCAACAATCGATCTTGGTTAGGATGA

TTAACCGAGTTAATCCAAAAATTCTGCTGATACATTCGAATAATTAAACGTTTCACAAGT

AGTGAACTAAATTTCTTGTTATTACAACTAACAATTTCCACAGGTTCGGAACCTTTTAAT

CCATAATCATGGGCAAATGCATAAATATACTCCTGAAAGAGAAGTGGGTAAACGAAGTAT

TGTTGACGAGATTTCTGTTTTTCTGAATACCCTTCCAATTTTTCCATTTGTATTTCTACT

TGAATCAGAAAGAAGAAGCATTTCTCGGTTTCTCAAATGATGATACATAGTGCAATATGG

TCAAAACAGGGTGTTGCATAATACAAACCTTTCTATCCAATTAGTTTATGTTTGTTCTAA

TTACAAAAGAGAACAAATCTTTTATTTTTGCAGGCCAATCGCTCTTTTGACTTTGGAATC

CAGTCTCTTTATCAATATACTGCTTCTTTTACACATTCAATCCATAACATCCTTTTCAAT

CTATAATCAAGAATAATTAGGATTTCAAAAAAAAAAGAAAAAGGGTCCGTTCATAGGAAA

ACCAACCTTTCCCCGCATCAGGCACTAATCTATTTTTAACGTCTAATTAGATCGGGGAAT

CATTTCAATTAAGAAGTTAAGCTCGTTGCTTTTTATTTTACCAGAATTGGAGCCAGGCTC

TATCCATTTATTCACTAGACCCAGAAAATAGGAATTTTTTTATTCCAAAAAAAAAAAGAA

ATTGATTTTATTACGACATGCTATTTTTTCCATTCATTACCCTTGAGGATCAGTCGTGGT

CTTCTAGACTCTACCAAGAGTCTGGACGAATTTGTTGCTCATCCAAATGTGTAAAGATCA

TAGTCGCACTTAAAAGCCGAGTACTCTACCATTGAGTAGCACCCAGATAAATAGGATCTT

AGATACGATCGAACCCAAAATCAATGGAATTACACCACATTGAACTAGCAAAACATTAAA

AGAAAGATTTTATCGCCATTAAAAACACTCAAATGCAAAATGAACAGGTCCGGCTAAATT

TCACTAAGGTTAAAAGCGGCCCCAATCACGATAGCAAAATTGTCATTTTTTTAGCATTTA

TATATATAAATAAATCTTGTATGAGAGTACATGCAAGAGGGACAACCTTATCATTTGAGC

GAAGTGTAGACAAAAAACCTAATATGGAGTGAGGATAAAGAGACCTATCTATCTACAAAT

TCTATTTGTTCAATAGACCTTTGTCAATGGAAATACAATGAAATTAGATAGAAAAAGTAA

ATAAAATAAGGGCTTATGTTGGATTGGCACGACATAAATCCAGTCAAAAATAGGACTAAG

AGGCAAATTGTGTCTAAATAATTAAGGGATACTAGTGATCCTCTCCTACTTTTTTATTCA

TTTAGTTCTTCAATTAACTCAAAGTTCCTTCTTTTTCTTTAAAGAATTCTGCCTTCCTTA

AAATATCATAAACAGTTCTTGTAGGTTGAGCACCCTTTTCAAGGAAATAGAGAATAGCTG

GAACATTTAAACAAGTTTGATTCTTTATCGGATCATAAAAACCTACTTTTCGAAGATCTC

TTCCTTCTCTTCGAGATCGAACATCAATTGCAACGATTCGATAGACAGCTTATTGGGATA

GATGTAGCTAAACAATCCCCCCCTAGAAACGTATAGGAGGTTTTCTCCTCATACGGCTCG

AGAATATGACTTGCATTAATTTCCTTACAGAAAAACAAATTTCATTTATACTCATGACTC

AAGTTGGTTAATTTTGACTGACAGACTTAAAAGGAAAAATCCTTCCAAATTTTTTGAGTC

GTCTCTAAACTCTTTTCTTTGTCTCATCTCGAACGAATTGACTTTTATTCCTTATTCTGA

TCCAATTCTATTGTTGAGCAATTGAAAATCGCGTTTACTTGTTCCGGAATTCTTTATCTT

TGATTTGTGAAATCCTTGGGTTTAGACATTACTTCGGGAATTCCTATTCTTTTTTCTTTC

AAAAGAGTAGCAACATACCCTTTTTTCTTATTTCCTTCGATAAAGCATTTCCCTCTTCTA

TAGAAATCGAATATGGGCGATTGATTCTGATAAACTTTTAATTGAAAGAGTTTTTCCAAT

CTTCCAAAATTGGACTTTTTCTTATTTTAACCTTTCGATTTCTATATTAAGGATAGACTG

ACAAAGTTGGCCTAATTTATTAGTTTTCACTAACCCTAGATTCTTTCCCTTGATAAAAAA

TCAATTCTGTCCTCTCGAGCTCCATCGTGTACTATTTACTTACAAACAACCCAGCGCAAA

TTTGGTTCGGGACGAATAGAACAGACTATGTCGAGCCAAGAGCATTTTCATTACTATGGA

AAATGATGGATAACAAAATCCACAATCGATCATGTCCTTCAAGTCGCACGTTGCTTTCTA

CCACATCGTTTTAAACGAAGTTTTACCATAACATTCCTCTAATTTCATTGCAAAGTGGTA

TAGGGAATTGATCCAATATGGATGGGATCATGAATAGTCATTTTTTTGTATACTAATTCA

AACTTGCTATCTATGGAGAAATATGGATAAAAGAAATAAGTATTTATCGGGGAAGACTCC

GCAAAGATCCAATTTATTTAAACCCATATTCTATCATATGAAGGAAACATAGTTCGAAAA

AGACGAATAAACAAGTTTGCTTAAGACTTATTTTTTATTGAATTTCCATCCTCAACAGAG

GACTCGAGATGGTCAATCCTGAAATGAGAAGGATCGACTCTTCTCCAACAAATAAACTAT

CAACCTCAAGTTTAATTAATTTAATTAGCAATATATTTTTCCATAACAAAAACTATTAAC

TAAATAAACTATTCCAATGAAAAGAAAGTTTTTTGGTAGTTATAGAATTCTCGTACTCTT

CGACTCGAATACCAAAAGAGGACTCCAAATCAAAATTGAATCCATTCTATCCAACGAACA

GTTCTTACCTTATCCTTACCAGAATGGATCATCTGGATATTTAAAGAATCGCAGATCGAG

ATGGTTTTCGCTTAACCAAAGAGGGGCCCTTTTTACTAATAATATAATACAACAAAAATC

TATCTCTATCATAAAGGGATAGGTCTCATTTTTTATACAGTGTTTTACGTTTTTTCATGA

AAAAAAGATATTCAATTTGACTGGACTTGACACTTGATTATGTTTTCTGAGAAAGAAAAA

AATGCTTAGAAATGCATCTAATCTAAGAGTTCATAAGAGATAATTATTCTCTTTAATAAA

CTTTTGTCTCGTGTGGGGTACAATATGATTTCATCTTTCGTTTCATCAGAAAAATCTGGG

ACGGAAGGATTCGAACCTCCGAGTAACGGGACCAAAACCCGCTGCCTTACCACTTGGCCA

CGCCCCATTTCTGGTTTTATGCGACACTAATAAACACTATTATGTTTATTTGTTATTCGT

CAATCCCACTTCAATTACATAAAAATGAGGGATACTCTCTTGCTAGGATTCTAGACATGC

GGATAATATAGAATCCAAAAAATGCATTGATCATTACATGGAATTCTATTAAGATATTAT

ATGAAAGTCGAATTTCTTCCATTCTCATTTGAGAGTGCGAATACAAGGAGGTATTTTGCG

TTTGGGAAAGTCCGAAGAAAAAAGGATTTTGAACCCGCCTTTTCTTTTTTCCCTTAGAAA

AATAACTCAATCAAAATCCAATTATCTACTCTACAAGAACGAAATGCTTGTTATGCCTAA

TATACTTAGTTTAACCTGTATCTGTTTTAATTCTGTTCTTTATCCGACTAGTTTTTTCTT

CGCCAAATTGCCCGAAGCTTATGCCATTTTCAACCCAATCGTGGATTTTATGCCTGTCAT

ACCTATACTCTTTTTTCTATTAGCCTTTGTTTGGCAAGCTGCTGTAAGTTTTCGATGAAA

TCTTTACTACTCTGTCTGCCAAATTGAATGATGTATTCATTCCAAAAAAAAAAATGAATA

AAAGCCGAGAAGTCTTATATTATGAACCTTCGATTCTAAAATTCTAATTCTTCTACATTG

AATGTATAGCTGCAGCAATAAATTGGGATCCGCCTTTCTACCCCTGCACCTACGTTGAGC

AGGTACCTTTAGGTACCCACACAATACCTAACCTAATTTTTTGATAAGAGTGCTTATTAT

AAATCAATTCTTGCAATTTTTTTAAGAATTGATTTTTGCATTTTTAGGTGTAAAAATAAA

AAAACCCATCCTAGTGGATCTGTGTGGTAAGGAAAACGGGTAATCTATTCCTTAAAAAAA

ATCTTGGAGATTATGTAATGCTTACTCTCAAACTTTTTGTTTATACAGTAGTGATATTCT

TTGTTTCCCTCTTTATCTTTGGATTCTTATCTAATGACCCAGGACGTAATCCTGGGCGTG

AGGAGTAAAAATCCAAAATTTTTTCTTACAAATTGGATTTGTTTCGTACATTTATCTATG

AGAAAATCCGGGGGTCAGAATTCCTTCCAATTCGAAAGTCCCAAATGATCCGAGGGGGCG

GAAAGAGAGGGATTCGAACCCTCGGTACAAAAAAATTGTACAACGGATTAGCAATCCGCC

GCTTTAGTCCACTCAGCCATCTCTCCCCGTTCCAAATCGAAAGGTTTCCGTGATATGACA

GAGGCAAGAAATAACGATTGCAAAAAATCCTTCCTTTTTCTTTCAAAAGTCCATAAAAAT

TATATTGCCAATTCCATTTTAATTATATTCTTTTTTCTTAATAAAAAAGAAGAAAATTCT

TGTTTTTTCTTTCTAAAATTCGATATTGGCTGAGAAACAATCAGATAGATTTTCTCTTCA

GCGGGCATTTTCATATAGGACTTGTTATAATAAAACAAGCAGGTTATATAAAAAATATTT

ATAAACAAAACAAAAAGGGTTCTTATCAAACCCACCATAAAATTGGAAAGAAAGATAAAG

TAAGTAGACCTGACTCCTTGAATGATGCCTCTATCCACTATTCTGATATATAAATTCGAT

GTAGATGAAATTGTATAAGCGGATTTTTGTATTTCCTTAGACTTAGACCGCGCAAGGCAA

GAATTTTTCGCTATTTACGATTTCATATTCTTGTTACTAGATGTTCTATAGGAATAAGAA

GAAATCGCAACTCCTTTCCGCTACACATAAAAATTGATTTCGAAAGTCAATTTTTTTCAA

TATCTTTCTTTTCCTTAAAAGATAGGCTTTGAAATAGGAGTCATGGAATAATGCTGAATT

CAAATGTTTATTTCTATAGTATAAGAAAAACTAATCGAATCAAATTCATGGATTTACCAC

GACCTCGGTTGTGACCCCATAGATAAAAATAAAAAATTTCTATCTTCGAGACCTTTGAAA

AAGGGCATTGAACGAGAAAAAATCGTCCACAGATAATCTATCGTATGCCTTGGAAGTGAT

ATGAGGTGCTCGGAAATGGTTGAAGTAATTGAATAGGAGGATCACTATGACTATAGCCCT

TGGTAGAGTTATTAAAGAAGAAAATGATCTATTTGATATTATGGACGACTGGTTACGAAG

GGACCGTTTCGTTTTTGTAGGATGGTCCGGCCTATTGCTCTTTCCTTGTGCTTATTTCGC

TTTAGGGGGTTGGTTTACAGGGACAACTTTTGTAACTTCTTGGTATACCCATGGATTGGC

TAGTTCCTATTTGGAAGGTTGTAATTTCTTAACCGCGGCAGTTTCCACCCCTGCCAATAG

TTTAGCACACTCTTTGTTGCTACTATGGGGCCCGGAAGCGCAAGGGGATTTTACTCGTTG

GTGTCAATTAGGCGGTCTGTGGACTTTTGTCGCTCTCCATGGGGCTTTTGCACTAATAGG

TTTCATGTTACGTCAATTTGAACTTGCTCGGTCTGTTCAATTGCGGCCTTATAATGCAAT

TTCATTCTCTGCTCCAATCGCTGTTTTTGTTTCCGTATTCCTTATTTATCCACTGGGGCA

ATCTGGTTGGTTCTTTGCGCCGAGTTTTGGCGTAGCAGCGATATTTCGATTCATCCTCTT

TTTCCAAGGATTTCATAATTGGACATTGAACCCATTTCATATGATGGGAGTTGCCGGAGT

ATTAGGCGCGGCTCTGCTATGCGCTATTCATGGGGCGACCGTAGAAAACACTCTATTCGA

GGACGGTGATGGTGCAAATACTTTCCGCGCTTTTAACCCAACTCAAGCTGAAGAAACTTA

TTCAATGGTCACTGCTAATCGCTTTTGGTCCCAAATCTTTGGTGTTGCTTTTTCTAATAA

ACGTTGGTTACATTTCTTTATGCTATTTGTACCCGTCACCGGTTTATGGATGAGTGCTAT

TGGCGTAGTCGGCCTGGCTCTGAACCTACGTGCCTATGACTTCGTTTCCCAGGAAATCCG

TGCAGCGGAAGATCCTGAATTTGAGACTTTCTACACTAAAAATATTCTTTTAAACGAGGG

TATTCGTGCGTGGATGGCAGCTCAGGATCAGCCTCATGAAAATCTTATATTCCCTGAGGA

GGTTCTACCACGTGGAAACGCTCTTTAATGGAACTTTCGTTTTAGCTGGTCGTGACCAAG

AAACCACCGGCTTTGCTTGGTGGGCTGGGAATGCCAGACTTATCAATTTGTCCGGTAAAC

TACTTGGAGCTCACGTAGCCCATGCCGGATTAATCGTATTCTGGGCCGGAGCAATGAACC

TATTTGAGGTGGCCCATTTCGTACCAGAAAAGCCCATGTATGAACAAGGGTTGATTTTAC

TTCCGCACTTAGCTACTCTAGGTTGGGGAGTAGGGCCGGGGGGAGAAGTTCTAGATACTT

TTCCGTACTTTGTATCTGGAGTACTTCACCTAATTTCCTCCGCAGTCTTAGGCTTCGGCG

GCATTTATCACGCGCTTCTGGGACCCGAGACTCTTGAAGAATCTTTTCCATTCTTTGGTT

ATGTATGGAAAGATAGAAATAAAATGACTACAATTTTGGGTATTCACTTAATTTTGTTAG

GTATAGGTGCTTTTCTTCTAGTACTCAAGGCTCTTTATTTTGGCGGTGTATATGATACCT

GGGCCCCGGGGGGGGGAGATGTAAGAAAAATTACCAATTTGACCCTTAGCCCCAGTGTTA

TATTTGGTTATTTACTAAAATCCCCTTTTGGGGGAGAAGGGTGGATTGTTAGTGTGGATG

ATTTAGAAGATATAATTGGGGGACATGTATGGTTGGGTTTCATTTGTGTATTTGGCGGAA

TTTGGCATATCTTAACCAAACCCTTCGCATGGGCTCGCCGTGCATTTGTATGGTCTGGAG

AAGCTTACTTGTCTTATAGTTTAGGCGCTTTATCTGTCTTTGGTGTTATCGCTTGTTGTT

TTGTCTGGTTCAATAATACGGCTTATCCGAGTGAGTTTTATGGACCCACTGGGCCAGAAG

CTTCTCAAGCTCAAGCATTTACTTTTCTAGTTAGAGACCAGCGTCTTGGAGCTAATGTGG

GATCTGCCCAAGGACCCACAGGTTTAGGTAAATATCTAATGCGTTCCCCAACGGGAGAGG

TTATTTTTGGAGGGGAAACTATGCGTTTTTGGGACCTTCGTGCTCCATGGTTAGAACCTC

TAAGGGGGCCCAACGGTTTGGACTTGAGTAGGTTGAAAAAAGACATACAACCTTGGCAAG

AACGACGTTCGGCAGAATATATGACCCATGCTCCTTTAGGCTCTTTAAATTCCGTGGGTG

GCGTAGCTACCGAGATCAATGCAGTTAATTATGTCTCTCCTAGAAGTTGGTTAGCGACCT

CCCATTTTGTTCTAGGATTCTTCTTTTTTGTGGGCCATTTGTGGCATGCAGGAAGAGCCC

GGGCTGCTGCAGCAGGCTTTGAAAAGGGAATCGATCGTGATTTGGAACCTGTTCTTTACA

TGAACCCTCTTAACTAAGATTTTTTATTTATAGCTGTTCTAGTTTTTTTCTGTTCTGGCT

CGGTTATTCCATCTAGCCGAGCCATTCATTCCTTAAAAACAAATAAAGAAACAAACGTAT

TCAATAAGCAAAAGGAGAGAGAGGGATTCGAACCCTCGATAGTTCCTAAAACTATACCGG

TTTTCAAGACCGGAGCTATCAACCACTCAGCCATCTCTCCACAGCCTAATCCCTATTTTA

TTCCTACAAATAGAACATAGCCATATGAAATGATCTACTAACCCATCTCAGATGCAAGTC

CCCTTTCGATATATCTCTGTATAAGGTGGTAAGTAATAAGTTTTAAAGAGAAGAATCAAT

GGATTCATGATTAAACCCCTCCTACTTCTTGTATTTTATTACAATTTTGGTTAAGTGAGG

GATCAAATATGTAGTCAACTTTATTTGATGGTAGCTTGGAGGATTAGAAATATGACTATT

GCTTTCCAATTAGCTGTTTTTGCATTAATTGCGACTTCCTCAGTGTTAGTAATTAGTGTA

CCCCTTGTATTTGCTTCTCCTGATGGTTGGTCAAATAATAAAAACGTTGTATTTTCCGGT

ACATCATTATGGATTGGACTAGTCTTTCTGGTAGCTATTCTGAATTCTCTCATTTCTTAA

ATTTGTTTAGTATTTAGTAGCCCGATACAAAATATAAAAAGGCCATTTCTTCGAATTGTG

AGACGCATTAAAATGCAATTTGCGTTCCGAATTGATTGACAGACAATTAAAAAAAGAAAA

CTCTAATAGAAAATGAAACGGTCGACCCAGACATAGACGGTCGACCCAGGCGGATATACC

CTATAAAATATATCCCGTAGCGAGCGTAGTTCAATGGTAAAACATCTCCTTGCCAAGGAG

AAGATACGGGTTCGATTCCCGCCGCTCGCCAGCTTAATTTAGTAAGGTACTATGATAAAA

AATTTAGTCTACTTATATTAAATTAATAGGTGTTAGTCTAGTACCGTATCCCTTACTATC

TTACCCTCTTTTGCACCCCACTCAAAAAAAGGGGCTCCGGAGGCGGGAATCGAACTCGCC

AACAGGGCTCCCTAAATTGGGGATTCACCGAGACAAACAACTGGCAAACTCTTTTAAAGG

GGTAGACTGTGCCTTTCTTTCATTTCTTTTTTCTTTTCTTCTTCTTGCTAATGAATAAAA

AGGGTTGGATCCAGCCCTCTACTCTATACAAATAGAATAGTCCTTTTATACAGACTGCTA

AGTGCGGAGACGGGAATCGAACCCGTGACCTCAAGGTTATGAGCCTCGTGAGCTACCAAA

CTGCTCTACTCCGCTCTGGAGGGACGGAAACTGGTGGACGAAAAAGGTTGAATACAGGAC

TCTACCATGTCTAGACAAATAGAATAGTCCTTTTATACAGAATGGAGCGGGTAGCGGGAA

TCGAACCCGCATCGTTAGCTTGGAAGGCTAGGGGTTATAGTCGACGTTGGTTGATTAGTT

TTAACGTCTCTAATTCAAAACCGAACATGAAATTTTGATTTCATTCGGCTCCTTTATGGA

TATTCTCACCACTTAACATCTATGTCAGCTTTTCTATCTGAATGGAACCAAAGCTCTCCG

CTTTCTAGATGATCCCTATAGAGTAGGAGATAGAAATTCTACTAAATCTATCTAATCTAC

TTACTTCGTTCCCTAATTTCATTCAAGAGATCCTGAGGAAAAGAATTAGGTTTCCACCGA

GCTGAAACAATATGCTGATGGTTCTAGTAAACCAAAACTACCGTTTTTTAGCTATTTGGC

TTCCATTTCCTTTTTAACAAAAGAAGATTTAGTTACGATTGGAAATAAACTTTTTTGTAT

CTTCATCCATAGATCCTTTACTCATATTTTAAAAATTGGAATACTTAATCCAATGCAAAA

TTATGCTTCGCGACTCTGTACTCATAATCCAATTTGTATTTTGGATGCAATTTCAATTAG

TTTTTGGGTACAAATCGCGAGAATGTATATTCTTCCTCAATATGCTATTGAGAGGAAAAG

GATTAAATCCTTTATAAGAACTAAAGTTTTCATCGGAATATAAAAAACTTAAGGACGCCT

TAAGTATATCATTTCAAATTCAGTTATTAATAGAACGAATCACACTTTTACCACTAAACT

ATACCCGCTACATGTAGATTATGATACCAACGCTACCCTTTGTCAAGGGTAGCCATTCGA

GAAGGAGGCTAATTCCCCCTTATTGAATCAAAGGAGAAGGTTCATGACAGTGAGCTGTTG

GTACTTCGATCGCGGGCCTTTATTTCTTTCTTTTTTGTTCAATTCTGAACAAAGAAATTG

GGGAAGATGTTTTCTTCCCCCACTTATCATGAAGTCCGAGCCCTAGAGAAAGAGTGAGAT

GCTTTTAAAAATTCATCATAGACTTTCCCTATGGCTTGAGAGAAGCAAGAAATAACTTAA

ACGGAGAAGCGGACAGGACCCGCTGGTTAGTCGATCCTCTCCATTTACCAATTTCTTCTC

CTCTTTTCCACTCAATTCTAGTTTATTAGATTCTTGTTTAAAAGAATCAAAGAAGATGAA

TAGAACTAAGAACACATAAAAAAAGCATAGAGGACCATTACCAAATGTTCCTCCCAAGAA

TCATATTGGGTATCTGTTCCCTTCCTTTTCCCGCTAGGATCGGGAATCTAGAATCCTCCT

TTTTCCTAATCTCGGAAACAGAAAACCCATAGCCAGGAGCAGTATAAATTCGACTGCCCA

CTTTTTACAAGCAAATTGTTGCTAAAACTCCAACATAGTTTGTTCAAAATGCACCAGAAT

CCTTTGAGAATATTCAAGTACCCCCCTTCCAAGGGGTACCTGTTAAAAATAGTTTCAATT

TCTCACCAAAACAGACAAGAAGTATATCACTGAAAATTAATACCCAACCATATGGGTATA

TGAAGAGCGCGAATTCCTTTATACCCTACCCAATTAGAAGAAATAAAACATAAATGGAGA

AAGTTCTCATCATAAGATCAAAAAAAACCTCTACTTTGTGCAAGTGATAAGAGAGAATGA

AATTCTTATTTTTCTTGATTTTTTTGAACCTCGCCATGAATAAACTTCTATATCTCGATA

TATACATATATTATGTACATTATGCAGTAGACTCATAATGAGAAATCAAAGTGGCTAATT

ATTGAATATCATAAAGGGCTTTTTATTTGGTGGTAGAGTAATGCCATGGTAAGACGTAAG

TCATCGGTTCGAATCCGATAAAGTACTTTTCTACTAAATTTATTCATTTCTTTTTTGAAA

ATTTCTCTTTTTTTCTTGAATTTTATGACTTAGTGTGGGATGCATGCATTTTTGGTCTGA

ACGCTAAACGAGCACGGGGTGGAAATTACAAAAAAGAAATTGGACTCTAGATCAATCAAT

ACCTGTACTGAACTAATCTAGAATAATCTATTCTTATTCTATACCCTTTAAATGAATTTC

CCTAAAAAGTAGGGAATGATCCGTGAATTAACCTAACCATCAACTAAAAAAAATCCTATG

AAAGCATAACAGAAAAGTAGGAAAGACTCTTTGCTTGATCTAGTTCTTCGAGTATATGAC

AATTCCAAAAAACTGCTCATACTATCATTATAGTATAATGACGAGCGGTTGTATATGGCC

CTATCGTCTAGTGATGCCCCTATCGTCTAGTGGTTCAGGACATCTCTCTTTCAAGGAGGC

AGCGGGGATTCGACTTCCCCTGGGGGTAGGGAGTATTATGAAAGGAGGTTAATCATAGAT

TCTAAAAAACCCTAGAATAAATTCTTCCTGGGTCGATGCCCGAGCGGTTAATGGGGACGG

ACTGTAAATTCGTTGACGATATGTCTACGCTGGTTCAAATCCAGCTCGGCCCAAAAATCT

AGGGCTTCGTGAATATGAACTAAATCCATTTTTTTCTTCCATAAAATAAAATGTCTGATC

CATAGAAATAAAGGATAAAGCGAAAGGGGGAAATTTCTTTCTAATCCATATCTCTCTCAT

TCCTTTTTTACAAACAAAAGAGTTTTTCTTATTGAAGGTGGATTATCATCCATTTTTAGC

GATAAAAAATCGCGACATACTAGTTATGTCACTCTCACTATACCCACATATGATATGTGG

GTATGTAGTATATGATTCGTCTATTTTTAGAGTACGACAGGCGAATCGAATCTTCTATTT

AGGTATGCCATACACCCCGCGGGGATTGTAGTTCAATTGGTCAGAGCACCGCCCTGTCAA

GGCGGAAGCTGCGGGTTCGAGCCCCGTCAGTCCCGAACTAGGGTTCAATGAATGGAGAAA

TTCATCTTTCCTTTTTCCATGAAAAAGGGGGGGCAGGAGAGAAGATCAAATACCTATGGG

GCACCCTTATTTCACTTTTTTATTTCGCATTTCTCATTAAGAGGGAGGGGTATAGGATTT

TTTTTCACTACTCCCGGTTGATAAGGAAAGACATACATATCATACTTGGAGGATCTTCCT

ATGTTATACTATTCCACTCTCAACCATGAATTGATTTGATAGATCCGATATTCATAATAT

TGAATTGATTCAGTATTATCAGAATGCAAGTCCTCCCCTTGAATTTACAGGATACCCTTT

TTCCTCTCCATGGGATTACATCCCGAGTTATTGTGAAAAAAAAAGAGGTTATGGAAGTCA

ATATTCTCGCATTTATTGCTACTGCACTGTTTATTCTAGTTCCTACTGCCTTTTTACTTA

TTATTTATGTAAAAACAGTCAGCCAAAATGATTAATTGGAATTCCAATTAATCATTGAAG

AAATGAAAAAGGGATTAAATAAAATAAAAATCCAAGTCTTAAATGAAAGGATCTGGTTGG

AATCATAAAGTGTGGTAGAAAGAACTACATATAGTTTTTTCTACGACACTTTAGAGTCTT

TCTATTATATTATCTTGAATCTACATAGAATAGATTAGTAGATTGAAATAGTAGTCTAAT

TCAATTTCTTTTTTCACTGCATCCACTTAATTTCAATCAAGTCAAAATGAAAGAATCCAT

GGAGGGAGAGAAAAATAATATGAGAATAGACTATAGAAAAGAAAAAAGTAAAAGAAAAAA

CCAGCGAATCTTTCATGCTTAAACATGCGGCGAGATGCTTCAAAAGAGCATAAAAATTAT

TTAAGAATAAGAAAAGAATATAAATGGAAAGTGTGCGATATGTTGTGAATAGCTCCGTGG

AAGAAAGTCTAATTTTCTTATGTATAGAACTTTTTTAACCATTCGTCACTTCTAGTAGAA

ATTTTGAATTGCTGTAATCGCTCTTTCTATTTCTATATAGTAGAATAGAACGACTCTTTC

TTACAAGAGTTTCTTACAGGAGTGAAACAAAACTAAAGAAAGAAAGAATAAAGTTTGGCA

AAATGATTAATGCAAAACGATCAATTAAAGAAAAAAGTTGATACAACAATTCGACTACTC

AATCAATTAGTAGTATCCCTAGAGTCCACTCCTCCCCCATACTACTAGTGAAAGAGAAAA

TGTAAAGACTACCATTAAAGCAGCCCAAGCGAGACTTACTATATCCATGTAAATTATGTC

TCCTATTTCTATGAAGGAATTATTCTACTATTGATCAATAATCATAGTGGAATCAAGGGT

ACAGAGTCAAAAAGGGATTCTGCCCTAACGCTATGGATGAATCAGTTCAAGGAATTTACT

CCTAACAAATTCTTATAGGATTTCTGGTAGAATTGGAGAGCATTAAGTATAAATACGATA

CATAGCCCTTTCTATTAATAAAAGAATAAGGAAACGCAACCTCATCCTTATTGGTAGCCG

TTTGGGCCACTACCGACAAAACAAACCCTAATAGAACTATGGATTCTCAAAATCCAGTAT

CGCCAGGCCTAGTTACTCTCTTGCCCCAACTTAGCAGGGTACGAATTTGTTGAGTTCGAT

CAGTACTATAAGCCTAAGTATTTTATTGATCAGGCGGCACCCAGATTTGAACTGGGGATA

AAGGATTTGCAGTCCCCTGCCTTACCGCTTGGCCATGCCGCCAAAAAATCCGATCTAAAA

TAGAGAAAAGAGCAAGTATTCATCCAGGTTTCTTACTAAAACCTCCTTTCTTTTATCTTG

AATCTAATTCTACTTACTTTTTTCCAATCTTTTTCAAAAAAATTCCTGCTTTTTTGAATC

CAGTTTCGATTATTCTCCTCGATGGATTCTATCTTAAAACAAACATTGCTAACACTAGAA

AACTTCCCTTTTCTTTCTATTGAGATGAAAAAAGAGAAAAGTGGATTTCCAGTCACAGGC

TGCAAAATTCAGAACAAATTGGAACCATTAACTAGAATTCTATTTTTTGAATTTCGGTAT

TCTCCCCCCTTCCTTTTAATGGCATAATAAAATAGAATGAATTTATGCCTAATCCGTGTA

TAGGTAAACTACAGGTCCGAACAGCATTATTATCCATGGATCCCCCTTATGTACATATCT

CTATGGGGAATCGTGCTTTAATTTTTCATTGCATTAAATATCTTGAATAAAAAAAAGAAA

TTTGGTCTGATATGGAGAGGTGGATAACTAGATTGGCATGTACTTAAAAAAGGACTTACT

TTATTTTAGGATTCTACAATGAAATCCTATATTTTCTAGCAATTCTACTACTACGAAACA

AAAAAGAACCCTCAAATTCTTTTTAAAGGAGATAAAATGAGAAATCTTTGCCATCCAATC

TGATTATATCATTAAGTGGCAGAATTTTTTTCTAGGAATGTTTTATCAATTCATTTTCAT

TCGATTTGTACCCCTGGCAAATTCGAACTTTCGTCGAAATTGTCTCTATTCATATGTATG

AAATACATATATGAAATATGTATGTGGAGTTCCCTAGAATTTCATGTGATTCAGTAAACA

GAATATGGATTCCATAATTGCTAGATCGATCCATAGGGATTGATGAAGAGTGAGCTGATA

ATGGAATTTTTCTTCGATAAACAGGAAACTTAAGATGCTCCGGAATGGAAATGAGGGAAT

GTCCACAATACCCGGATTTAGTCAGATCCAATTCGAGGGATTTTGTAGGTTCATTAATCA

AGGCTTGGCAGAAGAACTTGAGAAGTTTCCAACAATTAAAGATCCAGATCACGAAATTGC

ATTTCAATTATTTGCGAAAGGATATCAATTGCTAGAACCCTCGATAAAAGAAAGGGATGC

TGTGTATGAATCACTCACCTATTCTTCCGAATTATATGTATCTGCGAGATTAATTTTTGG

TTTCGATGTGCAAAAGCAAACCATTTCTATTGGAAACATTCCTATAATGAATTCCTTAGG

AACCTTTATAATAAATGGAATATACCGAATTGTGATCAATCAAATATTGCTAAGTCCTGG

TATTTACTACCGCTCGGAATTAGACCATAAGGGAATTTCTATCTACACTGGGACTATAAT

ATCAGATTGGGGAGGAAGATCGGAATTAGCAATTGATAAAAAAGAAAGGATATGGGCTCG

CGTGAGTAGAAAACAAAAGATATCTATTCTAGTTCTATCATCAGCTATGGGTTCGAATCT

AAAAGAAATTCTAGATAATGTTTCCTACCCTGAAATTTTCTTATCTTTCCCGAATGCTAA

GGAGAAGAAGAGGATTGAGTCAAAAGAAAAAGCTATTTTGGAGTTTTATCAACAATTTGC

TTGTGTAGGTGGGGACCTGGTATTTTCGGAGTCCTTATGTGAGGAATTACAAAAGAAATT

TTTTCAACAAAAATGTGAATTAGGAAGGATTGGTCGACGAAATATGAATCGGAGACTGAA

TCTTGATATACCTCAGAACAATACATTCTTGTTACCACGAGATGTATTGGCCGCTACGGA

TCATTTGATTGGAATGAAATTTGGAACGGGTATACTTGACGATGACGATATGAATCACTT

GAAAAATAAACGTATTCGTTCGGTTGCGGATCTGTTACAAGATCAATTCGGACTGGCTCT

TGGTCGTTTACAACATGCGGTTCAAAAAACTATCCGTAGAGTATTCATACGTCAATCGAA

ACCGACTCCACAAACTTTGGTAACTCCAACTTCAACTTCGATTTTATTAATAACTACTTA

TGAGACCTTTTTTGGCACATACCCCTTATCTCAAGTTTTTGATCAAACCAATCCATTGAC

ACAAACTGTTCATGGGCGAAAAGTGAGTTGTTTGGGTCCTGGAGGATTGACGGGGAGAAC

TGCAAGTTTTCGGAGCCGAGATATTCATCCGAGTCACTATGGGCGTATTTGTCCAATTGA

CACGTCCGAAGGAATCAATGTTGGACTTACTGGATCCTTAGCTATTCATGCGAGAATTGA

CCACTGGTGGGGGTCCATAGAGAGTCCCTTTTATGAAATATCTGAGAAAGCAAAAGAAAA

AAAAGAGAGACAGGTGGTTTATTTATCACCAAATAGAGATGAATATTATATGATAGCAGC

AGGAAATTCTTTGTCCTTGAATCAGGGTATTCAGGAAGAACAGGTTGTTCCAGCTAGATA

CCGTCAAGAATTCCTGACTATTGCATGGGAACAGATTCATGTTAGAAGTATTTTTCCTTT

CCAATATTTTTCTATTGGAGGTTCTCTCATTCCTTTTATTGAGCATAATGATGCGAATCG

GGCTTTAATGAGTTCTAATATGCAGCGCCAAGCAGTTCCGCTTTCTCGGTCCGAGAAGTG

CATTGTTGGAACTGGATTGGAACGCCAAACAGCTCTAGATTCGAGGGTTTCCGTTATAGC

CGAACGCGAGGGAAAGATCATTTCTACTGATAGTCACAAGATCCTTTTATCAAGTAGTGG

GAAGACTATAAGTATTCCTTTAGTTACCCATCGGCGCTCTAACAAAAATACTTGTATGCA

CCAAAAACCTCGGGTTCTGCGGGGTAAATCCATTAAAAAAGGACAAATTTTAGCGGAGGG

AGCTGCTACAGTTGGTGGGGAACTTGCTTTAGGAAAAAACGTATTAGTAGCTTATATGCC

ATGGGAAGGTTACAATTTTGAAGACGCAGTACTAATTAGCGAACGTTTGGTATATGAGGA

TATTTATACTTCTTTTCACATCCGAAAATATGAAATTCAGACGGATACGACAAGCCAAGG

CTCCGCTGAAAAAATTACTAAAGAAATACCACATCTAGAAGAACATTTACTCCGCAATTT

GGACAGAAATGGAGTTGTTAGGTTGGGATCCTGGGTAGAAACTGGCGATATTTTAGTAGG

TAAATTAACGCCTCAGATAGCGAGCGAATCGTCGTATATCGCGGAAGCTGGGTTATTACG

GGCCATATTTGGCCTTGAGGTATCCACTTCAAAAGAAACTTCTCTCAAACTACCTATAGG

TGGAAGAGGGCGCGTTATCGATGTGAAATGGATCCAGAGGGACCCCCTAGACATAATGGT

TCGTGTATATATTTTACAGAAACGCGAAATCAAAGTTGGGGATAAAGTAGCCGGAAGACA

CGGGAATAAGGGGATCATTTCCAAAATTTTGCCCAGGCAAGATATGCCCTATTTGCAAGA

TGGAACACCTGTTGATATGGTCTTCAATCCCTTAGGAGTACCCTCACGAATGAATGTGGG

ACAAATATTTGAAAGCTCGCTCGGATTAGCCGGGGATCTGCTAAAGAAACATTATAGAAT

AGCACCCTTTGATGAGAGATATGAGCAAGAGGCTTCAAGAAAACTTGTGTTTTCAGAATT

ATATGAAGCCAGTAAACAAACAAAAAATCCATGGGTATTTGAACCCGAGTACCCGGGAAA

AAGCAGAATATTTGATGGAAGAACAGGAGACCCCTTCGAACAACCTGTTCTAATAGGGAA

GTCCTATATCTTAAAATTAATTCATCAAGTTGATGAGAAAATCCATGGACGTTCTACTGG

GCCCTACTCACTTGTTACACAACAACCCGTTAGAGGAAGAGCCAAGCAAGGGGGACAACG

AGTAGGAGAAATGGAAGTTTGGGCTTTAGAAGGATTTGGTGTTGCTCATATTTTACAAGA

GATACTTACTTATAAATCTGATCATCTTATAGCTCGCCAAGAAATACTTAATGCTACGAT

CTGGGGAAAAAGAGTACCTAATCACGAGTATCCTCCAGAATCTTTTCGAGTGCTCGTTCG

AGAACTACGATCTTTGGCTCTAGAACTGAATCATTTCCTTGTATCTGAGAAGAACTTCCA

GGTTAATAGGGAGGAAGTTTGATCGGAATAAATATAAATTCTTTTCTTATTTATGATTGA

CCAATATAAACATCAACAACTTCAAATTGGACTCGTTTCCCCTCAACAAATAAAGGCTTG

GGCTAACAAAAACCTACCTAATGGGGAAGTCGTTGGCGAAGTCACAAGGCCCTCTACTTT

TCATTATAAAACCGATAAACCAGAAAAAGATGGATTGTTTTGCGAAAGAATCTTTGGACC

CATAAAAAGCGGAATTTGTGCTTGTGGAAATTCTCGAGCGAGCGGAGCTGAAAACGAAGA

GGAAAGATTTTGCCAAAAATGCGGGGTAGAATTTGTTGATTCTCGGATACGAAGATATCA

AATGGGATACATCAAACTCGCATGTCCCGCGACTCATGTGTGGTATTTGAAAGGTCTTCC

TAGTTATATCGCGAACCTTTTAGATAAACCCCTTAAGAAATTGGAGGGCCTAGTATACGG

CGATTTCTCTTTTGCTAGGCCCAGCGCTAAAAAACCTACTTTCTTACGATTACGAGGTTT

ATTCGAAGATGAAATTGCATCCTGTAACCACAGCATTTCTCCCTTTTTTTCTACCCCAGG

CTTTGCAACATTTCGAAATCGGGAAATTGCGACAGGAGCAGGTGCTATTAGAGAACAATT

AGCAGATTTGGATTTGCGAATTATTATAGAGAATTCCTTGGTCGAATGGAAGGAATTAGA

AGACGAGGGGTATAGTGGAGATGAATGGGAAGATAGAAAAAGACGAATAAGAAAAGTTTT

TTTGATTAGACGCATGCAATTGGCGAAACATTTTATTCAAACAAATGTAGAACCAGAATG

GATGGTTTTGTGCTTATTACCAGTTCTTCCTCCCGAATTGAGACCCATTGTTTATAGGTC

TGGGGATAAAGTAGTGACTTCGGATATTAATGAACTTTATAAGAGAGTTATTCGTCGGAA

CAACAACCTTGCCTATCTATTAAAAAGAAGTGAATTAGCGCCAGCAGATTTAGTAATGTG

CCAGGAAAAATTGGTACAAGAAGCCGTGGATACACTTCTTGATAGTGGGTCCCGCGGGCA

ACCAACGAGGGATGGTCACAATAAAGTATACAAATCACTTTCAGATGTAATTGAAGGTAA

AGAGGGAAGGTTTCGCGAGACTCTGCTTGGAAAACGGGTCGATTACTCGGGGCGTTCTGT

CATTGTTGTGGGTCCTTCGCTTTCATTACATCAATGTGGATTACCTCTAGAGATAGCAAT

AAAGCTTTTTCAGCTATTTGTAATTCGCGATTTAATCACGAAACGCGCTACTTCTAATGT

CAGGATTGCTAAAAGGAAAATTTGGGAAAAGGAACCCATTGTATGGGAAATACTTCAAGA

AGTTATGCGGGGACATCCTGTACTGTTGAATAGAGCACCTACCCTGCATAGATTAGGCAT

ACAGGCCTTCCAACCTACTTTAGTGGAGGGGCGTACTATTTGTTTACACCCATTAGTGTG

TAAGGGTTTCAATGCGGACTTTGATGGGGATCAAATGGCTGTTCATCTACCTTTATCCTT

GGAAGCTCAGGCGGAAGCCCGTTTACTTATGTTTTCTCATATGAATCTCCTATCTCCCGC

TATTGGGGATCCTATTTGCGTACCGACCCAAGACATGCTTATCGGACTTTATGTATTAAC

GATTGGAAACCGTCGGGGTATTTGTGCAAATAGATATAATAGTTGCGCAAACTATCCAAA

TCAAAAAGTAAATTACAATAATTATAAGTATACAAAAGATAAAGAACCCCATTTTTCTAA

TTCCTATGATGCACTGGGAGCTTATAGACAGAAACGAATCAGTTTAGACAGTCCCTTGTG

GCTCCGATGGAAACTAGATCAACGCGTCATTGGGTCAAGAGAAGTTCCGATTGAAGTTCA

ATATGAATCTTTGGGGACTTATCATGAGATTTATGCCCACTATCTAATAGTGGGAAATAG

AAAAAAAGAAATCCGTTCTATATACATTCGAAGCACTCTTGGTCATATTTCTTTTTATAG

AGAAATAGAGGAAGCCATACAAGGATTTAGTCAGGCCTATTCATACACTATCTAAACAAG

GAAGTTAGATTCGGCGATGCCTTTCGGGGGGCATTCCGATTTCGCTAGTATCATCATTTT

TGCCGCGCGAATCCAGATTGAGATTAAGGAAAGGAAGTTAATTAAATTTTGAATCACTGA

CTCAGGCCCATTGTCGAATCCTACTCAGCAATTGTCGAATCCTACTCAGCCGAAAAAGGG

GGTACTTATGTATGGCGGAACGGGCCAATCTGGTCTTTCATAATAAAGAGATAGACGGAA

CTGCTATGAAACGACTTATTAGCAGATTAATAGATCATTTCGGAATGGGATATACATCCC

ATATACTGGATCAAATAAAGACTCTGGGCTTTCATCAAGCCACTACTACATCGATTTCAT

TAGGAATCGAGGATCTTTTAACAATACCATCTAAGGGATGGTTAGTGCAAGACGCGGAAC

AACAGAGTTTTCTTTTGGAGAAACACTATTATTATGGGGCTGTACACGCGGTAGAAAAAT

TACGCCAATCCGTTGAGATATGGTATGCTACAAGTGAATATTTGAAACAAGAAATGAATT

CGAATTTTCGGATAACGGATCCTTCTAATCCAGTCTATCTAATGTCTTTTTCAGGAGCCA

GAGGAAATGCATCTCAGGTACACCAATTAGTAGGTATGAGAGGATTAATGGCGGATCCTC

AAGGACAAATGATTGATTTACCTATTCAAAGCAATTTACGCGAGGGACTTTCTTTGACAG

AATATATAATTTCCTGCTACGGAGCCCGCAAAGGGGTTGTAGATACTGCTGTACGAACAG

CGGATGCTGGATATCTTACACGTAGACTTGTTGAAGTAGTTCAACATATTATTGTGCGTA

GAAGAGATTGTGGTACTATCCAAGGTATTTCTTTGAGTCCTCAAAATGGGATGACGGAAA

AACTTTTTGTCCAAACACTAATTGGTCGTGTATTAGCAGACGATATATATATTGGTTCAC

GATGCATTGCCGCTCGAAATCAAGATATTGGAATTGGATTAGTCAATCGATTCATAACTG

CCTTTCGAGCACAACCATTTCGAGCACAACCAATATATATTAGAACCCCCTTTACTTGCC

GGAGCACATCTTGGATCTGTCAATTATGTTATGGTCGGAGTCCCACTCATGGCGATCTGG

TCGAATTGGGGGAAGCCGTAGGTATTATTGCAGGTCAATCAATTGGGGAGCCAGGGACTC

AACTAACATTAAGAACTTTTCATACTGGCGGAGTATTCACAGGGGGTACTGCCGACCTTG

TACGATCCCCTTCGAATGGAAAAATCCAATTCAATGAAGATTTGGTTCACCCCACACGTA

CCCGTCATGGGCAGCCTGCTTTTCTATGTTATATAGACTTGCATGTAACTATTCAGAGTC

AGGATATTCTATATAGTGTGAATATTCCCTCAAAAAGCTTGATTCTAGTGCAAAATGATC

AGTATGTAGAATCCGAACAAGTAATTGCGGAGATTCGTGCCGGAACGTCCACTTTGCATT

TTAAAGAAAAAGTACAAAAGCATATTTATTCCGAATCAGACGGGGAAATGCACTGGAGTA

CTGATGTTTACCATGCGCCCGAATATCAATATGGTAATCTTCGTCGATTACCAAAAACAA

GCCATTTATGGATATTGTCAGTAAGTATGTGCAGATCCAGTATAGCGTCTTTTTCGCTCC

ACAAGGATCAAGATCAAATGAATACTTATTCTTTTTCTGTTGACGGAAGATATCTCTTTG

ACCTCTCAATGGCTAATGATCAAGTAAGACATAGACTGTTGGATACTTTTGGTAAAAAAG

ATAGGGAAATTCTTGATTATTCAACGCCGGATCGAATCATGTCCAATGGCCATTGGAATT

TTGTCTATCCTTCTATTCTTCAAGATAATTCGGATTTGTTGGCGAAAAAGCGAAGAAATG

GGTTCGTCATTCCATTACAATATCATCAAGAACAAGAGAAAGAACTAATATCCTGTTTGG

GGATTTCGATTGAAATACCCTTTATGGGTGTTTTACGTAGAAATACTATTTTTGCTTATT

TTGACGATCCACGATACAGAAAAGATAAAAAGGGTTCAGGAATTGTTAAATTTAGATATA

GGACCCTAGAGGACGAATATAGGACTCGAGAGGAAGACTCAGAGGACGAATATGGGACAG

AGAACGAATATAGGACCCGAGAGGAAGAGGACGAATATGAAACCCTAGAAGATGAATATG

GGATCCTAGAGGACGAATATGAAGCCCTAGAAGACGAATATAGGACTGGAGAGAAAGACT

CAGAAGACGAATATGGGAGCCCAGAGAACGAATATAGAACCCGAGAGGACGAATATGGAA

CTCTAGAGGAAGACTCAGAGGACGAATATGGGACTTTAGAGGAAGACTCAGAAGAAGACT

CAGAGGACGAATACGGGAGCCCAGAGGAAGATTCCATCTTAAAAAAAGAGGGTTTGATTG

AGCATCGAGGAACAAAAGAATTTAGTCTAAAATACCAAAAAGAACTAGATCGGTTTTTTT

TCATTCTTCAAGAACTGCATATCTTGCCGAGATCCTCATCCCTAAAGGTACTTGATAATA

GTATCATTGGAGTGGATACACAACTCACAAAAAATACAAGAAGTCGACTAGGTGGATTGG

TCCGAGTGAAGAGAAAAAAAAGCCATACGGAACTCAAAATCTTTTCCGGAGATATGCATT

TTCCTGAAGAGGCGGATAAGATATTAGGTGGTAGTTTGATACCACCAGAAAGAGAAAAGA

AAGATTCTAAGGAATCAAAAAAAAGGAAAAATTGGGTCTATGTTCAACGGAAAAAAATTC

TCAAGAGCAAGGAAAAGTATTTTGTTTCGGTTCGACCTGCAGTCGCATATGAAATGGACG

AAGGGAGAAATTTAGCAACACTTTTCCCGCAAGATCTCTTGCAAGAAGAGGATAATTTCC

AACTTCGACTTGTCAATTTTATTTCTCATGAAAATAGCAAGTTAACTCAAAGAATTTATC

ATACGAATAGTCAATTTGTTCGAACTTGCTTAGTAGTGAATTGGGAACAAGAAGAAAAAG

AGGAGGCTCGTGCTTCCCTTGTTGAGGTAAGAGCAAATGATCTGATTCGCGATTTCCTAA

GAATTGAGTTAGTCAAGTCCACTATTTCGTATACACGAAGAAGGTATGATAGGACAAGTG

CAGGACCGATTCCCAATAATAGGTTAGATCGCACCAATTCCTTTTATTCCAAGGCGAAGA

TTCAATCACTTAGCCAACATCAAGAAGCTATTGGCACCTTGTTGAATCGAAATAAAGAAT

ACCAATCTTTGATGATTTTGTCGGCATCCAACTGTTCTCGAATTGGTTTATTCAAGAATT

CGAAATATCCCAATGCGGTAAAAGAATCGAATCCTAGAATTCCTATTCGAGATATTTTTG

GGCCCTTAGCCGCTATTGTACCTAGTATATCGAATTTTTCTTCATCTTACTATTTACTAA

CGCATAATCAGATCCTGTTAAAAAAATATTTGTTCCTTGACAATTTGAAACAAACCCTCC

AAGTACTTCAAGGGCTTAAATACTCTTTAATAGATGAAAATCAAAGGATTTCGAATTTCG

ATAGTAACATCATGTTGGATCCATTCCATTTGAATTGGCACTTTCTCCATCATGATTCTT

GGGAGGAGACATCGGCAATAATTCACCTTGGACAATTTATTTGCGAAAATGTATGTCTAT

TTAAATCGCACATAAAAAAATCTGGTCAAATTTTCATTGTTAATATTGATTCCTTTGTTA

TAAGAGCAGCTAAGCCTTATTTGGCCACTACAGGAGCAACTGTTCATGGTCATTATGGAG

AAATCCTTTACAAAGGAGATAGGTTAGTTACGTTTATATATGAAAAATCGAGATCTAGTG

ACATAACGCAAGGTCTTCCAAAAGTAGAACAAATCTTTGAAGCGCGTTCAATTGATTCAC

TATCGCCGAATCTCGAAAGGAGAATTGAGGATTGGAATGAGCGTATACCAAGAATTCTTG

GGGTCCCCTGGGGATTCTTGATTGGAGCTGAGCTAACCATAGCCCAAAGTCGTATCTCTT

TGGTTAATAAGATCCAAAAGGTTTATCGATCCCAAGGGGTACAGATCCATAATAGACATA

TAGAGATTATTATACGCCAAGTAACATCAAAAGTGCGGGTTTCCGAAGATGGAATGTCTA

ATGTTTTTTCACCTGGGGAATTAATTGGACTATTACGAGCAGAACGAGCAGGACGAGCTT

TGGATGAATCGGTCTATTATCGGGCAATCTTATTGGGAATAACAAGGGCTTCCCTGAATA

CCCAAAGTTTCATATCTGAAGCAAGTTTTCAAGAAACTGCTCGAGTTTTAGCAAAAGCTG

CCCTACGAGGTCGTATTGATTGGTTGAAAGGCCTGAAAGAAAACGTAGTTCTGGGGGGGA

TTATACCTGTTGGTACCGGATTCCAAAAATTTGTGCATCATTCCCCACAAGACAAGAACC

TTTATTTCGAAATTCAAAAAAAAAATCTATTCGCGTCGGAAATGAGAGATATTTTGTTTC

TCCATACAGAATTAGTTTCTTCTGATTCTGATGTAACAAACAATTTCTATGAGACATCAG

AACCCCATTTATACGATTTAAGGATACATAAAGCAGATTTTTTTATTTAAACTAGACTTT

TGACCTTAGAACACTAACAGGTCAGATTTTGATTTTTATTAATAAGTAAAGAAGTCAGTT

AATTCATTAAGGTTACGTTTATACCATGTAGAAGGTTACATCGGAACAATTATTATTTAT

TTCAAGCTATTTCGGCTCTTTCTTAATTTTCAAAAAGAAATAAATTCCGTAATGGAAAAA

AAAGAAAAAATCAAAAGGAAGTGTGGAAAAAATGACAAGAAGATATTGGAACATCAATTT

GAAAGAGATGATAGAAGCGGGAGTTCATTTTGGTCATGGTATTAAGAAATGGAATCCTAA

AATGGCCCCTTACATCTCGGCAAAGCGTAAAGGTACTCATATTACAAATCTCGCTAGAAC

GGCTCGTTTTTTATCAGAAGCTTGTGATTTAGTTTTTGATGCAGCAAGTCAGGGAAAAAG

CTTTTTAATTGTTGGTACCAAAAAAAGAGCAGCGGATTTAGTAGCATCAGCTGCAATAAG

GGCTCGTTGTCATTATGTTAATAAAAAGTGGTTCAGTGGTATGTTAACGAATTGGTCGAT

TACGAAAACTAGACTTTCTCAATTTAGAGACTTAAGAGCAGAAGAAAAGATGGGAAAATT

CCAGCATCTCCCAAAAAGAGATGTGGCAATCTTGAAGAGAAAATTATCTACCTTGCAAAG

ATATCTCGGCGGGATCAAATATATGACGAGGTTGCCGGACATTGTGATCGTCCTTGATCA

GCAAAAAGAGTATATAGCTCTTCGGGAATGTGCCATTTTGGGGATTCCTACTATTTCTTT

AGTCGATACAAATTGTGACCCAGATCTCGCAAATATATCGATTCCAGCCAACGATGACAC

TATGACTTCAATTCGATTGATTCTTAACAAATTAGTATTTGCAATTTGTGAGGGCCGTTC

TCTCTATATAAGAAATCGTTGATTAAGAAGAATAGTTCATTCTTGGGTAACTGCGTAGAT

TTATGGGATCACTTACTATTCTTTTTTGTTTTGCATAGATAAAAGAAGGGGAATATTGAT

ATATATTAGAGGGTATTGATATATATTATCATCTGATGTGATTTCTTGGTATCCTAAATA

TAAGATTAATACTTCAAGTTGCTGAGTTGAGAAAGAGATGGTTGAATCAAAAGAATTCCT

TTTTTGAAGTTCAATTTTTATCAGAGGACAATATGAATATTATACCATGTTCCGTTAAAA

CACTCAAGGGGTTATATGATATATCGGGTGTAGAAGTAGGCCAACACTTCTATTGGCAAA

TAGGAGGTTTCCAAATTCATGCCCAAGTACTCATCACTTCTTGGGTCGTAATTACTATCT

TGCTAGGTTCAGTTATCATAGCTGTTCGGAATCCACAAACCATCCCGACCGACGGTCAGA

ATTTCTTCGAATATGTGCTTGAGTTTATTCGAGACTTGAGCAAAACTCAGATTGGAGAAG

AATATGGTCCCTGGGTTCCCTTTATTGGAACTATGTTCCTTTTTATTTTTGTTTCGAATT

GGTCGGGTGCTCTTTTACCTTGGAAAATTATACAGTTACCCCATGGGGAATTAGCAGCAC

CCACGAATGATATAAATACTACTGTTGCTTTAGCTTTACTCACATCAGCGGCATATTTTT

ATGCGGGTCTTAGCAAAAAAGGATTGAGTTATTTCGAGAAATATATTAAACCAACTCCAA

TTCTTTTACCAATTAACATCCTAGAAGATTTCACAAAACCATTATCGCTTAGTTTTCGAC

TTTTCGGGAATATATTGGCGGATGAATTAGTCGTTGTTGTTCTTGTTTCTTTAGTCCCCT

TAGTAGTCCCTATACCGGTCATGTTTCTTGGATTATTTACAAGCGGTATTCAAGCTCTTA

TTTTTGCAACGTTAGCCGCAGCCTATATAGGTGAATCCATGGAAGGTCATCATTGAATTG

ACTAGTTTTCAAAATAGTCTTTTTTTAGCTTAGCTCAATTCATGCATGGTTCCAGATAAT

CCGCTTGGTTGGAAAACTAAATAGTTAGAAATGCGTATGAATATACAACCTAGAGTTGTA

GGAGAGAGAATAGACTATATTACGTGTCAAAGTATATATGCATTAAGGGGGGCGGAGTCA

GGCTAGATCTATATCCTTAATGTCTATAAGCCAGTCATCTTTTGTGCGGGTTTTTAAGGA

ATGATTTTAGAATCCGATTCAATAGAAAATGAGAAAATACGCAAAATAGAAGAAACAAAT

GTATATGGGATATTATATATTCCTAAGTTAGATTCATTATCTAATCCGATATATGGAATT

GGATTCCATATCCAGTTCTATGCAGCATATTGTTATCAATTGTATATCTTGATTTAATTC

CTATTGGATTTGGATTAGGTCGATTTCAATAGGGGTTCTTCCTCTATTTCGTCTTTTATT

ATGTTAGATGAAGGGGAAAAAATAGGAACTCAAGGATATCGAAGAGTAAAAAGAAGAATG

GAATGAAAGAGTGGTTGGTTGGAAAGAAAGAGAAATAGAATAATGAGTACACAAACCTCT

AATGATTAGAAACTAAAAATGAGATCTCGAAGTAGTTCGGACAATTCAGATTATCATTTA

TTTGTACTTTTTAGTTACTTCTCCCCAATAGAGCTTAGAAGTAAGAATTTCTTGGTTGAT

TGTATCCTTAACCATTTCTTTTTTTTGACACGAGGAACTCACCATGAATCCACTAATTGC

TGCTGCTCCGTTATTGCTGCTGGATGCCGTAGGGCTTGCTTCTATTGGGCCTGGAGTTGG

TCAAGGTACTGCTGCAGGACAAGCTGTAGAAGGTATTGCGAGACAGCCAGAAGCAGAAGG

TAAAATACGAGGTACTTTATTGCTTAGTCTAGCTTTTATGGAAGCTTTAACAATTTATGG

ACTAGTTGTGGCACTAGCGCTTTTATTTGCGAACCCTTTTGTTTAATCCTAAAAAAGAAA

ATGAGTCCTTTAGATTAGATACTTTTTTCTTTTTTAGTAAATTGGTATTTGCTTCTGCAA

TTCCAATTATATCAATACTTTACTCCTATTTATTACTCCTGGAATTACCTATTTATCGGG

ACAGACAATACCCCACCCCAGGAAGGGCTGATTTGAGGATGATCAATTTAGAGGATATGC

TCGCCTTCTTCCTTCCCGTCCTTAGTTTAGGACAGTGGAAAGTCTTTTTCCTTTTATTTT

AGGAATTTTTGGAACATTTCAACAAAGGAGTCTTTCACAGGTCAAACGAGACCTAAGACT

TAATCTAAAAGAAATTATTAGATTGAATCTATTTGCATTAAAAAACCGATCAAAAAGGGC

GAGCGAAGTAAGTGATCAAAAACTTTGCTCTTTGTTCGTCCTATCTATAAGAGGAGAGCA

TATGAAAAATGTAACCCATTCTTTCGTTTTTTTAGCTCACTGGCCATCCGCTGGGAGTTT

CGGGCTTAATACCGATATTTTAGCAACAAATCTAATAAATCTAACTGTAGTGGTTGGTGT

ATTGATTTTTTTTGGAAAGGGAGTGTGTGCGAGTTGTCTATTTCAAGAATAGATTGATCT

ATCCGGCTGCACTTTAAAATATTTTTTAGTATTTTTTGGATAAATAAGAAAAGGTGCACG

ATCTCGACGAATTACTTCTGAATAAATTCAGAAATCATATGGAAGACCATAGCATTTCGC

GACTCATTGGTAAATCAACTTTGATTCTCTATAGACCAATAATGTGAGACCATTAACACG

GTTAAAGCTAAACTGCTTGAAGTCCAGGCAAAAAGGGGTACTCTTTCTACAACTATATTA

GTATTAGTACCAAATGCTTTAAACGGGAAATAGCTAATGTAGAATTTATCTGATATAAAA

CACTCATATCGATAAAATGGTTTGAACTATTTACTAGAAGGGCACCCTGCCCTTTTTCCA

ATGCCGAATCGACGACCTATGTATAAAAAAAGAGAAATTTTTTGGATTTGAAGAAAAAAA

AGAATTCTATCAATTTTCATTTTCCATTTATTTAGTTTCTTAATGAAATTGAAATTATTA

ACTAAAGGGCAAATACAAATAAAGAAACAACTTTGCTGACCATGATAGATTTTTATCTAG

GCGGAAGAGTCCTCTTAATATTTATCTAGTCTTATATGGGTTTCGGTATATTGAAATATA

AACAGAAAAGAGAAGATAGAGGATAGGCTCATTACATAAAAAAGATATGGAAATAGCCAT

AGCAAAAAAAAAAAAGGAGCGTGAGAGCCAAATGAATCGAAAGATTCATGTTTGGTTCGG

GAAGAGATCATAAAAATTGTAAACTTAATAGCAAGATAATCTACTTTCATTAAAAGATTT

ATTAGATAATCGAAAACAGAGAATCTTGAGTACTATTCGAAATTCGGAAGAATTGCGTAG

AGGGACCATTGAGCAGCTCGAAAAAGCTCGGGTTCGATTACAGAAAGTCGAACTAGAAGC

GGATGAGTATCGAATGAATGGATACTCTGAGATAGAACGAGAAAAAGCAAATTTGATTAA

TGCTACTTCTATTAGTTTGGAACAATTAGAAAAGTCTAAAAATGAAATCCTTTATTTTGA

AAAACAAAGGGCGATGAATCAGGTCCGACAACGGGTTTTCCAACAGGCCGTACAAGGAGC

TCTAGGAACTCTGAATAGTTGTTTGAATACCGAGTTACATTTCCGTACGATTCGTGCTAA

TATTGGCATTCTAGGGGCCATAGAATCGAAGAATTAAATTAATTAGACCTTGAACTTCTA

CTTTCGTTTAGAATTTAGGCATTATTTTTCCCTTGCTTCCGAAAAAAGAGTCAAGAAACA

CTAATGGCAACCCTTCGAGTCGACGAAATTCATAAAATTCTCCGCGAACGTATTGAACAA

TATAATAGGAAAGTAGGGATTGAGAATATCGGTCGCGTAATTCAAGTGGGGGATGGGATT

GCTCGTATTATAGGTCTTGGTGGAATAATGTCAGGTGAATTAGTCGAATTTGCAGAAGGG

ACTAGGGGTATTGCTCTGAATTTGGAATCCAAAAATGTTGGGATTGTATTAATGGGCGAT

GGGTTGATGATACAAGAGGGAAGTTTTGTAAAAGCAACAGGAAGAATTGCTCAGATACCC

GTGAGCGAGGCTTACTTGGGTCGTGTTATAAATGCTCTGGCTAAACCTATTGATGGGAGA

GGCGAAATTGTAGCTTCGGAATCTCGCTTAATTGAATCTCCTGCTCCGGGTATAATTTCC

AGGCGTTCCGTATATGAACCCCTTCAAACAGGGCTTATTGCTATCGATTCGATGATCCCC

ATAGGGCGCGGTCAGCGAGAGTTAATTATTGGGGACAGACAGACTGGCAAAACAGCAGTA

GCCACAGATACAATTCTCAATCAAAAAGGGCAAGATGTAATATGTGTTTATGTAGCTATC

GGTCAAAGAGCATCCTCCGTGGCTCAAGTAGTAACTACTTTCCATGAAGAGGGGGCCATG

GAATACACTATTGTAGTAGCTGAAATGGCGGATTCACCTGCTACATTACAATACCTCGCC

CCTTATACGGGAGCAGCCCTGGCTGAGTATTTTATGTATCGCGAACGGCATACTTTAATA

ATTTATGATGATCTCTCCAAACAAGCACAAGCTTATCGCCAAATGTCCCTTCTATTAAGA

AGACCCCCCGGCCGCGAAGCTTATCCAGGGGATGTTTTTTATTTGCATTCACGGCTTTTA

GAAAGAGCCGCTAAATTAAGTTCTCTTTTAGGCGAAGGAAGTATGACCGCTTTACCAATA

GTTGAGACTCAATCTGGAGACGTTTCCGCCTATATTCCTACTAATGTAATCTCCATTACA

GATGGACAAATATTCTTATCCGCGGATCTATTTAATGCCGGAATTCGACCTGCTATTAAT

GTGGGTATTTCTGTTTCCAGAGTAGGATCCGCGGCTCAAATTAAAGCCATGAAACAAGTA

GCTGGCAAATCAAAATTGGAACTAGCTCAATTCGCAGAATTACAAGCCTTTGCACAATTC

GCCTCTGCTCTCGATAAAACAAGTCAGAATCAATTGGCAAGGGGTCGACGATTACGGGAA

TTGCTTAAACAATCCCAATCAAACCCTCTCTCAGTGGAAGAGCAGATAGCTACTATTTAT

ACCGGAACGAGAGGATATCTTGATTCGTTAGAAATTGGACAGGTAAAGAAATTTCTGGAT

GAGTTACGTAAACACCTAAAAGATACGAAACCTCAATTCCAAGAAATTATATCTTCTAGC

AAGACATTCACCGAGCAAGCGGAAATCCTTTTGAAGGAAGCTATTCAGGAACAGCTCGAA

GGGTTTTCCCTTCAGGAACAAACATAAATTTTGCATGTCTACTCTTGTTAGTAGAAGAGG

AATCAAAGATTTTTCATTTGAATCATGCAAAAAGTTTTCTTAGTTTTTAGTATAGTTATT

TAAAGAATAGATAGAAATAAGATTGCGTCCAATAGGATTTGAACCTATACCAAAGGTTTA

GAAGACCTCTGTCCTATCCATTAGACAATGGACGCTTTTTTCATATTTTCTTCTTTCTTT

TTTTTTTTTTCTTGTGATAAAAAACTCTTAGACCGAAACTCTTTTAGGAAAGAAAAATAA

ATCCATATACAAATGGATGATGCATATATCATAAAGAAGGAATATGGAGCGGGTAGTGGG

AATCGAACCCGCAACCCCAAGGTTATGAGCCTTGTGAGCTACCAAACAGCTCTATCCTCT

TAGCGGGGAACTAGGGGGTTGGATACGCCCCTCTACCATATCTATACAAATAGAATAGTC

CATTTATACAGAATGGTAAAGAGGGCTCTTCTACGATCATCCATTCTAGAAATCCATACA

AATATGAAAGGGTATTTTATCCTTACCAACTGGATCTTGTTGCACCCGGTAACAAACATG

CATAAACCATTTCTCGAAGTATGTGTCCGGATAGTCCAAAGTCTCGATAGTTAGCTCTAG

GTCTTCCGGTCAAAAAACAACGTCGATGAAGGCGTGTAGGTGCACTATTACGTGGTAGGG

ATTGCAATTTTTCTCGCATTTTCGTTTTTTCACTCAAACTCAAGGGGGAAACTTTGCTTC

TTATCTTTTTTTTTGAAGATCGACGAATCAAATGATATTTCTGTTCTAATTTCTGCCGCT

TCTTCTCCCTCTGAATCAAACTTTTTTTTGCCATAATGTGCCGTTCCTATTATTACCAAG

TATATGGTTCTAATCCTAGATGGAAAAATAAATAGAAAAAATCTAAGAAGGCGGATCCTC

CCTCTCCATCAAGAGTAATGAACTAGGTGCTGGTACAGTACAAAACTAAATTAACCAAAC

TTGCCTGATGTTGAGGCAATCAAGAAAGCTGCATAAGTGAATATATAACCCACGGAAAAG

TGGGCTAATCCAACCAATCTTGCTTGCACAATGGAAAGAGCCACGGGCTTATCTCTCCAG

CGAATTAAATTAGCCAAAGGTGTGCGTTCATGAGCCCATGCTAAAGTCTCAATTAATTCC

TGCCAATATCCACGCCAGGAAATTAAGAACATAAATCCAGTAGCCCAAACAAGATGTCCA

AATAAGAACATCCACGCCCATACCGATAAACTATTCATCCCAAAAGGATTATATCCATTG

ATAAGTTGTGAAGAGTTTAACCATAGGTAATCTCTTAACCATCCCATCAAATAAGTGGAG

GATTCATTAAATTGTGAAACGTTGCCCTGCCATAATGTGATGTGTTTCCAATGCCAATAA

AAAGTAACCCATCCAATGGTATTTAACATCCAGAAAACTGCCAAATAAAACGCGTCCCAA

GCAGAAATATCACAAGTACCGCCGCGCCCTGGGCCGTCACAAGGAAAACTATACCCAAAA

TCCTTTTTATCCGGCATTAATTTGGAACCGCGTGCATCTAAAGCACCCTTTACTAAAATC

AATGTAGTTGTATGCAAACCTAGAGCAATAGCATGATGAACCAAGAAATCTCCAGGTCCT

ATTGTTAAGAAAAGAGAATTACTATTCTCATTAACAGCATTCAACCATCCGGGCAACCAT

AGGGTTCGACCTGCATTGAAAGCGGGGCCGCTCGTTGAAGATAAGAGTATATCGAACCCA

TATGTCGTCTTACCATGAGCAGATTGTATCCATTGGGCAAATATAGGTTCGATCAAGATT

TGCTTTTCTGGAGTACCAAAAGCAAGCATGACGTCGTTATGAACATAAAGGCCCAAGGTA

TGGAATCCTAGGAAGAGGCTAGCCCAACTTAAATGAGATATGATAGCTTCTTTATGGTCT

AACATTCTTGCCAATACATTATCCTCATTCTGTTCCGGATTGTAATCCCTAATGAAAAAA

ATAGCTCCATGAGCAAAAGCCCCTGTCATGATGAACCCTGCAATATATTGGTGATGAGTA

TATAAAGCAGCTTGAGTGGTAAAGTCTTGTGCTATGAATGCATAAGCAGGTAAAGAGTAC

ATATGTTGAGCTACTAAGGAAGTAATAACCCCTAAAGAAGCTAGAGCAAGACCTAATTGA

AAATGAATCGAATTATTGATTGTGTCGTAAAGGCCCTTATGCCCACGCCCTAATCGACCC

CCCGGAGGAGTATGTGCTTCTAAAAGATCTTTGATACTGTGCCCAATTCCGAAGTTAGTT

CGATACATATGACCGGCAATGAGAAAAATAAATGCAATAGCTAAATGATGGTGAGCAATA

TCGGTCAGCCACAAACTTTGTGTTTGTGGATGGAATCCCCCAAGAAGAGTTAGAATGGCA

GTTCCGGCTCCTTGAGCGGTACCAAATAAATGATTACTCGAATCAGGGTTTTGGGCATAA

AGATTCCACTGACCCGTCAGAAGGGGTCCCAACCCCTGGGGATAGGGTAATACATCTAAG

AAATTATTCCATCGAACGTACTCCCCCCTGGATCCGGGAATAGCGACATGAACTAAATGT

CCTGTCCAAGCCAAAGAACTTACCCCGAAAAGTCCTGACAAATGATGATTGAGACGAGAT

TCCGCGTTTTTGAACCACGAAAGGCTTGGTTTCCATTTGGGTTGTAGATGTAACCAACCC

CCTATTAAGGATAGCGTAGAAAGAAATAATAGAAACAGAGCTCCAGTATAAAGATCTCCA

TTGGTGCGTAATCCAATTGTATACCACCACTGATAAACCCCAGAATAGGCGATATTCACT

GGACCGGCAGCACCTCCTCGAGTAAAGGCTTCCACAGCGGGTTGACCAAAATGAGGATCC

CAAATCGCATGAGCAATAGGCCTTACGTGTAAAGGATCCTGTATCCATGATTCAAAATTT

CCTTGCCAAGCTACATGAAACAGATTTCCGGATGTCCATAGAAAGATTATTGCTAACTGC

CCAAAGTGAGAAGCAAAAATGTTCTGATAAAGACGTTCCTCAGTAATATCATCATGACTT

TCGAAATCATGTGCGGTAGCAATACCAAACCAAATACGACGAGTAGTGGGGTCCTGAGCT

AAGCCTTGGCTAAACCTGGGAAATCTTAATTCCATAATGCCTTTCAAATCCTCCTAGCCA

CTATCCTACTGCAATAATTCTCGCTAAGAAGAATGCCCATGTTGTGGCAATTCCACCCAG

AAGGTAATGGGTTACTCCTACAGCACGTCCTTGTATAATGCTCAAGGCTCTAGGCTGAGT

AGCAGGAGCAACTTTTAATTTGTTATGAGCCCAAACGATAGATTCAATGAGTTCTTGCCA

ATAACCACGGCCGCTGAATAAAAACATTAAACTGAAGGCCCAGACAAAATGAGCACCTAA

GAAAAAAAGACCATATGCAGATAATGAAGAACCATAAGACTGAATTACTTGGGATGCCTG

TGCCCACAAGAAATCTCGGAGCCACCCATTAATCGTAATGGAACTCTGTGCAAAGTTTCC

CCCTGTGATATGAGTTACCATCCCTTCATCGCTTATAGTACCCCAAACATCCGACTGCAT

TTTCCAACTGAAATGGAAAATGACTACCGAAATTGCATTGTACATCCAGAATAGACCTAA

GAAAACATGATCCCAGGCGGATACTTGACATGTTCCCCCTCTCCCAGGCCCATCGCAAGG

GAAGCGAAAACCAAGATTTGCTTTATCGGGTATCAAACGGGAACTGCGAGCAAATAAAAC

ACCTTTCAAAAGTATTAATACAGTCACATGGATGGTAAATGCGTGAATGTGATGGACTAA

AAAATCTGCGGTTCCTAATGGAATAGGTAACAAAGCTACTTTGCCGCCTACTGCTACTAA

CTCGCCACCTCCCCACGTTAAGCTGGTACTTGTTGTTGCACCAGGAGCTGTTACGCCAGG

CGCGTTAGCATGGATATTTTGTACCCATTGAGCAAAGATGGGTTGTAATTGTATGGCGGT

ATCCGAAAACATATCTTGGGGACGGCCTAAAGCACTCATGGTATCATTATGAATGTACAA

GCCAAAACTGTGAAAACCTAGAAATATACATACCCAGTTAAGGTGGGATATGATTGCATC

GCGGTGTCTAAGGACGCGATCTAATAGATCGTTGTATCGAGTAGTTGGATCATAGTCTCT

TACCATAAAAATGGCTGCATGTGCAGCAGCACCGACTATTAGAAATCCGCCAATCCACAT

GTGGTGTGTGAACAAGGAAAGTTGTGTACCATAGTCAGTAGCTAGGTATGGATAGGGGGG

CATAGAGTACATATGATGAGCTACAACAATGGTTGTAGAGCCTAGCATAGCTAGGTTAAG

AGATAATTGAGCGTGCCATGACGTTGTTAGGATTTCATAGAGACCCTTATGGCCTTGTCC

TGTAAATGGGCCCTTATGAGCTTCCAAAATATCTTTAAGTCCATGACCAATACCCCAGTT

GGTCCTATACATATGACCTGCGATCAGGAAAAGAATAGCAATAGCTAAATGATGGTGCGC

AATATCGCTCAACCATAGACCTCCGGTTATTGGGTCTAGTCCTCCGCGAAAACTCAGAAA

TTCTGCGTATTTGGACCAATTCAAGGTGAAAAAGGGGGTTGCTCCTTCGGCAAAACTAGG

ATAAAGTTGAGCCAAAAGGTCGCGATTCAAGATAAATTCATGAGGAAGTGGTATCTCTTT

AGGATCAACCCCAGCGTCAAGAAATTGGTTAATTGGTAAAGATACATGGATTTGGTGTCC

CGCCCAAGAAAGAGACCCAAGTCCTAATAACCCCGCTAAGTGGTGATTCAACATGGATTC

TACATCTTGGAACCAGGCCAATTTGGGAGCGGCCTTGTGATAATGGAACCAACCAGCAAA

AAGCATTAACGATGCAAAAATCAATGCACCGATTGCGGTACAATAGAGTTGTAATTCATT

AGTTATTCCAGATGCTCGCCAAAGCTGAAAAAACCCAGAGGTTATTTGGATTCCTCGGAA

ACCCCCGCCTACATCACCATTCAATATTTCTTGCCCTACTATTGGCCAAACTACCTGAGC

ACTGGGTCCAATGTGAGTAGGATCACTTAGCCATGCTTCATAATTGGAAAAACGGGCACC

ATGGAAGTACATGCCACTCAACCAAAGAAAGATAATGGAGAGTTGACCGAAATGAGCACT

AAAGACTTTTCGAGAGATCTCCTCCAAATCACCGGTATGACTATCGAAATCGTGAGCATC

AGCATGTAGGTTCCAGATCCAAGTGGTAGTATCAGGGCCCTTAGCTATTGTTCTTGAGAA

ATGGCCGGGTTTGGCCCATTCCTCAAAAGATGTTTTTACAGGATCCCTATCCACAACAAT

TTTTACTTCTGGTTCCGGCGAACGAATAATCATTAAGTCCTCCTCTTTCCGGACAAGACA

TACAAAGAGACCCGCCAACTTTTTAGTGAACCTTTGAAAGATAGATATTATGATTAGTCC

TTTTCTTTACTATCTACCGTCCTTCTATTTTTTTTAGTTATTCACTGGAGCAATTATATA

TTGAAGTCAATCCGAGGCAAGTGTTCGGATCTATTATGACATAAGGATTAGGTGCCTAAC

GGACATTGTTTATTTTGGATTTCCCGACGTACTAAAAAAACCTTTTTTAATTTACGAAGC

TAGTGTATTTTTTTAGGGTATAAGCTCCTATCTACATCTACTTTCCTTGAGTATAGATTT

TTTTATTCGATTCCAAATTCCAAGATAACTCATTAGAATTATTAATAAGACGGTCCTGAT

ATATTAGCAATATTTAGATCGCCCCCTTTATTCGCTTTATTACTTCTATTCTAGACCCTA

TCGTTTATCCTTATGAAATATAATAAAAAAAAGAAGGTAGAAGAAAGGGATATAATGAAA

TTCTTGATTCGATTTACAACCAAACCCCCATTTTATGAAAAAGGAGAGTGGTCTTATTCA

AATTCAAAGCGCTTCGTAATCTTCAACCAGTTCTGTGCTTCAATATAATTTCCCGGAGTA

AGCGCTATAGCTTGTTTCCAATACTCAGCAGCTTGATCAAACCAAGCTTCCGCAATTTCC

GAATCACCCTGTAGAATGGCCTGTTCTCCTCGGTCGGAATAGGCAGTTCCTTCCCCTAGA

ACCGTACTTGAGAGTTTCCTACCTCATACGGCTCAGAAATTGCTATCCTAATTTCCCCTA

TCTTAACTGAATTCGATTTCTCAAAAATCGATCCAATTTCTTCTTGGGTTAAGCAGAAGA

AGTTAATTACCTAAGTTTCAAACCCTAATTTTGATCAATAATCAGTTTGATCTTTTCTCC

CACCTTCAGAAGAATGAAGCATAGATAGACCTATAGCCTTCGTTCGAATTTTCTGAAAGG

TAACTATCTCGGTTTCTTATATGAAATTTCTATAGAATCCTTGAAAAAGACTTTTTCCAT

AAGAAAGAAAAAAGAACTTACTATCTTTGGGATCTGATACTACACCGCTGCTTATTCCTT

AGTGGATCGGCTCTATTACATAAGCAGATTCCTAAATTTTGCCCCATATCATGGGATAAG

TAAGCAGTTTTTTTTAGTTGTATCGACCCAGTCGCTCACTAATGGATCTTTACGGTGCTT

TCTCTATCAATTTGGGCTTTATCCATAGAGTAGTATAGGCCATACTTTCTTCCTATTTTG

ATTCTCGTGAAGTGTCCTTCCTTCCTACAGCTGATAGGGCAAAATCGTTGTTTTGACGAT

CCCTATGTAGAAAGCCCTTTTTCTAGTATTTACTAGAAAATTTGATCCTTTCTTTTTCTT

CTTTCTATAGTGGAGATAGTCGCACGTAATGACAGATCACGGCCATATTATTAAAAGCTT

GCGGTAAGAAGGGGTTTCGTTCTAGTGCCCGGAAATAATATTCCAAAGCCTTTGTATGCT

CTCCATTGCTTGTGTGTATAAGGCCTATATTATAGAGTATATAACTTCGATCATAGGGAT

CAATTTCTAGTCGCGTAGCTTCATAATAATTCTGCAAAGCTTCCGCATAATTTCCTTCGG

ATTGAGCCAACATCCGTTACGGTCGTTCATTCTATTCAAAAAATCTCCGTTCCAAAACCG

TACATGAGGTTTTCATCTCATACGGCTCCTCCCTTCTGTACATAGTACTAAGCGAAAAAT

CTATAGAATAAAAATAGAATTAGTCCCATCTTATTATGAACCGAAAGGGGCTGGTATTTT

TCCAAGAAATCTCTAGCCAACCTTCCCGCAAGAGGTTTTTCTTAACACCAATGAATTCTA

TTAATGCTAGAGGAAAACGATAGCTCCAAGAATTTCTTTGTTCTCAACGCCTCCTATTTA

GAGGAATTAGCCACTTCAACGATCTTTGATGGTTATAGGGGTATCCAAAGTACAAACCTG

ATGGTTGTTTGTTATCCCAACCATTCTTCCCAGCCCTGATACCGATCAGGAAAGGGCTAA

TTTCTAACAAAGTTTTTCTCTTGTTGATTCCTATTTCTAGGTGTAGTGCTTTTCTCCCCT

ATGCTGCCTATTGGTACTAGTAGAGTAGGATTGGCCTGTAATACAGAACCTATCCTGTAG

GTGTAACCTTTCGCTCAATACTCAAATCTACAATTGAAGCATCTGAGGCCGCATCAATCG

AGGATACACGACAGAAGGAATTGTTAGTTCACCTCACCTTCCCCAAGCGTGGGTTTCCTT

TACTAATTTTGTTCTCTCTATGCGAACCCCCTCTCTTTCTCGTAAGACTGAGGTGTAGGT

AGGGCTAAAAAAAAAAAGAGTCAAATCGCACCATCTCTATAATAAGTAAATGCCCTTTTT

TCCCCTGAGGTTGTCGGAATTATTCGCAATAAAATATTGGCTACAATTGAAGAGGTCTTA

TCAATGAAATTTCCATTTATACGGGATCTAGGCATAATTCCCAACCCATTCTATATAGAA

TTGTTTTCATTTCTTCACAAAATAACATAAAAACAAAACATTGATTCTTATAAATCGATC

ATATGCTCTAAATGGATAAGAGAGGTATTTCCGCTCAGCTCAAATTGTCTCCTTTTCCTC

TGTTTGGACAAGAAGAGATATGAAAATTGACTAAGATTGGATTTCATTCCACTTTTCTAT

TTCTCACAATAACTTCTCTCATCAGCTATTTCGCGTTTCAAGTCATTAATTGTCTCATAC

CCTTTTTTATGGCGTAGCGTACTTATGCAAACAGAATTCTAGGGTTCCTTTTTATGGAAT

AAGAAGAATTCTTCCATCTCTTTTTCTTTGGTTTGAACCCAAACTAAAACTTTTCGAGGG

AGCGGAATTCCTAGTAAAAAAATCCTGGATCCTTCCACTTAGATGAAAAGGAATTTTTCC

AATAGAACCATGGAACCCCCGAGCCGTTGTGGTTGTACCTGTACTGCAGGAATAGGAAAA

CTCGCTATTCACTTAGTTTATTTTCCATAATAAGATTATGTAGGAGAGATGGCCGAGCGG

TTCAAGGCGTAGCATTGGAACTGCTATGTAGACTTTTGTTTACCGAGGGTTCGAATCCCT

CTCTTTCCGTTTCTCTTAATTGATCAACGTTAACGATCACAATGTATCAAATCAAATAAC

AATTTATTCCAGCAATAATACCTTTATTTAATAGAAATTTTTTATAGTAAATTACTGTGG

TATGTAAAATACACATAGAGGAAAGAACAAAGAAAAAGGATCCTAGGGTTAATCCATTTA

TGCTAGTTGAATGGAAAATACGAATTAAGGGCCTTAGGTCGATTTAGTTCGGGGGAAGGG

GAAGAAAATTCTATGAACTTTTCCTTTTTCGTTAAGTTCAAGTCTGACGAGAGTAATATT

CTACAACTAACAACTCATTTATTTTGAGACCGACCCACTTCCTATCTAGGATTTTTTTAA

CTAGTCCTTTATATTGCAATGTGTCAATCGTCAAATGCTTTGGCAATTTCCCCGGGTCGG

ATGAAGCAATAGAATTTTGAATCAGACGTTTTGATCTTTGGTTATCCTTCGTAGTAATAA

TATCTCGGGGTTTGCAACGAAAACTTGGTATATCGACTATACGACCATTAACTAAAATAT

GTCTATGGTTAACTAATTGCCGGGCCCCAGGAATGGTTGAAGCCATACCCAATCGAAAAA

GGATATTATCCAAACGCATTTCAAGTAATTGTAGTAAAACCTGACCTGTTGACCTTTTTG

CTTTTCCAGCGATATGTACATATCTAAGTAATTGTCGTTCTGTCAGACCATAATGAAAAC

GCAATTTCTGTTTTTCTTGAAGACGAATACGATATTGCTCTTTTTTCCCAGAATGGAATT

TCTTTTTCAGATTACTTCCGGATTTAGGTGTTTTTCTAGTGAGTCCTGGTAAAGCTCCCA

GACGGCGTATTTTTTTTAAACGAGGTCCTCGATAACGGGACATGAAGACTCCTTGTTTTA

TTGAAATTTCATTTTACACAATTAATTTCATTGTATTTACATTACAGAATACATCAAAAT

TAAAACTGAATTAAACTAAAGGATAAACAGAGTAAAATCTACTAAAGTACCACAAAAAAT

GGAATTTCATCAACATCTGGATTTTTGTATATATATTATTTATTTTATTGTTTTGTATCT

AGCAAAATTGTAAGGTAGAACCACATAATAGATCCTGATTCTCCATTTAATTCGGAGAAA

AAGAGAGATTCTTGTTCATGGAACATCGATATAGAAAAAAGCCGACTATCGGATTTGAAC

CGATGACCCTCGCATTACAAATGCGATGCTCTAACCTCTGAGCTAAGTGGGCTTACATAA

CAGAAATAGTGTAACAAATAGAAATATGTATATAGGAAATCCGTAAAATGTCAGATCTTA

ATTATTAATCTTAGCTATTAACTAGTTCGAAATTGGAAGTTCTACTTAGAAAAAAATACT

AGAACTTCATAAAATAAAGTTAAAGAAATTTTTGAACTTTCTTTTTTCTCTAATTTTTCT

AATAGAATCTATTCCAATTTCTATATTGAATTTGATTTCAGATATTTTCAAATGGCTCGG

ATGAGTAATCTAATACATAGAAAAGAATAATATATATGAAAGATATAATAAAGAGAAAAT

GCGAATTTCTTGCATTTTCATTCGATCATTATAGACATTTTTGAGATAATAATTTAATGA

TTAATATTTCACTAAGGAGAACATAGAATCATAGCAAATGAAATTGCTAATTCTGATTAG

AAAAAAAGAATGAATATCAAGCGTTATAGTATGATTTTGAATACTCTAAAAAAGAAAGGC

GGGGGAGAGAAAAACTTTGGGATATATTGATTCGGATTGAATTGCAAATACATCAACGAT

AGAATCAATTCAATTCTGAATTGCAATAAGCAGGGTCTGTCAAATAGAGACGAACTGCTA

GACTACGTCGAGTAATTAATTCAACGATTCAAAAAAAACTAAGAGATGGATGAAATTACA

CAAGGAATCCAGGTCTCAAGGAAAATGGGGATATGGCGAAATCGGTAGACGCTACGGACT

TGATTGTATTGAGCCTTGGTATGGAAACCTGCTAAGTGGTAACTTCCAAATTCAGAGAAA

CCCTGGAATTAAAAAAGGGCAATCTGAGCCAAATCCGTGTTTTGAAAAACAAGTGGTTCT

CGAACTAGAATCCAAAGGAAAAGGATAGGTGCAGAGACTCAATGGAAGCTGTTCTAACGA

ATCGAGTTGATAACGATTAATCACGGAACCCATATCATAATATAGGTTCTTTATTCTTTT

TTAGAATGAAATTAGGAATGATTATGAAATAGAAAATTCTGAATTTTTTTAGAATTATTG

TGAACCCATTCCAATCGAATATTGAGTAATCAAATCCTTCAATTCATAGTTTTCGAGATC

TTTTAAAAAGTGGATTAATCGGACGAGGATAAAGAGAGAGTCCCATTCTACATGTCAATA

CTGACAACAATGAAATTTCTAGTAAAAGGAAAATCCGTCGACTTTATAAGTTGTGAGGGT

TCAAGTCCCTCTATCCCCAAACCCTCTTTTATTCACTAACTATAGTATTTATCCTCTTTT

TTTTATCAATGGGTTTAAGATTCAATTGAATAGATTTCTTTTTTATTATAGTATCGGCAA

GGAATCTCGATTATTAACTCTATTTTTAAGTATTATTAAGTAAGCCATGCACAATGCATA

GGATTACCCCCCCTCATTTCCAAATTTCGAATATTGACATAGATACAAATACTCTACTAG

GATGATGCACAAGAAAAGGTCAGGATAGCTCAGTTGGTAGAGCAGAGGACTGAAAATCCT

CGTGTCACCAGTTCAAATCTGGTTCCTGGCACAGAAAAAAAGGATCTACCGAATAGGTAT

TGATACAAATACCTCGAGATGGGTTGGGATACATATTCGTTAATAATATAGATAGAGTAT

GATTTAATCTAAGTAGATAAATCTCTAAATAGAGGCACTTCTTTTTCTTCATTTTGCATT

TCTTAATTTTGTATTCCGCTATTCCGACGAATATTTATTTATTCTTGTTATCTTACTTTC

CTAGCTGTTCTAAGTAATGCGCGCGGTACAAAGTTCGTGGTAGGAACTTCTTTGAGTCAT

CATATTTTTCTGTTCATACGAAGGAAATGAATATGTGATTTTCCAACGAAATGAAGCCCT

TTTTGCTCAGTCTATCTGGACCTTTTGTATAATAGGAATTAATTAGAATATAATAGGTAT

TCCGTTTCATCTAGGAACAGAACGTAAAAATATTCCTTGACTTGAATAAAATCTGGAGTT

GTATTGTATAAGTGAGCATGAATTTCTTATCATTCAATGAGCATCTTGTATTTCATAGAA

ATTGGGGGTTATATAGTCCTTACGTAAGGGCCAGCCTATCCAACTTTCAGGCATTAGAAT

ACGTTTAAGGCGTGGATGATTATCATAAGAGATTCCCACCATATCATAAGATTCGCGTTC

TTGAAAATCGGCACTTCTCCAAATCCAGAAGACAGACGGGATTCTAGGATTATCCTTTTG

GGCAAAGACTTTTATGCATACTTCTTCTGGGTTATCTATACCATACTGTATTCTCGTAAG

ATGATACACGCTAGCTAAAGATCCACCGGGTGCTACGTCATAAGCACATTGGGAACGTAA

ATAATTGTAACCATATACATATAAAATGACAGCAATGGAATCCCAATCCCCTGCTTTTAT

TTGTAAAGTCTCTATTCCTCGGTGATCGAAGCCCAAAGATCTATGAACCACCTCATGTTT

GACTAGCCAATTAGATAACCAACCCTGCTGCATTGTCTTGATCTCTCCCCCTTTGTATAA

ATATTTCACATTTCGAATGCAAGTTTGAAAGATTGCCCTGCTCTTTCTTTTTCTACACAA

AGAACCCCTCCTAATTCACTAATTTGGAGGAAGATACTGGACTTTTGGATTTGAAAAAAG

TTTCAGAAGATATGTCTAAAGTAGATGGTGATTGATAGAGCAATTCTTGCTCGTAAGTTC

CAGTATAAGTACTGCGCCTAACATAAAGCTTGTGACTGGTAGTAAAACATCGATTTTTCT

TTTGAGATAGAGTTCGATCCTCAACTATTTCTCGCGATATCTTCTTACGAAGTTTTGTTA

GGGCATCTATAACTGCCTCCGGTTTAGGTGGGCAGCCCGGCAAGTAGACATCCACAGGAA

TTAACTTATCAACTCCCCGAACAGTACTATAGGAATCCGTACTGAACATTCCCCCTGTAA

TAGTACAGGCTCCCATAGCAATGACGTATTTTGGTTCAGGCATTTGCTCGTATAATCTCA

CTAAAGAGGGAGCCATTTTCATTGTTACCGTACCAGCTGTTAAAATTAGGTCTGCTTGCC

TAGGACTTGATCTTGGTACCAATCCATAACGATCAAAGTCGAATCGTGAGCCTATTAATG

AAGCAAATTCAATGAAACAACAACTGGTACCATATAGAAGGGGCCATAAACTGGAAAGTC

TTGACCAATTCGAAAGATCATTTGGTGTAGTTGAAATAACGGAATTGGAACTTGTTTGGT

CAAGTAAGGGAAACTCAATCAAACTCATAACTGTCTCAATGGAATCTTTTCCTTCTTTTT

TTTTTTTGTCTGAATATTCAGTTAAGACCATTCCAAGGCTCCTTTTCGCCATGCATAAAC

TGAACCAACAACTGGGATAAGCACGAAAATGAAAGCTTCGATAAAAACGGATACACCCAA

TACGTCGAAACTCATTGCCCAAGGGTAGAGAAAGACCGTTTCCACATCAAAAACAACAAA

AACTAGCGCAAACATGTAATAGCGTATTCGGAATTGTAACCAAGCCCCTCCCATGGGTTC

TATACCCGATTCATAACTAGAAAGCTTCTCTGGTCCTTCACTAATCGGGGCTAAAAGTCC

TGAAATCCAAAATGCAAAAATAGGAATAAGGCTTGCTATTATTAGAAATGTCCAAAAAAT

ATCATATTCGTGAAGCAGAAACATAAATGTACTCCCATTAATGTGGAATAGGCGGAACTG

AATTAGTCAATTCAAGTCAGCGTTGTCAATTTATCCAAAACTTCTCTCTTTTCCTCGGTG

AAACAAGAATCCGTTTTGCTCAAATCAAAGCACTTAGTTTAGCCTTTGTTTCCTCTGTGC

CCTGTCTTCTTTAAAGATTCATCCAATGGAATCCCGACTCCCTTTTTGATTTCCATTCTA

TTTATAATTAGAACCTAATTAAGATAGGATGACTAATGTATGCAGCCTAATGAGGAGTAA

TACAAAAATAAAGAACTCTATTTCAGAAAGTAGATCGATTTAGATAATGTATATAAAAGT

AATATACTTCAAACAAAGTAGGAATTCGCAAGATGGAGAAAATCTTGCAGTTGATTTGAT

AGAAATTCGTTTTTCTTTTCCTGTCTCTAAGATTTTCGATGAATGAGCCTGTGGTAATGC

TTTTATCTCTATTCTATGGCGCAAGCGACCGTCCAGTCTATAAACAAGTACTAATGAGGA

AATGAAAACTATACTAAAGGAAACATAGGATCTCTATCCTAAAATCTAAAAAAGGACATA

TTAGGGCTATACGGATTCGAACCGTAGACCTTCTCGGTAAAACAGATCAAACGGATATTA

TCGAAATGATTCGAACTGTTTCAAAGACCCAACATGCATTTTTTGCATTGGGCTCTTTCA

TCAACTGATGTAAAGATCAGTTAGTCCACCATAGTTTTTCTTTACGGAAAGATAATGAGA

TGGCTCCCTGTGCTCTGATTTATTATTTGTATTATGATCTATCTAGGAGCAATACCAAAG

TGTTTCAAAGGAGGATTACCTTGACTTAGGTCGCCTCCGGCCTAAATTAAATCAACCTAA

GTGAAATGGAGTCTCTATCGTTCCGCTGCAAGAGTTGACTATGAGACTTCATACACCTTA

AAGTTCATAGAACGAAAAGAAGTTTTTTGGAGGCCCTTATCCTCATTACGCCTAGCATTT

AGTGGGCTGGATATTTACCTTATCAACTAGCAAATCAATAAGGGTTCTATTTGATTAGGC

ACCTGAATTGGCACCTGAATCGGACTGAACCGACTGTTTGTCAGGCTACTGTTCTCCTAT

TCTCTCGAATCCATGAAGTAAGACATTGATTTTGCAATAAGATCAATTATGTTCATTGCA

TAATAAGCTCCCTTGAAAAGCATTGGCGCACGTGTAAGCGAGTTGCTCTACCGAACTGAG

CTATAGCCCTTGTCAGAGATATCTTAACATATAGATAATTTCTTGTCAAGATGAATATTC

TCTAATGCCAGAGGATATCCTTTGATCTGTTTACTATATAACATACCAATAACGGAGCAG

TATTGCTTATAAAAAGGATTCGATCTATAATCGATCGAAGTAATGGGTCTTCCTTTGTGG

TGATAAATTGCCTACTTAACTCAGTGGTTAGAGTATTGCTTTCATACGGCGGGAGTCATT

GGTTCAAATCCAATAGTAGGTAGGTAGGTAGAAAAATTACTAGATAGCATTGGACTTACT

TCGCTTCGCTATCTAATAACTTTTTCTACCCCTCTTCCCTTTTTCTTTGTATCAACTAAA

CCATTGGATTGTATTCAATTGGATGGGGGAATCCAATTGATAGCCTCGACTCGTATCCTA

GCTCGTCTGAGAGCTAGCTTCGCTTCAACCAACTCTTTCGTACCCTCAGCTCTACTCAAG

TTAGCTTCGGCTATTTCAAGTGCCTGTTGAGCTTCTTCCGGATCAATGTCACTACCCAGT

TCCGCATCATTTCCTAAAATGATGATCTCATTATTAACTATTCTCGCAAAACCGCTCCAC

AGAACCGCCGTTAACCATTGGTCGTTGAGGAGGCGTATTCTCAAAGGACCCATATCTACA

GCTGTGTTAATGGGGGCGTGGTTTGGTAATACGCCAATTTGGCCACTATTAGTAGATAAA

ATGATTTCTTTCACTTCACAATCCCAAATAATTCGCTTAGGAGTTAGTACATAAAGATTT

AATTTCATTTCTTCAATTTGTTCTCCTCTTCTAAGTTTATAGCTTTCGTGCTAGCTTCAT

CGATGTTACCCACCAAATAAAAAGCTTGTTCGGGTAGGCCGTCTAATTCTCCGGAAAGGA

TTAGTTGAAATCCCCTAATTGTTTCTGCAAGACCAACATACTTTCCTGCAGAACCGGTAA

AAACTTCTGCCACAAAGAACGGTTGTGATAAGAAACGTTCAATTTTTCGTGCTCTTGCTA

CAGTTAAACGATCCTCCTCTGATAATTCATCCAACCCAAGAATTGCGATAATGTCCTGAA

GTTCTTTGTAACGTTGTAAAGTTTCCTTAACTCTTTGCGCAGTTTCATAATGTTCGTTGC

CAACGATCCGAGGCTGTAACATAGTTGAGGTTGAATCTAAAGGATCTACTGCTGGATAAA

TACCCTTGGAAGCTAATCCTCTGGAAAGTACGGTAGTAGCATCCAAATGTGCAAATGTTG

TGGCAGGAGCAGGGTCGGTCAAATCGTCCGCAGGTACATAAACTGCTTGGATCGAAGTTA

TGGATCCCTTTTTTGTAGAAGCAATTCTTTCTTGCAAAGAACCCATTTCTGTACTAAGAG

TAGGTTGATAACCCACTGCGGAGGGCATTCTCCCTAATAAGGCGGATACCTCCGATCCTG

CTTGAACAAAACGAAAGATATTATCGATGAATAGAAGCACGTCTTGCTTATTAACATCTC

GGAAATATTCTGCCATAGTTAGGGCAGTTAAACCAACTCTCATACGAGCTCCCGGCGGTT

CATTCATTTGACCATAGACTAGAGCTACCTTTGATTCCTCCAAATTTTTTTCATTAATTA

CTCCGGATTCCTTCATTTCCATATAAAGATCATTTCCTTCACGAGTCCGTTCCCCTACTC

CGCCAAATACGGATACGCCTCCATGAGCTTTAGCAATGTTGTTGATTAATTCCATGATGA

GTACTGTTTTACCTACTCCAGCCCCCCCAAATAGTCCTATTTTTCCTCCACGTCGATAAG

GAGCTAAAAGATCGACCACCTTAATACCTGTTTCAAAGATGGATAATTTCGTATCTAGCT

CGATAAAGGCAGGCGCAGATCTATGAATAGGGAATGTTGCATTAATATCTACAGGACCCA

AATTGTCAATAGGTTCCCCAAGAACGTTGAAAATTCGTCCGAGAGTAGCTCCACCGACTG

GAACACTGAGAGGAGCTCCCGTGTCAATCACTTCCAATCCTCTCATCAACCCGTCTGTAG

CACTCATAGCTACAGCTCTAACTCGATTATTTCCTAATAATTGTTGTACCTCACAAGTCA

CATTAATTTGCTTACCGGCAGTGTCTCTACTCTTGACTACCAAAGCGTTATAAATATAAG

GTAACTTGCCCGGGGGAAAAGTGATATCCAGCACGGGTCCAATAATTTGATCGATACGCC

CTATGCTTTTTTCTTCAATTGTGGAAACCCCGGGACGAGAAGTAGTAGGATTGGTTCTCA

TAATTATCACATAATTTTCAAAAAAAAGGAATTTGTCGAATTTTTTCTTGTTGAATAATG

CCAAATCAAAAAAAATAGCCAAAAATCCAAAAGTCAAAAGGAAATGAATTAGTTAATTCA

ATAAGAGAGAAAGGGGGACGAGGACTTGATTTCGTTGCCCAAGCGAATCCCATTCAATCG

TTTACTCATGGAATGAGTCCGTTGGAAAGTTCAATCAATTTTTTTCATATACATTTCGCC

TTTTGTGGAGGATCTGTCCCTACTCTACTTTCCTATCTAGGACTTCGATATACAAAATAT

ATACTACTGTGAAGCATAGATTGCTGTCAACAGAGAATTTTCTTAGTATTTAGGTATTTA

CATTCAAAATAAGAAAGGGGCCTATTAAGAACTTAATAAGGATTAGGGATTGGGTTGCGC

TATATCTATCAAAGAGTATACAATAATGATGGATTTGGTGAATCAAATCATGGTTTAATA

ACGAACCATGTTAACTTACCATAACAACAACTCAATTCCTATCGAATTCCTATAGTAGAA

TTCCTATAGGATAGAACATACACAGGGTGTACGCATATATGAATGAAACATATTCATTAA

CTTAAGCATGCCCTCCATTTTCTTTAATGAGTTGATATTAATTGAATACCCTTTTTTTAG

ATTTTTGCAAAGGTTTCTTTCGCCTAATCCTATCGAGTAGACCCTGTCGTTGTGAGAATT

CTTAATTCATGAGTTGTAGGGAGGGACTTATGTCACCACAAACAGAAACTAAAGCAAGTG

TTGGATTTAAAGCTGGTGTTAAGGATTATAAATTGACTTATTACACCCCGGAGTATGAAA

CCAAGGATACTGATATCTTGGCAGCATTCCGAGTAACTCCTCAGCCCGGGGTTCCGCCCG

AAGAAGCAGGGGCTGCAGTAGCTGCCGAATCTTCTACTGGTACATGGACAACTGTTTGGA

CTGATGGACTTACCAGTCTTGATCGTTACAAAGGGCGATGCTATCACATCGAGCCCGTTG

TTGGGGAGGAAGATCAATTTATCGCTTATGTAGCTTATCCATTAGACCTATTTGAAGAGG

GTTCTGTTACTAACATGTTTACTTCCATTGTGGGTAACGTATTTGGTTTCAAAGCCCTAC

GCGCTCTACGTCTGGAGGATCTGCGAATTCCCCCTACTTATTCAAAAACTTTCCAAGGTC

CGCCTCATGGTATCCAAGTTGAAAGGGATAAGTTGAACAAGTACGGCCGTCCTTTATTGG

GATGTACTATTAAACCAAAATTGGGATTATCCGCAAAAAATTACGGTAGAGCGTGTTATG

AGTGTCTACGCGGTGGACTTGATTTTACCAAAGATGATGAAAACGTAAACTCACAACCAT

TTATGCGCTGGAGGGACCGTTTTGTCTTTTGTGCCGAAGCAATTTATAAATCACAGGCCG

AAACCGGTGAAATCAAGGGGCATTACTTGAATGCGACTGCAGGTACATGCGAAGAAATGA

TGAAGAGAGCTATATTTGCGAGGGAATTAGGGGTTCCTATTGTAATGCATGACTACTTAA

CTGGGGGATTCACCGCAAATACTACTTTGGCTCATTATTGCCGCGACAATGGCCTACTTC

TTCACATTCACCGGGCAATGCATGCAGTTATTGATAGACAGAAAAATCATGGTATGCATT

TTCGTGTATTAGCTAAAGCATTGCGTATGTCTGGGGGAGATCATGTCCACGCCGGTACAG

TAGTAGGTAAGTTAGAAGGGGAACGCGAAATGACTTTAGGTTTTGTTGATTTATTGCGCG

ATGATTTTATTGAAAAAGATCGTGCTCGCGGTATCTTTTTCACTCAGGACTGGGTATCTA

TGCCAGGTGTTATACCGGTGGCTTCAGGGGGTATTCATGTTTGGCATATGCCAGCTCTGA

CCGAAATCTTTGGAGATGATTCCGTATTACAATTTGGTGGAGGAACTTTAGGACATCCTT

GGGGAAATGCACCTGGTGCAGTAGCTAATCGGGTGGCTTTAGAAGCTTGTGTACAAGCTC

GTAACGAAGGGCGCGATCTTGCTCGTGAAGGTAATGAAATTATCCGAGCAGCTTGCAAAT

GGAGTCCTGAACTAGCCGCAGCTTGTGAAATATGGAAGGCGATCAAATTTGAGTTCGAGC

CGGTAGATACTATAGAAAATAAAAAAGAAGCGAAATAGAAAGAGAAAAAAGCAGTTACGA

AATGCAGTAATTCTTCTTTATTCTTCTAATTGATTGCAATTAAACTCGGCTCAATCTTAA

GATTGAGCCGAATAAAAATAGATCTTGATACGATCATGAGACTTGACAAATCGAGATTCC

TCTATTCTATATATTTAGAATATATAAAGGTATAATACAATAAATAAATACAAATATAGT

ATTTATTTACTTTATTGGGAAAGAATCAATGAAAAAAGATTAGGAATCGATAATATTCTT

CTATAATCCCGGGGTCTTACTAACAGGACTTCGCTGCACGGGAAGGTAAGAGAATTTGCC

CTATTTTGGAAATTACAGCTTAATAAAAAAGTCTAAGCACTATCAATCAACTGTCGTACT

TTGAATCCAATTTCAAGTTCGATTAGAAGGATAGAAAGGCCATGAGGACGGGAAAAGAAA

AATCAAATCTTTTTAATCTCCTTTTTTGCAATTTTCTTATTATCCATTCCATTCATTTTT

TTTATAGAATACTATCTATAAAAAATCTTTTTTTGCAAACTAAAAAATACAATAGTCAAT

ATTCCTTATAATAGATATACTTAATTATATTATAAGAATCCTAAGATATTTTTCGAATAG

ATAGAAATAGTAAATTTGAATTGAGACACCTATTCTATGACGGATTTTAACTTACCTTCT

ATTTTCGTGCCTTTAGTAGGCTTAGTATTTCCGGCAATTGCAATGGCTTCTTTATTTCTT

TATGTGCAGAAAAATAAGATTGTCTAGAACCGATGGGGCCGAATTTTCTCAATGTATTTC

CAGGATCATAATACAGATATTTTTTAGTGTAAGTAATATGGTAGGGTATGTGGCTCTTTC

TACACACAAATGAAAAACGGCTATGGATGCGGATATAGGCTACGAGCATAAATGCATGCA

TATGCGGAGCCGGGTATAGCGAGTTTTTTTAAGTAGATCAACAGATATTTTTTGAATAGA

AAGTCAATGTATCTAACCAATTATTTCACAGGAGTACTAGTTACTGAAGGCGATTTCAGA

ATCAAAAAAAGTAAAGTCAAAATCATTTAGCTTATTCTCTCAATTTCAATCGACCGCTGT

TAGTATATCTAATATGAATTGGCGATCAGAACACATATGGATAGAACTTCTAAAAGGTTC

TCGAAAAAGAGGTAATTTTTTCTGGGCCTGTATTCTTTTTCTAGGTTCACTAGGATTTTT

ATCGGTTGGGGCTTCCAGTTATCTTGGTAAGAATATGATATCTGTACTTCCATCTCAACA

AATTCTTTTTTTTCCACAGGGGGTCGTGATGTCTTTCTACGGAATCGCGGGCCTATTCAT

TAGCTCCTACTTGTGGTGCACTATTTTGTGGAATGTAGGCAGTGGTTATGACCGATTCGA

TAGAAAAGAGGGAATAGTGTGCATTTTTCGTTGGGGATTCCCTGGAATAAAACGTCGCGT

CTTCCTTCGATTCCTTATGCGGGATATCCAATCAATTAGAATTCAGGTTAAAGAGGGTCT

TTATCCTCGTCGTATCCTTTATATGGAAATCCGGGGCCAGGGGGTCATTCCCTTGACTCG

TACTGATGAGAAGTTTTTTACTCCACGAGAAATTGAACAAAAAGCTGCCGAATTGGCCTA

TTTCTTGCGCGTACCAATTGAAGTATTTTGAATACCGATTTCATTTTTTGAAATTGTAAG

GAGATTTTGAGTATTTATCTAAAGAAAGGAACAAACGAGGATAAGAGAAAATTGCTTCTA

ATTTGTCCAAGTGATGGCATAATATTCTTCCATTTTCATCCGAAAGGGCTTTTTCTATTT

CTCTATTCCACTCCATCTAGATCTAAGAAAGAACCCAATGCAATGAAATTCCACTAGTAT

ACAAAAAAGAGGAATAGATACAAGGTCTCAAACCTTGTTATAGAATTTTTGCTTCAAATA

AAAGAAATATCATATATATTCATTTACAGATCAAAATGAAAAAAAAGAAAGCATTGCCTT

CTTTCCTATATCTTGTATTTATCGTACTTTTGCCCTGGGGAGTCTCTTTCTCTTTTAACA

AATGTCTGGAACTTTGGATTAAGAATTGGTGGAATACCAGGCAATCTGAAACTCTCTTAA

CTGATATTCAAGAGAAAAAGGTTCTAGAAAGATTCATAGAATTAGAAGAACTTTTTCTCT

TGGACGAAATGATAAAAGAGAAACCGAAGACACATGTACAAAAACCTCCTATAGGAATAC

ACAAGGAAATAATACAATTGGTCAAAATAGATAATGAGGATCATCTCCATATCATTTTGC

ATTTCTCGACAAATATAATCTGTTTGGCTATTCTAAGTGGTTCTTTTTTTCTGGGTAAAG

AGGAACTTGTCATTTTGAATTCTTGGGTTCAGGAATTCTTCTATAACTTAAATGACTCAA

TAAAAGCTTTTTTGATTCTTTTAGTTACTGATTTTTTTGTTGGATTTCACTCCACCCGCG

GTTGGGAACTACTAATTCGTTGGGTCTACAACGATCTTGGATGGGCTCCTAATGAGCTAA

TTTTCACTATTTTTGTTTGTAGTTTTCCAGTGATTCTAGATACATGTTTTAAATTTTGGA

TCTTTTTTTCTTTAAACCGTCTATCTCCTTCGCTTGTAGTCATTTATCATTCAATTAGTG

AAGCATAAACTCATTCGATTTCCTGATATTAATCAAATTAGCATCTTTCTTCCTTTAGAA

AGAAAGCCCTTTTCCATTTTAGCAAAATTCTTTCTATTTCTACCTGCTCAAGGTATTCAT

CATTCCAGTACAACTGTTGCAGTAGAATGACAACAGACTCGTGTATAGGGAACTAGATTA

GCTTAGCTACCTATCTAATTTATTGTAGAAATTCTGGGATCTGCGATTGGATATGGAAAA

TAGAAATACTTTTTCTTGGGTAAAGGAACAGATGATTCGATCGATTTCTGTATCGATCAT

GATATACGTAATAACTCGGACATCTATTTCAAATGCATATCCCATTTTTGCGCAGCAGGG

TTATGAAAACCCACGAGAAGCAACCGGACGAATTGTATGTGCCAATTGCCATTTAGCTAA

TAAGCCCGTGGATATTGAAGTTCCCCAAACTGTGCTTCCCGATACTGTATTTGAAGCAGT

TCTTCGAATTCCTTATGATATGCAACTGAAACAAGTTCTTGGTAATGGGAAAAAGGGAGG

GTTAAATGTGGGTGCTGTTCTTATTTTGCCCGAGGGATTCGAATTAGCGCCGCCCGACCG

TATTTCTCCTGAGTTGAAAGAAAAGATAGGAAATCTTTCTTTTCAGAGTTATCGTCCCGA

TAAAAAAAATATTCTTGTGATAGGCCCTGTTCCCGGTAAGAAATATAGTGAAATCGTCTT

TCCCATTCTTTCCCCCGATCCTGCTACGAAGAAAGACGTTCATTTCTTAAAATATCCCAT

ATATGTAGGGGGAAACCGAGGAAGGGGACAGATCTATCCTGATGGTAGCAAGAGTAACAA

TACGGTCTATAATGCTACGTCAACAGGTATAGTAAGAAAAATACTACGTAAAGAAAAGGG

GGGATATGAAATATCCATAGTCGATGCATCGGATGGACGCCAAGTGATTGATATTATACC

TTCCGGGCCAGAACTTCTTGTTTCAGAAGGGGAATCGATCAAGCTTGATCAACCATTAAC

AAGCAATCCTAATGTGGGAGGGTTTGGTCAGGGGGATGCAGAAATCGTGCTTCAGGATCC

ATTACGCGTCCAAGGCCTTTTGTTCTTCTTCGCATCTGTTATTTTGGCACAAGTTTTTTT

GGTTCTCAAAAAGAAACAGTTTGAAAAGGTTCAATTGTACGAAATGAATTTCTAGGTCCC

GGCTTACCATCAAGTTGGTAAAAAGCCGCGATGCCTGGAATTCCTTATTTCTATCTCATG

CAAAAGAAGAGAATTCAAGGCAAAGGCGAGAACAATAAAAGGATTTCCTCCTTTTAGGAG

GGATTGCTGGTCTTCTTAATCCTCTATTGGGCACAAGAAAAAGGCTTTTTTGCCTTTTTC

TTGTGTCGATTCTTCTTGTATCAAATCAGAATCATTTTTCTTCCTATTCGTCAAAGATTA

CTATTTCTTCTTTATTCGGGTCTGTCTCTTGGACCTCTTTTGCTTAGGTTCAGGCGAGGT

AGTGGACAAACGGAAAGAAAATATGGCGGGGACAAATTTCTTGTGAGCAAATAGAATTGC

TTGACTTGTTCAATTAAGTTCAACTTCGAACTTACAGAATTTTTGCAAAAAAACGTATAC

TCTTCTATTGAAGGGTGGTTTTTCTTTTTAGGCTAGTTGAGTAGTTTTTATTAAGGTTTT

ATTAGTTTATTACTCTAAACTAAATCAATGATTTGCAGGATACTTCTTCCGTGGAATAAA

ATATTGGATCCTCCCCCTTCTTTCTTGTTGCTTCATAAGAGTGAATCAATTTCATGGGCG

AAGGGATAAATCAACCGATGGATTGCTTCACTAACACCATTAACAAACAAAAGAATAAAT

AGAGGGATTCTGACCATCAGAGCAAAGGTTTCTCTTTGTTATTTTTACAAATCGAAATAG

GTAACCCATTTGTAGGTTATGGAATAAAGTTATATTATAAGAGTAAGAATTCCGCGGGTC

CTTTCCGCTCTAATCAGATAAAAGGGGGTAAGGACCCGCTAAGCTCCTACTTTTTCATGT

TTACAATCTGGTCCCTCCGATTACTATAGAGATGAACCCAATCCAGAATATGAACCGTAA

AAGAAAACACCTATTAAACCAATCACAGGAATACCAGTTACAGTACCTATCAGCCAAAGA

GGAATTCTTCCAGTAGTATCGGCCATTTCCCCTACTTTCCTCCACATTTTATCAAGTGGT

CATGCTAGAGACAAAAACAGTCATGGATAGTTATAAGGATGGTATCCTTCCAAATGGGAT

AAGAGAATTCTTACTACTCTCTTTCTTTCTCTCAATTGAAGAAGTAATTGGAAAATAAAA

CAGCAAGTACAAAAATGAGTAATAAACCCCAGTATAGACTGGTACGATTCAATTCAACAT

TTTGTTCATTCGGGTTTGATTGTGTCATAGTTCTATAGTTGGAATTTGGTTTATCGTTGG

ATGAACTGCATTGCTGATATTGATCCCAAGAAAAAAACAGTAGGTACGGCTAGTCCGTGA

ACAGCCAGCCATCGGACTGTAAAAATAGGATAGGTTCGATCTATGGTCATTGAGGGCCTC

CTAAAAGGATCTACTAAATTCATCGAGTTGTTCTAAAGAATCAAAACGGTCGGTTATTAA

GGGAATTCCTTGTCGGCTTTCCGTGAAATACTCGTTTGGCCGAGGACTTCCAAACACGTC

ATAAGCTAAACCCGTACTGACAAATAACCAACCCGCAATGAATAGGGAAGGTATAGTAAT

GCTATGAATAACCCAGTATCGAATACTGGTAATAATATCAGCAAAAGAACGTTCTCCCGT

GCTTCCAGACATGCTGAGCTCCCCAAATTTTTATTCAAAAAAGGAATTGATTCCGTAAAA

GATGGGATCAACCAGTAAATAGAAAATTACTGATATTTCATCCTTGTGAGATTGTCAATT

TTGTACCAAAGGTGTATTTTGAGTATACCGAATTAGTATAGCTATCCTTCCTATGGCACA

GCAATCTAGTTTTGCTTGGTCCCGAAACATAATTCTTTTTTTCTCTTCTTTGTTCCTTGT

CTATAGGTAAGCTATATGTTATTCAAGGCATCAATAGAAAACCCCATTTTTGGGGTCCTG

CTTATTTTCATTGGCTTCGGATTAGTAGAATAATTCGGAATAGCGGCCAAGATCTTGGGA

AAATCCAAGTTAATGATCAATAGGATAAATAATTTAGGAAAGATATTCTCATACTGACAC

AATATAAGGACAAGTATATGCGAAATCTATCCCTTAAGGAATTTAATTGGTTAGCATAAT

ATCTAATAAATAGAAAATCGAATAGTGGATAATCTGTTATGAGAGAAAGAAAACATTCTT

TGAAGAATCAAGATTCGTAATCAATCCTTGCCTTGTTTACTAACTTTCTTGACTAAACTG

CAAGCGTGGAACTCAGGAAACTTCGAATCAACTTTGGTTGGGGTTCAAAAAGGAATAAAA

ATAAAGTAAATTCAAGGAAGATTTCCTTTTTTTAAGGGGCCCTCGGGGGTCGTGGAATGC

TTTTCTTCTCCTCTTATTCCATATGGAATACAATCAGTTAAAATAAGAAGGAATAGGGAA

TATTCGACTGTTTCAATTCTTTATTTATCTTATATTCAAAAATTCTCCCAAAATCCAATT

TAATTTTTCAATGGGGTTAGATGATCTAGTTCTTAATATTATTACTTTAAAGAACTGACA

GATTCCACAACAAATCTCTTGATTCGGAATTAGAGACTCATGTCCCATCTGATGAATCGA

TTTTCTTTTACACTTCTGTATCTCACTCTATCTTGTTTTTTAGTATTATCTAAAATAACC

GATGAATTATGAATTTTCCATAACTTAGGTAAGTGCTTTACCAACATATGTAGTGTAGTA

AAAAAAATGGAATTTAACCCTTTCATGCTTACTATAACTAGTTATTTCGGTTTTCTACTG

GCTGCTTTAACTATAACCCCAGCTCTATTTATTGGCTTGAACAAGATACGTCTTATTTGA

AATGAATTGAAAATATTTCTTTTGGATTCCTGGTATTCTAGCACTAATTATCAATTCTTT

TCTTGGTCATTGAGATTCGTGGATAATTTAGACTACTATTTAGGGATAGATCGTACCTCT

TTTTTTATCCCCTCGAACAAATCGAAATGATTGAAGTTTTTCTATTTGGAATCGTCTTAG

GCCTAATTCCTATTACTTTAGCGGGATTATTCGTGACTGCGTATTTGCAATACAGACGTG

GGGATCAGTTGGATCTTTGATTGAGTAATATTTCTTTTTTGATTGACCTCCTCCAGACCA

GAGAGGAGGTCAAATTGGAGTTGCAATTTGTTTTGTTAAGTTATTTTAGCTTCGACATAA

GATAGATGGAATCACGCTCTGTAGGATTTGAACCTACGACATCGGGTTTTGGAGACCCGC

GTTCTACCGAACTGAACTAAGAGCGCTTTCAAAAAGAAAACCCTTTTCTACTCCTAACGT

GTCTCACGTACGTATAGTATCCACAAATTCAAGTTATACCCACTTTAATTGATCTCCTCG

CTACTGCCCATAAAGAAGAAAGAAGTAATAGGTAGGGATGACAGGATTTGAACCTGTGAC

ATTTTGTACCCAAAACAAACGCGCTACCAAGCTGCGCTACATCCCTTTTCCAAATTGTTG

TATAATGGCATTGTACACAATTCCTGTCTTGTTTTCCACATCGTAATTTTCTTCTCTTTC

TCTATCTATATAGAACCTTCTTGTCATTTCTTCTTTTTGGTCTCATATAATCAAGGAATG

GTATACATCTAAATCCTATCTAATTTCACCTATAAAAGAAAGATTACTATTCCTTGGTAA

TGTATAGGAAGGGGTCTTTTTAGTTAGGAATTTCGCCTAAACAAAAGAAATACAAATGAT

CTTGGGCAAGAGTATCTGATCATATATGTATTCCAATAAGGAAGGAGGATTTTCAATGCG

GGATATAAAAACATATCTCTCTGTAGCGCCCGTGCTAAGTACTCTATGGTTTGGGGCTTT

AGCAGGTTTATTGATAGAAATTAATCGTTTATTCCCAGATGCTTTGTCATTCCCTTTTTT

TTAATTCTAGTTATTGCTATGCGAGGAATTCTTCGTGACATGACGCAAATTTTCCCTTTT

TCAATCTTTTTTATAGGAAGGAAAAAGAAAGAAAAGATGGATAAGGATTGTATTCTTTAA

TTATTTCTCTATTTTTTATTACTTAATTTAAGAATTTTAAAAATTTTTTATTCGAATGGA

TTTTATTCTTCTTCTTGGGATTCAAAATAGAAGAATAACAGAATAAGTAGAAGAATTAAG

TTAAGTCAATCCAAAAAAGAAAGGAGGTTCATGGCCAAGGGGAAAGGTGTTAGAATCAGA

GTTATTTTGGAATGTATCAGTTGTGTTCGAAAAGGTGCCAATGAGGAATCGACGGGGATT

TCTAGATATAGTACTCAAAAGAATCGCCACAATACACCCGGACAATTAGAATTAAAAAAA

TTTTGTCGTTATTGTCGCAAGCATACGACTCATGACGAAATAAAAAAATAGGAGCATCGT

GTGTTCGATCTTTCCAAAGAACAGATTTAATATATAGAACATAGATAGAATACAAAATAC

AAATCAATTCATTTGATTTCAGGTAGATATTATTTCATATGTATAGAGGGTATTCATATA

CTATATGGACCAAAGAAAGACTACTTCTTCTGGATCCAAAATTAATAAAATAAAGAAATC

CATTTTTTATTTTAAAATAAAGAATAAATCATGTATACATCTAAACAACCTTTTCATAAA

TCTAAGCAAACTTTTCATAAATCCAAGCAAACTTTTCATAAATCCAAGCAAACTTTTCGT

AAATCCAAACAACCTTTTCGTAGGCGTCCTCGGATTGGCCCGGGGGATCGAATTGATTAT

AGAAACATGAGTTTAATTAATCGATTTATTAGTGAACAAGGAAAAATATTATCGAGACGA

ATAAATAGATTAACCTTGAAACAACAACGATTAATTACTCTTGCTATAAAACAGGCTCGT

ATTTTATCTTTCTTACCATTTCGTAACTATGAGAATGAGAAGCAATTTCAAGCCCAGTCA

ATTTCAATAATTACTGGTCCTAGACCCAGAAAGAATAGACATATTCCTCAATTAACGCAA

AAGTTCAATTCCAATCGAAACTTAAGAAACTCCAACCAGAATTTAAGAAACAACAATCGG

AACTTAAGTTCCGATTGTTGATGTTTTATTCGAAAGGGCCAGACCTATATAAAGAAAGTA

ATCCAGTTTTGATTCTTGTGTTTGTTATAAGAAAGAAAAATGGGGAAGAATAAATAGTTT

TTTTATTTATTGCAACATGCTCGTTGATTCCTACCACTTAATCTTAATTTATTGTATCTT

CCCGGAGTTCCCTCTCCGGGAATTCGGTTTTAATTATTCCTGTATATTACTTTTTTATCC

TTTAATTGAGGATCTTTATTTTATTGGAAATCGTGTAAAGATTATTTGGATTTGATACAG

CTACTTGTGCAAGCATTTTACGATTAAGAATCAATTCCTTCTTGTACAGATTGTGTATTA

ATTTACTATAACTATCGAATACTTTATATATCCGCGTTGCTGCGTTTATCCGAGTGATCC

ACAAACGACGAAAATCCCTCTTTTGCCTGCCTCTATCTCGATGAGAGGAAACAAAAGCTC

TTCTTACTTGTTGAGTAATCATTCGATTAAGTCTTAAATGAGCCCCTCTAAAGTTTGAGG

CAAATGAACGCATTTTTGTTCGTCGTCTCCGAGCTATATATCCTCGCGGAACTCTGGTCA

TTGAATCAAATTAACCTTAATGAATAACTAATGATTTCTCTTCTTTTAGCCATCCTTTTT

CCCATTAATAACAAAACGAATTATTCCGATATATAAAATATTAATTCCAATGGCTTTTGC

TACTGTAACCTTCCCAACCACGATTTTTTATTCTATTCAGTTATTTCGCACGATACTAAA

AAATAGTGGGTTCCATCGTTTCTATGGTTCCCTTTTAAACGGTGAGGCCCTCTCTATACA

CCGGAGCCCTTTCTTTCATCAAAAGGTATTGTGAACTTGTATAGTTCACATTCTTTGGCT

CTACCTATCCATTATAGAGTAAATAGCTCTTTTCACAATAAGAGTTATCCATACAGTGAC

GGCATTTAATTATGAAAGTTGCTAAGTAGCTGACCCTGTTAGTCCGTTCTTTTAAGATAA

AGGAGCATAAGCCTTTTTCTTTTTATTACTATTTCCTCCGCTTAATGGATACCATTTTCT

ACCAATGGGGAATTGCTTCTTCCAATTTAGATGATTGGATTTGCACCAAAGGAAACCAGA

AATTCCATATACCATAGAATAGGATAGAGAAGCTCTATCCTATTCATTGGTACCGATCAT

GGATACTTCAAAAATTGTCTTATTTGTTTGAACTCATGATCTGAACGAGTCGCACATACA

CCCTAGCACATGTTCCTCGACGCTGAGGACATCCCTTAAGCGCGGGCGATTTTCTAGCAT

TTCGTATTGGCTGTCTTGCGTTTCTAATAAGTTGTTTAACCGTTGGCATGTCGTATGTAT

ATAGAAAAAGGATTGGTTTAGATCGATCTTAACCTGATGATTGATCATCATGAAGTATTT

CTATCGCAGAAAACCTGAATTTAGGTTTGAAATAAATTTACAAGAAATCCGGCCACTACC

AATCCTTAAACATTTCTGGAAACCACACTGGATCAGTATCGCAGTGCTCGTCAATCATTT

CATCCCCTACAATATCGACAAGTCCATAAGCTTTGGCTTCGTCTGCTGACATAAAAACAT

CCCTTTCCATGTCTTCGGATACAACCCAAAAAGGCTTGCCTGTTCTTAGGGCATAAACCC

TTGTGATCATTTCGCGAACTTTGTGTAACTCTTCCACTTCTAGTAAAAATTCTGGTGTCC

TTGCCCGATAATAAGCACTAGCAGGTTGGTGAAGCATAATCCTCGCGTGAGGGAATGCTA

TACGCTTGGTGGGTTCGCCTCCAAGCAGAATGAAGGATGCCATGGACGCAGCCATTCCGA

GGCATATTGTATATATATCTGGTGTCACCGTTTGCATCGTATCAAAAATCGCCATTCCTG

AGATTAGCCACCCGCCTGGGGAGTTTATAAACAAAAAAATATCGCTAATTCCATCTTCTA

TACTGAGATATACCATGAGACCTGTAATATGATTCGTGACCTCGCAACGAATCTCTTGAC

CTAAAAAAAGTGTCCTTTCTCGATACATAACATTGTATAAGTCAACCCAAGTCGCTTCTT

CATCTCCGGGAATCCGGTAAGGTACTTTTGGAACACCAATGGGCATATTAGATTAATATT

ATTAAATTTAAGTAAGAAAACTACACTTTAATATGGAAACGTAAGAATGGAAGAGAAAGA

AAGAATCCGCAGTTTATTGTCTTTTTTTTTTCTTATTCTATATGAATACTATAGATTCTA

TTAATACGTAGATTGAAATATACTATAAGGAAGGTAAGACAGAAAGAAAAAGGAATCGGT

GATTGGAATGATAAACAAAGAGGGATAGGGATCTATTCTTCGTTTTTCCAAATAGGCCAA

GCTACCCATTGCATATTGGCACTTATCGAGTATAGAATAGATCTGCTTCTCTTTCTTCTT

ACGAACAGAATTGGCTTCTTATTTTTAATGGAATGAAATAAATATTCACGCTTTCTGACA

CAGAATCCCCTAGAGGGGTTAGGTACATAGGATATAGATAGTCTTTTCCAATGCGATAAA

ATAAAGCGACATCGTGTCTATTTTTCTTTGCTAAAGGGGTATTTCCATGGGTTTGCCTTG

GTATCGTGTTCATACTGTTGTATTGAATGATCCGGGTCGATTGCTTTCGGTGCATATAAT

GCACACAGCTTTAGTTTCTGGTTGGGCCGGCTCGATGGCTTTATATGAATTAGCGGTTTT

TGATCCCTCTGATCCTGTTCTGGATCCAATGTGGAGACAAGGTATGTTCGTAATTCCCTT

CATGACTCGTTTAGGAATAACCAATTCGTGGGGTGGTTGGAGTATTTCAGGAGGAACTGT

AACGAATCCGGGTATTTGGAGTTATGAAGGTGTGGCAGGTGCGCATATTGTGTTTTCTGG

CTTGTGTTTCTTGGCAGCTATCTGGCATTGGGTATATTGGGACCTAGAAATATTCTGTGA

TGAGCGGACAGGAAAACCCTCTTTGGATTTGCCCAAGATCTTTGGAATTCATTTATTTCT

TGCAGGGGTGGCTTGCTTTGGCTTTGGCGCATTTCATGTAACGGGTTTGTATGGTCCTGG

GATATGGGTGTCCGATCCTTATGGACTAACTGGAAAAGTACAAGCTGTAAATCCAGCGTG

GGGTGTAGAAGGTTTTGATCCTTTTGTTCCGGGGGGAATAGCTTCTCATCATATTGCTGC

GGGTACATTGGGCATATTAGCGGGCCTATTCCATCTTAGTGTCCGTCCGCCTCAACGTCT

ATACAAAGGATTACGTATGGGCAATATTGAAACTGTACTTTCCAGTAGTATCGCTGCTGT

TTTTTTTGCAGCTTTCGTAGTTGCCGGAACTATGTGGTATGGGTCAGCAACTACCCCAAT

CGAATTATTTGGGCCTACTCGTTATCAGTGGGATCAGGGATACTTTCAGCAAGAAATATA

TCGAAGAGTTAGCGATGGGTTAGCCGAAAATCTTAGTTTATCAGAAGCTTGGTCTAAAAT

TCCCGAAAAATTAGCCTTTTATGATTATATTGGTAATAACCCGGCAAAGGGGGGATTATT

CAGAGCAGGCTCAATGGACAACGGGGATGGAATAGCTGTTGGATGGTTAGGACATCCCGT

CTTTAGAGATAAAGAAGGACGCGAGCTTTTTGTACGCCGTATGCCTACTTTTTTTGAAAC

ATTTCCGGTTGTTTTGGTAGATGAAGAGGGAATTGTGAGAGCGGACGTTCCTTTTAGAAG

AGCAGAATCCAAATATAGTGTTGAACAAGTAGGTGTAACGGTGGAGTTCTATGGTGGCGA

ACTTAATGGAGTAAGTTATTCTGATCCTGCTACTGTAAAAAAATATGCGAGGCGTTCCCA

ATTAGGGGAAATTTTTGAATTAGATCGGGCTACTTTGAAATCGGATGGTGTTTTTCGCAG

CAGTCCAAGGGGTTGGTTCACTTTTGGTCATGCTACCTTTGCTTTGCTCTTCTTTTTCGG

ACACATTTGGCATGGCGCTAGAACCTTGTTCCGAGATGTTTTTGCTGGTATTGATCCAGA

CTTGGATGCTCAAGTGGAATTTGGAACATTCCAAAAAGTTGGAGATCCAACTACAAGGAG

ACAAGCAGTCTGATACCACATTGCTATGGTATCTTTCATCTCTCTTTTTTGATTTGACAT

GGGAAACATCTCCCATCCTTTCTTTGACTCTTTTTCTTTCTTTATATGGGAAAAGATCCC

AAATGACAAATGAATAGGTGTGGAAGTTATAATTGTAAATAAACCACGATCGAATCTATG

GAAGCATTGGTTTATACGTTCCTTTTAGTTTCGACTTTAGGGATAATTTTTTTCGCTATC

TTCTTCCGAGAACCACCTAAGGTTCCGACTAAAAAATGAAATAATTTCATTGAAGTAAGA

AGTCTCCCAGAGGGGAGACTTCTTACTTCAATTAGTCCCCGTGTTCTTCGAATGGATCTC

TTAATTGTTGAGAGGGTTGCCCAAACGCGGTATATAAGGCATACCCAGTAAAGCTTACAA

GTAAACCAGATATGGAGATGGCGACTAAAGTTGCTGTTTCCATTTTTATAGAATTTCAAG

ATTACAATGGATCTACGAAAAGATCTTGTATTTACAACTACAACGGAATAGTATACAAAG

TCAACACCAATGATTAAATAGAATTTATGGCTACACAAACCGTTGAAGATAGTTCTAGAC

CTGGGCCAAGACGAACTCGCGTAGGTAGTTTATTGAAACCCTTGAATTCGGAATATGGGA

AAGTAGCTCCGGGTTGGGGGACTACTCCTTTTATGGGGGTCGCAATGGCTTTATTCGCGA

TATTCCTATCTATCATTTTAGAAATTTATAATTCTTCTGTTTTACTGGACGGAATTTTAA

TGAATTAGGTTTCTACTAACTAAAACTACGAAGTCATTGTTTTTCCATCCAAAAAAGCCT

TTCTACTTTAAGCTATACATTTCTAGACATTCTGGTAGTTCGACCGTGGATTTTTTTGTT

TTGGTATCTCTGGAATATGAGTGTGTGACTTGTTAGAATGGATCCTATTGATAATACATA

GAAAGGGCCTGTTATCTCTATCAAGATGATTCTAATTCGTCGGATATTTTTTATTCTAGT

ATCTGGAACACGAAATAGATAGAGTGGATCAAAAAAAATGGAACTATGATTCATACTCAC

TATTCAGACCTCGCAACCAGACTGAAAAAAATTCAAGTAGTTTTTAATAAAAAAAGAAAT

TTTCTTCCTTCCAATTTTGTTTGCCCAAAAGACAACTTTTTTCTCTTTCAATAAATGATC

ATCAAGCGGTTCTTATTCGAAGAACCCTTGCCTTTTGGTTAGCTTGAGACTCAATCATCG

TGGCTCTAGTATGAATCTAAGGTTTTAATTGAACTGATTCATAGGATCGCAACAAGATAA

TTTCTATCAGAAAACTACTAGAATTTTTGCTTTATTTATTTACTAGTCAAAAAAAAAAGA

AATCCAAAATAGGGAAGAGAAAAGTCAAGAAGCCTCTAATGACCAACATAAGGGAAAGAA

AGACAGATGAGCCAACTTGAGATTTTTTGGCATTATCATCACAAAGAATAAGTTCTGGAT

TTTTCTTATTTCATATCTTCAAGGCAAATCGACCCAATCCAGTGGCTGATGAAGTTTTGA

ACCTTTTTTCTAATATCCGTTGAAAATTTGTGTGTTTCTGCTTGAGCCGTACGAGATGAA

ATTTTCATATACGGTTCTCGGAGGGGGACTCGGGTTAGTTACCTATCTCAATAAAGTATA

TGATTGGTTTGAGGAACGTCTTGAGATTCAGGCAATTGCGGATGATATAACTAGTAAATA

TGTTCCTCCTCATGTCAACATATTTTATTGTTTAGGGGGAATTACACTTACTTGTTTTCT

AGTACAAGTCGCTACCGGTTTTGCTATGACTTTTTACTACCGCCCAACCGTTACAGAGGC

TTTTTCCTCGGTTCAATACATAATGACCGAGGCCAACTTTGGTTGGTTAATCCGATCAGT

TCATCGATGGTCAGCAAGTATGATGGTTCTAATGATGATCCTGCACGTATTTCGTGTGTA

TCTCACAGGTGGATTTAAAAAACCCCGCGAATTAACTTGGGTGACAGGTGTGGTTTTGGG

TGTATTGACTGCATCGTTTGGTGTAACTGGTTATTCTTTGCCTTGGGATCAAATTGGTTA

TTGGGCAGTCAAAATTGTGACAGGTGTACCTGAAGCGATTCCGGTAATAGGATCGCCTTT

AGTGGAGTTATTACGTGGAAGTGCTAGTGTGGGCCAATCCACTTTGACTCGTTTTTATAG

TTTACATACCTTTGTACTGCCTCTGCTTACTGCCGTATTTATGTTAATGCACTTTCCAAT

GATACGTAAGCAAGGTATTTCGGGTCCTTTATAGGGAAGCCATATCATAGAGAATTCTAA

TTTTCATATATCATATCGGGTAGGTTGTGGTATTTCATTGCTACAAACATGGGTTATTGT

AAAATAAGACATGTCATTTGGATACTTTTCTTCAACTCCGAAGTATTTTGATACAAATAG

TTGAAGTTCATTTTATGAAAGAAAATAAGGCGGATTATGGGAGTGTGTGACTTGAATTAT

TGATTTGGCCATGCAGATAAAGAATTGGATCTGCCACATTAGAATTCACAACAAAGTGTC

TCCGCATCCAATCAACACGTAAGTCCCCTATCTAGGAAGGATAGGCTGGTTCACTTGAGG

AGAATATTTCTATGATCATACCCCAACCATGTCATCCATGAACAGGCTCCGTAAGATCCC

ATAGAGTAGAAATAGAATAAGTCATGTGACATGATCCAATTCTCTATTTATTACACTTAC

TTTTTATTATAGTATGGAAATGCATTCATTTTCTTTGCATCGATTGCGATCCGCAATACT

ATCGGAGTAAAAGAAGGTATCTAAAGAAGAACGTAGGCTAGACTTTTTGATTTTTTATTA

GTAACAAGTAAATACTTTGTTTGGACGTAAGAAACTTGCGATATTGAGGGGATAAACACC

AACTAATCAAGAGACAATCCACAAAGCAATTGATCATGATCAAATTTGCAAGCCAACTTG

GATATTGAGCATTTACCCATAAGAATAAGATTCTTTTCAATAAGTAGTTGTAGGTGCAAC

TTCGGAAAAGAGAATCTGATAAAGCTTTTCTTACCTAGAGTCATTGAGTCATTATATACC

TTATTCTATTATGGATCTTCCACGGTCTTTTTTCTTTCATTCTTGCTCGAGCCGGATGAT

GAAAAATTCTCATGTCCGGTTCCTTTGGGGGATGGATCCTAAAGAATTCACCTATCCCAA

TAACAAAGAAACCTGACTTAAATGATCCTGTATTAAGAGCAAAATTAGCTAAAGGGATGG

GACATAATTATTACGGGGAACCCGCCTGGCCCAACGATCTTTTATATATTTTTCCAGTAG

TAATTCTAGGTACTATTGCATGTAATGTAGGTTTAGCGGTTCTCGAGCCGTCAATGATTG

GTGAACCGGCGGATCCGTTTGCAACTCCTCTGGAAATATTACCCGAGTGGTACTTCTTTC

CCGTCTTTCAAATACTCCGTACAGTACCCAATAAGTTATTGGGCGTTCTCTTAATGGTTT

CTGTGCCAACGGGCTTATTGACAGTACCCTTTCTAGAGAATGTCAATAAATTCCAAAATC

CATTTCGTCGCCCAGTAGCTACGACCGTTTTTTTAATCGGTACTGCAGTAGCTCTTTGGT

TAGGTATTGGAGCAACATTACCCATTGAAAAATCCTTAACTTTAGGTCTTTTTTAGGGAT

TTTTCAGGTTGATTCATTCAACCGTGAAGTACCGTGCATAGGTATCTAGGAAATAGTTAC

TTCCAAGTGAATCTTCCCTAGATACCTAAAATCCATTTTATTATGATCCATTTCGCGAAA

ATATAGATTGTGCCAAAGATGCAAAATTGTTTTTTTTTATTCTAACTCGAAAAAGAAGAA

GAGGAAAAAATTGCAATGATTTAAAACGAGAACTTATTCTTAGGTAAATCGATTGGGAGA

TGCTTCTCTAGAGTGTCCCATATCTGTTTTCCATCTTCCATACGAAAACTGTCAATTCTC

ATAAGATCTTCTTCAGTCTTACTCAAAAGGTCCAATAGTGTATGTATATTGGCCCTTTTG

AGACAATTATACGTTCTAGAAGGCAATTCTAATTGATCAATAAAAATACAATTCAATGGA

ATTCCTTTTTTGTTTTTCTTTAGATTAGTTAATCTTTTTTGAAAGGTTAAAAGGGGTGGA

GTAAACCTGTTTTTATTTTCTTCGAAACTAGTGCCCTCTTCCTCCGCGTGTAGAAAAGGA

AGAAATAAATCAATCAAATTACGAGAAGCCTCATAAAGCGCTTCCTTAGGGGTTAAACTT

CCATTCGTCCATATTTCTAGAAAAAGTATCTCGTGTTTTTCATTTCCATTCCCACAAGAA

AAAATACTATAATTCACATTTCGAACAGGCATGGATACAGCATCTATGGGATAACTTCCA

TCTTGAGAGTTCTTTCTGAGTTCCGTGTGATATCCGCGATCTCTCTTGATCTGTAACTCA

ATACAGAAATCAATGGGCTCTGTCAAGTTAGCTATAGGTTGTGCCGTATCAACGATCTCT

ACGGAAGGTGGTAAGATGATATCTTGAGCAGTTATGTATCTAGGACCTTTGACGCAAATT

GATGCGTCTCTAACTCCATAGAGATTACTTCTCAATACAATTTCTTTCAAATTTAGTAAA

ATTTCTTGTACGGATTCTTCAATACCAGCTATTGTAGAATATTCGTGTGGCACGCTCCCA

AATTTTGCACGTGTGATACATGTTCCTTCTATTTCTCCAAGTAAAGCTCTTCGCAAGGCA

ATACCGACGGTATCCGCTTGACCTTTTCTAAGCGGGGACAAAATGAAACGACCATAATAA

AGACGCTTACTATCTACTCTTGATTCAACACACTTCCACTGTAGTGTTTGAGTGGATCCT

GCTACCTCCTCTCGAACCATATAGACTATATTATTTGATCATTGAATCGTTTATTTCTCT

TGAAAGCGTTTTAATTCTTTTACAGACGTCTTTTTTTAGGAGGTCGACATCCATTATGCG

GCATAGGTGTTACATCGCGTATACAACTTAATCGTACACCACTTTTAGCAATGGCTCGTA

ATGCGGCATCTCTTCCACTACCAGCACCCTTTACCATAACTTCTGCTCGTTGCAAACCCA

CTGTACGAATAGCATCTACTGCTGTTCTTTGACCAGCATAGGGTGATGCTTTTCTTGAGC

TTTTGAATCCACAAGTACCCGCGGAGGACCAGAAAACCACCCGACCTTGCGGGTCTGTAA

CAGTTATAATGGTATTGTTGAAACTAGCTTGAACATGAATAACTCCTTTTGTTATTCTAC

GTGCACTTTTCCGTAAACTAAAACGCGCATTCCTACGCAAACCAATACGCACTCTCCTAC

GTGAACCAATTTTTGGTATAGCTTTTGTCATATTTTATTATCTCATAAATATGAGTTAGA

AATAACAAAAAGAAAAAAGATACAAAGATATCCGTTTCAGGGTAAAATATATCCTTTACT

TTAATTATTTTATTTGGAATTTGGACGTTTCCGCGGGTACTTTTTACTTTTTAGAAAGTA

AAGTTCTTTTTCGAAAGATTACCCCTGCCTTTGTTTATGCTTCGGATTGGAACAAATGAC

TCTAATTCGTCCACGCCTACGAATCAGTCGACATTTTGTACAAATTTTACGAACGGAAGC

TCTTATTTTCATATTTCCTTATTCCTTTCTTAAACTATGAATCTAATCTTTGGAAAAAAT

AAGTCTCTTCGCTTGAATTTTGGAACTTTGAATTTATTACCCTAGAAAAACCTAATCCTT

TGAATCTTTGGTATCCTTCAAATCTTCGGTATCCTTCGAGTCTTCGGTACGCTTCGAATC

CTTATGGGGAAGTCTATAAATTATACGTCCTTTGCTTGAATCATAACGACTTACTTCAAT

TTTGACCCTATCCCCCATCAGTATTCGTATAGAACTAGACCGGATCTTTCCTGAAATATA

GCCCAGGATGATGGTGTCATTCTCTAGGCGAACGCGGAACATTCCGTTGGGTAGGGCTTC

CGTAACTAAACCTTCGAAAGTGACTTTTGCTTCTCTCGGGTTTTTTTTTTCTGTCATATT

TTTTTCTCCTATTTTTCTATTTTGATTTTCAAAATAGGAAGTTCGAGATAGAATTCGAGT

ACTATAGGAGGGGCGATTACCATATATAACATAAGACTTCTCCCCCAATTCTGTTTAGTC

GAGCTTCTCGATCTGTCATTATACCTCGAGAAGTAGAAAGAATAGCAATTCCCATTCCGC

CCAAAACTTTAGGAATTCCTTGATAGTTGGCATAAATTCGTAAGCCGGGTCGGCTGATAC

GCTTTAAAAAGGTTCTAGTTCTATATATTCCTTTTCTAGTCTTTCTCTTTTGATGTCGCA

AAGTTGAAACCAAGAAATATCTGTTACTTTCCTGATGTTTCCGAACACTTTCAATAAAAC

CCTCTCGTAGAAGTATTTTAACAATGTTTTCGGTAATATTTGTAGATACTACTCGAACTG

TTCCTTTTTTATTCATGTCCGCGTTTCTTATAGAGGTTAGTAAATCAGCAATAGTGTCCT

TGCCCATAAGACTCTAATTCTAGGTTCCTCCTAATTTTTCTATAATCAACATGTTTTCTT

TTTCTTTTACTTTTGGATTTTAAAGCATATACGTGAGACATAATCTACTAATTTTTTCTT

TGATCTATATCTCGCCTACTAGTATTTATAATACTTCAGGAGCTAATGAAACTATTTTAG

TAAAATTCAATTCTCTCAATTCCTCGGCGATCGCGCCAAAAACTCGAGTTCCTTTTGGAT

TTCCTTTTTGATCAATGATAACCGCTGCATTGTCATCATAGCGTATTATTATACCGTCTT

CGCATTTGAACTCTTTACATGTACGTACAATTACAGCTCGAATTACTTCGGATCTTTCTA

GAGGCATTTGGGGCACTGCGTCTTTGATTACAGCAACAATAACATCACCAATACGAGCAT

ATCGCTGATTACTAGCAGCTCCTATGACTCGAATACACATCAATTTTCGAGCTCCACTGT

TATCTGCTACATTTAAAAGGGTCTGAGGTTGAATCATATTATTTTGATTTCAATTTGTTA

TTTCTATGCAAAGGATGAAAGAAATATTGTCTTTCCAGAAAAAAACCTGGTTTTTTACTT

CAATACTCCTTTTTGGGATGCTATATCTCTAATCGAAGAAATTGACTTCGTATGGGCATT

TTACTGGCAGCTATGGAGATAGCTGCTCTAGCTACAGTTTCAGATACTCCGCCCATTTCA

TAAAGTATTCGACCTGGTTTAACAACGGCTACCCAATATTCGGGGGATCCCTTTCCTGAG

CCCATACGTGTTTCCGTGGGTCTTAGTGTAACCGGTTTGTCGGGAAATATACGTACCCAT

ATTTTTCCACCACGACGTGCATATCGTGTCATTGCTCTTCGTCCTGCTTCTATCTGTCTC

GACGTGATCCAAGCGGGTTCAAGTGCTTGCAGAGCATATCTACCAAAACAAATACGATTG

CCTCGGCAGGATTTTCCCTTCATTCTTCCTCTATGTTGTTTACGAAATCTGGTTCTTTTG

GGGTTATAGTCGATGGTTCTTTCTTAGTTCCATCTCTACTGCAAAACTGGACATGAGAGT

TTCTTCTCATCCAGCTCCTCGCGAATGAAATGAGAAGCGTGCAAATTTCTCTAATTCCAT

AATATTTTGGAATATTATGGATAGATGCACATTAATAGATAAAGTTAGGGTTTTTAATAT

TGAATTTGTTAAAATAGAAAAATATATAGTCAAGAAAGAAGATCTAGAATATATCTAGAT

TCTATATTTAATGTAATCTTTCTTAATCTCCTTATTTATTCAATAAAGATTTCGCGGGCG

AATATTTACTCTTTCCTGTCTTATTTGTTAATTTATAACCTTACCAAATAAGGCAATTTT

TTGGTTTGTCCGCCATCCACCAATGAAGTCTTAGGATTCTTTCAATAAATCCTATGCAGT

CATAGGTTCTGTCGTTCCCACTACTTCTCCTTTAATGGTTAGGTCTGAATCCCACAATGG

AGCTTTCAAAATTTCTTTCCGAGTCAATTTCTCAGTTTTATTAACCCGGCCGCTCTTTAT

TATTGCTTAAAATTTCTATTTTTTGTTTATTCTCTGTTATTTTTATTATATTGATGCTTT

ATCACATTGCCTTTTATGATGAACTCATAGACCATACATATTGGAATCCTATATCTTTCT

TATTCTTCTTCCTTCTTTCTATCATCCCTCCTTTTATCCACATCCCTTTAGTTTTGCTTC

ACAACCTAGAATCCTTTTCTTTTTTAGAGAAAAATTGCAGTTGCTACAACTATATGATAG

ATCTACTCATTTATGATAGATGTATCATATAGTGACTGTTTCTTAGTTAGGATCTCGACA

ATACGAAGCAATAGGTTGGTTATTAGTTAATTTTCTATAATTTTCCATTTTTGACTCTAA

AAAAAAACTAACAGTCACACACTAAGCATAGCAATTATATTAAAAGATTTATCAATTTTC

ATTAAATCTTATAGAAAGAGGTAGAATTCTTCTTTTTTTCAGGGATTTCAGGAAAAATAA

GGTCTTGTCTTTTTTATTCTATCACTGACAGAATGGGAAGACAAGGCTAGTTATTCTTCG

TCTACGAATATCCAAATTTTTACACCTAATACTCCATAGATAGTTCGAATTGGATAGCAG

CAATAATCAATTTTAGCGCGAATTGTTTGGAGGGGAAGTCTACCCTTTTTGATGCATTCG

GCACGTGCAATTTCTTTCCCTGCGAGACGACCTGCAATTTTTACTTTTACCCCCCTTATA

TCTGCTTTTTTAGTTAATTCAATGGCTTTTTTCATTGCCTTTCGGAATGAAACTCTATTT

TTTAATTGGAAAGCTATATATTCTGCAAGAATGTTAGGTTGTCTATAAGGTTCTTTAACT

TTTTCAATAGCAATATTAAGTCTCTGGTTTACAGAATTAACTTCCTTTTGTAGATCTTTC

TCTAATTCTTCGATTGCTCCTTTTTTCTTTAATAAATTGGGGAATCCAATATGGATTATG

ACGTGGATCGTATCGATTTCTTTTTGAATTTCTATACGTGTAATTACTTCAGAACTTGAG

CCTGAGTCCATTTTTCTATTCGAGCCTTTTTTCCTATTCTTTTGTATATAGTTCTTGATA

CAATTCCGTATTTTTTTATCTTCCTGTAGACCTTCAGAATAATTTTTTGGTTGTGCGAAC

CAAAAGGAATGGTGATTTTGGGTTGTACCAAGTCTGAAACCGAGTGGATTTATTTTTTGT

CCCATATTTTTCTATTCTATTTTTTTTACTGGGAATCAAATCTAAGATGGATCTAAAGAT

TATTTAGATTTCTTTACTATATTTAGTACAATTGTTATATGACACATGGTTTTTTTTATG

GGACAACTACGTCCTCGAGCTCGAGGTCTTAATTTTTTCATGATAGTACTCCTACTGACT

TCGGCTTTAGTGATGAATAAATTCGCTTTGTCGAAATCCCTATAATGAGTAGCATTTGCT

GCTGCCGAATAAACCAACTTTAGGATGGGATAAGATGCTCGATAAGGCATGAGGTTCAGT

ATCATAACAGTTTCCTCGTAGTAACGCCAGCGAATCTCATCAAGAACTCTTTGTGCTTTG

AAAACAGACATATGGATGCGTTTTTGTGCTTTGAAAACCCGTTCGAACTTAAGTAGACGA

TCGGTTGGAACTTTCCTTGCGAGTTTCCTTAGCCCCTTCTTCTTCTTTATCCTAGGGGTA

TACTTTACCAGTTTGAAACTTGTCATAAATAAGGTTATTCCCCGCCTACCTTTGTTTTTT

TTATTTTGAATCTTTCTATTCTGAATTCAGTTAACGACGAGATTTAGTATCTTTTCTTGC

ACTTTCATAACTCGTGAAATGCCGAGTAGGCACGAATTCCCCCAATTTGCGACCTACCAT

AGGATTTGTTATGTAAATAGGTATATGTTCCTTTCCATTATGAATCGCGATTGTATGGCC

AACCATTGCGGGTAGAATGCTAGATGCCCGGGACCACGTTACTATTGTTTCTTTCTCCTC

CTTCATATTGACCTTTTCGATTTTTGCCAATAAATGATGAGCTACAAAAGGATTCGTTTT

TTTTCGTGTCACAGCTGATTACTCCTTTTTCATTTTAAAGAGTGGCATTCTATGTCCAAT

ATCTCGATCGAAGTATGGAGGTCAGAATAAATAGAATAATGATGAATGGAAAAAAGAGAA

AATCCTTTAGCTGGATAAGGGGCGGATGTAGCCAAGTGGATCAAGGCAGTGGATTGTGAA

TCCACCATGCGCGGGTTCAATTCCCGTCGTTCGCCCATCGCATTATTGCAAATTCCAAAA

ATGCAATTTTCCATATTCCTAGTTACGTATTTACTTACGGCGACGAAGAATAAAACTATC

ACTATATTTTTTCCTTTTCCTAGTTCTTCTTCCAAGCGCAGGATAACCCCAAGGGGTTGT

GGGTTTTTTTCTACCAATGGGGGCTTTCCCTTCACCGCCCCCATGGGGGTGGTCCACAGG

GTTCATAACTACCCCTCTTACTACGGGGCGTTTACCTAGCCAACACTTAGATCCGGCTCT

ACCCAAACTTTTTTGGTTCACCCCAACATTACCCACTTGTCCGACTGTTGCTAAGCAATT

TTGGGATACCAAACGGACCTCCCCAGATGGTAATCTTAAAGTGGCCGATTTACCCTCTTT

TGCAATCAGTTTCGCTACAGCACCTGCTGCTCTAGCTAATTGCCCACCCCTTCCACGTGT

GATTTCTATGTTATGTATGGCCGTGCCTAAGGGCATATCGGTTGAAGTAGATTCTTCTTT

TCTCTCAAAAACCCCTTCCCAAACTGTACAAGCTTCTTCCAAAGCATACAGCTTTCTAGA

TGTATATGACGATCTCTAGACAGATGGATCTTATATGAATCGTATGATGAAGTACCACAT

GAGTGGATATATAGGAAAGGAATCCAAATCTGCCGAATCGCTCATGTTATGATCTTCTAC

ATCCTAGGTCTCCGCGTTCCGTCATCTGGCTTATGTTCTTCATGTAGCATTCAGATCGAA

TGACTCTATGAAATTACGTCGATACTTCCACATATTATGGGTAACGTAGGAGACATCCCT

ATTTTCCCCGGGGGGTCTTAATTACCACTGCTTAGCTTTCAATTCGCCTCTGACCATCAA

ATTAAATGTGAATAACCCGTCCTCCTCTCTTTGAAACAAGGGGCGCTTCCGGTTCTGTGC

GTGCTTCAAACAATTTTGTCTTCTCCATATTACCATATCTCTAGAGTCAATAATTTTCTA

TGAGGAACTACTGAACTCAATCACTTGCTGCCGTTACTCAACAGTTTTCTGTTGAGGTCT

ATCCCGTAGAGGTAGTCAAATTGGATCAGTGATCGATTTCTAGGTTTCGTCGTAAACCTA

ATTGGTTACTTCCAATTACGTAAATCAATAGTTCAAACCGCACTCAAAGGTAGGGCATTT

CCCATTGATATAGGAACTTTTGTACCAGAAACAATAGTATCTCCAATTATAGCCCCTCTG

GGATGTAAAATATATCTCTTCTCACCATCCCCATAGTGTATGAGACAAATGTATGCATTT

CGATTAGGGTCGTATTCTATGGTTACGATTCTACCAGATATGTCTTTTTGATTCCGTCGA

AAATCGATTTTACGGTATAGGCGCTTATGACCTCCCCCTCTATGCCTTGCGGTAATGATT

CCTCTGGAATTACGACCTTTACCACAACGGTGCCGTCCATGGATCAAATTATTTCGTGGA

TTGGATTTCACTTGCCTGTCTACGGTTCCCTTGCGTGTGCTCGGGATAGGTGTTTTGTAT

AAATGTTTCGCCGTATTATTAAGTATTCTCCTTTAGTTTTTTTCTCTATCTAGAAGTGGA

ATAGAATAACCCGGTTGAAGGGTAATGATCATACGTCTGTAATGCATTGTATGTCCCAGA

ATAGGTCCCATTCTTCTACCCTTTCCGGGTAGTCGATGGCTATTCACAGCTACTACCTTA

ACACCAAAGAAGAGTTCGACCCAATGCTTTATTTCTGTCTTAGTGAATCCCGATTCGACA

TTAAAAGTATATTGATTCTTTCCCAATAAACGAAGACTTTTTTCTGTAAATACTGCGTAT

TTGATTCCATCCATAAATCGACTTTCCCTCCTATGCTCTGAGTTCCAGTATCGATAAGAA

TTCGAGTTCTTATTGTTCTTATGTTATGGTATGAATATACCATACCAATTCGTTATGTAT

GGATGATGGATGAGATTCCATGGATAGAGAGCCAGTTCCAATAGACCGTTCCCGTTCGCG

TGCATCCAGCAGGAATTGAACCCGCAAATTTACCAATTATGAGTTGGGCGCTTTAACCAT

TCAGCCATGGATGCTTAACAGGGATCATCGTACATCGTAAATAACCAATTTTCATATAGA

AAGACATATCATAGAAAAATGAAATCGAAAATATTCGGAGATGGCAAATATTCGGAGATG

ACTATGAAACACCTCTCTGGATCCTCGAATTGAAAGAGAGATTGAGAGGGATCAAGAATC

CTAATTCTCGCTATTTGGAATGGATCCAATTCTATTGAGTCTGACTCATAGTGATCATTT

CTCTTTAGCAAAGAATGACCTTGGTTATCAAAGGATTGAACAACCGGGATCCATTTACTT

ATGATACCTAGTTGACATTGATAACAAGGATCTAATGAATTATGAGTTTAATAGATCCTC

TTTAGCAGAAAGACGTATATTCCTTGCTCATCACTTATCCCAAACTCGTGTGGGCTAATC

GTTTTCATTTACCATCTCATGGAAAACCCTTTTCGTTCCGCTTAGCCCTATCGGGTATTT

TAGTGATAGGTTCTATAGGAACTGGACGATCCTATTTGGTCAAATACCTAACGAAAAATT

CCTATTTTCCTTTCATTAAGGTACGAGGGCTTCTTATTCCACAAGAACGAAAGCACCTTT

TCATTCTTTCATATACTAGGGGTTTTTACTTGGAAAAGACAATGTTCCATACTAAAGGAT

TCGGGTCCATAACCACGAGTTCCAGTGCACTAGATCTTGTAGCACTTAGCAACGAGGCCC

TATGAATAGACATATAGAATTTTTGGTCGGGAAATTCGAATGAATCATTGAGTGAAAAAG

GAGCAAAGAATGACAAAAGACGAGACTCTACTAGTCTTCACTCTTGTGGTTTCCTCGGTT

TCTGTTTTCTTATTCGGGATCTTGCTTTTCATGGTTCTCATCTCTGCAACTCGCGATTTT

CGCGAGAGAACCAAATCCAAGTTGGTGAAGATCATGATTTGGGCTGGCATAGTAGTTATT

ACCTTTGCAATTGCGGTTCGAATCTATCCGATCTTTATCTTTTTGCTCAAAGAACGAATA

AAACCCCTTGTCGAAGCCCTTTATGATAAGCTTCCCTGGATCTGGGAAGTTTCTCTTTCA

CGGTATTGGGATCGTTTGATCGATTTCCTTGATCGCTACTTATGGGCGTGCGCTCAAAGG

ATACAAACAGGAATTCGCAAACAAAAAGGGGAATTCGTAGTCACTTTTTCCTGTCGCGTA

AAAAAAAGGCTTTACGCGAGAGCAATAGAGGTTGGGATACATCTATCTCTTCTGAGCAAC

CTCTTTTGGATTCTTAAGACCACCCTTGCAGTAGGATACCGTCTGCTTTGGGTTCTTTAT

TATCTCCTTCCAGGGATTTTTAGGATCGTTCAGGCTATATTTAGTCTATTTTGGCTTTTA

CTGTCTACTTTTCTCAGGGAGATGGTTAAGGACCTCAGAAGATAGAGGAGAGCGCCAGGC

GCAGATTTCCGGAATACTTCTACGGGGAATGCTCATTCATGAGCATTCTCCATATTATGC

CTTGAAGAGGACTCGAACCTCCACGCTCTTTAGCACGAGATTTTGAGTCTCGCGTGTCTA

CCATTTCACCATCAAGGCATCTTGAAAGTGAATCGTATTCCATGAATATGATATCTATCT

AATGTGATATATTCCATATATGACAAAGGTGGAGTCTTGGAGTATTTCGATCGATCGGTC

ATATAGGCCTGAGTCAGACATCAAATAGCTTCGATTTGCATTATCCGTAGAACACCTTAT

ATGTATCAAAATCGAAAAGATGTACAATCCAATTTCTCGATTCAATAGAAGCCCAAAGAG

GTGCATATGGTACCCAAATAAGGATAGGATAGATATGTCAAAAGCAGGTCTGATTACACC

TATTCCTAATCCTAAATAGAATGTAAGGGCGTAGGGATTTCTATGTAAACAGAGTATCCT

ATTTCCATAGGCTCGAATGACCCCTTCTCATAATAAGAATGTGCACGGTCTGGTCCGGTA

TGGAATGAACTTATAATCTGATGATCGAGTCGATTCCATGATTATAAGTTCATAACCCTA

GCGCCCATTCCCATTTTGGGCGGAACAGATCTACTAATTCTTTTATTCCAGTTAGTAAGA

GGGATCTTGAACTAAGAAATAGACCTAGCAGCTAAAAGAGGGTATCCTGAGCAATTGCAA

GAATGGGGTTCATTGATATTCCTGGTATAGTAGATGCTATCACACATACAGTCATACTCA

ATTCGATGGAATTGTTTGATCTTAAAGGGGATCTTCTATAATTTCGCACATAAGGGGTTA

TTTCTTGGTTTCGTCCAGTCATTAATAACTTGATTATTTTTAGATAATAGTAGATAGAAA

GAACGCTCGTAAGGAGTCCTATTGAAACCAAGAAATATAGGCCTGCTTGCCATCCACACC

AGAATAGATAGAGTTTTCCGAAGAAACCCGCTAGTGGAGGAAGGCCTCCTAGGGATAAGA

GACATAGGGCTAAAGAGAGAGCCAAAAAAGGATCTTTCGTGTATAATCCTGCATAATCTC

GAATGTTATCAGTTCCGGTACGTAGACCAAATAATACAATGCAAGCAAAAGTTCCTAGAT

TCATGGAGATATAGAACAGCATATAAGTTATCATGCTTGCATATCCATCATTTGAGTCTC

CAACAATTATTCCAATAATTACATATCCGATTTGCCCTATGGACGAATATGCAAGCATAC

GTTTCATGCTTGTTTGAGTAATAGCAAGGAGATTCCCCAATATCATGCTAAGAATAGCTA

GGATTTCCAGAAGAAGATGCCATTCGTTTGATGAGAAATAAAAAGGAATATCGAGAATTC

GCGTGGCTGAAGCTGAAGCAGCTACTTTCGAAGTAACAGAAAGAAAAGCAACGACTGGAG

TGGGGGAGTCAGAGTCGAAAAGAGGATTCCTCGCTTCTTTCTCTCATGCAAAACCGTGCA

TGAGACTTTCATCTCGCACGGCTCCTAAGTGATAAAAGAAAGAAGAACTCGTCTTCTTTC

TTTTTTGATTACCTTCCTCGCGTATGTATAAGACCCAATCCATTCTTTTTCGAAATCGAT

TTCGAAAAAGAACTACTAATCCTTAACTTTTCGAGGAATCCTTCATCAGTGGTTGTGAAT

GACTGACTTTTTCAATCCTTTCGACTTTGGTTCCGTAGGAGCAAGTCAGAAAGGTTGAGA

AATAGAACCATCTGATTTGATTCGTTCCCAATAGCCATGAGATGATTATCTTAGGGTGAT

CCTTTTGTCAACGGATGCTCCTATTACACTCGTAGTCTCTGAAGGATGAGAACCCACTAT

GTAGCATCTACATTGATAATTCAAGCATTGTATACGTCATTAGTCCGATTCTTTGTAGGA

ACTACCCGTAATAACGAGCTTGCAAAATGGATCTGTTTATCATAAAGAGATTCGTTGTTC

CTGACCCTGCTTCACCTTAATTGTTATTTGAACAAAAAGATCACAATAAACTTTTGGTAA

AAGTTCTGTCTTGGTCGGAGTGGGGATAGCATTTCTCTTCTGCATGTCTATGGAGTTTTG

CAAAACCCAAACACCTCAGATAGAGGTAGGAATTTGTCGAACGAACCACACTCCTTCGTA

GACGTCAGGAGTCCATTGATGAAAAGGGGCTGGGGAAAGCTTGAACCCAAGTCCTACAGT

GATGGATATAAGCGCAATTGAAATTCCTGGGGAGTTATACATTTGTGTATTGATAAGACC

GTTCACAATTTCTTGAAGCTCGATCTCCCCCCCAGATGAACCATATAGCCAAGAGAAACC

ATGAACCAGAATAGAAGAGCTTGCCCCACCCATGAGTAAATATTTCATAGTAGCCTCATT

AGACCGTAGATCTCTCTTGGTATATCCAGACAATAGGTAGGAACATAAACTGAAACATTC

TGGAGCTACAAAGATAGTTATTAAATCGTTAGCACCACATAAAAACATTCCCCCTAGAGT

AGCTGTTAATACGAATAACAGAAACTCTGTTATAGCCATTTCTGTACATTCAATGTACTC

TACGGATAGAGGAATACATAAAGTTGAACATAATAAAATAAGAAATTGAAAGATTTCGTT

GAAATTGTTCGTTTGGAAATTTCCCGAAAAGCTAATTATAGGTTCTTCTCTCCATCGGAA

CAATAGGGCCGTTATGCTTATTACTAAACTTGTTGAAGAGATGAAATAGAACCAAGGTCT

ATCTTTTTGATCAGAGGTTGAATCGATCATCAGAAGAAGAATTAGGCCAAAAATTAGGAT

ACATTCTGGGAAAATGAAACTTCCATGGAAGAGAAGCAAATGAAACGCTTTCATAAAAAT

TCTCGTAGAATCGAGAATGAAGTTTTCATTCTGTACATGCCAGATCATGAATTAGTAACT

GCATCCAATCTCCGAAAAGTCCCGATTGTTTCGATTTTTGGAATGGGATATTTACGGAAT

CCCCATGAATAGGATCAAACCTTATTCCATGCTATTTCCATAAGATTCCTCTTTCTTATT

CTTAAGCAAGCCCCCGAGAGGGCTTAGTTGATCATGATTTCTGTTTTCTCTTTCTTTTCC

TTTTTGTTTGTTTCGAGAAAGATATCGTCCGATTCTCCTTCTATTGATTCTTTTCCGATC

GAGATGTATGGATCCATGTGTCTACATACCTAGATTCTGTTCATGGATTAACGAAAATGT

GCAAGAGCTCTATTTGCCTCTGCCATTCTATGAGTCGCTTCCTTTTTGCGTATGGCACCC

CCACTCCCTTTGGCAGCATCTACTAATTCGGAACTTAATTTGAAAGCCATATTTCGACCC

GGACGCTTTTGGGATGCTTCTAATAACCAACGAATGGCAAGTGCTCTTCCTTGTTTAGAT

CCTATTTCAATCGGAACTTTCCGCGTCGATCCTTTTTTATTACGTCTTGTTTTTACTCCT

ATATTGGGAGTTACTCTACGTATTGCTTGACGTAAAACCAATAGTGGATTTGTTTCTGTC

TTTTGTTGAATCTTTTTCACGGCTCGATAGAGAATTTGATAAGCCAATGATTTTTTTCCG

TCTTTCATAATACGGTTAACCACCATGTTAACTAATCGATTACGAAAAATTGGATCGGAT

TTTGCAGTTCTTTTTTCTGCAGTACCTCGACGTGACATGAGCGTGAAAGAGGTTCAAGAA

TCCGTTTTCTTTTTATAAGGGCTAAAATCACTTATTTTTTTGGCTTTTTGACCCCATATT

GTAGGGTGGATCTCGAAAGATAGGAAAGATCTCCCTCCAAGCCGTACATACGACTTTCAT

CGAATACGGCTTTCCACAGAATTCTATAGGGATCTATGAGATCGAGTATGGAATTCTGTT

TACTCACTTTAAATTGAGTATCCGTTTCCCTCCTTTCCCCCTAGGATCGGAAATCCTGTA

TTTTCCATATCCATACGATCGAGTCCTTAGGTTTCCGAAATAGTGTAATGGAAAAAGAAG

TGCTTCGAATCATTGCTATTTGACTCGGACCTGTTCTGAAAAAGTCGAGGTATTTCGAAT

TGTTTGTTGACACGGACAAAGTAAGGGAAAACCTCTGAAAGAATTTCCATATTGACCTTG

GACATATAAGAGTTCCGAATCGAATCTCTTTAGAAAGAAGATCTTTTGTCTCATGGTAGC

CTGCTCCAGTCCCCTTACGAAACTTTCGTTATTGGGTTAGCCATACACTTCACATGTTTC

TAGCGATTCACATGGCATCATCAAATGATACAAGTCTTGGATAAGAATCTACAACGCACT

AGAACGCCCTTGTTGACGATTCTTTACTGCGACAGCATCTAGGGTTCCTCGAATAATGCG

ATATCTCACACCGGGTAAATCCTTAACCCTTCCTCCTCTTACTAATACTACAGAATGTTC

TTGTAAATTATGGCCAATACCTGGTATATAAGCAGTGATTTCAAATCCAGAGGTTAATCG

TACTCTGGCAACTTTACGTAAGGCAGAGTTGGGTTTTTTGGGGTTGATAGTGGAAAAGTC

GACAGATAAGTCACCCTTACTGTCCCTCTACAGAACCGTACATGAGATTTTCACCTCATA

CGGCTCCTCGTTCAATTCTTTCGAAGGGATCCTTTTCCTCGTTCGAGAGTCTCCGCCCTT

CTTCCACTCCGTCCCGAAGACTAACTAAGACCAATGGAGTCACGTTTTCATGTTCTAATT

GAACACTTTCCATTTATGATTAAAGGAGAAGATTGTTCTTTTACCAAACATATGCGGATC

AAATCACGTCTTATAATAAGAAGAAATCTTTCTCGGTATCAATCCCCTTGCCCCTCATTC

TTTGAGAATCAGAAGGATCCTTTTCGAGTTTCCATTTCTTCATTTGGAATCTGGGCTCTT

CTATCTTCGACTTATTTTTTGGCTTTATTCTTTATTTATTTCATTTCGATTTTTCCCTCT

TCCTCTATCCCTATCCTCTAGGTACAGCGTTTGCATCAATAGAGAACCTTTTCCTCTGTA

TGAATCGATATTATTCCAATTTCTTCCCGAAACTTCCCAAGAAAAATCCCGAATTGGATC

CAAAATTGACGGGTTAATGTGAGCTTATCCATGCGGTTAGGCACTCTTCAAATAGGAATC

CATTTTCTAACTGGCTTTCGTGCTTTGGTGAGTCGTCCGAGATCCTTTCGATGACCTATG

TTGTGTTGAGGGGATATCTATATGATCCGATCGATTGCATAAGACCCGCGGTAGCATAGA

ACGGGGAAAGTATACAGAAAAGACAGTTCTTTTCAATTTCGATTATCTATATATTAGTTC

GTTTCTATTTCTAGATATCTATTTCTATATATTAGTATTAGTTAGTAGTACTATTCTATT

AGTTAGCGATCCCGGCTCTGTGAGTTCTTTCTTCCGTGATGAACTGTCGGCACCAGTCCT

ACATTTTTTCTCTGTGGACCGAGGAGAAAGGGGGTTAGCAGGATTACCATGAGAGAACAA

CCCGCTTCAAATATGGAACATGGATTCTGGCAATGCAACGGAGTTGGGTCCTCATATCGA

TCCGAATGAATCAGTCTTTCTACAGAGGTCAATCTTTCTCGATAGGACATGGATTTCTAT

TCCTATGAAATTCATAAATTAGTTAATGGAGGGCTACCATTCCTTTTTCTTTATGTGTTC

CTAAGAGAAGGAATTTGTCCATTTCATGTTTCGAGGTCTCAAAAAAGGGCGTGGAAACAG

ATAGAAACTCTTGAATGGAAATTGAAAAGAAATGTAGCCCCAGTTCCTTCGGAAATGGTA

AGATCTTTGGCGCAAGAAGAAGGGGCGACCCGTATCATCTTGACTTGGTTCTGCTTCCCC

TCTTTTTTTAAGAATACCGAGTCGGGTTCTTCTCCTACCAGTATCGAATAGAACATGCTG

AACAAGATCTTCTTCATGGAAACCTGCTCGATTTAGATCGGGAAAATCGTACAGATTTTA

TGAAACCATGTGCTATGGCTCGAATCCATAGTCAATCCTACTTTCGATAGGACCGGTTGA

CAATTGAATCCAATTTTTCCCATTATTTGACTATCCATAATAGTGCGGAAAGAAAGCCCG

GAGGAAGAGTGGCCTTGAGTTTCTCGCCCCTTTGCCTTAGGATTCGTTAATTCTCTTTCT

CGATGGGACGGGGAAGGGATATAACTCAGCGGTAGAGTGTCACCTTGACGTGGTGGAAGT

CATCAGTTCGAGCCTGATTATCCCTAAACCCAATGTGAGTTTTTTCTATTTTGACTTACT

CCCCCGCCACGATCGAACGGGAATGTATAAGAGGCTTGTGGGATTGACGTGATAGGGTAG

GGTTGGCTATACTGCTGGTGGCGAACTCCAGGCTAATAATCTGAAGCGCATGGATACAAG

TTATCCTTGGAAGGAAAGACAATTCCGAATCCGCTTTGTCTACGAATAAGGAAGCTATAA

GTAATGCAACTATGAATCTCATGGAGAGTTCGATCCTGGCTCAGGATGAACGCTGGCGGC

ATGCTTAACACATGCAAGTCGAACGGGAAGTGGTGTTTCCAGTGGCGAACGGGTGAGTAA

CGCGTAAGAACCTGCCCTTGGGAGGGGAACAACAACTGGAAACGGTTGCTAATACCCCGT

AGGCTGAGGAGCAAAAGGAGAAATCCGCCCAAGGAGGGGCTCGCGTCTGATTAGCTAGTT

GGTGAGGCAATAGCTTACCAAGGCGATGATCAGTAGCTGGTCCGAGAGGATGATCAGCCA

CACTGGGACTGAGACACGGCCCAGACTCCTACGGGAGGCAGCAGTGGGGAATTTTCCGCA

ATGGGCGAAAGCCTGACGGAGCAATGCCGCGTGGAGGTGGAAGGCCTACGGGTCGTCAAC

TTCTTTTCTCGGAGAAGAAACAATGACGGTATCTGAGGAATAAGCATCGGCTAACTCTGT

GCCAGCAGCCGCGGTAAGACAGAGGATGCAAGCGTTATCCGGAATGATTGGGCGTAAAGC

GTCTGTAGGTGGCTTTTCAAGTCCGCCGTCAAATCCCAGGGCTCAACCCTGGACAGGCGG

TGGAAACTACCAAGCTGGAGTACGGTAGGGGCAGAGGGAATTTCCGGTGGAGCGGTGAAA

TGCATTGAGATCGGAAAGAACACCAACGGCGAAAGCACTCTGCTGGGCCGACACTGACAC

TGAGAGACGAAAGCTAGGGGAGCAAATGGGATTAGAGACCCCAGTAGTCCTAGCCGTAAA

CGATGGATACTAGGTGCTGTGCGACTCGACCCGTGCAGTGCTGTAGCTAACGCGTTAAGT

ATCCCGCCTGGGGAGTACGTTCGCAAGAATGAAACTCAAAGGAATTGACGGGGGCCCGCA

CAAGCGGTGGAGCATGTGGTTTAATTCGATGCAAAGCGAAGAACCTTACCAGGGCTTGAC

ATGCCGCGAATCCTCTTGAAAGAGAGGGGTGCCCTCGGGAACGCGGACACAGGTGGTGCA

TGGCTGTCGTCAGCTCGTGCCGTAAGGTGTTGGGTTAAGTCTCGCAACGAGCGCAACCCT

CGTGTTTAGTTGCCACTATGAGTTTGGAACCCTGAACAGACCGCCGGTGTTAAGCCGGAG

GAAGGAGAGGATGAGGCCAAGTCATCATGCCCCTTATGCCCTGGGCGACACACGTGCTAC

AATGGGCGGGACAAAGGGTCGCGATCTCGCGAGGGTGAGCTAACTCCAAAAACCCGTCCT

CAGTTCGGATTGCAGGCTGCAACTCGCCTGCATGAAGCAGGAATCGCTAGTAATCGCCGG

TCAGCCATACGGCGGTGAATCCGTTCCCGGGCCTTGTACACACCGCCCGTCACACTATAG

GAGCTGGCCATGTTTGAAGTCATTACCCTTAACCGTAAGGAGGGGGATGCCTAAGGCTAG

GCTTGCGACTGGAGTGAAGTCGTAACAAGGTAGCCGTACTGGAAGGTGCGGCTGGATCAC

CTCCTTTTCAGGGAGAGCTAATGCTTATGCTTATTGGGTATTTTGGTTTGACACTGCTTC

ACGCCCAAAAAGAAGGCAGCTACGTCTGAGCTAAACTTGGATATGGAAGTCTTCTTTCGT

TTAGGGTGAAGTAAGACCAAGCTCATGAGCTTATTATCCTAGGTCGGAACAAATTAGTTG

ATAGTGATAGGATCCCCTTTTTGACGTCCCCATGTCCCCCCGTATGGCGGCATGGGGATG

TCAAAAGGAAAGGGATGGAGTTTTTCTCGCTTTTGGCGTAGCGGCCTCCCTTTGGGAGGC

CGCGCGACGGGCTATTAGCTCAGTGGTAGAGCGCGCCCCTGATAATTCGTCGTTGTGCCT

GGGCTGTGAGGGCTCTCAGCCACATGGATAGTTCAATGTGCTCATCAGCGCCTGACCCGA

AGATGTGGATCATCCAAGGCACATTAGCATGGCGTACTCCTCCTGTTTGAATCGGAGTTT

GAAACCAAACAAACTTCTCCTCAGGAGGATAGATGGGGCGATTCAGGTGAGATCCCATGT

AGATCTAACTTTCTATTCACTCGTGGGATCCGGGCGGTCCGGGGGGGGCACCACGGCTCC

TCTCTTCTCGAGAATCCATACATCCCTTATCAGTGTATGGAGAGCTATCTCTCGAGCACA

GGTTGAGGTTCGTCCTCAATGGGAAAATGGAGCACCTAACAACGCATCTTCACAGACCAA

GAACTACGAGATCACCCTTTCATTCTGGGGTGACGGAGGGATCGTACCATTCGAGCCTTT

TTTTCATGCTTTTCCCGGCGGTCTGGAGAAAGCAGCAATCAATAGGACTTCCCTAATCCT

CCCTTCCTGAAAGAAGAACGTGAAATTCTTTTTCCTTTCCGCAGGGACCAGGAGATTGGA

TCTAGCCATAAGAGGAATGCTTGGTATAAATAAGCCGCTTATTGGTCTTCGACCCCCTAT

ACGAGCGCCCCCGATCAGTGCAATGGGATGTGGCTATTTATCTATCTCTTGACTCGAAAT

GGGAGCAGAGCAGGTTTGAAAAAGGATCTTAGAGTGTCTAGGGTTGGGCCAGGAGGGTCT

CTTAACGCCTTCCTTTTTCTGCCCATCAGAGTTATTTCCCAAGGACTTGCCATGGTAAGG

GGGAGAAGGGGAAGAAGCACACTTGAAGAGCGCAGTACAACGGGGAGTTGTATGCTGCGT

TCGGGAAGGATGAATCGCTCCCGAAAAGGAGTCTATTGATTCTCTCCCAATTGGTTGGAT

CGTAGGGGCGATGATTTACTTCACGGGCGAGGTCTCTGGTTCAAGTCCAGGATGGCCCAG

CTGCGCAGGGAAAAGAATAGAAGAAGCATCTGACTCTTTCATGCATACTCCACTTGGCTC

GGGGGGATATAGCTCAGTTGGTAGAGCTCCGCTCTTGCAATTGGGTCGTTGCGATTACGG

GTTGGCTGTCTAATTGTCCAGGCGGTAATGATAGTATCTTGTACCTGAACCGGTGGCTCA

CTTTTTCTAAGTAATGGGGAAGAGGACTGAAACATGCCACTGAAAGACTCTACTGAGACA

AAAAGATGGGCTGTCAAAAAGGTAGAGGAGGTAGGATGGGCAGTTGGTCAGATCTAGTAT

GGATCGTACATGGACGATAGTTGGAGTCGGCGGCTCTCCTAGGCTTCCCTCATCTGGGAT

CCCTGGGGAAGAGGATCAAGTTGGCCCTTGCGAATAGCTTGATGCACTATCTCCCTTCAA

CCCTTTGAGCGAAATGTGGCAAAAGGAAGGAAAATCCATGGACCGACCCCATTGTCTCCA

CCCCGTAGGAACTACGAGATCACCCCAAGGACGCCTTCGGCGGGGGTCACGGACCGACCA

TAGACCCTGTTCAATAAGTGGAACACATTAGCCGTCCGCTCTCCGGTTGGGCAGTAAGGG

TCGGAGAAGGGCAATCACTCGTTCTTAAAACCAGCATTCTTAAGTTAAGATCAAAGAGTC

GGGCGGAAAAAGGGGAGAGCTCCCCGTTCCTGGTTCTCCTGTAGCTGGATTCCCCGGAAC

CACAAGAATCCTTAGAATGGGATTCCAACTCAGCACCTTTTGTTTTGAGATTTTGAGAAG

AGTTGCTCTTTGGAGAGCACAGTACGATGAAAGTTGTAAGCTGTGTTCGGGGGGGAGTTA

TTGTCTATCGTTGGCCTCTATGGTAGAACCCGTCGGGGAGGCCTGAGAGGCGGTGGTTTA

CCCTGTGGCGGATGTCAGCGGTTCGAGTCCGCTTATCTCCAGCCCGTGAACTTAGCGGAT

ACTATGATAGCACCGAATTTTGCCAATTCGGCAGTTCGATCTATGATTTCGCATTCATGG

ACGTTGATAAGATCCTTCCATTTAGTAGCACCTTAGGATGGCATAGCCTTAACGTTAATG

GCGAGGTTCAAAAGAGGAAAGGCTTGCGGTGGATACCTAGGCACCCAGAGACGAGGAAGG

GCGTAGCAAGCGACGAAATGCTTCGGGGAGTTGAAAATAAGCATAGATCCGGAGATTCCC

AAATAGGTCAACCTTTTAAACTGCCTGCTGAATCCATGAGCAGGCAAGAGACAACCTGGC

GAACTGAAACATCTTAGTAGCCAGAGGAAAAGAAAGCAAAAGCGATTCCCGTAGTAGCGG

CGAGCGAAATGGGAGCAGCCTAAACCGTGAAAACGGGGTTGTGGGAGAGCAATACAAGCG

TTGTGCTGCTAGGCGAAGCGGTTGAGTGCCGCACCCTAGATGGCTAAAGTCCAGTAGCCG

AAAGCATCACTGCTTACGCTCTGACCCGAGTAGCATGGGGCACGTGGAATCCCGTGTGAA

TCAGCAAGGACCACCTTGCAAGGCTAAATACTCCTGGGTGACCGATAGCGAAGTAGTACC

GTGAGGGAAAGGTGAAAAGAACCCCCAGTGGGTAGTGAAATAGAACGTGAAACCGTGCTG

AGCTCCCAAGCAGTGGGAGGGGAAAGTGATCTCTGACCGCGTGCCTGTTGAAGAATGAGC

CGGCGACTCATAGGCAGTGGCTTGGTTAAGGGAACGGAACCCACCGGAGCCGTAGCGAAA

GCGAGTCTTCATAGGGCGATTGTCACTGCTTATGGACCCGAACCTGGGTGATCTATCCAT

GACCAGGATGAAGCTTGGATGAAACTAAGCAGAGGTCCGAACCGACTGATGTTGAAGAAT

CAGCGGATGAGTTGTGGTTAGGGGTGAAATGCCACTCGAACCCAGAGCTAGCTGGTTCTC

CCCGAAATGCGTTGAGGCGCAGCAGTTGACTGGACATCTAGGGGTAAAGCACTGTTTCGG

TGCGGCTGCGCGAGCGGTACCAAATCGAGGCAAACTCTGAATACTAGATATGACCCAAAA

ATAACAGGGGTCAAGGTCGGCCAGTGAGACGATGGGGGATAAGCTTCATCGTCGAGAGGG

AAACAGCCCGGATCACCAGCTAAGGCCCCTAAATGACCGCTCAGTGATAAAGGAGGTGGG

GGTGCAAAGACAGCCAGGAGGTTTGCCTAGAAGCAGCCACCCTTTAAAGAGTGCGTAATA

GCTCACTGATCGGCGCCCTTGCGCTGAAGATGAACGGGGCTAAGCGATCTGCCGAAGCTG

TGGGATGTCAAAATGCATCGGTAGGGGAGCGTTCCGCCTTAGATGGAAGCAAACGCGAAA

GCGGGGGTCGACGAAGCGGAAGCGAGAATGTCGGCTTGAGTAACGAAAACATTGGTGAGA

ATCCAATGCCCCGAAAACCCAAGGTTTCCTCCGCAAGGTTCGTCCACGGAGGGTGAGTCA

GGGCCTAAGATCAGGCCGAAAGGCGTAGTCGATGGACAACAGGTCAATATTCCTGTACTA

CCCCTTGTTGGTACGGAGGGACGGAGGAGGCTAGGTTAGCCGAAAGATGGTTATAGGTTT

AAGGACACAAGGTGACCCTGCTTTTTCAGGGTAAGAAGGGGTAGAGAAAATGCCTCGAGC

CGAGGTCCGAGTACCAAGCGCTGCAGCGCTGAAGTATGAGCCCCGTGGACTAGCCATTGC

TTCTCCACGAGCCTCATACCAGGCGCTACGGCGCGAAGTATGTAACCCATGCCATACTCC

CAGGAAAAGCTCGAACGACCTTCAACAAAAGGGTACCTGTACCCGAAACCGACACAGGTG

GGTAGGTAGAGAATACCTAGGGGCGCGAGACAACTCTCTCTAAGGAACTCGGCAAAATAG

CCCCGTAACTTCGGGAGAAGGGGTGCCCCCTCGCAAAAGGGGGTCGCAGTGACCAGGCCC

GGGCGACTGTTTACCAAAAACACAGGTCTCCGCAAAGTCGTAAGACCATGTATGGGGGCT

GACGCCTGCCCAGTGCCGGAAGGTCAAGGAAGTTGGTGAACTGATGACAGGGAAGCCGGC

GACCGAAGCCCCGGTGAACGGCGGCCGTAACTATAACGGTCCTAAGGTAGCGAAATTCCT

TGTCGGGTAAGTTCCGACCCGCACGAAAGGCGTAACGATCTGGGCACTGTCTCGGAGAGA

GGCTCGGTGAAATAGACATGTCTGTGAAGATGCGGACTACCTGCACCTGGACAGAAAGAC

CCTATGAAGCTTTACTGTTCCCTGGGATTGGCTTTGGGCCTTTCCTGCGCAGCTTAGGTG

GAAGGCGAAGAAGGCCCCCTTCCGGGGGGGCCGAGCCATCAGTGAGATACCACTCTGGAA

GAGCTCGGATTCTAACCTTGTGTCAGACCCGCGGGCCAAGGGACAGTCTCAGGTAGACAG

TTTCTATGGGGCGTAGGCCTCCCAAAAGGTAACGGAGGCGTGCAAAGGTTTCCTCGGGCC

AGACGGACATTGGTCCTCGAGTGCAAAGGCAGAAGGGAGCTTGACTGCAAGACTCACCCG

TCGAGCAGAGACGAAAGTCGGCCTTAGTGATCCGACGGTGCCGAGTGGAAGGGCCGTCGC

TCAACGGATAAAAGTTACTCTAGGGATAACAGGCTGATCTTCCCCAAGAGTCCACATCGA

CGGGAAGGTTTGGCACCTCGATGTCGGCTCTTCGCCACCTGGAGCTGTAGGTGGTTCCAA

GGGTTGGGCTGTTCGCCCATTAATGCGGTACGTGAGCTGGGTTCAGAACGTCGTGAGACG

TTCGGTCCATATCCGGTGTGGGCGTTAGAGCATTGAGAGGACCTTTCCCTAGTACGAGAG

GACCGGGAAGGACGCACCTCTGGTGTACCAGTTATCGTGCCTACGGTAAACGCTGGGTAG

CCAAGTGCGGAGAGGATAACTGCTGAAAGCATATAAGTAGTAAGCCCACCCCAAGATGAG

TGCTCTCTCCTCCGACTTCCCTAGAGCCTCCGGTAGCACAGCCGAGACAGCGACGGGTTC

TCCACCCATACGGGGATGGAGCGACAGAAGTATGGAAATAGGATAAGGTAGCGGCGAGAC

GAGCCGTTTAAATAGGTGTCAAGTGGAAGTGCAGTGATGTATGCAGCTGAGGCATCCTAA

CGAACGAACGATTTGAACCTTGTTCCTACACGACCTGATCAAATTGATCAGGCACTTGCC

ATCTATCTTCATTGTTCAACTCTTTGATGAAAAGATGAAAAACCAAAAAAAGCTCTGCCC

TTCCATCTCTTGGATAGATAGAGAGGGAGGGCAGAGGCCTTTGGTGTCCCTTCCAGTCAA

GAATTGGGGCTTCACAATTACTAGCCAATATTTATCTCATGCCTTTCCTCGTTCATGGTT

CGATATTCTGGTGTCCTAGGCGTAGAGGAACCACACCAATCCATCCCGAATTTGGTGGTT

AAACTCTACTGCGGTGACGATACTGTAGGGGAGGTCCTGCGGCAAAATAGCTCGATGCCA

GAATGATAAAAAGCTTAACACCTCTTATTTGACTTTTTCACTATTTTGAAATACGAAAAA

GATCCAAATCCAAAATGCAAAGGTCGTCTTATTCAAAACCTCAATCATCCCCTCCTCCCA

CTTCACACCTCGGAACGCACTGTTCTTATAGAGAGAAAGGCGCTTTCCCATCTTCTTAAC

CCGAAATGAAATGGCTGAGGAGAGGAAGGTTCCTTTTGGGGGGTACCCCCGGGAAGAGAT

CCAGTGGAGACGGGGTGGGCCTGTAGCTCAGAGGATTAGAGCACGTGGCTACGAACCACG

GTGTCGGGGGTTCGAATCCCTCCTCGCCCACAGCCTTCCAAAGGGGAAGGGCCTTTACTT

TCCCCCTGAGGGTAGGAAAATCATGATCGGGATAGCGGACGTAAAGCTATTGAACTTGGG

TATGCTCTTTCCTTTTGTCGAAGTGGAATCGTAGAACAGAATGTGATACGATGAGATAGA

ATGCATAGAAACAAGGATAGCGAACGGGTTACCTACTCCTAAGGGTCAAAGCAAGCCCTT

TAATTCAATTCTTTATTCTTACATTAAAGAATGAATCAAATCTCCCCAAGTAGGATTCGA

ACCTACGACCAGTCAGTTAACAGCCGACCGCTCTACCACTGAGCTACTGAGGAACAAGGG

GGATTCGACCTCCTAGAGTTCAACTCCCGCTCTCAACCCATGAACAATATGAGTCCGAAG

CTTCTTTCGTAACTCCCGGAATTTCTTCGTAGTGACTCCGTTCCATGCCTCATTTCATAG

GGAAGCCCAAAGTGGCTCTATTTCATTCTATTTCACTTCCTAGCACTTCCTATCATTTAA

TATCCATCCCTTTGGTCTTATTTACATAAGAGATGTCATTTATAGTCTATCTCTTTCTAT

ATATGGAAAGTCAAGAAATTCTCATCGAAACATCGAGAAATTGTGCATATAGAAAACTCT

AAAGAAAGAAAAAAGGAGACCCATGCCATGATTTTCAAATCTTTTCTACTTAGTAGTCTA

AGTTTCTCGATGAGGATAATTAATTCGGTCGTTGTGGTCGGACTCTATTATGGATTTCTG

ACCACATTCTCCATAGGTCCCTCTTAGATCTTCTTTCTCCAATCTTGGATTAGGGAAGAA

GGAGATATTCGCGACTACTGGCGGTTTCATTATGGGGCAGCTCATGATCTTCATATCGAT

CTATTATCCACCTCTGCATCTATTCTTTCTTAGCTAAACGGGTGGAAGATCCATCCAATT

TGGTTATATCATGGACTCAAAAGCGGATCTGAATGTGACTGAAATGCACGATCTTCACAG

GTATCACTTTTCACGATACCTAAAGATGGAATAGCGATTTGAACCATTTCCTATACGAGA

ATGGTTTCCATTACTTTGAGAAATGGATTCTATATCAAACTATAGCTATTGCATTAAAGA

AGAAAAGAAACTAATAGAAGTCGAAGACGCGGAATGGTAGTGAATAGAGAGAAAGATTCT

TCTGATTTTCTTGTTCCTGAAAATATTCTATCTATCTCCTAGACGCCGTAGAGAATTGAG

AATTTTCATGTCTTTCAATTCTCGTACTCGTAATTGGAAAGTTACGGAAGGAGGTCCATC

ATTTTGCAATGAAAACAACATAAAAAACTCTGGACAATTTCGAAATCAGGCCAAGCGTCT

TAATACATATGCAAAAAAATTCATTATTGGCCCACCATTGATTAGAAGATTTAGCTTGTA

TGAATCGCTATTGGTTTGATACGAATAATGGCAGTCGTTTCAGTATGTTAAGGATACAGA

TGTATCCACAATTCATTTAGAGTTACTTAATAGCCTATTTCTTATACCATATCTCTATCC

CGTGAAATTCTCGAGCCGAAAGATGGATGCATATGCTGTGTTTCATTTTGCTAAACGATA

TCAATTAAATGGTGTATCAATTCCATAAATTGGATATAGCAATAAATAAATCAGCAAAAT

TCTTTTATTTTAGATAGAAGAAATGTTTCTTCTATCTAAAATAAAAGAATGTACCCTTCT

ATCCAAATCCAATTTGCATCGATAAAATAAATCCAAATTCCAGTAGTGGATGAATAATTG

CAAATTTTTGTGTGTACGAGATTAGAATAACTTCAAAATAACTGACATAATTTTTTATTT

TTCCTGATCAGAAAAATACATGAAAAAGAAAGGAGGTAGAAAAATTTTGGGATTTATGGT

TAAAGAAGAAAAAGAAGAAAACAGGGGTTCTGTTGAATTTCAAGTATTCAGTTTCACCAA

TAAGATACGGAGACTTGCTTCACATTTGGAATTACACAAAAAAGATTTTTCATCGGAAAG

AGGTCTACGAAGACTTTTGGGAAAACGTCAACGTTTGCTGGCTTATTTGGCAAAGAAAAA

TAGAGTACGTTATAAGAAATTAATCAGTCAGTTGGATATTCGGGAGAAGTAATTTAATCG

TTCGAATTTTTTTCTTATTTTATTAGTAGTCTTATAGTAGTCTTAGATTTTTCATTTTGA

TGAGCCTCGTTTTGAGGAATTCATGGAATAATCCATTTTCATGGAATAAAGAATAAGAAC

ACGGATACATAACATAAAAAAAAGAATAAATAAGACGATATTCGCCCTCCCCCTACATAT

TTAATTTCTTCTCCTATACAAAAACCAGCAAGACCTACTCCATTGGTAATTCCATCAATG

ACACCCTTATCGAAAAACTGCGTTAGTTCAGTTAATCCTCTTATACCCAGGGTAAAGACC

CTAGTATAGAAAATATCTATATAACCACGATTATATGACCAACTGTATATCTTTTTTTTT

ACTTGATCCGAAAAAAACTTTTTCGGACCCTCTTTTACAAGAGAATTTATTAAATCCAAA

TTCTGAAAAAAGGAATAAGCGGATCCATAGAAGATATATGCTATGGATAGACCAAACATA

GCTAGACTTACAGAAGAAATTGCATTAGTGATAAATTCATATGAATTTATGGAAGAATTA

GAACTTTCCTGGAAAAAGTTTATTGAGGGAGTTAACCACTTTGATAATATGGTTAACTCC

GCTATTTCATTATCAAAATGGATTCCTATGGATCCAATGAACAAAGTAAAAAGCAGTAAT

ATAAAAAGAGGGAATAGCATAGTATTTCCCGTTTCATGAGGATAGACAAAAGTGTTTTTA

GCCCCAAAGGAAGTACTAAAGGACCCTATCCTATTTCTTGTATTACCATGAATTTTGGAT

CTATTTTGTGAAAAAAAAGAAACTCCACTCTTCGTTGTTGATAAAATGAAATCTCTATTC

ACTCCTTTGGGTATCCTTTTTCCCCATAAGGATATTGAATACAACGAACCCTCTTTAGTG

CTACTGTAATTTTGAAAATGAACACGCAGGTACCCATCAAAAGTAAGTAAATATATCCGA

AACATATAAAACGCAGTTAATCCTGCAGTAAAAGAGGCTATTATTCCAAAAAAGGGTGAA

TACAACCAACTATTACTAAGGATTTCATCTTTGGACCAGAAGCAAGCAAGAGGTGGAATA

CCACAAAGAGAAAGCGTACCCCATAAAAAAGTAGTTCTTGTAATTGGAACGTATTTTCTT

AAACCACCCATAAGAACCATATTCTGACTTTTATCTGGTGAATATCCAACAAGAGGTTCC

ATTGAATGAATAATGGATCCGGATCCCAAGAACAATAAAGCTTTCGAATAAGCATGAGTG

ATCAAATGGAATAAAGCAGCTTGATAAGAACCTATACCTAGAGCTAACATCATATAACCC

AATTGAGACATTGTAGAATAGGCTAAGCTTCTTTTAATATCTCTCTGAGCAAGAGCTAAA

GTAGCTCCTAAGAAGAGTGTTATTGTACCTACTAAAGAAATGAAACTCATTATTAAAGGT

AGGGATATGAAAAGAGGAAGAAGTCGAGCTAGAAGAAAAATCCCCGCAGCAACCATAGTT

GCTGCGTGTATAAGAGCCGAAATGGGAGTGGGTCCTTCCATAGCATCGGGTAACCATACG

TGAAGAGGGAATTGTGCAGATTTCGCAACTGCACCAAGGAATAATAAAAAAGCACACAAA

GTAGTAAGTAAGGAATTAATCCCATTATTAGGAATCCAGTTATTAGCTATTTTGAACAAA

TCCCTAAACTCTAAACTACCTGTTATCCAAAAAAAACCTAAAATTCCTAATAACAGACCA

AAATCCCCTACACGATTAGTTACAAAAGCTTTTTGACAAGCACTCGCTGCAATTGGCCGT

GTAAACCAAAAGCCTATCAATAAATAGGAACACATTCCCACAAGTTCCCAAAAAAAATAA

ATTTGTATCAAATTGGAACTAGTAACCAATCCCAACATGGAAGTATTGAAAAAACTTATA

TAAACAAAAAATCTCAAATATCCTTCATCGTGAGACATATAATCGTCACTATAAATAAGA

ACGAGGATTCCTACAGTAGTAATTAGTATTAACATAATAGAAGTAAGCGGGTCGATCAAG

TATCCAAATTCTAAGGAAAAATCATTATTGACGGTCCAAGACCATAGATATTGATAGATA

GAACTTCCATTTATTTGTTGAATAGATAGGTGAACTGAGAATACCATAGCTATACTTAAG

AGTAAAACACAAGGAAAAGCCCATATGCGACGAAGATTTTTTGTTGCTGTCGGAATAAGA

ATAAGTCCAAACCCCATTGACATAATAACTGGAAGTGGGAGAAGAGGGATTACCCATGCA

TATTGATATGTATGTTCCATAAGAAAAGAAATTGCAATTTTTCTATAAAATAAAATTGTT

TCCGATTCACCAAACCAATTCTTATCTCTTTCTGAAGGAATTCCAAAATACTGGAATTCT

TCATTTTTCCAATTTCTCTCATTGAAATATCAAAAATGAAGAATGGGTTTACTTGGTTAA

ATTCAAAAAGTTAATTAAATAACTTTGTTACCTAGTTATTACTAAAGAAGGATATTTGTT

AAAATACAAAAAAGGATTGAATCATTTTACTTTTGTATTTCTTTCTATTAAAATGAAGCA

GCTCTCATGTTTCGTAACTGAAATTGATTGGAATTCCTTAATTAACTATTTGAATTTTCC

CTTCCTTTTATCCCCCATCTTATATGGGGGATAGGCCGTAGATCTATATATGGAGTATAC

TTAATATTAATTTAAATAGAAAACAAAAAATTCTTGTCTTATCCGCATTAGAGAAAATCA

AGTAAAAAAGAATTCAGAATTTCAGTTCAGTATCTAGTATAAATACTAAGAAAAAACAGA

AAGAAGGATTGATTTGCGGCAATAGATGTCTTTCACATACAACTAGAAAAAGTAATCTCC

TTTTTGAATGGCAGTTCCAAAAAAACGTACTTCGATGTCAAAAAAGCGTATTCGTAAAAA

TCTTTGGAAGAAAAAGACTTATTTTTCCATAGTACAATCTTATTCTTTAGCAAAATCAAG

ATCATTTTCCAGCGGTAGCGAGCATCCAAAACCAAAGGGTTTTTCTGGGCAACAAACAAA

TAATCTGGTTTTGGAATAATCTGAATTGACCTATCCCAAAGAAATTCCAATTATTTAAAA

TGAATAATTCGGATTAATTAATGAATGTACTTTTATGTGTCGAATTCCTCGGTACAATAT

TCTTAGAACTAACCCCTCTGATATATAGAACAAAAGTTTTTGGTATACTGTGTCCTAAGT

ATTCTTTTCCTATCAACGAACTTTTCATAATAGAATCCTCTATTATGAGGATTCTATTAT

GAAAAGTAGAGTATTCTTGCAATAGGACTTACAACTTCTACCTATCTTATCAAAAATCCA

TTGCAACTGAAAAAAAATCCCCAATGATGAAATTCTAATGTCCTAAATTCTATGGACTCT

CCCAATCTCGACGATTTGCGAGAAAATAACTATTATTCTTTTAACTTCCCTATTATTTAA

AGTTAGCCGCCATGGTGAAATTGGTAGACACGCTGCTCTTAGGAAGCAGTGCTCAAGCAT

CTCGGTTCGAGTCCGAGTGGCGGCATTCTCGAAAAAGAATACAATAGATTAGAAATGATT

CAATTCGAAATTTCCAATTTTGTAATGGGACCTTCTCCTTATGCTATTTGCAACTTTAGA

ACATATACTAACTCATATCTCTTTCTCAACCATTTCAATTGTGATTACAATTCATTTGAT

AACCTTATTAGTTCGTGAACTTGGGGGATTACGTGATTCGTCAGAAAAAGGAATGATAGC

TACTTTTTTCTCTATAACAGGATTCTTAGTTTCTCGTTGGGCTTCTTCGGGACATTTTCC

ATTAAGTAATTTATATGAGTCATTGATCTTCCTTTCATGGGCTCTGTATATTCTTCATAC

GATTCCTAAGATACAGAACTCTAAAAATGATTTAAGCACAATAACTACGCCAAGTACTAT

TTTAACGCAAGGCTTTGCCACGTCGGGTCTTTTAACTGAAATGCATCAATCCACAATACT

AGTACCTGCTCTACAATCTCAGTGGTTAATGATGCATGTCAGTATGATGTTACTAAGCTA

TGCAACTCTTTTGTGCGGATCCTTATTATCCGCCGCTCTTCTAATCATTAAATTTCGAAA

GAATTTCAATTTCTTTTTAGAAAAGAAAAATGTTTTAAATAAAACATTTTTCTTTAGTGA

GATTGAATATTTCTATGTAAAAAGAAGTGCTTTAAAAAACACCTCTTTTCCTTCATTTCC

AAATTATTACAAATATCAATTAATTGAGCGTTTGGATTCTTGGAGTTATCGTGTCATTAG

CCTAGGGTTTACCCTTTTAACCATAGGTATTCTTTGTGGAGCAGTATGGGCTAATGAGGC

GTGGGGATCCTATTGGAATTGGGATCCTAAGGAAACTTGGGCATTTATTACTTGGACCAT

ATTCGCAATTTATTTACATAGTAGAACAAATCCAAATTGGAAGGGTACGAATTCCGCACT

TGTAGCTTCGATAGGATTTCTTATAATTTGGATCTGCTATTTTGGTATCAATCTATTAGG

AATAGGTTTACATAGTTATGGTGCATTTACATTACCATCTAAATGATTACATAACATAAA

ACCTTCGAGTTTCCATTTTTGTTTGATTTGAGAACCCTTGAACGCCTTCTCAAAGGGTTC

TCAAAAATTCGAGATAGATCTAATTAGACTTTTTTACTTTTTTCTGAATTTTTCACTATG

GAATATAGAGCGGACTAGTAAAAAAAAATTATTTAGGATAATAATTGGATAAGAGAGCCT

CTACCTTGTCAACCGATAGCGAGAGAACAAAATCTGGATAAATACCAATTCCTATTACTG

GTAAAAAGATACAGATTAAAAGAAAGAGTTCTCGTGGTCCAGAATCCACCAAATTTGCGT

TTGGAACATGAAATAGCTTGTATCCATAGAACATCTGGCGTAACATAGATAATAAAAAAA

TAGGAGTTAATATCATTCCAATTGCCATTACAAAAGTAATTAGCATTTTTGGTATTAACA

GAAATTTTGGACTAGTAATTAGTCCAAAAAATACTACTAATTCTGCAACAAAACCGCTCA

TTCCTGGTAAGGCAAGAGAAGCCATTGAAAAGCTACTAAACATGGTAAAAATTTTCGGCA

TTGGGATAGATATCCCCCCCAGTTCTTCGAGATAAACAAGACGCATTCTATCACAAGCCG

TTCCCGCCAAGAAAAAAAGTGTAGCACCAATAAATCCATGGGATAGTATTTGTAAAATAG

CTCCATTGAGTCCAATGTTGGTTATGGAACCAATTCCTATAATTATGAAACCCATGTGAG

ATACGGAGGAGTAGGCTATTCTTTTTTTGAAATTTCGTTGACCAAGAGAAGTTGAAGCTG

CATAGATTATTTGCATCGCTCCTATTATTACCAACCAGGGGGAAAATAGATAATGAGCAT

GAGGTAACAATTCCATATTGATCCGAATCAATCCGTATGCTCCCATCTTTAATAGGATTC

CCGCTAAAAGCATACATGTACTGTAATGCGCTTCCCCATGGGTATCTGGTAACCACGTAT

GTAGGGGTATAATCGGCAATTTGACAGCATAAGCAATAAGGAAGCCAAAATAAAATAGTA

TTTCCAATGTTGCAGGGTATGATCGATTAATTAATCTTTCCAAATCTAATCTTGGTTCGT

TGGAACCATATAAGCCCATACCTAGAACTCCGATTAAGAAAAAAATGGAACCGCCTGCAG

TATACAAAATAAATTTTGTAGCTGAATACAGACGCCTCTTTCCCCCCCACATGGATAAAA

GTAAGTAAACAGGAATTAATTCTAACTCCCACATGATAAAAAAAAGTAAAAGGTCTCGCG

AAGAAAATAATCCTATTTGACCACTATACATTGCTAGCATCAGGAAATAGAATAATCGCG

AATTCCGGGTAACTGGCCAAGCTGCTAAAGTAGCTAAAGTAGTGATAAATCCTGTCAATA

AAATAGATCCTAATGAAAGTCCATCGATTCCCAATCTCCAGTGGAAATCAAAGACATCTA

TCCATTTAGAATCCTCCTTTAATTGGATTAAGGGATCCTCCAATTGGAAATGATAACAGA

ATGCATAAGTCATTAGAAGGAATTCTAATAAACAAATAGATATAGTATACCACCTAACGA

TTTTGTTTCCCCTATGAGGTAAAAAGAAAATTAATGAACCTGCAAATATCGGCAAAACAA

CAAGTATTGTTAACCAAGGAAAATAACTCATGATAAAGTGATAAAGAGAAGATACGTTTT

GACCAGAAAAGCCCGTGCTCGAAATAAGCGAGCACAGGCTTCCTCGGTAAAGAGGAATCA

GACGATTCAAGTGGAGTTTTTTGTAACGTATCAATAAGATAGAGCCATGCTGCGGGTTGT

TTCAGGCCCTAAATAAACGCGGACACTTAAAAAATCTGTTGGGCAGGCAGATTCACATCT

CTTACAACCCACACAATCTTCGGTTCTCGGCGCGGAAGCAATTTGCTTGGCTTTACACCC

ATCCCAGGGTATCATTTCTAATACATCTGTTGGACAAGCTCGTACACATTGAGTGCATCC

TATACATGTATCATAAATTTTTACGGAATGTGACATTGGATCTATAAATTTTCCTTTTCA

ACATAAAAATTTTCGATCTGGTAAAAATGAAATTAGTACTATATGAGTCATATGTATTGT

AGACACCAGACGAAGCAATGGTTTATCCAAACTTCAACAAATAATGCAATATATTTCTTA

ATCCGTTTGTGAGAAAGCGTGAAAAGAGCCAAGAGACTTGAATTTTTGGCTTCAACAATC

ATAATTATACGAATTGTACATACGAATTCGAATTAGCCAATTTATTGGCTATCGTCTTTT

CAATATAAATTATTGCAATATTCAAATTGCAATATCAATGAATTGCAAAAATTCAATAAG

TAAAAAGAATACTATGTAATAACCTAATCAAAAAATAGATATTATAAAATAATAAATAGT

ATTATTAATATTTGTTATTATTATATGTGCGCCTTTGCTTAGAGGATTTTATGTCTAATT

ATTCAAAAAATTAGATTGATTGATACGAGTTGATTTCTTGTTACGATGGATGGAAGAAAG

AATGGATAGTCCAATAGCTGCTTCAGCAGCCGCAAGGGCTATAACAAAAATTGCGAAAAT

GTCTCCTTTTAATTGGCGACTATCAAATAGATCAGAAAATGTTACGAGATTTAGATTAAT

TGAATTCAGTATAAGTTCAAGACATATTAGAGCTCTAACCATGTTTCGGCTTGTGATCAA

TCCATAGATACCAATCGAAAATAAATAGACACTAAAAAAAAGTACATGCTCAAACATCAT

TAACTAACTCCTTATCAATCTCGATTCATTTCAATATGAGGACAAGAATTGAACCGATTC

CATTAATTAGAATAGAACAGTTACACAACAAAAGAGAAAAGAAGGTATTTGTTGGCAGTA

GATGGGTTTTACTAAATCAAAATTGTGATTCTTTAGTTATTTATTTTAGATTTGAAATTC

TAAGAATTTGACTAATTCTAAGTATTTCTTATTGCCGAGCCATAGTAATTGCACCTATTA

AAGAAACTAGAAGAATTATGGAAATGAGTTCAAACGGAAGATAAAAATCAGTTGCTAAAT

GAATCCCAATTTGTTGAACGTTATTTATGAGACCCTGTTCTACTATTTGGTTTGATCTTG

TAGTCCAAAGAATTCCATACCATGACGTATCTGGGATAGTAGTCATTAGTGAAAAAAGAA

TAGTTATACAAACGAGTGAAGTGAACCCATCTCCAATAGTCCAATAATTCTTATTTTTAG

ACCATTCTGAGCCATTTACGAACATTACGGCAAATATGATCAAAACATTTATAGCTCCCA

CATAAATAAGAAGTTGTGCGACAGCTACAAAGTAGGAATTCAATAAAATATAGAATAAGG

ATATACAAACAAGAACTAATCCCAGCGAAAAGGCAGAATAAATTGGGTTGGTAAGTAATA

CTACTCCTAGACCTCCTAGTAGAAGAACAAATCCCCCAAATAGCACAAGAATCTCATGTA

TTGGTCCAGGTAAATCCATTATGGATAAGAAGAAATTAATAGTATAAAATTTTTCATGAA

CTGACTAAAACTAAAAGATTCAAGGAAGGAAAAAGGGATTAGGATATTTTTTGTATATAA

GTTGTTAGTTATTCGTTTTTCTTTATAGTTAATAAAAATGGATTCTAAAAGATAAATCCT

AATAATTAGTAACCGTTCTTGAATTCCAAGATTTTTCTTCGTCTATTTTACTTTGAGTCG

AATTCCTAATTGTTTGAATTGTGTAATCTCCCATTATGGAGATTGGTAACCGACTCAAAG

CAATTTGATTGTAATTCAATTCATGACGATCATAAGTAGAAAGTTCATATTCTTCAGTCA

TTGATAAACAGCTTGTCGGACAGTACTCAACACAATTACCACAAAATATACAAACTCCGA

AATCAATACTATAATTAAGCAATTGTTTCCTTTTAATATCCTTTTCAAATCTCCAATCCA

CAAGGGGTAGATCTATCGGGCATACGCGAACACATACTTCACAAGCAATACATTTATCAA

ATTCAAAGTGGATTCGCCCCCGGAAACGCTCCGATGTAATTGATTTTTCATAAGGGTAGT

GAATCGTTATAGGTAAACGATTTGTGTGGGATAAGGTAATTATGAAACTTTGACCAATGT

ACCTTGCAGCGCGTATTGTTTGTTGACCATAACTCATGAACCCAGTTACCATAGGGAACA

TATTCTAAATATCTATGAAAAAGATATGTTTCTTTCTCTTGTTTGAGAGAACTTTTGTGT

TGAAAATATTCTTACTGTTATTGTATTCTTATTTATAGTGAAACAAGTTGGGAAGAAGTT

GTTAATAAGAGATTGCCCAGGGAAATAGGTAAAAGAAATTTCCATCCAAGATTTAATAAC

TGATCCATTCTCATCCTGGGTAAAGTCCATCTTATTGTGATAGAAATGAAGAGAAATAAA

TAAGCTTTAGTTAATGTAATAAAGATACCCATTGTCATTTCCAAAATTCCAACCATTTTA

TTCATTTGGAAAAATTCAAAAAAGGATATATAGGGAATAGAGAAATTCCACCCGCCTAAG

TAGAGAACTGTTACAAATAAAGAGGAAACTAATAAATTTAGGTAAGAAACAAGATAAAAT

AAACCATATTTGATACCGGAATATTCGGTTTGATAACCTGCTACTAATTCTTCCTCTGCT

TCTGGTAAATCAAAGGGTAATCTTTCACATTCTGCCAAAGAAGAAATTAGAAAAACCAGA

AAACCTATAGGCTGACGCCAAAGATTCCATCCAAAAAAACCATATTTTGACTGTGCTTCA

ACTATATCAACTGTACTTGAACTGTTAGATAATCATAGTCGATGATAACATCACAGTTCC

CACCGCTATTCCAAAACCGTACATGAAACCTTAGCTTCATACGGCTTCTCTATGATCAGA

AAAAGGAAAGGGTTGTTTCGTTTCGGTATTATCCCCTGGGCATAGATAGAATTAGGTAAG

ATAAAATCGATTGGAAAGTCCTAAATTAGACCAAAGGAATTCCGTCTGCTAGAATAAGAA

AAAGCGCTTCCGAATTGATCTCGTCCTTTATAATATAAAATTTTTCTTTGTTCAGTAATA

ACTTAATCTTGGAATAAAACACTCGTTATAGCAATTAATAAATGAAAAGAATTAGGCATT

AATTCATGAGGAATTCTGTATTAATATGAATAGAGGAAGGAAAAAATAAATAAATATCTT

TTTTTGTATTGCATTCCATATCTTTTGTCCTATTCTTCTTTCCCCGAAGGGTATAAAAAG

AAAAAAGGAATAAAGGATTAATTCGTTCTTGATAGCCATTTCTTTAACAAGTGAAAGGGA

ACATACTCTGGATCGGAATCCGAAGAAGTACTACTTGATCATTTCCACCAATTTCAAGTC

CTTATTATGATTCCTTTTATGAGGAAAAATCTCTAATGCCCTTTATGTACTTTAGTGTTC

CTAACCCCTCACTAATTTTTGATGGATTCCCTTATGATTATAAGTTTCCATATCGCGAAT

CCTTTATTCTTGCCCGCTTCAAGATATGATGACTAATCAAAAAATCTCAACCTTGGGGTA

AAGAGTTTACACTACTTATGTTTACTTCAATTTTTTCTTGTACGTAGGAAATGAGATTTT

TTCTTTTTACTACAAATTAATAAGTTGTTTTGTTTCACTCATATAGCTATCTAGTTTAAC

TTACCAACCCGAGAATAAGAAAAGGAAGATAAATATTCAATGGATTTTGGAGGAAAAAGA

TCCTATTTTAACGAATCACACGTAGAGATATTGCTAGCACACAAAAAGTTAATGGTATTT

CATAACTAATAGATTGAGCAGCAGCTCGTAGACCGCCTGAAAAAGAATATTTATTATTTG

AGCTATATCCTGCCATAAGAAGACCAATAGGAGCAATACTTGAAATGGCAATCCATAAAA

AAACACCAATACTAAGATCGGCTAAAACAAAACGATATCCCAAAGGGATAACTAAAAAAC

TTAATAAAATTGATATGACTGCTATAGAAGGTCCAATGCTAAATAAAGGAATATCTCCTC

GGGATGGCAGGATATCCTCCTTAAAAAGTAGCTTAGTTCCATCGGCTATAGCTTGAAGCA

GTCCCAGGGGGCCAGCATATTCAGGACCAATACGTTGTTGTATCGATGCGGATATTTCTC

TTTCTAACCACACAATTACGAGTACTTCTATTGTGATTCCCAGTAGGAGGGTCAAAATGG

GTAGAATCCATATCAGTCCATAGACTTCTTTTAATAATTCCGATTTCGAAAAAGAATTGA

TAGTTTCTATCTCTACCCTATCTATTATCATTTCAACGATCAACTTCCCCCATAATGATA

TCTATACTACCTAATATCGTCATGATATCAGCCAATTTCATTTTTTTAACTAGTTGAGGA

AGAATTTGCAAATTAATAAAACCGGGTGGACGAATTTTCCATCTCCAGGGGAAAAGACTA

TCATCTCCTACCAGATAAATTCCTAATTCACCTTTTGGAGCTTCCACTCTTACATAAAGC

TCTTGCTTTGACAATTCAAAATTGGGCGAAGGTTTTTTACCAAGAAATCGATATTCAAAA

TCATTCCATTCGGAATTCTTTGTTTTCTTAAAGCGTCGGACTTCTAAATTCTCATAAGGG

CCTCCAGGAATTTTCTCTACAGCCTGTTGAATAATTTTGATGGATTCCCTCATTTCACCC

ATTCGTACTAAATAGCGAGCTAATGAATCCCCTTCTTTTTGCCATTGGACTTTCCAATCG

AATTGGTTGTAAGACTCATAAGGATCAACTTTACGAAGATCCCATTGTATTCCAGAAGCT

CGTAACATCGGTCCCGATAAGCCCCAATTTACTGCTTCTTCTCCGCTAATAAAACCGACT

CCTTCAACTCGTTCTAAAAAAACGGGATTCCGTGTAATAAGTTGTTGATATTCAACAACT

CCTCGTAAAAAATAATCACAGAAATCTAAACATTTATCGACCCATCCATAAGGTAGATCG

GCGGCTACCCCTCCGATGCGAAAGTAATTATGCATCATTCGCATACCTGTAGCAGCTTCA

AATAGATCATATATCAATTCTCTCTCTCTAAAAATATAGAAAAAAGGGGTCTGTGCGCCT

AGATCCGCCATAAAAGGTCCAAGCCATAACAAGTGAGAAGCTATACGGCTCAACTCTAAC

ATAATTACCCTAATATAGCTGGCTCTTTGGGGTATTTGAATATTCTCCAAGAATTCTGGT

GCATTTACCGTTATTGCTTCTGTAAACATAGTAGC

[4] Fargesia_hygrophila

CCCAATATCTTGCTGGAACAAGATATTGGGTATTTCTGGCTTTCCTTCCTTCAAAAATTG

CTATATGTTAGCAGAAAAGCCTTATCCATTAAGAGATGGAACTTCAAGAACAGCTAGGTC

TAGAGGGAAGTTGTGAGCATTACGTTCGTGCATTACCTCCATACCAAGATTAGCACGGTT

GATGATATCAGCCCAAGTATTAATAACGCGACCTTGGCTATCAACTACGGATTGGTTGAA

ATTGAAACCATTTAGGTTGAAAGCCATAGTACTAATACCTAAAGCAGTGAACCAGATCCC

TACTACAGGCCAAGCAGCCAAGAAGAAGTGTAAAGAACGAGAGTTGTTGAAACTAGCATA

TTGGAAGATTAATCGGCCAAAATAACCATGAGCAGCCACAATATTATAAGTCTCTTCCTC

TTGACCAAATTTGTAACCCTCATTAGCAGATTCATTTTCAGTGGTTTCCCTGATCAAACT

AGAGGTTACCAAGGAACCATGCATAGCACTGAATAGGGAACCGCCGAATACACCAGCTAC

ACCTAACATGTGAAATGGATGCATAAGGATGTTGTGCTCTGCCTGGAATACAATCATAAA

GTTGAAAGTACCAGATATTCCTAAAGGCATACCATCAGAGAAACTTCCTTGACCAATAGG

GTAAATCAAGAAAACAGCAGTAGCAGCTGCAACAGGAGCTGAATATGCAACAGCAATCCA

AGGACGCATACCCAGACGGAAACTAAGTTCCCACTCACGACCCATATAACAAGCTACACC

AAGTAAGAAGTGTAGAACAATTAGCTCATAAGGACCGCCGTTGTATAACCACTCATCAAC

AGATGCAGCTTCCCAAATTGGGTAAAAGTGCAATCCGATCGCCGCAGAAGTAGGAATAAT

GGCACCAGAGATAATATTGTTTCCATAAAGTAAAGAACCAGAAACAGGCTCACGAATACC

ATCAATATCTACTGGAGGAGCAGCGATGAAGGCGATAATAAATACGGAAGTTGCGGTCAA

TAAGGTAGGGATCATCAAAACACCGAACCATCCGATGTAAAGACGATTTTCGGTGCTAGT

TATCCAGTGCAGAAGCGACCCCACAGGCTTGTACTTTCGCGTCTCTCTAAAATTGCAGTC

ATGGTAAGATCTTGGTTTATTCAAATTGCAAGGACTCCCAAGCACACGTATTAACTAAAA

AGATAATAGAAGGCTTGTTATTTAACAGTATAACATAGACTGTATACCAATGTCAACCAA

GCCAGCCCCAACGATTGGATATCCATATAACTAAATTCACCAAACCAAAAATTTTGTAAA

TGAAGTGAGTGAAAATTCAAAACTCAGATTATTTCCATATGGGTTGCCCGGGACTCGAAC

CCGGAACTAGTCGGATGGAGTAGATAATTCTTCCTTGTTACAATAGAAAAAATCCCTCCC

CAAATCGTGCTTGCATTTTTCATTGCACACGACTTTCCCTATGTAGAAATAGTCAATTTC

TATTCCAAAGAGGAAGTCTACCAATTTTTTGAATAGTAAGTTGATTCACCTACTATGAAC

ATTTCAGAATGGAAAATGTGAAAGTTTTATCTTGATATCGATCTTTCTAGTGTATTAGTT

TTGTCTAATGATTAATTAAAAGGGTTCACCAGGTCATTGATACGGATAATATCCAAATAC

CAAATACGGTCACTGTGTGATCCACGGAAAGAAAAGTGGGTTGTTTTGGTGAACATCAAA

GAAAAAACTTGCTCTTCTTCCGTAAAAAATTCTTCTAAAAATACCGAACCCAACCGTTGC

ATAAAAGTTCGTACCGTGCTTTTATGTTTACGAGCTAAAGTTCTAGCGCATGAAAGTCGA

AGTATATACTTTAGTCGATACAAAGTCCGTTTTTTCGAGGATCCACTATGATAATGAAAA

AGATTTCTACATATCCGACCAAATCGATCAAGAATATCCCAATCTGATAAATCGGTCCAA

ATGGGTTTACTAATAGGATGCCCCGATCCAGTACAAAATTGAGCTTTTGATAAGGATCCA

ATGAGGGGAGTAGCAGGGACTATGGTATCGAATTTTTTCATTTGAGTCTCTATTAGAAAT

GAATTCTCTAGCATTTGATTCCTTACTAACAAAGGATTTATTGGTACACTTGAAAGGTAC

CCCAGAAAATCGAAGCAAGAGTTTACTAATTGGTTTAGATGGATCCTTCGCGGTTGAGTC

CAAAAAGAAAAAGAATATTGCCAGAAATTGACAAGGTAACATTTCCATTTCTTCTTCAAA

AGAAGAGTTCCTTTTGATGCAAGAATTGCCTTTCCTTGATATCGAACATAATGCATAAGA

GGATCCATAAAGATCCATATGGTTTTCCGAAAAAAACCTGGGTACATTACCCCAAAATGT

TCCATCTTCCTAGAAAAGTGGATTCGTTCCACAAAGGTTCCAGAAGATGTTAATGGTAAG

CAAGAAGATTGTTTACGAAGAAACAACAAGAAAAATTCATATTCTGATACATAAGAGTTA

TATAGGAATCGAAATAGTCTTTTATTTTCTTTTTTCAAAAGAAAAATCGATTTCATTGAA

GTAATAAGACTATTCCAATTCGAATAGTAGTTGAGAAAGAATCGCAATAAATGCAAAGAT

GGAACATCTTTGATCCGGTATTGAAGGAGTTGAACCAAGATTTCAAAATGGATAGGATAG

GGTATTTCTATATGTGATAGATAATGTAAATGCAAAAATTTGTCTTCTAAAAAGGGAAAT

ATTGAATGAATAGATCGTAAATTCTGAAACTTTGGTGTTTCTTTTTCTTTCGGACAAGAT

AATTCTCGTAGCGAGAATGGGATTTCTACAACGATCGCAAACCCCTCAGATAGAATCTGA

GAATAAAACTCAGAATAAAAAAAATTGTTGTAATCCAACAATCGATCTTGGTTAGGATGA

TTAACCGAGTTAATCCAAAAATTCTGCTGATACATTCGAATAATTAAACGTTTCACAAGT

AGTGAACTAAATTTCTTGTTATTACAACTAACAATTTCCACAGGTTCGGAACCTTTTAAT

CCATAATCATGGGCAAATGCATAAATATACTCCTGAAAGAGAAGTGGGTAAACGAAGTAT

TGTTGACGAGATTTCTGTTTTTCTGAATACCCTTCCAATTTTTCCATTTGTATTTCTACT

TGAATCAGAAAGAAGAAGCATTTCTCGGTTTCTCAAATGATGATACATAGTGCAATATGG

TCAAAACAGGGTGTTGCATAATACAAACCTTTCTATCCAATTAGTTTATGTTTGTTCTAA

TTACAAAAGAGAACAAATCTTTTATTTTTGCAGGCCAATCGCTCTTTTGACTTTGGAATC

CAGTCTCTTTATCAATATACTGCTTCTTTTACACATTCAATCCATAACATCCTTTTCAAT

CTATAATCAAGAATAATTAGGATTTCAAAAAAAAAAGAAAAAGGGTCCGTTCATAGGAAA

ACCAACCTTTCCCCGCATCAGGCACTAATCTATTTTTAACGTCTAATTAGATCGGGGAAT

CATTTCAATTAAGAAGTTAAGCTCGTTGCTTTTTATTTTACCAGAATTGGAGCCAGGCTC

TATCCATTTATTCACTAGACCCAGAAAATAGGAATTTTTTTATTCCAAAAAAAAAAAGAA

ATTGATTTTATTACGACATGCTATTTTTTCCATTCATTACCCTTGAGGATCAGTCGTGGT

CTTCTAGACTCTACCAAGAGTCTGGACGAATTTGTTGCTCATCCAAATGTGTAAAGATCA

TAGTCGCACTTAAAAGCCGAGTACTCTACCATTGAGTAGCACCCAGATAAATAGGATCTT

AGATACGATCGAACCCAAAATCAATGGAATTACACCACATTGAACTAGCAAAACATTAAA

AGAAAGATTTTATCGCCATTAAAAACACTCAAATGCAAAATGAACAGGTCCGGCTAAATT

TCACTAAGGTTAAAAGCGGCCCCAATCACGATAGCAAAATTGTCATTTTTTTAGCATTTA

TATATATAAATAAATCTTGTATGAGAGTACATGCAAGAGGGACAACCTTATCATTTGAGC

GAAGTGTAGACAAAAAACCTAATATGGAGTGAGGATAAAGAGACCTATCTATCTACAAAT

TCTATTTGTTCAATAGACCTTTGTCAATGGAAATACAATGAAATTAGATAGAAAAAGTAA

ATAAAATAAGGGCTTATGTTGGATTGGCACGACATAAATCCAGTCAAAAATAGGACTAAG

AGGCAAATTGTGTCTAAATAATTAAGGGATACTAGTGATCCTCTCCTACTTTTTTATTCA

TTTAGTTCTTCAATTAACTCAAAGTTCCTTCTTTTTCTTTAAAGAATTCTGCCTTCCTTA

AAATATCATAAACAGTTCTTGTAGGTTGAGCACCCTTTTCAAGGAAATAGAGAATAGCTG

GAACATTTAAACAAGTTTGATTCTTTATCGGATCATAAAAACCTACTTTTCGAAGATCTC

TTCCTTCTCTTCGAGATCGAACATCAATTGCAACGATTCGATAGACAGCTTATTGGGATA

GATGTAGCTAAACAATCCCCCCCTAGAAACGTATAGGAGGTTTTCTCCTCATACGGCTCG

AGAATATGACTTGCATTAATTTCCTTACAGAAAAACAAATTTCATTTATACTCATGACTC

AAGTTGGTTAATTTTGACTGACAGACTTAAAAGGAAAAATCCTTCCAAATTTTTTGAGTC

GTCTCTAAACTCTTTTCTTTGTCTCATCTCGAACGAATTGACTTTTATTCCTTATTCTGA

TCCAATTCTATTGTTGAGCAATTGAAAATCGCGTTTACTTGTTCCGGAATTCTTTATCTT

TGATTTGTGAAATCCTTGGGTTTAGACATTACTTCGGGAATTCCTATTCTTTTTTCTTTC

AAAAGAGTAGCAACATACCCTTTTTTCTTATTTCCTTCGATAAAGCATTTCCCTCTTCTA

TAGAAATCGAATATGGGCGATTGATTCTGATAAACTTTTAATTGAAAGAGTTTTTCCAAT

CTTCCAAAATTGGACTTTTTCTTATTTTAACCTTTCGATTTCTATATTAAGGATAGACTG

ACAAAGTTGGCCTAATTTATTAGTTTTCACTAACCCTAGATTCTTTCCCTTGATAAAAAA

TCAATTCTGTCCTCTCGAGCTCCATCGTGTACTATTTACTTACAAACAACCCAGCGCAAA

TTTGGTTCGGGACGAATAGAACAGACTATGTCGAGCCAAGAGCATTTTCATTACTATGGA

AAATGATGGATAACAAAATCCACAATCGATCATGTCCTTCAAGTCGCACGTTGCTTTCTA

CCACATCGTTTTAAACGAAGTTTTACCATAACATTCCTCTAATTTCATTGCAAAGTGGTA

TAGGGAATTGATCCAATATGGATGGGATCATGAATAGTCATTTTTTTGTATACTAATTCA

AACTTGCTATCTATGGAGAAATATGGATAAAAGAAATAAGTATTTATCGGGGAAGACTCC

GCAAAGATCCAATTTATTTAAACCCATATTCTATCATATGAAGGAAACATAGTTCGAAAA

AGACGAATAAACAAGTTTGCTTAAGACTTATTTTTTATTGAATTTCCATCCTCAACAGAG

GACTCGAGATGGTCAATCCTGAAATGAGAAGGATCGACTCTTCTCCAACAAATAAACTAT

CAACCTCAAGTTTAATTAATTTAATTAGCAATATATTTTTCCATAACAAAAACTATTAAC

TAAATAAACTATTCCAATGAAAAGAAAGTTTTTTGGTAGTTATAGAATTCTCGTACTCTT

CGACTCGAATACCAAAAGAGGACTCCAAATCAAAATTGAATCCATTCTATCCAACGAACA

GTTCTTACCTTATCCTTACCAGAATGGATCATCTGGATATTTAAAGAATCGCAGATCGAG

ATGGTTTTCGCTTAACCAAAGAGGGGCCCTTTTTACTAATAATATAATACAACAAAAATC

TATCTCTATCATAAAGGGATAGGTCTCATTTTTTATACAGTGTTTTACGTTTTTTCATGA

AAAAAAGATATTCAATTTGACTGGACTTGACACTTGATTATGTTTTCTGAGAAAGAAAAA

AATGCTTAGAAATGCATCTAATCTAAGAGTTCATAAGAGATAATTATTCTCTTTAATAAA

CTTTTGTCTCGTGTGGGGTACAATATGATTTCATCTTTCGTTTCATCAGAAAAATCTGGG

ACGGAAGGATTCGAACCTCCGAGTAACGGGACCAAAACCCGCTGCCTTACCACTTGGCCA

CGCCCCATTTCTGGTTTTATGCGACACTAATAAACACTATTATGTTTATTTGTTATTCGT

CAATCCCACTTCAATTACATAAAAATGAGGGATACTCTCTTGCTAGGATTCTAGACATGC

GGATAATATAGAATCCAAAAAATGCATTGATCATTACATGGAATTCTATTAAGATATTAT

ATGAAAGTCGAATTTCTTCCATTCTCATTTGAGAGTGCGAATACAAGGAGGTATTTTGCG

TTTGGGAAAGTCCGAAGAAAAAAGGATTTTGAACCCGCCTTTTCTTTTTTCCCTTAGAAA

AATAACTCAATCAAAATCCAATTATCTACTCTACAAGAACGAAATGCTTGTTATGCCTAA

TATACTTAGTTTAACCTGTATCTGTTTTAATTCTGTTCTTTATCCGACTAGTTTTTTCTT

CGCCAAATTGCCCGAAGCTTATGCCATTTTCAACCCAATCGTGGATTTTATGCCTGTCAT

ACCTATACTCTTTTTTCTATTAGCCTTTGTTTGGCAAGCTGCTGTAAGTTTTCGATGAAA

TCTTTACTACTCTGTCTGCCAAATTGAATGATGTATTCATTCCAAAAAAAAAAATGAATA

AAAGCCGAGAAGTCTTATATTATGAACCTTCGATTCTAAAATTCTAATTCTTCTACATTG

AATGTATAGCTGCAGCAATAAATTGGGATCCGCCTTTCTACCCCTGCACCTACGTTGAGC

AGGTACCTTTAGGTACCCACACAATACCTAACCTAATTTTTTGATAAGAGTGCTTATTAT

AAATCAATTCTTGCAATTTTTTTAAGAATTGATTTTTGCATTTTTAGGTGTAAAAATAAA

AAAACCCATCCTAGTGGATCTGTGTGGTAAGGAAAACGGGTAATCTATTCCTTAAAAAAA

ATCTTGGAGATTATGTAATGCTTACTCTCAAACTTTTTGTTTATACAGTAGTGATATTCT

TTGTTTCCCTCTTTATCTTTGGATTCTTATCTAATGACCCAGGACGTAATCCTGGGCGTG

AGGAGTAAAAATCCAAAATTTTTTCTTACAAATTGGATTTGTTTCGTACATTTATCTATG

AGAAAATCCGGGGGTCAGAATTCCTTCCAATTCGAAAGTCCCAAATGATCCGAGGGGGCG

GAAAGAGAGGGATTCGAACCCTCGGTACAAAAAAATTGTACAACGGATTAGCAATCCGCC

GCTTTAGTCCACTCAGCCATCTCTCCCCGTTCCAAATCGAAAGGTTTCCGTGATATGACA

GAGGCAAGAAATAACGATTGCAAAAAATCCTTCCTTTTTCTTTCAAAAGTCCATAAAAAT

TATATTGCCAATTCCATTTTAATTATATTCTTTTTTCTTAATAAAAAAGAAGAAAATTCT

TGTTTTTTCTTTCTAAAATTCGATATTGGCTGAGAAACAATCAGATAGATTTTCTCTTCA

GCGGGCATTTTCATATAGGACTTGTTATAATAAAACAAGCAGGTTATATAAAAAATATTT

ATAAACAAAACAAAAAGGGTTCTTATCAAACCCACCATAAAATTGGAAAGAAAGATAAAG

TAAGTAGACCTGACTCCTTGAATGATGCCTCTATCCACTATTCTGATATATAAATTCGAT

GTAGATGAAATTGTATAAGCGGATTTTTGTATTTCCTTAGACTTAGACCGCGCAAGGCAA

GAATTTTTCGCTATTTACGATTTCATATTCTTGTTACTAGATGTTCTATAGGAATAAGAA

GAAATCGCAACTCCTTTCCGCTACACATAAAAATTGATTTCGAAAGTCAATTTTTTTCAA

TATCTTTCTTTTCCTTAAAAGATAGGCTTTGAAATAGGAGTCATGGAATAATGCTGAATT

CAAATGTTTATTTCTATAGTATAAGAAAAACTAATCGAATCAAATTCATGGATTTACCAC

GACCTCGGTTGTGACCCCATAGATAAAAATAAAAAATTTCTATCTTCGAGACCTTTGAAA

AAGGGCATTGAACGAGAAAAAATCGTCCACAGATAATCTATCGTATGCCTTGGAAGTGAT

ATGAGGTGCTCGGAAATGGTTGAAGTAATTGAATAGGAGGATCACTATGACTATAGCCCT

TGGTAGAGTTATTAAAGAAGAAAATGATCTATTTGATATTATGGACGACTGGTTACGAAG

GGACCGTTTCGTTTTTGTAGGATGGTCCGGCCTATTGCTCTTTCCTTGTGCTTATTTCGC

TTTAGGGGGTTGGTTTACAGGGACAACTTTTGTAACTTCTTGGTATACCCATGGATTGGC

TAGTTCCTATTTGGAAGGTTGTAATTTCTTAACCGCGGCAGTTTCCACCCCTGCCAATAG

TTTAGCACACTCTTTGTTGCTACTATGGGGCCCGGAAGCGCAAGGGGATTTTACTCGTTG

GTGTCAATTAGGCGGTCTGTGGACTTTTGTCGCTCTCCATGGGGCTTTTGCACTAATAGG

TTTCATGTTACGTCAATTTGAACTTGCTCGGTCTGTTCAATTGCGGCCTTATAATGCAAT

TTCATTCTCTGCTCCAATCGCTGTTTTTGTTTCCGTATTCCTTATTTATCCACTGGGGCA

ATCTGGTTGGTTCTTTGCGCCGAGTTTTGGCGTAGCAGCGATATTTCGATTCATCCTCTT

TTTCCAAGGATTTCATAATTGGACATTGAACCCATTTCATATGATGGGAGTTGCCGGAGT

ATTAGGCGCGGCTCTGCTATGCGCTATTCATGGGGCGACCGTAGAAAACACTCTATTCGA

GGACGGTGATGGTGCAAATACTTTCCGCGCTTTTAACCCAACTCAAGCTGAAGAAACTTA

TTCAATGGTCACTGCTAATCGCTTTTGGTCCCAAATCTTTGGTGTTGCTTTTTCTAATAA

ACGTTGGTTACATTTCTTTATGCTATTTGTACCCGTCACCGGTTTATGGATGAGTGCTAT

TGGCGTAGTCGGCCTGGCTCTGAACCTACGTGCCTATGACTTCGTTTCCCAGGAAATCCG

TGCAGCGGAAGATCCTGAATTTGAGACTTTCTACACTAAAAATATTCTTTTAAACGAGGG

TATTCGTGCGTGGATGGCAGCTCAGGATCAGCCTCATGAAAATCTTATATTCCCTGAGGA

GGTTCTACCACGTGGAAACGCTCTTTAATGGAACTTTCGTTTTAGCTGGTCGTGACCAAG

AAACCACCGGCTTTGCTTGGTGGGCTGGGAATGCCAGACTTATCAATTTGTCCGGTAAAC

TACTTGGAGCTCACGTAGCCCATGCCGGATTAATCGTATTCTGGGCCGGAGCAATGAACC

TATTTGAGGTGGCCCATTTCGTACCAGAAAAGCCCATGTATGAACAAGGGTTGATTTTAC

TTCCGCACTTAGCTACTCTAGGTTGGGGAGTAGGGCCGGGGGGAGAAGTTCTAGATACTT

TTCCGTACTTTGTATCTGGAGTACTTCACCTAATTTCCTCCGCAGTCTTAGGCTTCGGCG

GCATTTATCACGCGCTTCTGGGACCCGAGACTCTTGAAGAATCTTTTCCATTCTTTGGTT

ATGTATGGAAAGATAGAAATAAAATGACTACAATTTTGGGTATTCACTTAATTTTGTTAG

GTATAGGTGCTTTTCTTCTAGTACTCAAGGCTCTTTATTTTGGCGGTGTATATGATACCT

GGGCCCCGGGGGGGGGAGATGTAAGAAAAATTACCAATTTGACCCTTAGCCCCAGTGTTA

TATTTGGTTATTTACTAAAATCCCCTTTTGGGGGAGAAGGGTGGATTGTTAGTGTGGATG

ATTTAGAAGATATAATTGGGGGACATGTATGGTTGGGTTTCATTTGTGTATTTGGCGGAA

TTTGGCATATCTTAACCAAACCCTTCGCATGGGCTCGCCGTGCATTTGTATGGTCTGGAG

AAGCTTACTTGTCTTATAGTTTAGGCGCTTTATCTGTCTTTGGTGTTATCGCTTGTTGTT

TTGTCTGGTTCAATAATACGGCTTATCCGAGTGAGTTTTATGGACCCACTGGGCCAGAAG

CTTCTCAAGCTCAAGCATTTACTTTTCTAGTTAGAGACCAGCGTCTTGGAGCTAATGTGG

GATCTGCCCAAGGACCCACAGGTTTAGGTAAATATCTAATGCGTTCCCCAACGGGAGAGG

TTATTTTTGGAGGGGAAACTATGCGTTTTTGGGACCTTCGTGCTCCATGGTTAGAACCTC

TAAGGGGGCCCAACGGTTTGGACTTGAGTAGGTTGAAAAAAGACATACAACCTTGGCAAG

AACGACGTTCGGCAGAATATATGACCCATGCTCCTTTAGGCTCTTTAAATTCCGTGGGTG

GCGTAGCTACCGAGATCAATGCAGTTAATTATGTCTCTCCTAGAAGTTGGTTAGCGACCT

CCCATTTTGTTCTAGGATTCTTCTTTTTTGTGGGCCATTTGTGGCATGCAGGAAGAGCCC

GGGCTGCTGCAGCAGGCTTTGAAAAGGGAATCGATCGTGATTTGGAACCTGTTCTTTACA

TGAACCCTCTTAACTAAGATTTTTTATTTATAGCTGTTCTAGTTTTTTTCTGTTCTGGCT

CGGTTATTCCATCTAGCCGAGCCATTCATTCCTTAAAAACAAATAAAGAAACAAACGTAT

TCAATAAGCAAAAGGAGAGAGAGGGATTCGAACCCTCGATAGTTCCTAAAACTATACCGG

TTTTCAAGACCGGAGCTATCAACCACTCAGCCATCTCTCCACAGCCTAATCCCTATTTTA

TTCCTACAAATAGAACATAGCCATATGAAATGATCTACTAACCCATCTCAGATGCAAGTC

CCCTTTCGATATATCTCTGTATAAGGTGGTAAGTAATAAGTTTTAAAGAGAAGAATCAAT

GGATTCATGATTAAACCCCTCCTACTTCTTGTATTTTATTACAATTTTGGTTAAGTGAGG

GATCAAATATGTAGTCAACTTTATTTGATGGTAGCTTGGAGGATTAGAAATATGACTATT

GCTTTCCAATTAGCTGTTTTTGCATTAATTGCGACTTCCTCAGTGTTAGTAATTAGTGTA

CCCCTTGTATTTGCTTCTCCTGATGGTTGGTCAAATAATAAAAACGTTGTATTTTCCGGT

ACATCATTATGGATTGGACTAGTCTTTCTGGTAGCTATTCTGAATTCTCTCATTTCTTAA

ATTTGTTTAGTATTTAGTAGCCCGATACAAAATATAAAAAGGCCATTTCTTCGAATTGTG

AGACGCATTAAAATGCAATTTGCGTTCCGAATTGATTGACAGACAATTAAAAAAAGAAAA

CTCTAATAGAAAATGAAACGGTCGACCCAGACATAGACGGTCGACCCAGGCGGATATACC

CTATAAAATATATCCCGTAGCGAGCGTAGTTCAATGGTAAAACATCTCCTTGCCAAGGAG

AAGATACGGGTTCGATTCCCGCCGCTCGCCAGCTTAATTTAGTAAGGTACTATGATAAAA

AATTTAGTCTACTTATATTAAATTAATAGGTGTTAGTCTAGTACCGTATCCCTTACTATC

TTACCCTCTTTTGCACCCCACTCAAAAAAAGGGGCTCCGGAGGCGGGAATCGAACTCGCC

AACAGGGCTCCCTAAATTGGGGATTCACCGAGACAAACAACTGGCAAACTCTTTTAAAGG

GGTAGACTGTGCCTTTCTTTCATTTCTTTTTTCTTTTCTTCTTCTTGCTAATGAATAAAA

AGGGTTGGATCCAGCCCTCTACTCTATACAAATAGAATAGTCCTTTTATACAGACTGCTA

AGTGCGGAGACGGGAATCGAACCCGTGACCTCAAGGTTATGAGCCTCGTGAGCTACCAAA

CTGCTCTACTCCGCTCTGGAGGGACGGAAACTGGTGGACGAAAAAGGTTGAATACAGGAC

TCTACCATGTCTAGACAAATAGAATAGTCCTTTTATACAGAATGGAGCGGGTAGCGGGAA

TCGAACCCGCATCGTTAGCTTGGAAGGCTAGGGGTTATAGTCGACGTTGGTTGATTAGTT

TTAACGTCTCTAATTCAAAACCGAACATGAAATTTTGATTTCATTCGGCTCCTTTATGGA

TATTCTCACCACTTAACATCTATGTCAGCTTTTCTATCTGAATGGAACCAAAGCTCTCCG

CTTTCTAGATGATCCCTATAGAGTAGGAGATAGAAATTCTACTAAATCTATCTAATCTAC

TTACTTCGTTCCCTAATTTCATTCAAGAGATCCTGAGGAAAAGAATTAGGTTTCCACCGA

GCTGAAACAATATGCTGATGGTTCTAGTAAACCAAAACTACCGTTTTTTAGCTATTTGGC

TTCCATTTCCTTTTTAACAAAAGAAGATTTAGTTACGATTGGAAATAAACTTTTTTGTAT

CTTCATCCATAGATCCTTTACTCATATTTTAAAAATTGGAATACTTAATCCAATGCAAAA

TTATGCTTCGCGACTCTGTACTCATAATCCAATTTGTATTTTGGATGCAATTTCAATTAG

TTTTTGGGTACAAATCGCGAGAATGTATATTCTTCCTCAATATGCTATTGAGAGGAAAAG

GATTAAATCCTTTATAAGAACTAAAGTTTTCATCGGAATATAAAAAACTTAAGGACGCCT

TAAGTATATCATTTCAAATTCAGTTATTAATAGAACGAATCACACTTTTACCACTAAACT

ATACCCGCTACATGTAGATTATGATACCAACGCTACCCTTTGTCAAGGGTAGCCATTCGA

GAAGGAGGCTAATTCCCCCTTATTGAATCAAAGGAGAAGGTTCATGACAGTGAGCTGTTG

GTACTTCGATCGCGGGCCTTTATTTCTTTCTTTTTTGTTCAATTCTGAACAAAGAAATTG

GGGAAGATGTTTTCTTCCCCCACTTATCATGAAGTCCGAGCCCTAGAGAAAGAGTGAGAT

GCTTTTAAAAATTCATCATAGACTTTCCCTATGGCTTGAGAGAAGCAAGAAATAACTTAA

ACGGAGAAGCGGACAGGACCCGCTGGTTAGTCGATCCTCTCCATTTACCAATTTCTTCTC

CTCTTTTCCACTCAATTCTAGTTTATTAGATTCTTGTTTAAAAGAATCAAAGAAGATGAA

TAGAACTAAGAACACATAAAAAAAGCATAGAGGACCATTACCAAATGTTCCTCCCAAGAA

TCATATTGGGTATCTGTTCCCTTCCTTTTCCCGCTAGGATCGGGAATCTAGAATCCTCCT

TTTTCCTAATCTCGGAAACAGAAAACCCATAGCCAGGAGCAGTATAAATTCGACTGCCCA

CTTTTTACAAGCAAATTGTTGCTAAAACTCCAACATAGTTTGTTCAAAATGCACCAGAAT

CCTTTGAGAATATTCAAGTACCCCCCTTCCAAGGGGTACCTGTTAAAAATAGTTTCAATT

TCTCACCAAAACAGACAAGAAGTATATCACTGAAAATTAATACCCAACCGTATGGGTATA

TGAAGAGCGCGAATTCCTTTATACCCTACCCAATTAGAAGAAATAAAACATAAATGGAGA

AAGTTCTCATCATAAGATCAAAAAAAACCTCTACTTTGTGCAAGTGATAAGAGAGAATGA

AATTCTTATTTTTCTTGATTTTTTTGAACCTCGCCATGAATAAACTTCTATATCTCGATA

TATACATATATTATGTACATTATGCAGTAGACTCATAATGAGAAATCAAAGTGGCTAATT

ATTGAATATCATAAAGGGCTTTTTATTTGGTGGTAGAGTAATGCCATGGTAAGACGTAAG

TCATCGGTTCGAATCCGATAAAGTACTTTTCTACTAAATTTATTCATTTCTTTTTTGAAA

ATTTCTCTTTTTTTCTTGAATTTTATGACTTAGTGTGGGATGCATGCATTTTTGGTCTGA

ACGCTAAACGAGCACGGGGTGGAAATTACAAAAAAGAAATTGGACTCTAGATCAATCAAT

ACCTGTACTGAACTAATCTAGAATAATCTATTCTTATTCTATACCCTTTAAATGAATTTC

CCTAAAAAGTAGGGAATGATCCGTGAATTAACCTAACCATCAACTAAAAAAAATCCTATG

AAAGCATAACAGAAAAGTAGGAAAGACTCTTTGCTTGATCTAGTTCTTCGAGTATATGAC

AATTCCAAAAAACTGCTCATACTATCATTATAGTATAATGACGAGCGGTTGTATATGGCC

CTATCGTCTAGTGATGCCCCTATCGTCTAGTGGTTCAGGACATCTCTCTTTCAAGGAGGC

AGCGGGGATTCGACTTCCCCTGGGGGTAGGGAGTATTATGAAAGGAGGTTAATCATAGAT

TCTAAAAAACCCTAGAATAAATTCTTCCTGGGTCGATGCCCGAGCGGTTAATGGGGACGG

ACTGTAAATTCGTTGACGATATGTCTACGCTGGTTCAAATCCAGCTCGGCCCAAAAATCT

AGGGCTTCGTGAATATGAACTAAATCCATTTTTTTCTTCCATAAAATAAAATGTCTGATC

CATAGAAATAAAGGATAAAGCGAAAGGGGGAAATTTCTTTCTAATCCATATCTCTCTCAT

TCCTTTTTTACAAACAAAAGAGTTTTTCTTATTGAAGGTGGATTATCATCCATTTTTAGC

GATAAAAAATCGCGACATACTAGTTATGTCACTCTCACTATACCCACATATGATATGTGG

GTATGTAGTATATGATCCGTCTATTTTTAGAGTACGACAGGCGAATCGAATCTTCTATTT

AGGTATGCCATACACCCCGCGGGGATTGTAGTTCAATTGGTCAGAGCACCGCCCTGTCAA

GGCGGAAGCTGCGGGTTCGAGCCCCGTCAGTCCCGAACTAGGGTTCAATGAATGGAGAAA

TTCATCTTTCCTTTTTCCATGAAAAAGGGGGGGCAGGAGAGAAGATCAAATACCTATGGG

GCACCCTTATTTCACTTTTTTATTTCGCATTTCTCATTAAGAGGGAGGGGTATAGGATTT

TTTTTCACTACTCCCGGTTGATAAGGAAAGACATACATATCATACTTGGAGGATCTTCCT

ATGTTATACTATTCCACTCTCAACCATGAATTGATTTGATAGATCCGATATTCATAATAT

TGAATTGATTCAGTATTATCAGAATGCAAGTCCTCCCCTTGAATTTACAGGATACCCTTT

TTCCTCTCCATGGGATTACATCCCGAGTTATTGTGAAAAAAAAAGAGGTTATGGAAGTCA

ATATTCTCGCATTTATTGCTACTGCACTGTTTATTCTAGTTCCTACTGCCTTTTTACTTA

TTATTTATGTAAAAACAGTCAGCCAAAATGATTAATTGGAATTCCAATTAATCATTGAAG

AAATGAAAAAGGGATTAAATAAAATAAAAATCCAAGTCTTAAATGAAAGGATCTGGTTGG

AATCATAAAGTGTGGTAGAAAGAACTACATATAGTTTTTTCTACGACACTTTAGAGTCTT

TCTATTATATTATCTTGAATCTACATAGAATAGATTAGTAGATTGAAATAGTAGTCTAAT

TCAATTTCTTTTTTCACTGCATCCACTTAATTTCAATCAAGTCAAAATGAAAGAATCCAT

GGAGGGAGAGAAAAATAATATGAGAATAGACTATAGAAAAGAAAAAAGTAAAAGAAAAAA

CCAGCGAATCTTTCATGCTTAAACATGCGGCGAGATGCTTCAAAAGAGCATAAAAATTAT

TTAAGAATAAGAAAAGAATATAAATGGAAAGTGTGCGATATGTTGTGAATAGCTCCGTGG

AAGAAAGTCTAATTTTCTTATGTATAGAACTTTTTTAACCATTCGTCACTTCTAGTAGAA

ATTTTGAATTGCTGTAATCGCTCTTTCTATTTCTATATAGTAGAATAGAACGACTCTTTC

TTACAAGAGTTTCTTACAGGAGTGAAACAAAACTAAAGAAAGAAAGAATCAAGTTTGGCA

AAATGATTAATGCAAAACGATCAATTAAAGAAAAAAGTTGATACAACAATTCGACTACTC

AATCAATTAGTAGTATCCCTAGAGTCCACTCCTCCCCCATACTACTAGTGAAAGAGAAAA

TGTAAAGACTACCATTAAAGCAGCCCAAGCGAGACTTACTATATCCATGTAAATTATGTC

TCCTATTTCTATGAAGGAATTATTCTACTATTGATCAATAATCATAGTGGAATCAAGGGT

ACAGAGTCAAAAAGGGATTCTGCCCTAACGCTATGGATGAATCAGTTCAAGGAATTTACT

CCTAACAAATTCTTATAGGATTTCTGGTAGAATTGGAGAGCATTAAGTATAAATACGATA

CATAGCCCTTTCTATTAATAAAAGAATAAGGAAACGCAACCTCATCCTTATTGGTAGCCG

TTTGGGCCACTACCGACAAAACAAACCCTAATAGAACTATGGATTCTCAAAATCCAGTAT

CGCCAGGCCTAGTTACTCTCTTGCCCCAACTTAGCAGGGTACGAATTTGTTGAGTTCGAT

CAGTACTATAAGCCTAAGTATTTTATTGATCAGGCGGCACCCAGATTTGAACTGGGGATA

AAGGATTTGCAGTCCCCTGCCTTACCGCTTGGCCATGCCGCCAAAAAATCCGATCTAAAA

TAGAGAAAAGAGCAAGTATTCATCCAGGTTTCTTACTAAAACCTCCTTTCTTTTATCTTG

AATCTAATTCTACTTACTTTTTTCCAATCTTTTTCAAAAAAATTCCTGCTTTTTTGAATC

CAGTTTCGATTATTCTCCTCGATGGATTCTATCTTAAAACAAACATTGCTAACACTAGAA

AACTTCCCTTTTCTTTCTATTGAGATGAAAAAAGAGAAAAGTGGATTTCCAGTCACAGGC

TGCAAAATTCATAACAAATTGGAACCATTAACTAGAATTCTATTTTTTGAATTTCGGTAT

TCTCCCCCCTTCCTTTTAATGGCATAATAAAATAGAATGAATTTATGCCTAATCCGTGTA

TAGGTAAACTACAGGTCCGAACAGCATTATTATCCATGGATCCCCCTTATGTACATATCT

CTATGGGGAATCGTGCTTTAATTTTTCATTGCATTAAATATCTTGAATAAAAAAAAGAAA

TTTGGTCTGATATGGAGAGGTGGATAACTAGATTGGCATGTACTTAAAAAAGGACTTACT

TTATTTTAGGATTCTACAATGAAATCCTATATTTTCTAGCAATTCTACTACTACGAAACA

AAAAAGAACCCTCAAATTCTTTTTAAAGGGGATAAAATGAGAAATCTTTGCCATCCAATC

TGATTATATCATTAAGTGGCAGAATTTTTTTCTAGGAATGTTTTATCAATTCATTTTCAT

TCGATTTGTACCCCTGGCAAATTCGAACTTTCGTCGAAATTGTCTCTATTCATATGTATG

AAATACATATATGAAATATGTATGTGGAGTTCCCTAGAATTTCATGTGATTCAGTAAACA

GAATATGGATTCCATAATTGCTAGATCGATCCATAGGGATTGATGAAGAGTGAGCTGATA

ATGGAATTTTTCTTCGATAAACAGGAAACTTAAGATGCTCCGGAATGGAAATGAGGGAAT

GTCCACAATACCCGGATTTAGTCAGATCCAATTCGAGGGATTTTGTAGGTTCATTAATCA

AGGCTTGGCAGAAGAACTTGAGAAGTTTCCAACAATTAAAGATCCAGATCACGAAATTGC

ATTTCAATTATTTGCGAAAGGATATCAATTGCTAGAACCCTCGATAAAAGAAAGGGATGC

TGTGTATGAATCACTCACCTATTCTTCCGAATTATATGTATCTGCGAGATTAATTTTTGG

TTTCGATGTGCAAAAGCAAACCATTTCTATTGGAAACATTCCTATAATGAATTCCTTAGG

AACCTTTATAATAAATGGAATATACCGAATTGTGATCAATCAAATATTGCTAAGTCCTGG

TATTTACTACCGCTCGGAATTAGACCATAAGGGAATTTCTATCTACACTGGGACTATAAT

ATCAGATTGGGGAGGAAGATCGGAATTAGCAATTGATAAAAAAGAAAGGATATGGGCTCG

CGTGAGTAGAAAACAAAAGATATCTATTCTAGTTCTATCATCAGCTATGGGTTCGAATCT

AAAAGAAATTCTAGATAATGTTTCCTACCCTGAAATTTTCTTATCTTTCCCGAATGCTAA

GGAGAAGAAGAGGATTGAGTCAAAAGAAAAAGCTATTTTGGAGTTTTATCAACAATTTGC

TTGTGTAGGTGGGGACCTGGTATTTTCGGAGTCCTTATGTGAGGAATTACAAAAGAAATT

TTTTCAACAAAAATGTGAATTAGGAAGGATTGGTCGACGAAATATGAATCGGAGACTGAA

TCTTGATATACCTCAGAACAATACATTCTTGTTACCACGAGATGTATTGGCCGCTACGGA

TCATTTGATTGGAATGAAATTTGGAACGGGTATACTTGACGATGACGATATGAATCACTT

GAAAAATAAACGTATTCGTTCGGTTGCGGATCTGTTACAAGATCAATTCGGACTGGCTCT

TGGTCGTTTACAACATGCGGTTCAAAAAACTATCCGTAGAGTATTCATACGTCAATCGAA

ACCGACTCCACAAACTTTGGTAACTCCAACTTCAACTTCGATTTTATTAATAACTACTTA

TGAGACCTTTTTTGGCACATACCCCTTATCTCAAGTTTTTGATCAAACCAATCCATTGAC

ACAAACTGTTCATGGGCGAAAAGTGAGTTGTTTGGGTCCTGGAGGATTGACGGGGAGAAC

TGCAAGTTTTCGGAGCCGAGATATTCATCCGAGTCACTATGGGCGTATTTGTCCAATTGA

CACGTCCGAAGGAATCAATGTTGGACTTACTGGATCCTTAGCTATTCATGCGAGAATTGA

CCACTGGTGGGGGTCCATAGAGAGTCCCTTTTATGAAATATCTGAGAAAGCAAAAGAAAA

AAAAGAGAGACAGGTGGTTTATTTATCACCAAATAGAGATGAATATTATATGATAGCAGC

AGGAAATTCTTTGTCCTTGAATCAGGGTATTCAGGAAGAACAGGTTGTTCCAGCTAGATA

CCGTCAAGAATTCCTGACTATTGCATGGGAACAGATTCATGTTAGAAGTATTTTTCCTTT

CCAATATTTTTCTATTGGAGGTTCTCTCATTCCTTTTATTGAGCATAATGATGCGAATCG

GGCTTTAATGAGTTCTAATATGCAGCGCCAAGCAGTTCCGCTTTCTCGGTCCGAGAAGTG

CATTGTTGGAACTGGATTGGAACGCCAAACAGCTCTAGATTCGAGGGTTTCCGTTATAGC

CGAACGCGAGGGAAAGATCATTTCTACTGATAGTCACAAGATCCTTTTATCAAGTAGTGG

GAAGACTATAAGTATTCCTTTAGTTACCCATCGGCGCTCTAACAAAAATACTTGTATGCA

CCAAAAACCTCGGGTTCTGCGGGGTAAATCCATTAAAAAAGGACAAATTTTAGCGGAGGG

AGCTGCTACAGTTGGTGGGGAACTTGCTTTAGGAAAAAACGTATTAGTAGCTTATATGCC

ATGGGAAGGTTACAATTTTGAAGACGCAGTACTAATTAGCGAACGTTTGGTATATGAGGA

TATTTATACTTCTTTTCACATCCGAAAATATGAAATTCAGACGGATACGACAAGCCAAGG

CTCCGCTGAAAAAATTACTAAAGAAATACCACATCTAGAAGAACATTTACTCCGCAATTT

GGACAGAAATGGAGTTGTTAGGTTGGGATCCTGGGTAGAAACTGGCGATATTTTAGTAGG

TAAATTAACGCCTCAGATAGCGAGCGAATCGTCGTATATCGCGGAAGCTGGGTTATTACG

GGCCATATTTGGCCTTGAGGTATCCACTTCAAAAGAAACTTCTCTCAAACTACCTATAGG

TGGAAGAGGGCGCGTTATCGATGTGAAATGGATCCAGAGGGACCCCCTAGACATAATGGT

TCGTGTATATATTTTACAGAAACGCGAAATCAAAGTTGGGGATAAAGTAGCCGGAAGACA

CGGGAATAAGGGGATCATTTCCAAAATTTTGCCCAGGCAAGATATGCCCTATTTGCAAGA

TGGAACACCTGTTGATATGGTCTTCAATCCCTTAGGAGTACCCTCACGAATGAATGTGGG

ACAAATATTTGAAAGCTCGCTCGGATTAGCCGGGGATCTGCTAAAGAAACATTATAGAAT

AGCACCCTTTGATGAGAGATATGAGCAAGAGGCTTCAAGAAAACTTGTGTTTTCAGAATT

ATATGAAGCCAGTAAACAAACAAAAAATCCATGGGTATTTGAACCCGAGTACCCGGGAAA

AAGCAGAATATTTGATGGAAGAACAGGAGACCCCTTCGAACAACCTGTTCTAATAGGGAA

GTCCTATATCTTAAAATTAATTCATCAAGTTGATGAGAAAATCCATGGACGTTCTACTGG

GCCCTACTCACTTGTTACACAACAACCCGTTAGAGGAAGAGCCAAGCAAGGGGGACAACG

AGTAGGAGAAATGGAAGTTTGGGCTTTAGAAGGATTTGGTGTTGCTCATATTTTACAAGA

GATACTTACTTATAAATCTGATCATCTTATAGCTCGCCAAGAAATACTTAATGCTACGAT

CTGGGGAAAAAGAGTACCTAATCACGAGTATCCTCCAGAATCTTTTCGAGTGCTCGTTCG

AGAACTACGATCTTTGGCTCTAGAACTGAATCATTTCCTTGTATCTGAGAAGAACTTCCA

GGTTAATAGGGAGGAAGTTTGATCGGAATAAATATAAATTCTTTTCTTATTTATGATTGA

CCAATATAAACATCAACAACTTCAAATTGGACTCGTTTCCCCTCAACAAATAAAGGCTTG

GGCTAACAAAAACCTACCTAATGGGGAAGTCGTTGGCGAAGTCACAAGGCCCTCTACTTT

TCATTATAAAACCGATAAACCAGAAAAAGATGGATTGTTTTGCGAAAGAATCTTTGGACC

CATAAAAAGCGGAATTTGTGCTTGTGGAAATTCTCGAGCGAGCGGAGCTGAAAACGAAGA

GGAAAGATTTTGCCAAAAATGCGGGGTAGAATTTGTTGATTCTCGGATACGAAGATATCA

AATGGGATACATCAAACTCGCATGTCCCGCGACTCATGTGTGGTATTTGAAAGGTCTTCC

TAGTTATATCGCGAACCTTTTAGATAAACCCCTTAAGAAATTGGAGGGCCTAGTATACGG

CGATTTCTCTTTTGCTAGGCCCAGCGCTAAAAAACCTACTTTCTTACGATTACGAGGTTT

ATTCGAAGATGAAATTGCATCCTGTAACCACAGCATTTCTCCCTTTTTTTCTACCCCAGG

CTTTGCAACATTTCGAAATCGGGAAATTGCGACAGGAGCAGGTGCTATTAGAGAACAATT

AGCAGATTTGGATTTGCGAATTATTATAGAGAATTCCTTGGTCGAATGGAAGGAATTAGA

AGACGAGGGGTATAGTGGAGATGAATGGGAAGATAGAAAAAGACGAATAAGAAAAGTTTT

TTTGATTAGACGCATGCAATTGGCGAAACATTTTATTCAAACAAATGTAGAACCAGAATG

GATGGTTTTGTGCTTATTACCAGTTCTTCCTCCCGAATTGAGACCCATTGTTTATAGGTC

TGGGGATAAAGTAGTGACTTCGGATATTAATGAACTTTATAAGAGAGTTATTCGTCGGAA

CAACAACCTTGCCTATCTATTAAAAAGAAGTGAATTAGCGCCAGCAGATTTAGTAATGTG

CCAGGAAAAATTGGTACAAGAAGCCGTGGATACACTTCTTGATAGTGGGTCCCGCGGGCA

ACCAACGAGGGATGGTCACAATAAAGTATACAAATCACTTTCAGATGTAATTGAAGGTAA

AGAGGGAAGGTTTCGCGAGACTCTGCTTGGAAAACGGGTCGATTACTCGGGGCGTTCTGT

CATTGTTGTGGGTCCTTCGCTTTCATTACATCAATGTGGATTACCTCTAGAGATAGCAAT

AAAGCTTTTTCAGCTATTTGTAATTCGCGATTTAATCACGAAACGCGCTACTTCTAATGT

CAGGATTGCTAAAAGGAAAATTTGGGAAAAGGAACCCATTGTATGGGAAATACTTCAAGA

AGTTATGCGGGGACATCCTGTACTGTTGAATAGAGCACCTACCCTGCATAGATTAGGCAT

ACAGGCCTTCCAACCTACTTTAGTGGAGGGGCGTACTATTTGTTTACACCCATTAGTGTG

TAAGGGTTTCAATGCGGACTTTGATGGGGATCAAATGGCTGTTCATCTACCTTTATCCTT

GGAAGCTCAGGCGGAAGCCCGTTTACTTATGTTTTCTCATATGAATCTCCTATCTCCCGC

TATTGGGGATCCTATTTGCGTACCGACCCAAGACATGCTTATCGGACTTTATGTATTAAC

GATTGGAAACCGTCGGGGTATTTGTGCAAATAGATATAATAGTTGCGCAAACTATCCAAA

TCAAAAAGTAAATTACAATAATTATAAGTATACAAAAGATAAAGAACCCCATTTTTCTAA

TTCCTATGATGCACTGGGAGCTTATAGACAGAAACGAATCAGTTTAGACAGTCCCTTGTG

GCTCCGATGGAAACTAGATCAACGCGTCATTGGGTCAAGAGAAGTTCCGATTGAAGTTCA

ATATGAATCTTTGGGGACTTATCATGAGATTTATGCCCACTATCTAATAGTGGGAAATAG

AAAAAAAGAAATCCGTTCTATATACATTCGAAGCACTCTTGGTCATATTTCTTTTTATAG

AGAAATAGAGGAAGCCATACAAGGATTTAGTCAGGCCTATTCATACACTATCTAAACAAG

GAAGTTAGATTCGGCGATGCCTTTCGGGGGGCATTCCGATTTCGCTAGTATCATCATTTT

TGCCGCGCGAATCCAGATTGAGATTAAGGAAAGGAAGTTAATTAAATTTTGAATCACTGA

CTCAGGCCCATTGTCGAATCCTACTCAGCAATTGTCGAATCCTACTCAGCCGAAAAAGGG

GGTACTTATGTATGGCGGAACGGGCCAATCTGGTCTTTCATAATAAAGAGATAGACGGAA

CTGCTATGAAACGACTTATTAGCAGATTAATAGATCATTTCGGAATGGGATATACATCCC

ATATACTGGATCAAATAAAGACTCTGGGCTTTCATCAAGCCACTACTACATCGATTTCAT

TAGGAATCGAGGATCTTTTAACAATACCATCTAAGGGATGGTTAGTGCAAGACGCGGAAC

AACAGAGTTTTCTTTTGGAGAAACACTATTATTATGGGGCTGTACACGCGGTAGAAAAAT

TACGCCAATCCGTTGAGATATGGTATGCTACAAGTGAATATTTGAAACAAGAAATGAATT

CGAATTTTCGGATAACGGATCCTTCTAATCCAGTCTATCTAATGTCTTTTTCAGGAGCCA

GAGGAAATGCATCTCAGGTACACCAATTAGTAGGTATGAGAGGATTAATGGCGGATCCTC

AAGGACAAATGATTGATTTACCTATTCAAAGCAATTTACGCGAGGGACTTTCTTTGACAG

AATATATAATTTCCTGCTACGGAGCCCGCAAAGGGGTTGTAGATACTGCTGTACGAACAG

CGGATGCTGGATATCTTACACGTAGACTTGTTGAAGTAGTTCAACATATTATTGTGCGTA

GAAGAGATTGTGGTACTATCCAAGGTATTTCTTTGAGTCCTCAAAATGGGATGACGGAAA

AACTTTTTGTCCAAACACTAATTGGTCGTGTATTAGCAGACGATATATATATTGGTTCAC

GATGCATTGCCGCTCGAAATCAAGATATTGGAATTGGATTAGTCAATCGATTCATAACTG

CCTTTCGAGCACAACCATTTCGAGCACAACCAATATATATTAGAACCCCCTTTACTTGCC

GGAGCACATCTTGGATCTGTCAATTATGTTATGGTCGGAGTCCCACTCATGGCGATCTGG

TCGAATTGGGGGAAGCCGTAGGTATTATTGCAGGTCAATCAATTGGGGAGCCAGGGACTC

AACTAACATTAAGAACTTTTCATACTGGCGGAGTATTCACAGGGGGTACTGCCGACCTTG

TACGATCCCCTTCGAATGGAAAAATCCAATTCAATGAAGATTTGGTTCACCCCACACGTA

CCCGTCATGGGCAGCCTGCTTTTCTATGTTATATAGACTTGCATGTAACTATTCAGAGTC

AGGATATTCTATATAGTGTGAATATTCCCTCAAAAAGCTTGATTCTAGTGCAAAATGATC

AGTATGTAGAATCCGAACAAGTAATTGCGGAGATTCGTGCCGGAACGTCCACTTTGCATT

TTAAAGAAAAAGTACAAAAGCATATTTATTCCGAATCAGACGGGGAAATGCACTGGAGTA

CTGATGTTTACCATGCGCCCGAATATCAATATGGTAATCTTCGTCGATTACCAAAAACAA

GCCATTTATGGATATTGTCAGTAAGTATGTGCAGATCCAGTATAGCGTCTTTTTCGCTCC

ACAAGGATCAAGATCAAATGAATACTTATTCTTTTTCTGTTGACGGAAGATATCTCTTTG

ACCTCTCAATGGCTAATGATCAAGTAAGACATAGACTGTTGGATACTTTTGGTAAAAAAG

ATAGGGAAATTCTTGATTATTCAACGCCGGATCGAATCATGTCCAATGGCCATTGGAATT

TTGTCTATCCTTCTATTCTTCAAGATAATTCGGATTTGTTGGCGAAAAAGCGAAGAAATG

GGTTCGTCATTCCATTACAATATCATCAAGAACAAGAGAAAGAACTAATATCCTGTTTGG

GGATTTCGATTGAAATACCCTTTATGGGTGTTTTACGTAGAAATACTATTTTTGCTTATT

TTGACGATCCACGATACAGAAAAGATAAAAAGGGTTCAGGAATTGTTAAATTTAGATATA

GGACCCTAGAGGACGAATATAGGACTCGAGAGGAAGACTCAGAGGACGAATATGGGACAG

AGAACGAATATAGGACCCGAGAGGAAGAGGACGAATATGAAACCCTAGAAGATGAATATG

GGATCCTAGAGGACGAATATGAAGCCCTAGAAGACGAATATAGGACTGGAGAGAAAGACT

CAGAAGACGAATATGGGAGCCCAGAGAACGAATATAGAACCCGAGAGGACGAATATGGAA

CTCTAGAGGAAGACTCAGAGGACGAATATGGGACTTTAGAGGAAGACTCAGAAGAAGACT

CAGAGGACGAATACGGGAGCCCAGAGGAAGATTCCATCTTAAAAAAAGAGGGTTTGATTG

AGCATCGAGGAACAAAAGAATTTAGTCTAAAATACCAAAAAGAACTAGATCGGTTTTTTT

TCATTCTTCAAGAACTGCATATCTTGCCGAGATCCTCATCCCTAAAGGTACTTGATAATA

GTATCATTGGAGTGGATACACAACTCACAAAAAATACAAGAAGTCGACTAGGTGGATTGG

TCCGAGTGAAGAGAAAAAAAAGCCATACGGAACTCAAAATCTTTTCCGGAGATATGCATT

TTCCTGAAGAGGCGGATAAGATATTAGGTGGTAGTTTGATACCACCAGAAAGAGAAAAGA

AAGATTCTAAGGAATCAAAAAAAAGGAAAAATTGGGTCTATGTTCAACGGAAAAAAATTC

TCAAGAGCAAGGAAAAGTATTTTGTTTCGGTTCGACCTGCAGTCGCATATGAAATGGACG

AAGGGAGAAATTTAGCAACACTTTTCCCGCAAGATCTCTTGCAAGAAGAGGATAATTTCC

AACTTCGACTTGTCAATTTTATTTCTCATGAAAATAGCAAGTTAACTCAAAGAATTTATC

ATACGAATAGTCAATTTGTTCGAACTTGCTTAGTAGTGAATTGGGAACAAGAAGAAAAAG

AGGAGGCTCGTGCTTCCCTTGTTGAGGTAAGAGCAAATGATCTGATTCGCGATTTCCTAA

GAATTGAGTTAGTCAAGTCCACTATTTCGTATACACGAAGAAGGTATGATAGGACAAGTG

CAGGACCGATTCCCAATAATAGGTTAGATCGCACCAATTCCTTTTATTCCAAGGCGAAGA

TTCAATCACTTAGCCAACATCAAGAAGCTATTGGCACCTTGTTGAATCGAAATAAAGAAT

ACCAATCTTTGATGATTTTGTCGGCATCCAACTGTTCTCGAATTGGTTTATTCAAGAATT

CGAAATATCCCAATGCGGTAAAAGAATCGAATCCTAGAATTCCTATTCGAGATATTTTTG

GGCCCTTAGCCGCTATTGTACCTAGTATATCGAATTTTTCTTCATCTTACTATTTACTAA

CGCATAATCAGATCCTGTTAAAAAAATATTTGTTCCTTGACAATTTGAAACAAACCCTCC

AAGTACTTCAAGGGCTTAAATACTCTTTAATAGATGAAAATCAAAGGATTTCGAATTTCG

ATAGTAACATCATGTTGGATCCATTCCATTTGAATTGGCACTTTCTCCATCATGATTCTT

GGGAGGAGACATCGGCAATAATTCACCTTGGACAATTTATTTGCGAAAATGTATGTCTAT

TTAAATCGCACATAAAAAAATCTGGTCAAATTTTCATTGTTAATATTGATTCCTTTGTTA

TAAGAGCAGCTAAGCCTTATTTGGCCACTACAGGAGCAACTGTTCATGGTCATTATGGAG

AAATCCTTTACAAAGGAGATAGGTTAGTTACGTTTATATATGAAAAATCGAGATCTAGTG

ACATAACGCAAGGTCTTCCAAAAGTAGAACAAATCTTTGAAGCGCGTTCAATTGATTCAC

TATCGCCGAATCTCGAAAGGAGAATTGAGGATTGGAATGAGCGTATACCAAGAATTCTTG

GGGTCCCCTGGGGATTCTTGATTGGAGCTGAGCTAACCATAGCCCAAAGTCGTATCTCTT

TGGTTAATAAGATCCAAAAGGTTTATCGATCCCAAGGGGTACAGATCCATAATAGACATA

TAGAGATTATTATACGCCAAGTAACATCAAAAGTGCGGGTTTCCGAAGATGGAATGTCTA

ATGTTTTTTCACCTGGGGAATTAATCGGACTATTACGAGCAGAACGAGCAGGACGAGCTT

TGGATGAATCGGTCTATTATCGGGCAATCTTATTGGGAATAACAAGGGCTTCCCTGAATA

CCCAAAGTTTCATATCTGAAGCAAGTTTTCAAGAAACTGCTCGAGTTTTAGCAAAAGCTG

CCCTACGAGGTCGTATTGATTGGTTGAAAGGCCTGAAAGAAAACGTAGTTCTGGGGGGGA

TTATACCTGTTGGTACCGGATTCCAAAAATTTGTGCATCATTCCCCACAAGACAAGAACC

TTTATTTCGAAATTCAAAAAAAAAATCTATTCGCGTCGGAAATGAGAGATATTTTGTTTC

TCCATACAGAATTAGTTTCTTCTGATTCTGATGTAACAAACAATTTCTATGAGACATCAG

AACCCCATTTATACGATTTAAGGATACATAAAGCAGATTTTTTTATTTAAACTAGACTTT

TGACCTTAGAACACTAACAGGTCAGATTTTGATTTTTATTAATAAGTAAAGAAGTCAGTT

AATTCATTAAGGTTACGTTTATACCATGTAGAAGGTTACATCGGAACAATTATTATTTAT

TTCAAGCTATTTCGGCTCTTTCTTAATTTTCAAAAAGAAATAAATTCCGTAATGGAAAAA

AAAGAAAAAATCAAAAGGAAGTGTGGAAAAAATGACAAGAAGATATTGGAACATCAATTT

GAAAGAGATGATAGAAGCGGGAGTTCATTTTGGTCATGGTATTAAGAAATGGAATCCTAA

AATGGCCCCTTACATCTCGGCAAAGCGTAAAGGTACTCATATTACAAATCTCGCTAGAAC

GGCTCGTTTTTTATCAGAAGCTTGTGATTTAGTTTTTGATGCAGCAAGTCAGGGAAAAAG

CTTTTTAATTGTTGGTACCAAAAAAAGAGCAGCGGATTTAGTAGCATCAGCTGCAATAAG

GGCTCGTTGTCATTATGTTAATAAAAAGTGGTTCAGTGGTATGTTAACGAATTGGTCGAT

TACGAAAACTAGACTTTCTCAATTTAGAGACTTAAGAGCAGAAGAAAAGATGGGAAAATT

CCACCATCTCCCAAAAAGAGATGTGGCAATCTTGAAGAGAAAATTATCTACCTTGCAAAG

ATATCTCGGCGGGATCAAATATATGACGAGGTTGCCGGACATTGTGATCGTCCTTGATCA

GCAAAAAGAGTATATAGCTCTTCGGGAATGTGCCATTTTGGGGATTCCTACTATTTCTTT

AGTCGATACAAATTGTGACCCAGATCTCGCAAATATATCGATTCCAGCCAACGATGACAC

TATGACTTCAATTCGATTGATTCTTAACAAATTAGTATTTGCAATTTGTGAGGGCCGTTC

TCTCTATATAAGAAATCGTTGATTAAGAAGAATAGTTCATTCTTGGGTAACTGCGTAGAT

TTATGGGATCACTTACTATTCTTTTTTGTTTTGCATAGATAAAAGAAGGGGAATATTGAT

ATATATTAGAGGGTATTGATATATATTATCATCTGATGTGATTTCTTGGTATCCTAAATA

TAAGATTAATACTTCAAGTTGCTGAGTTGAGAAAGAGATGGTTGAATCAAAAGAATTCCT

TTTTTGAAGTTCAATTTTTATCAGAGGACAATATGAATATTATACCATGTTCCGTTAAAA

CACTCAAGGGGTTATATGATATATCGGGTGTAGAAGTAGGCCAACACTTCTATTGGCAAA

TAGGAGGTTTCCAAATTCATGCCCAAGTACTCATCACTTCTTGGGTCGTAATTACTATCT

TGCTAGGTTCAGTTATCATAGCTGTTCGGAATCCACAAACCATCCCGACCGACGGTCAGA

ATTTCTTCGAATATGTGCTTGAGTTTATTCGAGACTTGAGCAAAACTCAGATTGGAGAAG

AATATGGTCCCTGGGTTCCCTTTATTGGAACTATGTTCCTTTTTATTTTTGTTTCGAATT

GGTCGGGTGCTCTTTTACCTTGGAAAATTATACAGTTACCCCATGGGGAATTAGCAGCAC

CCACGAATGATATAAATACTACTGTTGCTTTAGCTTTACTCACATCAGCGGCATATTTTT

ATGCGGGTCTTAGCAAAAAAGGATTGAGTTATTTCGAGAAATATATTAAACCAACTCCAA

TTCTTTTACCAATTAACATCCTAGAAGATTTCACAAAACCATTATCGCTTAGTTTTCGAC

TTTTCGGGAATATATTGGCGGATGAATTAGTCGTTGTTGTTCTTGTTTCTTTAGTCCCCT

TAGTAGTCCCTATACCGGTCATGTTTCTTGGATTATTTACAAGCGGTATTCAAGCTCTTA

TTTTTGCAACGTTAGCCGCAGCCTATATAGGTGAATCCATGGAAGGTCATCATTGAATTG

ACTAGTTTTCAAAATAGTCTTTTTTTAGCTTAGCTCAATTCATGCATGGTTCCAGATAAT

CCGCTTGGTTGGAAAACTAAATAGTTAGAAATGCGTATGAATATACAACCTAGAGTTGTA

GGAGAGAGAATAGACTATATTACGTGTCAAAGTATATATGCATTAAGGGGGGCGGAGTCA

GGCTAGATCTATATCCTTAATGTCTATAAGCCAGTCATCTTTTGTGCGGGTTTTTAAGGA

ATGATTTTAGAATCCGATTCAATAGAAAATGAGAAAATACGCAAAATAGAAGAAACAAAT

GTATATGGGATATTATATATTCCTAAGTTAGATTCATTATCTAATCCGATATATGGAATT

GGATTCCATATCCAGTTCTATGCAGCATATTGTTATCAATTGTATATCTTGATTTAATTC

CTATTGGATTTGGATTAGGTCGATTTCAATAGGGGTTCTTCCTCTATTTCGTCTTTTATT

ATGTTAGATGAAGGGGAAAAAATAGGAACTCAAGGATATCGAAGAGTAAAAAGAAGAATG

GAATGAAAGAGTGGTTGGTTGGAAAGAAAGAGAAATAGAATAATGAGTACACAAACCTCT

AATGATTAGAAACTAAAAATGAGATCTCGAAGTAGTTCGGACAATTCAGATTATCATTTA

TTTGTACTTTTTAGTTACTTCTCCCCAATAGAGCTTAGAAGTAAGAATTTCTTGGTTGAT

TGTATCCTTAACCATTTCTTTTTTTTGACACGAGGAACTCACCATGAATCCACTAATTGC

TGCTGCTCCGTTATTGCTGCTGGATGCCGTAGGGCTTGCTTCTATTGGGCCTGGAGTTGG

TCAAGGTACTGCTGCAGGACAAGCTGTAGAAGGTATTGCGAGACAGCCAGAAGCAGAAGG

TAAAATACGAGGTACTTTATTGCTTAGTCTAGCTTTTATGGAAGCTTTAACAATTTATGG

ACTAGTTGTGGCACTAGCGCTTTTATTTGCGAACCCTTTTGTTTAATCCTAAAAAAGAAA

ATGAGTCCTTTAGATTAGATACTTTTTTCTTTTTTAGTAAATTGGTATTTGCTTCTGCAA

TTCCAATTATATCAATACTTTACTCCTATTTATTACTCCTGGAATTACCTATTTATCGGG

ACAGACAATACCCCACCCCAGGAAGGGCTGATTTGAGGATGATCAATTTAGAGGATATGC

TCGCCTTCTTCCTTCCCGTCCTTAGTTTAGGACAGTGGAAAGTCTTTTTCCTTTTATTTT

AGGAATTTTTGGAACATTTCAACAAAGGAGTCTTTCACAGGTCAAACGAGACCTAAGACT

TAATCTAAAAGAAATTATTAGATTGAATCTATTTGCATTAAAAAACCGATCAAAAAGGGC

GAGCGAAGTAAGTGATCAAAAACTTTGCTCTTTGTTCGTCCTATCTATAAGAGGAGAGCA

TATGAAAAATGTAACCCATTCTTTCGTTTTTTTAGCTCACTGGCCATCCGCTGGGAGTTT

CGGGCTTAATACCGATATTTTAGCAACAAATCTAATAAATCTAACTGTAGTGGTTGGTGT

ATTGATTTTTTTTGGAAAGGGAGTGTGTGCGAGTTGTCTATTTCAAGAATAGATTGATCT

ATCCGGCTGCACTTTAAAATATTTTTTAGTATTTTTTGGATAAATAAGAAAAGGTGCACG

ATCTCGACGAATTACTTCTGAATAAATTCAGAAATCATATGGAAGACCATAGCATTTCGC

GACTCATTGGTAAATCAACTTTGATTCTCTATAGACCAATAATGTGAGACCATTAACACG

GTTAAAGCTAAACTGCTTGAAGTCCAGGCAAAAAGGGGTACTCTTTCTACAACTATATTA

GTATTAGTACCAAATGCTTTAAACGGGAAATAGCTAATGTAGAATTTATCTGATATAAAA

CACTCATATCGATAAAATGGTTTGAACTATTTACTAGAAGGGCACCCTGCCCTTTTTCCA

ATGCCGAATCGACGACCTATGTATAAAAAAAGAGAAATTTTTTGGATTTGAAGAAAAAAA

AGAATTCTATCAATTTTCATTTTCCATTTATTTAGTTTCTTAATGAAATTGAAATTATTA

ACTAAAGGGCAAATACAAATAAAGAAACAACTTTGCTGACCATGATAGATTTTTATCTAG

GCGGAAGAGTCCTCTTAATATTTATCTAGTCTTATATGGGTTTCGGTATATTGAAATATA

AACAGAAAAGAGAAGATAGAGGATAGGCTCATTACATAAAAAAGATATGGAAATAGCCAT

AGCAAAAAAAAAAAAGGAGCGTGAGAGCCAAATGAATCGAAAGATTCATGTTTGGTTCGG

GAAGAGATCATAAAAATTGTAAACTTAATAGCAAGATAATCTACTTTCATTAAAAGATTT

ATTAGATAATCGAAAACAGAGAATCTTGAGTACTATTCGAAATTCGGAAGAATTGCGTAG

AGGGACCATTGAGCAGCTCGAAAAAGCTCGGGTTCGATTACAGAAAGTCGAACTAGAAGC

GGATGAGTATCGAATGAATGGATACTCTGAGATAGAACGAGAAAAAGAAAATTTGATTAA

TGCTACTTCTATTAGTTTGGAACAATTAGAAAAGTCTAAAAATGAAATCCTTTATTTTGA

AAAACAAAGGGCGATGAATCAGGTCCGACAACGGGTTTTCCAACAGGCCGTACAAGGAGC

TCTAGGAACTCTGAATAGTTGTTTGAATACCGAGTTACATTTCCGTACGATTCGTGCTAA

TATTGGCATTCTAGGGGCCATAGAATCGAAGAATTAAATTAATTAGACCTTGAACTTCTA

CTTTCGTTTAGAATTTAGGCATTATTTTTCCCTTGCTTCCGAAAAAAGAGTCAAGAAACA

CTAATGGCAACCCTTCGAGTCGACGAAATTCATAAAATTCTCCGCGAACGTATTGAACAA

TATAATAGGAAAGTAGGGATTGAGAATATCGGTCGCGTAATTCAAGTGGGGGATGGGATT

GCTCGTATTATAGGTCTTGGTGGAATAATGTCAGGTGAATTAGTCGAATTTGCAGAAGGG

ACTAGGGGTATTGCTCTGAATTTGGAATCCAAAAATGTTGGGATTGTATTAATGGGCGAT

GGGTTGATGATACAAGAGGGAAGTTTTGTAAAAGCAACAGGAAGAATTGCTCAGATACCC

GTGAGCGAGGCTTACTTGGGTCGTGTTATAAATGCTCTGGCTAAACCTATTGATGGGAGA

GGCGAAATTGTAGCTTCGGAATCTCGCTTAATTGAATCTCCTGCTCCGGGTATAATTTCC

AGGCGTTCCGTATATGAACCCCTTCAAACAGGGCTTATTGCTATCGATTCGATGATCCCC

ATAGGGCGCGGTCAGCGAGAGTTAATTATTGGGGACAGACAGACTGGCAAAACAGCAGTA

GCCACAGATACAATTCTCAATCAAAAAGGGCAAGATGTAATATGTGTTTATGTAGCTATC

GGTCAAAGAGCATCCTCCGTGGCTCAAGTAGTAACTACTTTCCATGAAGAGGGGGCCATG

GAATACACTATTGTAGTAGCTGAAATGGCGGATTCACCTGCTACATTACAATACCTCGCC

CCTTATACGGGAGCAGCCCTGGCTGAGTATTTTATGTATCGCGAACGGCATACTTTAATA

ATTTATGATGATCTCTCCAAACAAGCACAAGCTTATCGCCAAATGTCCCTTCTATTAAGA

AGACCCCCCGGCCGCGAAGCTTATCCAGGGGATGTTTTTTATTTGCATTCACGGCTTTTA

GAAAGAGCCGCTAAATTAAGTTCTCTTTTAGGCGAAGGAAGTATGACCGCTTTACCAATA

GTTGAGACTCAATCTGGAGACGTTTCCGCCTATATTCCTACTAATGTAATCTCCATTACA

GATGGACAAATATTCTTATCCGCGGATCTATTTAATGCCGGAATTCGACCTGCTATTAAT

GTGGGTATTTCTGTTTCCAGAGTAGGATCCGCGGCTCAAATTAAAGCCATGAAACAAGTA

GCTGGCAAATCAAAATTGGAACTAGCTCAATTCGCAGAATTACAAGCCTTTGCACAATTC

GCCTCTGCTCTCGATAAAACAAGTCAGAATCAATTGGCAAGGGGTCGACGATTACGGGAA

TTGCTTAAACAATCCCAATCAAACCCTCTCTCAGTGGAAGAGCAGATAGCTACTATTTAT

ACCGGAACGAGAGGATATCTTGATTCGTTAGAAATTGGACAGGTAAAGAAATTTCTGGAT

GAGTTACGTAAACACCTAAAAGATACGAAACCTCAATTCCAAGAAATTATATCTTCTAGC

AAGACATTCACCGAGCAAGCGGAAATCCTTTTGAAGGAAGCTATTCAGGAACAGCTCGAA

GGGTTTTCCCTTCAGGAACAAACATAAATTTTGCATGTCTACTCTTGTTAGTAGAAGAGG

AATCAAAGATTTTTCATTTGAATCATGCAAAAAGTTTTCTTAGTTTTTAGTATAGTTATT

TAAAGAATAGATAGAAATAAGATTGCGTCCAATAGGATTTGAACCTATACCAAAGGTTTA

GAAGACCTCTGTCCTATCCATTAGACAATGGACGCTTTTTTCATATTTTCTTCTTTCTTT

TTTTTTTTTTCTTGTGATAAAAAACTCTTAGACCGAAACTCTTTTAGGAAAGAAAAATAA

ATCCATATACAAATGGATGATGCATATATCATAAAGAAGGAATATGGAGCGGGTAGTGGG

AATCGAACCCGCAACCCCAAGGTTATGAGCCTTGTGAGCTACCAAACAGCTCTATCCTCT

TAGCGGGGAACTAGGGGGTTGGATACGCCCCTCTACCATATCTATACAAATAGAATAGTC

CATTTATACAGAATGGTAAAGAGGGCTCTTCTACGATCATCCATTCTAGAAATCCATACA

AATATGAAAGGGTATTTTATCCTTACCAACTGGATCTTGTTGCACCCGGTAACAAACATG

CATAAACCATTTCTCGAAGTATGTGTCCGGATAGTCCAAAGTCTCGATAGTTAGCTCTAG

GTCTTCCGGTCAAAAAACAACGTCGATGAAGGCGTGTAGGTGCACTATTACGTGGTAGGG

ATTGCAATTTTTCTCGCATTTTCGTTTTTTCACTCAAACTCAAGGGGGAAACTTTGCTTC

TTATCTTTTTTTTTGAAGATCGACGAATCAAATGATATTTCTGTTCTAATTTCTGCCGCT

TCTTCTCCCTCTGAATCAAACTTTTTTTTGCCATAATGTGCCGTTCCTATTATTACCAAG

TATATGGTTCTAATCCTAGATGGAAAAATAAATAGAAAAAATCTAAGAAGGCGGATCCTC

CCTCTCCATCAAGAGTAATGAACTAGGTGCTGGTACAGTACAAAACTAAATTAACCAAAC

TTGCCTGATGTTGAGGCAATCAAGAAAGCTGCATAAGTGAATATATAACCCACGGAAAAG

TGGGCTAATCCAACCAATCTTGCTTGCACAATGGAAAGAGCCACGGGCTTATCTCTCCAG

CGAATTAAATTAGCCAAAGGTGTGCGTTCATGAGCCCATGCTAAAGTCTCAATTAATTCC

TGCCAATATCCACGCCAGGAAATTAAGAACATAAATCCAGTAGCCCAAACAAGATGTCCA

AATAAGAACATCCACGCCCATACCGATAAACTATTCATCCCAAAAGGATTATATCCATTG

ATAAGTTGTGAAGAGTTTAACCATAGGTAATCTCTTAACCATCCCATCAAATAAGTGGAG

GATTCATTAAATTGTGAAACGTTGCCCTGCCATAATGTGATGTGTTTCCAATGCCAATAA

AAAGTAACCCATCCAATGGTATTTAACATCCAGAAAACTGCCAAATAAAACGCGTCCCAA

GCAGAAATATCACAAGTACCGCCGCGCCCTGGGCCGTCACAAGGAAAACTATACCCAAAA

TCCTTTTTATCCGGCATTAATTTGGAACCGCGTGCATCTAAAGCACCCTTTACTAAAATC

AATGTAGTTGTATGCAAACCTAGAGCAATAGCATGATGAACCAAGAAATCTCCAGGTCCT

ATTGTTAAGAAAAGAGAATTACTATTCTCATTAACAGCATTCAACCATCCGGGCAACCAT

AGGGTTCGACCTGCATTGAAAGCGGGGCCGCTCGTTGAAGATAAGAGTATATCGAACCCA

TATGTCGTCTTACCATGAGCAGATTGTATCCATTGGGCAAATATAGGTTCGATCAAGATT

TGCTTTTCTGGAGTACCAAAAGCAAGCATGACGTCGTTATGAACATAAAGGCCCAAGGTA

TGGAATCCTAGGAAGAGGCTAGCCCAACTTAAATGAGATATGATAGCTTCTTTATGGTCT

AACATTCTTGCCAATACATTATCCTCATTCTGTTCCGGATTGTAATCCCTAATGAAAAAA

ATAGCTCCATGAGCAAAAGCCCCTGTCATGATGAACCCTGCAATATATTGGTGATGAGTA

TATAAAGCAGCTTGAGTGGTAAAGTCTTGTGCTATGAATGCATAAGCAGGTAAAGAGTAC

ATATGTTGAGCTACTAAGGAAGTAATAACCCCTAAAGAAGCTAGAGCAAGACCTAATTGA

AAATGAATCGAATTATTGATTGTGTCGTAAAGGCCCTTATGCCCACGCCCTAATCGACCC

CCCGGAGGAGTATGTGCTTCTAAAAGATCTTTGATACTGTGCCCAATTCCGAAGTTAGTT

CGATACATATGACCGGCAATGAGAAAAATAAATGCAATAGCTAAATGATGGTGAGCAATA

TCGGTCAGCCACAAACTTTGTGTTTGTGGATGGAATCCCCCAAGAAGAGTTAGAATGGCA

GTTCCGGCTCCTTGAGCGGTACCAAATAAATGATTACTCGAATCAGGGTTTTGGGCATAA

AGATTCCACTGACCCGTCAGAAGGGGTCCCAACCCCTGGGGATAGGGTAATACATCTAAG

AAATTATTCCATCGAACGTACTCCCCCCTGGATCCGGGAATAGCGACATGAACTAAATGT

CCTGTCCAAGCCAAAGAACTTACCCCGAAAAGTCCTGACAAATGATGATTGAGACGAGAT

TCCGCGTTTTTGAACCACGAAAGGCTTGGTTTCCATTTGGGTTGTAGATGTAACCAACCC

CCTATTAAGGATAGCGTAGAAAGAAATAATAGAAACAGAGCTCCAGTATAAAGATCTCCA

TTGGTGCGTAATCCAATTGTATACCACCACTGATAAACCCCAGAATAGGCGATATTCACT

GGACCGGCAGCACCTCCTCGAGTAAAGGCTTCCACAGCGGGTTGACCAAAATGAGGATCC

CAAATCGCATGAGCAATAGGCCTTACGTGTAAAGGATCCTGTATCCATGATTCAAAATTT

CCTTGCCAAGCTACATGAAACAGATTTCCGGATGTCCATAGAAAGATTATTGCTAACTGC

CCAAAGTGAGAAGCAAAAATGTTCTGATAAAGACGTTCCTCAGTAATATCATCATGACTT

TCGAAATCATGTGCGGTAGCAATACCAAACCAAATACGACGAGTAGTGGGGTCCTGAGCT

AAGCCTTGGCTAAACCTGGGAAATCTTAATTCCATAATGCCTTTCAAATCCTCCTAGCCA

CTATCCTACTGCAATAATTCTCGCTAAGAAGAATGCCCATGTTGTGGCAATTCCACCCAG

AAGGTAATGGGTTACTCCTACAGCACGTCCTTGTATAATGCTCAAGGCTCTAGGCTGAGT

AGCAGGAGCAACTTTTAATTTGTTATGAGCCCAAACGATAGATTCAATGAGTTCTTGCCA

ATAACCACGGCCGCTGAATAAAAACATTAAACTGAAGGCCCAGACAAAATGAGCACCTAA

GAAAAAAAGACCATATGCAGATAATGAAGAACCATAAGACTGAATTACTTGGGATGCCTG

TGCCCACAAGAAATCTCGGAGCCACCCATTAATCGTAATGGAACTCTGTGCAAAGTTTCC

CCCTGTGATATGAGTTACCATCCCTTCATCGCTTATAGTACCCCAAACATCCGACTGCAT

TTTCCAACTGAAATGGAAAATGACTACCGAAATTGCATTGTACATCCAGAATAGACCTAA

GAAAACATGATCCCAGGCGGATACTTGACATGTTCCCCCTCTCCCAGGCCCATCGCAAGG

GAAGCGAAAACCAAGATTTGCTTTATCGGGTATCAAACGGGAACTGCGAGCAAATAAAAC

ACCTTTCAAAAGTATTAATACAGTCACATGGATGGTAAATGCGTGAATGTGATGGACTAA

AAAATCTGCGGTTCCTAATGGAATAGGTAACAAAGCTACTTTGCCGCCTACTGCTACTAA

CTCGCCACCTCCCCACGTTAAGCTGGTACTTGTTGTTGCACCAGGAGCTGTTACGCCAGG

CGCGTTAGCATGGATATTTTGTACCCATTGAGCAAAGATGGGTTGTAATTGTATGGCGGT

ATCCGAAAACATATCTTGGGGACGGCCTAAAGCACTCATGGTATCATTATGAATGTACAA

GCCAAAACTGTGAAAACCTAGAAATATACATACCCAGTTAAGGTGGGATATGATTGCATC

GCGGTGTCTAAGGACGCGATCTAATAGATCGTTGTATCGAGTAGTTGGATCATAGTCTCT

TACCATAAAAATGGCTGCATGTGCAGCAGCACCGACTATTAGAAATCCGCCAATCCACAT

GTGGTGTGTGAACAAGGAAAGTTGTGTACCATAGTCAGTAGCTAGGTATGGATAGGGGGG

CATAGAGTACATATGATGAGCTACAACAATGGTTGTAGAGCCTAGCATAGCTAGGTTAAG

AGATAATTGAGCGTGCCATGACGTTGTTAGGATTTCATAGAGACCCTTATGGCCTTGTCC

TGTAAATGGGCCCTTATGAGCTTCCAAAATATCTTTAAGTCCATGACCAATACCCCAGTT

GGTCCTATACATATGACCTGCGATCAGGAAAAGAATAGCAATAGCTAAATGATGGTGCGC

AATATCGCTCAACCATAGACCTCCGGTTATTGGGTCTAGTCCTCCGCGAAAACTCAGAAA

TTCTGCGTATTTGGACCAATTCAAGGTGAAAAAGGGGGTTGCTCCTTCGGCAAAACTAGG

ATAAAGTTGAGCCAAAAGGTCGCGATTCAAGATAAATTCATGAGGAAGTGGTATCTCTTT

AGGATCAACCCCAGCGTCAAGAAATTGGTTAATTGGTAAAGATACATGGATTTGGTGTCC

CGCCCAAGAAAGAGACCCAAGTCCTAATAACCCCGCTAAGTGGTGATTCAACATGGATTC

TACATCTTGGAACCAGGCCAATTTGGGAGCGGCCTTGTGATAATGGAACCAACCAGCAAA

AAGCATTAACGATGCAAAAATCAATGCACCGATTGCGGTACAATAGAGTTGTAATTCATT

AGTTATTCCAGATGCTCGCCAAAGCTGAAAAAACCCAGAGGTTATTTGGATTCCTCGGAA

ACCCCCGCCTACATCACCATTCAATATTTCTTGCCCTACTATTGGCCAAACTACCTGAGC

ACTGGGTCCAATGTGAGTAGGATCACTTAGCCATGCTTCATAATTGGAAAAACGGGCACC

ATGGAAGTACATGCCACTCAACCAAAGAAAGATAATGGAGAGTTGACCGAAATGAGCACT

AAAGACTTTTCGAGAGATCTCCTCCAAATCACCGGTATGACTATCGAAATCGTGAGCATC

AGCATGTAGGTTCCAGATCCAAGTGGTAGTATCAGGGCCCTTAGCTATTGTTCTTGAGAA

ATGGCCGGGTTTGGCCCATTCCTCAAAAGATGTTTTTACAGGATCCCTATCCACAACAAT

TTTTACTTCTGGTTCCGGCGAACGAATAATCATTAAGTCCTCCTCTTTCCGGACAAGACA

TACAAAGAGACCCGCCAACTTTTTAGTGAACCTTTGAAAGATAGATATTATGATTAGTCC

TTTTCTTTACTATCTACCGTCCTTCTATTTTTTTTAGTTATTCACTGGAGCAATTATATA

TTGAAGTCAATCCGAGGCAAGTGTTCGGATCTATTATGACATAAGGATTAGGTGCCTAAC

GGACATTGTTTATTTTGGATTTCCCGACGTACTAAAAAAACCTTTTTTAATTTACGAAGC

TAGTGTATTTTTTTAGGGTATAAGCTCCTATCTACATCTACTTTCCTTGAGTATAGATTT

TTTTATTCGATTCCAAATTCCAAGATAACTCATTAGAATTATTAATAAGACGGTCCTGAT

ATATTAGCAATATTTAGATCGCCCCCTTTATTCGCTTTATTACTTCTATTCTAGACCCTA

TCGTTTATCCTTATGAAATATAATAAAAAATAGAAGGTAGAAGAAAGGGATATAATGAAA

TTCTTGATTCGATTTACAACCAAACCCCCATTTTATGAAAAAGGAGAGTGGTCTTATTCA

AATTCAAAGCGCTTCGTAATCTTCAACCAGTTCTGTGCTTCAATATAATTTCCCGGAGTA

AGCGCTATAGCTTGTTTCCAATACTCAGCAGCTTGATCAAACCAAGCTTCCGCAATTTCC

GAATCACCCTGTAGAATGGCCTGTTCTCCTCGGTCGGAATAGGCAGTTCCTTCCCCTAGA

ACCGTACTTGAGAGTTTCCTACCTCATACGGCTCAGAAATTGCTATCCTAATTTCCCCTA

TCTTAACTGAATTCGATTTCTCAAAAATCGATCCAATTTCTTCTTGGGTTAAGCAGAAGA

AGTTAATTACCTAAGTTTCAAACCCTAATTTTGATCAATAATCAGTTTGATCTTTTCTCC

CACCTTCAGAAGAATGAAGCATAGATAGACCTATAGCCTTCGTTCGAATTTTCTGAAAGG

TAACTATCTCGGTTTCTTATATGAAATTTCTATAGAATCCTTGAAAAAGACTTTTTCCAT

AAGAAAGAAAAAAGAACTTACTATCTTTGGGATCTGATACTACACCGCTGCTTATTCCTT

AGTGGATCGGCTCTATTACATAAGCAGATTCCTAAATTTTGCCCCATATCATGGGATAAG

TAAGCAGTTTTTTTTAGTTGTATCGACCCAGTCGCTCACTAATGGATCTTTACGGTGCTT

TCTCTATCAATTTGGGCTTTATCCATAGAGTAGTATAGGCCATACTTTCTTCCTATTTTG

ATTCTCGTGAAGTGTCCTTCCTTCCTACAGCTGATAGGGCAAAATCGTTGTTTTGACGAT

CCCTATGTAGAAAGCCCTTTTTCTAGTATTTACTAGAAAATTTGATCCTTTCTTTTTCTT

CTTTCTATAGTGGAGATAGTCGCACGTAATGACAGATCACGGCCATATTATTAAAAGCTT

GCGGTAAGAAGGGGTTTCGTTCTAGTGCCCGGAAATAATATTCCAAAGCCTTTGTATGCT

CTCCATTGCTTGTGTGTATAAGGCCTATATTATAGAGTATATAACTTCGATCATAGGGAT

CAATTTCTAGTCGCGTAGCTTCATAATAATTCTGCAAAGCTTCCGCATAATTTCCTTCGG

ATTGAGCCAACATCCGTTACGGTCGTTCATTCTATTCAAAAAATCTCCGTTCCAAAACCG

TACATGAGGTTTTCATCTCATACGGCTCCTCCCTTCTGTACATAGTACTAAGCGAAAAAT

CTATAGAATAAAAATAGAATTAGTCCCATCTTATTATGAACCGAAAGGGGCTGGTATTTT

TCCAAGAAATCTCTAGCCAACCTTCCCGCAAGAGGTTTTTCTTAACACCAATGAATTCTA

TTAATGCTAGAGGAAAACGATAGCTCCAAGAATTTCTTTGTTCTCAACGCCTCCTATTTA

GAGGAATTAGCCACTTCAACGATCTTTGATGGTTATAGGGGTATCCAAAGTACAAACCTG

ATGGTTGTTTGTTATCCCAACCATTCTTCCCAGCCCTGATACCGATCAGGAAAGGGCTAA

TTTCTAACAAAGTTTTTCTCTTGTTGATTCCTATTTCTAGGTGTAGTGCTTTTCTCCCCT

ATGCTGCCTATTGGTACTAGTAGAGTAGGATTGGCCTGTAATACAGAACCTATCCTGTAG

GTGTAACCTTTCGCTCAATACTCAAATCTACAATTGAAGCATCTGAGGCCGCATCAATCG

AGGATACACGACAGAAGGAATTGTTAGTTCACCTCACCTTCCCCAAGCGTGGGTTTCCTT

TACTAATTTTGTTCTCTCTATGCGAACCCCCTCTCTTTCTCGTAAGACTGAGGTGTAGGT

AGGGCTAAAAAAAAAAAGAGTCAAATCGCACCATCTCTATAATAAGTAAATGCCCTTTTT

TCCCCTGAGGTTGTCGGAATTATTCGCAATAAAATATTGGCTACAATTGAAGAGGTCTTA

TCAATGAAATTTCCATTTATACGGGATCTAGGCATAATTCCCAACCCATTCTATATAGAA

TTGTTTTCATTTCTTCACAAAATAACATAAAAACAAAACATTGATTCTTATAAATCGATC

ATATGCTCTAAATGGATAAGAGAGGTATTTCCGCTCAGCTCAAATTGTCTCCTTTTCCTC

TGTTTGGACAAGAAGAGATATGAAAATTGACTAAGATTGGATTTCATTCCACTTTTCTAT

TTCTCACAATAACTTCTCTCATCAGCTATTTCGCGTTTCAAGTCATTAATTGTCTCATAC

CCTTTTTTATGGCGTAGCGTACTTATGCAAACAGAATTCTAGGGTTCCTTTTTATGGAAT

AAGAAGAATTCTTCCATCTCTTTTTCTTTGGTTTGAACCCAAACTAAAACTTTTCGAGGG

AGCGGAATTCCTAGTAAAAAAATCCTGGATCCTTCCACTTAGATGAAAAGGAATTTTTCC

AATAGAACCATGGAACCCCCGAGCCGTTGTGGTTGTACCTGTACTGCAGGAATAGGAAAA

CTCGCTATTCACTTAGTTTATTTTCCATAATAAGATTATGTAGGAGAGATGGCCGAGCGG

TTCAAGGCGTAGCATTGGAACTGCTATGTAGACTTTTGTTTACCGAGGGTTCGAATCCCT

CTCTTTCCGTTTCTCTTAATTGATCAACGTTAACGATCACAATGTATCAAATCAAATAAC

AATTTATTCCAGCAATAATACCTTTATTTAATAGAAATTTTTTATAGTAAATTACTGTGG

TATGTAAAATACACATAGAGGAAAGAACAAAGAAAAAGGATCCTAGGGTTAATCCATTTA

TGCTAGTTGAATGGAAAATACGAATTAAGGGCCTTAGGTCGATTTAGTTCGGGGGAAGGG

GAAGAAAATTCTATGAACTTTTCCTTTTTCGTTAAGTTCAAGTCTGACGAGAGTAATATT

CTACAACTAACAACTCATTTATTTTGAGACCGACCCACTTCCTATCTAGGATTTTTTTAA

CTAGTCCTTTATATTGCAATGTGTCAATCGTCAAATGCTTTGGCAATTTCCCCGGGTCGG

ATGAAGCAATAGAATTTTGAATCAGACGTTTTGATCTTTGGTTATCCTTCGTAGTAATAA

TATCTCGGGGTTTGCAACGAAAACTTGGTATATCGACTATACGACCATTAACTAAAATAT

GTCTATGGTTAACTAATTGCCGGGCCCCAGGAATGGTTGAAGCCATACCCAATCGAAAAA

GGATATTATCCAAACGCATTTCAAGTAATTGTAGTAAAACCTGACCTGTTGACCTTTTTG

CTTTTCCAGCGATATGTACATATCTAAGTAATTGTCGTTCTGTCAGACCATAATGAAAAC

GCAATTTCTGTTTTTCTTGAAGACGAATACGATATTGCTCTTTTTTCCCAGAATGGAATT

TCTTTTTCAGATTACTTCCGGATTTAGGTGTTTTTCTAGTGAGTCCTGGTAAAGCTCCCA

GACGGCGTATTTTTTTTAAACGAGGTCCTCGATAACGGGACATGAAGACTCCTTGTTTTA

TTGAAATTTCATTTTACACAATTAATTTCATTGTATTTACATTACAGAATACATCAAAAT

TAAAACTGAATTAAACTAAAGGATAAACAGAGTAAAATCTACTAAAGTACCACAAAAAAT

GGAATTTCATCAACATCTGGATTTTTGTATATATATTATTTATTTTATTGTTTTGTATCT

AGCAAAATTGTAAGGTAGAACCACATAATAGATCCTGATTCTCCATTTAATTCGGAGAAA

AAGAGAGATTCTTGTTCATGGAACATCGATATAGAAAAAAGCCGACTATCGGATTTGAAC

CGATGACCCTCGCATTACAAATGCGATGCTCTAACCTCTGAGCTAAGTGGGCTTACATAA

CAGAAATAGTGTAACAAATAGAAATATGTATATAGGAAATCCGTAAAATGTCAGATCTTA

ATTATTAATCTTAGCTATTAACTAGTTCGAAATTGGAAGTTCTACTTAGAAAAAAATACT

AGAACTTCATAAAATAAAGTTAAAGAAATTTTTGAACTTTCTTTTTTCTCTAATTTTTCT

AATAGAATCTATTCCAATTTCTATATTGAATTTGATTTCAGATATTTTCAAATGGCTCGG

ATGAGTAATCTAATACATAGAAAAGAATAATATATATGAAAGATATAATAAAGAGAAAAT

GCGAATTTCTTGCATTTTCATTCGATCATTATAGACATTTTTGAGATAATAATTTAATGA

TTAATATTTCACTAAGGAGAACATAGAATCATAGCAAATGAAATTGCTAATTCTGATTAG

AAAAAAAGAATGAATATCAAGCGTTATAGTATGATTTTGAATACTCTAAAAAAGAAAGGC

GGGGGAGAGAAAAACTTTGGGATATATTGATTCGGATTGAATTGCAAATACATCAACGAT

AGAATCAATTCAATTCTGAATTGCAATAAGCAGGGTCTGTCAAATAGAGACGAACTGCTA

GACTACGTCGAGTAATTAATTCAACGATTCAAAAAAAACTAAGAGATGGATGAAATTACA

CAAGGAATCCAGGTCTCAAGGAAAATGGGGATATGGCGAAATCGGTAGACGCTACGGACT

TGATTGTATTGAGCCTTGGTATGGAAACCTGCTAAGTGGTAACTTCCAAATTCAGAGAAA

CCCTGGAATTAAAAAAGGGCAATCTGAGCCAAATCCGTGTTTTGAAAAACAAGTGGTTCT

CGAACTAGAATCCAAAGGAAAAGGATAGGTGCAGAGACTCAATGGAAGCTGTTCTAACGA

ATCGAGTTGATAACGATTAATCACGGAACCCATATCATAATATAGGTTCTTTATTCTTTT

TTAGAATGAAATTAGGAATGATTATGAAATAGAAAATTCTGAATTTTTTTAGAATTATTG

TGAACCCATTCCAATCGAATATTGAGTAATCAAATCCTTCAATTCATAGTTTTCGAGATC

TTTTAAAAAGTGGATTAATCGGACGAGGATAAAGAGAGAGTCCCATTCTACATGTCAATA

CTGACAACAATGAAATTTCTAGTAAAAGGAAAATCCGTCGACTTTATAAGTTGTGAGGGT

TCAAGTCCCTCTATCCCCAAACCCTCTTTTATTCACTAACTATAGTATTTATCCTCTTTT

TTTTATCAATGGGTTTAAGATTCAATTGAATAGATTTCTTTTTTATTATAGTATCGGCAA

GGAATCTCGATTATTAACTCTATTTTTAAGTATTATTAAGTAAGCCATGCACAATGCATA

GGATTACCCCCCCTCATTTCCAAATTTCGAATATTGACATAGATACAAATACTCTACTAG

GATGATGCACAAGAAAAGGTCAGGATAGCTCAGTTGGTAGAGCAGAGGACTGAAAATCCT

CGTGTCACCAGTTCAAATCTGGTTCCTGGCACAGAAAAAAAGGATCTACCGAATAGGTAT

TGATACAAATACCTCGAGATGGGTTGGGATACATATTCGTTAATAATATAGATAGAGTAT

GATTTAATCTAAGTAGATAAATCTCTAAATAGAGGCACTTCTTTTTCTTCATTTTGCATT

TCTTAATTTTGTATTCCGCTATTCCGACGAATATTTATTTATTCTTGTTATCTTACTTTC

CTAGCTGTTCTAAGTAATGCGCGCGGTACAAAGTTCGTGGTAGGAACTTCTTTGAGTCAT

CATATTTTTCTGTTCATACGAAGGAAATGAATATGTGATTTTCCAACGAAATGAAGCCCT

TTTTGCTCAGTCTATCTGGACCTTTTGTATAATAGGAATTAATTAGAATATAATAGGTAT

TCCGTTTCATCTAGGAACAGAACGTAAAAATATTCCTTGACTTGAATAAAATCTGGAGTT

GTGTTGTATAAGTGAGCATGAATTTCTTATCATTCAATGAGCATCTTGTATTTCATAGAA

ATTGGGGGTTATATAGTCCTTACGTAAGGGCCAGCCTATCCAACTTTCAGGCATTAGAAT

ACGTTTAAGGCGTGGATGATTATCATAAGAGATTCCCACCATATCATAAGATTCGCGTTC

TTGAAAATCGGCACTTCTCCAAATCCAGAAGACAGACGGGATTCTAGGATTATCCTTTTG

GGCAAAGACTTTTATGCATACTTCTTCTGGGTTATCTATACCATACTGTATTCTCGTAAG

ATGATACACGCTAGCTAAAGATCCACCGGGTGCTACGTCATAAGCACATTGGGAACGTAA

ATAATTGTAACCATATACATATAAAATGACAGCAATGGAATCCCAATCCCCTGCTTTTAT

TTGTAAAGTCTCTATTCCTCGGTGATCGAAGCCCAAAGATCTATGAACCACCTCATGTTT

GACTAGCCAATTAGATAACCAACCCTGCTGCATTGTCTTGATCTCTCCCCCTTTGTATAA

ATATTTCACATTTCGAATGCAAGTTTGAAAGATTGCCCTGCTCTTTCTTTTTCTACACAA

AGAACCCCTCCTAATTCACTAATTTGGAGGAAGATACTGGACTTTTGGATTTGAAAAAAG

TTTCAGAAGATATGTCTAAAGTAGATGGTGATTGATAGAGCAATTCTTGCTCGTAAGTTC

CAGTATAAGTACTGCGCCTAACATAAAGCTTGTGACTGGTAGTAAAACATCGATTTTTCT

TTTGAGATAGAGTTCGATCCTCAACTATTTCTCGCGATATCTTCTTACGAAGTTTTGTTA

GGGCATCTATAACTGCCTCCGGTTTAGGTGGGCAGCCCGGCAAGTAGACATCCACAGGAA

TTAACTTATCAACTCCCCGAACAGTACTATAGGAATCCGTACTGAACATTCCCCCTGTAA

TAGTACAGGCTCCCATAGCAATGACGTATTTTGGTTCAGGCATTTGCTCGTATAATCTCA

CTAAAGAGGGAGCCATTTTCATTGTTACCGTACCAGCTGTTAAAATTAGGTCTGCTTGCC

TAGGACTTGATCTTGGTACCAATCCATAACGATCAAAGTCGAATCGTGAGCCTATTAATG

AAGCAAATTCAATGAAACAACAACTGGTACCATATAGAAGGGGCCATAAACTGGAGAGTC

TTGACCAATTCGAAAGATCATTTGGTGTAGTTGAAATAACGGAATTGGAACTTGTTTGGT

CAAGTAAGGGAAACTCAATCAAACTCATAACTGTCTCAATGGAATCTTTTCCTTCTTTTT

TTTTTTTGTCTGAATATTCAGTTAAGACCATTCCAAGGCTCCTTTTCGCCATGCATAAAC

TGAACCAACAACTGGGATAAGCACGAAAATGAAAGCTTCGATAAAAACGGATACACCCAA

TACGTCGAAACTCATTGCCCAAGGGTAGAGAAAGACCGTTTCCACATCAAAAACAACAAA

AACTAGCGCAAACATGTAATAGCGTATTCGGAATTGTAACCAAGCCCCTCCCATGGGTTC

TATACCCGATTCATAACTAGAAAGCTTCTCTGGTCCTTCACTAATCGGGGCTAAAAGTCC

TGAAATCCAAAATGCAAAAATAGGAATAAGGCTTGCTATTATTAGAAATGTCCAAAAAAT

ATCATATTCGTGAAGCAGAAACATAAATGTACTCCCATTAATGTGGAATAGGCGGAACTG

AATTAGTCAATTCAAGTCAGCGTTGTCAATTTATCCAGAACTTCTCTCTTTTCCTCGGTG

AAACAAGAATCCGTTTTGCTCAAATCAAAGCACTTAGTTTAGCCTTTGTTTCCTCTGTGC

CCTGTCTTCTTTAAAGATTCATCCAATGGAATCCCGACTCCCTTTTTGATTTCCATTCTA

TTTATAATTAGAACCTAATTAAGATAGGATGACTAATGTATGCAGCCTAATGAGGAGTAA

TACAAAAATAAAGAACTCTATTTCAGAAAGTAGATCGATTTAGATAATGTATATAAAAGT

AATATACTTCAAACAAAGTAGGAATTCGCAAGATGGAGAAAATCTTGCAGTTGATTTGAT

AGAAATTCGTTTTTCTTTTCCTGTCTCTAAGATTTTCGATGAATGAGCCTGTGGTAATGC

TTTTATCTCTATTCTATGGCGCAAGCGACCGTCCAGTCTATAAACAAGTACTAATGAGGA

AATGAAAACTATACTAAAGGAAACATAGGATCTCTATCCTAAAATCTAAAAAAGGACATA

TTAGGGCTATACGGATTCGAACCGTAGACCTTCTCGGTAAAACAGATCAAACGGATATTA

TCGAAATGATTCGAACTGTTTCAAAGACCCAACATGCATTTTTTGCATTGGGCTCTTTCA

TCAACTGATGTAAAGATCAGTTAGTCCACCATAGTTTTTCTTTACGGAAAGATAATGAGA

TGGCTCCCTGTGCTCTGATTGATTATTTGTATTATGATCTATCTAGGAGCAATACCAAAG

TGTTTCAAAGGAGGATTACCTTGACTTAGGTCGCCTCCGGCCTAAATTAAATCAACCTAA

GTGAAATGGAGTCTCTATCGTTCCGCTGCAAGAGTTGACTATGAGACTTCATACACCTTA

AAGTTCATAGAACGAAAAGAAGTTTTTTGGAGGCCCTTATCCTCATTACGCCTAGCATTT

AGTGGGCTGGATATTTACCTTATCAACTAGCAAATCAATAAGGGTTCTATTTGATTAGGC

ACCTGAATTGGCACCTGAATCGGACTGAACCGACTGTTTGTCAGGCTACTGTTCTCCTAT

TCTCTCGAATCCATGAAGTAAGACATTGATTTTGCAATAAGATCAATTATGTTCATTGCA

TAATAAGCTCCCTTGAAAAGCATTGGCGCACGTGTAAGCGAGTTGCTCTACCGAACTGAG

CTATAGCCCTTGTCAGAGATATCTTAACATATAGATAATTTCTTGTCAAGATGAATATTC

TCTAATGCCAGAGGATATCCTTTGATCTGTTTACTATATAACATACCAATAACGGAGCAG

TATTGCTTATAAAAAGGATTCGATCTATAATCGATCGAAGTAATGGGTCTTCCTTTGTGG

TGATAAATTGCCTACTTAACTCAGTGGTTAGAGTATTGCTTTCATACGGCGGGAGTCATT

GGTTCAAATCCAATAGTAGGTAGGTAGGTAGAAAAATTACTAGATAGCATTGGACTTACT

TCGCTTCGCTATCTAATAACTTTTTCTACCCCTCTTCCCTTTTTCTTTGTATCAACTAAA

CCATTGGATTGTATTCAATTGGATGGGGGAATCCAATTGATAGCCTCGACTCGTATCCTA

GCTCGTCTGAGAGCTAGCTTCGCTTCAACCAACTCTTTCGTACCCTCAGCTCTACTCAAG

TTAGCTTCGGCTATTTCAAGTGCCTGTTGAGCTTCTTCCGGATCAATGTCACTACCCAGT

TCCGCATCATTTCCTAAAATGATGATCTCATTATTAACTATTCTCGCAAAACCGCTCCAC

AGAACCGCCGTTAACCATTGGTCGTTGAGGAGGCGTATTCTCAAAGGACCCATATCTACA

GCTGTGTTAATGGGGGCGTGGTTTGGTAATACGCCAATTTGGCCACTATTAGTAGATAAA

ATGATTTCTTTCACTTCACAATCCCAAATAATTCGCTTAGGAGTTAGTACATAAAGATTT

AATTTCATTTCTTCAATTTGTTCTCCTCTTCTAAGTTTATAGCTTTCGTGCTAGCTTCAT

CGATGTTACCCACCAAATAAAAAGCTTGTTCGGGTAGGCCGTCTAATTCTCCGGAAAGGA

TTAGTTGAAATCCCCTAATTGTTTCTGCAAGACCAACATACTTTCCTGCAGAACCGGTAA

AAACTTCTGCCACAAAGAACGGTTGTGATAAGAAACGTTCAATTTTTCGTGCTCTTGCTA

CAGTTAAACGATCCTCCTCTGATAATTCATCCAACCCAAGAATTGCGATAATGTCCTGAA

GTTCTTTGTAACGTTGTAAAGTTTCCTTAACTCTTTGCGCAGTTTCATAATGTTCGTTGC

CAACGATCCGAGGCTGTAACATAGTTGAGGTTGAATCTAAAGGATCTACTGCTGGATAAA

TACCCTTGGAAGCTAATCCTCTGGAAAGTACGGTAGTAGCATCCAAATGTGCAAATGTTG

TGGCAGGAGCAGGGTCGGTCAAATCGTCCGCAGGTACATAAACTGCTTGGATCGAAGTTA

TGGATCCCTTTTTTGTAGAAGCAATTCTTTCTTGCAAAGAACCCATTTCTGTACTAAGAG

TAGGTTGATAACCCACTGCGGAGGGCATTCTCCCTAATAAGGCGGATACCTCCGATCCTG

CTTGAACAAAACGAAAGATATTATCGATGAATAGAAGCACGTCTTGCTTATTAACATCTC

GGAAATATTCTGCCATAGTTAGGGCAGTTAAACCAACTCTCATACGAGCTCCCGGCGGTT

CATTCATTTGACCATAGACTAGAGCTACCTTTGATTCCTCCAAATTTTTTTCATTAATTA

CTCCGGATTCCTTCATTTCCATATAAAGATCATTTCCTTCACGAGTCCGTTCCCCTACTC

CGCCAAATACGGATACGCCTCCATGAGCTTTAGCAATGTTGTTGATTAATTCCATGATGA

GTACTGTTTTACCTACTCCAGCCCCCCCAAATAGTCCTATTTTTCCTCCACGTCGATAAG

GAGCTAAAAGATCGACCACCTTAATACCTGTTTCAAAGATGGATAATTTCGTATCTAGCT

CGATAAAGGCAGGCGCAGATCTATGAATAGGGAATGTTGCATTAATATCTACAGGACCCA

AATTGTCAATAGGTTCCCCAAGAACGTTGAAAATTCGTCCGAGAGTAGCTCCACCGACTG

GAACACTGAGAGGAGCTCCCGTGTCAATCACTTCCAATCCTCTCATCAACCCGTCTGTAG

CACTCATAGCTACAGCTCTAACTCGATTATTTCCTAATAATTGTTGTACCTCACAAGTCA

CATTAATTTGCTTACCGGCAGTGTCTCTACTCTTGACTACCAAAGCGTTATAAATATAAG

GTAACTTGCCCGGGGGAAAAGTGATATCCAGCACGGGTCCAATAATTTGATCGATACGCC

CTATGCTTTTTTCTTCAATTGTGGAAACCCCGGGACGAGAAGTAGTAGGATTGGTTCTCA

TAATTATCACATAATTTTCAAAAAAAAGGAATTTGTCGAATTTTTTCTTGTTGAATAATG

CCAAATCAAAAAAAATAGCCAAAAATCCAAAAGTCAAAAGGAAATGAATTAGTTAATTCA

ATAAGAGAGAAAGGGGGACGAGGACTTGATTTCGTTGCCCAAGCGAATCCCATTCAATCG

TTTACTCATGGAATGAGTCCGTTGGAAAGTTCAATCAATTTTTTTCATATACATTTCGCC

TTTTGTGGAGGATCTGTCCCTACTCTACTTTCCTATCTAGGACTTCGATATACAAAATAT

ATACTACTGTGAAGCATAGATTGCTGTCAACAGAGAATTTTCTTAGTATTTAGGTATTTA

CATTCAAAATAAGAAAGGGGCCTATTAAGAACTTAATAAGGATTAGGGATTGGGTTGCGC

TATATCTATCAAAGAGTATACAATAATGATGGATTTGGTGAATCAAATCATGGTTTAATA

ACGAACCATGTTAACTTACCATAACAACAACTCAATTCCTATCGAATTCCTATAGTAGAA

TTCCTATAGGATAGAACATACACAGGGTGTACGCATATATGAATGAAACATATTCATTAA

CTTAAGCATGCCCTCCATTTTCTTTAATGAGTTGATATTAATTGAATACCCTTTTTTTAG

ATTTTTGCAAAGGTTTCTTTCGCCTAATCCTATCGAGTAGACCCTGTCGTTGTGAGAATT

CTTAATTCATGAGTTGTAGGGAGGGACTTATGTCACCACAAACAGAAACTAAAGCAAGTG

TTGGATTTAAAGCTGGTGTTAAGGATTATAAATTGACTTATTACACCCCGGAGTATGAAA

CCAAGGATACTGATATCTTGGCAGCATTCCGAGTAACTCCTCAGCCCGGGGTTCCGCCCG

AAGAAGCAGGGGCTGCAGTAGCTGCCGAATCTTCTACTGGTACATGGACAACTGTTTGGA

CTGATGGACTTACCAGTCTTGATCGTTACAAAGGGCGATGCTATCACATCGAGCCCGTTG

TTGGGGAGGAAGATCAATTTATCGCTTATGTAGCTTATCCATTAGACCTATTTGAAGAGG

GTTCTGTTACTAACATGTTTACTTCCATTGTGGGTAACGTATTTGGTTTCAAAGCCCTAC

GCGCTCTACGTCTGGAGGATCTGCGAATTCCCCCTACTTATTCAAAAACTTTCCAAGGTC

CGCCTCATGGTATCCAAGTTGAAAGGGATAAGTTGAACAAGTACGGCCGTCCTTTATTGG

GATGTACTATTAAACCAAAATTGGGATTATCCGCAAAAAATTACGGTAGAGCGTGTTATG

AGTGTCTACGCGGTGGACTTGATTTTACCAAAGATGATGAAAACGTAAACTCACAACCAT

TTATGCGCTGGAGGGACCGTTTTGTCTTTTGTGCCGAAGCAATTTATAAATCACAGGCCG

AAACCGGTGAAATCAAGGGGCATTACTTGAATGCGACTGCAGGTACATGCGAAGAAATGA

TGAAGAGAGCTATATTTGCGAGGGAATTAGGGGTTCCTATTGTAATGCATGACTACTTAA

CTGGGGGATTCACCGCAAATACTACTTTGGCTCATTATTGCCGCGACAATGGCCTACTTC

TTCACATTCACCGGGCAATGCATGCAGTTATTGATAGACAGAAAAATCATGGTATGCATT

TTCGTGTATTAGCTAAAGCATTGCGTATGTCTGGGGGAGATCATGTCCACGCCGGTACAG

TAGTAGGTAAGTTAGAAGGGGAACGCGAAATGACTTTAGGTTTTGTTGATTTATTGCGCG

ATGATTTTATTGAAAAAGATCGTGCTCGCGGTATCTTTTTCACTCAGGACTGGGTATCTA

TGCCAGGTGTTATACCGGTGGCTTCAGGGGGTATTCATGTTTGGCATATGCCAGCTCTGA

CCGAAATCTTTGGAGATGATTCCGTATTACAATTTGGTGGAGGAACTTTAGGACATCCTT

GGGGAAATGCACCTGGTGCAGTAGCTAATCGGGTGGCTTTAGAAGCTTGTGTACAAGCTC

GTAACGAAGGGCGCGATCTTGCTCGTGAAGGTAATGAAATTATCCGAGCAGCTTGCAAAT

GGAGTCCTGAACTAGCCGCAGCTTGTGAAATATGGAAGGCGATCAAATTTGAGTTCGAGC

CGGTAGATACTATAGAAAATAAAAAAGAAGCGAAATAGAAAGAGAAAAAAGCAGTTACGA

AATGCAGTAATTCTTCTTTATTCTTCTAATTGATTGCAATTAAACTCGGCTCAATCTTAA

GATTGAGCCGAATAAAAATAGATCTTGATACGATCATGAGACTTGACAAATCGAGATTCC

TCTATTCTATATATTTAGAATATATAAAGGTATAATACAATAAATAAATACAAATATAGT

ATTTATTTACTTTATTGGGAAAGAATCAATGAAAAAAGATTAGGAATCGATAATATTCTT

CTATAATCCCGGGGTCTTACTAACAGGACTTCGCTGCACGGGAAGGTAAGAGAATTTGCC

CTATTTTGGAAATTACAGCTTAATAAAAAAGTCTAAGCACTATCAATCAACTGTCGTACT

TTGAATCCAATTTCAAGTTCGATTAGAAGGATAGAAAGGCCATGAGGACGGGAAAAGAAA

AATCAAATCTTTTTAATCTCCTTTTTTGCAATTTTCTTATTATCCATTCCATTCATTTTT

TTTATAGAATACTATCTATAAAAAATCTTTTTTTGCAAACTAAAAAATACAATAGTCAAT

ATTCCTTATAATAGATATACTTAATTATATTATAAGAATCCTAAGATATTTTTCGAATAG

ATAGAAATAGTAAATTTGAATTGAGACACCTATTCTATGACGGATTTTAACTTACCTTCT

ATTTTCGTGCCTTTAGTAGGCTTAGTATTTCCGGCAATTGCAATGGCTTCTTTATTTCTT

TATGTGCAGAAAAATAAGATTGTCTAGAACCGATGGGGCCGAATTTTCTCAATGTATTTC

CAGGATCATAATACAGATATTTTTTAGTGTAAGTAATATGGTAGGGTATGTGGCTCTTTC

TACACACAAATGAAAAACGGCTATGGATGCGGATATAGGCTACGAGCATAAATGCATGCA

TATGCGGAGCCGGGTATAGCGAGTTTTTTTAAGTAGATCAACAGATATTTTTTGAATAGA

AAGTCAATGTATCTAACCAATTATTTCACAGGAGTACTAGTTACTGAAGGCGATTTCAGA

ATCAAAAAAAGTAAAGTCAAAATCATTTAGCTTATTCTCTCAATTTCAATCGACCGCTGT

TAGTATATCTAATATGAATTGGCGATCAGAACACATATGGATAGAACTTCTAAAAGGTTC

TCGAAAAAGAGGTAATTTTTTCTGGGCCTGTATTCTTTTTCTAGGTTCACTAGGATTTTT

ATCGGTTGGGGCTTCCAGTTATCTTGGTAAGAATATGATATCTGTACTTCCATCTCAACA

AATTCTTTTTTTTCCACAGGGGGTCGTGATGTCTTTCTACGGAATCGCGGGCCTATTCAT

TAGCTCCTACTTGTGGTGCACTATTTTGTGGAATGTAGGCAGTGGTTATGACCGATTCGA

TAGAAAAGAGGGAATAGTGTGCATTTTTCGTTGGGGATTCCCTGGAATAAAACGTCGCGT

CTTCCTTCGATTCCTTATGCGGGATATCCAATCAATTAGAATTCAGGTTAAAGAGGGTCT

TTATCCTCGTCGTATCCTTTATATGGAAATCCGGGGCCAGGGGGTCATTCCCTTGACTCG

TACTGATGAGAAGTTTTTTACTCCACGAGAAATTGAACAAAAAGCTGCCGAATTGGCCTA

TTTCTTGCGCGTACCAATTGAAGTATTTTGAATACCGATTTAATTTTTTGAAATTGTAAG

GAGATTTTGAGTATTTATCTAAAGAAAGGAACAAACGAGGATAAGAGAAAATTGCTTCTA

ATTTGTCCAAGTGATGGCATAATATTCTTCCATTTTCATCCGAAAGGGCTTTTTCTATTT

CTCTATTCCACTCCATCTAGATCTAAGAAAGAACCCAATGCAATGAAATTCCACTAGTAT

ACAAAAAAGAGGAATAGATACAAGGTCTCAAACCTTGTTATAGAATTTTTGCTTCAAATA

AAAGAAATATCATATATATTCATTTACAGATCAAAATGAAAAAAAAGAAAGCATTGCCTT

CTTTCCTATATCTTGTATTTATCGTACTTTTGCCCTGGGGAGTCTCTTTCTCTTTTAACA

AATGTCTGGAACTTTGGATTAAGAATTGGTGGAATACCAGGCAATCTGAAACTCTCTTAA

CTGATATTCAAGAGAAAAAGGTTCTAGAAAGATTCATAGAATTAGAAGAACTTTTTCTCT

TGGACGAAATGATAAAAGAGAAACCGAAGACACATGTACAAAAACCTCCTATAGGAATAC

ACAAGGAAATAATACAATTGGTCAAAATAGATAATGAGGATCATCTCCATATCATTTTGC

ATTTCTCGACAAATATAATCTGTTTGGCTATTCTAAGTGGTTCTTTTTTTCTGGGTAAAG

AGGAACTTGTCATTTTGAATTCTTGGGTTCAGGAATTCTTCTATAACTTAAATGACTCAA

TAAAAGCTTTTTTGATTCTTTTAGTTACTGATTTTTTTGTTGGATTTCACTCCACCCGCG

GTTGGGAACTACTAATTCGTTGGGTCTACAACGATCTTGGATGGGCTCCTAATGAGCTAA

TTTTCACTATTTTTGTTTGTAGTTTTCCAGTGATTCTAGATACATGTTTTAAATTTTGGA

TCTTTTTTTCTTTAAACCGTCTATCTCCTTCGCTTGTAGTCATTTATCATTCAATTAGTG

AAGCATAAACTCATTCGATTTCCTGATATTAATCAAATTAGCATCTTTCTTCCTTTAGAA

AGAAAGCCCTTTTCCATTTTAGCAAAATTCTTTCTATTTCTACCTGCTCAAGGTATTCAT

CATTCCAGTACAACTGTTGCAGTAGAATGACAACAGACTCGTGTATAGGGAACTAGATTA

GCTTAGCTACCTATCTAATTTATTGTAGAAATTCTGGGATCTGCGATTGGATATGGAAAA

TAGAAATACTTTTTCTTGGGTAAAGGAACAGATGATTCGATCGATTTCTGTATCGATCAT

GATATACGTAATAACTCGGACATCTATTTCAAATGCATATCCCATTTTTGCGCAGCAGGG

TTATGAAAACCCACGAGAAGCAACCGGACGAATTGTATGTGCCAATTGCCATTTAGCTAA

TAAGCCCGTGGATATTGAAGTTCCCCAAACTGTGCTTCCCGATACTGTATTTGAAGCAGT

TCTTCGAATTCCTTATGATATGCAACTGAAACAAGTTCTTGGTAATGGGAAAAAGGGAGG

GTTAAATGTGGGTGCTGTTCTTATTTTGCCCGAGGGATTCGAATTAGCGCCGCCCGACCG

TATTTCTCCTGAGTTGAAAGAAAAGATAGGAAATCTTTCTTTTCAGAGTTATCGTCCCGA

TAAAAAAAATATTCTTGTGATAGGCCCTGTTCCCGGTAAGAAATATAGTGAAATCGTCTT

TCCCATTCTTTCCCCCGATCCTGCTACGAAGAAAGACGTTCATTTCTTAAAATATCCCAT

ATATGTAGGGGGAAACCGAGGAAGGGGACAGATCTATCCTGATGGTAGCAAGAGTAACAA

TACGGTCTATAATGCTACGTCAACAGGTATAGTAAGAAAAATACTACGTAAAGAAAAGGG

GGGATATGAAATATCCATAGTCGATGCATCGGATGGACGCCAAGTGATTGATATTATACC

TTCCGGGCCAGAACTTCTTGTTTCAGAAGGGGAATCGATCAAGCTTGATCAACCATTAAC

AAGCAATCCTAATGTGGGAGGGTTTGGTCAGGGGGATGCAGAAATCGTGCTTCAGGATCC

ATTACGCGTCCAAGGCCTTTTGTTCTTCTTCGCATCTGTTATTTTGGCACAAGTTTTTTT

GGTTCTCAAAAAGAAACAGTTTGAAAAGGTTCAATTGTACGAAATGAATTTCTAGGTCCC

GGCTTACCATCAAGTTGGTAAAAAGCCGCGATGCCTGGAATTCCTTATTTCTATCTCATG

CAAAAGAAGAGAATTCAAGGCAAAGGCGAGAACAATAAAAGGATTTCCTCCTTTTAGGAG

GGATTGCTGGTCTTCTTAATCCTCTATTGGGCACAAGAAAAAGGCTTTTTTGCCTTTTTC

TTGTGTCGATTCTTCTTGTATCAAATCAGAATCATTTTTCTTCCTATTCGTCAAAGATTA

CTATTTCTTCTTTATTCGGGTCTGTCTCTTGGACCTCTTTTGCTTAGGTTCAGGCGAGGT

AGTGGACAAACGGAAAGAAAATATGGCGGGGACAAATTTCTTGTGAGCAAATAGAATTGC

TTGACTTGTTCAATTAAGTTCAACTTCGAACTTACAGAATTTTTGCAAAAAAACGTATAC

TCTTCTATTGAAGGGTGGTTTTTCTTTTTAGGCTAGTTGAGTAGTTTTTATTAAGGTTTT

ATTAGTTTATTACTCTAAACTAAATCAATGATTTGCAGGATACTTCTTCCGTGGAATAAA

ATATTGGATCCTCCCCCTTCTTTCTTGTTGCTTCATAAGAGTGAATCAATTTCATGGGCG

AAGGGATAAATCAACCGATGGATTGCTTCACTAACACCATTAACAAACAAAAGAATAAAT

AGAGGGATTCTGACCATCAGAGCAAAGGTTTCTCTTTGTTATTTTTACAAATCGAAATAG

GTAACCCATTTGTAGGTTATGGAATAAAGTTATATTATAAGAGTAAGAATTCCGCGGGTC

CTTTCCGCTCTAATCAGATAAAAGGGGGTAAGGACCCGCTAAGCTCCTACTTTTTCATGT

TTACAATCTGGTCCCTCCGATTACTATAGAGATGAACCCAATCCAGAATATGAACCGTAA

AAGAAAACACCTATTAAACCAATCACAGGAATACCAGTTACAGTACCTATCAGCCAAAGA

GGAATTCTTCCAGTAGTATCGGCCATTTCCCCTACTTTCCTCCACATTTTATCAAGTGGT

CATGCTAGAGACAAAAACAGTCATGGATAGTTATAAGGATGGTATCCTTCCAAATGGGAT

AAGAGAATTCTTACTACTCTCTTTCTTTCTCTCAATTGAAGAAGTAATTGGAAAATAAAA

CAGCAAGTACAAAAATGAGTAATAAACCCCAGTATAGACTGGTACGATTCAATTCAACAT

TTTGTTCATTCGGGTTTGATTGTGTCATAGTTCTATAGTTGGAATTTGGTTTATCGTTGG

ATGAACTGCATTGCTGATATTGATCCCAAGAAAAAAACAGTAGGTACGGCTAGTCCGTGA

ACAGCCAGCCATCGGACTGTAAAAATAGGATAGGTTCGATCTATGGTCATTGAGGGCCTC

CTAAAAGGATCTACTAAATTCATCGAGTTGTTCTAAAGAATCAAAACGGTCGGTTATTAA

GGGAATTCCTTGTCGGCTTTCCGTGAAATACTCGTTTGGCCGAGGACTTCCAAACACGTC

ATAAGCTAAACCCGTACTGACAAATAACCAACCCGCAATGAATAGGGAAGGTATAGTAAT

GCTATGAATAACCCAGTATCGAATACTGGTAATAATATCAGCAAAAGAACGTTCTCCCGT

GCTTCCAGACATGCTGAGCTCCCCAAATTTTTATTCAAAAAAGGAATTGATTCCGTAAAA

GATGGGATCAACCAGTAAATAGAAAATTACTGATATTTCATCCTTGTGAGATTGTCAATT

TTGTACCAAAGGTGTATTTTGAGTATACCGAATTAGTATAGCTATCCTTCCTATGGCACA

GCAATCTAGTTTTGCTTGGTCCCGAAACATAATTCTTTTTTTCTCTTCTTTGTTCCTTGT

CTATAGGTAAGCTATATGTTATTCAAGGCATCAATAGAAAACCCCATTTTTGGGGTCCTG

CTTATTTTCATTGGCTTCGGATTAGTAGAATAATTCGGAATAGCGGCCAAGATCTTGGGA

AAATCCAAGTTAATGATCAATAGGATAAATAATTTAGGAAAGATATTCTCATACTGACAC

AATATAAGGACAAGTATATGCGAAATCTATCCCTTAAGGAATTTAATTGGTTAGCATAAT

ATCTAATAAATAGAAAATCGAATAGTGGATAATCTGTTATGAGAGAAAGAAAACATTCTT

TGAAGAATCAAGATTCGTAATCAATCCTTGCCTTGTTTACTAACTTTCTTGACTAAACTG

CAAGCGTGGAACTCAGGAAACTTCGAATCAACTTTGGTTGGGGTTCAAAAAGGAATAAAA

ATAAAGTAAATTCAAGGAAGATTTCCTTTTTTTAAGGGGCCCTCGGGGGTCGTGGAATGC

TTTTCTTCTCCTCTTATTCCATATGGAATACAATCAGTTAAAATAAGAAGGAATAGGGAA

TATTCGACTGTTTCAATTCTTTATTTATCTTATATTCCAAAATTCTCCCAAAATCCAATT

TAATTTTTCAATGGGGTTAGATGATCTAGTTCTTAATATTATTACTTTAAAGAACTGACA

GATTCCACAACAAATCTCTTGATTCGGAATTAGAGACTCATGTCCCATCTGATGAATCGA

TTTTCTTTTACACTTCTGTATCTCACTCTATCTTGTTTTTTAGTATTATCTAAAATAACC

GATGAATTATGAATTTTCCATAACTTAGGTAAGTGCTTTACCAACATATGTAGTGTAGTA

AAAAAAATGGAATTTAACCCTTTCATGCTTACTATAACTAGTTATTTCGGTTTTCTACTG

GCTGCTTTAACTATAACCCCAGCTCTATTTATTGGCTTGAACAAGATACGTCTTATTTGA

AATGAATTGAAAATCTTTCTTTTGGATTCCTGGTATTCTAGCACTAATTATCAATTCTTT

TCTTGGTCATTGAGATTCGTGGATAATTTAGACTACTATTTAGGGATAGATCGTACCTCT

TTTTTTATCCCCTCGAACAAATCGAAATGATTGAAGTTTTTCTATTTGGAATCGTCTTAG

GCCTAATTCCTATTACTTTAGCGGGATTATTCGTGACTGCGTATTTGCAATACAGACGTG

GGGATCAGTTGGATCTTTGATTGAGTAATATTTCTTTTTTGATTGACCTCCTCCAGACCA

GAGAGGAGGTCAAATTGGAGTTGCAATTTGTTTTGTTAAGTTATTTTAGCTTCGACATAA

GATAGATGGAATCACGCTCTGTAGGATTTGAACCTACGACATCGGGTTTTGGAGACCCGC

GTTCTACCGAACTGAACTAAGAGCGCTTTCAAAAAGAAAACCCTTTTCTACTCCTAACGT

GTCTCACGTACGTATAGTATCCACAAATTCAAGTTATACCCACTTTAATGGATCTCCTCG

CTACTGCCCATAAAGAAGAAAGAAGTAATAGGTAGGGATGACAGGATTTGAACCTGTGAC

ATTTTGTACCCAAAACAAACGCGCTACCAAGCTGCGCTACATCCCTTTTCCAAATTGTTG

TATAATGGCATTGTACACAATTCCTGTCTTGTTTTCCACATCGTAATTTTCTTCTCTTTC

TCTATCTATATAGAACCTTCTTGTCATTTCTTCTTTTTGGTCTCATATAATCAAGGAATG

GTATACATCTAAATCCTATCTAATTTCACCTATAAAAGAAAGATTACTATTCCTTGGTAA

TGTATAGGAAGGGGTCTTTTTAGTTAGGAATTTCGCCTAAACAAAAGAAATACAAATGAT

CTTGGGCAAGAGTATCTGATCATATATGTATTCCAATAAGGAAGGAGGATTTTCAATGCG

GGATATAAAAACATATCTCTCTGTAGCGCCCGTGCTAAGTACTCTATGGTTTGGGGCTTT

AGCAGGTTTATTGATAGAAATTAATCGTTTATTCCCAGATGCTTTGTCATTCCCTTTTTT

TTAATTCTAGTTATTGCTATGCGAGGAATTCTTCGTGACATGACGCAAATTTTCCCTTTT

TCAATCTTTTTTATAGGAAGGAAAAAGAAAGAAAAGATGGATAAGGATTGTATTCTTTAA
[truncated: 4,423,041 more chars]
