# Supplementary material for: Straight From the Plastome: Molecular Phylogeny and Morphological Evolution of Fargesia (Bambusoideae: Poaceae)
Source: Front Plant Sci. 2019 Aug 6;10:981. doi: 10.3389/fpls.2019.00981 (PMC6691181; doi:10.3389/fpls.2019.00981)
Supplement: TABLE S4 — The results of substitution saturation tests implemented in DAMBE. [file Table_4.DOCX]

Table S4. The test of substitution saturation in DAMBE

| **Data sets** | **NumOTU** | **Iss** | **Iss.cSym** | **T** | **DF** | **P** | **Iss.cAsym** | **T** | **DF** | **P** |
| --- | --- | --- | --- | --- | --- | --- | --- | --- | --- | --- |
| **Complete plastome sequences** | 4 | 0.008 | 0.860 | 3838.438 | 108514 | .0000 | 0.856 | 3818.800 | 108514 | .0000 |
|  | 8 | 0.007 | 0.573 | 3787.390 | 108514 | .0000 | 0.783 | 3455.8021 | 108514 | .0000 |
|  | 16 | 0.008 | 0.846 | 3681.398 | 108514 | .0000 | 0.705 | 3064.704 | 108514 | .0000 |
|  | 32 | 0.009 | 0.820 | 3957.484 | 108514 | .0000 | 0.603 | 2900.7741 | 108514 | .0000 |
| **Coding** | 4 | 0.005 | 0.859 | 3491.404 | 62272 | .0000 | 0.855 | 3474.818 | 62272 | .0000 |
|  | 8 | 0.005 | 0.856 | 3417.846 | 62272 | .0000 | 0.782 | 3117.241 | 62272 | .0000 |
|  | 16 | 0.006 | 0.844 | 3302.483 | 62272 | .0000 | 0.704 | 2751.267 | 62272 | .0000 |
|  | 32 | 0.006 | 0.818 | 3551.536 | 62272 | .0000 | 0.600 | 2597.562 | 62272 | .0000 |
| **Noncoding** | 4 | 0.011 | 0.859 | 2132.234 | 48161 | .0000 | 0.854 | 2120.809 | 48161 | .0000 |
|  | 8 | 0.010 | 0.855 | 2124.676 | 48161 | .0000 | 0.780 | 1934.496 | 48161 | .0000 |
|  | 16 | 0.012 | 0.844 | 2069.733 | 48161 | .0000 | 0.702 | 1715.347 | 48161 | .0000 |
|  | 32 | 0.013 | 0.818 | 2225.919 | 48161 | .0000 | 0.598 | 1616.714 | 48161 | .0000 |
| **LSC** | 4 | 0.009 | 0.859 | 2986.346 | 76309 | .0000 | 0.855 | 2972.119 | 76309 | .0000 |
|  | 8 | 0.009 | 0.857 | 2953.539 | 76309 | .0000 | 0.782 | 2693.778 | 76309 | .0000 |
|  | 16 | 0.010 | 0.844 | 2872.664 | 76309 | .0000 | 0.705 | 2392.575 | 76309 | .0000 |
|  | 32 | 0.010 | 0.819 | 3091.812 | 76309 | .0000 | 0.602 | 2261.072 | 76309 | .0000 |
| **SSC** | 4 | 0.010 | 0.858 | 1112.270 | 11821 | .0000 | 0.847 | 1096.758 | 11821 | .0000 |
|  | 8 | 0.010 | 0.845 | 1061.888 | 11821 | .0000 | 0.761 | 955.996 | 11821 | .0000 |
|  | 16 | 0.012 | 0.851 | 1050.930 | 11821 | .0000 | 0.675 | 830.795 | 11821 | .0000 |
|  | 32 | 0.012 | 0.818 | 1117.580 | 11821 | .0000 | 0.572 | 776.258 | 11821 | .0000 |
| **IR** | 4 | 0.002 | 0.861 | 3214.450 | 20609 | .0000 | 0.849 | 3170.300 | 20609 | .0000 |
|  | 8 | 0.002 | 0.846 | 3093.338 | 20609 | .0000 | 0.763 | 2787.184 | 20609 | .0000 |
|  | 16 | 0.003 | 0.853 | 3011.250 | 20609 | .0000 | 0.678 | 2392.226 | 20609 | .0000 |
|  | 32 | 0.003 | 0.820 | 3238.864 | 20609 | .0000 | 0.578 | 2281.442 | 20609 | .0000 |
