# Supplementary material for: Straight From the Plastome: Molecular Phylogeny and Morphological Evolution of Fargesia (Bambusoideae: Poaceae)
Source: Front Plant Sci. 2019 Aug 6;10:981. doi: 10.3389/fpls.2019.00981 (PMC6691181; doi:10.3389/fpls.2019.00981)
Supplement: TABLE S5 — Comparison of partitioning strategies used for the data sets implemented in PartitionFinder v.1.1.1 (Lanfear et al., 2012). [file Table_5.DOCX]

Table S5. Comparison of partitioning strategies used for the data sets implemented in PartitionFinder v.1.1.1 (Lanfear et al., 2012)

| Partitioning strategy | Description | Comparison with PartitionFinder | | | | |
| --- | --- | --- | --- | --- | --- | --- |
|  |  | parameters | lnL | AIC | AICc | BIC |
| all | Complete plastome sequences | 93 | -216569.31 | 433324.62 | 433324.78 | 434218.56 |
| partition2 | Coding, noncoding | 186 | -215443.09 | 431258.17 | 431258.80 | 433046.04 |
| partition3 | LSC, SSC, IRs | 276 | -215199.41 | 430950.82 | 430952.20 | 433603.78 |
